# Computational exploration of *cis*-regulatory modules in rhythmic expression data using the “Exploration of Distinctive CREs and CRMs” (EDCC) and “CRM Network Generator” (CNG) programs

**DOI:** 10.1371/journal.pone.0190421

**Published:** 2018-01-03

**Authors:** Pavlos Stephanos Bekiaris, Tobias Tekath, Dorothee Staiger, Selahattin Danisman

**Affiliations:** RNA Biology and Molecular Physiology, Faculty of Biology, Bielefeld University, Bielefeld, Germany; Meiji Daigaku - Ikuta Campus, JAPAN

## Abstract

Understanding the effect of *cis*-regulatory elements (CRE) and clusters of CREs, which are called *cis*-regulatory modules (CRM), in eukaryotic gene expression is a challenge of computational biology. We developed two programs that allow simple, fast and reliable analysis of candidate CREs and CRMs that may affect specific gene expression and that determine positional features between individual CREs within a CRM. The first program, “Exploration of Distinctive CREs and CRMs” (EDCC), correlates candidate CREs and CRMs with specific gene expression patterns. For pairs of CREs, EDCC also determines positional preferences of the single CREs in relation to each other and to the transcriptional start site. The second program, “CRM Network Generator” (CNG), prioritizes these positional preferences using a neural network and thus allows unbiased rating of the positional preferences that were determined by EDCC. We tested these programs with data from a microarray study of circadian gene expression in *Arabidopsis thaliana*. Analyzing more than 1.5 million pairwise CRE combinations, we found 22 candidate combinations, of which several contained known clock promoter elements together with elements that had not been identified as relevant to circadian gene expression before. CNG analysis further identified positional preferences of these CRE pairs, hinting at positional information that may be relevant for circadian gene expression. Future wet lab experiments will have to determine which of these combinations confer daytime specific circadian gene expression.

## Introduction

Temporal and spatial regulation of gene expression is a common process in eukaryotic organisms. Transcription factor-mediated control of gene expression has been studied for decades and involves complex interplays between DNA and proteins. Transcription factors bind to CREs, i.e. short sequences that are usually situated upstream of coding sequences, and affect the set-up of the transcriptional machinery. Today large numbers of CREs are known, e.g. in humans [1,2], yeast [3], and plants [4,5]. However, CREs not only function as single elements, they also combine with other CREs. The sum of all CREs that convey specific gene expression is called *cis*-regulatory module (CRM) [6]. The gene expression patterns regulated by CRMs are highly dependent on the composition of these CRMs, i.e. the number of repeats of a specific CRE [7], the combination of CREs present [8], the spacing between CREs [9,10], and the CREs’ positions within the CRM [9,10]. In plants, CRMs control the expression of genes that are involved in the cell cycle, photosynthesis, development of the male germline, stress response, and circadian gene expression [5,11–13].

Circadian gene expression denotes rhythmic expression of a gene that follows a rhythm of about 24 hours (from ‘circa diem’ = ‘about a day’). The circadian clock, a biological timekeeper that consists of proteins controlling each other in regulatory feedback loops, maintains this rhythm even under free-running conditions, i.e. when there is no external rhythm, a *Zeitgeber*, indicating the begin of a day. In Arabidopsis, up to 90% of all genes display rhythmic behavior under at least one light/temperature regime [14]. Rhythmic expression under free-running conditions has been shown in up to 36% of all genes [15], covering a plethora of physiological processes including photosynthesis [16], starch metabolism [17], growth [18], flowering time determination [19,20] and regulation of the plant immune system [21,22].

Several CREs are known to confer circadian gene expression. The evening element (AAAATATCT) was identified based on its over-representation in circadianly regulated genes that exhibited maximum expression in the subjective evening [23,24]. The morning element (AAAAAATCT) was identified in a mutational analysis of the *PSEUDO-RESPONSE REGULATOR 5* promoter, a clock gene that is involved in repression of the core clock genes *CIRCADIAN CLOCK ASSOCIATED 1 (CCA1)* and *LATE ELONGATED HYPOCOTYL* during the day [25,26]. Michael and colleagues conducted bioinformatics analyses of microarray experiments in which Arabidopsis was subjected to 11 different rhythmic conditions (e.g. photocycles, thermocycles, short days, long days). Here they identified the protein box (ATGGGCC), the telobox (AAACCCTT) and the starch box (AAGCCC) elements as CREs that confer midnight-specific gene expression and that are conserved between Arabidopsis, rice and poplar [14]. The so-called Hormone-up-in-Dawn (HUD) element (CACATG) was found to be over-represented in genes that respond to brassinosteroid and auxin treatments and in genes that are expressed preferentially at dawn [27].

The identification of CREs that correlate with specific gene expression has long been established [28–31]. For example, Bussemaker and colleagues detected new regulatory motifs in the upstream regions of genes by correlating the presence of these motifs with genome-wide gene expression in *Saccharomyces cerevisiae* [28]. Another tool, called *‘in silico* expression analysis’, determines which genes contain a given CRE and compares the expression of these genes in microarray data [31]. With the help of this program, the authors were able to determine that a CGACTTTT sequence was involved in the response of Arabidopsis to infection with the fungus *Botrytis cinerea* [31]. In another approach, the MEME suite [30] was used to detect over-represented CREs in rhythmically expressed genes and further gene expression profiles were compared with a neural network approach [32]. The respective calculations were so computation intensive that a supercomputer was used for this study [32]. Most programs focus on the detection and analysis of single CREs, although it is long established that CREs affect gene expression in a combinatorial manner. Studies to identify and analyze CRMs are less straightforward. For this, Hidden Markov models have been successfully used in simulated and real data sets of fruitflies and humans [29]. Also, Hidden Markov Models have been used to identify CRMs by analyzing correlations between binding sites and multispecies comparisons in yeast and fruitfly experimental data [33]. CRMs were further detected using position weight matrices [34,35], Monte Carlo methods [36], phylogenetic approaches [37], and chromatin signatures and neural networks, respectively [38].

We propose a simpler approach to determine candidate CREs and CRMs that may confer specific gene expression. This approach reliably analyzes the potential of millions of CRMs in a relatively short time. It uses programs that run on a table-top computer and can be used by users with minimal bioinformatics knowledge. These two programs are called “Exploration of Distinctive CREs and CRMs” (EDCC) and “CRM Network Generator” (CNG). EDCC correlates the presence and positions of known CREs/CRMs with gene expression data, and CNG further assesses the importance of positional features within CRMs that were determined by EDCC. We tested the performance of these programs using data from a circadian microarray experiment of *Arabidopsis thaliana* seedlings [14]. EDCC identified both known and candidate CREs and CRMs in circadian gene expression control. CNG analysis shows that some of the identified CRE pairs occur at specific locations in the promoters of downstream genes, indicating functional CRMs in circadian gene expression.

## Results

### Principle of EDCC analysis

**Fig 1:**
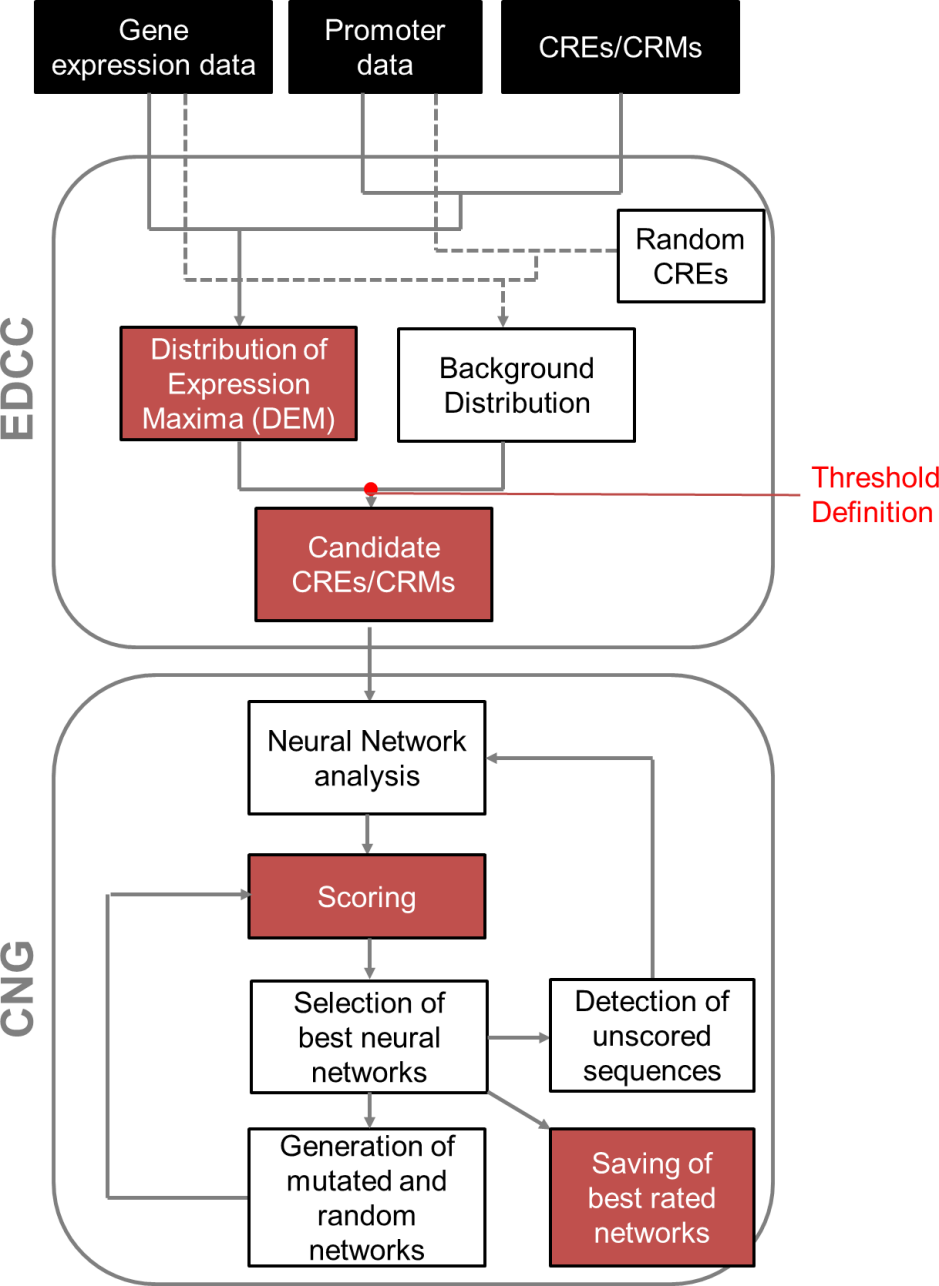
Flowchart of EDCC and CNG analyses. The flowchart shows which data input is needed for EDCC and CNG analyses, the principle behind their functions and the outputs of the two programs. Further detailed graphics explain the calculations of EDCC and CNG in Supporting figures S1 and S2 Figs, respectively.

We designed two programs to analyze whether user-determined CREs and CRMs correlate with specific expression patterns and thus, whether they may be involved in regulation of the specific gene expression (Fig 1). The first program, EDCC, correlates candidate CREs and CRMs with gene expression patterns, and compares this with the expression pattern of all genes under different experimental conditions. For pairs of CREs, EDCC further determines whether they are positioned at a specific distance to each other, whether they are positioned in a specific order towards the transcriptional start site (TSS), and whether the two CREs are positioned at a specific distance to the TSS.

**Fig 2:**
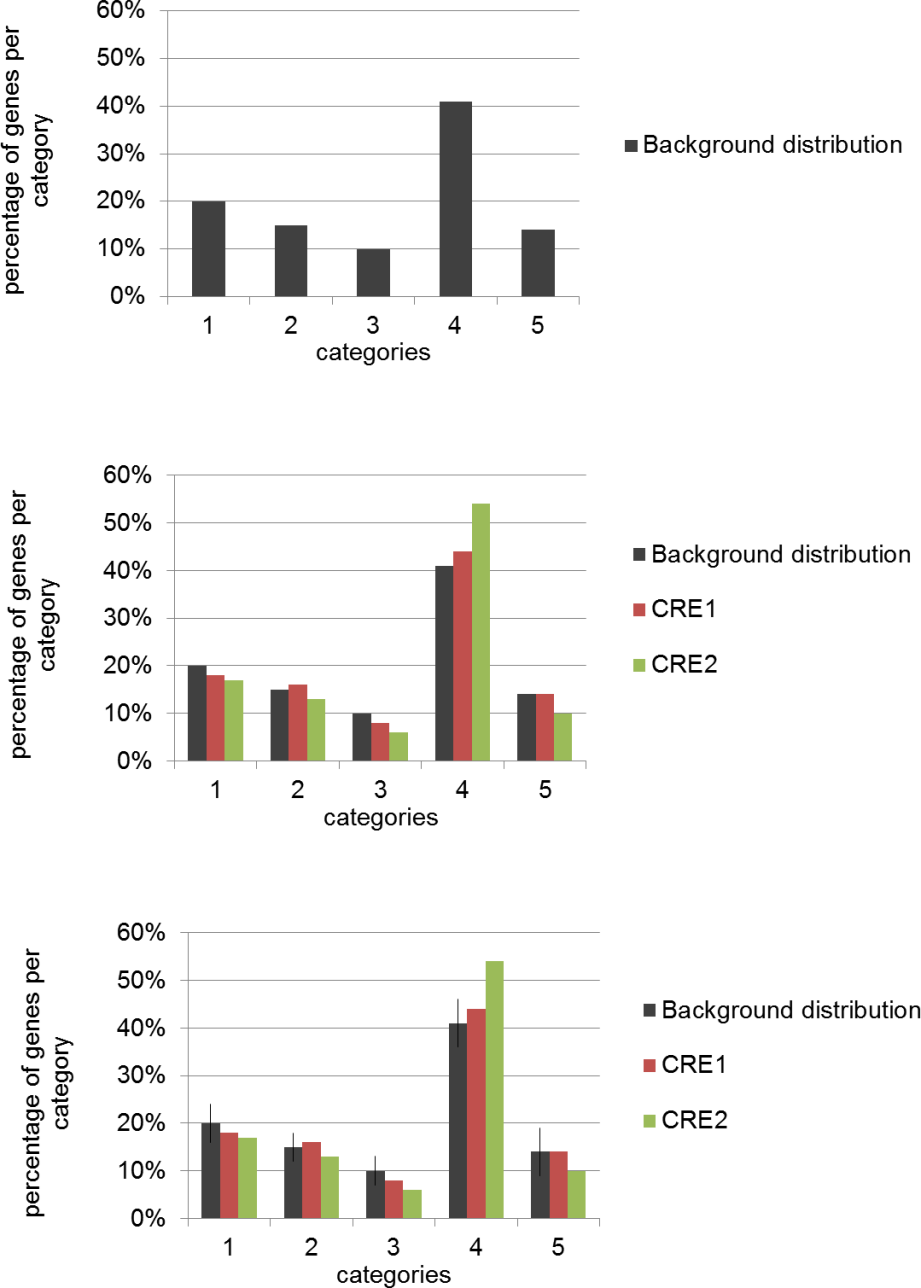
Principle of EDCC analysis. A) Presented is the background distribution of gene expression across five generic categories. B) DEM of two exemplary CREs compared to the background distribution. Genes containing CRE1 (red) do not correlate with a shift in the DEM, whereas genes containing CRE 2 (green) do. C) Addition of standard deviations after analysis of random CREs allows establishing thresholds for the determination of candidate CREs.

EDCC uses three initial data sets: gene expression data, promoter sequences of the respective genes, and a list of CREs and CRMs defined by the user. The gene expression data needs to be categorized over the different treatments that the user wants to analyze. Only genes that are differentially expressed between treatments will be included in the analysis. EDCC categorizes each gene according to its maximum gene expression, and each gene is categorized in only one condition. EDCC then plots the percentage of genes per category, which results in the background distribution (Fig 2A). Queried with a CRE/CRM, EDCC determines the promoters that contain the motifs and the expression category that the respective genes belong to. EDCC then plots the percentage of genes that contain the CRE/CRM per category, resulting in a distribution of expression maxima (DEM) which is specific for each given CRE/CRM (Fig 2B). This DEM is then compared to the background distribution. A CRE/CRM that has no effect on gene expression in the analyzed conditions should lead to a DEM that is similar to the background distribution (Fig 2B). Inversely, a CRE/CRM that affects genes towards expression under a specific treatment or condition should lead to a shift between the DEM and the background distribution (Fig 2B). EDCC determines a threshold at which a CRE/CRM is identified as a candidate by calculating the DEMs of a large number of random CREs and determining the standard deviation from the mean for each category. A CRE that correlates with a DEM that differs from the background by at least one standard deviation in one or more conditions is identified as a candidate CRE (Fig 2C). EDCC also allows for more conservative approaches by increasing the threshold to a multifold of the standard deviation.

### Testing EDCC on circadian microarray data

**Fig 3:**
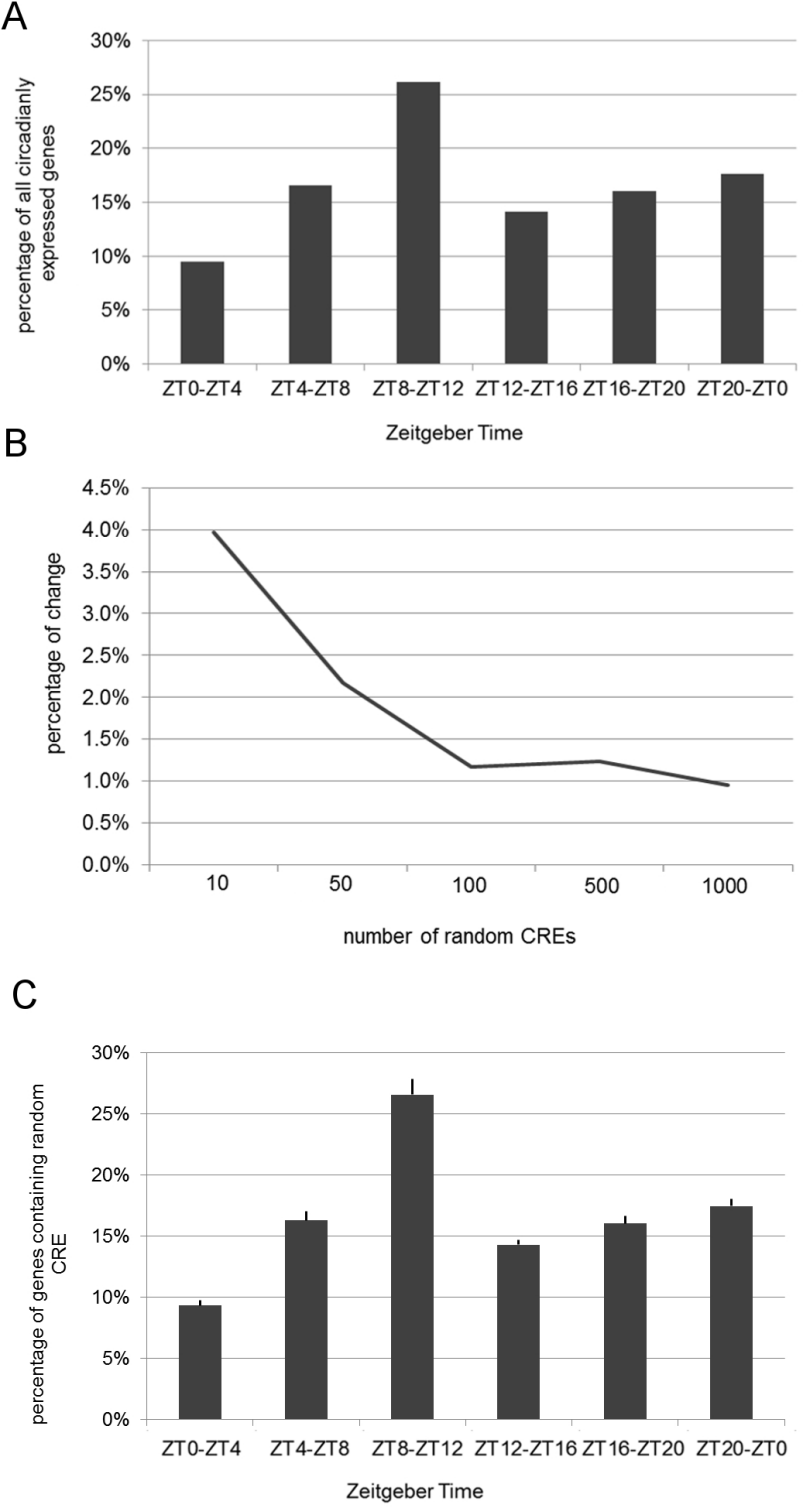
EDCC analysis of circadian microarray data. A) Distribution of maximum gene expression times of circadianly genes expressed in Arabidopsis seedlings [14], which was used as background distribution for the EDCC analysis. Distribution is shown as percentage of all circadianly expressed genes. Maxima are categorized in six categories, i.e. ZT0-ZT4 (morning), ZT4-ZT8 (midday), ZT8-ZT12 (evening), ZT12-ZT16 (early night), ZT16-ZT20 (midnight), ZT20-ZT0 (before dawn). B) Decrease of standard deviations of randomized CREs in percent plotted against the number of randomized CREs used (10, 50, 100, 500, and 1000 random CREs, respectively). C) Mean DEM of random CREs after 100 iterations, including standard deviations.

We tested EDCC with data of a circadian microarray experiment, in which Arabidopsis seedlings were entrained for nine days in a 12 h dark/12 h light cycle and then transferred into continuous light [14]. Seedlings were harvested every four hours for 48 hours, beginning at Zeitgeber Time 0 (ZT0), i.e. the hour at which the lights are switched on. Gene expression for each time point was determined using an Affymetrix *Arabidopsis* ATH1 gene chip (E-MEXP-1304) [14]. We identified circadianly expressed genes using ARSER [39] and categorized the genes into six categories according to the respective peak expression times. We found that 3561 genes (10% of the TAIR10 genome annotation) were expressed circadianly under these experimental conditions. A majority of these exhibited peak expression between ZT8 and ZT12 (26%), i.e. before the subjective dusk (Fig 3A). This was followed by the category ZT20-ZT0 (18%), i.e. just before dawn, with all other categories exhibiting lower percentages (Fig 3A). This background distribution was queried with random CREs of 5–8 bp lengths to determine the standard deviation and hence the threshold for further EDCC analyses. To test the optimum number of CREs for background models, we queried EDCC with 10, 50, 100, 500, and 1000 random CREs, respectively, and analyzed the difference between the background distribution and the DEM of the randomized CREs. This difference decreased with a higher number of queries (Fig 3B). As the difference between 100 and 1000 queries was negligible, we decided to further use 100 random CREs to determine EDCC thresholds (Fig 3C).

### Testing EDCC with known circadian clock CREs

**Fig 4:**
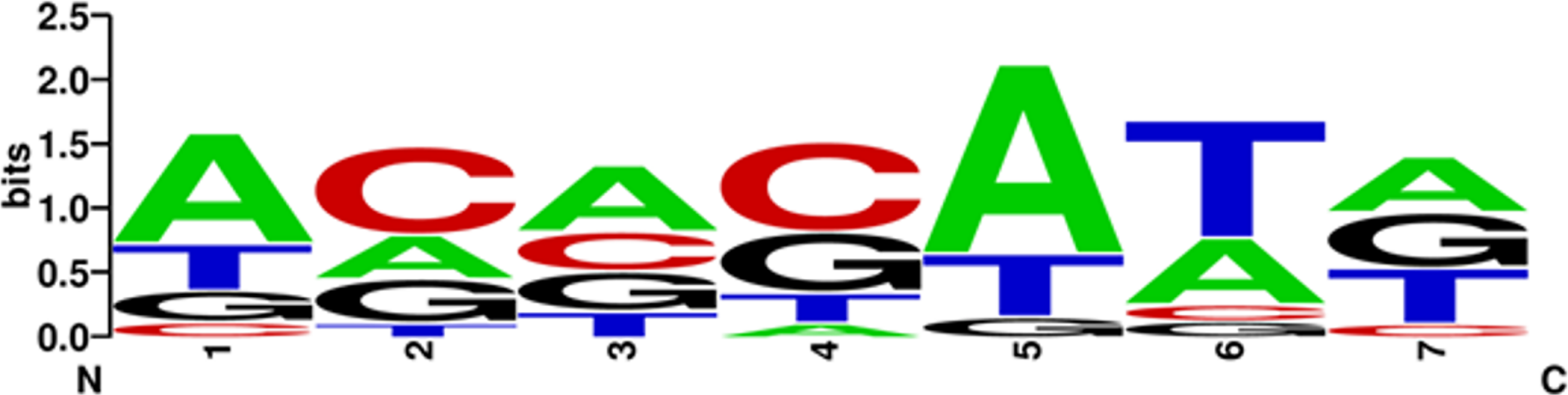
DEM for genes containing the evening and telobox elements compared to randomized background. Shaded areas indicate one to three standard deviations distance from the background.

After having established a random background with thresholds for the circadian microarray experiment, we tested CREs that are known to confer circadian gene expression, i.e. the evening element, the morning element, the three midnight elements and the HUD-domain [14,23,25,27]. Genes containing the evening element and the telobox element (AAACCCTT) exhibited DEMs that differed from the background at ZT8-12 (evening) and ZT16-20 (midnight), respectively (Fig 4). As the evening element indeed confers evening specific gene expression [23], this indicates that EDCC is able to correctly identify CREs that may be involved in circadian gene expression and the time point that is affected by the CRE. The evening element is marked “candidate” in the EDCC analysis even when using a threshold of three standard deviations, correctly indicating the strength of the evening element as a CRE conferring evening specific circadian gene expression. EDCC also correctly identifies the telobox element as a CRE that confers midnight specific gene expression between ZT16 and ZT20 [14]. All other tested CREs were not indicated as candidates by EDCC, which means that the EDCC analysis is more conservative than other types of analysis.

### Testing EDCC performance with 1755 single CREs

We then tested EDCC performance using 1755 CREs that are known in plants [5]. We only counted CREs that were present in at least 10 promoters to prevent a false positive effect on the DEM. We ran the analysis five times and found 182.8 candidate CREs on average, i.e. 10.4% of all queries were identified as candidate CREs for at least one time point (S1 Table). Although EDCC creates a new random background in each run, 98% of the CREs that were found overlapped in all five iterations. We also calculated the quartile dispersion coefficient [40] and found a 0.27% variation between runs, indicating that the results generated by EDCC are extremely consistent.

**Fig 5:**
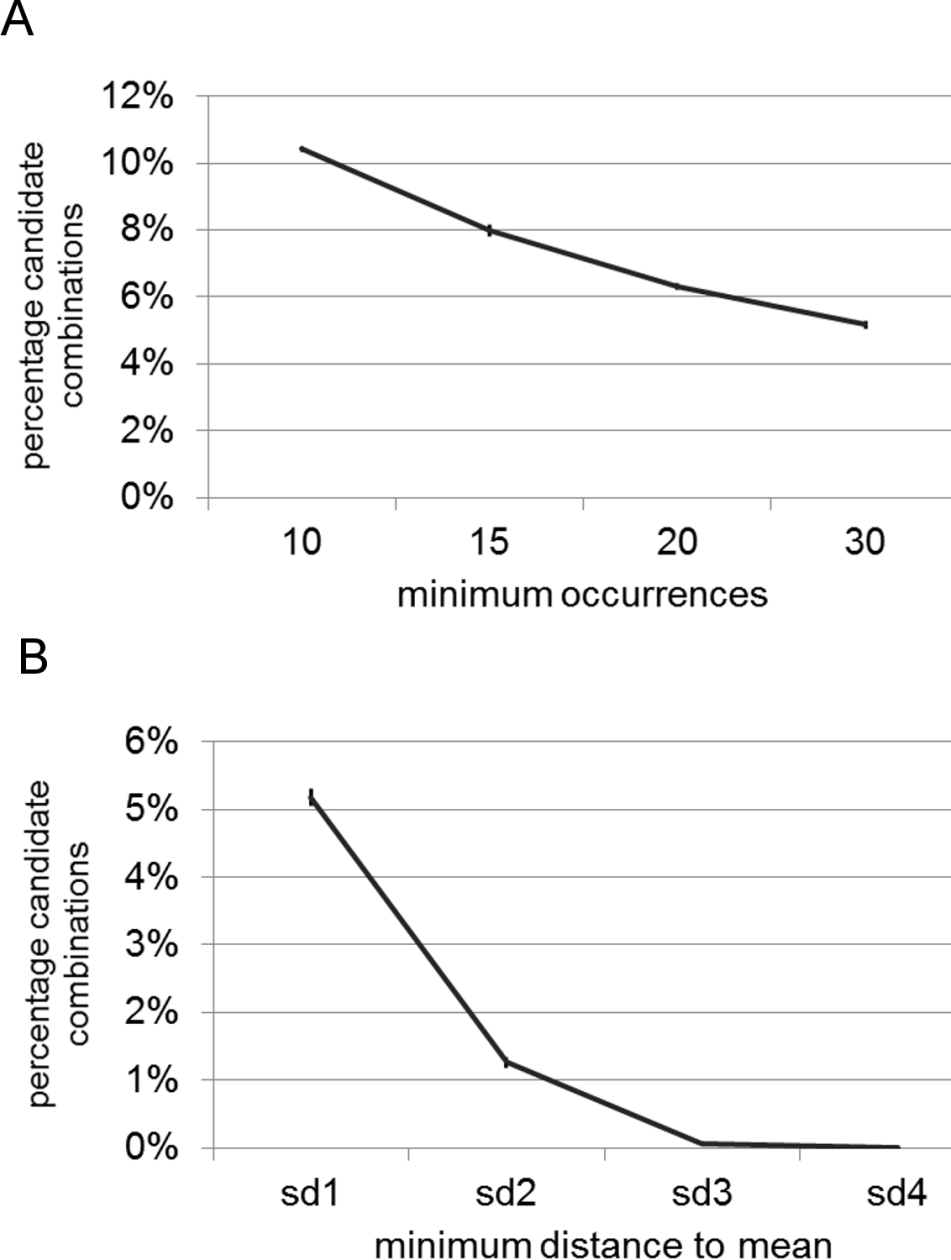
Number of candidate CREs under different EDCC settings. A) Graph depicting the decrease of candidate CREs when increasing the minimum number of promoters a CRE has to be present in. B) Graph depicting the decrease of candidate CREs when increasing the thresholds from one to four standard deviations (sd).

We then ran the same test under more conservative conditions. In the first approach, we increased the number of promoters that a CRE must be present in to 15, 20, and 30, respectively, and ran each test five times. This led to smaller numbers of candidate CREs (Fig 5A; S2 Table). In each case, the overlap among the five iterations of the analysis was large, i.e. 98%, 100%, and 100%, respectively. In the second approach, we increased the threshold to two, three or four standard deviations, respectively. This dramatically reduced the number of candidate CREs (Fig 5B). Also here, we found a high overlap between the individual runs. At a distance of minimum three standard deviations, we found only one consistent candidate CRE: GACGTGTA, which has been described as an abscisic acid (ABRE) binding response element [41]. The list of CREs that were found to be candidates in all five analyses with a threshold of two standard deviations is given in Table 1. Non-surprisingly, the evening element was one of the candidates that were identified by the EDCC analysis. Further candidate elements that have been found are involved in light-controlled or circadian gene expression, e.g. MYB transcription factor binding sites, which are involved in the light responsiveness of enzymes of the flavonol biosynthetic pathway in Arabidopsis [42], and GATA and G box motifs, which belong to the earliest promoter elements found in light-regulated and circadian clock regulated genes [43,44]. Also a binding site for TCP transcription factors was found (Table 1). These transcription factors have recently been shown to bind to clock genes and affect their expression [45–47]. Abscisic acid (ABA) response elements, which are similar to the G box, have been found several times by the EDCC analysis (Table 1). ABA signaling has been found to be connected to the circadian clock in several studies [48–50]. In case of non-annotated CREs, we used agriGO to determine the enrichment of gene ontology (GO) terms [51] (Table 1).

**Table 1:** Single CREs that were identified as candidates with a threshold of at least two standard deviations.

single sequence	interesting timepoints	sum of matches	Annotation or agriGO enrichment	Reference
AAAATATCT	ZT8-ZT12	267	evening element	[23]
AACCTACC	ZT20-ZT0	63	MYB binding site promoter	[52]
AATATTTTTATT	ZT4-ZT8	36	AT1BOX AT-1 box (AT-rich element)	[43,53,54]
AAWGTATCSA	ZT20-ZT24	32	Wound-responsive element	[55]
ATCCAACC	ZT4-ZT8	81	MYB1 binding site motif	[56]
ATCCTACC	ZT16-20, ZT20-ZT24	33	MYB1 binding site motif	[56]
CAATGATTG	ZT8-12, ZT16-ZT20	35	ATHB5 binding site motif	[57]
CACCTACC	ZT8-ZT12, ZT20-ZT0	42	MYB1 binding site motif	[56]
CACGCAAT	ZT8-ZT12	33	Sequence found in auxin responsive genes of Soybean	[58]
CAGAAGATA	ZT16-ZT20	44	GATA motif binding factor	[59]
CCAGGTGG	ZT16-ZT20	38	Class I TCP binding site in rice	[60]
GACGTGTA	ZT16-ZT20	48	ABRE-like binding site motif	[41]
GATGAYRTGG	ZT12-ZT16	39	opaque-2 binding site of maize b-32 type I ribosome-inactivating protein gene	[61]
GCGGCAAA	ZT16-ZT20	37	E2F binding site in tobacco Ribonucleotide reductase gene promoter	[62]
MAGGTAAGT	ZT8-ZT12	56	*cis*-element in exon-intron splice junctions of plant introns	[63]
MCACGTGGC	ZT4-ZT8	80	G box/Conserved sequence upstream of light-regulated genes	[64]
NCCCGCCA	ZT16-ZT20	68	enriched in GOs DNA replication, DNA-dependent DNA replication, DNA metabolism etc	
TAACTCGTT	ZT4-ZT8, ZT8-ZT12, ZT16-ZT20, ZT0-ZT4	32	MYB2 binding site motif	[65]
TAACTGGTT	ZT12-ZT16	55	MYB2 binding site motif	[65]
TACGTGGA	ZT4-ZT8	63	ABRE-like binding site motif	[41]
TACGTGTC	ZT16-ZT20	59	ABRE-like sequence found in rice	[66]

Annotations are from AtCOEcis [5], alternatively enriched GO terms according to agriGO [51] are given.

### Analysis of pairwise CRE combinations

**Fig 6:**
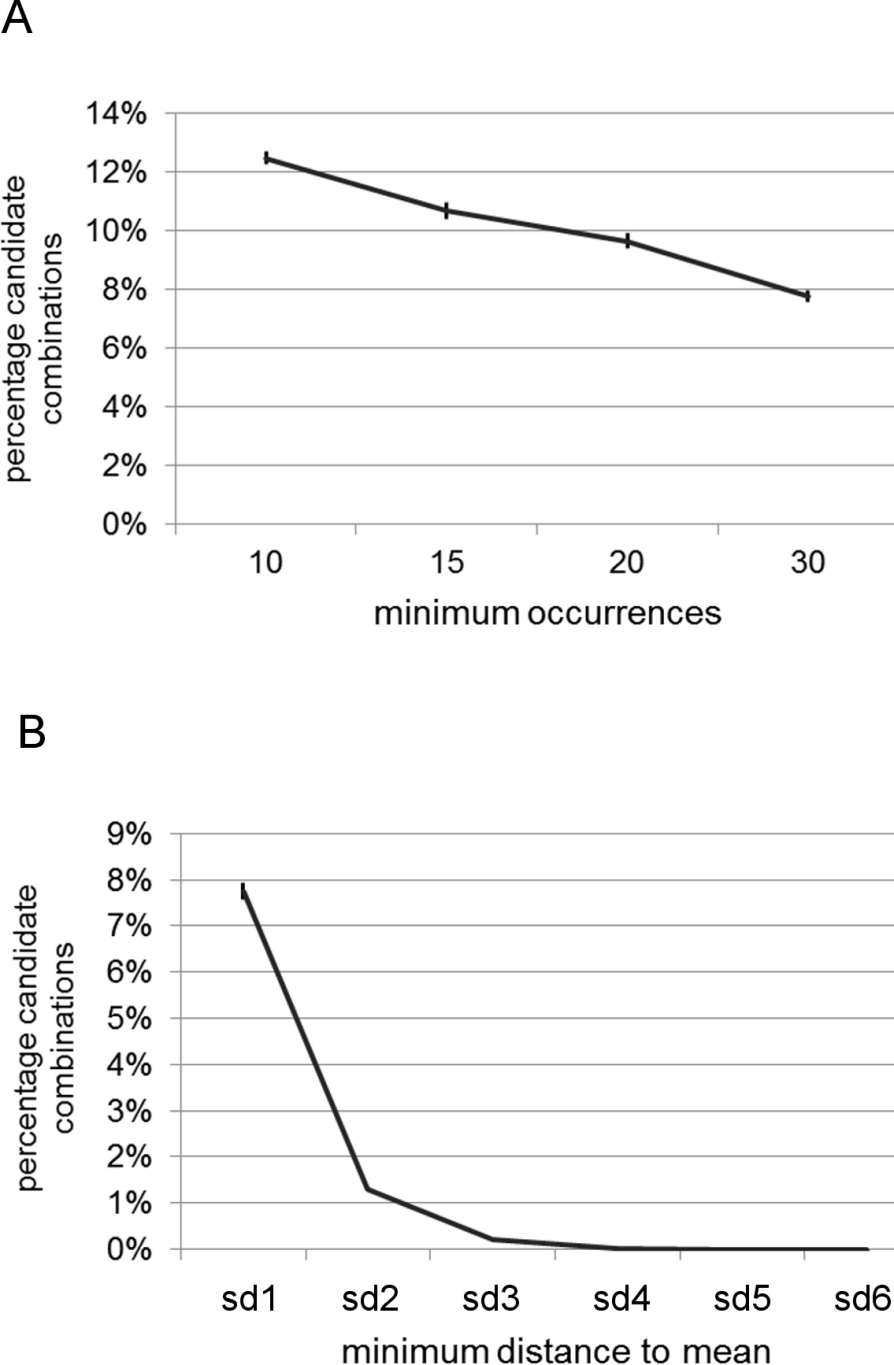
Analysis of pairwise CRE correlation with circadian gene expression. A) Graph depicting the decrease in candidate CRMs when increasing minimum number of promoters the CRM must be present in. B) Graph depicting the decrease of candidate CRMs with increasing thresholds (sd: multifold of standard deviation from background).

We then analyzed the simplest type of CRMs: pairwise combinations of CREs. Here, we combined each of the 1755 CREs with each other, leading to 1,540,890 tested combinations, including homotypic combinations. Analogous to the tests with single CREs, we first estimated the conditions under which the test needed to be conducted. Under the least conservative conditions (10 occurrences, one standard deviation threshold), we found on average 192,010.6 candidate combinations (12.46% of all combinations). Increasing the number of minimum occurrences to 15, 20 and 30 led to a decrease of candidate CRMs analogous to the case in single CREs (Fig 6A). A stepwise increase of the threshold distance from the background from one to six standard deviations led to a strong decrease in the number of candidate CRMs, respectively (Fig 6B). Under the most restrictive conditions—at minimum six standard deviations and minimum 30 hits in promoters—only one combination remained: the evening element together with a Dc3 Promoter-Binding Factor-1 and 2 (DPBF1&2) element, which first has been described as an ABA responsive element in the promoter of the carrot *Dc3* gene [67].

21 candidate CRMs were found with a threshold of five standard deviations and a minimum occurrence of 30 promoters in all five repetitions of the EDCC analysis (Table 2). The evening element was present in six candidate CRMs. The evening element was found in combinations with the LEAFY consensus site motif [68], the DPBF1&2 binding site motif described above, an undefined motif (AATNCCNC), elements that were found in genes that are involved in glucosyltransferase activity (ATGGCNNC), calmodulin regulated protein kinase activity and ATPase activity (GAANGAGA), and in auxin signaling (ACACATG), respectively (Table 2). Other candidate CRE combinations contained G boxes together with an element that is overrepresented in metal homeostasis genes, and the ABRE-like motif (GACGTGTA) together with an undefined motif (CNANAGAA). Also here, unannotated CREs were subjected to GO term analysis using agriGO [51].

**Table 2:** Candidate CRMs in circadianly expressed genes.

no.	sequence combination	interesting timepoints	sum of matches	annotation+citation element 1	annotation+citation element 2
1	AAAATATCT, ATGGCNNC	ZT8-ZT12	31	evening element [23]	enriched in GO glucosyltransferase activity
2	AAAATATCT, CCAGTG	ZT8-ZT12	38	evening element [23]	LFY consensus binding site motif [68]
3	AAAATATCT, GAANGAGA	ZT8-ZT12	56	evening element [23]	enriched in GO calmodulin regulated protein kinase activity and ATPase activity
4	AATNCCNC, AAAATATCT	ZT8-ZT12	52	undefined	evening element [23]
5	ACACATG, AAAATATCT	ZT8-ZT12	30	DPBF1&2 binding site motif [67]	evening element [23]
6	ACACCGG, AAGNGTNG	ZT12-ZT16	30	DPBF1&2 binding site motif [67]	enriched in GO calmodulin regulated protein kinase activity
7	ACANTACN, ATCCAACC	ZT4-ZT8	44	undefined	MYB1 binding site motif [52]
8	ACANTACN, MCACGTGGC	ZT4-ZT8	34	undefined	G box; Conserved sequence upstream of light-regulated genes [64]
9	AGNGATAN, MCACGTGGC	ZT4-ZT8	33	enriched in metal ion homeostasis	G box; Conserved sequence upstream of light-regulated genes [64]
10	ANCACATG, AAAATATCT	ZT8-ZT12	36	enriched in auxin stimulus	evening element [23]
11	ATACGTGT, TAACAAA	ZT0-ZT4	40	Z-DNA-forming sequence found in the Arabidopsis chlorophyll a/b binding protein gene (cab1) promoter; Involved in light-dependent developmental expression of the gene [69]	MYBGAHV Central element of gibberellin (GA) response complex (GARC) in high-pI alpha-amylase gene in barley (H.v.) [70]
12	ATGNTTCA, ACGTGGC	ZT16-ZT20	39	enriched in GO protein serine/threonine kinase activity	enriched in GO glucan biosynthesis, chloroplast part
13	CATGCATG, AGNAACAA	ZT4-ZT8	34	RY-repeat motif; Binding site of FUS3; TRAB1, bZIP transcription factor, interacts with VP1 and mediates ABA-induced transcription [71]	n/a
14	CATGCATG, NGCNTGAA	ZT4-ZT8	30	RY-repeat motif; Binding site of FUS3; TRAB1, bZIP transcription factor, interacts with VP1 and mediates ABA-induced transcription [71]	n/a
15	CCNNCACN, GTGATCAC	ZT0-ZT4	32	n/a	PIATGAPB found in the Arabidopsis thaliana GAPB gene promoter; Mutations resulted in reductions of light-activated gene transcription [72]
16	CNANAGAA, GACGTGTA	ZT16-ZT20	32	n/a	ABRE-like binding site motif [41]
17	CTCATTTN, AGATCCAA	ZT4-ZT8	30	n/a	AG-motif found in the NtMyb2 gene promoter; AGP1binding site [73]
18	GACGTGTA, CNNACANC	ZT16-ZT20	30	ABRE-like binding site motif [41]	n/a
19	TCNTNAGA, CAAAACGC	ZT16-ZT20	31	n/a	CDA1ATCAB2 CDA-1 binding site in DtRE (dark response element) f of chlorophyll a/b-binding protein2 gene in Arabidopsis [74]
20	TGTCACA, TGAGTCA	ZT4-ZT8	31	motif found cucumisin gene promoter in melon fruits [75]	Required for endosperm-specific expression [76]
21	TGTGNGNA, TAGTGGAT	ZT4-ZT8	32	enriched in GO external encapsulating structure organization and biogenesis, cell wall biogenesis	negative regulatory region in promoter region of Brassica napus (B.n.) extA extensin gene [77]

Candidate CRE pairs are present in at least 30 promoters and correlate with DEMs that deviate from the background by at least five standard deviations. Annotations are as given by AtCOEcis [5], alternatively, enriched GO terms according to agriGO [51] are given, n/a depicts CREs without annotation or enriched GO term.

### Mutational analysis of a CRE pair: An example

**Fig 7:**
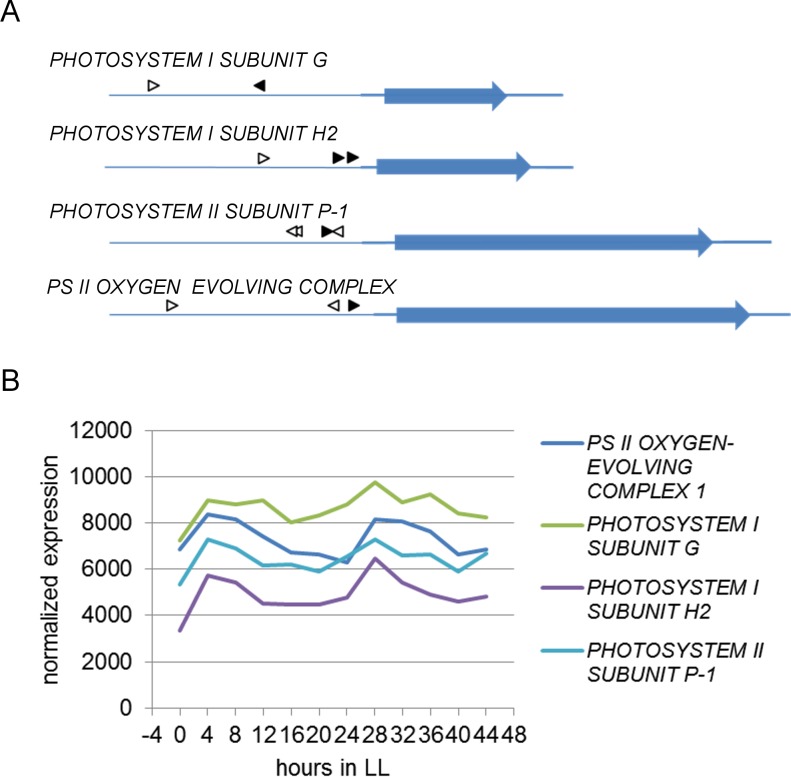
Position weight matrix of nucleotides in the DPBF1&2 element that correlate with a shift in the DEM when combined with the evening element. The size of the letters at each position indicate which bases lead to a decrease in the performance of the CRE pair when mutated prior to the EDCC analysis. That means that changing the adenine on position 5 to any other nucleotide led to a decreased correlation of the mutated CRE pair with time point specific gene expression in almost all cases.

Finding the evening element represented in six of the 21 CRE pairs led us to an interesting question: is it possible that EDCC identifies a CRE pair as a candidate only because one of the two CREs would be identified as a candidate in any case? This might lead to false positive CRE pairs. We tested whether the evening element/DPBF1&2 binding element (ACACATG) pair is specific by generating mutations within both CREs and subjecting these to EDCC analysis. We generated one million unique CRE pairs including 0 to 16 mutations from the original pair each. Of the one million pairs, only 13 pairs performed comparably to the original pair in the EDCC analysis. All other mutant combinations did not correlate with a shift in peak expression times. Of the 13 mutations, none included a mutated evening element, indicating that mutation of the evening element may have a stronger effect on evening-specific gene expression than mutation of the DPBF1&2 element. This also indicated that indeed the evening element may be more important for the specific gene expression conferred by the CRE pair than the DPBF1&2 element. We were further able to determine which nucleotides of the DPBF1&2 element correlated with a better performance in the EDCC analysis, i.e. positions 1, 4, 5 and 6 of the ACACATG sequence (Fig 7). It is however not possible to finally decide whether one of the two elements is irrelevant for a possible function as a CRM without resorting to wetlab experiments, which were beyond the scope of this study.

### Gene ontology analysis of pairwise CRE combinations

The EDCC output includes a list of Arabidopsis Genome Initiative (AGI) identifiers for all those genes that contain a CRE or CRM in their promoters. A GO analysis was conducted with the genes that contain the 21 candidate CRMs [14]. Amongst the biological processes, the sequence combinations no. 3, 8, 9, 11 and 16 were most interesting, as they included processes that are known to be under the control of the circadian clock, i.e. shoot morphogenesis, photosynthesis, the regulation of defense response and the response to light stimuli (Table 3). Interestingly, six of the 21 combinations were enriched in the GO term chloroplast, i.e. the gene products of genes containing these CRE are more often located in the chloroplast than expected.

**Table 3:** Gene ontology (GO) analysis of pairwise CRE combinations.

no.	sequence combination	GO Biological Process	GO Cellular Component
1	AAAATATCT,ATGGCNNC	N/A	plastid, chloroplast, intracellular part, plastid part
2	AAAATATCT,CCAGTG	metabolic process, response to cadmium ion, response to inorganic substance	N/A
3	AAAATATCT,GAANGAGA	shoot morphogenesis, regulation of cellular process	N/A
4	AATNCCNC,AAAATATCT	N/A	N/A
5	ACACATG,AAAATATCT	N/A	N/A
6	ACACCGG,AAGNGTNG	response to stress, glycoside metabolic process,	N/A
7	ACANTACN,ATCCAACC	N/A	N/A
8	ACANTACN,MCACGTGGC	photosynthesis, cystein metabolic process	chloroplast thylakoid membrane
9	AGNGATAN,MCACGTGGC	regulation of defence response	chloroplast part
10	ANCACATG,AAAATATCT	N/A	N/A
11	ATACGTGT,TAACAAA	response to light stimulus, organic acid biosynthetic process	N/A
12	ATGNTTCA,ACGTGGC	cellular carbohydrate metabolic process	N/A
13	CATGCATG,AGNAACAA	N/A	N/A
14	CATGCATG,NGCNTGAA	N/A	cell wall; external encapsulating structure
15	CCNNCACN,GTGATCAC	cellular protein catabolic process	chloroplast thylakoid membrane
16	CNANAGAA,GACGTGTA	response to external stimulus, photosynthesis light reaction, alcohol metabolic process	chloroplast stroma
17	CTCATTTN,AGATCCAA	catalytic activity	N/A
18	GACGTGTA,CNNACANC	cellular protein complex assembly, photosynthesis light reaction, response to external stimulus, cellular amino acid biosynthetic process	chloroplast part
19	TCNTNAGA,CAAAACGC	N/A	N/A
20	TGTCACA,TGAGTCA	ubiquitin-dependent protein catabolic process; ligase activity	N/A
21	TGTGNGNA,TAGTGGAT	N/A	plasma membrane

### Comparison with other approaches

There are few approaches that work similarly to EDCC and CNG. However, circadian gene expression has been subject of earlier studies on CREs and CRMs. In an earlier analysis, Ding and colleagues used a frequent mining pattern [78] based approach to identify sequence combinations that frequently co-occur in Arabidopsis and poplar promoters [79]. We compared the 21 combinations we found to correlate with a shift in the DEM of circadianly expressed genes and compared these with the combinations which were found by Ding and colleagues. Here, we found that 4 out of 21 CRMs are over-represented in Arabidopsis and poplar promoters (Table 4). Note that Ding and colleagues only used CREs from the PLACE database [80], which is a subset of the AtCOEcis database that we used for this study [5]. Thus, it is likely that more combinations that we found in our analysis are over-represented in Arabidopsis promoters.

**Table 4:** Overlap between EDCC analysis and combinations found in an earlier analysis.

Combination found in this study	Combination found by Ding et al.
MCACGTGGC/ACANTACN	MCACGTGGC/CcaTACatt
CATGCATG/AGNAACAA	CATGCATG/gctaAACAAt
CCNCACN/GTGATCAC	CCnnnnnnnnnnnncCACg/GTGATCAC CAAACACC/GTGATCAC
TCNTNAGA/CAAAACGC	TCaTcttctt/CAAAACGC

Partially overlapping CREs contain large and small letters. The large letters indicate nucleotides that are identic between the CRE analysed by EDCC and the CRE analysed by Ding et al [79].

Another study found 10 CREs that correlated with diurnal and circadian gene expression in Arabidopsis [32]. For this they used MEME [30] but as the analysis with MEME is very computation intensive, the authors had to use a supercomputer [32]. We analyzed the 10 CREs they found using EDCC, and identified only CCACGTG as a candidate. EDCC determined that the motif deviates from the background at ZT0-ZT4 (at the start of the day), whereas the authors of the previous study only identified two sets of genes that contained this motif but displaying different expression patterns.

Both comparisons indicate that EDCC may be more conservative than other approaches to correlate gene expression with presence of CREs.

### Analyzing positional attributes of candidate CRE combinations

EDCC determines three positional features between CREs: Over-representation of specific distances between two CREs, the distance of the closest of two CREs to the TSS, and a specific order of the two CREs in respect to the TSS. Depending on the number of identified ‘candidate’ CRE pairs, this leads to a large number of positional features that need to be evaluated by the user. To prevent user-bias, we introduced a neural network generator that categorizes the positional features and allows for unbiased scoring of the data: CNG.

CNG is able to classify a large amount of CRE pairs at once by using two-class neural networks. We used the 21 candidate CRE pairs that were identified in the previous EDCC analysis to perform the CNG analysis (S3 Table). CNG was run eight times resulting in 7.125 networks, respectively.

**Fig 8:**
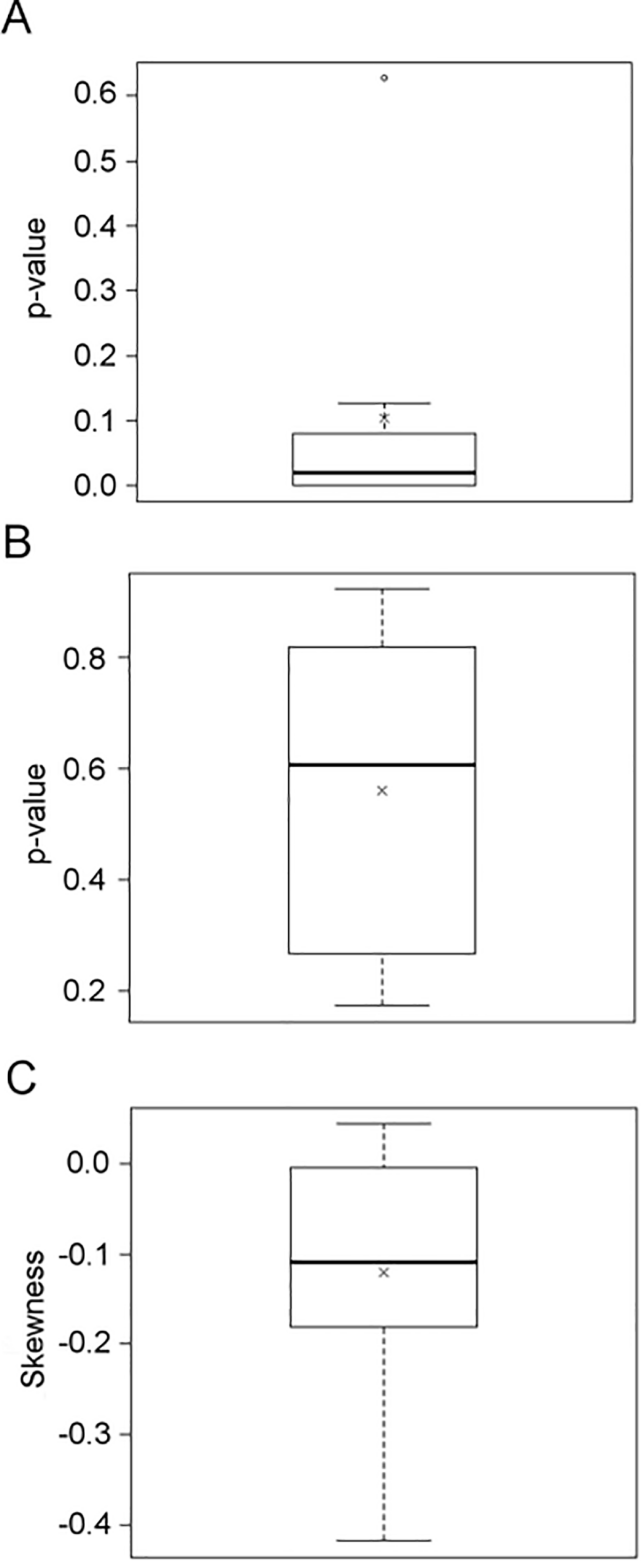
Representative output of the CNG analysis. A) Distribution of p-values for binomial order test. B) Distribution of p-values for distance G-test. C) Distribution of Bowley skewness analysis.

**Fig 9:**
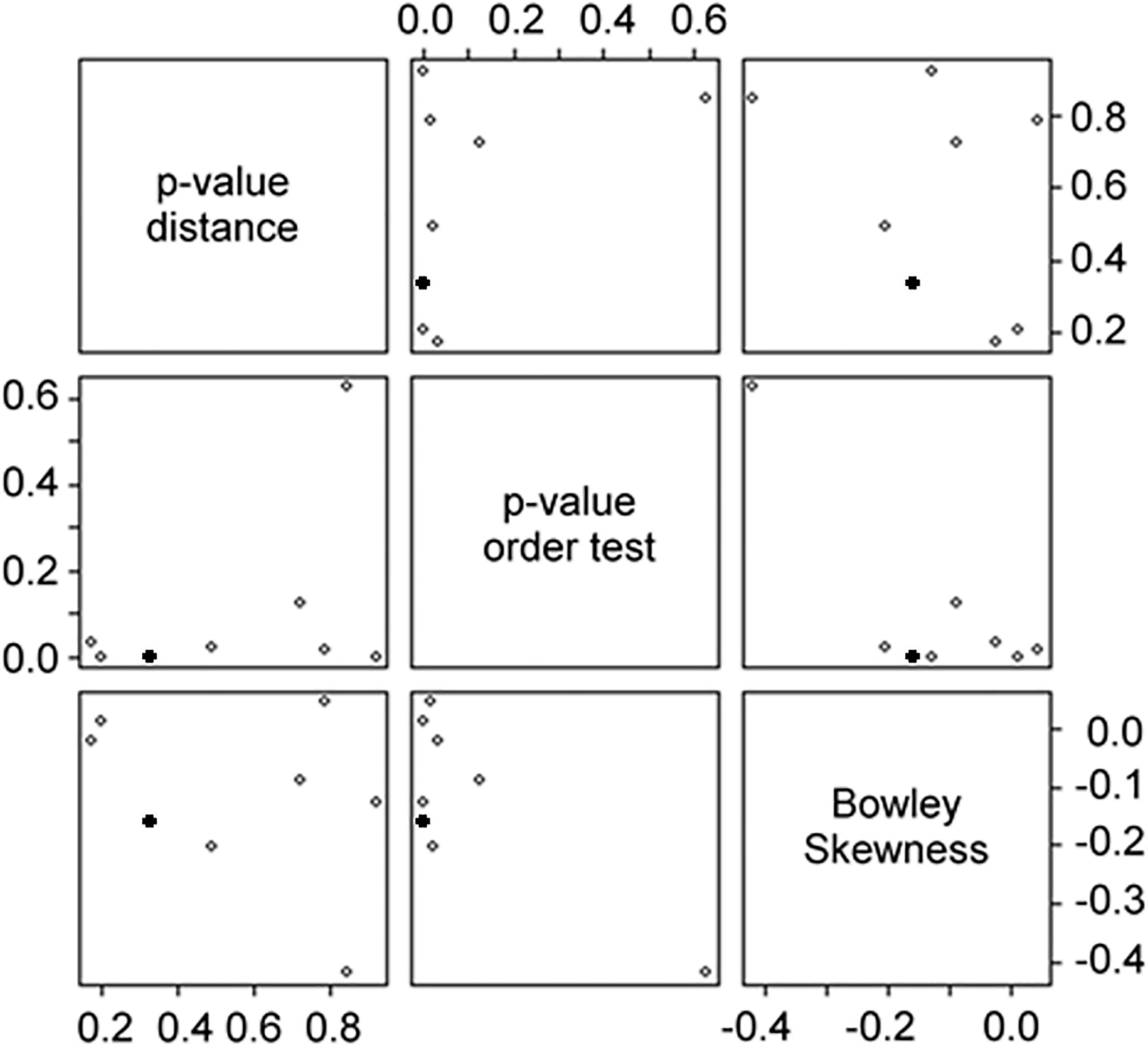
Scatterplot matrix summarizing the representative neural network analysis of three positional attributes. Each dot represents one CRE pair. Filled dots represent gene pairs that indicate the G box/ACANTACN pair, which is present in four photosystem genes and correlated with midday specific gene expression.

**Fig 10:**
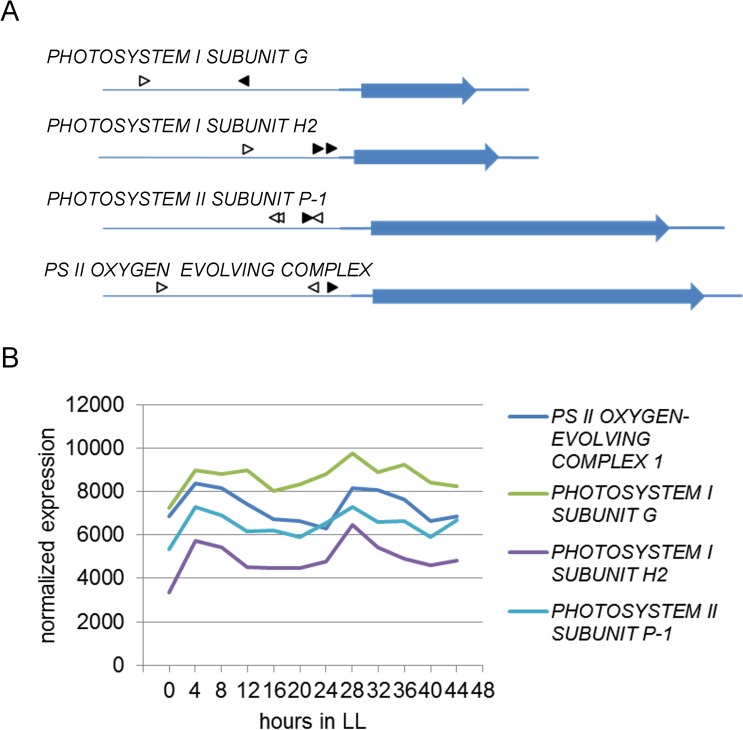
Positions of ACANTACN and G box motifs in photosystem subunit gene promoters and correlation with circadian gene expression. A) Positions of ACANTACN (white arrows) and the G box MCACGTGGC (black arrows) CREs in promoters of photosystem subunit genes. Blue arrows indicate CDS in 5’-3’ direction (introns are ignored), thicker blue lines indicate 5’ and 3’ UTRs. Thin blue line represents 1000 bp upstream region of the TSS. B) Circadian gene expression of the same photosystem genes as given by DIURNAL [81].

One exemplary CNG network includes eight CRE pairs, of which six showed significant overrepresentation of a specific order between the two CREs and the TSS (Fig 8A). None of the combinations showed a preference for a specific distance between the individual CREs (Fig 8B), and most combinations are positioned close to the TSS (Fig 8C). CNG summarizes the analysis of all three positional features in a scatterplot matrix, in which each point represents a specific CRE pair (Fig 9). One of the pairs that showed strong order preference and a tendency to be close to the TSS consists of a G box (MCACGTGGC) [64] and an undefined ACANTACN motif. Genes containing this CRE pair are enriched in the GO term photosynthesis. Four of the genes containing this combination belong to the photosystems I and II, respectively. These were the genes *PHOTOSYSTEM I SUBUNIT G*, *PHOTOSYSTEM I SUBUNIT H2*, *PHOTOSYSTEM II SUBUNIT P-1*, and *PS II OXYGEN-EVOLVING COMPLEX 1* (Fig 10A). They all exhibit their maximum expression between ZT4 and ZT8, i.e. in the middle of the subjective light phase (Fig 10B). In the promoters of these and 30 other genes, the G box motif is positioned closer to the TSS than the ACANTACN motif (p = 3.86∙10^−5^).

## Discussion

### EDCC correctly identifies known circadian clock promoter elements

Although a plethora of programs exist that allow deciphering of the influence of *cis*-regulatory elements on gene expression, most programs are either complicated to handle or cannot be used for large data sets, especially if statistical calculations are included. For example, the analysis of more than 1.5 million pairwise CRE combinations would suffer from a large multiple comparison error, or require large computing power. Here, we introduce the EDCC and CNG programs, which allow simple and fast identification of a large number of CREs and CRMs which may influence gene expression.

EDCC determines whether the presence of a CRE or CRM in promoters correlates with a specific expression pattern. For this, the expression data needs to be categorized into different treatment conditions prior to the EDCC analysis. EDCC compares the DEM of genes containing queried CREs/CRMs with the background distribution. With each analysis, EDCC runs a large set of random CREs and determines their standard deviation from the background. This standard deviation serves as the threshold at which a queried CRE is marked as a candidate.

In our study of CREs and CRM in circadian gene expression [14] we were able to identify only two of the known CREs as candidates, i.e. the evening element [23] and one of the known three midnight elements [14]. The morning element [25], two of the midnight elements [14] and the HUD-domain [27] were not found in the EDCC analysis. This means that i) EDCC is generally conservative and may generate false negatives, ii) the given positive controls have a small effect on circadian gene expression, and/or iii) the positive control CREs would have been discovered when using another circadian microarray experiment. As we mainly wanted to avoid discovery of false positive CREs, we were satisfied with the performance of EDCC on the positive control queries and continued to analyze all CREs given in the AtCOEcis database [5].

### EDCC finds both known and unknown CREs and CRE combinations that correlate with circadian gene expression

We used EDCC to analyze 1755 CREs with circadian microarray data and to identify candidate elements that correlate with gene expression at a specific time of the day. In one of the most conservative approaches we found 21 candidate CREs, which included the evening element [23], MYB1 and MYB2 binding site motifs [52,56,65], a wound responsive element [55], a TCP binding site [60], a GATA motif [59], ABA response element binding sites [41,66], and a G box element [64]. Whereas the Myb-domain transcription factors CCA1 and LATE ELONGATED HYPOCOTYL are involved in the regulation of the core clock, the homologs MYB1 and MYB2 have not been shown to be involved in circadian gene expression yet. Furthermore, MYB1 and MYB2 were both found to influence ABA signaling and responses [82,83]. ABA is a phytohormone that is essential in plant developmental processes as well as plant stress responses. Genes that are expressed rhythmically during a day-night cycle are overrepresented among ABA responsive genes [84] and ABA response to drought is gated by the circadian clock core component TIMING OF CAB EXPRESSION 1 [48,50,85]. Conversely, ABA treatment lengthens the circadian expression period of circadian clock genes [86]. Thus, it is fitting that the EDCC analysis identifies ABA response elements as candidate CREs in the regulation of circadian gene expression. Class I TCP transcription factors have been identified to control circadian gene expression, especially via binding to the promoter of the core clock gene *CCA1* [45–47,87]. In sum, these findings point out that EDCC indeed is able to identify candidate CREs that may confer specific gene expression.

In a next step, we used EDCC to analyze over 1.5 million pairs of CREs that were created by pairing each of the 1755 CREs with each other. We found a plethora of potential CRE pairs that correlate with daytime specific gene expression. The strongest effect was seen in the co-occurrence of the evening element with the DPBF1&2 binding site motif (ACACATG). Although first defined in carrots [67], a similar site (ACACNNG) has been found in Arabidopsis, where the motif is bound by the bZIP class transcription factor ABA-INSENSITIVE 5 (ABI5) [88], again pointing out the close association of ABA signaling with the circadian clock. No indications exist as yet to what the function of this pairwise combination is, and it would be one of the first CRE pairs to study in wetlab experiments after the EDCC analysis. Some positions within a CRE are less important for its function than others, leading to annotated CREs containing ambiguity code. When mutating the evening element/ DPBF1&2 binding site motif pair, we found that all positions of the evening element were important for EDCC to define the pair as a candidate. For the DPBF1&2 binding site motif, we found several variations which allowed us to indicate specific positions that are important for its presumed function. It would be interesting to determine whether these positions are indeed important for the evening element/ DPBF1&2 binding site motif pair to confer daytime specific gene expression, however this was beyond the scope of this study. This example also highlights another potential of EDCC: the EDCC program is able to analyze CREs with ambiguity code. For this, EDCC first analyzes the component CREs (e.g. AAAGA and AAAAA when calculating of AAARA) and then summarizes the results. EDCC would thus also be able to determine which of the component CREs correlates stronger with a specific expression pattern, allowing the identification of important positions. We have not tested this, but it would be an interesting future experiment.

When applying less restrictive conditions to the analysis of 1.5 million CRE pairs, EDCC identified more candidate CRE pairs. These often included at least one CRE that was previously found in circadian or light-responsive gene regulation, e.g. the evening element [23], a G box [89], a Z-DNA-forming sequence [90], or a dark responsive element [74]. In a previous study, Ding and colleagues used a frequent pattern mining approach to determine which CRE pairs are over-represented in Arabidopsis and poplar promoters [79]. When comparing our CRE pairs with those, we found that four CRE pairs were similar. Hence, these four combinations not only coincide often in plant promoters, they also correlate with specific peak circadian expression times of the respective genes. In summary, the EDCC program was able to not only detect CREs that are known to control circadian gene expression, further analysis also allowed to detect secondary CREs that are likely to influence circadian gene expression in combination with the previously known CREs. After validating these in wetlab approaches, it will be interesting to analyze, how they influence expression of target genes and what kinds of protein complexes bind to these.

### CNG scoring of positional CRE/CRM offers an unbiased approach to analyzing large-scale EDCC outputs

EDCC not only determines interesting secondary CREs, it also calculates positional features, as CRE positions are an important feature of CRE-mediated gene control [9,10,91]. The positional features calculated are: the distance of two CREs to each other, the distance of a CRM to the TSS, and the orientation of two CREs regarding which one is closer to the TSS. To prevent user-bias, we created the CNG program, which scores these positional features using a neural network. We used the CNG program to analyze CRE pairs that were found by EDCC. In a representative network scored by CNG we found the combination of a G box element with a ACANTACN sequence. This combination was found in 34 gene promoters and correlates with gene expression in the middle of the subjective day. One of the reasons that this combination was included by CNG is that the ACANTACN element is mostly positioned 5’ of the G box. We found this combination to be very prominent in the promoters of four photosystem subunit genes that are all expressed in the middle of the day. This indicates that this CRE combination indeed may affect day time specific gene expression. To our knowledge, this is the first description of this potential CRE pair and it would be interesting to validate these findings in wetlab experiments.

### Possibilities of EDCC and CNG and comparison with other approaches

The EDCC and CNG analysis have certain limitations, which will be discussed here. First of all, EDCC is designed to work with gene expression data, in which each gene exhibits maximum gene expression in one expression category. Circadian data was an ideal test case, as circadianly expressed genes exhibit a defined peak in contrast to other treatments or conditions. We see possible applications of this program in deciphering regulation of organ growth processes. For example, the identity of Arabidopsis floral organs is controlled by the presence of different MADS box transcription factors, each controlling different sets of genes (for a review, see [92]). These may be identified using the EDCC and CNG programs. In principle, any expression data that follows an OR logic, is suitable to be analyzed with the programs presented here. Furthermore, we have limited the analysis of CRMs to pairs of CREs. EDCC is in principle able to analyze combinations with more than two individual CREs, however the determination of positional features would not be possible yet. For example, the order of the two CREs in relation to the TSS is calculated using a binomial order test. A variation of EDCC with a multinomial test would be able to conduct the analysis. Also, the number of positional features that are calculated by EDCC can be increased. Such possible features are e.g. the number of repeats of a CRE, non-traditional positions like introns or downstream sequences, and the orientation of CREs, amongst others. EDCC is already able to include orientations of the CREs, but for this study we allowed CREs to appear in all possible orientations.

Whereas many programs were developed to identify CREs and CRMs in data sets, we designed a program that works with a user-identified list of CREs and CRMs. The simple approach of EDCC to correlate CREs/CRMs with gene expression data is reliable without being hindered by multiple comparison errors or by a lack in computing infrastructure. EDCC and CNG both run on PCs using free software (R and Phython), allowing non-experts fast identification of candidate CREs that may confer specific expression under different treatments and conditions.

Ultimately, the EDCC analysis provides a starting point for further in depth analysis of CRMs in gene expression. We showed that EDCC correctly identifies candidate CREs that are known for their effect on circadian gene expression. EDCC further identified candidate single CREs and CRE pairs that were not known to affect circadian gene expression. Some of the pairs are found in specific positions upstream of the respective genes. In the future, wetlab experiments need to show whether the presence and positions of these CREs are also functionally linked to circadian gene expression.

## Material and methods

### Exploration of Distinctive CREs and CRMs (EDCC)

EDCC compares the expression of genes containing a queried CRE with the background distribution of all genes that are affected by specific treatments or conditions. The CNG program scores the positional features that EDCC determines for candidate CRE pairs, avoiding user bias. Both EDCC and CNG are available for download under the link https://sourceforge.net/projects/edcc/. A manual is given in S1 File.

EDCC and CNG both provide graphical user interfaces (GUI). Additionally, EDCC provides an additional command line interface. The application is licensed under Apache License Version 2.0. EDCC is written in Python 3 and CGN in Python 3 and R, which makes them compatible with Microsoft Windows, macOS and Unix-like systems.

EDCC allows combining multiple CREs of interest in one query, by using the separator (,). Combinations of two CREs are further analyzed in respect to their positional attributes. The EDCC/CNG programs are able to include complementary and inversed sequences to the query CREs when specified by the user. All combined queries are split into single CREs before being validity checked, expanded and matched against the selected database (S3 Fig). Expansion means that query CREs that contain ambiguity code are broken down into their component CREs (e.g. AAAGCC and AAAACC in case of a AAARCC query). *K*-mer based indexing is used to maintain a high speed of the analysis. Peak expression times of promoters that match with the queries are extracted from an expression database (see below). If the initial query consisted of multiple CREs and was therefore split prior to the analysis, the results of all CRE are combined.

EDCC identifies whether a given query correlates with a DEM that differs from the background. The background contains all genes that are differentially expressed under the experimental conditions. The threshold is calculated using a user-determined number of random CREs (by default 100). EDCC calculates a DEM for each random CRE and determines a standard deviation for each expression category based on these DEMs. One standard deviation is the minimum threshold that is recommended in the EDCC analysis. As the random background is calculated in each run of EDCC, each run may produce slightly different results. To eliminate randomly occurring extreme variations, a default total of 100 backgrounds are produced per run and a query is termed ‘candidate’ when it deviates from the majority of the runs, respectively.

CREs that only occur in few promoters may exhibit distribution biases. Hence, the number of minimum matches a query has to meet is user-determined, but we do recommend using CREs that occur in at least 10 to 30 promoters. The default setting is 20 promoters.

### Analysis of positional features of CRE pairs

EDCC calculates three different positional features per candidate CRE pair:

#### Distance test

A two-sided Kolmogorov-Smirnov test is used to determine whether two CREs prefer a specific distance towards each other. The distribution of expected distances is generated using a stochastic approach: at first, the probability that a CRE occurs in a promoter is calculated. Then for each CRE as many random numbers are generated as expected to occur in 1000 bp, which represents the length of the analyzed promoter regions. The probabilities of the CREs are subtracted from each other and the smallest absolute difference between the probabilities is taken by EDCC to determine the distance of the CRM elements in a promoter. This procedure is performed 10,000 times to calculate the distribution of expected distances.

#### Order test

To determine whether CRE pairs occur predominantly in a given order in relation to the TSS, a binomial test is performed with the null hypothesis that each possible order of the two given CREs occurs with the same probability. The formula for this test is given below:

$$p(X)=\frac{n!}{(n-X)!X!}∙{\left(p\right)}^{X}∙{\left(q\right)}^{n-X})$$

Here, *p* and *q* are equal to 0.5, *n* is the total of pairwise occurrences and *X* is the number of occurrences of one possible order.

#### Bowley skewness of CRM positions

We defined the position of a CRM as the smallest distance of its constituent CREs to the TSS. As CRMs are predominantly positioned near the TSS, we expected that the distribution of the single positions of a CRM in the affected promoters is left-skewed. The skewness is calculated with Bowley‘s coefficient of skewness. The value range lies between -1 and 1. Positive values indicate a right-skewed distribution; negative values a left-skewed distribution. The skewness coefficient is calculated as follows:

$$S=\frac{Q_{3}+Q_{1}-2Q_{2}}{Q_{3}-Q_{1}}$$

where Q1 is the first, Q2 the second and Q3 the third quartile of the position‘s distribution.

### CRM Network Generator (CNG)

CNG uses an artificial two-class neural network to categorize and weigh positional features that were determined by EDCC (S2 Fig), thus precluding bias when manually assessing the EDCC output [33]. Via the CNG GUI, the user can change most parameters of the neural network generation. All neural networks created by CNG are feedforward networks that take the numeric results from the three statistical tests of EDCC as input. The networks consist of a neuron with a sigmoid activation function for the input and a Heaviside activation function for the output [93].

The CNG is trained with three types of neural network training data: the output of EDCC (i.e. the sequences of interest), sequences that exhibit p-values of 1 and Bowley skewness of -1, 0, and 1 (the negative control), and random sequences. The random sequences are used to ensure that the network categorization does not become too broad within the numeric data range of the positive sequences, as very broad categorizations could simply include all positive sequences without performing categorizations based on their properties.

The network training follows an evolutionary approach in order to get a more start values-independent categorization than with classical backpropagation [79,94]. In order to be more controllable in respect to the small number of inputs and outputs, the CNG training method only evolves the weights and biases of a neural network, but not its structure. Each evolutionary training process of networks is separated in “cycles“, which are separated again in “rounds”. The currently trained networks are scored each round (see below). Afterwards, the networks are sorted according to their score, and the best rated networks are selected for the next round. Mutated variants of the currently best rated networks and new randomly created networks are generated and scored together with the best networks of the previous round. The mutations can be either single incremental or disruptive changes of the biases or the weights, or crosses of two of the best rated networks. One CNG cycle ends when the score of the best network does not increase for a user-defined number of rounds. The best network of the last round of a cycle is saved internally and can be visualized later.

In the next cycle, the newly generated networks are forced to include positive sequences that have not been categorized before to ensure that the new networks are not identical to previous ones, and to increase the total number of categorized sequences. The CNG analysis ends when all positive sequences were categorized at least once in a generated network.

### Scoring of neural networks

The score of a network depends on the quantity of positive, negative and random sequences that are included in the network. If a network includes one of the negative sequences in its categorization, it gets the lowest score. If this is not the case, the network‘s score is calculated by dividing the number of positive sequences with the number of random sequences. If two or more networks have the same rating, the networks including more sequences are rated higher to avoid too narrow categories. The user can change most of the training process settings via the CNG user interface. This includes the fixed number of neurons of the hidden layer as well as all other numeric parameters to set or change the bias and the weights of the hidden layer‘s and the output layer‘s neurons during the training process. Each ongoing or finished training process, as well as each generated neural network, can be saved in a binary format. The binary files of training processes can be reloaded by the CNG.

### Visual output of the CNG

The CNG user interface shows the results of an ongoing or finished neural network training process. These are documented in HTML files which include textual information and plots. The subsequently generated index file is the starting point for the visualization. It shows all settings of the training process as well as an overview of all generated networks. Each generated network is also described and visualized in its own HTML file. The binary files of the training process and the single networks are automatically created with the HTML report. The index HTML file also shows the differences of the categorized sequences of the networks. This is done by generating a distance matrix of all generated networks. A value of 1 means that no sequences can be found in both categories, whereas a value of 0 means that all sequences of the smaller category are included in the larger category. This distance matrix is visualized as a 2D plot using the “symmetric SMACOF” multidimensional scaling method [95]. Additionally, the index file also shows whether a correlation between each of the input data of the categorized sequences of each particular network was detected using Spearman‘s correlation coefficient. The HTML files describing the single networks show a scatterplot of the input data, boxplots of the single data, the categorized sequences as well as a table containing the all included CREs and the genes in which they occur. The gene identifiers are provided in separate text files. These gene identifier lists allow subsequent analyses, such as GO analyses.

### Experimental data

The programs were tested using published data of a circadian microarray experiment (E-MEXP-1304) [14]. In this experiment Arabidopsis seedlings were grown for 9 days in a 12 hours light/12 hours dark regime and subsequently transferred to continuous light. Samples were taken every four hours for 48 hours after transfer to continuous light. We analyzed the continuous light experiment with the ARSER package and a significance cut-off of q = 0.05 [39]. Genes that exhibited circadian gene expression were categorized according to their peak expression time (ZT0-ZT4, ZT4-ZT8, ZT8-ZT12, ZT12-ZT16, ZT16-ZT20, ZT20-ZT0). Arabidopsis sequence data including 1000 bp upstream of the TSS for all coding and non-coding genes represented in TAIR10 was used to query for CREs, respectively [96]. We used 1755 CREs as given in the AtCOEcis database to test the programs [5]. These 1755 CREs include known motifs from PLACE [80] and AGRIS [4] and *de novo* motifs that were identified by homology between Arabidopsis and poplar [5]. Based on this collection we created a dataset in which each CRE was paired with a second CRE (disregarding the order), resulting in a query dataset of 1,540,890 CRE combinations.

## Supporting information

S1 Fig Schematic representation of EDCC analysis. Legend indicates input data, processes, and output of the EDCC analysis.
(TIF)

S2 FigSchematic representations of two-class neural networks generated by CNG.Neurons are shown as circles, numeric inputs as rectangles. All of these networks take the Bowley Skewness of a CRM's positions, the p value of the distance test of the CRM and the p value of the order test of the CRM as numeric input. The activation function of the *n* neurons in the sole hidden layer of these networks is the sigmoid function $(t)=\frac{1}{1+e^{-t}}$. For each of these neurons, the parameter for the activation function is the sum of the neuron‘s bias value with *t*. *t* is the sum of the weighted numeric inputs. Each hidden layer neuron has its own weight *w* for each numeric input. The output layer consists of one neuron. This neuron has the Heaviside function *h* as activation function. As parameter for *h*, the sum of the neuron‘s bias *b* and *t* is used. In this case, *t* is the sum of the weighted outputs of the hidden layer‘s neurons.(TIF)

S3 Fig Expansion of a CRE by EDCC. Handling of ambiguity code by EDCC. First, the ambiguity code is unscrambled into the component four bases. In the second step, complementary and inverse CREs are determined. Then, EDCC analysis is performed for each component CRE and the results united.
(TIF)

S1 Table Candidate single CREs identified by EDCC. 1755 CREs [5] were analyzed for correlation with a shift in circadian peak expression time. The table depicts all CREs that were found as candidates in five runs and occurred at least 10 times in Arabidopsis promoters.
(PDF)

S2 Table Candidate single CREs under conservative settings. EDCC analysis of 1755 CREs for correlation with a shift in circadian peak expression time in Arabidopsis. The number of minimum occurrences was increased to 15, 20, and 30, respectively. Given are all CREs that were found as candidates in five runs.
(PDF)

S3 Table Candidate CRE pairs that were used for CNG analysis. Given are 21 CRE pairs that have been found to correlate with a shift in peak expression time of circadianly expressed genes in Arabidopsis. All listed pairs occurred in at least 30 promoters and deviated from the background by at least five standard deviations in all five EDCC runs.
(PDF)

S1 File EDCC and CNG manual. The manual is also available as.html file under https://sourceforge.net/projects/edcc/files/edcc_cng.zip/download.
(DOCX)
