# Supplementary figures and images for: Computational exploration of cis-regulatory modules in rhythmic expression data using the “Exploration of Distinctive CREs and CRMs” (EDCC) and “CRM Network Generator” (CNG) programs
Source: PLoS One. 2018 Jan 3;13(1):e0190421. doi: 10.1371/journal.pone.0190421 (PMC5752016; doi:10.1371/journal.pone.0190421)

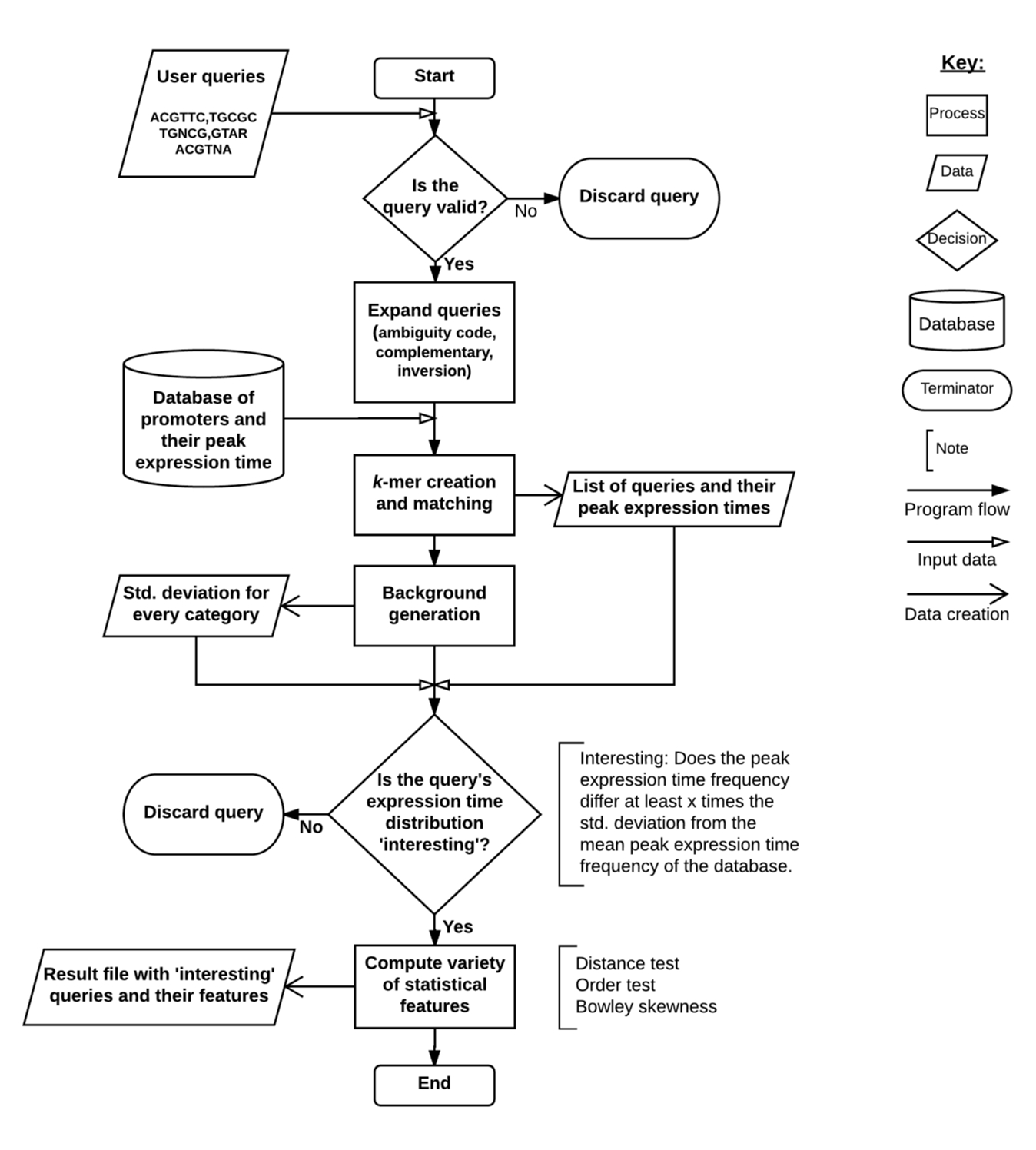

Supplement: S1 Fig — Legend indicates input data, processes, and output of the EDCC analysis. (TIF) [file pone.0190421.s001.tif]

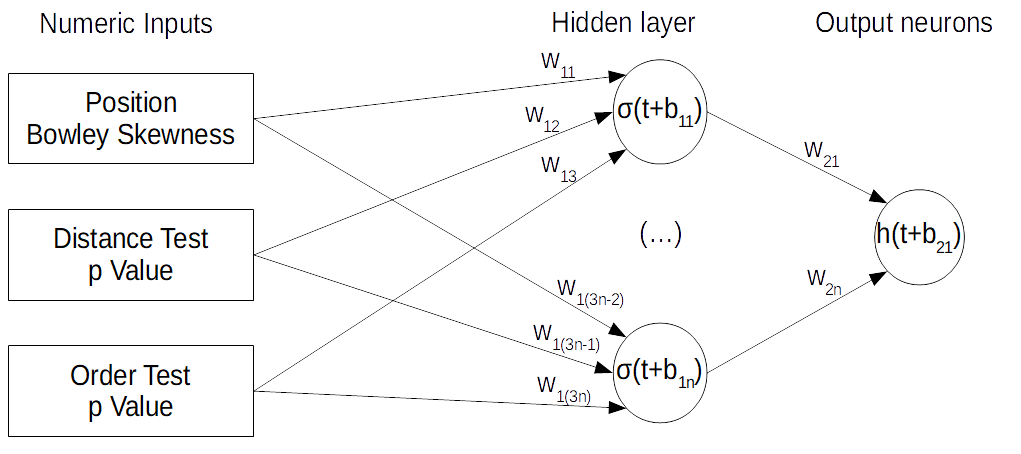

Supplement: S2 Fig — Neurons are shown as circles, numeric inputs as rectangles. All of these networks take the Bowley Skewness of a CRM's positions, the p value of the distance test of the CRM and the p value of the order test of the CRM as numeric input. The activation function of the n neurons in the sole hidden layer of these networks is the sigmoid function (t)=11+e−t. For each of these neurons, the parameter for the activation function is the sum of the neuron‘s bias value with t. t is the sum of the weighted numeric inputs. Each hidden layer neuron has its own weight w for each numeric input. The output layer consists of one neuron. This neuron has the Heaviside function h as activation function. As parameter for h, the sum of the neuron‘s bias b and t is used. In this case, t is the sum of the weighted outputs of the hidden layer‘s neurons. (TIF) [file pone.0190421.s002.tif]

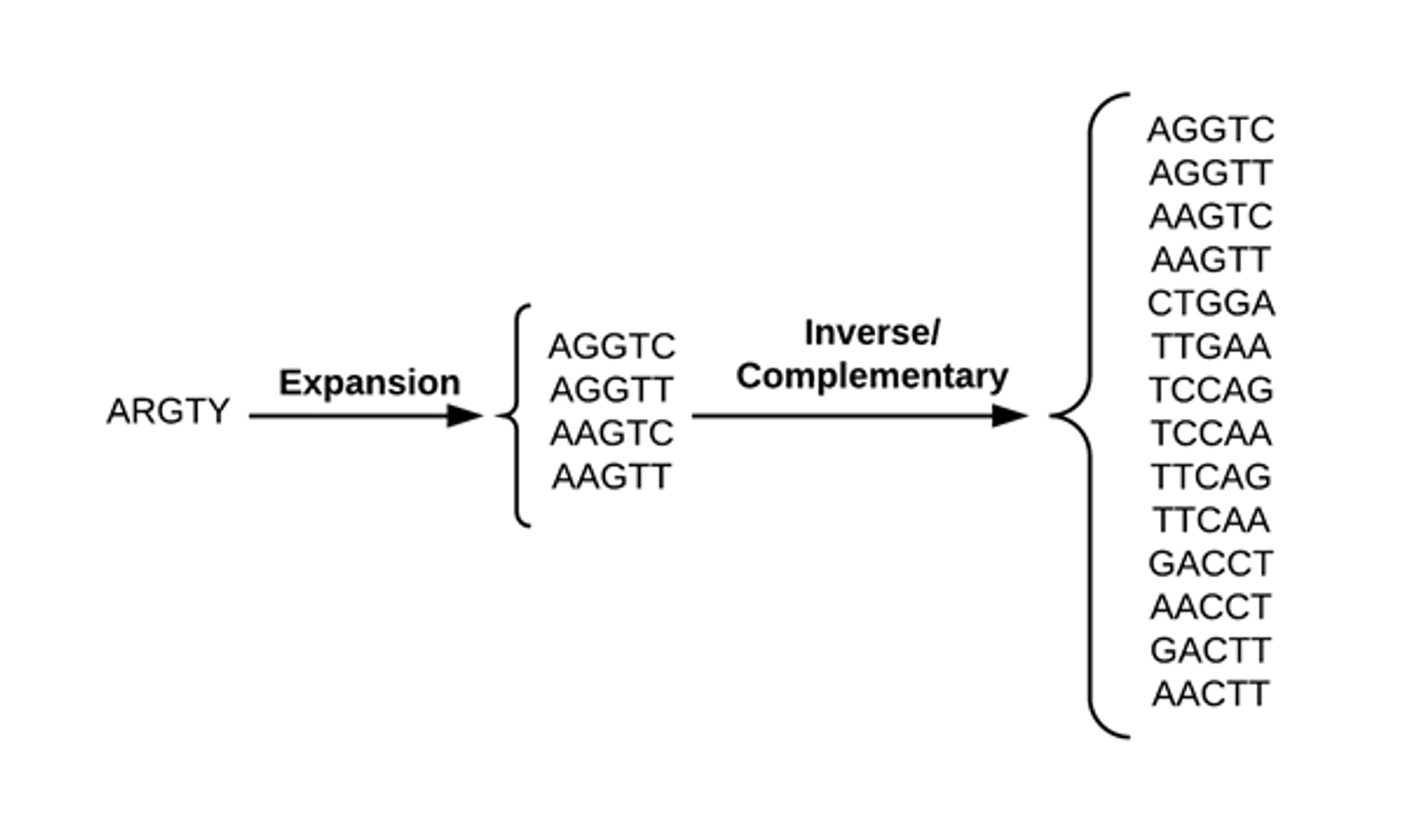

Supplement: S3 Fig — Handling of ambiguity code by EDCC. First, the ambiguity code is unscrambled into the component four bases. In the second step, complementary and inverse CREs are determined. Then, EDCC analysis is performed for each component CRE and the results united. (TIF) [file pone.0190421.s003.tif]
