# Supplementary material for: Computational exploration of cis-regulatory modules in rhythmic expression data using the “Exploration of Distinctive CREs and CRMs” (EDCC) and “CRM Network Generator” (CNG) programs
Source: PLoS One. 2018 Jan 3;13(1):e0190421. doi: 10.1371/journal.pone.0190421 (PMC5752016; doi:10.1371/journal.pone.0190421)
Supplement: S1 Table — 1755 CREs [5] were analyzed for correlation with a shift in circadian peak expression time. The table depicts all CREs that were found as candidates in five runs and occurred at least 10 times in Arabidopsis promoters. (PDF) [file pone.0190421.s004.pdf]

S1 Table: Candidate single CREs identified by EDCC

single sequence	minimum occurrences	run no.	interesting timepoints	sum of matches	genes
AAAATATCT	10	1	['8-12', '16-20', '20-24']	267	AT2G15090,AT5G51030,AT3G53800,AT1G34340,AT5G50780,AT3G53250,AT4G00400,AT1G06470,AT5G49910,AT3G52950,AT5G49120,AT5G49240,AT3G01120,AT3G05130,AT3G51620,AT1G75800,AT2G28840,AT1G28600,AT5G48250,AT1G66980,AT1G10760,AT1G01470,AT1G01500,AT1G14920,AT4G16146,AT5G45820,AT1G07190,AT1G07040,AT1G59870,AT1G20620,AT1G35350,AT3G47860,AT1G18070,AT1G54410,AT4G16860,AT1G28050,AT4G16950,AT2G23840,AT3G47470,AT3G47130,AT2G29670,AT1G51790,AT5G43150,AT2G18170,AT3G46510,AT3G46290,AT3G46440,AT4G14270,AT2G29630,AT1G29400,AT1G78460,AT1G78600,AT1G29670,AT2G29650,AT5G42310,AT2G47010,AT1G44100,AT1G35150,AT5G40500,AT3G42800,AT2G45990,AT4G39960,AT2G10370,AT1G42650,AT2G03890,AT1G76590,AT5G38510,AT4G39270,AT1G67970,AT2G17220,AT3G28340,AT1G68820,AT5G20630,AT5G08410,AT1G78210,AT2G22300,AT1G73760,AT2G41430,AT3G30380,AT4G37300,AT5G20380,AT2G21660,AT2G37520,AT1G66330,AT4G37480,AT4G37240,AT5G24150,AT3G13790,AT5G23870,AT1G52890,AT1G04400,AT1G26940,AT1G60000,AT1G74470,AT2G21380,AT3G13430,AT1G80280,AT5G22270,AT4G33985,AT2G40080,AT1G58150,AT1G69380,AT4G33980,AT4G33500,AT4G33470,AT1G69780,AT1G69880,AT2G36430,AT2G36060,AT2G33810,AT2G33830,AT2G02710,AT2G02760,AT3G28860,AT3G27300,AT5G15710,AT2G30600,AT2G22430,AT4G31550,AT5G14730,AT2G03680,AT4G31050,AT4G30660,AT2G22450,AT5G14320,AT4G30650,AT5G13950,AT3G15570,AT3G26570,AT4G29510,AT1G62430,AT1G08080,AT5G12150,AT1G32440,AT4G29840,AT4G29040,AT1G19450,AT5G11670,AT5G11930,AT4G28660,AT1G22770,AT5G26570,AT4G27800,AT5G11160,AT4G27780,AT3G26290,AT3G12800,AT1G12080,AT4G27710,AT5G08520,AT2G25190,AT3G27090,AT1G10210,AT1G06040,AT4G26530,AT3G26740,AT1G10200,AT5G66710,AT1G53540,AT4G26130,AT5G06690,AT1G26590,AT1G27440,AT5G66410,AT1G01320,AT1G55730,AT4G25660,AT4G24650,AT1G07420,AT5G05690,AT1G04710,AT1G62960,AT1G65640,AT3G20810,AT5G64860,AT1G19650,AT1G34440,AT4G24390,AT4G24470,AT5G64260,AT5G63860,AT1G12970,AT5G63780,AT5G63820,AT5G03220,AT2G17880,AT5G03350,AT1G08660,AT5G02820,AT3G21560,AT5G62160,AT1G07700,AT1G23210,AT1G14380,AT1G14400,AT1G14350,AT5G01950,AT5G61440,AT4G19500,AT3G16040,AT5G61590,AT3G63170,AT1G77000,AT3G63160,AT3G15450,AT3G62720,AT5G60540,AT1G24440,AT5G59950,AT3G16560,AT4G18000,AT3G02630,AT1G18330,AT1G18460,AT4G13250,AT1G23540,AT4G12500,AT3G61580,AT3G61470,AT5G58120,AT2G36720,AT3G08030,AT1G08230,AT5G57630,AT1G15950,AT3G60260,AT4G10920,AT5G57345,AT4G10360,AT2G22720,AT3G07840,AT1G80760,AT5G57040,AT3G59940,AT2G18220,AT3G05880,AT3G05800,AT3G59480,AT3G59350,AT2G18300,AT5G56240,AT1G33780,AT3G04550,AT2G46490,AT2G46530,AT4G05370,AT2G15970,AT1G74940,AT4G05300,AT3G57170,AT3G06410,AT2G20170,AT1G48330,AT2G25450,AT4G03520,AT4G03600,AT3G56090,AT1G73020,AT2G32100,AT3G08940,AT3G09390,AT4G02420,AT3G09250,AT3G55450,AT4G02260,AT5G51460,AT5G51390

AAAATATCT	10	2	['8-12', '16-20', '20-24']	267	AT2G15090,AT5G51030,AT3G53800,AT1G34340,AT5G50780,AT3G53250,AT4G00400,AT1G06470,AT5G49910,AT3G52950,AT5G49120,AT5G49240,AT3G01120,AT3G05130,AT3G51620,AT1G75800,AT2G28840,AT1G28600,AT5G48250,AT1G66980,AT1G10760,AT1G01470,AT1G01500,AT1G14920,AT4G16146,AT5G45820,AT1G07190,AT1G07040,AT1G59870,AT1G20620,AT1G35350,AT3G47860,AT1G18070,AT1G54410,AT4G16860,AT1G28050,AT4G16950,AT2G23840,AT3G47470,AT3G47130,AT2G29670,AT1G51790,AT5G43150,AT2G18170,AT3G46510,AT3G46290,AT3G46440,AT4G14270,AT2G29630,AT1G29400,AT1G78460,AT1G78600,AT1G29670,AT2G29650,AT5G42310,AT2G47010,AT1G44100,AT1G35150,AT5G40500,AT3G42800,AT2G45990,AT4G39960,AT2G10370,AT1G42650,AT2G03890,AT1G76590,AT5G38510,AT4G39270,AT1G67970,AT2G17220,AT3G28340,AT1G68820,AT5G20630,AT5G08410,AT1G78210,AT2G22300,AT1G73760,AT2G41430,AT3G30380,AT4G37300,AT5G20380,AT2G21660,AT2G37520,AT1G66330,AT4G37480,AT4G37240,AT5G24150,AT3G13790,AT5G23870,AT1G52890,AT1G04400,AT1G26940,AT1G60000,AT1G74470,AT2G21380,AT3G13430,AT1G80280,AT5G22270,AT4G33985,AT2G40080,AT1G58150,AT1G69380,AT4G33980,AT4G33500,AT4G33470,AT1G69780,AT1G69880,AT2G36430,AT2G36060,AT2G33810,AT2G33830,AT2G02710,AT2G02760,AT3G28860,AT3G27300,AT5G15710,AT2G30600,AT2G22430,AT4G31550,AT5G14730,AT2G03680,AT4G31050,AT4G30660,AT2G22450,AT5G14320,AT4G30650,AT5G13950,AT3G15570,AT3G26570,AT4G29510,AT1G62430,AT1G08080,AT5G12150,AT1G32440,AT4G29840,AT4G29040,AT1G19450,AT5G11670,AT5G11930,AT4G28660,AT1G22770,AT5G26570,AT4G27800,AT5G11160,AT4G27780,AT3G26290,AT3G12800,AT1G12080,AT4G27710,AT5G08520,AT2G25190,AT3G27090,AT1G10210,AT1G06040,AT4G26530,AT3G26740,AT1G10200,AT5G66710,AT1G53540,AT4G26130,AT5G06690,AT1G26590,AT1G27440,AT5G66410,AT1G01320,AT1G55730,AT4G25660,AT4G24650,AT1G07420,AT5G05690,AT1G04710,AT1G62960,AT1G65640,AT3G20810,AT5G64860,AT1G19650,AT1G34440,AT4G24390,AT4G24470,AT5G64260,AT5G63860,AT1G12970,AT5G63780,AT5G63820,AT5G03220,AT2G17880,AT5G03350,AT1G08660,AT5G02820,AT3G21560,AT5G62160,AT1G07700,AT1G23210,AT1G14380,AT1G14400,AT1G14350,AT5G01950,AT5G61440,AT4G19500,AT3G16040,AT5G61590,AT3G63170,AT1G77000,AT3G63160,AT3G15450,AT3G62720,AT5G60540,AT1G24440,AT5G59950,AT3G16560,AT4G18000,AT3G02630,AT1G18330,AT1G18460,AT4G13250,AT1G23540,AT4G12500,AT3G61580,AT3G61470,AT5G58120,AT2G36720,AT3G08030,AT1G08230,AT5G57630,AT1G15950,AT3G60260,AT4G10920,AT5G57345,AT4G10360,AT2G22720,AT3G07840,AT1G80760,AT5G57040,AT3G59940,AT2G18220,AT3G05880,AT3G05800,AT3G59480,AT3G59350,AT2G18300,AT5G56240,AT1G33780,AT3G04550,AT2G46490,AT2G46530,AT4G05370,AT2G15970,AT1G74940,AT4G05300,AT3G57170,AT3G06410,AT2G20170,AT1G48330,AT2G25450,AT4G03520,AT4G03600,AT3G56090,AT1G73020,AT2G32100,AT3G08940,AT3G09390,AT4G02420,AT3G09250,AT3G55450,AT4G02260,AT5G51460,AT5G51390

AAAATATCT	10	3	['8-12', '20-24', '16-20']	267	AT2G15090,AT5G51030,AT3G53800,AT1G34340,AT5G50780,AT3G53250,AT4G00400,AT1G06470,AT5G49910,AT3G52950,AT5G49120,AT5G49240,AT3G01120,AT3G05130,AT3G51620,AT1G75800,AT2G28840,AT1G28600,AT5G48250,AT1G66980,AT1G10760,AT1G01470,AT1G01500,AT1G14920,AT4G16146,AT5G45820,AT1G07190,AT1G07040,AT1G59870,AT1G20620,AT1G35350,AT3G47860,AT1G18070,AT1G54410,AT4G16860,AT1G28050,AT4G16950,AT2G23840,AT3G47470,AT3G47130,AT2G29670,AT1G51790,AT5G43150,AT2G18170,AT3G46510,AT3G46290,AT3G46440,AT4G14270,AT2G29630,AT1G29400,AT1G78460,AT1G78600,AT1G29670,AT2G29650,AT5G42310,AT2G47010,AT1G44100,AT1G35150,AT5G40500,AT3G42800,AT2G45990,AT4G39960,AT2G10370,AT1G42650,AT2G03890,AT1G76590,AT5G38510,AT4G39270,AT1G67970,AT2G17220,AT3G28340,AT1G68820,AT5G20630,AT5G08410,AT1G78210,AT2G22300,AT1G73760,AT2G41430,AT3G30380,AT4G37300,AT5G20380,AT2G21660,AT2G37520,AT1G66330,AT4G37480,AT4G37240,AT5G24150,AT3G13790,AT5G23870,AT1G52890,AT1G04400,AT1G26940,AT1G60000,AT1G74470,AT2G21380,AT3G13430,AT1G80280,AT5G22270,AT4G33985,AT2G40080,AT1G58150,AT1G69380,AT4G33980,AT4G33500,AT4G33470,AT1G69780,AT1G69880,AT2G36430,AT2G36060,AT2G33810,AT2G33830,AT2G02710,AT2G02760,AT3G28860,AT3G27300,AT5G15710,AT2G30600,AT2G22430,AT4G31550,AT5G14730,AT2G03680,AT4G31050,AT4G30660,AT2G22450,AT5G14320,AT4G30650,AT5G13950,AT3G15570,AT3G26570,AT4G29510,AT1G62430,AT1G08080,AT5G12150,AT1G32440,AT4G29840,AT4G29040,AT1G19450,AT5G11670,AT5G11930,AT4G28660,AT1G22770,AT5G26570,AT4G27800,AT5G11160,AT4G27780,AT3G26290,AT3G12800,AT1G12080,AT4G27710,AT5G08520,AT2G25190,AT3G27090,AT1G10210,AT1G06040,AT4G26530,AT3G26740,AT1G10200,AT5G66710,AT1G53540,AT4G26130,AT5G06690,AT1G26590,AT1G27440,AT5G66410,AT1G01320,AT1G55730,AT4G25660,AT4G24650,AT1G07420,AT5G05690,AT1G04710,AT1G62960,AT1G65640,AT3G20810,AT5G64860,AT1G19650,AT1G34440,AT4G24390,AT4G24470,AT5G64260,AT5G63860,AT1G12970,AT5G63780,AT5G63820,AT5G03220,AT2G17880,AT5G03350,AT1G08660,AT5G02820,AT3G21560,AT5G62160,AT1G07700,AT1G23210,AT1G14380,AT1G14400,AT1G14350,AT5G01950,AT5G61440,AT4G19500,AT3G16040,AT5G61590,AT3G63170,AT1G77000,AT3G63160,AT3G15450,AT3G62720,AT5G60540,AT1G24440,AT5G59950,AT3G16560,AT4G18000,AT3G02630,AT1G18330,AT1G18460,AT4G13250,AT1G23540,AT4G12500,AT3G61580,AT3G61470,AT5G58120,AT2G36720,AT3G08030,AT1G08230,AT5G57630,AT1G15950,AT3G60260,AT4G10920,AT5G57345,AT4G10360,AT2G22720,AT3G07840,AT1G80760,AT5G57040,AT3G59940,AT2G18220,AT3G05880,AT3G05800,AT3G59480,AT3G59350,AT2G18300,AT5G56240,AT1G33780,AT3G04550,AT2G46490,AT2G46530,AT4G05370,AT2G15970,AT1G74940,AT4G05300,AT3G57170,AT3G06410,AT2G20170,AT1G48330,AT2G25450,AT4G03520,AT4G03600,AT3G56090,AT1G73020,AT2G32100,AT3G08940,AT3G09390,AT4G02420,AT3G09250,AT3G55450,AT4G02260,AT5G51460,AT5G51390

AAAATATCT	10	4	['8-12', '16-20', '20-24']	267	AT2G15090,AT5G51030,AT3G53800,AT1G34340,AT5G50780,AT3G53250,AT4G00400,AT1G06470,AT5G49910,AT3G52950,AT5G49120,AT5G49240,AT3G01120,AT3G05130,AT3G51620,AT1G75800,AT2G28840,AT1G28600,AT5G48250,AT1G66980,AT1G10760,AT1G01470,AT1G01500,AT1G14920,AT4G16146,AT5G45820,AT1G07190,AT1G07040,AT1G59870,AT1G20620,AT1G35350,AT3G47860,AT1G18070,AT1G54410,AT4G16860,AT1G28050,AT4G16950,AT2G23840,AT3G47470,AT3G47130,AT2G29670,AT1G51790,AT5G43150,AT2G18170,AT3G46510,AT3G46290,AT3G46440,AT4G14270,AT2G29630,AT1G29400,AT1G78460,AT1G78600,AT1G29670,AT2G29650,AT5G42310,AT2G47010,AT1G44100,AT1G35150,AT5G40500,AT3G42800,AT2G45990,AT4G39960,AT2G10370,AT1G42650,AT2G03890,AT1G76590,AT5G38510,AT4G39270,AT1G67970,AT2G17220,AT3G28340,AT1G68820,AT5G20630,AT5G08410,AT1G78210,AT2G22300,AT1G73760,AT2G41430,AT3G30380,AT4G37300,AT5G20380,AT2G21660,AT2G37520,AT1G66330,AT4G37480,AT4G37240,AT5G24150,AT3G13790,AT5G23870,AT1G52890,AT1G04400,AT1G26940,AT1G60000,AT1G74470,AT2G21380,AT3G13430,AT1G80280,AT5G22270,AT4G33985,AT2G40080,AT1G58150,AT1G69380,AT4G33980,AT4G33500,AT4G33470,AT1G69780,AT1G69880,AT2G36430,AT2G36060,AT2G33810,AT2G33830,AT2G02710,AT2G02760,AT3G28860,AT3G27300,AT5G15710,AT2G30600,AT2G22430,AT4G31550,AT5G14730,AT2G03680,AT4G31050,AT4G30660,AT2G22450,AT5G14320,AT4G30650,AT5G13950,AT3G15570,AT3G26570,AT4G29510,AT1G62430,AT1G08080,AT5G12150,AT1G32440,AT4G29840,AT4G29040,AT1G19450,AT5G11670,AT5G11930,AT4G28660,AT1G22770,AT5G26570,AT4G27800,AT5G11160,AT4G27780,AT3G26290,AT3G12800,AT1G12080,AT4G27710,AT5G08520,AT2G25190,AT3G27090,AT1G10210,AT1G06040,AT4G26530,AT3G26740,AT1G10200,AT5G66710,AT1G53540,AT4G26130,AT5G06690,AT1G26590,AT1G27440,AT5G66410,AT1G01320,AT1G55730,AT4G25660,AT4G24650,AT1G07420,AT5G05690,AT1G04710,AT1G62960,AT1G65640,AT3G20810,AT5G64860,AT1G19650,AT1G34440,AT4G24390,AT4G24470,AT5G64260,AT5G63860,AT1G12970,AT5G63780,AT5G63820,AT5G03220,AT2G17880,AT5G03350,AT1G08660,AT5G02820,AT3G21560,AT5G62160,AT1G07700,AT1G23210,AT1G14380,AT1G14400,AT1G14350,AT5G01950,AT5G61440,AT4G19500,AT3G16040,AT5G61590,AT3G63170,AT1G77000,AT3G63160,AT3G15450,AT3G62720,AT5G60540,AT1G24440,AT5G59950,AT3G16560,AT4G18000,AT3G02630,AT1G18330,AT1G18460,AT4G13250,AT1G23540,AT4G12500,AT3G61580,AT3G61470,AT5G58120,AT2G36720,AT3G08030,AT1G08230,AT5G57630,AT1G15950,AT3G60260,AT4G10920,AT5G57345,AT4G10360,AT2G22720,AT3G07840,AT1G80760,AT5G57040,AT3G59940,AT2G18220,AT3G05880,AT3G05800,AT3G59480,AT3G59350,AT2G18300,AT5G56240,AT1G33780,AT3G04550,AT2G46490,AT2G46530,AT4G05370,AT2G15970,AT1G74940,AT4G05300,AT3G57170,AT3G06410,AT2G20170,AT1G48330,AT2G25450,AT4G03520,AT4G03600,AT3G56090,AT1G73020,AT2G32100,AT3G08940,AT3G09390,AT4G02420,AT3G09250,AT3G55450,AT4G02260,AT5G51460,AT5G51390

AAAATATCT	10	5	['8-12', '16-20', '20-24']	267	AT2G15090,AT5G51030,AT3G53800,AT1G34340,AT5G50780,AT3G53250,AT4G00400,AT1G06470,AT5G49910,AT3G52950,AT5G49120,AT5G49240,AT3G01120,AT3G05130,AT3G51620,AT1G75800,AT2G28840,AT1G28600,AT5G48250,AT1G66980,AT1G10760,AT1G01470,AT1G01500,AT1G14920,AT4G16146,AT5G45820,AT1G07190,AT1G07040,AT1G59870,AT1G20620,AT1G35350,AT3G47860,AT1G18070,AT1G54410,AT4G16860,AT1G28050,AT4G16950,AT2G23840,AT3G47470,AT3G47130,AT2G29670,AT1G51790,AT5G43150,AT2G18170,AT3G46510,AT3G46290,AT3G46440,AT4G14270,AT2G29630,AT1G29400,AT1G78460,AT1G78600,AT1G29670,AT2G29650,AT5G42310,AT2G47010,AT1G44100,AT1G35150,AT5G40500,AT3G42800,AT2G45990,AT4G39960,AT2G10370,AT1G42650,AT2G03890,AT1G76590,AT5G38510,AT4G39270,AT1G67970,AT2G17220,AT3G28340,AT1G68820,AT5G20630,AT5G08410,AT1G78210,AT2G22300,AT1G73760,AT2G41430,AT3G30380,AT4G37300,AT5G20380,AT2G21660,AT2G37520,AT1G66330,AT4G37480,AT4G37240,AT5G24150,AT3G13790,AT5G23870,AT1G52890,AT1G04400,AT1G26940,AT1G60000,AT1G74470,AT2G21380,AT3G13430,AT1G80280,AT5G22270,AT4G33985,AT2G40080,AT1G58150,AT1G69380,AT4G33980,AT4G33500,AT4G33470,AT1G69780,AT1G69880,AT2G36430,AT2G36060,AT2G33810,AT2G33830,AT2G02710,AT2G02760,AT3G28860,AT3G27300,AT5G15710,AT2G30600,AT2G22430,AT4G31550,AT5G14730,AT2G03680,AT4G31050,AT4G30660,AT2G22450,AT5G14320,AT4G30650,AT5G13950,AT3G15570,AT3G26570,AT4G29510,AT1G62430,AT1G08080,AT5G12150,AT1G32440,AT4G29840,AT4G29040,AT1G19450,AT5G11670,AT5G11930,AT4G28660,AT1G22770,AT5G26570,AT4G27800,AT5G11160,AT4G27780,AT3G26290,AT3G12800,AT1G12080,AT4G27710,AT5G08520,AT2G25190,AT3G27090,AT1G10210,AT1G06040,AT4G26530,AT3G26740,AT1G10200,AT5G66710,AT1G53540,AT4G26130,AT5G06690,AT1G26590,AT1G27440,AT5G66410,AT1G01320,AT1G55730,AT4G25660,AT4G24650,AT1G07420,AT5G05690,AT1G04710,AT1G62960,AT1G65640,AT3G20810,AT5G64860,AT1G19650,AT1G34440,AT4G24390,AT4G24470,AT5G64260,AT5G63860,AT1G12970,AT5G63780,AT5G63820,AT5G03220,AT2G17880,AT5G03350,AT1G08660,AT5G02820,AT3G21560,AT5G62160,AT1G07700,AT1G23210,AT1G14380,AT1G14400,AT1G14350,AT5G01950,AT5G61440,AT4G19500,AT3G16040,AT5G61590,AT3G63170,AT1G77000,AT3G63160,AT3G15450,AT3G62720,AT5G60540,AT1G24440,AT5G59950,AT3G16560,AT4G18000,AT3G02630,AT1G18330,AT1G18460,AT4G13250,AT1G23540,AT4G12500,AT3G61580,AT3G61470,AT5G58120,AT2G36720,AT3G08030,AT1G08230,AT5G57630,AT1G15950,AT3G60260,AT4G10920,AT5G57345,AT4G10360,AT2G22720,AT3G07840,AT1G80760,AT5G57040,AT3G59940,AT2G18220,AT3G05880,AT3G05800,AT3G59480,AT3G59350,AT2G18300,AT5G56240,AT1G33780,AT3G04550,AT2G46490,AT2G46530,AT4G05370,AT2G15970,AT1G74940,AT4G05300,AT3G57170,AT3G06410,AT2G20170,AT1G48330,AT2G25450,AT4G03520,AT4G03600,AT3G56090,AT1G73020,AT2G32100,AT3G08940,AT3G09390,AT4G02420,AT3G09250,AT3G55450,AT4G02260,AT5G51460,AT5G51390
AAACAATCTA	10	1	['0-4', '16-20']	35	AT2G05920,AT3G02660,AT5G26820,AT1G06680,AT4G38850,AT3G61830,AT2G31820,AT1G12080,AT1G23540,AT2G42600,AT1G73180,AT5G67020,AT1G27480,AT4G10360,AT2G20260,AT3G23940,AT5G56230,AT5G04490,AT4G05370,AT1G69390,AT3G31980,AT2G22500,AT2G22360,AT1G29490,AT5G14730,AT1G79590,AT5G04140,AT1G53320,AT3G11050,AT3G15690,AT5G09810,AT3G54890,AT5G51570,AT5G51770,AT2G36100
AAACAATCTA	10	2	['0-4', '16-20']	35	AT2G05920,AT3G02660,AT5G26820,AT1G06680,AT4G38850,AT3G61830,AT2G31820,AT1G12080,AT1G23540,AT2G42600,AT1G73180,AT5G67020,AT1G27480,AT4G10360,AT2G20260,AT3G23940,AT5G56230,AT5G04490,AT4G05370,AT1G69390,AT3G31980,AT2G22500,AT2G22360,AT1G29490,AT5G14730,AT1G79590,AT5G04140,AT1G53320,AT3G11050,AT3G15690,AT5G09810,AT3G54890,AT5G51570,AT5G51770,AT2G36100
AAACAATCTA	10	3	['0-4', '16-20']	35	AT2G05920,AT3G02660,AT5G26820,AT1G06680,AT4G38850,AT3G61830,AT2G31820,AT1G12080,AT1G23540,AT2G42600,AT1G73180,AT5G67020,AT1G27480,AT4G10360,AT2G20260,AT3G23940,AT5G56230,AT5G04490,AT4G05370,AT1G69390,AT3G31980,AT2G22500,AT2G22360,AT1G29490,AT5G14730,AT1G79590,AT5G04140,AT1G53320,AT3G11050,AT3G15690,AT5G09810,AT3G54890,AT5G51570,AT5G51770,AT2G36100
AAACAATCTA	10	4	['0-4', '16-20']	35	AT2G05920,AT3G02660,AT5G26820,AT1G06680,AT4G38850,AT3G61830,AT2G31820,AT1G12080,AT1G23540,AT2G42600,AT1G73180,AT5G67020,AT1G27480,AT4G10360,AT2G20260,AT3G23940,AT5G56230,AT5G04490,AT4G05370,AT1G69390,AT3G31980,AT2G22500,AT2G22360,AT1G29490,AT5G14730,AT1G79590,AT5G04140,AT1G53320,AT3G11050,AT3G15690,AT5G09810,AT3G54890,AT5G51570,AT5G51770,AT2G36100

AAACAATCTA	10	5	['0-4', '16-20']	35	AT2G05920,AT3G02660,AT5G26820,AT1G06680,AT4G38850,AT3G61830,AT2G31820,AT1G12080,AT1G23540,AT2G42600,AT1G73180,AT5G67020,AT1G27480,AT4G10360,AT2G20260,AT3G23940,AT5G56230,AT5G04490,AT4G05370,AT1G69390,AT3G31980,AT2G22500,AT2G22360,AT1G29490,AT5G14730,AT1G79590,AT5G04140,AT1G53320,AT3G11050,AT3G15690,AT5G09810,AT3G54890,AT5G51570,AT5G51770,AT2G36100
AAATTAACCAA	10	1	['0-4', '4-8', '12-16', '16-20', '20-24']	11	AT5G38980,AT5G24530,AT3G51520,AT3G56000,AT2G18640,AT5G62130,AT1G78240,AT4G19350,AT5G29000,AT5G54310,AT1G60970
AAATTAACCAA	10	2	['0-4', '4-8', '16-20', '20-24', '12-16']	11	AT5G38980,AT5G24530,AT3G51520,AT3G56000,AT2G18640,AT5G62130,AT1G78240,AT4G19350,AT5G29000,AT5G54310,AT1G60970
AAATTAACCAA	10	3	['0-4', '4-8', '16-20', '20-24', '12-16']	11	AT5G38980,AT5G24530,AT3G51520,AT3G56000,AT2G18640,AT5G62130,AT1G78240,AT4G19350,AT5G29000,AT5G54310,AT1G60970
AAATTAACCAA	10	4	['0-4', '4-8', '16-20', '20-24']	11	AT5G38980,AT5G24530,AT3G51520,AT3G56000,AT2G18640,AT5G62130,AT1G78240,AT4G19350,AT5G29000,AT5G54310,AT1G60970
AAATTAACCAA	10	5	['0-4', '4-8', '12-16', '16-20', '20-24']	11	AT5G38980,AT5G24530,AT3G51520,AT3G56000,AT2G18640,AT5G62130,AT1G78240,AT4G19350,AT5G29000,AT5G54310,AT1G60970
AACAATCT	10	1	['20-24']	210	AT3G53720,AT5G51040,AT2G05920,AT4G00620,AT2G40935,AT5G49740,AT3G07700,AT1G06680,AT5G49480,AT3G01120,AT2G33420,AT1G67660,AT1G07950,AT2G28740,AT1G67830,AT1G13280,AT5G48370,AT1G66970,AT1G75800,AT2G29250,AT1G06310,AT1G22050,AT1G13880,AT5G46910,AT3G49590,AT1G11680,AT5G46630,AT1G19060,AT1G14810,AT3G48580,AT1G15820,AT4G17090,AT5G45170,AT2G07050,AT1G20693,AT1G59930,AT3G47650,AT4G16520,AT3G47550,AT5G44390,AT3G47560,AT3G46970,AT3G46620,AT5G43200,AT2G16070,AT3G12600,AT5G42810,AT1G29490,AT1G72030,AT3G45210,AT5G42310,AT3G06180,AT5G04140,AT3G11050,AT3G11170,AT2G25930,AT3G43670,AT1G76730,AT2G36100,AT2G45980,AT1G31410,AT5G28280,AT5G09270,AT1G76450,AT1G76460,AT3G31915,AT3G30840,AT4G38850,AT2G41080,AT2G41040,AT1G73670,AT2G31820,AT5G21170,AT3G52340,AT2G42600,AT1G78180,AT1G73180,AT2G31040,AT1G36390,AT5G24610,AT5G20840,AT5G24380,AT3G22200,AT2G04795,AT2G25310,AT1G31814,AT3G23940,AT5G23060,AT2G30150,AT4G34370,AT5G22310,AT3G51820,AT3G21110,AT3G56700,AT2G36880,AT3G28715,AT2G26170,AT1G58150,AT1G69390,AT5G16910,AT5G16730,AT2G30600,AT3G24260,AT3G24430,AT2G22500,AT2G32765,AT2G22360,AT5G14730,AT2G43550,AT5G14760,AT2G45300,AT2G32880,AT1G53320,AT5G09810,AT5G28140,AT1G53230,AT1G79270,AT1G62340,AT5G12950,AT4G28750,AT1G22690,AT5G11450,AT3G24760,AT5G26340,AT4G27790,AT5G26820,AT1G06200,AT1G78670,AT1G78320,AT1G06750,AT1G12080,AT5G67020,AT1G27480,AT4G26130,AT5G66710,AT5G05950,AT1G07280,AT5G65530,AT1G17460,AT3G21750,AT1G65640,AT1G08890,AT5G64630,AT5G04490,AT1G12860,AT5G03560,AT1G05910,AT5G03220,AT1G08640,AT1G79610,AT1G79590,AT5G02760,AT3G15840,AT3G27700,AT4G19110,AT5G62360,AT3G15690,AT1G14370,AT1G60600,AT1G24440,AT5G59850,AT3G02660,AT3G62100,AT5G59300,AT3G61830,AT1G23540,AT3G08030,AT1G25155,AT2G01940,AT1G16000,AT4G11320,AT1G11545,AT4G10360,AT2G20260,AT3G09740,AT2G18410,AT4G08930,AT5G56230,AT1G64640,AT2G13260,AT1G35580,AT1G80030,AT3G58610,AT4G07970,AT5G56090,AT4G07425,AT4G05370,AT3G58620,AT3G10030,AT3G31980,AT4G04955,AT1G48350,AT5G54680,AT5G53880,AT5G54080,AT3G10690,AT4G03110,AT5G53770,AT5G53350,AT4G02940,AT3G08920,AT3G07350,AT2G35620,AT3G54890,AT5G52120,AT5G52010,AT5G51570,AT5G51770,AT4G01130,AT1G17090

AACAATCT	10	2	['20-24']	210	AT3G53720,AT5G51040,AT2G05920,AT4G00620,AT2G40935,AT5G49740,AT3G07700,AT1G06680,AT5G49480,AT3G01120,AT2G33420,AT1G67660,AT1G07950,AT2G28740,AT1G67830,AT1G13280,AT5G48370,AT1G66970,AT1G75800,AT2G29250,AT1G06310,AT1G22050,AT1G13880,AT5G46910,AT3G49590,AT1G11680,AT5G46630,AT1G19060,AT1G14810,AT3G48580,AT1G15820,AT4G17090,AT5G45170,AT2G07050,AT1G20693,AT1G59930,AT3G47650,AT4G16520,AT3G47550,AT5G44390,AT3G47560,AT3G46970,AT3G46620,AT5G43200,AT2G16070,AT3G12600,AT5G42810,AT1G29490,AT1G72030,AT3G45210,AT5G42310,AT3G06180,AT5G04140,AT3G11050,AT3G11170,AT2G25930,AT3G43670,AT1G76730,AT2G36100,AT2G45980,AT1G31410,AT5G28280,AT5G09270,AT1G76450,AT1G76460,AT3G31915,AT3G30840,AT4G38850,AT2G41080,AT2G41040,AT1G73670,AT2G31820,AT5G21170,AT3G52340,AT2G42600,AT1G78180,AT1G73180,AT2G31040,AT1G36390,AT5G24610,AT5G20840,AT5G24380,AT3G22200,AT2G04795,AT2G25310,AT1G31814,AT3G23940,AT5G23060,AT2G30150,AT4G34370,AT5G22310,AT3G51820,AT3G21110,AT3G56700,AT2G36880,AT3G28715,AT2G26170,AT1G58150,AT1G69390,AT5G16910,AT5G16730,AT2G30600,AT3G24260,AT3G24430,AT2G22500,AT2G32765,AT2G22360,AT5G14730,AT2G43550,AT5G14760,AT2G45300,AT2G32880,AT1G53320,AT5G09810,AT5G28140,AT1G53230,AT1G79270,AT1G62340,AT5G12950,AT4G28750,AT1G22690,AT5G11450,AT3G24760,AT5G26340,AT4G27790,AT5G26820,AT1G06200,AT1G78670,AT1G78320,AT1G06750,AT1G12080,AT5G67020,AT1G27480,AT4G26130,AT5G66710,AT5G05950,AT1G07280,AT5G65530,AT1G17460,AT3G21750,AT1G65640,AT1G08890,AT5G64630,AT5G04490,AT1G12860,AT5G03560,AT1G05910,AT5G03220,AT1G08640,AT1G79610,AT1G79590,AT5G02760,AT3G15840,AT3G27700,AT4G19110,AT5G62360,AT3G15690,AT1G14370,AT1G60600,AT1G24440,AT5G59850,AT3G02660,AT3G62100,AT5G59300,AT3G61830,AT1G23540,AT3G08030,AT1G25155,AT2G01940,AT1G16000,AT4G11320,AT1G11545,AT4G10360,AT2G20260,AT3G09740,AT2G18410,AT4G08930,AT5G56230,AT1G64640,AT2G13260,AT1G35580,AT1G80030,AT3G58610,AT4G07970,AT5G56090,AT4G07425,AT4G05370,AT3G58620,AT3G10030,AT3G31980,AT4G04955,AT1G48350,AT5G54680,AT5G53880,AT5G54080,AT3G10690,AT4G03110,AT5G53770,AT5G53350,AT4G02940,AT3G08920,AT3G07350,AT2G35620,AT3G54890,AT5G52120,AT5G52010,AT5G51570,AT5G51770,AT4G01130,AT1G17090
AACAATCT	10	3	['20-24']	210	AT3G53720,AT5G51040,AT2G05920,AT4G00620,AT2G40935,AT5G49740,AT3G07700,AT1G06680,AT5G49480,AT3G01120,AT2G33420,AT1G67660,AT1G07950,AT2G28740,AT1G67830,AT1G13280,AT5G48370,AT1G66970,AT1G75800,AT2G29250,AT1G06310,AT1G22050,AT1G13880,AT5G46910,AT3G49590,AT1G11680,AT5G46630,AT1G19060,AT1G14810,AT3G48580,AT1G15820,AT4G17090,AT5G45170,AT2G07050,AT1G20693,AT1G59930,AT3G47650,AT4G16520,AT3G47550,AT5G44390,AT3G47560,AT3G46970,AT3G46620,AT5G43200,AT2G16070,AT3G12600,AT5G42810,AT1G29490,AT1G72030,AT3G45210,AT5G42310,AT3G06180,AT5G04140,AT3G11050,AT3G11170,AT2G25930,AT3G43670,AT1G76730,AT2G36100,AT2G45980,AT1G31410,AT5G28280,AT5G09270,AT1G76450,AT1G76460,AT3G31915,AT3G30840,AT4G38850,AT2G41080,AT2G41040,AT1G73670,AT2G31820,AT5G21170,AT3G52340,AT2G42600,AT1G78180,AT1G73180,AT2G31040,AT1G36390,AT5G24610,AT5G20840,AT5G24380,AT3G22200,AT2G04795,AT2G25310,AT1G31814,AT3G23940,AT5G23060,AT2G30150,AT4G34370,AT5G22310,AT3G51820,AT3G21110,AT3G56700,AT2G36880,AT3G28715,AT2G26170,AT1G58150,AT1G69390,AT5G16910,AT5G16730,AT2G30600,AT3G24260,AT3G24430,AT2G22500,AT2G32765,AT2G22360,AT5G14730,AT2G43550,AT5G14760,AT2G45300,AT2G32880,AT1G53320,AT5G09810,AT5G28140,AT1G53230,AT1G79270,AT1G62340,AT5G12950,AT4G28750,AT1G22690,AT5G11450,AT3G24760,AT5G26340,AT4G27790,AT5G26820,AT1G06200,AT1G78670,AT1G78320,AT1G06750,AT1G12080,AT5G67020,AT1G27480,AT4G26130,AT5G66710,AT5G05950,AT1G07280,AT5G65530,AT1G17460,AT3G21750,AT1G65640,AT1G08890,AT5G64630,AT5G04490,AT1G12860,AT5G03560,AT1G05910,AT5G03220,AT1G08640,AT1G79610,AT1G79590,AT5G02760,AT3G15840,AT3G27700,AT4G19110,AT5G62360,AT3G15690,AT1G14370,AT1G60600,AT1G24440,AT5G59850,AT3G02660,AT3G62100,AT5G59300,AT3G61830,AT1G23540,AT3G08030,AT1G25155,AT2G01940,AT1G16000,AT4G11320,AT1G11545,AT4G10360,AT2G20260,AT3G09740,AT2G18410,AT4G08930,AT5G56230,AT1G64640,AT2G13260,AT1G35580,AT1G80030,AT3G58610,AT4G07970,AT5G56090,AT4G07425,AT4G05370,AT3G58620,AT3G10030,AT3G31980,AT4G04955,AT1G48350,AT5G54680,AT5G53880,AT5G54080,AT3G10690,AT4G03110,AT5G53770,AT5G53350,AT4G02940,AT3G08920,AT3G07350,AT2G35620,AT3G54890,AT5G52120,AT5G52010,AT5G51570,AT5G51770,AT4G01130,AT1G17090

AACAATCT	10	4	['20-24']	210	AT3G53720,AT5G51040,AT2G05920,AT4G00620,AT2G40935,AT5G49740,AT3G07700,AT1G06680,AT5G49480,AT3G01120,AT2G33420,AT1G67660,AT1G07950,AT2G28740,AT1G67830,AT1G13280,AT5G48370,AT1G66970,AT1G75800,AT2G29250,AT1G06310,AT1G22050,AT1G13880,AT5G46910,AT3G49590,AT1G11680,AT5G46630,AT1G19060,AT1G14810,AT3G48580,AT1G15820,AT4G17090,AT5G45170,AT2G07050,AT1G20693,AT1G59930,AT3G47650,AT4G16520,AT3G47550,AT5G44390,AT3G47560,AT3G46970,AT3G46620,AT5G43200,AT2G16070,AT3G12600,AT5G42810,AT1G29490,AT1G72030,AT3G45210,AT5G42310,AT3G06180,AT5G04140,AT3G11050,AT3G11170,AT2G25930,AT3G43670,AT1G76730,AT2G36100,AT2G45980,AT1G31410,AT5G28280,AT5G09270,AT1G76450,AT1G76460,AT3G31915,AT3G30840,AT4G38850,AT2G41080,AT2G41040,AT1G73670,AT2G31820,AT5G21170,AT3G52340,AT2G42600,AT1G78180,AT1G73180,AT2G31040,AT1G36390,AT5G24610,AT5G20840,AT5G24380,AT3G22200,AT2G04795,AT2G25310,AT1G31814,AT3G23940,AT5G23060,AT2G30150,AT4G34370,AT5G22310,AT3G51820,AT3G21110,AT3G56700,AT2G36880,AT3G28715,AT2G26170,AT1G58150,AT1G69390,AT5G16910,AT5G16730,AT2G30600,AT3G24260,AT3G24430,AT2G22500,AT2G32765,AT2G22360,AT5G14730,AT2G43550,AT5G14760,AT2G45300,AT2G32880,AT1G53320,AT5G09810,AT5G28140,AT1G53230,AT1G79270,AT1G62340,AT5G12950,AT4G28750,AT1G22690,AT5G11450,AT3G24760,AT5G26340,AT4G27790,AT5G26820,AT1G06200,AT1G78670,AT1G78320,AT1G06750,AT1G12080,AT5G67020,AT1G27480,AT4G26130,AT5G66710,AT5G05950,AT1G07280,AT5G65530,AT1G17460,AT3G21750,AT1G65640,AT1G08890,AT5G64630,AT5G04490,AT1G12860,AT5G03560,AT1G05910,AT5G03220,AT1G08640,AT1G79610,AT1G79590,AT5G02760,AT3G15840,AT3G27700,AT4G19110,AT5G62360,AT3G15690,AT1G14370,AT1G60600,AT1G24440,AT5G59850,AT3G02660,AT3G62100,AT5G59300,AT3G61830,AT1G23540,AT3G08030,AT1G25155,AT2G01940,AT1G16000,AT4G11320,AT1G11545,AT4G10360,AT2G20260,AT3G09740,AT2G18410,AT4G08930,AT5G56230,AT1G64640,AT2G13260,AT1G35580,AT1G80030,AT3G58610,AT4G07970,AT5G56090,AT4G07425,AT4G05370,AT3G58620,AT3G10030,AT3G31980,AT4G04955,AT1G48350,AT5G54680,AT5G53880,AT5G54080,AT3G10690,AT4G03110,AT5G53770,AT5G53350,AT4G02940,AT3G08920,AT3G07350,AT2G35620,AT3G54890,AT5G52120,AT5G52010,AT5G51570,AT5G51770,AT4G01130,AT1G17090
AACAATCT	10	5	['20-24']	210	AT3G53720,AT5G51040,AT2G05920,AT4G00620,AT2G40935,AT5G49740,AT3G07700,AT1G06680,AT5G49480,AT3G01120,AT2G33420,AT1G67660,AT1G07950,AT2G28740,AT1G67830,AT1G13280,AT5G48370,AT1G66970,AT1G75800,AT2G29250,AT1G06310,AT1G22050,AT1G13880,AT5G46910,AT3G49590,AT1G11680,AT5G46630,AT1G19060,AT1G14810,AT3G48580,AT1G15820,AT4G17090,AT5G45170,AT2G07050,AT1G20693,AT1G59930,AT3G47650,AT4G16520,AT3G47550,AT5G44390,AT3G47560,AT3G46970,AT3G46620,AT5G43200,AT2G16070,AT3G12600,AT5G42810,AT1G29490,AT1G72030,AT3G45210,AT5G42310,AT3G06180,AT5G04140,AT3G11050,AT3G11170,AT2G25930,AT3G43670,AT1G76730,AT2G36100,AT2G45980,AT1G31410,AT5G28280,AT5G09270,AT1G76450,AT1G76460,AT3G31915,AT3G30840,AT4G38850,AT2G41080,AT2G41040,AT1G73670,AT2G31820,AT5G21170,AT3G52340,AT2G42600,AT1G78180,AT1G73180,AT2G31040,AT1G36390,AT5G24610,AT5G20840,AT5G24380,AT3G22200,AT2G04795,AT2G25310,AT1G31814,AT3G23940,AT5G23060,AT2G30150,AT4G34370,AT5G22310,AT3G51820,AT3G21110,AT3G56700,AT2G36880,AT3G28715,AT2G26170,AT1G58150,AT1G69390,AT5G16910,AT5G16730,AT2G30600,AT3G24260,AT3G24430,AT2G22500,AT2G32765,AT2G22360,AT5G14730,AT2G43550,AT5G14760,AT2G45300,AT2G32880,AT1G53320,AT5G09810,AT5G28140,AT1G53230,AT1G79270,AT1G62340,AT5G12950,AT4G28750,AT1G22690,AT5G11450,AT3G24760,AT5G26340,AT4G27790,AT5G26820,AT1G06200,AT1G78670,AT1G78320,AT1G06750,AT1G12080,AT5G67020,AT1G27480,AT4G26130,AT5G66710,AT5G05950,AT1G07280,AT5G65530,AT1G17460,AT3G21750,AT1G65640,AT1G08890,AT5G64630,AT5G04490,AT1G12860,AT5G03560,AT1G05910,AT5G03220,AT1G08640,AT1G79610,AT1G79590,AT5G02760,AT3G15840,AT3G27700,AT4G19110,AT5G62360,AT3G15690,AT1G14370,AT1G60600,AT1G24440,AT5G59850,AT3G02660,AT3G62100,AT5G59300,AT3G61830,AT1G23540,AT3G08030,AT1G25155,AT2G01940,AT1G16000,AT4G11320,AT1G11545,AT4G10360,AT2G20260,AT3G09740,AT2G18410,AT4G08930,AT5G56230,AT1G64640,AT2G13260,AT1G35580,AT1G80030,AT3G58610,AT4G07970,AT5G56090,AT4G07425,AT4G05370,AT3G58620,AT3G10030,AT3G31980,AT4G04955,AT1G48350,AT5G54680,AT5G53880,AT5G54080,AT3G10690,AT4G03110,AT5G53770,AT5G53350,AT4G02940,AT3G08920,AT3G07350,AT2G35620,AT3G54890,AT5G52120,AT5G52010,AT5G51570,AT5G51770,AT4G01130,AT1G17090
AACCGACAA	10	1	['8-12', '12-16', '16-20', '20-24']	19	AT4G34100,AT3G56260,AT1G26760,AT1G76550,AT1G21300,AT5G62540,AT5G06690,AT1G62040,AT4G09970,AT1G34000,AT3G44110,AT1G06570,AT3G12030,AT1G08570,AT5G36950,AT1G08720,AT2G45200,AT1G14910,AT5G12250

AACCGACAA	10	2	['8-12', '12-16', '16-20', '20-24']	19	AT4G34100,AT3G56260,AT1G26760,AT1G76550,AT1G21300,AT5G62540,AT5G06690,AT1G62040,AT4G09970,AT1G34000,AT3G44110,AT1G06570,AT3G12030,AT1G08570,AT5G36950,AT1G08720,AT2G45200,AT1G14910,AT5G12250
AACCGACAA	10	3	['8-12', '12-16', '16-20', '20-24']	19	AT4G34100,AT3G56260,AT1G26760,AT1G76550,AT1G21300,AT5G62540,AT5G06690,AT1G62040,AT4G09970,AT1G34000,AT3G44110,AT1G06570,AT3G12030,AT1G08570,AT5G36950,AT1G08720,AT2G45200,AT1G14910,AT5G12250
AACCGACAA	10	4	['8-12', '12-16', '16-20', '20-24']	19	AT4G34100,AT3G56260,AT1G26760,AT1G76550,AT1G21300,AT5G62540,AT5G06690,AT1G62040,AT4G09970,AT1G34000,AT3G44110,AT1G06570,AT3G12030,AT1G08570,AT5G36950,AT1G08720,AT2G45200,AT1G14910,AT5G12250
AACCGACAA	10	5	['8-12', '12-16', '16-20', '20-24']	19	AT4G34100,AT3G56260,AT1G26760,AT1G76550,AT1G21300,AT5G62540,AT5G06690,AT1G62040,AT4G09970,AT1G34000,AT3G44110,AT1G06570,AT3G12030,AT1G08570,AT5G36950,AT1G08720,AT2G45200,AT1G14910,AT5G12250
AACCGACAT	10	1	['0-4', '4-8', '20-24']	19	AT1G76180,AT1G45230,AT2G19650,AT5G65380,AT4G18280,AT1G31580,AT4G24830,AT4G14350,AT1G04910,AT4G00355,AT5G03050,AT5G47620,AT3G16250,AT1G32550,AT1G02270,AT5G36870,AT5G56730,AT1G11850,AT3G05270
AACCGACAT	10	2	['0-4', '4-8', '20-24']	19	AT1G76180,AT1G45230,AT2G19650,AT5G65380,AT4G18280,AT1G31580,AT4G24830,AT4G14350,AT1G04910,AT4G00355,AT5G03050,AT5G47620,AT3G16250,AT1G32550,AT1G02270,AT5G36870,AT5G56730,AT1G11850,AT3G05270
AACCGACAT	10	3	['0-4', '4-8', '20-24']	19	AT1G76180,AT1G45230,AT2G19650,AT5G65380,AT4G18280,AT1G31580,AT4G24830,AT4G14350,AT1G04910,AT4G00355,AT5G03050,AT5G47620,AT3G16250,AT1G32550,AT1G02270,AT5G36870,AT5G56730,AT1G11850,AT3G05270
AACCGACAT	10	4	['0-4', '4-8', '20-24']	19	AT1G76180,AT1G45230,AT2G19650,AT5G65380,AT4G18280,AT1G31580,AT4G24830,AT4G14350,AT1G04910,AT4G00355,AT5G03050,AT5G47620,AT3G16250,AT1G32550,AT1G02270,AT5G36870,AT5G56730,AT1G11850,AT3G05270
AACCGACAT	10	5	['0-4', '4-8', '20-24']	19	AT1G76180,AT1G45230,AT2G19650,AT5G65380,AT4G18280,AT1G31580,AT4G24830,AT4G14350,AT1G04910,AT4G00355,AT5G03050,AT5G47620,AT3G16250,AT1G32550,AT1G02270,AT5G36870,AT5G56730,AT1G11850,AT3G05270
AACCGACCA	10	1	['0-4', '4-8', '8-12', '20-24']	13	AT5G12120,AT2G32180,AT1G21780,AT4G19910,AT3G59350,AT2G15970,AT5G17990,AT3G57170,AT1G78240,AT5G41150,AT1G11680,AT1G19110,AT4G29700
AACCGACCA	10	2	['0-4', '4-8', '8-12', '20-24']	13	AT5G12120,AT2G32180,AT1G21780,AT4G19910,AT3G59350,AT2G15970,AT5G17990,AT3G57170,AT1G78240,AT5G41150,AT1G11680,AT1G19110,AT4G29700
AACCGACCA	10	3	['0-4', '4-8', '8-12', '20-24']	13	AT5G12120,AT2G32180,AT1G21780,AT4G19910,AT3G59350,AT2G15970,AT5G17990,AT3G57170,AT1G78240,AT5G41150,AT1G11680,AT1G19110,AT4G29700
AACCGACCA	10	4	['0-4', '4-8', '8-12', '20-24']	13	AT5G12120,AT2G32180,AT1G21780,AT4G19910,AT3G59350,AT2G15970,AT5G17990,AT3G57170,AT1G78240,AT5G41150,AT1G11680,AT1G19110,AT4G29700
AACCGACCA	10	5	['4-8', '8-12', '20-24', '0-4']	13	AT5G12120,AT2G32180,AT1G21780,AT4G19910,AT3G59350,AT2G15970,AT5G17990,AT3G57170,AT1G78240,AT5G41150,AT1G11680,AT1G19110,AT4G29700
AACCGACCT	10	1	['0-4', '4-8', '16-20']	12	AT3G10410,AT3G03770,AT2G43840,AT5G66920,AT2G46240,AT1G13370,AT2G41040,AT5G63810,AT1G02090,AT1G04750,AT5G54310,AT4G09020
AACCGACCT	10	2	['0-4', '4-8', '16-20']	12	AT3G10410,AT3G03770,AT2G43840,AT5G66920,AT2G46240,AT1G13370,AT2G41040,AT5G63810,AT1G02090,AT1G04750,AT5G54310,AT4G09020
AACCGACCT	10	3	['0-4', '4-8', '16-20']	12	AT3G10410,AT3G03770,AT2G43840,AT5G66920,AT2G46240,AT1G13370,AT2G41040,AT5G63810,AT1G02090,AT1G04750,AT5G54310,AT4G09020
AACCGACCT	10	4	['0-4', '4-8', '16-20']	12	AT3G10410,AT3G03770,AT2G43840,AT5G66920,AT2G46240,AT1G13370,AT2G41040,AT5G63810,AT1G02090,AT1G04750,AT5G54310,AT4G09020
AACCGACCT	10	5	['0-4', '4-8', '16-20']	12	AT3G10410,AT3G03770,AT2G43840,AT5G66920,AT2G46240,AT1G13370,AT2G41040,AT5G63810,AT1G02090,AT1G04750,AT5G54310,AT4G09020
AACCGACGA	10	1	['0-4', '4-8', '8-12', '20-24']	17	AT4G03153,AT5G19260,AT3G04550,AT2G32700,AT2G36880,AT1G25155,AT5G39090,AT2G43840,AT5G55120,AT2G27880,AT1G23205,AT1G11680,AT1G62750,AT4G32530,AT4G04410,AT5G48830,AT1G08720
AACCGACGA	10	2	['0-4', '4-8', '8-12', '20-24']	17	AT4G03153,AT5G19260,AT3G04550,AT2G32700,AT2G36880,AT1G25155,AT5G39090,AT2G43840,AT5G55120,AT2G27880,AT1G23205,AT1G11680,AT1G62750,AT4G32530,AT4G04410,AT5G48830,AT1G08720
AACCGACGA	10	3	['0-4', '4-8', '8-12', '20-24']	17	AT4G03153,AT5G19260,AT3G04550,AT2G32700,AT2G36880,AT1G25155,AT5G39090,AT2G43840,AT5G55120,AT2G27880,AT1G23205,AT1G11680,AT1G62750,AT4G32530,AT4G04410,AT5G48830,AT1G08720

AACCGACGA	10	4	['0-4', '4-8', '8-12', '20-24']	17	AT4G03153,AT5G19260,AT3G04550,AT2G32700,AT2G36880,AT1G25155,AT5G39090,AT2G43840,AT5G55120,AT2G27880,AT1G23205,AT1G11680,AT1G62750,AT4G32530,AT4G04410,AT5G48830,AT1G08720
AACCGACGA	10	5	['0-4', '4-8', '8-12', '20-24']	17	AT4G03153,AT5G19260,AT3G04550,AT2G32700,AT2G36880,AT1G25155,AT5G39090,AT2G43840,AT5G55120,AT2G27880,AT1G23205,AT1G11680,AT1G62750,AT4G32530,AT4G04410,AT5G48830,AT1G08720
AACCGACGT	10	1	['8-12', '12-16', '16-20', '20-24']	17	AT1G08640,AT1G13280,AT1G18070,AT3G09390,AT5G26340,AT5G11070,AT3G57785,AT4G05210,AT3G06150,AT3G09970,AT1G49250,AT5G54980,AT5G06830,AT3G49590,AT1G01230,AT3G54680,AT5G42765
AACCGACGT	10	2	['8-12', '12-16', '16-20', '20-24']	17	AT1G08640,AT1G13280,AT1G18070,AT3G09390,AT5G26340,AT5G11070,AT3G57785,AT4G05210,AT3G06150,AT3G09970,AT1G49250,AT5G54980,AT5G06830,AT3G49590,AT1G01230,AT3G54680,AT5G42765
AACCGACGT	10	3	['8-12', '12-16', '16-20', '20-24']	17	AT1G08640,AT1G13280,AT1G18070,AT3G09390,AT5G26340,AT5G11070,AT3G57785,AT4G05210,AT3G06150,AT3G09970,AT1G49250,AT5G54980,AT5G06830,AT3G49590,AT1G01230,AT3G54680,AT5G42765
AACCGACGT	10	4	['8-12', '12-16', '16-20', '20-24']	17	AT1G08640,AT1G13280,AT1G18070,AT3G09390,AT5G26340,AT5G11070,AT3G57785,AT4G05210,AT3G06150,AT3G09970,AT1G49250,AT5G54980,AT5G06830,AT3G49590,AT1G01230,AT3G54680,AT5G42765
AACCGACGT	10	5	['8-12', '12-16', '16-20', '20-24']	17	AT1G08640,AT1G13280,AT1G18070,AT3G09390,AT5G26340,AT5G11070,AT3G57785,AT4G05210,AT3G06150,AT3G09970,AT1G49250,AT5G54980,AT5G06830,AT3G49590,AT1G01230,AT3G54680,AT5G42765
AACCGACTA	10	1	['0-4', '4-8', '8-12', '12-16', '16-20', '20-24']	22	AT1G60600,AT3G53870,AT5G26820,AT5G58380,AT2G02040,AT3G51240,AT1G17880,AT3G50440,AT4G35950,AT2G15670,AT5G63880,AT2G05620,AT5G17380,AT5G03550,AT5G03190,AT3G45230,AT1G78995,AT2G01760,AT4G01610,AT1G33450,AT3G14270,AT4G29840
AACCGACTA	10	2	['0-4', '4-8', '8-12', '12-16', '16-20', '20-24']	22	AT1G60600,AT3G53870,AT5G26820,AT5G58380,AT2G02040,AT3G51240,AT1G17880,AT3G50440,AT4G35950,AT2G15670,AT5G63880,AT2G05620,AT5G17380,AT5G03550,AT5G03190,AT3G45230,AT1G78995,AT2G01760,AT4G01610,AT1G33450,AT3G14270,AT4G29840
AACCGACTA	10	3	['0-4', '4-8', '8-12', '12-16', '20-24', '16-20']	22	AT1G60600,AT3G53870,AT5G26820,AT5G58380,AT2G02040,AT3G51240,AT1G17880,AT3G50440,AT4G35950,AT2G15670,AT5G63880,AT2G05620,AT5G17380,AT5G03550,AT5G03190,AT3G45230,AT1G78995,AT2G01760,AT4G01610,AT1G33450,AT3G14270,AT4G29840
AACCGACTA	10	4	['0-4', '4-8', '8-12', '12-16', '16-20', '20-24']	22	AT1G60600,AT3G53870,AT5G26820,AT5G58380,AT2G02040,AT3G51240,AT1G17880,AT3G50440,AT4G35950,AT2G15670,AT5G63880,AT2G05620,AT5G17380,AT5G03550,AT5G03190,AT3G45230,AT1G78995,AT2G01760,AT4G01610,AT1G33450,AT3G14270,AT4G29840
AACCGACTA	10	5	['4-8', '8-12', '12-16', '16-20', '20-24', '0-4']	22	AT1G60600,AT3G53870,AT5G26820,AT5G58380,AT2G02040,AT3G51240,AT1G17880,AT3G50440,AT4G35950,AT2G15670,AT5G63880,AT2G05620,AT5G17380,AT5G03550,AT5G03190,AT3G45230,AT1G78995,AT2G01760,AT4G01610,AT1G33450,AT3G14270,AT4G29840
AACCGACTT	10	1	['0-4', '4-8', '8-12', '12-16', '16-20', '20-24']	20	AT2G23610,AT2G25740,AT4G37290,AT2G46400,AT2G34140,AT1G70820,AT4G20280,AT2G37520,AT4G37830,AT1G60490,AT1G09870,AT4G27790,AT1G61520,AT4G36040,AT1G09390,AT1G78670,AT1G73470,AT1G01250,AT1G75950,AT5G08590
AACCGACTT	10	2	['0-4', '4-8', '8-12', '16-20', '20-24', '12-16']	20	AT2G23610,AT2G25740,AT4G37290,AT2G46400,AT2G34140,AT1G70820,AT4G20280,AT2G37520,AT4G37830,AT1G60490,AT1G09870,AT4G27790,AT1G61520,AT4G36040,AT1G09390,AT1G78670,AT1G73470,AT1G01250,AT1G75950,AT5G08590
AACCGACTT	10	3	['0-4', '4-8', '8-12', '12-16', '16-20', '20-24']	20	AT2G23610,AT2G25740,AT4G37290,AT2G46400,AT2G34140,AT1G70820,AT4G20280,AT2G37520,AT4G37830,AT1G60490,AT1G09870,AT4G27790,AT1G61520,AT4G36040,AT1G09390,AT1G78670,AT1G73470,AT1G01250,AT1G75950,AT5G08590
AACCGACTT	10	4	['0-4', '4-8', '8-12', '12-16', '16-20', '20-24']	20	AT2G23610,AT2G25740,AT4G37290,AT2G46400,AT2G34140,AT1G70820,AT4G20280,AT2G37520,AT4G37830,AT1G60490,AT1G09870,AT4G27790,AT1G61520,AT4G36040,AT1G09390,AT1G78670,AT1G73470,AT1G01250,AT1G75950,AT5G08590
AACCGACTT	10	5	['0-4', '4-8', '8-12', '12-16', '16-20', '20-24']	20	AT2G23610,AT2G25740,AT4G37290,AT2G46400,AT2G34140,AT1G70820,AT4G20280,AT2G37520,AT4G37830,AT1G60490,AT1G09870,AT4G27790,AT1G61520,AT4G36040,AT1G09390,AT1G78670,AT1G73470,AT1G01250,AT1G75950,AT5G08590

AACCTAAC	10	1	['12-16']	187	ATMG00650,AT5G50990,AT4G00820,AT1G34220,AT3G53680,AT3G01660,AT2G40780,AT4G00400,AT1G32060,AT2G45140,AT3G01120,AT1G75830,AT1G67740,AT1G28530,AT4G25530,AT5G47890,AT1G17745,AT1G10760,AT4G15440,AT5G47390,AT3G49250,AT1G19140,AT5G45820,AT1G15740,AT2G31070,AT5G44520,AT3G47650,AT4G16410,AT3G47430,AT3G47550,AT5G44070,AT5G43830,AT4G15530,AT5G43200,AT3G46170,AT1G71030,AT5G42470,AT4G14440,AT1G56700,AT3G45230,AT1G64330,AT1G66840,AT2G03110,AT5G41810,AT5G41050,AT3G44250,AT3G11200,AT1G05570,AT5G40850,AT2G45990,AT2G04240,AT1G37537,AT1G53670,AT2G39450,AT1G76550,AT2G31710,AT5G38550,AT5G19760,AT1G67970,AT4G38570,AT2G21240,AT5G37640,AT3G28340,AT4G38360,AT2G31750,AT2G42600,AT5G36170,AT2G23420,AT1G73180,AT2G25210,AT4G37480,AT4G37020,AT3G20970,AT2G02515,AT1G60000,AT1G31812,AT5G23060,AT2G30150,AT5G22310,AT3G56880,AT1G80500,AT5G19260,AT1G16680,AT4G34030,AT4G34150,AT4G33670,AT2G04450,AT4G33150,AT1G68160,AT3G13100,AT2G22430,AT3G14900,AT2G21130,AT5G15580,AT5G35380,AT5G14240,AT3G29760,AT4G30470,AT5G12210,AT1G30800,AT1G54570,AT2G24880,AT1G09230,AT5G26820,AT5G26030,AT5G09760,AT4G27585,AT5G67200,AT5G67300,AT1G21500,AT5G07860,AT5G67030,AT4G26530,AT1G67110,AT5G07400,AT1G09380,AT4G25660,AT3G23280,AT1G04750,AT5G65380,AT1G65560,AT5G64860,AT3G19500,AT4G24220,AT5G04490,AT1G32920,AT1G12860,AT1G09795,AT1G09870,AT4G23750,AT3G16190,AT4G22930,AT1G20020,AT5G03795,AT4G22600,AT4G22540,AT4G22753,AT1G08640,AT5G02830,AT3G15840,AT5G61820,AT1G60650,AT4G18270,AT3G16560,AT1G07940,AT1G03970,AT5G59830,AT1G63340,AT4G12480,AT1G23490,AT3G02830,AT1G76100,AT2G43018,AT1G76240,AT2G01940,AT1G16000,AT5G57760,AT3G08650,AT2G18280,AT3G59940,AT3G59770,AT2G18300,AT4G08690,AT5G56240,AT5G56020,AT1G77920,AT4G05180,AT4G05100,AT2G15670,AT5G54950,AT3G57550,AT3G10050,AT1G70782,AT2G27360,AT4G03110,AT1G73020,AT3G08940,AT4G03050,AT3G56050,AT3G55670,AT4G02540,AT3G03380,AT3G55010,AT1G21680,AT3G04870,AT3G54240,AT3G54300
AACCTAAC	10	2	['12-16']	187	ATMG00650,AT5G50990,AT4G00820,AT1G34220,AT3G53680,AT3G01660,AT2G40780,AT4G00400,AT1G32060,AT2G45140,AT3G01120,AT1G75830,AT1G67740,AT1G28530,AT4G25530,AT5G47890,AT1G17745,AT1G10760,AT4G15440,AT5G47390,AT3G49250,AT1G19140,AT5G45820,AT1G15740,AT2G31070,AT5G44520,AT3G47650,AT4G16410,AT3G47430,AT3G47550,AT5G44070,AT5G43830,AT4G15530,AT5G43200,AT3G46170,AT1G71030,AT5G42470,AT4G14440,AT1G56700,AT3G45230,AT1G64330,AT1G66840,AT2G03110,AT5G41810,AT5G41050,AT3G44250,AT3G11200,AT1G05570,AT5G40850,AT2G45990,AT2G04240,AT1G37537,AT1G53670,AT2G39450,AT1G76550,AT2G31710,AT5G38550,AT5G19760,AT1G67970,AT4G38570,AT2G21240,AT5G37640,AT3G28340,AT4G38360,AT2G31750,AT2G42600,AT5G36170,AT2G23420,AT1G73180,AT2G25210,AT4G37480,AT4G37020,AT3G20970,AT2G02515,AT1G60000,AT1G31812,AT5G23060,AT2G30150,AT5G22310,AT3G56880,AT1G80500,AT5G19260,AT1G16680,AT4G34030,AT4G34150,AT4G33670,AT2G04450,AT4G33150,AT1G68160,AT3G13100,AT2G22430,AT3G14900,AT2G21130,AT5G15580,AT5G35380,AT5G14240,AT3G29760,AT4G30470,AT5G12210,AT1G30800,AT1G54570,AT2G24880,AT1G09230,AT5G26820,AT5G26030,AT5G09760,AT4G27585,AT5G67200,AT5G67300,AT1G21500,AT5G07860,AT5G67030,AT4G26530,AT1G67110,AT5G07400,AT1G09380,AT4G25660,AT3G23280,AT1G04750,AT5G65380,AT1G65560,AT5G64860,AT3G19500,AT4G24220,AT5G04490,AT1G32920,AT1G12860,AT1G09795,AT1G09870,AT4G23750,AT3G16190,AT4G22930,AT1G20020,AT5G03795,AT4G22600,AT4G22540,AT4G22753,AT1G08640,AT5G02830,AT3G15840,AT5G61820,AT1G60650,AT4G18270,AT3G16560,AT1G07940,AT1G03970,AT5G59830,AT1G63340,AT4G12480,AT1G23490,AT3G02830,AT1G76100,AT2G43018,AT1G76240,AT2G01940,AT1G16000,AT5G57760,AT3G08650,AT2G18280,AT3G59940,AT3G59770,AT2G18300,AT4G08690,AT5G56240,AT5G56020,AT1G77920,AT4G05180,AT4G05100,AT2G15670,AT5G54950,AT3G57550,AT3G10050,AT1G70782,AT2G27360,AT4G03110,AT1G73020,AT3G08940,AT4G03050,AT3G56050,AT3G55670,AT4G02540,AT3G03380,AT3G55010,AT1G21680,AT3G04870,AT3G54240,AT3G54300

AACCTAAC	10	3	['12-16']	187	ATMG00650,AT5G50990,AT4G00820,AT1G34220,AT3G53680,AT3G01660,AT2G40780,AT4G00400,AT1G32060,AT2G45140,AT3G01120,AT1G75830,AT1G67740,AT1G28530,AT4G25530,AT5G47890,AT1G17745,AT1G10760,AT4G15440,AT5G47390,AT3G49250,AT1G19140,AT5G45820,AT1G15740,AT2G31070,AT5G44520,AT3G47650,AT4G16410,AT3G47430,AT3G47550,AT5G44070,AT5G43830,AT4G15530,AT5G43200,AT3G46170,AT1G71030,AT5G42470,AT4G14440,AT1G56700,AT3G45230,AT1G64330,AT1G66840,AT2G03110,AT5G41810,AT5G41050,AT3G44250,AT3G11200,AT1G05570,AT5G40850,AT2G45990,AT2G04240,AT1G37537,AT1G53670,AT2G39450,AT1G76550,AT2G31710,AT5G38550,AT5G19760,AT1G67970,AT4G38570,AT2G21240,AT5G37640,AT3G28340,AT4G38360,AT2G31750,AT2G42600,AT5G36170,AT2G23420,AT1G73180,AT2G25210,AT4G37480,AT4G37020,AT3G20970,AT2G02515,AT1G60000,AT1G31812,AT5G23060,AT2G30150,AT5G22310,AT3G56880,AT1G80500,AT5G19260,AT1G16680,AT4G34030,AT4G34150,AT4G33670,AT2G04450,AT4G33150,AT1G68160,AT3G13100,AT2G22430,AT3G14900,AT2G21130,AT5G15580,AT5G35380,AT5G14240,AT3G29760,AT4G30470,AT5G12210,AT1G30800,AT1G54570,AT2G24880,AT1G09230,AT5G26820,AT5G26030,AT5G09760,AT4G27585,AT5G67200,AT5G67300,AT1G21500,AT5G07860,AT5G67030,AT4G26530,AT1G67110,AT5G07400,AT1G09380,AT4G25660,AT3G23280,AT1G04750,AT5G65380,AT1G65560,AT5G64860,AT3G19500,AT4G24220,AT5G04490,AT1G32920,AT1G12860,AT1G09795,AT1G09870,AT4G23750,AT3G16190,AT4G22930,AT1G20020,AT5G03795,AT4G22600,AT4G22540,AT4G22753,AT1G08640,AT5G02830,AT3G15840,AT5G61820,AT1G60650,AT4G18270,AT3G16560,AT1G07940,AT1G03970,AT5G59830,AT1G63340,AT4G12480,AT1G23490,AT3G02830,AT1G76100,AT2G43018,AT1G76240,AT2G01940,AT1G16000,AT5G57760,AT3G08650,AT2G18280,AT3G59940,AT3G59770,AT2G18300,AT4G08690,AT5G56240,AT5G56020,AT1G77920,AT4G05180,AT4G05100,AT2G15670,AT5G54950,AT3G57550,AT3G10050,AT1G70782,AT2G27360,AT4G03110,AT1G73020,AT3G08940,AT4G03050,AT3G56050,AT3G55670,AT4G02540,AT3G03380,AT3G55010,AT1G21680,AT3G04870,AT3G54240,AT3G54300
AACCTAAC	10	4	['12-16']	187	ATMG00650,AT5G50990,AT4G00820,AT1G34220,AT3G53680,AT3G01660,AT2G40780,AT4G00400,AT1G32060,AT2G45140,AT3G01120,AT1G75830,AT1G67740,AT1G28530,AT4G25530,AT5G47890,AT1G17745,AT1G10760,AT4G15440,AT5G47390,AT3G49250,AT1G19140,AT5G45820,AT1G15740,AT2G31070,AT5G44520,AT3G47650,AT4G16410,AT3G47430,AT3G47550,AT5G44070,AT5G43830,AT4G15530,AT5G43200,AT3G46170,AT1G71030,AT5G42470,AT4G14440,AT1G56700,AT3G45230,AT1G64330,AT1G66840,AT2G03110,AT5G41810,AT5G41050,AT3G44250,AT3G11200,AT1G05570,AT5G40850,AT2G45990,AT2G04240,AT1G37537,AT1G53670,AT2G39450,AT1G76550,AT2G31710,AT5G38550,AT5G19760,AT1G67970,AT4G38570,AT2G21240,AT5G37640,AT3G28340,AT4G38360,AT2G31750,AT2G42600,AT5G36170,AT2G23420,AT1G73180,AT2G25210,AT4G37480,AT4G37020,AT3G20970,AT2G02515,AT1G60000,AT1G31812,AT5G23060,AT2G30150,AT5G22310,AT3G56880,AT1G80500,AT5G19260,AT1G16680,AT4G34030,AT4G34150,AT4G33670,AT2G04450,AT4G33150,AT1G68160,AT3G13100,AT2G22430,AT3G14900,AT2G21130,AT5G15580,AT5G35380,AT5G14240,AT3G29760,AT4G30470,AT5G12210,AT1G30800,AT1G54570,AT2G24880,AT1G09230,AT5G26820,AT5G26030,AT5G09760,AT4G27585,AT5G67200,AT5G67300,AT1G21500,AT5G07860,AT5G67030,AT4G26530,AT1G67110,AT5G07400,AT1G09380,AT4G25660,AT3G23280,AT1G04750,AT5G65380,AT1G65560,AT5G64860,AT3G19500,AT4G24220,AT5G04490,AT1G32920,AT1G12860,AT1G09795,AT1G09870,AT4G23750,AT3G16190,AT4G22930,AT1G20020,AT5G03795,AT4G22600,AT4G22540,AT4G22753,AT1G08640,AT5G02830,AT3G15840,AT5G61820,AT1G60650,AT4G18270,AT3G16560,AT1G07940,AT1G03970,AT5G59830,AT1G63340,AT4G12480,AT1G23490,AT3G02830,AT1G76100,AT2G43018,AT1G76240,AT2G01940,AT1G16000,AT5G57760,AT3G08650,AT2G18280,AT3G59940,AT3G59770,AT2G18300,AT4G08690,AT5G56240,AT5G56020,AT1G77920,AT4G05180,AT4G05100,AT2G15670,AT5G54950,AT3G57550,AT3G10050,AT1G70782,AT2G27360,AT4G03110,AT1G73020,AT3G08940,AT4G03050,AT3G56050,AT3G55670,AT4G02540,AT3G03380,AT3G55010,AT1G21680,AT3G04870,AT3G54240,AT3G54300

AACCTAAC	10	5	['12-16']	187	ATMG00650,AT5G50990,AT4G00820,AT1G34220,AT3G53680,AT3G01660,AT2G40780,AT4G00400,AT1G32060,AT2G45140,AT3G01120,AT1G75830,AT1G67740,AT1G28530,AT4G25530,AT5G47890,AT1G17745,AT1G10760,AT4G15440,AT5G47390,AT3G49250,AT1G19140,AT5G45820,AT1G15740,AT2G31070,AT5G44520,AT3G47650,AT4G16410,AT3G47430,AT3G47550,AT5G44070,AT5G43830,AT4G15530,AT5G43200,AT3G46170,AT1G71030,AT5G42470,AT4G14440,AT1G56700,AT3G45230,AT1G64330,AT1G66840,AT2G03110,AT5G41810,AT5G41050,AT3G44250,AT3G11200,AT1G05570,AT5G40850,AT2G45990,AT2G04240,AT1G37537,AT1G53670,AT2G39450,AT1G76550,AT2G31710,AT5G38550,AT5G19760,AT1G67970,AT4G38570,AT2G21240,AT5G37640,AT3G28340,AT4G38360,AT2G31750,AT2G42600,AT5G36170,AT2G23420,AT1G73180,AT2G25210,AT4G37480,AT4G37020,AT3G20970,AT2G02515,AT1G60000,AT1G31812,AT5G23060,AT2G30150,AT5G22310,AT3G56880,AT1G80500,AT5G19260,AT1G16680,AT4G34030,AT4G34150,AT4G33670,AT2G04450,AT4G33150,AT1G68160,AT3G13100,AT2G22430,AT3G14900,AT2G21130,AT5G15580,AT5G35380,AT5G14240,AT3G29760,AT4G30470,AT5G12210,AT1G30800,AT1G54570,AT2G24880,AT1G09230,AT5G26820,AT5G26030,AT5G09760,AT4G27585,AT5G67200,AT5G67300,AT1G21500,AT5G07860,AT5G67030,AT4G26530,AT1G67110,AT5G07400,AT1G09380,AT4G25660,AT3G23280,AT1G04750,AT5G65380,AT1G65560,AT5G64860,AT3G19500,AT4G24220,AT5G04490,AT1G32920,AT1G12860,AT1G09795,AT1G09870,AT4G23750,AT3G16190,AT4G22930,AT1G20020,AT5G03795,AT4G22600,AT4G22540,AT4G22753,AT1G08640,AT5G02830,AT3G15840,AT5G61820,AT1G60650,AT4G18270,AT3G16560,AT1G07940,AT1G03970,AT5G59830,AT1G63340,AT4G12480,AT1G23490,AT3G02830,AT1G76100,AT2G43018,AT1G76240,AT2G01940,AT1G16000,AT5G57760,AT3G08650,AT2G18280,AT3G59940,AT3G59770,AT2G18300,AT4G08690,AT5G56240,AT5G56020,AT1G77920,AT4G05180,AT4G05100,AT2G15670,AT5G54950,AT3G57550,AT3G10050,AT1G70782,AT2G27360,AT4G03110,AT1G73020,AT3G08940,AT4G03050,AT3G56050,AT3G55670,AT4G02540,AT3G03380,AT3G55010,AT1G21680,AT3G04870,AT3G54240,AT3G54300
AACCTAACCT	10	1	['4-8', '12-16', '16-20', '20-24']	16	AT1G08640,AT2G24880,AT2G43018,AT1G76240,AT2G45990,AT3G47650,AT5G04490,AT4G16410,AT4G03050,AT4G05180,AT4G23750,AT4G25660,AT3G23280,AT1G31812,AT2G27360,AT3G54300
AACCTAACCT	10	2	['4-8', '12-16', '16-20', '20-24']	16	AT1G08640,AT2G24880,AT2G43018,AT1G76240,AT2G45990,AT3G47650,AT5G04490,AT4G16410,AT4G03050,AT4G05180,AT4G23750,AT4G25660,AT3G23280,AT1G31812,AT2G27360,AT3G54300
AACCTAACCT	10	3	['4-8', '12-16', '16-20', '20-24']	16	AT1G08640,AT2G24880,AT2G43018,AT1G76240,AT2G45990,AT3G47650,AT5G04490,AT4G16410,AT4G03050,AT4G05180,AT4G23750,AT4G25660,AT3G23280,AT1G31812,AT2G27360,AT3G54300
AACCTAACCT	10	4	['4-8', '12-16', '16-20', '20-24']	16	AT1G08640,AT2G24880,AT2G43018,AT1G76240,AT2G45990,AT3G47650,AT5G04490,AT4G16410,AT4G03050,AT4G05180,AT4G23750,AT4G25660,AT3G23280,AT1G31812,AT2G27360,AT3G54300
AACCTAACCT	10	5	['4-8', '12-16', '16-20', '20-24']	16	AT1G08640,AT2G24880,AT2G43018,AT1G76240,AT2G45990,AT3G47650,AT5G04490,AT4G16410,AT4G03050,AT4G05180,AT4G23750,AT4G25660,AT3G23280,AT1G31812,AT2G27360,AT3G54300
AACCTACC	10	1	['4-8', '16-20', '20-24']	63	AT5G27280,AT3G53470,AT5G60540,AT3G62410,AT2G40150,AT1G50020,AT4G00660,AT4G00370,AT3G52950,AT3G53030,AT1G19920,AT3G52290,AT2G31750,AT1G16520,AT2G33420,AT5G35540,AT3G03770,AT5G08580,AT3G27050,AT3G26710,AT5G57630,AT1G08130,AT5G66710,AT4G10360,AT1G26560,AT4G25760,AT1G65060,AT4G08970,AT1G07010,AT4G14930,AT5G65430,AT4G24860,AT1G43930,AT4G34190,AT5G05110,AT5G45300,AT3G04550,AT5G64460,AT5G44910,AT1G54500,AT4G33490,AT3G14620,AT4G33430,AT5G17780,AT4G16190,AT5G16970,AT1G23010,AT2G07490,AT4G22310,AT2G41870,AT1G78480,AT5G42470,AT2G41720,AT5G27650,AT5G14640,AT3G55760,AT1G61210,AT5G02160,AT5G61990,AT1G72800,AT1G02140,AT3G14270,AT1G01790
AACCTACC	10	2	['4-8', '16-20', '20-24']	63	AT5G27280,AT3G53470,AT5G60540,AT3G62410,AT2G40150,AT1G50020,AT4G00660,AT4G00370,AT3G52950,AT3G53030,AT1G19920,AT3G52290,AT2G31750,AT1G16520,AT2G33420,AT5G35540,AT3G03770,AT5G08580,AT3G27050,AT3G26710,AT5G57630,AT1G08130,AT5G66710,AT4G10360,AT1G26560,AT4G25760,AT1G65060,AT4G08970,AT1G07010,AT4G14930,AT5G65430,AT4G24860,AT1G43930,AT4G34190,AT5G05110,AT5G45300,AT3G04550,AT5G64460,AT5G44910,AT1G54500,AT4G33490,AT3G14620,AT4G33430,AT5G17780,AT4G16190,AT5G16970,AT1G23010,AT2G07490,AT4G22310,AT2G41870,AT1G78480,AT5G42470,AT2G41720,AT5G27650,AT5G14640,AT3G55760,AT1G61210,AT5G02160,AT5G61990,AT1G72800,AT1G02140,AT3G14270,AT1G01790

AACCTACC	10	3	['4-8', '20-24', '16-20']	63	AT5G27280,AT3G53470,AT5G60540,AT3G62410,AT2G40150,AT1G50020,AT4G00660,AT4G00370,AT3G52950,AT3G53030,AT1G19920,AT3G52290,AT2G31750,AT1G16520,AT2G33420,AT5G35540,AT3G03770,AT5G08580,AT3G27050,AT3G26710,AT5G57630,AT1G08130,AT5G66710,AT4G10360,AT1G26560,AT4G25760,AT1G65060,AT4G08970,AT1G07010,AT4G14930,AT5G65430,AT4G24860,AT1G43930,AT4G34190,AT5G05110,AT5G45300,AT3G04550,AT5G64460,AT5G44910,AT1G54500,AT4G33490,AT3G14620,AT4G33430,AT5G17780,AT4G16190,AT5G16970,AT1G23010,AT2G07490,AT4G22310,AT2G41870,AT1G78480,AT5G42470,AT2G41720,AT5G27650,AT5G14640,AT3G55760,AT1G61210,AT5G02160,AT5G61990,AT1G72800,AT1G02140,AT3G14270,AT1G01790
AACCTACC	10	4	['4-8', '16-20', '20-24']	63	AT5G27280,AT3G53470,AT5G60540,AT3G62410,AT2G40150,AT1G50020,AT4G00660,AT4G00370,AT3G52950,AT3G53030,AT1G19920,AT3G52290,AT2G31750,AT1G16520,AT2G33420,AT5G35540,AT3G03770,AT5G08580,AT3G27050,AT3G26710,AT5G57630,AT1G08130,AT5G66710,AT4G10360,AT1G26560,AT4G25760,AT1G65060,AT4G08970,AT1G07010,AT4G14930,AT5G65430,AT4G24860,AT1G43930,AT4G34190,AT5G05110,AT5G45300,AT3G04550,AT5G64460,AT5G44910,AT1G54500,AT4G33490,AT3G14620,AT4G33430,AT5G17780,AT4G16190,AT5G16970,AT1G23010,AT2G07490,AT4G22310,AT2G41870,AT1G78480,AT5G42470,AT2G41720,AT5G27650,AT5G14640,AT3G55760,AT1G61210,AT5G02160,AT5G61990,AT1G72800,AT1G02140,AT3G14270,AT1G01790
AACCTACC	10	5	['4-8', '16-20', '20-24']	63	AT5G27280,AT3G53470,AT5G60540,AT3G62410,AT2G40150,AT1G50020,AT4G00660,AT4G00370,AT3G52950,AT3G53030,AT1G19920,AT3G52290,AT2G31750,AT1G16520,AT2G33420,AT5G35540,AT3G03770,AT5G08580,AT3G27050,AT3G26710,AT5G57630,AT1G08130,AT5G66710,AT4G10360,AT1G26560,AT4G25760,AT1G65060,AT4G08970,AT1G07010,AT4G14930,AT5G65430,AT4G24860,AT1G43930,AT4G34190,AT5G05110,AT5G45300,AT3G04550,AT5G64460,AT5G44910,AT1G54500,AT4G33490,AT3G14620,AT4G33430,AT5G17780,AT4G16190,AT5G16970,AT1G23010,AT2G07490,AT4G22310,AT2G41870,AT1G78480,AT5G42470,AT2G41720,AT5G27650,AT5G14640,AT3G55760,AT1G61210,AT5G02160,AT5G61990,AT1G72800,AT1G02140,AT3G14270,AT1G01790
AACGGCTA	10	1	['0-4']	62	AT5G27150,AT3G05500,AT1G38065,AT2G07730,AT5G60680,AT3G02630,AT5G11260,AT1G78670,AT1G70410,AT2G38000,AT1G76030,AT5G35840,AT1G21460,AT5G67200,AT4G27320,AT5G67390,AT5G35540,AT5G58070,AT4G26520,AT2G29560,AT4G26530,AT5G66950,AT1G10760,AT3G18600,AT1G01540,AT5G65630,AT5G65660,AT1G19110,AT2G07140,AT5G05740,AT1G74560,AT2G21300,AT5G23060,AT2G23760,AT3G56690,AT4G17640,AT2G40400,AT3G13470,AT5G19210,AT1G63800,AT5G44780,AT4G23630,AT5G43810,AT1G55330,AT4G04330,AT2G25510,AT5G16260,AT4G03600,AT4G03110,AT1G03530,AT5G16310,AT1G54100,AT2G03680,AT2G46780,AT3G06180,AT5G52180,AT2G35690,AT5G52040,AT3G16170,AT4G01610,AT5G11890,AT5G27720
AACGGCTA	10	2	['0-4']	62	AT5G27150,AT3G05500,AT1G38065,AT2G07730,AT5G60680,AT3G02630,AT5G11260,AT1G78670,AT1G70410,AT2G38000,AT1G76030,AT5G35840,AT1G21460,AT5G67200,AT4G27320,AT5G67390,AT5G35540,AT5G58070,AT4G26520,AT2G29560,AT4G26530,AT5G66950,AT1G10760,AT3G18600,AT1G01540,AT5G65630,AT5G65660,AT1G19110,AT2G07140,AT5G05740,AT1G74560,AT2G21300,AT5G23060,AT2G23760,AT3G56690,AT4G17640,AT2G40400,AT3G13470,AT5G19210,AT1G63800,AT5G44780,AT4G23630,AT5G43810,AT1G55330,AT4G04330,AT2G25510,AT5G16260,AT4G03600,AT4G03110,AT1G03530,AT5G16310,AT1G54100,AT2G03680,AT2G46780,AT3G06180,AT5G52180,AT2G35690,AT5G52040,AT3G16170,AT4G01610,AT5G11890,AT5G27720
AACGGCTA	10	3	['0-4']	62	AT5G27150,AT3G05500,AT1G38065,AT2G07730,AT5G60680,AT3G02630,AT5G11260,AT1G78670,AT1G70410,AT2G38000,AT1G76030,AT5G35840,AT1G21460,AT5G67200,AT4G27320,AT5G67390,AT5G35540,AT5G58070,AT4G26520,AT2G29560,AT4G26530,AT5G66950,AT1G10760,AT3G18600,AT1G01540,AT5G65630,AT5G65660,AT1G19110,AT2G07140,AT5G05740,AT1G74560,AT2G21300,AT5G23060,AT2G23760,AT3G56690,AT4G17640,AT2G40400,AT3G13470,AT5G19210,AT1G63800,AT5G44780,AT4G23630,AT5G43810,AT1G55330,AT4G04330,AT2G25510,AT5G16260,AT4G03600,AT4G03110,AT1G03530,AT5G16310,AT1G54100,AT2G03680,AT2G46780,AT3G06180,AT5G52180,AT2G35690,AT5G52040,AT3G16170,AT4G01610,AT5G11890,AT5G27720

AACGGCTA	10	4	['0-4']	62	AT5G27150,AT3G05500,AT1G38065,AT2G07730,AT5G60680,AT3G02630,AT5G11260,AT1G78670,AT1G70410,AT2G38000,AT1G76030,AT5G35840,AT1G21460,AT5G67200,AT4G27320,AT5G67390,AT5G35540,AT5G58070,AT4G26520,AT2G29560,AT4G26530,AT5G66950,AT1G10760,AT3G18600,AT1G01540,AT5G65630,AT5G65660,AT1G19110,AT2G07140,AT5G05740,AT1G74560,AT2G21300,AT5G23060,AT2G23760,AT3G56690,AT4G17640,AT2G40400,AT3G13470,AT5G19210,AT1G63800,AT5G44780,AT4G23630,AT5G43810,AT1G55330,AT4G04330,AT2G25510,AT5G16260,AT4G03600,AT4G03110,AT1G03530,AT5G16310,AT1G54100,AT2G03680,AT2G46780,AT3G06180,AT5G52180,AT2G35690,AT5G52040,AT3G16170,AT4G01610,AT5G11890,AT5G27720
AACGGCTA	10	5	['0-4']	62	AT5G27150,AT3G05500,AT1G38065,AT2G07730,AT5G60680,AT3G02630,AT5G11260,AT1G78670,AT1G70410,AT2G38000,AT1G76030,AT5G35840,AT1G21460,AT5G67200,AT4G27320,AT5G67390,AT5G35540,AT5G58070,AT4G26520,AT2G29560,AT4G26530,AT5G66950,AT1G10760,AT3G18600,AT1G01540,AT5G65630,AT5G65660,AT1G19110,AT2G07140,AT5G05740,AT1G74560,AT2G21300,AT5G23060,AT2G23760,AT3G56690,AT4G17640,AT2G40400,AT3G13470,AT5G19210,AT1G63800,AT5G44780,AT4G23630,AT5G43810,AT1G55330,AT4G04330,AT2G25510,AT5G16260,AT4G03600,AT4G03110,AT1G03530,AT5G16310,AT1G54100,AT2G03680,AT2G46780,AT3G06180,AT5G52180,AT2G35690,AT5G52040,AT3G16170,AT4G01610,AT5G11890,AT5G27720
AAGACGTAG	10	1	['4-8', '8-12', '16-20', '20-24']	17	AT3G03830,AT1G63670,AT1G29490,AT3G14900,AT3G58140,AT2G21130,AT1G50120,AT5G04760,AT5G62200,AT4G36390,AT3G55010,AT4G23100,AT5G61850,AT2G18390,AT1G72630,AT2G45440,AT1G74370
AAGACGTAG	10	2	['4-8', '8-12', '16-20', '20-24']	17	AT3G03830,AT1G63670,AT1G29490,AT3G14900,AT3G58140,AT2G21130,AT1G50120,AT5G04760,AT5G62200,AT4G36390,AT3G55010,AT4G23100,AT5G61850,AT2G18390,AT1G72630,AT2G45440,AT1G74370
AAGACGTAG	10	3	['4-8', '8-12', '20-24', '16-20']	17	AT3G03830,AT1G63670,AT1G29490,AT3G14900,AT3G58140,AT2G21130,AT1G50120,AT5G04760,AT5G62200,AT4G36390,AT3G55010,AT4G23100,AT5G61850,AT2G18390,AT1G72630,AT2G45440,AT1G74370
AAGACGTAG	10	4	['4-8', '8-12', '16-20', '20-24']	17	AT3G03830,AT1G63670,AT1G29490,AT3G14900,AT3G58140,AT2G21130,AT1G50120,AT5G04760,AT5G62200,AT4G36390,AT3G55010,AT4G23100,AT5G61850,AT2G18390,AT1G72630,AT2G45440,AT1G74370
AAGACGTAG	10	5	['4-8', '8-12', '16-20', '20-24']	17	AT3G03830,AT1G63670,AT1G29490,AT3G14900,AT3G58140,AT2G21130,AT1G50120,AT5G04760,AT5G62200,AT4G36390,AT3G55010,AT4G23100,AT5G61850,AT2G18390,AT1G72630,AT2G45440,AT1G74370
AATACTAAT	10	1	['8-12']	116	AT3G53500,AT5G49910,AT3G07650,AT2G47600,AT5G49480,AT2G33330,AT1G13280,AT4G01070,AT1G02305,AT4G17230,AT1G13180,AT2G27130,AT2G32280,AT1G01620,AT1G01500,AT1G11755,AT4G15140,AT1G14920,AT5G45830,AT1G20693,AT5G44520,AT1G18170,AT2G16860,AT3G47500,AT1G23980,AT1G68920,AT4G14270,AT1G75240,AT2G44670,AT1G32130,AT2G34580,AT2G15320,AT1G33270,AT5G37600,AT5G13120,AT3G28270,AT5G21060,AT2G42580,AT2G38230,AT2G34140,AT5G24010,AT2G02515,AT4G36640,AT1G26880,AT5G22310,AT4G34370,AT5G19240,AT2G19270,AT2G36870,AT4G33985,AT3G15220,AT3G27300,AT3G27840,AT1G55850,AT1G55910,AT3G26520,AT1G19450,AT1G10660,AT3G24760,AT1G09160,AT4G28080,AT3G13370,AT3G12710,AT3G18440,AT2G25180,AT5G24930,AT3G27090,AT5G67160,AT4G26050,AT5G66920,AT3G16960,AT1G09380,AT5G05950,AT3G19930,AT1G65640,AT3G21690,AT5G62540,AT2G17330,AT1G21130,AT1G23080,AT5G62280,AT5G01820,AT4G19170,AT5G01750,AT5G61380,AT1G61140,AT1G14370,AT1G60600,AT1G67360,AT1G03900,AT4G12390,AT1G76100,AT5G58410,AT3G60680,AT3G60350,AT1G10670,AT4G10360,AT3G09750,AT3G59930,AT3G59920,AT4G08930,AT2G22570,AT3G59020,AT3G10850,AT1G35580,AT3G57280,AT1G74940,AT2G07490,AT2G28200,AT3G56410,AT4G03190,AT2G01290,AT1G49260,AT3G54900,AT4G01280,AT4G00270

AATACTAAT	10	2	['8-12']	116	AT3G53500,AT5G49910,AT3G07650,AT2G47600,AT5G49480,AT2G33330,AT1G13280,AT4G01070,AT1G02305,AT4G17230,AT1G13180,AT2G27130,AT2G32280,AT1G01620,AT1G01500,AT1G11755,AT4G15140,AT1G14920,AT5G45830,AT1G20693,AT5G44520,AT1G18170,AT2G16860,AT3G47500,AT1G23980,AT1G68920,AT4G14270,AT1G75240,AT2G44670,AT1G32130,AT2G34580,AT2G15320,AT1G33270,AT5G37600,AT5G13120,AT3G28270,AT5G21060,AT2G42580,AT2G38230,AT2G34140,AT5G24010,AT2G02515,AT4G36640,AT1G26880,AT5G22310,AT4G34370,AT5G19240,AT2G19270,AT2G36870,AT4G33985,AT3G15220,AT3G27300,AT3G27840,AT1G55850,AT1G55910,AT3G26520,AT1G19450,AT1G10660,AT3G24760,AT1G09160,AT4G28080,AT3G13370,AT3G12710,AT3G18440,AT2G25180,AT5G24930,AT3G27090,AT5G67160,AT4G26050,AT5G66920,AT3G16960,AT1G09380,AT5G05950,AT3G19930,AT1G65640,AT3G21690,AT5G62540,AT2G17330,AT1G21130,AT1G23080,AT5G62280,AT5G01820,AT4G19170,AT5G01750,AT5G61380,AT1G61140,AT1G14370,AT1G60600,AT1G67360,AT1G03900,AT4G12390,AT1G76100,AT5G58410,AT3G60680,AT3G60350,AT1G10670,AT4G10360,AT3G09750,AT3G59930,AT3G59920,AT4G08930,AT2G22570,AT3G59020,AT3G10850,AT1G35580,AT3G57280,AT1G74940,AT2G07490,AT2G28200,AT3G56410,AT4G03190,AT2G01290,AT1G49260,AT3G54900,AT4G01280,AT4G00270
AATACTAAT	10	3	['8-12']	116	AT3G53500,AT5G49910,AT3G07650,AT2G47600,AT5G49480,AT2G33330,AT1G13280,AT4G01070,AT1G02305,AT4G17230,AT1G13180,AT2G27130,AT2G32280,AT1G01620,AT1G01500,AT1G11755,AT4G15140,AT1G14920,AT5G45830,AT1G20693,AT5G44520,AT1G18170,AT2G16860,AT3G47500,AT1G23980,AT1G68920,AT4G14270,AT1G75240,AT2G44670,AT1G32130,AT2G34580,AT2G15320,AT1G33270,AT5G37600,AT5G13120,AT3G28270,AT5G21060,AT2G42580,AT2G38230,AT2G34140,AT5G24010,AT2G02515,AT4G36640,AT1G26880,AT5G22310,AT4G34370,AT5G19240,AT2G19270,AT2G36870,AT4G33985,AT3G15220,AT3G27300,AT3G27840,AT1G55850,AT1G55910,AT3G26520,AT1G19450,AT1G10660,AT3G24760,AT1G09160,AT4G28080,AT3G13370,AT3G12710,AT3G18440,AT2G25180,AT5G24930,AT3G27090,AT5G67160,AT4G26050,AT5G66920,AT3G16960,AT1G09380,AT5G05950,AT3G19930,AT1G65640,AT3G21690,AT5G62540,AT2G17330,AT1G21130,AT1G23080,AT5G62280,AT5G01820,AT4G19170,AT5G01750,AT5G61380,AT1G61140,AT1G14370,AT1G60600,AT1G67360,AT1G03900,AT4G12390,AT1G76100,AT5G58410,AT3G60680,AT3G60350,AT1G10670,AT4G10360,AT3G09750,AT3G59930,AT3G59920,AT4G08930,AT2G22570,AT3G59020,AT3G10850,AT1G35580,AT3G57280,AT1G74940,AT2G07490,AT2G28200,AT3G56410,AT4G03190,AT2G01290,AT1G49260,AT3G54900,AT4G01280,AT4G00270
AATACTAAT	10	4	['8-12']	116	AT3G53500,AT5G49910,AT3G07650,AT2G47600,AT5G49480,AT2G33330,AT1G13280,AT4G01070,AT1G02305,AT4G17230,AT1G13180,AT2G27130,AT2G32280,AT1G01620,AT1G01500,AT1G11755,AT4G15140,AT1G14920,AT5G45830,AT1G20693,AT5G44520,AT1G18170,AT2G16860,AT3G47500,AT1G23980,AT1G68920,AT4G14270,AT1G75240,AT2G44670,AT1G32130,AT2G34580,AT2G15320,AT1G33270,AT5G37600,AT5G13120,AT3G28270,AT5G21060,AT2G42580,AT2G38230,AT2G34140,AT5G24010,AT2G02515,AT4G36640,AT1G26880,AT5G22310,AT4G34370,AT5G19240,AT2G19270,AT2G36870,AT4G33985,AT3G15220,AT3G27300,AT3G27840,AT1G55850,AT1G55910,AT3G26520,AT1G19450,AT1G10660,AT3G24760,AT1G09160,AT4G28080,AT3G13370,AT3G12710,AT3G18440,AT2G25180,AT5G24930,AT3G27090,AT5G67160,AT4G26050,AT5G66920,AT3G16960,AT1G09380,AT5G05950,AT3G19930,AT1G65640,AT3G21690,AT5G62540,AT2G17330,AT1G21130,AT1G23080,AT5G62280,AT5G01820,AT4G19170,AT5G01750,AT5G61380,AT1G61140,AT1G14370,AT1G60600,AT1G67360,AT1G03900,AT4G12390,AT1G76100,AT5G58410,AT3G60680,AT3G60350,AT1G10670,AT4G10360,AT3G09750,AT3G59930,AT3G59920,AT4G08930,AT2G22570,AT3G59020,AT3G10850,AT1G35580,AT3G57280,AT1G74940,AT2G07490,AT2G28200,AT3G56410,AT4G03190,AT2G01290,AT1G49260,AT3G54900,AT4G01280,AT4G00270

AATAGAAAA	10	1	['12-16']	282	AT3G54050,AT3G53720,AT5G04130,AT5G51040,AT4G00770,AT4G00895,AT2G05920,AT5G50180,AT2G06005,AT2G18640,AT4G00130,AT1G32060,AT2G47600,AT2G47590,AT3G05180,AT3G51620,AT2G28840,AT1G17970,AT4G25570,AT1G12990,AT5G47730,AT3G50860,AT5G47390,AT3G49250,AT4G15550,AT5G47020,AT2G14660,AT1G18990,AT3G49590,AT1G14810,AT1G29900,AT1G15710,AT2G07140,AT1G34160,AT1G07010,AT5G45780,AT1G15670,AT1G20696,AT5G45240,AT5G45300,AT1G13540,AT5G44580,AT5G44730,AT4G16410,AT1G20540,AT1G79410,AT3G08510,AT2G23840,AT5G43930,AT1G56430,AT3G47240,AT2G35590,AT1G77570,AT4G15100,AT2G16070,AT1G71020,AT3G12290,AT2G29630,AT5G42470,AT5G42720,AT1G65210,AT1G72030,AT1G72060,AT1G47900,AT1G56590,AT1G03000,AT5G41760,AT2G03110,AT1G54880,AT1G52230,AT3G06150,AT2G25930,AT5G40840,AT2G26800,AT2G44770,AT3G42970,AT1G76730,AT2G36100,AT1G32090,AT5G40390,AT2G18960,AT1G58380,AT5G39410,AT4G39100,AT5G11060,AT4G39150,AT3G31320,AT2G28720,AT5G37780,AT2G38410,AT2G41120,AT5G37540,AT1G73650,AT2G38130,AT1G73180,AT5G24520,AT5G20935,AT4G37020,AT1G04400,AT2G04840,AT3G21420,AT3G22190,AT5G23210,AT1G26920,AT1G74370,AT1G31814,AT5G23000,AT2G02570,AT3G18610,AT3G22530,AT5G22460,AT3G18930,AT3G51840,AT5G19260,AT3G21110,AT5G22300,AT4G34090,AT3G14080,AT1G58150,AT3G19100,AT2G04420,AT2G21880,AT1G69880,AT1G69870,AT1G69830,AT2G36050,AT2G33830,AT4G33140,AT2G30490,AT2G45690,AT4G32530,AT3G27270,AT3G27210,AT3G24430,AT5G15860,AT5G15230,AT5G15020,AT2G32765,AT4G31550,AT2G26690,AT2G03750,AT4G30660,AT3G24190,AT4G30690,AT5G11890,AT5G27720,AT4G29840,AT1G32360,AT5G12250,AT5G12200,AT1G54850,AT1G17665,AT5G11930,AT1G32470,AT1G69040,AT5G27140,AT3G26580,AT1G49010,AT4G27820,AT4G27780,AT5G10210,AT5G25220,AT1G12080,AT5G09870,AT1G43770,AT3G29200,AT5G67160,AT5G66880,AT5G07580,AT5G66920,AT1G26590,AT1G09390,AT3G16910,AT4G25830,AT4G25280,AT4G25050,AT3G17040,AT5G65380,AT1G65490,AT1G08980,AT5G64460,AT1G09750,AT5G64280,AT4G23820,AT1G62180,AT5G03430,AT5G63780,AT3G18215,AT1G26800,AT5G03240,AT4G21990,AT2G17880,AT4G22310,AT5G63370,AT5G63050,AT5G62540,AT5G02620,AT3G13950,AT5G02710,AT1G07700,AT5G02190,AT5G62220,AT1G23090,AT4G19120,AT3G27690,AT3G15790,AT1G60650,AT5G01990,AT1G60590,AT5G61570,AT5G61590,AT3G63310,AT3G17630,AT4G18120,AT1G24575,AT3G62860,AT1G50030,AT3G02468,AT5G59300,AT1G18470,AT5G58950,AT3G61830,AT4G12830,AT5G58870,AT4G12730,AT3G04480,AT1G76270,AT4G12590,AT3G02830,AT3G02720,AT3G04460,AT1G76100,AT3G61080,AT4G11600,AT3G60680,AT2G28390,AT2G28360,AT5G57660,AT5G57655,AT1G80840,AT3G09630,AT3G60080,AT1G65900,AT5G57070,AT3G59660,AT4G09620,AT2G18300,AT4G08930,AT5G55990,AT4G08980,AT2G16710,AT3G59052,AT3G05940,AT3G10810,AT3G04550,AT5G56020,AT2G46530,AT1G77920,AT3G57770,AT3G05670,AT5G54390,AT1G48350,AT5G54680,AT2G20170,AT2G27360,AT1G70790,AT1G27630,AT3G10460,AT5G52850,AT2G01290,AT3G09390,AT1G49500,AT3G55450,AT1G11130,AT2G35620,AT4G02080,AT2G01620,AT3G03420,AT1G21780,AT3G05000,AT4G01130,AT3G54300

AATAGAAAA	10	2	['12-16']	282	AT3G54050,AT3G53720,AT5G04130,AT5G51040,AT4G00770,AT4G00895,AT2G05920,AT5G50180,AT2G06005,AT2G18640,AT4G00130,AT1G32060,AT2G47600,AT2G47590,AT3G05180,AT3G51620,AT2G28840,AT1G17970,AT4G25570,AT1G12990,AT5G47730,AT3G50860,AT5G47390,AT3G49250,AT4G15550,AT5G47020,AT2G14660,AT1G18990,AT3G49590,AT1G14810,AT1G29900,AT1G15710,AT2G07140,AT1G34160,AT1G07010,AT5G45780,AT1G15670,AT1G20696,AT5G45240,AT5G45300,AT1G13540,AT5G44580,AT5G44730,AT4G16410,AT1G20540,AT1G79410,AT3G08510,AT2G23840,AT5G43930,AT1G56430,AT3G47240,AT2G35590,AT1G77570,AT4G15100,AT2G16070,AT1G71020,AT3G12290,AT2G29630,AT5G42470,AT5G42720,AT1G65210,AT1G72030,AT1G72060,AT1G47900,AT1G56590,AT1G03000,AT5G41760,AT2G03110,AT1G54880,AT1G52230,AT3G06150,AT2G25930,AT5G40840,AT2G26800,AT2G44770,AT3G42970,AT1G76730,AT2G36100,AT1G32090,AT5G40390,AT2G18960,AT1G58380,AT5G39410,AT4G39100,AT5G11060,AT4G39150,AT3G31320,AT2G28720,AT5G37780,AT2G38410,AT2G41120,AT5G37540,AT1G73650,AT2G38130,AT1G73180,AT5G24520,AT5G20935,AT4G37020,AT1G04400,AT2G04840,AT3G21420,AT3G22190,AT5G23210,AT1G26920,AT1G74370,AT1G31814,AT5G23000,AT2G02570,AT3G18610,AT3G22530,AT5G22460,AT3G18930,AT3G51840,AT5G19260,AT3G21110,AT5G22300,AT4G34090,AT3G14080,AT1G58150,AT3G19100,AT2G04420,AT2G21880,AT1G69880,AT1G69870,AT1G69830,AT2G36050,AT2G33830,AT4G33140,AT2G30490,AT2G45690,AT4G32530,AT3G27270,AT3G27210,AT3G24430,AT5G15860,AT5G15230,AT5G15020,AT2G32765,AT4G31550,AT2G26690,AT2G03750,AT4G30660,AT3G24190,AT4G30690,AT5G11890,AT5G27720,AT4G29840,AT1G32360,AT5G12250,AT5G12200,AT1G54850,AT1G17665,AT5G11930,AT1G32470,AT1G69040,AT5G27140,AT3G26580,AT1G49010,AT4G27820,AT4G27780,AT5G10210,AT5G25220,AT1G12080,AT5G09870,AT1G43770,AT3G29200,AT5G67160,AT5G66880,AT5G07580,AT5G66920,AT1G26590,AT1G09390,AT3G16910,AT4G25830,AT4G25280,AT4G25050,AT3G17040,AT5G65380,AT1G65490,AT1G08980,AT5G64460,AT1G09750,AT5G64280,AT4G23820,AT1G62180,AT5G03430,AT5G63780,AT3G18215,AT1G26800,AT5G03240,AT4G21990,AT2G17880,AT4G22310,AT5G63370,AT5G63050,AT5G62540,AT5G02620,AT3G13950,AT5G02710,AT1G07700,AT5G02190,AT5G62220,AT1G23090,AT4G19120,AT3G27690,AT3G15790,AT1G60650,AT5G01990,AT1G60590,AT5G61570,AT5G61590,AT3G63310,AT3G17630,AT4G18120,AT1G24575,AT3G62860,AT1G50030,AT3G02468,AT5G59300,AT1G18470,AT5G58950,AT3G61830,AT4G12830,AT5G58870,AT4G12730,AT3G04480,AT1G76270,AT4G12590,AT3G02830,AT3G02720,AT3G04460,AT1G76100,AT3G61080,AT4G11600,AT3G60680,AT2G28390,AT2G28360,AT5G57660,AT5G57655,AT1G80840,AT3G09630,AT3G60080,AT1G65900,AT5G57070,AT3G59660,AT4G09620,AT2G18300,AT4G08930,AT5G55990,AT4G08980,AT2G16710,AT3G59052,AT3G05940,AT3G10810,AT3G04550,AT5G56020,AT2G46530,AT1G77920,AT3G57770,AT3G05670,AT5G54390,AT1G48350,AT5G54680,AT2G20170,AT2G27360,AT1G70790,AT1G27630,AT3G10460,AT5G52850,AT2G01290,AT3G09390,AT1G49500,AT3G55450,AT1G11130,AT2G35620,AT4G02080,AT2G01620,AT3G03420,AT1G21780,AT3G05000,AT4G01130,AT3G54300

AATAGAAAA	10	3	['12-16']	282	AT3G54050,AT3G53720,AT5G04130,AT5G51040,AT4G00770,AT4G00895,AT2G05920,AT5G50180,AT2G06005,AT2G18640,AT4G00130,AT1G32060,AT2G47600,AT2G47590,AT3G05180,AT3G51620,AT2G28840,AT1G17970,AT4G25570,AT1G12990,AT5G47730,AT3G50860,AT5G47390,AT3G49250,AT4G15550,AT5G47020,AT2G14660,AT1G18990,AT3G49590,AT1G14810,AT1G29900,AT1G15710,AT2G07140,AT1G34160,AT1G07010,AT5G45780,AT1G15670,AT1G20696,AT5G45240,AT5G45300,AT1G13540,AT5G44580,AT5G44730,AT4G16410,AT1G20540,AT1G79410,AT3G08510,AT2G23840,AT5G43930,AT1G56430,AT3G47240,AT2G35590,AT1G77570,AT4G15100,AT2G16070,AT1G71020,AT3G12290,AT2G29630,AT5G42470,AT5G42720,AT1G65210,AT1G72030,AT1G72060,AT1G47900,AT1G56590,AT1G03000,AT5G41760,AT2G03110,AT1G54880,AT1G52230,AT3G06150,AT2G25930,AT5G40840,AT2G26800,AT2G44770,AT3G42970,AT1G76730,AT2G36100,AT1G32090,AT5G40390,AT2G18960,AT1G58380,AT5G39410,AT4G39100,AT5G11060,AT4G39150,AT3G31320,AT2G28720,AT5G37780,AT2G38410,AT2G41120,AT5G37540,AT1G73650,AT2G38130,AT1G73180,AT5G24520,AT5G20935,AT4G37020,AT1G04400,AT2G04840,AT3G21420,AT3G22190,AT5G23210,AT1G26920,AT1G74370,AT1G31814,AT5G23000,AT2G02570,AT3G18610,AT3G22530,AT5G22460,AT3G18930,AT3G51840,AT5G19260,AT3G21110,AT5G22300,AT4G34090,AT3G14080,AT1G58150,AT3G19100,AT2G04420,AT2G21880,AT1G69880,AT1G69870,AT1G69830,AT2G36050,AT2G33830,AT4G33140,AT2G30490,AT2G45690,AT4G32530,AT3G27270,AT3G27210,AT3G24430,AT5G15860,AT5G15230,AT5G15020,AT2G32765,AT4G31550,AT2G26690,AT2G03750,AT4G30660,AT3G24190,AT4G30690,AT5G11890,AT5G27720,AT4G29840,AT1G32360,AT5G12250,AT5G12200,AT1G54850,AT1G17665,AT5G11930,AT1G32470,AT1G69040,AT5G27140,AT3G26580,AT1G49010,AT4G27820,AT4G27780,AT5G10210,AT5G25220,AT1G12080,AT5G09870,AT1G43770,AT3G29200,AT5G67160,AT5G66880,AT5G07580,AT5G66920,AT1G26590,AT1G09390,AT3G16910,AT4G25830,AT4G25280,AT4G25050,AT3G17040,AT5G65380,AT1G65490,AT1G08980,AT5G64460,AT1G09750,AT5G64280,AT4G23820,AT1G62180,AT5G03430,AT5G63780,AT3G18215,AT1G26800,AT5G03240,AT4G21990,AT2G17880,AT4G22310,AT5G63370,AT5G63050,AT5G62540,AT5G02620,AT3G13950,AT5G02710,AT1G07700,AT5G02190,AT5G62220,AT1G23090,AT4G19120,AT3G27690,AT3G15790,AT1G60650,AT5G01990,AT1G60590,AT5G61570,AT5G61590,AT3G63310,AT3G17630,AT4G18120,AT1G24575,AT3G62860,AT1G50030,AT3G02468,AT5G59300,AT1G18470,AT5G58950,AT3G61830,AT4G12830,AT5G58870,AT4G12730,AT3G04480,AT1G76270,AT4G12590,AT3G02830,AT3G02720,AT3G04460,AT1G76100,AT3G61080,AT4G11600,AT3G60680,AT2G28390,AT2G28360,AT5G57660,AT5G57655,AT1G80840,AT3G09630,AT3G60080,AT1G65900,AT5G57070,AT3G59660,AT4G09620,AT2G18300,AT4G08930,AT5G55990,AT4G08980,AT2G16710,AT3G59052,AT3G05940,AT3G10810,AT3G04550,AT5G56020,AT2G46530,AT1G77920,AT3G57770,AT3G05670,AT5G54390,AT1G48350,AT5G54680,AT2G20170,AT2G27360,AT1G70790,AT1G27630,AT3G10460,AT5G52850,AT2G01290,AT3G09390,AT1G49500,AT3G55450,AT1G11130,AT2G35620,AT4G02080,AT2G01620,AT3G03420,AT1G21780,AT3G05000,AT4G01130,AT3G54300

AATAGAAAA	10	4	['12-16']	282	AT3G54050,AT3G53720,AT5G04130,AT5G51040,AT4G00770,AT4G00895,AT2G05920,AT5G50180,AT2G06005,AT2G18640,AT4G00130,AT1G32060,AT2G47600,AT2G47590,AT3G05180,AT3G51620,AT2G28840,AT1G17970,AT4G25570,AT1G12990,AT5G47730,AT3G50860,AT5G47390,AT3G49250,AT4G15550,AT5G47020,AT2G14660,AT1G18990,AT3G49590,AT1G14810,AT1G29900,AT1G15710,AT2G07140,AT1G34160,AT1G07010,AT5G45780,AT1G15670,AT1G20696,AT5G45240,AT5G45300,AT1G13540,AT5G44580,AT5G44730,AT4G16410,AT1G20540,AT1G79410,AT3G08510,AT2G23840,AT5G43930,AT1G56430,AT3G47240,AT2G35590,AT1G77570,AT4G15100,AT2G16070,AT1G71020,AT3G12290,AT2G29630,AT5G42470,AT5G42720,AT1G65210,AT1G72030,AT1G72060,AT1G47900,AT1G56590,AT1G03000,AT5G41760,AT2G03110,AT1G54880,AT1G52230,AT3G06150,AT2G25930,AT5G40840,AT2G26800,AT2G44770,AT3G42970,AT1G76730,AT2G36100,AT1G32090,AT5G40390,AT2G18960,AT1G58380,AT5G39410,AT4G39100,AT5G11060,AT4G39150,AT3G31320,AT2G28720,AT5G37780,AT2G38410,AT2G41120,AT5G37540,AT1G73650,AT2G38130,AT1G73180,AT5G24520,AT5G20935,AT4G37020,AT1G04400,AT2G04840,AT3G21420,AT3G22190,AT5G23210,AT1G26920,AT1G74370,AT1G31814,AT5G23000,AT2G02570,AT3G18610,AT3G22530,AT5G22460,AT3G18930,AT3G51840,AT5G19260,AT3G21110,AT5G22300,AT4G34090,AT3G14080,AT1G58150,AT3G19100,AT2G04420,AT2G21880,AT1G69880,AT1G69870,AT1G69830,AT2G36050,AT2G33830,AT4G33140,AT2G30490,AT2G45690,AT4G32530,AT3G27270,AT3G27210,AT3G24430,AT5G15860,AT5G15230,AT5G15020,AT2G32765,AT4G31550,AT2G26690,AT2G03750,AT4G30660,AT3G24190,AT4G30690,AT5G11890,AT5G27720,AT4G29840,AT1G32360,AT5G12250,AT5G12200,AT1G54850,AT1G17665,AT5G11930,AT1G32470,AT1G69040,AT5G27140,AT3G26580,AT1G49010,AT4G27820,AT4G27780,AT5G10210,AT5G25220,AT1G12080,AT5G09870,AT1G43770,AT3G29200,AT5G67160,AT5G66880,AT5G07580,AT5G66920,AT1G26590,AT1G09390,AT3G16910,AT4G25830,AT4G25280,AT4G25050,AT3G17040,AT5G65380,AT1G65490,AT1G08980,AT5G64460,AT1G09750,AT5G64280,AT4G23820,AT1G62180,AT5G03430,AT5G63780,AT3G18215,AT1G26800,AT5G03240,AT4G21990,AT2G17880,AT4G22310,AT5G63370,AT5G63050,AT5G62540,AT5G02620,AT3G13950,AT5G02710,AT1G07700,AT5G02190,AT5G62220,AT1G23090,AT4G19120,AT3G27690,AT3G15790,AT1G60650,AT5G01990,AT1G60590,AT5G61570,AT5G61590,AT3G63310,AT3G17630,AT4G18120,AT1G24575,AT3G62860,AT1G50030,AT3G02468,AT5G59300,AT1G18470,AT5G58950,AT3G61830,AT4G12830,AT5G58870,AT4G12730,AT3G04480,AT1G76270,AT4G12590,AT3G02830,AT3G02720,AT3G04460,AT1G76100,AT3G61080,AT4G11600,AT3G60680,AT2G28390,AT2G28360,AT5G57660,AT5G57655,AT1G80840,AT3G09630,AT3G60080,AT1G65900,AT5G57070,AT3G59660,AT4G09620,AT2G18300,AT4G08930,AT5G55990,AT4G08980,AT2G16710,AT3G59052,AT3G05940,AT3G10810,AT3G04550,AT5G56020,AT2G46530,AT1G77920,AT3G57770,AT3G05670,AT5G54390,AT1G48350,AT5G54680,AT2G20170,AT2G27360,AT1G70790,AT1G27630,AT3G10460,AT5G52850,AT2G01290,AT3G09390,AT1G49500,AT3G55450,AT1G11130,AT2G35620,AT4G02080,AT2G01620,AT3G03420,AT1G21780,AT3G05000,AT4G01130,AT3G54300

AATAGAAAA	10	5	['12-16']	282	AT3G54050,AT3G53720,AT5G04130,AT5G51040,AT4G00770,AT4G00895,AT2G05920,AT5G50180,AT2G06005,AT2G18640,AT4G00130,AT1G32060,AT2G47600,AT2G47590,AT3G05180,AT3G51620,AT2G28840,AT1G17970,AT4G25570,AT1G12990,AT5G47730,AT3G50860,AT5G47390,AT3G49250,AT4G15550,AT5G47020,AT2G14660,AT1G18990,AT3G49590,AT1G14810,AT1G29900,AT1G15710,AT2G07140,AT1G34160,AT1G07010,AT5G45780,AT1G15670,AT1G20696,AT5G45240,AT5G45300,AT1G13540,AT5G44580,AT5G44730,AT4G16410,AT1G20540,AT1G79410,AT3G08510,AT2G23840,AT5G43930,AT1G56430,AT3G47240,AT2G35590,AT1G77570,AT4G15100,AT2G16070,AT1G71020,AT3G12290,AT2G29630,AT5G42470,AT5G42720,AT1G65210,AT1G72030,AT1G72060,AT1G47900,AT1G56590,AT1G03000,AT5G41760,AT2G03110,AT1G54880,AT1G52230,AT3G06150,AT2G25930,AT5G40840,AT2G26800,AT2G44770,AT3G42970,AT1G76730,AT2G36100,AT1G32090,AT5G40390,AT2G18960,AT1G58380,AT5G39410,AT4G39100,AT5G11060,AT4G39150,AT3G31320,AT2G28720,AT5G37780,AT2G38410,AT2G41120,AT5G37540,AT1G73650,AT2G38130,AT1G73180,AT5G24520,AT5G20935,AT4G37020,AT1G04400,AT2G04840,AT3G21420,AT3G22190,AT5G23210,AT1G26920,AT1G74370,AT1G31814,AT5G23000,AT2G02570,AT3G18610,AT3G22530,AT5G22460,AT3G18930,AT3G51840,AT5G19260,AT3G21110,AT5G22300,AT4G34090,AT3G14080,AT1G58150,AT3G19100,AT2G04420,AT2G21880,AT1G69880,AT1G69870,AT1G69830,AT2G36050,AT2G33830,AT4G33140,AT2G30490,AT2G45690,AT4G32530,AT3G27270,AT3G27210,AT3G24430,AT5G15860,AT5G15230,AT5G15020,AT2G32765,AT4G31550,AT2G26690,AT2G03750,AT4G30660,AT3G24190,AT4G30690,AT5G11890,AT5G27720,AT4G29840,AT1G32360,AT5G12250,AT5G12200,AT1G54850,AT1G17665,AT5G11930,AT1G32470,AT1G69040,AT5G27140,AT3G26580,AT1G49010,AT4G27820,AT4G27780,AT5G10210,AT5G25220,AT1G12080,AT5G09870,AT1G43770,AT3G29200,AT5G67160,AT5G66880,AT5G07580,AT5G66920,AT1G26590,AT1G09390,AT3G16910,AT4G25830,AT4G25280,AT4G25050,AT3G17040,AT5G65380,AT1G65490,AT1G08980,AT5G64460,AT1G09750,AT5G64280,AT4G23820,AT1G62180,AT5G03430,AT5G63780,AT3G18215,AT1G26800,AT5G03240,AT4G21990,AT2G17880,AT4G22310,AT5G63370,AT5G63050,AT5G62540,AT5G02620,AT3G13950,AT5G02710,AT1G07700,AT5G02190,AT5G62220,AT1G23090,AT4G19120,AT3G27690,AT3G15790,AT1G60650,AT5G01990,AT1G60590,AT5G61570,AT5G61590,AT3G63310,AT3G17630,AT4G18120,AT1G24575,AT3G62860,AT1G50030,AT3G02468,AT5G59300,AT1G18470,AT5G58950,AT3G61830,AT4G12830,AT5G58870,AT4G12730,AT3G04480,AT1G76270,AT4G12590,AT3G02830,AT3G02720,AT3G04460,AT1G76100,AT3G61080,AT4G11600,AT3G60680,AT2G28390,AT2G28360,AT5G57660,AT5G57655,AT1G80840,AT3G09630,AT3G60080,AT1G65900,AT5G57070,AT3G59660,AT4G09620,AT2G18300,AT4G08930,AT5G55990,AT4G08980,AT2G16710,AT3G59052,AT3G05940,AT3G10810,AT3G04550,AT5G56020,AT2G46530,AT1G77920,AT3G57770,AT3G05670,AT5G54390,AT1G48350,AT5G54680,AT2G20170,AT2G27360,AT1G70790,AT1G27630,AT3G10460,AT5G52850,AT2G01290,AT3G09390,AT1G49500,AT3G55450,AT1G11130,AT2G35620,AT4G02080,AT2G01620,AT3G03420,AT1G21780,AT3G05000,AT4G01130,AT3G54300
AATATTTTTATT	10	1	['4-8', '8-12']	36	AT4G39960,AT1G72770,AT1G77000,AT5G26770,AT5G26790,AT3G62720,AT2G26530,AT5G26570,AT3G54500,AT3G16840,AT5G21060,AT5G24910,AT1G44446,AT2G04795,AT4G16570,AT5G46910,AT5G56870,AT3G48690,AT3G48140,AT4G24340,AT5G64800,AT3G16220,AT4G15730,AT5G17170,AT3G12350,AT5G42810,AT3G23530,AT1G72060,AT4G02370,AT2G46820,AT3G44950,AT3G03380,AT3G44720,AT1G17145,AT1G30270,AT5G27920
AATATTTTTATT	10	2	['4-8', '8-12']	36	AT4G39960,AT1G72770,AT1G77000,AT5G26770,AT5G26790,AT3G62720,AT2G26530,AT5G26570,AT3G54500,AT3G16840,AT5G21060,AT5G24910,AT1G44446,AT2G04795,AT4G16570,AT5G46910,AT5G56870,AT3G48690,AT3G48140,AT4G24340,AT5G64800,AT3G16220,AT4G15730,AT5G17170,AT3G12350,AT5G42810,AT3G23530,AT1G72060,AT4G02370,AT2G46820,AT3G44950,AT3G03380,AT3G44720,AT1G17145,AT1G30270,AT5G27920
AATATTTTTATT	10	3	['4-8', '8-12']	36	AT4G39960,AT1G72770,AT1G77000,AT5G26770,AT5G26790,AT3G62720,AT2G26530,AT5G26570,AT3G54500,AT3G16840,AT5G21060,AT5G24910,AT1G44446,AT2G04795,AT4G16570,AT5G46910,AT5G56870,AT3G48690,AT3G48140,AT4G24340,AT5G64800,AT3G16220,AT4G15730,AT5G17170,AT3G12350,AT5G42810,AT3G23530,AT1G72060,AT4G02370,AT2G46820,AT3G44950,AT3G03380,AT3G44720,AT1G17145,AT1G30270,AT5G27920
AATATTTTTATT	10	4	['4-8', '8-12']	36	AT4G39960,AT1G72770,AT1G77000,AT5G26770,AT5G26790,AT3G62720,AT2G26530,AT5G26570,AT3G54500,AT3G16840,AT5G21060,AT5G24910,AT1G44446,AT2G04795,AT4G16570,AT5G46910,AT5G56870,AT3G48690,AT3G48140,AT4G24340,AT5G64800,AT3G16220,AT4G15730,AT5G17170,AT3G12350,AT5G42810,AT3G23530,AT1G72060,AT4G02370,AT2G46820,AT3G44950,AT3G03380,AT3G44720,AT1G17145,AT1G30270,AT5G27920

AATATTTTTATT	10	5	['4-8', '8-12']	36	AT4G39960,AT1G72770,AT1G77000,AT5G26770,AT5G26790,AT3G62720,AT2G26530,AT5G26570,AT3G54500,AT3G16840,AT5G21060,AT5G24910,AT1G44446,AT2G04795,AT4G16570,AT5G46910,AT5G56870,AT3G48690,AT3G48140,AT4G24340,AT5G64800,AT3G16220,AT4G15730,AT5G17170,AT3G12350,AT5G42810,AT3G23530,AT1G72060,AT4G02370,AT2G46820,AT3G44950,AT3G03380,AT3G44720,AT1G17145,AT1G30270,AT5G27920
AATGGAAATG	10	1	['16-20', '20-24']	14	AT3G63310,AT1G78180,AT1G79500,AT3G01790,AT5G55070,AT4G27820,AT3G15630,AT1G55490,AT1G06460,AT1G78995,AT1G50940,AT5G25070,AT3G14270,AT4G14930
AATGGAAATG	10	2	['16-20', '20-24']	14	AT3G63310,AT1G78180,AT1G79500,AT3G01790,AT5G55070,AT4G27820,AT3G15630,AT1G55490,AT1G06460,AT1G78995,AT1G50940,AT5G25070,AT3G14270,AT4G14930
AATGGAAATG	10	3	['16-20', '20-24']	14	AT3G63310,AT1G78180,AT1G79500,AT3G01790,AT5G55070,AT4G27820,AT3G15630,AT1G55490,AT1G06460,AT1G78995,AT1G50940,AT5G25070,AT3G14270,AT4G14930
AATGGAAATG	10	4	['16-20', '20-24']	14	AT3G63310,AT1G78180,AT1G79500,AT3G01790,AT5G55070,AT4G27820,AT3G15630,AT1G55490,AT1G06460,AT1G78995,AT1G50940,AT5G25070,AT3G14270,AT4G14930
AATGGAAATG	10	5	['16-20', '20-24']	14	AT3G63310,AT1G78180,AT1G79500,AT3G01790,AT5G55070,AT4G27820,AT3G15630,AT1G55490,AT1G06460,AT1G78995,AT1G50940,AT5G25070,AT3G14270,AT4G14930
AAWGTATCSA	10	1	['8-12', '20-24']	32	AT3G15430,AT1G27090,AT2G40780,AT3G11620,AT5G59180,AT2G41090,AT1G06820,AT4G12390,AT3G01120,AT3G05130,AT1G76140,AT4G25530,AT1G67120,AT1G26560,AT1G09340,AT1G26880,AT3G48530,AT1G33780,AT3G28715,AT4G17760,AT1G21250,AT3G07080,AT3G58120,AT3G26080,AT1G78010,AT2G05520,AT1G56290,AT1G78510,AT5G62980,AT4G31550,AT5G15160,AT2G43710
AAWGTATCSA	10	2	['8-12', '20-24']	32	AT3G15430,AT1G27090,AT2G40780,AT3G11620,AT5G59180,AT2G41090,AT1G06820,AT4G12390,AT3G01120,AT3G05130,AT1G76140,AT4G25530,AT1G67120,AT1G26560,AT1G09340,AT1G26880,AT3G48530,AT1G33780,AT3G28715,AT4G17760,AT1G21250,AT3G07080,AT3G58120,AT3G26080,AT1G78010,AT2G05520,AT1G56290,AT1G78510,AT5G62980,AT4G31550,AT5G15160,AT2G43710
AAWGTATCSA	10	3	['8-12', '20-24']	32	AT3G15430,AT1G27090,AT2G40780,AT3G11620,AT5G59180,AT2G41090,AT1G06820,AT4G12390,AT3G01120,AT3G05130,AT1G76140,AT4G25530,AT1G67120,AT1G26560,AT1G09340,AT1G26880,AT3G48530,AT1G33780,AT3G28715,AT4G17760,AT1G21250,AT3G07080,AT3G58120,AT3G26080,AT1G78010,AT2G05520,AT1G56290,AT1G78510,AT5G62980,AT4G31550,AT5G15160,AT2G43710
AAWGTATCSA	10	4	['8-12', '20-24']	32	AT3G15430,AT1G27090,AT2G40780,AT3G11620,AT5G59180,AT2G41090,AT1G06820,AT4G12390,AT3G01120,AT3G05130,AT1G76140,AT4G25530,AT1G67120,AT1G26560,AT1G09340,AT1G26880,AT3G48530,AT1G33780,AT3G28715,AT4G17760,AT1G21250,AT3G07080,AT3G58120,AT3G26080,AT1G78010,AT2G05520,AT1G56290,AT1G78510,AT5G62980,AT4G31550,AT5G15160,AT2G43710
AAWGTATCSA	10	5	['8-12', '20-24']	32	AT3G15430,AT1G27090,AT2G40780,AT3G11620,AT5G59180,AT2G41090,AT1G06820,AT4G12390,AT3G01120,AT3G05130,AT1G76140,AT4G25530,AT1G67120,AT1G26560,AT1G09340,AT1G26880,AT3G48530,AT1G33780,AT3G28715,AT4G17760,AT1G21250,AT3G07080,AT3G58120,AT3G26080,AT1G78010,AT2G05520,AT1G56290,AT1G78510,AT5G62980,AT4G31550,AT5G15160,AT2G43710

ACACAGG	10	1	['8-12', '12-16']	162	AT4G00340,AT1G15400,AT2G26500,AT3G01660,AT3G52960,AT1G06470,AT3G53030,AT3G52150,AT1G32060,AT2G38000,AT3G01180,AT2G45200,AT1G19920,AT3G03830,AT5G48330,AT1G75800,AT1G75840,AT1G69160,AT4G25570,AT1G02305,AT1G13880,AT5G45830,AT2G47850,AT1G65720,AT3G47430,AT1G36830,AT1G54270,AT2G25730,AT1G77740,AT1G68920,AT3G12350,AT3G12600,AT5G42810,AT4G14440,AT1G29395,AT1G64330,AT3G44720,AT1G73390,AT3G44250,AT1G30210,AT2G44670,AT5G40500,AT5G40160,AT2G10370,AT1G71450,AT5G28280,AT2G20480,AT2G40150,AT4G39120,AT3G31320,AT5G19860,AT2G34250,AT3G28270,AT2G28670,AT4G38800,AT3G12030,AT3G32280,AT5G37140,AT5G36790,AT2G32560,AT5G19290,AT5G20280,AT5G35540,AT5G20320,AT2G24765,AT4G37830,AT3G27170,AT2G25250,AT1G79910,AT2G25310,AT4G36790,AT5G23000,AT4G34490,AT4G36540,AT3G51840,AT2G46340,AT2G44360,AT2G33740,AT4G32940,AT1G69730,AT2G32720,AT4G31040,AT5G14660,AT2G03750,AT4G30500,AT4G29670,AT5G11890,AT4G29700,AT1G02160,AT2G24880,AT5G11500,AT1G09160,AT5G26230,AT5G10210,AT4G27330,AT1G11850,AT3G29200,AT5G07960,AT1G53540,AT1G53450,AT1G01240,AT1G09415,AT1G10090,AT5G06460,AT3G19810,AT3G21750,AT5G65430,AT1G04530,AT1G19660,AT5G04810,AT5G04790,AT3G16200,AT1G09870,AT5G63940,AT1G62180,AT1G20190,AT5G64040,AT1G61390,AT3G14520,AT4G22300,AT5G63380,AT5G63410,AT2G17900,AT4G21210,AT2G17340,AT2G17450,AT1G18740,AT4G19340,AT3G16110,AT5G01990,AT1G71695,AT3G16040,AT5G61580,AT4G18240,AT4G17880,AT5G60210,AT3G61780,AT1G31050,AT3G02830,AT5G58070,AT1G76240,AT4G11175,AT2G43010,AT4G10840,AT3G08640,AT3G10912,AT5G56230,AT3G58230,AT3G07100,AT5G54950,AT3G10050,AT5G54390,AT3G06380,AT3G10460,AT2G24490,AT4G03080,AT5G53170,AT3G09390,AT5G51460,AT4G01610,AT2G25710,AT5G59920
ACACAGG	10	2	['8-12', '12-16']	162	AT4G00340,AT1G15400,AT2G26500,AT3G01660,AT3G52960,AT1G06470,AT3G53030,AT3G52150,AT1G32060,AT2G38000,AT3G01180,AT2G45200,AT1G19920,AT3G03830,AT5G48330,AT1G75800,AT1G75840,AT1G69160,AT4G25570,AT1G02305,AT1G13880,AT5G45830,AT2G47850,AT1G65720,AT3G47430,AT1G36830,AT1G54270,AT2G25730,AT1G77740,AT1G68920,AT3G12350,AT3G12600,AT5G42810,AT4G14440,AT1G29395,AT1G64330,AT3G44720,AT1G73390,AT3G44250,AT1G30210,AT2G44670,AT5G40500,AT5G40160,AT2G10370,AT1G71450,AT5G28280,AT2G20480,AT2G40150,AT4G39120,AT3G31320,AT5G19860,AT2G34250,AT3G28270,AT2G28670,AT4G38800,AT3G12030,AT3G32280,AT5G37140,AT5G36790,AT2G32560,AT5G19290,AT5G20280,AT5G35540,AT5G20320,AT2G24765,AT4G37830,AT3G27170,AT2G25250,AT1G79910,AT2G25310,AT4G36790,AT5G23000,AT4G34490,AT4G36540,AT3G51840,AT2G46340,AT2G44360,AT2G33740,AT4G32940,AT1G69730,AT2G32720,AT4G31040,AT5G14660,AT2G03750,AT4G30500,AT4G29670,AT5G11890,AT4G29700,AT1G02160,AT2G24880,AT5G11500,AT1G09160,AT5G26230,AT5G10210,AT4G27330,AT1G11850,AT3G29200,AT5G07960,AT1G53540,AT1G53450,AT1G01240,AT1G09415,AT1G10090,AT5G06460,AT3G19810,AT3G21750,AT5G65430,AT1G04530,AT1G19660,AT5G04810,AT5G04790,AT3G16200,AT1G09870,AT5G63940,AT1G62180,AT1G20190,AT5G64040,AT1G61390,AT3G14520,AT4G22300,AT5G63380,AT5G63410,AT2G17900,AT4G21210,AT2G17340,AT2G17450,AT1G18740,AT4G19340,AT3G16110,AT5G01990,AT1G71695,AT3G16040,AT5G61580,AT4G18240,AT4G17880,AT5G60210,AT3G61780,AT1G31050,AT3G02830,AT5G58070,AT1G76240,AT4G11175,AT2G43010,AT4G10840,AT3G08640,AT3G10912,AT5G56230,AT3G58230,AT3G07100,AT5G54950,AT3G10050,AT5G54390,AT3G06380,AT3G10460,AT2G24490,AT4G03080,AT5G53170,AT3G09390,AT5G51460,AT4G01610,AT2G25710,AT5G59920

ACACAGG	10	3	['8-12', '12-16']	162	AT4G00340,AT1G15400,AT2G26500,AT3G01660,AT3G52960,AT1G06470,AT3G53030,AT3G52150,AT1G32060,AT2G38000,AT3G01180,AT2G45200,AT1G19920,AT3G03830,AT5G48330,AT1G75800,AT1G75840,AT1G69160,AT4G25570,AT1G02305,AT1G13880,AT5G45830,AT2G47850,AT1G65720,AT3G47430,AT1G36830,AT1G54270,AT2G25730,AT1G77740,AT1G68920,AT3G12350,AT3G12600,AT5G42810,AT4G14440,AT1G29395,AT1G64330,AT3G44720,AT1G73390,AT3G44250,AT1G30210,AT2G44670,AT5G40500,AT5G40160,AT2G10370,AT1G71450,AT5G28280,AT2G20480,AT2G40150,AT4G39120,AT3G31320,AT5G19860,AT2G34250,AT3G28270,AT2G28670,AT4G38800,AT3G12030,AT3G32280,AT5G37140,AT5G36790,AT2G32560,AT5G19290,AT5G20280,AT5G35540,AT5G20320,AT2G24765,AT4G37830,AT3G27170,AT2G25250,AT1G79910,AT2G25310,AT4G36790,AT5G23000,AT4G34490,AT4G36540,AT3G51840,AT2G46340,AT2G44360,AT2G33740,AT4G32940,AT1G69730,AT2G32720,AT4G31040,AT5G14660,AT2G03750,AT4G30500,AT4G29670,AT5G11890,AT4G29700,AT1G02160,AT2G24880,AT5G11500,AT1G09160,AT5G26230,AT5G10210,AT4G27330,AT1G11850,AT3G29200,AT5G07960,AT1G53540,AT1G53450,AT1G01240,AT1G09415,AT1G10090,AT5G06460,AT3G19810,AT3G21750,AT5G65430,AT1G04530,AT1G19660,AT5G04810,AT5G04790,AT3G16200,AT1G09870,AT5G63940,AT1G62180,AT1G20190,AT5G64040,AT1G61390,AT3G14520,AT4G22300,AT5G63380,AT5G63410,AT2G17900,AT4G21210,AT2G17340,AT2G17450,AT1G18740,AT4G19340,AT3G16110,AT5G01990,AT1G71695,AT3G16040,AT5G61580,AT4G18240,AT4G17880,AT5G60210,AT3G61780,AT1G31050,AT3G02830,AT5G58070,AT1G76240,AT4G11175,AT2G43010,AT4G10840,AT3G08640,AT3G10912,AT5G56230,AT3G58230,AT3G07100,AT5G54950,AT3G10050,AT5G54390,AT3G06380,AT3G10460,AT2G24490,AT4G03080,AT5G53170,AT3G09390,AT5G51460,AT4G01610,AT2G25710,AT5G59920
ACACAGG	10	4	['8-12', '12-16']	162	AT4G00340,AT1G15400,AT2G26500,AT3G01660,AT3G52960,AT1G06470,AT3G53030,AT3G52150,AT1G32060,AT2G38000,AT3G01180,AT2G45200,AT1G19920,AT3G03830,AT5G48330,AT1G75800,AT1G75840,AT1G69160,AT4G25570,AT1G02305,AT1G13880,AT5G45830,AT2G47850,AT1G65720,AT3G47430,AT1G36830,AT1G54270,AT2G25730,AT1G77740,AT1G68920,AT3G12350,AT3G12600,AT5G42810,AT4G14440,AT1G29395,AT1G64330,AT3G44720,AT1G73390,AT3G44250,AT1G30210,AT2G44670,AT5G40500,AT5G40160,AT2G10370,AT1G71450,AT5G28280,AT2G20480,AT2G40150,AT4G39120,AT3G31320,AT5G19860,AT2G34250,AT3G28270,AT2G28670,AT4G38800,AT3G12030,AT3G32280,AT5G37140,AT5G36790,AT2G32560,AT5G19290,AT5G20280,AT5G35540,AT5G20320,AT2G24765,AT4G37830,AT3G27170,AT2G25250,AT1G79910,AT2G25310,AT4G36790,AT5G23000,AT4G34490,AT4G36540,AT3G51840,AT2G46340,AT2G44360,AT2G33740,AT4G32940,AT1G69730,AT2G32720,AT4G31040,AT5G14660,AT2G03750,AT4G30500,AT4G29670,AT5G11890,AT4G29700,AT1G02160,AT2G24880,AT5G11500,AT1G09160,AT5G26230,AT5G10210,AT4G27330,AT1G11850,AT3G29200,AT5G07960,AT1G53540,AT1G53450,AT1G01240,AT1G09415,AT1G10090,AT5G06460,AT3G19810,AT3G21750,AT5G65430,AT1G04530,AT1G19660,AT5G04810,AT5G04790,AT3G16200,AT1G09870,AT5G63940,AT1G62180,AT1G20190,AT5G64040,AT1G61390,AT3G14520,AT4G22300,AT5G63380,AT5G63410,AT2G17900,AT4G21210,AT2G17340,AT2G17450,AT1G18740,AT4G19340,AT3G16110,AT5G01990,AT1G71695,AT3G16040,AT5G61580,AT4G18240,AT4G17880,AT5G60210,AT3G61780,AT1G31050,AT3G02830,AT5G58070,AT1G76240,AT4G11175,AT2G43010,AT4G10840,AT3G08640,AT3G10912,AT5G56230,AT3G58230,AT3G07100,AT5G54950,AT3G10050,AT5G54390,AT3G06380,AT3G10460,AT2G24490,AT4G03080,AT5G53170,AT3G09390,AT5G51460,AT4G01610,AT2G25710,AT5G59920

ACACAGG	10	5	['8-12', '12-16']	162	AT4G00340,AT1G15400,AT2G26500,AT3G01660,AT3G52960,AT1G06470,AT3G53030,AT3G52150,AT1G32060,AT2G38000,AT3G01180,AT2G45200,AT1G19920,AT3G03830,AT5G48330,AT1G75800,AT1G75840,AT1G69160,AT4G25570,AT1G02305,AT1G13880,AT5G45830,AT2G47850,AT1G65720,AT3G47430,AT1G36830,AT1G54270,AT2G25730,AT1G77740,AT1G68920,AT3G12350,AT3G12600,AT5G42810,AT4G14440,AT1G29395,AT1G64330,AT3G44720,AT1G73390,AT3G44250,AT1G30210,AT2G44670,AT5G40500,AT5G40160,AT2G10370,AT1G71450,AT5G28280,AT2G20480,AT2G40150,AT4G39120,AT3G31320,AT5G19860,AT2G34250,AT3G28270,AT2G28670,AT4G38800,AT3G12030,AT3G32280,AT5G37140,AT5G36790,AT2G32560,AT5G19290,AT5G20280,AT5G35540,AT5G20320,AT2G24765,AT4G37830,AT3G27170,AT2G25250,AT1G79910,AT2G25310,AT4G36790,AT5G23000,AT4G34490,AT4G36540,AT3G51840,AT2G46340,AT2G44360,AT2G33740,AT4G32940,AT1G69730,AT2G32720,AT4G31040,AT5G14660,AT2G03750,AT4G30500,AT4G29670,AT5G11890,AT4G29700,AT1G02160,AT2G24880,AT5G11500,AT1G09160,AT5G26230,AT5G10210,AT4G27330,AT1G11850,AT3G29200,AT5G07960,AT1G53540,AT1G53450,AT1G01240,AT1G09415,AT1G10090,AT5G06460,AT3G19810,AT3G21750,AT5G65430,AT1G04530,AT1G19660,AT5G04810,AT5G04790,AT3G16200,AT1G09870,AT5G63940,AT1G62180,AT1G20190,AT5G64040,AT1G61390,AT3G14520,AT4G22300,AT5G63380,AT5G63410,AT2G17900,AT4G21210,AT2G17340,AT2G17450,AT1G18740,AT4G19340,AT3G16110,AT5G01990,AT1G71695,AT3G16040,AT5G61580,AT4G18240,AT4G17880,AT5G60210,AT3G61780,AT1G31050,AT3G02830,AT5G58070,AT1G76240,AT4G11175,AT2G43010,AT4G10840,AT3G08640,AT3G10912,AT5G56230,AT3G58230,AT3G07100,AT5G54950,AT3G10050,AT5G54390,AT3G06380,AT3G10460,AT2G24490,AT4G03080,AT5G53170,AT3G09390,AT5G51460,AT4G01610,AT2G25710,AT5G59920
ACACCCG	10	1	['16-20']	86	ATMG01040,AT3G54120,AT2G37450,AT1G15100,AT3G53240,AT2G47610,AT5G48490,AT3G51140,AT5G48370,AT5G47020,AT1G07260,AT2G47780,AT4G17760,AT1G79470,AT1G20440,AT1G10510,AT1G72170,AT4G39710,AT3G18850,AT2G31750,AT5G21060,AT4G38160,AT2G30860,AT5G24620,AT4G35850,AT2G04670,AT5G22750,AT1G31910,AT4G33670,AT2G33740,AT5G18340,AT3G30810,AT5G16910,AT2G30600,AT3G23530,AT5G16290,AT2G47400,AT1G79370,ATMG01330,AT5G11970,AT4G29840,AT1G04050,AT2G24880,AT1G54830,AT1G16150,AT1G32470,AT1G22740,AT5G26240,AT1G21880,AT5G08580,AT4G26520,AT3G26710,AT4G26130,AT1G27430,AT3G16940,AT5G66410,AT5G66530,AT5G65990,AT3G26060,AT3G18080,AT5G03350,AT3G17790,AT5G63370,AT3G21690,AT3G14010,AT5G61440,AT1G24450,AT1G55490,AT3G06500,AT3G02720,AT3G09820,AT2G28370,AT3G60130,AT1G80840,AT5G57110,AT1G80730,AT5G56240,AT1G80090,AT5G54950,AT3G57770,AT3G05720,AT5G53500,AT5G52850,AT3G01850,AT4G01690,AT4G00830
ACACCCG	10	2	['16-20']	86	ATMG01040,AT3G54120,AT2G37450,AT1G15100,AT3G53240,AT2G47610,AT5G48490,AT3G51140,AT5G48370,AT5G47020,AT1G07260,AT2G47780,AT4G17760,AT1G79470,AT1G20440,AT1G10510,AT1G72170,AT4G39710,AT3G18850,AT2G31750,AT5G21060,AT4G38160,AT2G30860,AT5G24620,AT4G35850,AT2G04670,AT5G22750,AT1G31910,AT4G33670,AT2G33740,AT5G18340,AT3G30810,AT5G16910,AT2G30600,AT3G23530,AT5G16290,AT2G47400,AT1G79370,ATMG01330,AT5G11970,AT4G29840,AT1G04050,AT2G24880,AT1G54830,AT1G16150,AT1G32470,AT1G22740,AT5G26240,AT1G21880,AT5G08580,AT4G26520,AT3G26710,AT4G26130,AT1G27430,AT3G16940,AT5G66410,AT5G66530,AT5G65990,AT3G26060,AT3G18080,AT5G03350,AT3G17790,AT5G63370,AT3G21690,AT3G14010,AT5G61440,AT1G24450,AT1G55490,AT3G06500,AT3G02720,AT3G09820,AT2G28370,AT3G60130,AT1G80840,AT5G57110,AT1G80730,AT5G56240,AT1G80090,AT5G54950,AT3G57770,AT3G05720,AT5G53500,AT5G52850,AT3G01850,AT4G01690,AT4G00830
ACACCCG	10	3	['16-20']	86	ATMG01040,AT3G54120,AT2G37450,AT1G15100,AT3G53240,AT2G47610,AT5G48490,AT3G51140,AT5G48370,AT5G47020,AT1G07260,AT2G47780,AT4G17760,AT1G79470,AT1G20440,AT1G10510,AT1G72170,AT4G39710,AT3G18850,AT2G31750,AT5G21060,AT4G38160,AT2G30860,AT5G24620,AT4G35850,AT2G04670,AT5G22750,AT1G31910,AT4G33670,AT2G33740,AT5G18340,AT3G30810,AT5G16910,AT2G30600,AT3G23530,AT5G16290,AT2G47400,AT1G79370,ATMG01330,AT5G11970,AT4G29840,AT1G04050,AT2G24880,AT1G54830,AT1G16150,AT1G32470,AT1G22740,AT5G26240,AT1G21880,AT5G08580,AT4G26520,AT3G26710,AT4G26130,AT1G27430,AT3G16940,AT5G66410,AT5G66530,AT5G65990,AT3G26060,AT3G18080,AT5G03350,AT3G17790,AT5G63370,AT3G21690,AT3G14010,AT5G61440,AT1G24450,AT1G55490,AT3G06500,AT3G02720,AT3G09820,AT2G28370,AT3G60130,AT1G80840,AT5G57110,AT1G80730,AT5G56240,AT1G80090,AT5G54950,AT3G57770,AT3G05720,AT5G53500,AT5G52850,AT3G01850,AT4G01690,AT4G00830

ACACCCG	10	4	['16-20']	86	ATMG01040,AT3G54120,AT2G37450,AT1G15100,AT3G53240,AT2G47610,AT5G48490,AT3G51140,AT5G48370,AT5G47020,AT1G07260,AT2G47780,AT4G17760,AT1G79470,AT1G20440,AT1G10510,AT1G72170,AT4G39710,AT3G18850,AT2G31750,AT5G21060,AT4G38160,AT2G30860,AT5G24620,AT4G35850,AT2G04670,AT5G22750,AT1G31910,AT4G33670,AT2G33740,AT5G18340,AT3G30810,AT5G16910,AT2G30600,AT3G23530,AT5G16290,AT2G47400,AT1G79370,ATMG01330,AT5G11970,AT4G29840,AT1G04050,AT2G24880,AT1G54830,AT1G16150,AT1G32470,AT1G22740,AT5G26240,AT1G21880,AT5G08580,AT4G26520,AT3G26710,AT4G26130,AT1G27430,AT3G16940,AT5G66410,AT5G66530,AT5G65990,AT3G26060,AT3G18080,AT5G03350,AT3G17790,AT5G63370,AT3G21690,AT3G14010,AT5G61440,AT1G24450,AT1G55490,AT3G06500,AT3G02720,AT3G09820,AT2G28370,AT3G60130,AT1G80840,AT5G57110,AT1G80730,AT5G56240,AT1G80090,AT5G54950,AT3G57770,AT3G05720,AT5G53500,AT5G52850,AT3G01850,AT4G01690,AT4G00830
ACACCCG	10	5	['16-20']	86	ATMG01040,AT3G54120,AT2G37450,AT1G15100,AT3G53240,AT2G47610,AT5G48490,AT3G51140,AT5G48370,AT5G47020,AT1G07260,AT2G47780,AT4G17760,AT1G79470,AT1G20440,AT1G10510,AT1G72170,AT4G39710,AT3G18850,AT2G31750,AT5G21060,AT4G38160,AT2G30860,AT5G24620,AT4G35850,AT2G04670,AT5G22750,AT1G31910,AT4G33670,AT2G33740,AT5G18340,AT3G30810,AT5G16910,AT2G30600,AT3G23530,AT5G16290,AT2G47400,AT1G79370,ATMG01330,AT5G11970,AT4G29840,AT1G04050,AT2G24880,AT1G54830,AT1G16150,AT1G32470,AT1G22740,AT5G26240,AT1G21880,AT5G08580,AT4G26520,AT3G26710,AT4G26130,AT1G27430,AT3G16940,AT5G66410,AT5G66530,AT5G65990,AT3G26060,AT3G18080,AT5G03350,AT3G17790,AT5G63370,AT3G21690,AT3G14010,AT5G61440,AT1G24450,AT1G55490,AT3G06500,AT3G02720,AT3G09820,AT2G28370,AT3G60130,AT1G80840,AT5G57110,AT1G80730,AT5G56240,AT1G80090,AT5G54950,AT3G57770,AT3G05720,AT5G53500,AT5G52850,AT3G01850,AT4G01690,AT4G00830
ACACCGG	10	1	['0-4', '12-16', '20-24', '4-8']	97	AT2G06005,AT1G13930,AT3G01660,AT4G00400,AT2G40840,AT2G18790,AT2G45190,AT2G40610,AT3G50610,AT1G01470,AT1G18990,AT5G46760,AT4G16146,AT1G18180,AT4G17650,AT4G15810,AT2G16070,AT3G06160,AT2G44670,AT1G37537,AT1G31500,AT1G75140,AT2G39480,AT2G21240,AT5G19540,AT5G08330,AT2G42590,AT5G20280,AT2G38130,AT2G24820,AT4G36210,AT2G25210,AT1G26940,AT5G23240,AT3G18610,AT5G23050,AT1G26880,AT3G26380,AT2G21260,AT2G30150,AT1G63800,AT4G33700,AT2G42670,AT2G47410,AT4G31340,AT5G14760,AT3G24170,AT2G43750,AT2G43790,AT1G53210,AT4G29750,AT1G02140,AT5G27380,AT5G27010,AT1G32520,AT4G28210,AT3G20390,AT2G25080,AT1G53640,AT5G07400,AT5G06720,AT1G26590,AT5G65110,AT1G04530,AT4G24350,AT2G04390,AT1G05850,AT5G03190,AT3G14000,AT4G19710,AT1G07890,AT5G62090,AT5G62200,AT3G23325,AT3G15430,AT1G01940,AT3G02690,AT3G06510,AT4G13250,AT3G61830,AT2G36800,AT1G15520,AT4G11600,AT3G02930,AT3G08580,AT3G09750,AT5G57030,AT3G59770,AT3G59020,AT3G10810,AT2G07490,AT3G56260,AT3G56290,AT4G02420,AT4G02260,AT4G01940,AT1G17050
ACACCGG	10	2	['0-4', '4-8', '12-16', '20-24']	97	AT2G06005,AT1G13930,AT3G01660,AT4G00400,AT2G40840,AT2G18790,AT2G45190,AT2G40610,AT3G50610,AT1G01470,AT1G18990,AT5G46760,AT4G16146,AT1G18180,AT4G17650,AT4G15810,AT2G16070,AT3G06160,AT2G44670,AT1G37537,AT1G31500,AT1G75140,AT2G39480,AT2G21240,AT5G19540,AT5G08330,AT2G42590,AT5G20280,AT2G38130,AT2G24820,AT4G36210,AT2G25210,AT1G26940,AT5G23240,AT3G18610,AT5G23050,AT1G26880,AT3G26380,AT2G21260,AT2G30150,AT1G63800,AT4G33700,AT2G42670,AT2G47410,AT4G31340,AT5G14760,AT3G24170,AT2G43750,AT2G43790,AT1G53210,AT4G29750,AT1G02140,AT5G27380,AT5G27010,AT1G32520,AT4G28210,AT3G20390,AT2G25080,AT1G53640,AT5G07400,AT5G06720,AT1G26590,AT5G65110,AT1G04530,AT4G24350,AT2G04390,AT1G05850,AT5G03190,AT3G14000,AT4G19710,AT1G07890,AT5G62090,AT5G62200,AT3G23325,AT3G15430,AT1G01940,AT3G02690,AT3G06510,AT4G13250,AT3G61830,AT2G36800,AT1G15520,AT4G11600,AT3G02930,AT3G08580,AT3G09750,AT5G57030,AT3G59770,AT3G59020,AT3G10810,AT2G07490,AT3G56260,AT3G56290,AT4G02420,AT4G02260,AT4G01940,AT1G17050

ACACCGG	10	3	['0-4', '12-16', '20-24', '4-8']	97	AT2G06005,AT1G13930,AT3G01660,AT4G00400,AT2G40840,AT2G18790,AT2G45190,AT2G40610,AT3G50610,AT1G01470,AT1G18990,AT5G46760,AT4G16146,AT1G18180,AT4G17650,AT4G15810,AT2G16070,AT3G06160,AT2G44670,AT1G37537,AT1G31500,AT1G75140,AT2G39480,AT2G21240,AT5G19540,AT5G08330,AT2G42590,AT5G20280,AT2G38130,AT2G24820,AT4G36210,AT2G25210,AT1G26940,AT5G23240,AT3G18610,AT5G23050,AT1G26880,AT3G26380,AT2G21260,AT2G30150,AT1G63800,AT4G33700,AT2G42670,AT2G47410,AT4G31340,AT5G14760,AT3G24170,AT2G43750,AT2G43790,AT1G53210,AT4G29750,AT1G02140,AT5G27380,AT5G27010,AT1G32520,AT4G28210,AT3G20390,AT2G25080,AT1G53640,AT5G07400,AT5G06720,AT1G26590,AT5G65110,AT1G04530,AT4G24350,AT2G04390,AT1G05850,AT5G03190,AT3G14000,AT4G19710,AT1G07890,AT5G62090,AT5G62200,AT3G23325,AT3G15430,AT1G01940,AT3G02690,AT3G06510,AT4G13250,AT3G61830,AT2G36800,AT1G15520,AT4G11600,AT3G02930,AT3G08580,AT3G09750,AT5G57030,AT3G59770,AT3G59020,AT3G10810,AT2G07490,AT3G56260,AT3G56290,AT4G02420,AT4G02260,AT4G01940,AT1G17050
ACACCGG	10	4	['0-4', '12-16', '20-24']	97	AT2G06005,AT1G13930,AT3G01660,AT4G00400,AT2G40840,AT2G18790,AT2G45190,AT2G40610,AT3G50610,AT1G01470,AT1G18990,AT5G46760,AT4G16146,AT1G18180,AT4G17650,AT4G15810,AT2G16070,AT3G06160,AT2G44670,AT1G37537,AT1G31500,AT1G75140,AT2G39480,AT2G21240,AT5G19540,AT5G08330,AT2G42590,AT5G20280,AT2G38130,AT2G24820,AT4G36210,AT2G25210,AT1G26940,AT5G23240,AT3G18610,AT5G23050,AT1G26880,AT3G26380,AT2G21260,AT2G30150,AT1G63800,AT4G33700,AT2G42670,AT2G47410,AT4G31340,AT5G14760,AT3G24170,AT2G43750,AT2G43790,AT1G53210,AT4G29750,AT1G02140,AT5G27380,AT5G27010,AT1G32520,AT4G28210,AT3G20390,AT2G25080,AT1G53640,AT5G07400,AT5G06720,AT1G26590,AT5G65110,AT1G04530,AT4G24350,AT2G04390,AT1G05850,AT5G03190,AT3G14000,AT4G19710,AT1G07890,AT5G62090,AT5G62200,AT3G23325,AT3G15430,AT1G01940,AT3G02690,AT3G06510,AT4G13250,AT3G61830,AT2G36800,AT1G15520,AT4G11600,AT3G02930,AT3G08580,AT3G09750,AT5G57030,AT3G59770,AT3G59020,AT3G10810,AT2G07490,AT3G56260,AT3G56290,AT4G02420,AT4G02260,AT4G01940,AT1G17050
ACACCGG	10	5	['12-16', '20-24', '0-4', '4-8']	97	AT2G06005,AT1G13930,AT3G01660,AT4G00400,AT2G40840,AT2G18790,AT2G45190,AT2G40610,AT3G50610,AT1G01470,AT1G18990,AT5G46760,AT4G16146,AT1G18180,AT4G17650,AT4G15810,AT2G16070,AT3G06160,AT2G44670,AT1G37537,AT1G31500,AT1G75140,AT2G39480,AT2G21240,AT5G19540,AT5G08330,AT2G42590,AT5G20280,AT2G38130,AT2G24820,AT4G36210,AT2G25210,AT1G26940,AT5G23240,AT3G18610,AT5G23050,AT1G26880,AT3G26380,AT2G21260,AT2G30150,AT1G63800,AT4G33700,AT2G42670,AT2G47410,AT4G31340,AT5G14760,AT3G24170,AT2G43750,AT2G43790,AT1G53210,AT4G29750,AT1G02140,AT5G27380,AT5G27010,AT1G32520,AT4G28210,AT3G20390,AT2G25080,AT1G53640,AT5G07400,AT5G06720,AT1G26590,AT5G65110,AT1G04530,AT4G24350,AT2G04390,AT1G05850,AT5G03190,AT3G14000,AT4G19710,AT1G07890,AT5G62090,AT5G62200,AT3G23325,AT3G15430,AT1G01940,AT3G02690,AT3G06510,AT4G13250,AT3G61830,AT2G36800,AT1G15520,AT4G11600,AT3G02930,AT3G08580,AT3G09750,AT5G57030,AT3G59770,AT3G59020,AT3G10810,AT2G07490,AT3G56260,AT3G56290,AT4G02420,AT4G02260,AT4G01940,AT1G17050
ACACGCG	10	1	['12-16']	92	AT3G05500,AT3G53190,AT2G18700,AT3G07700,AT5G49360,AT5G12470,AT3G03870,AT5G48250,AT1G13195,AT1G06310,AT4G17615,AT1G34000,AT2G14660,AT5G44730,AT1G20540,AT4G15840,AT1G78570,AT5G41810,AT5G40840,AT2G44770,AT2G10370,AT4G39100,AT2G39400,AT5G37260,AT5G08410,AT2G31800,AT3G52340,AT5G20070,AT4G37830,AT2G37760,AT4G36470,AT3G18610,AT3G24010,AT1G80440,AT1G51940,AT4G33640,AT2G44360,AT2G35880,AT5G16370,AT2G22500,AT2G45590,AT3G14900,AT2G43520,AT5G14800,AT2G42190,AT2G32950,AT3G22060,AT4G30350,AT3G15580,AT1G78995,AT4G30060,AT5G27830,AT5G12050,AT1G32520,AT4G28210,AT5G11340,AT3G26580,AT3G12800,AT5G65990,AT5G04590,AT5G03430,AT4G23060,AT3G14520,AT2G17840,AT3G17930,AT5G03140,AT5G02490,AT3G14000,AT5G62090,AT5G62130,AT1G76990,AT3G63060,AT3G02700,AT1G03970,AT3G61630,AT3G06500,AT4G11590,AT3G60260,AT5G57030,AT2G18230,AT3G59350,AT1G22590,AT2G20830,AT3G03160,AT5G54970,AT4G04330,AT2G20170,AT3G56660,AT1G73030,AT3G56090,AT1G50320,AT4G02195

ACACGCG	10	2	['12-16']	92	AT3G05500,AT3G53190,AT2G18700,AT3G07700,AT5G49360,AT5G12470,AT3G03870,AT5G48250,AT1G13195,AT1G06310,AT4G17615,AT1G34000,AT2G14660,AT5G44730,AT1G20540,AT4G15840,AT1G78570,AT5G41810,AT5G40840,AT2G44770,AT2G10370,AT4G39100,AT2G39400,AT5G37260,AT5G08410,AT2G31800,AT3G52340,AT5G20070,AT4G37830,AT2G37760,AT4G36470,AT3G18610,AT3G24010,AT1G80440,AT1G51940,AT4G33640,AT2G44360,AT2G35880,AT5G16370,AT2G22500,AT2G45590,AT3G14900,AT2G43520,AT5G14800,AT2G42190,AT2G32950,AT3G22060,AT4G30350,AT3G15580,AT1G78995,AT4G30060,AT5G27830,AT5G12050,AT1G32520,AT4G28210,AT5G11340,AT3G26580,AT3G12800,AT5G65990,AT5G04590,AT5G03430,AT4G23060,AT3G14520,AT2G17840,AT3G17930,AT5G03140,AT5G02490,AT3G14000,AT5G62090,AT5G62130,AT1G76990,AT3G63060,AT3G02700,AT1G03970,AT3G61630,AT3G06500,AT4G11590,AT3G60260,AT5G57030,AT2G18230,AT3G59350,AT1G22590,AT2G20830,AT3G03160,AT5G54970,AT4G04330,AT2G20170,AT3G56660,AT1G73030,AT3G56090,AT1G50320,AT4G02195
ACACGCG	10	3	['12-16']	92	AT3G05500,AT3G53190,AT2G18700,AT3G07700,AT5G49360,AT5G12470,AT3G03870,AT5G48250,AT1G13195,AT1G06310,AT4G17615,AT1G34000,AT2G14660,AT5G44730,AT1G20540,AT4G15840,AT1G78570,AT5G41810,AT5G40840,AT2G44770,AT2G10370,AT4G39100,AT2G39400,AT5G37260,AT5G08410,AT2G31800,AT3G52340,AT5G20070,AT4G37830,AT2G37760,AT4G36470,AT3G18610,AT3G24010,AT1G80440,AT1G51940,AT4G33640,AT2G44360,AT2G35880,AT5G16370,AT2G22500,AT2G45590,AT3G14900,AT2G43520,AT5G14800,AT2G42190,AT2G32950,AT3G22060,AT4G30350,AT3G15580,AT1G78995,AT4G30060,AT5G27830,AT5G12050,AT1G32520,AT4G28210,AT5G11340,AT3G26580,AT3G12800,AT5G65990,AT5G04590,AT5G03430,AT4G23060,AT3G14520,AT2G17840,AT3G17930,AT5G03140,AT5G02490,AT3G14000,AT5G62090,AT5G62130,AT1G76990,AT3G63060,AT3G02700,AT1G03970,AT3G61630,AT3G06500,AT4G11590,AT3G60260,AT5G57030,AT2G18230,AT3G59350,AT1G22590,AT2G20830,AT3G03160,AT5G54970,AT4G04330,AT2G20170,AT3G56660,AT1G73030,AT3G56090,AT1G50320,AT4G02195
ACACGCG	10	4	['12-16']	92	AT3G05500,AT3G53190,AT2G18700,AT3G07700,AT5G49360,AT5G12470,AT3G03870,AT5G48250,AT1G13195,AT1G06310,AT4G17615,AT1G34000,AT2G14660,AT5G44730,AT1G20540,AT4G15840,AT1G78570,AT5G41810,AT5G40840,AT2G44770,AT2G10370,AT4G39100,AT2G39400,AT5G37260,AT5G08410,AT2G31800,AT3G52340,AT5G20070,AT4G37830,AT2G37760,AT4G36470,AT3G18610,AT3G24010,AT1G80440,AT1G51940,AT4G33640,AT2G44360,AT2G35880,AT5G16370,AT2G22500,AT2G45590,AT3G14900,AT2G43520,AT5G14800,AT2G42190,AT2G32950,AT3G22060,AT4G30350,AT3G15580,AT1G78995,AT4G30060,AT5G27830,AT5G12050,AT1G32520,AT4G28210,AT5G11340,AT3G26580,AT3G12800,AT5G65990,AT5G04590,AT5G03430,AT4G23060,AT3G14520,AT2G17840,AT3G17930,AT5G03140,AT5G02490,AT3G14000,AT5G62090,AT5G62130,AT1G76990,AT3G63060,AT3G02700,AT1G03970,AT3G61630,AT3G06500,AT4G11590,AT3G60260,AT5G57030,AT2G18230,AT3G59350,AT1G22590,AT2G20830,AT3G03160,AT5G54970,AT4G04330,AT2G20170,AT3G56660,AT1G73030,AT3G56090,AT1G50320,AT4G02195
ACACGCG	10	5	['12-16']	92	AT3G05500,AT3G53190,AT2G18700,AT3G07700,AT5G49360,AT5G12470,AT3G03870,AT5G48250,AT1G13195,AT1G06310,AT4G17615,AT1G34000,AT2G14660,AT5G44730,AT1G20540,AT4G15840,AT1G78570,AT5G41810,AT5G40840,AT2G44770,AT2G10370,AT4G39100,AT2G39400,AT5G37260,AT5G08410,AT2G31800,AT3G52340,AT5G20070,AT4G37830,AT2G37760,AT4G36470,AT3G18610,AT3G24010,AT1G80440,AT1G51940,AT4G33640,AT2G44360,AT2G35880,AT5G16370,AT2G22500,AT2G45590,AT3G14900,AT2G43520,AT5G14800,AT2G42190,AT2G32950,AT3G22060,AT4G30350,AT3G15580,AT1G78995,AT4G30060,AT5G27830,AT5G12050,AT1G32520,AT4G28210,AT5G11340,AT3G26580,AT3G12800,AT5G65990,AT5G04590,AT5G03430,AT4G23060,AT3G14520,AT2G17840,AT3G17930,AT5G03140,AT5G02490,AT3G14000,AT5G62090,AT5G62130,AT1G76990,AT3G63060,AT3G02700,AT1G03970,AT3G61630,AT3G06500,AT4G11590,AT3G60260,AT5G57030,AT2G18230,AT3G59350,AT1G22590,AT2G20830,AT3G03160,AT5G54970,AT4G04330,AT2G20170,AT3G56660,AT1G73030,AT3G56090,AT1G50320,AT4G02195
ACACGGG	10	1	['8-12', '16-20', '20-24']	60	AT2G07730,AT1G22750,AT4G18120,AT1G32310,AT5G59850,AT3G02660,AT4G39120,AT5G59540,AT3G06510,AT2G22250,AT1G73670,AT1G19920,AT3G61440,AT4G12320,AT2G42580,AT5G47910,AT1G36390,AT5G08110,AT5G57815,AT1G22200,AT3G08570,AT3G17130,AT1G53450,AT5G23760,AT1G70730,AT4G25280,AT1G44000,AT3G59940,AT3G05880,AT1G31814,AT4G08690,AT1G64640,AT3G51820,AT4G17640,AT5G04940,AT1G56090,AT3G58680,AT5G04590,AT4G16520,AT1G51640,AT3G10050,AT2G36320,AT5G54510,AT5G16715,AT2G27490,AT3G13070,AT3G01990,AT1G70820,AT4G20360,AT2G17340,AT1G72960,AT3G55630,AT4G19860,AT2G43790,AT4G02420,AT3G15850,AT1G73390,AT1G35150,AT5G01750,AT5G12150

ACACGGG	10	2	['8-12', '16-20', '20-24']	60	AT2G07730,AT1G22750,AT4G18120,AT1G32310,AT5G59850,AT3G02660,AT4G39120,AT5G59540,AT3G06510,AT2G22250,AT1G73670,AT1G19920,AT3G61440,AT4G12320,AT2G42580,AT5G47910,AT1G36390,AT5G08110,AT5G57815,AT1G22200,AT3G08570,AT3G17130,AT1G53450,AT5G23760,AT1G70730,AT4G25280,AT1G44000,AT3G59940,AT3G05880,AT1G31814,AT4G08690,AT1G64640,AT3G51820,AT4G17640,AT5G04940,AT1G56090,AT3G58680,AT5G04590,AT4G16520,AT1G51640,AT3G10050,AT2G36320,AT5G54510,AT5G16715,AT2G27490,AT3G13070,AT3G01990,AT1G70820,AT4G20360,AT2G17340,AT1G72960,AT3G55630,AT4G19860,AT2G43790,AT4G02420,AT3G15850,AT1G73390,AT1G35150,AT5G01750,AT5G12150
ACACGGG	10	3	['8-12', '16-20', '20-24']	60	AT2G07730,AT1G22750,AT4G18120,AT1G32310,AT5G59850,AT3G02660,AT4G39120,AT5G59540,AT3G06510,AT2G22250,AT1G73670,AT1G19920,AT3G61440,AT4G12320,AT2G42580,AT5G47910,AT1G36390,AT5G08110,AT5G57815,AT1G22200,AT3G08570,AT3G17130,AT1G53450,AT5G23760,AT1G70730,AT4G25280,AT1G44000,AT3G59940,AT3G05880,AT1G31814,AT4G08690,AT1G64640,AT3G51820,AT4G17640,AT5G04940,AT1G56090,AT3G58680,AT5G04590,AT4G16520,AT1G51640,AT3G10050,AT2G36320,AT5G54510,AT5G16715,AT2G27490,AT3G13070,AT3G01990,AT1G70820,AT4G20360,AT2G17340,AT1G72960,AT3G55630,AT4G19860,AT2G43790,AT4G02420,AT3G15850,AT1G73390,AT1G35150,AT5G01750,AT5G12150
ACACGGG	10	4	['8-12', '16-20', '20-24']	60	AT2G07730,AT1G22750,AT4G18120,AT1G32310,AT5G59850,AT3G02660,AT4G39120,AT5G59540,AT3G06510,AT2G22250,AT1G73670,AT1G19920,AT3G61440,AT4G12320,AT2G42580,AT5G47910,AT1G36390,AT5G08110,AT5G57815,AT1G22200,AT3G08570,AT3G17130,AT1G53450,AT5G23760,AT1G70730,AT4G25280,AT1G44000,AT3G59940,AT3G05880,AT1G31814,AT4G08690,AT1G64640,AT3G51820,AT4G17640,AT5G04940,AT1G56090,AT3G58680,AT5G04590,AT4G16520,AT1G51640,AT3G10050,AT2G36320,AT5G54510,AT5G16715,AT2G27490,AT3G13070,AT3G01990,AT1G70820,AT4G20360,AT2G17340,AT1G72960,AT3G55630,AT4G19860,AT2G43790,AT4G02420,AT3G15850,AT1G73390,AT1G35150,AT5G01750,AT5G12150
ACACGGG	10	5	['8-12', '20-24', '16-20']	60	AT2G07730,AT1G22750,AT4G18120,AT1G32310,AT5G59850,AT3G02660,AT4G39120,AT5G59540,AT3G06510,AT2G22250,AT1G73670,AT1G19920,AT3G61440,AT4G12320,AT2G42580,AT5G47910,AT1G36390,AT5G08110,AT5G57815,AT1G22200,AT3G08570,AT3G17130,AT1G53450,AT5G23760,AT1G70730,AT4G25280,AT1G44000,AT3G59940,AT3G05880,AT1G31814,AT4G08690,AT1G64640,AT3G51820,AT4G17640,AT5G04940,AT1G56090,AT3G58680,AT5G04590,AT4G16520,AT1G51640,AT3G10050,AT2G36320,AT5G54510,AT5G16715,AT2G27490,AT3G13070,AT3G01990,AT1G70820,AT4G20360,AT2G17340,AT1G72960,AT3G55630,AT4G19860,AT2G43790,AT4G02420,AT3G15850,AT1G73390,AT1G35150,AT5G01750,AT5G12150
ACCTACC	10	1	['20-24', '8-12']	197	AT3G05500,AT5G50990,AT1G31280,AT3G53470,AT1G15400,AT2G26460,AT4G00660,AT4G00370,AT2G18770,AT3G52950,AT3G53030,AT1G06430,AT1G75950,AT1G19920,AT3G05130,AT1G16520,AT2G33420,AT3G03770,AT1G13440,AT4G25570,AT4G25530,AT1G22360,AT1G22370,AT5G47870,AT3G50810,AT1G07010,AT4G14930,AT1G07040,AT1G43930,AT3G47960,AT2G06925,AT5G45300,AT1G18170,AT5G44910,AT1G54500,AT1G36830,AT3G47560,AT4G16190,AT1G23980,AT3G46170,AT4G15160,AT1G78480,AT5G42470,AT1G56700,AT1G29510,AT1G78570,AT2G19830,AT1G72030,AT1G54880,AT2G47010,AT5G41050,AT3G11050,AT1G72140,AT2G25930,AT1G10470,AT1G72800,AT2G40150,AT2G31710,AT2G28720,AT4G38690,AT4G38570,AT3G12030,AT3G13620,AT3G52290,AT2G41430,AT2G31750,AT3G52340,AT5G35540,AT2G30860,AT5G20380,AT3G52240,AT2G25210,AT2G30900,AT5G24380,AT4G35060,AT2G02570,AT2G21300,AT5G22310,AT3G22530,AT3G51820,AT4G34190,AT1G80280,AT5G22300,AT1G70610,AT2G39930,AT2G37040,AT2G44300,AT2G44500,AT4G33490,AT4G33430,AT5G18340,AT1G69880,AT5G17780,AT5G16970,AT2G30490,AT2G36430,AT2G41870,AT2G32720,AT2G41760,AT2G41720,AT2G32765,AT5G14640,AT5G14660,AT3G24170,AT5G35180,AT5G13950,AT3G26570,AT1G79050,AT5G13650,AT5G27650,AT1G02140,AT1G65430,AT5G27380,AT5G27280,AT4G28660,AT4G28460,AT5G11500,AT1G09160,AT4G27470,AT5G24930,AT5G08580,AT1G21400,AT3G27050,AT1G10370,AT1G06040,AT3G26710,AT5G66710,AT5G66920,AT1G26560,AT4G25760,AT1G01240,AT5G65990,AT4G25450,AT1G17460,AT5G65430,AT4G24860,AT5G05110,AT5G64860,AT1G09960,AT5G64460,AT3G14620,AT4G23060,AT1G23010,AT1G26760,AT3G24590,AT4G22300,AT4G22310,AT4G20840,AT5G63370,AT5G02820,AT1G79790,AT3G21670,AT4G19710,AT5G62200,AT1G61210,AT5G62130,AT5G02160,AT3G15610,AT5G61990,AT3G14270,AT1G01790,AT3G15450,AT1G67360,AT5G60540,AT3G62410,AT1G49740,AT1G24575,AT1G50020,AT3G02468,AT3G02590,AT4G12320,AT1G76150,AT5G57630,AT1G08130,AT4G10360,AT1G50480,AT1G65060,AT4G09570,AT4G08970,AT2G22660,AT4G08690,AT5G55620,AT3G04550,AT3G58230,AT3G07200,AT3G03160,AT5G54950,AT2G07490,AT3G06410,AT3G06350,AT5G53170,AT3G55760,AT3G01860,AT3G55630,AT1G47530,AT3G55120,AT4G00830

ACCTACC	10	2	['8-12', '20-24']	197	AT3G05500,AT5G50990,AT1G31280,AT3G53470,AT1G15400,AT2G26460,AT4G00660,AT4G00370,AT2G18770,AT3G52950,AT3G53030,AT1G06430,AT1G75950,AT1G19920,AT3G05130,AT1G16520,AT2G33420,AT3G03770,AT1G13440,AT4G25570,AT4G25530,AT1G22360,AT1G22370,AT5G47870,AT3G50810,AT1G07010,AT4G14930,AT1G07040,AT1G43930,AT3G47960,AT2G06925,AT5G45300,AT1G18170,AT5G44910,AT1G54500,AT1G36830,AT3G47560,AT4G16190,AT1G23980,AT3G46170,AT4G15160,AT1G78480,AT5G42470,AT1G56700,AT1G29510,AT1G78570,AT2G19830,AT1G72030,AT1G54880,AT2G47010,AT5G41050,AT3G11050,AT1G72140,AT2G25930,AT1G10470,AT1G72800,AT2G40150,AT2G31710,AT2G28720,AT4G38690,AT4G38570,AT3G12030,AT3G13620,AT3G52290,AT2G41430,AT2G31750,AT3G52340,AT5G35540,AT2G30860,AT5G20380,AT3G52240,AT2G25210,AT2G30900,AT5G24380,AT4G35060,AT2G02570,AT2G21300,AT5G22310,AT3G22530,AT3G51820,AT4G34190,AT1G80280,AT5G22300,AT1G70610,AT2G39930,AT2G37040,AT2G44300,AT2G44500,AT4G33490,AT4G33430,AT5G18340,AT1G69880,AT5G17780,AT5G16970,AT2G30490,AT2G36430,AT2G41870,AT2G32720,AT2G41760,AT2G41720,AT2G32765,AT5G14640,AT5G14660,AT3G24170,AT5G35180,AT5G13950,AT3G26570,AT1G79050,AT5G13650,AT5G27650,AT1G02140,AT1G65430,AT5G27380,AT5G27280,AT4G28660,AT4G28460,AT5G11500,AT1G09160,AT4G27470,AT5G24930,AT5G08580,AT1G21400,AT3G27050,AT1G10370,AT1G06040,AT3G26710,AT5G66710,AT5G66920,AT1G26560,AT4G25760,AT1G01240,AT5G65990,AT4G25450,AT1G17460,AT5G65430,AT4G24860,AT5G05110,AT5G64860,AT1G09960,AT5G64460,AT3G14620,AT4G23060,AT1G23010,AT1G26760,AT3G24590,AT4G22300,AT4G22310,AT4G20840,AT5G63370,AT5G02820,AT1G79790,AT3G21670,AT4G19710,AT5G62200,AT1G61210,AT5G62130,AT5G02160,AT3G15610,AT5G61990,AT3G14270,AT1G01790,AT3G15450,AT1G67360,AT5G60540,AT3G62410,AT1G49740,AT1G24575,AT1G50020,AT3G02468,AT3G02590,AT4G12320,AT1G76150,AT5G57630,AT1G08130,AT4G10360,AT1G50480,AT1G65060,AT4G09570,AT4G08970,AT2G22660,AT4G08690,AT5G55620,AT3G04550,AT3G58230,AT3G07200,AT3G03160,AT5G54950,AT2G07490,AT3G06410,AT3G06350,AT5G53170,AT3G55760,AT3G01860,AT3G55630,AT1G47530,AT3G55120,AT4G00830
ACCTACC	10	3	['8-12', '20-24']	197	AT3G05500,AT5G50990,AT1G31280,AT3G53470,AT1G15400,AT2G26460,AT4G00660,AT4G00370,AT2G18770,AT3G52950,AT3G53030,AT1G06430,AT1G75950,AT1G19920,AT3G05130,AT1G16520,AT2G33420,AT3G03770,AT1G13440,AT4G25570,AT4G25530,AT1G22360,AT1G22370,AT5G47870,AT3G50810,AT1G07010,AT4G14930,AT1G07040,AT1G43930,AT3G47960,AT2G06925,AT5G45300,AT1G18170,AT5G44910,AT1G54500,AT1G36830,AT3G47560,AT4G16190,AT1G23980,AT3G46170,AT4G15160,AT1G78480,AT5G42470,AT1G56700,AT1G29510,AT1G78570,AT2G19830,AT1G72030,AT1G54880,AT2G47010,AT5G41050,AT3G11050,AT1G72140,AT2G25930,AT1G10470,AT1G72800,AT2G40150,AT2G31710,AT2G28720,AT4G38690,AT4G38570,AT3G12030,AT3G13620,AT3G52290,AT2G41430,AT2G31750,AT3G52340,AT5G35540,AT2G30860,AT5G20380,AT3G52240,AT2G25210,AT2G30900,AT5G24380,AT4G35060,AT2G02570,AT2G21300,AT5G22310,AT3G22530,AT3G51820,AT4G34190,AT1G80280,AT5G22300,AT1G70610,AT2G39930,AT2G37040,AT2G44300,AT2G44500,AT4G33490,AT4G33430,AT5G18340,AT1G69880,AT5G17780,AT5G16970,AT2G30490,AT2G36430,AT2G41870,AT2G32720,AT2G41760,AT2G41720,AT2G32765,AT5G14640,AT5G14660,AT3G24170,AT5G35180,AT5G13950,AT3G26570,AT1G79050,AT5G13650,AT5G27650,AT1G02140,AT1G65430,AT5G27380,AT5G27280,AT4G28660,AT4G28460,AT5G11500,AT1G09160,AT4G27470,AT5G24930,AT5G08580,AT1G21400,AT3G27050,AT1G10370,AT1G06040,AT3G26710,AT5G66710,AT5G66920,AT1G26560,AT4G25760,AT1G01240,AT5G65990,AT4G25450,AT1G17460,AT5G65430,AT4G24860,AT5G05110,AT5G64860,AT1G09960,AT5G64460,AT3G14620,AT4G23060,AT1G23010,AT1G26760,AT3G24590,AT4G22300,AT4G22310,AT4G20840,AT5G63370,AT5G02820,AT1G79790,AT3G21670,AT4G19710,AT5G62200,AT1G61210,AT5G62130,AT5G02160,AT3G15610,AT5G61990,AT3G14270,AT1G01790,AT3G15450,AT1G67360,AT5G60540,AT3G62410,AT1G49740,AT1G24575,AT1G50020,AT3G02468,AT3G02590,AT4G12320,AT1G76150,AT5G57630,AT1G08130,AT4G10360,AT1G50480,AT1G65060,AT4G09570,AT4G08970,AT2G22660,AT4G08690,AT5G55620,AT3G04550,AT3G58230,AT3G07200,AT3G03160,AT5G54950,AT2G07490,AT3G06410,AT3G06350,AT5G53170,AT3G55760,AT3G01860,AT3G55630,AT1G47530,AT3G55120,AT4G00830

ACCTACC	10	4	['20-24', '8-12']	197	AT3G05500,AT5G50990,AT1G31280,AT3G53470,AT1G15400,AT2G26460,AT4G00660,AT4G00370,AT2G18770,AT3G52950,AT3G53030,AT1G06430,AT1G75950,AT1G19920,AT3G05130,AT1G16520,AT2G33420,AT3G03770,AT1G13440,AT4G25570,AT4G25530,AT1G22360,AT1G22370,AT5G47870,AT3G50810,AT1G07010,AT4G14930,AT1G07040,AT1G43930,AT3G47960,AT2G06925,AT5G45300,AT1G18170,AT5G44910,AT1G54500,AT1G36830,AT3G47560,AT4G16190,AT1G23980,AT3G46170,AT4G15160,AT1G78480,AT5G42470,AT1G56700,AT1G29510,AT1G78570,AT2G19830,AT1G72030,AT1G54880,AT2G47010,AT5G41050,AT3G11050,AT1G72140,AT2G25930,AT1G10470,AT1G72800,AT2G40150,AT2G31710,AT2G28720,AT4G38690,AT4G38570,AT3G12030,AT3G13620,AT3G52290,AT2G41430,AT2G31750,AT3G52340,AT5G35540,AT2G30860,AT5G20380,AT3G52240,AT2G25210,AT2G30900,AT5G24380,AT4G35060,AT2G02570,AT2G21300,AT5G22310,AT3G22530,AT3G51820,AT4G34190,AT1G80280,AT5G22300,AT1G70610,AT2G39930,AT2G37040,AT2G44300,AT2G44500,AT4G33490,AT4G33430,AT5G18340,AT1G69880,AT5G17780,AT5G16970,AT2G30490,AT2G36430,AT2G41870,AT2G32720,AT2G41760,AT2G41720,AT2G32765,AT5G14640,AT5G14660,AT3G24170,AT5G35180,AT5G13950,AT3G26570,AT1G79050,AT5G13650,AT5G27650,AT1G02140,AT1G65430,AT5G27380,AT5G27280,AT4G28660,AT4G28460,AT5G11500,AT1G09160,AT4G27470,AT5G24930,AT5G08580,AT1G21400,AT3G27050,AT1G10370,AT1G06040,AT3G26710,AT5G66710,AT5G66920,AT1G26560,AT4G25760,AT1G01240,AT5G65990,AT4G25450,AT1G17460,AT5G65430,AT4G24860,AT5G05110,AT5G64860,AT1G09960,AT5G64460,AT3G14620,AT4G23060,AT1G23010,AT1G26760,AT3G24590,AT4G22300,AT4G22310,AT4G20840,AT5G63370,AT5G02820,AT1G79790,AT3G21670,AT4G19710,AT5G62200,AT1G61210,AT5G62130,AT5G02160,AT3G15610,AT5G61990,AT3G14270,AT1G01790,AT3G15450,AT1G67360,AT5G60540,AT3G62410,AT1G49740,AT1G24575,AT1G50020,AT3G02468,AT3G02590,AT4G12320,AT1G76150,AT5G57630,AT1G08130,AT4G10360,AT1G50480,AT1G65060,AT4G09570,AT4G08970,AT2G22660,AT4G08690,AT5G55620,AT3G04550,AT3G58230,AT3G07200,AT3G03160,AT5G54950,AT2G07490,AT3G06410,AT3G06350,AT5G53170,AT3G55760,AT3G01860,AT3G55630,AT1G47530,AT3G55120,AT4G00830
ACCTACC	10	5	['8-12', '20-24']	197	AT3G05500,AT5G50990,AT1G31280,AT3G53470,AT1G15400,AT2G26460,AT4G00660,AT4G00370,AT2G18770,AT3G52950,AT3G53030,AT1G06430,AT1G75950,AT1G19920,AT3G05130,AT1G16520,AT2G33420,AT3G03770,AT1G13440,AT4G25570,AT4G25530,AT1G22360,AT1G22370,AT5G47870,AT3G50810,AT1G07010,AT4G14930,AT1G07040,AT1G43930,AT3G47960,AT2G06925,AT5G45300,AT1G18170,AT5G44910,AT1G54500,AT1G36830,AT3G47560,AT4G16190,AT1G23980,AT3G46170,AT4G15160,AT1G78480,AT5G42470,AT1G56700,AT1G29510,AT1G78570,AT2G19830,AT1G72030,AT1G54880,AT2G47010,AT5G41050,AT3G11050,AT1G72140,AT2G25930,AT1G10470,AT1G72800,AT2G40150,AT2G31710,AT2G28720,AT4G38690,AT4G38570,AT3G12030,AT3G13620,AT3G52290,AT2G41430,AT2G31750,AT3G52340,AT5G35540,AT2G30860,AT5G20380,AT3G52240,AT2G25210,AT2G30900,AT5G24380,AT4G35060,AT2G02570,AT2G21300,AT5G22310,AT3G22530,AT3G51820,AT4G34190,AT1G80280,AT5G22300,AT1G70610,AT2G39930,AT2G37040,AT2G44300,AT2G44500,AT4G33490,AT4G33430,AT5G18340,AT1G69880,AT5G17780,AT5G16970,AT2G30490,AT2G36430,AT2G41870,AT2G32720,AT2G41760,AT2G41720,AT2G32765,AT5G14640,AT5G14660,AT3G24170,AT5G35180,AT5G13950,AT3G26570,AT1G79050,AT5G13650,AT5G27650,AT1G02140,AT1G65430,AT5G27380,AT5G27280,AT4G28660,AT4G28460,AT5G11500,AT1G09160,AT4G27470,AT5G24930,AT5G08580,AT1G21400,AT3G27050,AT1G10370,AT1G06040,AT3G26710,AT5G66710,AT5G66920,AT1G26560,AT4G25760,AT1G01240,AT5G65990,AT4G25450,AT1G17460,AT5G65430,AT4G24860,AT5G05110,AT5G64860,AT1G09960,AT5G64460,AT3G14620,AT4G23060,AT1G23010,AT1G26760,AT3G24590,AT4G22300,AT4G22310,AT4G20840,AT5G63370,AT5G02820,AT1G79790,AT3G21670,AT4G19710,AT5G62200,AT1G61210,AT5G62130,AT5G02160,AT3G15610,AT5G61990,AT3G14270,AT1G01790,AT3G15450,AT1G67360,AT5G60540,AT3G62410,AT1G49740,AT1G24575,AT1G50020,AT3G02468,AT3G02590,AT4G12320,AT1G76150,AT5G57630,AT1G08130,AT4G10360,AT1G50480,AT1G65060,AT4G09570,AT4G08970,AT2G22660,AT4G08690,AT5G55620,AT3G04550,AT3G58230,AT3G07200,AT3G03160,AT5G54950,AT2G07490,AT3G06410,AT3G06350,AT5G53170,AT3G55760,AT3G01860,AT3G55630,AT1G47530,AT3G55120,AT4G00830

ACGTGGCA	10	1	['4-8', '12-16']	120	AT5G51110,AT2G37240,AT2G37450,AT2G07738,AT1G14150,AT5G49540,AT1G06680,AT2G38000,AT3G07680,AT3G05130,AT1G76030,AT2G47490,AT1G28530,AT2G27710,AT3G04860,AT5G47640,AT1G22370,AT1G02340,AT1G01520,AT1G21000,AT4G16515,AT2G06980,AT4G17140,AT3G46780,AT1G30130,AT1G56220,AT1G71020,AT1G05570,AT5G41050,AT1G52230,AT1G68440,AT5G39570,AT1G75210,AT1G53670,AT5G19860,AT2G34250,AT3G19970,AT2G31790,AT5G19940,AT3G29320,AT4G35850,AT4G37240,AT3G22970,AT1G26940,AT3G22890,AT2G21300,AT2G21330,AT2G40400,AT2G40080,AT3G19100,AT4G33980,AT3G15210,AT2G04550,AT5G18340,AT2G36390,AT1G69830,AT1G10960,AT5G15230,AT2G41760,AT2G47400,AT5G15510,AT3G14930,AT2G42220,AT1G79040,AT1G79270,AT1G30800,AT4G28750,AT4G27410,AT4G26850,AT5G67480,AT5G06690,AT5G66570,AT3G17000,AT5G65630,AT1G17460,AT5G65430,AT5G05300,AT1G08890,AT5G64840,AT5G05200,AT5G64260,AT5G64180,AT2G17840,AT5G03190,AT3G13980,AT4G19710,AT1G18740,AT3G63520,AT5G61530,AT3G63060,AT1G55510,AT1G18330,AT1G18310,AT1G62620,AT3G61820,AT3G61830,AT4G13010,AT5G58070,AT1G76150,AT2G42930,AT5G57345,AT2G18350,AT3G60200,AT4G09620,AT4G09020,AT3G10720,AT3G59220,AT1G78020,AT1G74880,AT3G10670,AT4G03560,AT5G53880,AT3G56490,AT1G27600,AT3G56050,AT3G01860,AT1G11130,AT4G01610,AT3G54500,AT3G54680
ACGTGGCA	10	2	['4-8', '12-16']	120	AT5G51110,AT2G37240,AT2G37450,AT2G07738,AT1G14150,AT5G49540,AT1G06680,AT2G38000,AT3G07680,AT3G05130,AT1G76030,AT2G47490,AT1G28530,AT2G27710,AT3G04860,AT5G47640,AT1G22370,AT1G02340,AT1G01520,AT1G21000,AT4G16515,AT2G06980,AT4G17140,AT3G46780,AT1G30130,AT1G56220,AT1G71020,AT1G05570,AT5G41050,AT1G52230,AT1G68440,AT5G39570,AT1G75210,AT1G53670,AT5G19860,AT2G34250,AT3G19970,AT2G31790,AT5G19940,AT3G29320,AT4G35850,AT4G37240,AT3G22970,AT1G26940,AT3G22890,AT2G21300,AT2G21330,AT2G40400,AT2G40080,AT3G19100,AT4G33980,AT3G15210,AT2G04550,AT5G18340,AT2G36390,AT1G69830,AT1G10960,AT5G15230,AT2G41760,AT2G47400,AT5G15510,AT3G14930,AT2G42220,AT1G79040,AT1G79270,AT1G30800,AT4G28750,AT4G27410,AT4G26850,AT5G67480,AT5G06690,AT5G66570,AT3G17000,AT5G65630,AT1G17460,AT5G65430,AT5G05300,AT1G08890,AT5G64840,AT5G05200,AT5G64260,AT5G64180,AT2G17840,AT5G03190,AT3G13980,AT4G19710,AT1G18740,AT3G63520,AT5G61530,AT3G63060,AT1G55510,AT1G18330,AT1G18310,AT1G62620,AT3G61820,AT3G61830,AT4G13010,AT5G58070,AT1G76150,AT2G42930,AT5G57345,AT2G18350,AT3G60200,AT4G09620,AT4G09020,AT3G10720,AT3G59220,AT1G78020,AT1G74880,AT3G10670,AT4G03560,AT5G53880,AT3G56490,AT1G27600,AT3G56050,AT3G01860,AT1G11130,AT4G01610,AT3G54500,AT3G54680
ACGTGGCA	10	3	['4-8', '12-16']	120	AT5G51110,AT2G37240,AT2G37450,AT2G07738,AT1G14150,AT5G49540,AT1G06680,AT2G38000,AT3G07680,AT3G05130,AT1G76030,AT2G47490,AT1G28530,AT2G27710,AT3G04860,AT5G47640,AT1G22370,AT1G02340,AT1G01520,AT1G21000,AT4G16515,AT2G06980,AT4G17140,AT3G46780,AT1G30130,AT1G56220,AT1G71020,AT1G05570,AT5G41050,AT1G52230,AT1G68440,AT5G39570,AT1G75210,AT1G53670,AT5G19860,AT2G34250,AT3G19970,AT2G31790,AT5G19940,AT3G29320,AT4G35850,AT4G37240,AT3G22970,AT1G26940,AT3G22890,AT2G21300,AT2G21330,AT2G40400,AT2G40080,AT3G19100,AT4G33980,AT3G15210,AT2G04550,AT5G18340,AT2G36390,AT1G69830,AT1G10960,AT5G15230,AT2G41760,AT2G47400,AT5G15510,AT3G14930,AT2G42220,AT1G79040,AT1G79270,AT1G30800,AT4G28750,AT4G27410,AT4G26850,AT5G67480,AT5G06690,AT5G66570,AT3G17000,AT5G65630,AT1G17460,AT5G65430,AT5G05300,AT1G08890,AT5G64840,AT5G05200,AT5G64260,AT5G64180,AT2G17840,AT5G03190,AT3G13980,AT4G19710,AT1G18740,AT3G63520,AT5G61530,AT3G63060,AT1G55510,AT1G18330,AT1G18310,AT1G62620,AT3G61820,AT3G61830,AT4G13010,AT5G58070,AT1G76150,AT2G42930,AT5G57345,AT2G18350,AT3G60200,AT4G09620,AT4G09020,AT3G10720,AT3G59220,AT1G78020,AT1G74880,AT3G10670,AT4G03560,AT5G53880,AT3G56490,AT1G27600,AT3G56050,AT3G01860,AT1G11130,AT4G01610,AT3G54500,AT3G54680

ACGTGGCA	10	4	['4-8']	120	AT5G51110,AT2G37240,AT2G37450,AT2G07738,AT1G14150,AT5G49540,AT1G06680,AT2G38000,AT3G07680,AT3G05130,AT1G76030,AT2G47490,AT1G28530,AT2G27710,AT3G04860,AT5G47640,AT1G22370,AT1G02340,AT1G01520,AT1G21000,AT4G16515,AT2G06980,AT4G17140,AT3G46780,AT1G30130,AT1G56220,AT1G71020,AT1G05570,AT5G41050,AT1G52230,AT1G68440,AT5G39570,AT1G75210,AT1G53670,AT5G19860,AT2G34250,AT3G19970,AT2G31790,AT5G19940,AT3G29320,AT4G35850,AT4G37240,AT3G22970,AT1G26940,AT3G22890,AT2G21300,AT2G21330,AT2G40400,AT2G40080,AT3G19100,AT4G33980,AT3G15210,AT2G04550,AT5G18340,AT2G36390,AT1G69830,AT1G10960,AT5G15230,AT2G41760,AT2G47400,AT5G15510,AT3G14930,AT2G42220,AT1G79040,AT1G79270,AT1G30800,AT4G28750,AT4G27410,AT4G26850,AT5G67480,AT5G06690,AT5G66570,AT3G17000,AT5G65630,AT1G17460,AT5G65430,AT5G05300,AT1G08890,AT5G64840,AT5G05200,AT5G64260,AT5G64180,AT2G17840,AT5G03190,AT3G13980,AT4G19710,AT1G18740,AT3G63520,AT5G61530,AT3G63060,AT1G55510,AT1G18330,AT1G18310,AT1G62620,AT3G61820,AT3G61830,AT4G13010,AT5G58070,AT1G76150,AT2G42930,AT5G57345,AT2G18350,AT3G60200,AT4G09620,AT4G09020,AT3G10720,AT3G59220,AT1G78020,AT1G74880,AT3G10670,AT4G03560,AT5G53880,AT3G56490,AT1G27600,AT3G56050,AT3G01860,AT1G11130,AT4G01610,AT3G54500,AT3G54680
ACGTGGCA	10	5	['4-8', '12-16']	120	AT5G51110,AT2G37240,AT2G37450,AT2G07738,AT1G14150,AT5G49540,AT1G06680,AT2G38000,AT3G07680,AT3G05130,AT1G76030,AT2G47490,AT1G28530,AT2G27710,AT3G04860,AT5G47640,AT1G22370,AT1G02340,AT1G01520,AT1G21000,AT4G16515,AT2G06980,AT4G17140,AT3G46780,AT1G30130,AT1G56220,AT1G71020,AT1G05570,AT5G41050,AT1G52230,AT1G68440,AT5G39570,AT1G75210,AT1G53670,AT5G19860,AT2G34250,AT3G19970,AT2G31790,AT5G19940,AT3G29320,AT4G35850,AT4G37240,AT3G22970,AT1G26940,AT3G22890,AT2G21300,AT2G21330,AT2G40400,AT2G40080,AT3G19100,AT4G33980,AT3G15210,AT2G04550,AT5G18340,AT2G36390,AT1G69830,AT1G10960,AT5G15230,AT2G41760,AT2G47400,AT5G15510,AT3G14930,AT2G42220,AT1G79040,AT1G79270,AT1G30800,AT4G28750,AT4G27410,AT4G26850,AT5G67480,AT5G06690,AT5G66570,AT3G17000,AT5G65630,AT1G17460,AT5G65430,AT5G05300,AT1G08890,AT5G64840,AT5G05200,AT5G64260,AT5G64180,AT2G17840,AT5G03190,AT3G13980,AT4G19710,AT1G18740,AT3G63520,AT5G61530,AT3G63060,AT1G55510,AT1G18330,AT1G18310,AT1G62620,AT3G61820,AT3G61830,AT4G13010,AT5G58070,AT1G76150,AT2G42930,AT5G57345,AT2G18350,AT3G60200,AT4G09620,AT4G09020,AT3G10720,AT3G59220,AT1G78020,AT1G74880,AT3G10670,AT4G03560,AT5G53880,AT3G56490,AT1G27600,AT3G56050,AT3G01860,AT1G11130,AT4G01610,AT3G54500,AT3G54680
ACTGTGTA	10	1	['0-4', '20-24']	94	AT5G51030,AT3G01310,AT4G38290,AT2G47600,AT1G19920,AT1G18020,AT1G13180,AT1G20840,AT5G46630,AT1G15750,AT4G17090,AT4G16330,AT3G47430,AT1G23960,AT3G12350,AT1G03055,AT1G54100,AT1G47900,AT2G46780,AT5G04170,AT5G04140,AT1G73470,AT5G11070,AT3G28780,AT1G73760,AT5G20320,AT2G04900,AT3G18610,AT4G34830,AT4G34190,AT5G19140,AT3G56700,AT4G33670,AT5G18340,AT2G02710,AT5G16620,AT2G22450,AT4G30470,AT3G26590,AT1G62430,AT1G08080,AT4G29890,AT4G28130,AT1G78260,AT5G10240,AT1G04140,AT1G04120,AT1G29240,AT4G26130,AT4G26050,AT4G25770,AT3G16960,AT5G65910,AT3G17040,AT4G24280,AT1G34440,AT1G09680,AT1G12900,AT5G64180,AT1G12845,AT5G03795,AT1G08540,AT5G03190,AT1G03600,AT3G15610,AT3G16170,AT1G71720,AT5G61530,AT4G18370,AT3G62720,AT1G24575,AT5G60120,AT3G62660,AT3G62370,AT3G06780,AT3G61630,AT1G18460,AT1G76270,AT3G08010,AT3G60810,AT2G28390,AT5G57110,AT3G59350,AT5G56230,AT5G56260,AT2G06510,AT1G80030,AT5G55480,AT4G05330,AT2G25510,AT1G73030,AT5G53370,AT4G02420,AT3G03420
ACTGTGTA	10	2	['0-4', '20-24']	94	AT5G51030,AT3G01310,AT4G38290,AT2G47600,AT1G19920,AT1G18020,AT1G13180,AT1G20840,AT5G46630,AT1G15750,AT4G17090,AT4G16330,AT3G47430,AT1G23960,AT3G12350,AT1G03055,AT1G54100,AT1G47900,AT2G46780,AT5G04170,AT5G04140,AT1G73470,AT5G11070,AT3G28780,AT1G73760,AT5G20320,AT2G04900,AT3G18610,AT4G34830,AT4G34190,AT5G19140,AT3G56700,AT4G33670,AT5G18340,AT2G02710,AT5G16620,AT2G22450,AT4G30470,AT3G26590,AT1G62430,AT1G08080,AT4G29890,AT4G28130,AT1G78260,AT5G10240,AT1G04140,AT1G04120,AT1G29240,AT4G26130,AT4G26050,AT4G25770,AT3G16960,AT5G65910,AT3G17040,AT4G24280,AT1G34440,AT1G09680,AT1G12900,AT5G64180,AT1G12845,AT5G03795,AT1G08540,AT5G03190,AT1G03600,AT3G15610,AT3G16170,AT1G71720,AT5G61530,AT4G18370,AT3G62720,AT1G24575,AT5G60120,AT3G62660,AT3G62370,AT3G06780,AT3G61630,AT1G18460,AT1G76270,AT3G08010,AT3G60810,AT2G28390,AT5G57110,AT3G59350,AT5G56230,AT5G56260,AT2G06510,AT1G80030,AT5G55480,AT4G05330,AT2G25510,AT1G73030,AT5G53370,AT4G02420,AT3G03420

ACTGTGTA	10	3	['0-4', '20-24']	94	AT5G51030,AT3G01310,AT4G38290,AT2G47600,AT1G19920,AT1G18020,AT1G13180,AT1G20840,AT5G46630,AT1G15750,AT4G17090,AT4G16330,AT3G47430,AT1G23960,AT3G12350,AT1G03055,AT1G54100,AT1G47900,AT2G46780,AT5G04170,AT5G04140,AT1G73470,AT5G11070,AT3G28780,AT1G73760,AT5G20320,AT2G04900,AT3G18610,AT4G34830,AT4G34190,AT5G19140,AT3G56700,AT4G33670,AT5G18340,AT2G02710,AT5G16620,AT2G22450,AT4G30470,AT3G26590,AT1G62430,AT1G08080,AT4G29890,AT4G28130,AT1G78260,AT5G10240,AT1G04140,AT1G04120,AT1G29240,AT4G26130,AT4G26050,AT4G25770,AT3G16960,AT5G65910,AT3G17040,AT4G24280,AT1G34440,AT1G09680,AT1G12900,AT5G64180,AT1G12845,AT5G03795,AT1G08540,AT5G03190,AT1G03600,AT3G15610,AT3G16170,AT1G71720,AT5G61530,AT4G18370,AT3G62720,AT1G24575,AT5G60120,AT3G62660,AT3G62370,AT3G06780,AT3G61630,AT1G18460,AT1G76270,AT3G08010,AT3G60810,AT2G28390,AT5G57110,AT3G59350,AT5G56230,AT5G56260,AT2G06510,AT1G80030,AT5G55480,AT4G05330,AT2G25510,AT1G73030,AT5G53370,AT4G02420,AT3G03420
ACTGTGTA	10	4	['0-4', '20-24']	94	AT5G51030,AT3G01310,AT4G38290,AT2G47600,AT1G19920,AT1G18020,AT1G13180,AT1G20840,AT5G46630,AT1G15750,AT4G17090,AT4G16330,AT3G47430,AT1G23960,AT3G12350,AT1G03055,AT1G54100,AT1G47900,AT2G46780,AT5G04170,AT5G04140,AT1G73470,AT5G11070,AT3G28780,AT1G73760,AT5G20320,AT2G04900,AT3G18610,AT4G34830,AT4G34190,AT5G19140,AT3G56700,AT4G33670,AT5G18340,AT2G02710,AT5G16620,AT2G22450,AT4G30470,AT3G26590,AT1G62430,AT1G08080,AT4G29890,AT4G28130,AT1G78260,AT5G10240,AT1G04140,AT1G04120,AT1G29240,AT4G26130,AT4G26050,AT4G25770,AT3G16960,AT5G65910,AT3G17040,AT4G24280,AT1G34440,AT1G09680,AT1G12900,AT5G64180,AT1G12845,AT5G03795,AT1G08540,AT5G03190,AT1G03600,AT3G15610,AT3G16170,AT1G71720,AT5G61530,AT4G18370,AT3G62720,AT1G24575,AT5G60120,AT3G62660,AT3G62370,AT3G06780,AT3G61630,AT1G18460,AT1G76270,AT3G08010,AT3G60810,AT2G28390,AT5G57110,AT3G59350,AT5G56230,AT5G56260,AT2G06510,AT1G80030,AT5G55480,AT4G05330,AT2G25510,AT1G73030,AT5G53370,AT4G02420,AT3G03420
ACTGTGTA	10	5	['20-24', '0-4']	94	AT5G51030,AT3G01310,AT4G38290,AT2G47600,AT1G19920,AT1G18020,AT1G13180,AT1G20840,AT5G46630,AT1G15750,AT4G17090,AT4G16330,AT3G47430,AT1G23960,AT3G12350,AT1G03055,AT1G54100,AT1G47900,AT2G46780,AT5G04170,AT5G04140,AT1G73470,AT5G11070,AT3G28780,AT1G73760,AT5G20320,AT2G04900,AT3G18610,AT4G34830,AT4G34190,AT5G19140,AT3G56700,AT4G33670,AT5G18340,AT2G02710,AT5G16620,AT2G22450,AT4G30470,AT3G26590,AT1G62430,AT1G08080,AT4G29890,AT4G28130,AT1G78260,AT5G10240,AT1G04140,AT1G04120,AT1G29240,AT4G26130,AT4G26050,AT4G25770,AT3G16960,AT5G65910,AT3G17040,AT4G24280,AT1G34440,AT1G09680,AT1G12900,AT5G64180,AT1G12845,AT5G03795,AT1G08540,AT5G03190,AT1G03600,AT3G15610,AT3G16170,AT1G71720,AT5G61530,AT4G18370,AT3G62720,AT1G24575,AT5G60120,AT3G62660,AT3G62370,AT3G06780,AT3G61630,AT1G18460,AT1G76270,AT3G08010,AT3G60810,AT2G28390,AT5G57110,AT3G59350,AT5G56230,AT5G56260,AT2G06510,AT1G80030,AT5G55480,AT4G05330,AT2G25510,AT1G73030,AT5G53370,AT4G02420,AT3G03420
AGAAACTTCT	10	1	['0-4', '4-8', '12-16', '16-20']	18	AT3G03830,AT4G18270,AT2G46400,AT3G21630,AT5G08030,AT5G67480,AT5G03140,AT5G20900,AT2G34430,AT1G53540,AT5G07400,AT1G04050,AT1G18330,AT1G22850,AT4G23180,AT3G23940,AT1G19140,AT1G22590
AGAAACTTCT	10	2	['0-4', '4-8', '12-16', '16-20']	18	AT3G03830,AT4G18270,AT2G46400,AT3G21630,AT5G08030,AT5G67480,AT5G03140,AT5G20900,AT2G34430,AT1G53540,AT5G07400,AT1G04050,AT1G18330,AT1G22850,AT4G23180,AT3G23940,AT1G19140,AT1G22590
AGAAACTTCT	10	3	['0-4', '4-8', '12-16', '16-20']	18	AT3G03830,AT4G18270,AT2G46400,AT3G21630,AT5G08030,AT5G67480,AT5G03140,AT5G20900,AT2G34430,AT1G53540,AT5G07400,AT1G04050,AT1G18330,AT1G22850,AT4G23180,AT3G23940,AT1G19140,AT1G22590
AGAAACTTCT	10	4	['0-4', '4-8', '12-16', '16-20']	18	AT3G03830,AT4G18270,AT2G46400,AT3G21630,AT5G08030,AT5G67480,AT5G03140,AT5G20900,AT2G34430,AT1G53540,AT5G07400,AT1G04050,AT1G18330,AT1G22850,AT4G23180,AT3G23940,AT1G19140,AT1G22590
AGAAACTTCT	10	5	['0-4', '4-8', '12-16', '16-20']	18	AT3G03830,AT4G18270,AT2G46400,AT3G21630,AT5G08030,AT5G67480,AT5G03140,AT5G20900,AT2G34430,AT1G53540,AT5G07400,AT1G04050,AT1G18330,AT1G22850,AT4G23180,AT3G23940,AT1G19140,AT1G22590
AGAAAGTTCT	10	1	['0-4', '4-8', '8-12', '12-16', '20-24']	17	AT2G19310,AT4G00770,AT3G04870,AT1G22200,AT1G70250,AT5G44410,AT3G62260,AT4G23290,AT3G10970,AT2G40700,AT5G41150,AT1G51080,AT5G28140,AT3G01060,AT1G23540,AT2G36240,AT5G65720

AGAAAGTTCT	10	2	['0-4', '4-8', '8-12', '12-16', '20-24']	17	AT2G19310,AT4G00770,AT3G04870,AT1G22200,AT1G70250,AT5G44410,AT3G62260,AT4G23290,AT3G10970,AT2G40700,AT5G41150,AT1G51080,AT5G28140,AT3G01060,AT1G23540,AT2G36240,AT5G65720
AGAAAGTTCT	10	3	['0-4', '4-8', '8-12', '12-16', '20-24']	17	AT2G19310,AT4G00770,AT3G04870,AT1G22200,AT1G70250,AT5G44410,AT3G62260,AT4G23290,AT3G10970,AT2G40700,AT5G41150,AT1G51080,AT5G28140,AT3G01060,AT1G23540,AT2G36240,AT5G65720
AGAAAGTTCT	10	4	['0-4', '4-8', '8-12', '12-16', '20-24']	17	AT2G19310,AT4G00770,AT3G04870,AT1G22200,AT1G70250,AT5G44410,AT3G62260,AT4G23290,AT3G10970,AT2G40700,AT5G41150,AT1G51080,AT5G28140,AT3G01060,AT1G23540,AT2G36240,AT5G65720
AGAAAGTTCT	10	5	['0-4', '4-8', '8-12', '12-16', '20-24']	17	AT2G19310,AT4G00770,AT3G04870,AT1G22200,AT1G70250,AT5G44410,AT3G62260,AT4G23290,AT3G10970,AT2G40700,AT5G41150,AT1G51080,AT5G28140,AT3G01060,AT1G23540,AT2G36240,AT5G65720
AGAACATTCT	10	1	['16-20', '4-8']	23	AT2G05070,AT3G26280,AT4G27680,AT1G14040,AT4G38460,AT2G02070,AT4G25570,AT2G29310,AT1G52870,AT3G42570,AT1G18900,AT3G05940,AT3G14080,AT1G62080,AT2G30390,AT5G53580,AT5G03050,AT5G02490,AT2G03750,AT5G02760,AT1G49250,AT1G73470,AT5G62280
AGAACATTCT	10	2	['16-20']	23	AT2G05070,AT3G26280,AT4G27680,AT1G14040,AT4G38460,AT2G02070,AT4G25570,AT2G29310,AT1G52870,AT3G42570,AT1G18900,AT3G05940,AT3G14080,AT1G62080,AT2G30390,AT5G53580,AT5G03050,AT5G02490,AT2G03750,AT5G02760,AT1G49250,AT1G73470,AT5G62280
AGAACATTCT	10	3	['16-20', '4-8']	23	AT2G05070,AT3G26280,AT4G27680,AT1G14040,AT4G38460,AT2G02070,AT4G25570,AT2G29310,AT1G52870,AT3G42570,AT1G18900,AT3G05940,AT3G14080,AT1G62080,AT2G30390,AT5G53580,AT5G03050,AT5G02490,AT2G03750,AT5G02760,AT1G49250,AT1G73470,AT5G62280
AGAACATTCT	10	4	['16-20']	23	AT2G05070,AT3G26280,AT4G27680,AT1G14040,AT4G38460,AT2G02070,AT4G25570,AT2G29310,AT1G52870,AT3G42570,AT1G18900,AT3G05940,AT3G14080,AT1G62080,AT2G30390,AT5G53580,AT5G03050,AT5G02490,AT2G03750,AT5G02760,AT1G49250,AT1G73470,AT5G62280
AGAACATTCT	10	5	['16-20', '4-8']	23	AT2G05070,AT3G26280,AT4G27680,AT1G14040,AT4G38460,AT2G02070,AT4G25570,AT2G29310,AT1G52870,AT3G42570,AT1G18900,AT3G05940,AT3G14080,AT1G62080,AT2G30390,AT5G53580,AT5G03050,AT5G02490,AT2G03750,AT5G02760,AT1G49250,AT1G73470,AT5G62280
AGAACTTTCT	10	1	['4-8', '8-12', '16-20', '20-24']	17	AT3G18930,AT3G03710,AT3G16690,AT5G24520,AT3G21690,AT3G62410,AT3G58140,AT5G57730,AT2G15670,AT4G02080,AT4G38570,AT1G06200,AT5G14090,AT5G57030,AT5G41150,AT5G03555,AT4G10300
AGAACTTTCT	10	2	['4-8', '8-12', '16-20', '20-24']	17	AT3G18930,AT3G03710,AT3G16690,AT5G24520,AT3G21690,AT3G62410,AT3G58140,AT5G57730,AT2G15670,AT4G02080,AT4G38570,AT1G06200,AT5G14090,AT5G57030,AT5G41150,AT5G03555,AT4G10300
AGAACTTTCT	10	3	['4-8', '8-12', '16-20', '20-24']	17	AT3G18930,AT3G03710,AT3G16690,AT5G24520,AT3G21690,AT3G62410,AT3G58140,AT5G57730,AT2G15670,AT4G02080,AT4G38570,AT1G06200,AT5G14090,AT5G57030,AT5G41150,AT5G03555,AT4G10300
AGAACTTTCT	10	4	['4-8', '8-12', '16-20', '20-24']	17	AT3G18930,AT3G03710,AT3G16690,AT5G24520,AT3G21690,AT3G62410,AT3G58140,AT5G57730,AT2G15670,AT4G02080,AT4G38570,AT1G06200,AT5G14090,AT5G57030,AT5G41150,AT5G03555,AT4G10300
AGAACTTTCT	10	5	['4-8', '8-12', '16-20', '20-24']	17	AT3G18930,AT3G03710,AT3G16690,AT5G24520,AT3G21690,AT3G62410,AT3G58140,AT5G57730,AT2G15670,AT4G02080,AT4G38570,AT1G06200,AT5G14090,AT5G57030,AT5G41150,AT5G03555,AT4G10300
AGAAGATTCT	10	1	['0-4', '4-8', '8-12', '16-20', '20-24']	22	AT2G45980,AT2G05820,AT5G37960,AT5G49120,AT5G48100,AT4G37480,AT4G36040,AT2G02515,AT4G10120,AT1G01230,AT5G05710,AT1G12860,AT1G36830,AT1G74920,AT5G03510,AT2G47390,AT3G44720,AT1G52230,AT3G15690,AT1G79370,AT3G16040,AT1G30270
AGAAGATTCT	10	2	['0-4', '4-8', '8-12', '16-20', '20-24']	22	AT2G45980,AT2G05820,AT5G37960,AT5G49120,AT5G48100,AT4G37480,AT4G36040,AT2G02515,AT4G10120,AT1G01230,AT5G05710,AT1G12860,AT1G36830,AT1G74920,AT5G03510,AT2G47390,AT3G44720,AT1G52230,AT3G15690,AT1G79370,AT3G16040,AT1G30270

AGAAGATTCT	10	3	['0-4', '4-8', '8-12', '16-20', '20-24']	22	AT2G45980,AT2G05820,AT5G37960,AT5G49120,AT5G48100,AT4G37480,AT4G36040,AT2G02515,AT4G10120,AT1G01230,AT5G05710,AT1G12860,AT1G36830,AT1G74920,AT5G03510,AT2G47390,AT3G44720,AT1G52230,AT3G15690,AT1G79370,AT3G16040,AT1G30270
AGAAGATTCT	10	4	['0-4', '4-8', '8-12', '16-20', '20-24']	22	AT2G45980,AT2G05820,AT5G37960,AT5G49120,AT5G48100,AT4G37480,AT4G36040,AT2G02515,AT4G10120,AT1G01230,AT5G05710,AT1G12860,AT1G36830,AT1G74920,AT5G03510,AT2G47390,AT3G44720,AT1G52230,AT3G15690,AT1G79370,AT3G16040,AT1G30270
AGAAGATTCT	10	5	['4-8', '8-12', '16-20', '20-24', '0-4']	22	AT2G45980,AT2G05820,AT5G37960,AT5G49120,AT5G48100,AT4G37480,AT4G36040,AT2G02515,AT4G10120,AT1G01230,AT5G05710,AT1G12860,AT1G36830,AT1G74920,AT5G03510,AT2G47390,AT3G44720,AT1G52230,AT3G15690,AT1G79370,AT3G16040,AT1G30270
AGAAGCTTCT	10	1	['0-4', '16-20', '20-24']	13	AT5G60850,AT1G16880,AT4G39030,AT3G26660,AT2G04039,AT5G11270,AT3G23660,AT4G22750,AT5G66060,AT4G38470,AT2G18170,AT2G20170,AT4G34500
AGAAGCTTCT	10	2	['0-4', '16-20']	13	AT5G60850,AT1G16880,AT4G39030,AT3G26660,AT2G04039,AT5G11270,AT3G23660,AT4G22750,AT5G66060,AT4G38470,AT2G18170,AT2G20170,AT4G34500
AGAAGCTTCT	10	3	['0-4', '16-20', '20-24']	13	AT5G60850,AT1G16880,AT4G39030,AT3G26660,AT2G04039,AT5G11270,AT3G23660,AT4G22750,AT5G66060,AT4G38470,AT2G18170,AT2G20170,AT4G34500
AGAAGCTTCT	10	4	['0-4', '16-20', '20-24']	13	AT5G60850,AT1G16880,AT4G39030,AT3G26660,AT2G04039,AT5G11270,AT3G23660,AT4G22750,AT5G66060,AT4G38470,AT2G18170,AT2G20170,AT4G34500
AGAAGCTTCT	10	5	['16-20', '20-24', '0-4']	13	AT5G60850,AT1G16880,AT4G39030,AT3G26660,AT2G04039,AT5G11270,AT3G23660,AT4G22750,AT5G66060,AT4G38470,AT2G18170,AT2G20170,AT4G34500
AGAAGGTTCT	10	1	['0-4', '8-12', '12-16', '20-24']	12	AT1G54830,AT2G46490,AT3G26740,AT1G76580,AT1G67470,AT4G38690,AT1G74880,AT1G01520,AT3G12800,AT4G37220,AT1G31812,AT5G01090
AGAAGGTTCT	10	2	['0-4', '8-12', '20-24', '12-16']	12	AT1G54830,AT2G46490,AT3G26740,AT1G76580,AT1G67470,AT4G38690,AT1G74880,AT1G01520,AT3G12800,AT4G37220,AT1G31812,AT5G01090
AGAAGGTTCT	10	3	['0-4', '8-12', '12-16', '20-24']	12	AT1G54830,AT2G46490,AT3G26740,AT1G76580,AT1G67470,AT4G38690,AT1G74880,AT1G01520,AT3G12800,AT4G37220,AT1G31812,AT5G01090
AGAAGGTTCT	10	4	['0-4', '8-12', '12-16', '20-24']	12	AT1G54830,AT2G46490,AT3G26740,AT1G76580,AT1G67470,AT4G38690,AT1G74880,AT1G01520,AT3G12800,AT4G37220,AT1G31812,AT5G01090
AGAAGGTTCT	10	5	['0-4', '8-12', '12-16', '20-24']	12	AT1G54830,AT2G46490,AT3G26740,AT1G76580,AT1G67470,AT4G38690,AT1G74880,AT1G01520,AT3G12800,AT4G37220,AT1G31812,AT5G01090
AGAAGTTTCT	10	1	['0-4', '8-12', '16-20', '20-24', '4-8']	18	AT5G15230,AT3G59400,AT2G41720,AT3G61210,AT1G73060,AT1G34440,AT1G66080,AT4G20360,AT3G31320,AT3G45210,AT2G46650,AT1G74940,AT5G14320,AT5G01810,AT1G17455,AT5G64170,AT1G16750,AT5G08590
AGAAGTTTCT	10	2	['0-4', '4-8', '8-12', '16-20', '20-24']	18	AT5G15230,AT3G59400,AT2G41720,AT3G61210,AT1G73060,AT1G34440,AT1G66080,AT4G20360,AT3G31320,AT3G45210,AT2G46650,AT1G74940,AT5G14320,AT5G01810,AT1G17455,AT5G64170,AT1G16750,AT5G08590
AGAAGTTTCT	10	3	['0-4', '4-8', '8-12', '16-20', '20-24']	18	AT5G15230,AT3G59400,AT2G41720,AT3G61210,AT1G73060,AT1G34440,AT1G66080,AT4G20360,AT3G31320,AT3G45210,AT2G46650,AT1G74940,AT5G14320,AT5G01810,AT1G17455,AT5G64170,AT1G16750,AT5G08590
AGAAGTTTCT	10	4	['0-4', '8-12', '16-20', '20-24', '4-8']	18	AT5G15230,AT3G59400,AT2G41720,AT3G61210,AT1G73060,AT1G34440,AT1G66080,AT4G20360,AT3G31320,AT3G45210,AT2G46650,AT1G74940,AT5G14320,AT5G01810,AT1G17455,AT5G64170,AT1G16750,AT5G08590
AGAAGTTTCT	10	5	['0-4', '4-8', '8-12', '16-20', '20-24']	18	AT5G15230,AT3G59400,AT2G41720,AT3G61210,AT1G73060,AT1G34440,AT1G66080,AT4G20360,AT3G31320,AT3G45210,AT2G46650,AT1G74940,AT5G14320,AT5G01810,AT1G17455,AT5G64170,AT1G16750,AT5G08590

AGAATATTCT	10	1	['4-8', '8-12', '16-20', '20-24']	17	AT3G05500,AT5G20280,AT1G08550,AT3G03770,AT2G43140,AT3G47860,AT2G18670,AT1G66980,AT1G34440,AT4G24470,AT3G45210,AT1G54880,AT2G46650,AT1G09430,AT5G56170,AT1G04620,AT1G65190
AGAATATTCT	10	2	['4-8', '8-12', '16-20', '20-24']	17	AT3G05500,AT5G20280,AT1G08550,AT3G03770,AT2G43140,AT3G47860,AT2G18670,AT1G66980,AT1G34440,AT4G24470,AT3G45210,AT1G54880,AT2G46650,AT1G09430,AT5G56170,AT1G04620,AT1G65190
AGAATATTCT	10	3	['4-8', '8-12', '20-24', '16-20']	17	AT3G05500,AT5G20280,AT1G08550,AT3G03770,AT2G43140,AT3G47860,AT2G18670,AT1G66980,AT1G34440,AT4G24470,AT3G45210,AT1G54880,AT2G46650,AT1G09430,AT5G56170,AT1G04620,AT1G65190
AGAATATTCT	10	4	['4-8', '8-12', '16-20', '20-24']	17	AT3G05500,AT5G20280,AT1G08550,AT3G03770,AT2G43140,AT3G47860,AT2G18670,AT1G66980,AT1G34440,AT4G24470,AT3G45210,AT1G54880,AT2G46650,AT1G09430,AT5G56170,AT1G04620,AT1G65190
AGAATATTCT	10	5	['4-8', '8-12', '16-20', '20-24']	17	AT3G05500,AT5G20280,AT1G08550,AT3G03770,AT2G43140,AT3G47860,AT2G18670,AT1G66980,AT1G34440,AT4G24470,AT3G45210,AT1G54880,AT2G46650,AT1G09430,AT5G56170,AT1G04620,AT1G65190
AGAATCTTCT	10	1	['0-4', '4-8', '8-12', '12-16']	18	AT4G34590,AT5G19250,AT5G67390,AT2G43730,AT2G47400,AT1G24440,AT5G49950,AT2G39400,AT1G52870,AT2G43840,AT4G30660,AT1G52220,AT3G02590,AT3G60020,AT5G49230,AT5G35180,AT5G52120,AT5G40450
AGAATCTTCT	10	2	['0-4', '4-8', '8-12', '12-16']	18	AT4G34590,AT5G19250,AT5G67390,AT2G43730,AT2G47400,AT1G24440,AT5G49950,AT2G39400,AT1G52870,AT2G43840,AT4G30660,AT1G52220,AT3G02590,AT3G60020,AT5G49230,AT5G35180,AT5G52120,AT5G40450
AGAATCTTCT	10	3	['0-4', '4-8', '8-12', '12-16']	18	AT4G34590,AT5G19250,AT5G67390,AT2G43730,AT2G47400,AT1G24440,AT5G49950,AT2G39400,AT1G52870,AT2G43840,AT4G30660,AT1G52220,AT3G02590,AT3G60020,AT5G49230,AT5G35180,AT5G52120,AT5G40450
AGAATCTTCT	10	4	['0-4', '4-8', '8-12', '12-16']	18	AT4G34590,AT5G19250,AT5G67390,AT2G43730,AT2G47400,AT1G24440,AT5G49950,AT2G39400,AT1G52870,AT2G43840,AT4G30660,AT1G52220,AT3G02590,AT3G60020,AT5G49230,AT5G35180,AT5G52120,AT5G40450
AGAATCTTCT	10	5	['0-4', '4-8', '8-12', '12-16']	18	AT4G34590,AT5G19250,AT5G67390,AT2G43730,AT2G47400,AT1G24440,AT5G49950,AT2G39400,AT1G52870,AT2G43840,AT4G30660,AT1G52220,AT3G02590,AT3G60020,AT5G49230,AT5G35180,AT5G52120,AT5G40450
AGAATGTTCT	10	1	['4-8', '8-12', '12-16', '16-20', '20-24']	16	AT3G59052,AT5G48230,AT2G02040,AT5G39570,AT4G37680,AT3G52240,AT1G76280,AT2G30860,AT2G47320,AT2G44770,AT1G53540,AT5G18170,AT3G46610,AT4G27670,AT3G52290,AT2G30070
AGAATGTTCT	10	2	['4-8', '8-12', '12-16', '16-20', '20-24']	16	AT3G59052,AT5G48230,AT2G02040,AT5G39570,AT4G37680,AT3G52240,AT1G76280,AT2G30860,AT2G47320,AT2G44770,AT1G53540,AT5G18170,AT3G46610,AT4G27670,AT3G52290,AT2G30070
AGAATGTTCT	10	3	['4-8', '8-12', '12-16', '16-20', '20-24']	16	AT3G59052,AT5G48230,AT2G02040,AT5G39570,AT4G37680,AT3G52240,AT1G76280,AT2G30860,AT2G47320,AT2G44770,AT1G53540,AT5G18170,AT3G46610,AT4G27670,AT3G52290,AT2G30070
AGAATGTTCT	10	4	['4-8', '8-12', '12-16', '16-20', '20-24']	16	AT3G59052,AT5G48230,AT2G02040,AT5G39570,AT4G37680,AT3G52240,AT1G76280,AT2G30860,AT2G47320,AT2G44770,AT1G53540,AT5G18170,AT3G46610,AT4G27670,AT3G52290,AT2G30070
AGAATGTTCT	10	5	['4-8', '8-12', '12-16', '16-20', '20-24']	16	AT3G59052,AT5G48230,AT2G02040,AT5G39570,AT4G37680,AT3G52240,AT1G76280,AT2G30860,AT2G47320,AT2G44770,AT1G53540,AT5G18170,AT3G46610,AT4G27670,AT3G52290,AT2G30070
AGAATTTTCT	10	1	['4-8', '8-12', '12-16']	37	AT1G75140,AT1G22770,AT1G48060,AT5G10530,AT5G25060,AT1G62750,AT1G73660,AT3G27090,AT5G47890,AT1G27440,AT4G17070,AT5G66530,AT3G49220,AT2G16710,AT1G64780,AT5G05520,AT1G19650,AT5G64630,AT3G21210,AT2G15570,AT1G78020,AT1G20440,AT4G23200,AT4G22300,AT3G46670,AT5G42920,AT1G78600,AT1G03550,AT2G19810,AT2G39710,AT4G02480,AT3G15630,AT5G35220,AT3G44250,AT3G15690,AT4G01940,ATMG01180
AGAATTTTCT	10	2	['4-8', '8-12']	37	AT1G75140,AT1G22770,AT1G48060,AT5G10530,AT5G25060,AT1G62750,AT1G73660,AT3G27090,AT5G47890,AT1G27440,AT4G17070,AT5G66530,AT3G49220,AT2G16710,AT1G64780,AT5G05520,AT1G19650,AT5G64630,AT3G21210,AT2G15570,AT1G78020,AT1G20440,AT4G23200,AT4G22300,AT3G46670,AT5G42920,AT1G78600,AT1G03550,AT2G19810,AT2G39710,AT4G02480,AT3G15630,AT5G35220,AT3G44250,AT3G15690,AT4G01940,ATMG01180

AGAATTTTCT	10	3	['4-8', '8-12']	37	AT1G75140,AT1G22770,AT1G48060,AT5G10530,AT5G25060,AT1G62750,AT1G73660,AT3G27090,AT5G47890,AT1G27440,AT4G17070,AT5G66530,AT3G49220,AT2G16710,AT1G64780,AT5G05520,AT1G19650,AT5G64630,AT3G21210,AT2G15570,AT1G78020,AT1G20440,AT4G23200,AT4G22300,AT3G46670,AT5G42920,AT1G78600,AT1G03550,AT2G19810,AT2G39710,AT4G02480,AT3G15630,AT5G35220,AT3G44250,AT3G15690,AT4G01940,ATMG01180
AGAATTTTCT	10	4	['8-12', '4-8']	37	AT1G75140,AT1G22770,AT1G48060,AT5G10530,AT5G25060,AT1G62750,AT1G73660,AT3G27090,AT5G47890,AT1G27440,AT4G17070,AT5G66530,AT3G49220,AT2G16710,AT1G64780,AT5G05520,AT1G19650,AT5G64630,AT3G21210,AT2G15570,AT1G78020,AT1G20440,AT4G23200,AT4G22300,AT3G46670,AT5G42920,AT1G78600,AT1G03550,AT2G19810,AT2G39710,AT4G02480,AT3G15630,AT5G35220,AT3G44250,AT3G15690,AT4G01940,ATMG01180
AGAATTTTCT	10	5	['4-8', '8-12', '12-16']	37	AT1G75140,AT1G22770,AT1G48060,AT5G10530,AT5G25060,AT1G62750,AT1G73660,AT3G27090,AT5G47890,AT1G27440,AT4G17070,AT5G66530,AT3G49220,AT2G16710,AT1G64780,AT5G05520,AT1G19650,AT5G64630,AT3G21210,AT2G15570,AT1G78020,AT1G20440,AT4G23200,AT4G22300,AT3G46670,AT5G42920,AT1G78600,AT1G03550,AT2G19810,AT2G39710,AT4G02480,AT3G15630,AT5G35220,AT3G44250,AT3G15690,AT4G01940,ATMG01180
AGACCGTTG	10	1	['0-4', '4-8', '8-12', '12-16', '16-20', '20-24']	13	AT1G54830,AT1G01700,AT5G11740,AT2G23880,AT4G11410,AT2G01290,AT2G25250,AT1G74880,AT5G23760,AT2G29670,AT1G68820,AT4G15840,AT2G15040
AGACCGTTG	10	2	['0-4', '4-8', '8-12', '12-16', '16-20', '20-24']	13	AT1G54830,AT1G01700,AT5G11740,AT2G23880,AT4G11410,AT2G01290,AT2G25250,AT1G74880,AT5G23760,AT2G29670,AT1G68820,AT4G15840,AT2G15040
AGACCGTTG	10	3	['0-4', '4-8', '8-12', '12-16', '16-20', '20-24']	13	AT1G54830,AT1G01700,AT5G11740,AT2G23880,AT4G11410,AT2G01290,AT2G25250,AT1G74880,AT5G23760,AT2G29670,AT1G68820,AT4G15840,AT2G15040
AGACCGTTG	10	4	['0-4', '4-8', '8-12', '12-16', '16-20', '20-24']	13	AT1G54830,AT1G01700,AT5G11740,AT2G23880,AT4G11410,AT2G01290,AT2G25250,AT1G74880,AT5G23760,AT2G29670,AT1G68820,AT4G15840,AT2G15040
AGACCGTTG	10	5	['4-8', '8-12', '12-16', '16-20', '20-24', '0-4']	13	AT1G54830,AT1G01700,AT5G11740,AT2G23880,AT4G11410,AT2G01290,AT2G25250,AT1G74880,AT5G23760,AT2G29670,AT1G68820,AT4G15840,AT2G15040
AGATCGACG	10	1	['0-4', '4-8', '20-24']	10	AT2G34770,AT3G19400,AT4G27030,AT5G42310,AT1G62620,AT3G15580,AT4G38420,AT4G15140,AT1G15710,AT3G12470
AGATCGACG	10	2	['0-4', '4-8', '20-24']	10	AT2G34770,AT3G19400,AT4G27030,AT5G42310,AT1G62620,AT3G15580,AT4G38420,AT4G15140,AT1G15710,AT3G12470
AGATCGACG	10	3	['0-4', '4-8', '20-24']	10	AT2G34770,AT3G19400,AT4G27030,AT5G42310,AT1G62620,AT3G15580,AT4G38420,AT4G15140,AT1G15710,AT3G12470
AGATCGACG	10	4	['0-4', '4-8', '20-24']	10	AT2G34770,AT3G19400,AT4G27030,AT5G42310,AT1G62620,AT3G15580,AT4G38420,AT4G15140,AT1G15710,AT3G12470
AGATCGACG	10	5	['0-4', '4-8', '20-24']	10	AT2G34770,AT3G19400,AT4G27030,AT5G42310,AT1G62620,AT3G15580,AT4G38420,AT4G15140,AT1G15710,AT3G12470
AGCCGACAA	10	1	['12-16', '16-20', '20-24']	14	AT5G27290,AT1G18040,AT5G57630,AT1G14140,AT3G23160,AT1G01240,AT2G32480,AT1G61100,AT4G38770,AT1G74750,AT5G17910,AT4G36460,AT1G08540,AT5G51140
AGCCGACAA	10	2	['12-16', '16-20', '20-24']	14	AT5G27290,AT1G18040,AT5G57630,AT1G14140,AT3G23160,AT1G01240,AT2G32480,AT1G61100,AT4G38770,AT1G74750,AT5G17910,AT4G36460,AT1G08540,AT5G51140
AGCCGACAA	10	3	['12-16', '16-20', '20-24']	14	AT5G27290,AT1G18040,AT5G57630,AT1G14140,AT3G23160,AT1G01240,AT2G32480,AT1G61100,AT4G38770,AT1G74750,AT5G17910,AT4G36460,AT1G08540,AT5G51140

AGCCGACAA	10	4	['12-16', '16-20', '20-24']	14	AT5G27290,AT1G18040,AT5G57630,AT1G14140,AT3G23160,AT1G01240,AT2G32480,AT1G61100,AT4G38770,AT1G74750,AT5G17910,AT4G36460,AT1G08540,AT5G51140
AGCCGACAA	10	5	['12-16', '16-20', '20-24']	14	AT5G27290,AT1G18040,AT5G57630,AT1G14140,AT3G23160,AT1G01240,AT2G32480,AT1G61100,AT4G38770,AT1G74750,AT5G17910,AT4G36460,AT1G08540,AT5G51140
AGCCGACAT	10	1	['0-4', '4-8', '8-12', '16-20', '20-24']	13	AT4G16515,AT2G15320,AT1G08890,AT5G08000,AT1G33270,AT5G66710,AT3G24170,AT1G53440,AT2G36320,AT2G41410,AT4G16140,AT3G15580,AT2G23760
AGCCGACAT	10	2	['0-4', '4-8', '8-12', '16-20']	13	AT4G16515,AT2G15320,AT1G08890,AT5G08000,AT1G33270,AT5G66710,AT3G24170,AT1G53440,AT2G36320,AT2G41410,AT4G16140,AT3G15580,AT2G23760
AGCCGACAT	10	3	['0-4', '4-8', '8-12', '16-20', '20-24']	13	AT4G16515,AT2G15320,AT1G08890,AT5G08000,AT1G33270,AT5G66710,AT3G24170,AT1G53440,AT2G36320,AT2G41410,AT4G16140,AT3G15580,AT2G23760
AGCCGACAT	10	4	['0-4', '4-8', '8-12', '16-20', '20-24']	13	AT4G16515,AT2G15320,AT1G08890,AT5G08000,AT1G33270,AT5G66710,AT3G24170,AT1G53440,AT2G36320,AT2G41410,AT4G16140,AT3G15580,AT2G23760
AGCCGACAT	10	5	['4-8', '8-12', '16-20', '20-24', '0-4']	13	AT4G16515,AT2G15320,AT1G08890,AT5G08000,AT1G33270,AT5G66710,AT3G24170,AT1G53440,AT2G36320,AT2G41410,AT4G16140,AT3G15580,AT2G23760
AGCCGACTT	10	1	['0-4', '4-8', '16-20', '20-24']	12	AT2G37240,AT2G34040,AT2G01980,AT2G07738,AT1G52880,AT3G03160,AT3G10050,AT1G02160,AT4G19340,AT3G15510,AT2G45990,AT5G61530
AGCCGACTT	10	2	['0-4', '4-8', '16-20', '20-24']	12	AT2G37240,AT2G34040,AT2G01980,AT2G07738,AT1G52880,AT3G03160,AT3G10050,AT1G02160,AT4G19340,AT3G15510,AT2G45990,AT5G61530
AGCCGACTT	10	3	['0-4', '4-8', '16-20', '20-24']	12	AT2G37240,AT2G34040,AT2G01980,AT2G07738,AT1G52880,AT3G03160,AT3G10050,AT1G02160,AT4G19340,AT3G15510,AT2G45990,AT5G61530
AGCCGACTT	10	4	['0-4', '4-8', '16-20', '20-24']	12	AT2G37240,AT2G34040,AT2G01980,AT2G07738,AT1G52880,AT3G03160,AT3G10050,AT1G02160,AT4G19340,AT3G15510,AT2G45990,AT5G61530
AGCCGACTT	10	5	['0-4', '4-8', '16-20', '20-24']	12	AT2G37240,AT2G34040,AT2G01980,AT2G07738,AT1G52880,AT3G03160,AT3G10050,AT1G02160,AT4G19340,AT3G15510,AT2G45990,AT5G61530
AGCCGCC	10	1	['16-20']	81	AT2G07730,AT5G60550,AT1G07940,AT5G11600,AT1G01970,AT4G28210,AT3G13860,AT4G00370,AT3G26580,AT3G01510,AT3G02590,AT5G49280,AT3G52070,AT3G52060,AT3G07640,AT1G14040,AT2G37970,AT3G04210,AT5G58730,AT1G73820,AT5G58260,AT1G16470,AT5G35735,AT1G61700,AT3G09820,AT3G02930,AT1G71740,AT1G44510,AT4G35800,AT1G20840,AT2G23060,AT3G17000,AT1G07140,AT1G60000,AT1G07010,AT1G01250,AT3G04880,AT1G74270,AT1G75330,AT1G08890,AT3G13470,AT1G34440,AT5G55960,AT5G18525,AT1G79470,AT5G04590,AT3G03150,AT4G33480,AT3G57280,AT2G46240,AT4G16870,AT2G22030,AT1G62180,AT3G03180,AT3G10050,AT3G18215,AT4G15810,AT3G06470,AT1G77570,AT5G43430,AT5G15710,AT3G46340,AT1G71180,AT1G03530,AT2G22360,AT5G53280,AT1G35900,AT5G14730,AT5G35380,AT3G44950,AT4G02195,AT1G55850,AT3G54890,AT3G06160,AT1G72300,AT5G51460,AT5G01410,AT1G21680,AT1G62430,AT3G04790,AT1G01790
AGCCGCC	10	2	['16-20']	81	AT2G07730,AT5G60550,AT1G07940,AT5G11600,AT1G01970,AT4G28210,AT3G13860,AT4G00370,AT3G26580,AT3G01510,AT3G02590,AT5G49280,AT3G52070,AT3G52060,AT3G07640,AT1G14040,AT2G37970,AT3G04210,AT5G58730,AT1G73820,AT5G58260,AT1G16470,AT5G35735,AT1G61700,AT3G09820,AT3G02930,AT1G71740,AT1G44510,AT4G35800,AT1G20840,AT2G23060,AT3G17000,AT1G07140,AT1G60000,AT1G07010,AT1G01250,AT3G04880,AT1G74270,AT1G75330,AT1G08890,AT3G13470,AT1G34440,AT5G55960,AT5G18525,AT1G79470,AT5G04590,AT3G03150,AT4G33480,AT3G57280,AT2G46240,AT4G16870,AT2G22030,AT1G62180,AT3G03180,AT3G10050,AT3G18215,AT4G15810,AT3G06470,AT1G77570,AT5G43430,AT5G15710,AT3G46340,AT1G71180,AT1G03530,AT2G22360,AT5G53280,AT1G35900,AT5G14730,AT5G35380,AT3G44950,AT4G02195,AT1G55850,AT3G54890,AT3G06160,AT1G72300,AT5G51460,AT5G01410,AT1G21680,AT1G62430,AT3G04790,AT1G01790

AGCCGCC	10	3	['16-20']	81	AT2G07730,AT5G60550,AT1G07940,AT5G11600,AT1G01970,AT4G28210,AT3G13860,AT4G00370,AT3G26580,AT3G01510,AT3G02590,AT5G49280,AT3G52070,AT3G52060,AT3G07640,AT1G14040,AT2G37970,AT3G04210,AT5G58730,AT1G73820,AT5G58260,AT1G16470,AT5G35735,AT1G61700,AT3G09820,AT3G02930,AT1G71740,AT1G44510,AT4G35800,AT1G20840,AT2G23060,AT3G17000,AT1G07140,AT1G60000,AT1G07010,AT1G01250,AT3G04880,AT1G74270,AT1G75330,AT1G08890,AT3G13470,AT1G34440,AT5G55960,AT5G18525,AT1G79470,AT5G04590,AT3G03150,AT4G33480,AT3G57280,AT2G46240,AT4G16870,AT2G22030,AT1G62180,AT3G03180,AT3G10050,AT3G18215,AT4G15810,AT3G06470,AT1G77570,AT5G43430,AT5G15710,AT3G46340,AT1G71180,AT1G03530,AT2G22360,AT5G53280,AT1G35900,AT5G14730,AT5G35380,AT3G44950,AT4G02195,AT1G55850,AT3G54890,AT3G06160,AT1G72300,AT5G51460,AT5G01410,AT1G21680,AT1G62430,AT3G04790,AT1G01790
AGCCGCC	10	4	['16-20']	81	AT2G07730,AT5G60550,AT1G07940,AT5G11600,AT1G01970,AT4G28210,AT3G13860,AT4G00370,AT3G26580,AT3G01510,AT3G02590,AT5G49280,AT3G52070,AT3G52060,AT3G07640,AT1G14040,AT2G37970,AT3G04210,AT5G58730,AT1G73820,AT5G58260,AT1G16470,AT5G35735,AT1G61700,AT3G09820,AT3G02930,AT1G71740,AT1G44510,AT4G35800,AT1G20840,AT2G23060,AT3G17000,AT1G07140,AT1G60000,AT1G07010,AT1G01250,AT3G04880,AT1G74270,AT1G75330,AT1G08890,AT3G13470,AT1G34440,AT5G55960,AT5G18525,AT1G79470,AT5G04590,AT3G03150,AT4G33480,AT3G57280,AT2G46240,AT4G16870,AT2G22030,AT1G62180,AT3G03180,AT3G10050,AT3G18215,AT4G15810,AT3G06470,AT1G77570,AT5G43430,AT5G15710,AT3G46340,AT1G71180,AT1G03530,AT2G22360,AT5G53280,AT1G35900,AT5G14730,AT5G35380,AT3G44950,AT4G02195,AT1G55850,AT3G54890,AT3G06160,AT1G72300,AT5G51460,AT5G01410,AT1G21680,AT1G62430,AT3G04790,AT1G01790
AGCCGCC	10	5	['16-20']	81	AT2G07730,AT5G60550,AT1G07940,AT5G11600,AT1G01970,AT4G28210,AT3G13860,AT4G00370,AT3G26580,AT3G01510,AT3G02590,AT5G49280,AT3G52070,AT3G52060,AT3G07640,AT1G14040,AT2G37970,AT3G04210,AT5G58730,AT1G73820,AT5G58260,AT1G16470,AT5G35735,AT1G61700,AT3G09820,AT3G02930,AT1G71740,AT1G44510,AT4G35800,AT1G20840,AT2G23060,AT3G17000,AT1G07140,AT1G60000,AT1G07010,AT1G01250,AT3G04880,AT1G74270,AT1G75330,AT1G08890,AT3G13470,AT1G34440,AT5G55960,AT5G18525,AT1G79470,AT5G04590,AT3G03150,AT4G33480,AT3G57280,AT2G46240,AT4G16870,AT2G22030,AT1G62180,AT3G03180,AT3G10050,AT3G18215,AT4G15810,AT3G06470,AT1G77570,AT5G43430,AT5G15710,AT3G46340,AT1G71180,AT1G03530,AT2G22360,AT5G53280,AT1G35900,AT5G14730,AT5G35380,AT3G44950,AT4G02195,AT1G55850,AT3G54890,AT3G06160,AT1G72300,AT5G51460,AT5G01410,AT1G21680,AT1G62430,AT3G04790,AT1G01790
AGCGGG	10	1	['16-20']	150	ATMG00650,AT1G31270,AT3G05480,AT3G53800,AT2G26520,AT3G01510,AT3G52150,AT5G49450,AT1G14040,AT5G12480,AT2G02280,AT1G66980,AT1G22360,AT3G50860,AT5G46690,AT3G48460,AT2G47780,AT3G47450,AT1G21270,AT4G16860,AT2G35500,AT1G75690,AT3G47130,AT4G16190,AT1G58200,AT3G11250,AT3G11200,AT3G43670,AT1G33490,AT2G31450,AT1G76730,AT3G43020,AT2G38820,AT1G32550,AT4G39710,AT4G30260,AT2G13360,AT1G76550,AT1G66100,AT5G39080,AT5G38480,AT5G19860,AT2G38410,AT1G73760,AT4G38160,AT5G19290,AT5G35560,AT5G20380,AT4G37320,AT3G25690,AT1G79910,AT1G70730,AT3G27980,AT2G19270,AT2G36880,AT1G80310,AT3G28580,AT5G18590,AT3G15290,AT4G33700,AT2G36060,AT5G17310,AT4G32410,AT5G16715,AT4G32530,AT2G42670,AT4G31820,AT5G16430,AT2G22360,AT3G14900,AT4G31310,AT2G43550,AT2G42160,AT2G22450,AT5G14240,AT2G28000,AT1G53210,AT3G14450,AT5G34830,ATMG01180,AT4G29700,AT1G32360,AT5G27010,AT5G26770,AT5G11480,AT1G09160,AT4G27330,AT5G09650,AT3G12920,AT5G66770,AT1G53450,AT5G06790,AT1G09570,AT5G66180,AT5G06460,AT3G20810,AT1G04850,AT1G12960,AT1G12845,AT4G23290,AT3G23660,AT5G63420,AT5G02810,AT1G79730,AT5G63050,AT5G02880,AT3G14000,AT5G02760,AT5G62090,AT4G19530,AT4G19110,AT1G21065,AT1G14280,AT5G01810,AT5G61820,AT1G14350,AT1G71695,AT5G01650,AT3G63160,AT1G01970,AT3G62280,AT3G61220,AT5G58760,AT5G58380,ATMG00610,AT5G58140,AT3G60810,AT3G08570,AT2G28390,AT4G10750,AT4G10360,AT4G08320,AT3G58120,AT3G07170,AT2G05520,AT2G05620,AT1G53840,AT4G04340,AT5G54500,AT3G57250,AT5G54080,AT2G24490,AT4G03260,AT1G49270,AT2G01290,AT5G52390,AT2G35690,AT3G03320,AT5G51840,AT3G07550

AGCGGG	10	2	['16-20']	150	ATMG00650,AT1G31270,AT3G05480,AT3G53800,AT2G26520,AT3G01510,AT3G52150,AT5G49450,AT1G14040,AT5G12480,AT2G02280,AT1G66980,AT1G22360,AT3G50860,AT5G46690,AT3G48460,AT2G47780,AT3G47450,AT1G21270,AT4G16860,AT2G35500,AT1G75690,AT3G47130,AT4G16190,AT1G58200,AT3G11250,AT3G11200,AT3G43670,AT1G33490,AT2G31450,AT1G76730,AT3G43020,AT2G38820,AT1G32550,AT4G39710,AT4G30260,AT2G13360,AT1G76550,AT1G66100,AT5G39080,AT5G38480,AT5G19860,AT2G38410,AT1G73760,AT4G38160,AT5G19290,AT5G35560,AT5G20380,AT4G37320,AT3G25690,AT1G79910,AT1G70730,AT3G27980,AT2G19270,AT2G36880,AT1G80310,AT3G28580,AT5G18590,AT3G15290,AT4G33700,AT2G36060,AT5G17310,AT4G32410,AT5G16715,AT4G32530,AT2G42670,AT4G31820,AT5G16430,AT2G22360,AT3G14900,AT4G31310,AT2G43550,AT2G42160,AT2G22450,AT5G14240,AT2G28000,AT1G53210,AT3G14450,AT5G34830,ATMG01180,AT4G29700,AT1G32360,AT5G27010,AT5G26770,AT5G11480,AT1G09160,AT4G27330,AT5G09650,AT3G12920,AT5G66770,AT1G53450,AT5G06790,AT1G09570,AT5G66180,AT5G06460,AT3G20810,AT1G04850,AT1G12960,AT1G12845,AT4G23290,AT3G23660,AT5G63420,AT5G02810,AT1G79730,AT5G63050,AT5G02880,AT3G14000,AT5G02760,AT5G62090,AT4G19530,AT4G19110,AT1G21065,AT1G14280,AT5G01810,AT5G61820,AT1G14350,AT1G71695,AT5G01650,AT3G63160,AT1G01970,AT3G62280,AT3G61220,AT5G58760,AT5G58380,ATMG00610,AT5G58140,AT3G60810,AT3G08570,AT2G28390,AT4G10750,AT4G10360,AT4G08320,AT3G58120,AT3G07170,AT2G05520,AT2G05620,AT1G53840,AT4G04340,AT5G54500,AT3G57250,AT5G54080,AT2G24490,AT4G03260,AT1G49270,AT2G01290,AT5G52390,AT2G35690,AT3G03320,AT5G51840,AT3G07550
AGCGGG	10	3	['16-20']	150	ATMG00650,AT1G31270,AT3G05480,AT3G53800,AT2G26520,AT3G01510,AT3G52150,AT5G49450,AT1G14040,AT5G12480,AT2G02280,AT1G66980,AT1G22360,AT3G50860,AT5G46690,AT3G48460,AT2G47780,AT3G47450,AT1G21270,AT4G16860,AT2G35500,AT1G75690,AT3G47130,AT4G16190,AT1G58200,AT3G11250,AT3G11200,AT3G43670,AT1G33490,AT2G31450,AT1G76730,AT3G43020,AT2G38820,AT1G32550,AT4G39710,AT4G30260,AT2G13360,AT1G76550,AT1G66100,AT5G39080,AT5G38480,AT5G19860,AT2G38410,AT1G73760,AT4G38160,AT5G19290,AT5G35560,AT5G20380,AT4G37320,AT3G25690,AT1G79910,AT1G70730,AT3G27980,AT2G19270,AT2G36880,AT1G80310,AT3G28580,AT5G18590,AT3G15290,AT4G33700,AT2G36060,AT5G17310,AT4G32410,AT5G16715,AT4G32530,AT2G42670,AT4G31820,AT5G16430,AT2G22360,AT3G14900,AT4G31310,AT2G43550,AT2G42160,AT2G22450,AT5G14240,AT2G28000,AT1G53210,AT3G14450,AT5G34830,ATMG01180,AT4G29700,AT1G32360,AT5G27010,AT5G26770,AT5G11480,AT1G09160,AT4G27330,AT5G09650,AT3G12920,AT5G66770,AT1G53450,AT5G06790,AT1G09570,AT5G66180,AT5G06460,AT3G20810,AT1G04850,AT1G12960,AT1G12845,AT4G23290,AT3G23660,AT5G63420,AT5G02810,AT1G79730,AT5G63050,AT5G02880,AT3G14000,AT5G02760,AT5G62090,AT4G19530,AT4G19110,AT1G21065,AT1G14280,AT5G01810,AT5G61820,AT1G14350,AT1G71695,AT5G01650,AT3G63160,AT1G01970,AT3G62280,AT3G61220,AT5G58760,AT5G58380,ATMG00610,AT5G58140,AT3G60810,AT3G08570,AT2G28390,AT4G10750,AT4G10360,AT4G08320,AT3G58120,AT3G07170,AT2G05520,AT2G05620,AT1G53840,AT4G04340,AT5G54500,AT3G57250,AT5G54080,AT2G24490,AT4G03260,AT1G49270,AT2G01290,AT5G52390,AT2G35690,AT3G03320,AT5G51840,AT3G07550

AGCGGG	10	4	['16-20']	150	ATMG00650,AT1G31270,AT3G05480,AT3G53800,AT2G26520,AT3G01510,AT3G52150,AT5G49450,AT1G14040,AT5G12480,AT2G02280,AT1G66980,AT1G22360,AT3G50860,AT5G46690,AT3G48460,AT2G47780,AT3G47450,AT1G21270,AT4G16860,AT2G35500,AT1G75690,AT3G47130,AT4G16190,AT1G58200,AT3G11250,AT3G11200,AT3G43670,AT1G33490,AT2G31450,AT1G76730,AT3G43020,AT2G38820,AT1G32550,AT4G39710,AT4G30260,AT2G13360,AT1G76550,AT1G66100,AT5G39080,AT5G38480,AT5G19860,AT2G38410,AT1G73760,AT4G38160,AT5G19290,AT5G35560,AT5G20380,AT4G37320,AT3G25690,AT1G79910,AT1G70730,AT3G27980,AT2G19270,AT2G36880,AT1G80310,AT3G28580,AT5G18590,AT3G15290,AT4G33700,AT2G36060,AT5G17310,AT4G32410,AT5G16715,AT4G32530,AT2G42670,AT4G31820,AT5G16430,AT2G22360,AT3G14900,AT4G31310,AT2G43550,AT2G42160,AT2G22450,AT5G14240,AT2G28000,AT1G53210,AT3G14450,AT5G34830,ATMG01180,AT4G29700,AT1G32360,AT5G27010,AT5G26770,AT5G11480,AT1G09160,AT4G27330,AT5G09650,AT3G12920,AT5G66770,AT1G53450,AT5G06790,AT1G09570,AT5G66180,AT5G06460,AT3G20810,AT1G04850,AT1G12960,AT1G12845,AT4G23290,AT3G23660,AT5G63420,AT5G02810,AT1G79730,AT5G63050,AT5G02880,AT3G14000,AT5G02760,AT5G62090,AT4G19530,AT4G19110,AT1G21065,AT1G14280,AT5G01810,AT5G61820,AT1G14350,AT1G71695,AT5G01650,AT3G63160,AT1G01970,AT3G62280,AT3G61220,AT5G58760,AT5G58380,ATMG00610,AT5G58140,AT3G60810,AT3G08570,AT2G28390,AT4G10750,AT4G10360,AT4G08320,AT3G58120,AT3G07170,AT2G05520,AT2G05620,AT1G53840,AT4G04340,AT5G54500,AT3G57250,AT5G54080,AT2G24490,AT4G03260,AT1G49270,AT2G01290,AT5G52390,AT2G35690,AT3G03320,AT5G51840,AT3G07550
AGCGGG	10	5	['16-20']	150	ATMG00650,AT1G31270,AT3G05480,AT3G53800,AT2G26520,AT3G01510,AT3G52150,AT5G49450,AT1G14040,AT5G12480,AT2G02280,AT1G66980,AT1G22360,AT3G50860,AT5G46690,AT3G48460,AT2G47780,AT3G47450,AT1G21270,AT4G16860,AT2G35500,AT1G75690,AT3G47130,AT4G16190,AT1G58200,AT3G11250,AT3G11200,AT3G43670,AT1G33490,AT2G31450,AT1G76730,AT3G43020,AT2G38820,AT1G32550,AT4G39710,AT4G30260,AT2G13360,AT1G76550,AT1G66100,AT5G39080,AT5G38480,AT5G19860,AT2G38410,AT1G73760,AT4G38160,AT5G19290,AT5G35560,AT5G20380,AT4G37320,AT3G25690,AT1G79910,AT1G70730,AT3G27980,AT2G19270,AT2G36880,AT1G80310,AT3G28580,AT5G18590,AT3G15290,AT4G33700,AT2G36060,AT5G17310,AT4G32410,AT5G16715,AT4G32530,AT2G42670,AT4G31820,AT5G16430,AT2G22360,AT3G14900,AT4G31310,AT2G43550,AT2G42160,AT2G22450,AT5G14240,AT2G28000,AT1G53210,AT3G14450,AT5G34830,ATMG01180,AT4G29700,AT1G32360,AT5G27010,AT5G26770,AT5G11480,AT1G09160,AT4G27330,AT5G09650,AT3G12920,AT5G66770,AT1G53450,AT5G06790,AT1G09570,AT5G66180,AT5G06460,AT3G20810,AT1G04850,AT1G12960,AT1G12845,AT4G23290,AT3G23660,AT5G63420,AT5G02810,AT1G79730,AT5G63050,AT5G02880,AT3G14000,AT5G02760,AT5G62090,AT4G19530,AT4G19110,AT1G21065,AT1G14280,AT5G01810,AT5G61820,AT1G14350,AT1G71695,AT5G01650,AT3G63160,AT1G01970,AT3G62280,AT3G61220,AT5G58760,AT5G58380,ATMG00610,AT5G58140,AT3G60810,AT3G08570,AT2G28390,AT4G10750,AT4G10360,AT4G08320,AT3G58120,AT3G07170,AT2G05520,AT2G05620,AT1G53840,AT4G04340,AT5G54500,AT3G57250,AT5G54080,AT2G24490,AT4G03260,AT1G49270,AT2G01290,AT5G52390,AT2G35690,AT3G03320,AT5G51840,AT3G07550
ATAAAACGT	10	1	['4-8']	75	AT1G77090,AT1G01080,AT5G39080,AT2G18790,AT4G00370,AT3G62260,AT5G37600,AT1G63340,AT4G27990,AT1G72300,AT1G51240,AT3G61430,AT2G23600,AT5G24930,AT5G67200,AT4G26850,AT1G29240,AT2G28740,AT2G21560,AT5G58120,AT2G43010,AT1G66980,AT2G29250,AT3G50440,AT3G18600,AT3G21420,AT3G09630,AT3G50240,AT5G65910,AT1G58290,AT4G37260,AT4G34710,AT3G18610,AT4G08970,AT3G59480,AT5G55990,AT5G65530,AT4G24810,AT1G80420,AT5G65310,AT3G19880,AT4G24280,AT1G63830,AT5G55070,AT3G03150,AT5G18680,AT2G04390,AT3G47500,AT5G63920,AT3G19680,AT5G03795,AT1G69880,AT2G05520,AT2G05620,AT5G17010,AT4G04340,AT5G43200,AT4G31390,AT2G47410,AT2G03340,AT5G62720,AT3G14000,AT1G56680,AT4G30660,AT4G02540,AT3G55330,AT2G28000,AT1G53210,AT3G14450,AT1G44100,AT5G52320,AT5G52040,AT1G66900,AT5G61410,AT2G15040

ATAAAACGT	10	2	['4-8']	75	AT1G77090,AT1G01080,AT5G39080,AT2G18790,AT4G00370,AT3G62260,AT5G37600,AT1G63340,AT4G27990,AT1G72300,AT1G51240,AT3G61430,AT2G23600,AT5G24930,AT5G67200,AT4G26850,AT1G29240,AT2G28740,AT2G21560,AT5G58120,AT2G43010,AT1G66980,AT2G29250,AT3G50440,AT3G18600,AT3G21420,AT3G09630,AT3G50240,AT5G65910,AT1G58290,AT4G37260,AT4G34710,AT3G18610,AT4G08970,AT3G59480,AT5G55990,AT5G65530,AT4G24810,AT1G80420,AT5G65310,AT3G19880,AT4G24280,AT1G63830,AT5G55070,AT3G03150,AT5G18680,AT2G04390,AT3G47500,AT5G63920,AT3G19680,AT5G03795,AT1G69880,AT2G05520,AT2G05620,AT5G17010,AT4G04340,AT5G43200,AT4G31390,AT2G47410,AT2G03340,AT5G62720,AT3G14000,AT1G56680,AT4G30660,AT4G02540,AT3G55330,AT2G28000,AT1G53210,AT3G14450,AT1G44100,AT5G52320,AT5G52040,AT1G66900,AT5G61410,AT2G15040
ATAAAACGT	10	3	['4-8']	75	AT1G77090,AT1G01080,AT5G39080,AT2G18790,AT4G00370,AT3G62260,AT5G37600,AT1G63340,AT4G27990,AT1G72300,AT1G51240,AT3G61430,AT2G23600,AT5G24930,AT5G67200,AT4G26850,AT1G29240,AT2G28740,AT2G21560,AT5G58120,AT2G43010,AT1G66980,AT2G29250,AT3G50440,AT3G18600,AT3G21420,AT3G09630,AT3G50240,AT5G65910,AT1G58290,AT4G37260,AT4G34710,AT3G18610,AT4G08970,AT3G59480,AT5G55990,AT5G65530,AT4G24810,AT1G80420,AT5G65310,AT3G19880,AT4G24280,AT1G63830,AT5G55070,AT3G03150,AT5G18680,AT2G04390,AT3G47500,AT5G63920,AT3G19680,AT5G03795,AT1G69880,AT2G05520,AT2G05620,AT5G17010,AT4G04340,AT5G43200,AT4G31390,AT2G47410,AT2G03340,AT5G62720,AT3G14000,AT1G56680,AT4G30660,AT4G02540,AT3G55330,AT2G28000,AT1G53210,AT3G14450,AT1G44100,AT5G52320,AT5G52040,AT1G66900,AT5G61410,AT2G15040
ATAAAACGT	10	4	['4-8']	75	AT1G77090,AT1G01080,AT5G39080,AT2G18790,AT4G00370,AT3G62260,AT5G37600,AT1G63340,AT4G27990,AT1G72300,AT1G51240,AT3G61430,AT2G23600,AT5G24930,AT5G67200,AT4G26850,AT1G29240,AT2G28740,AT2G21560,AT5G58120,AT2G43010,AT1G66980,AT2G29250,AT3G50440,AT3G18600,AT3G21420,AT3G09630,AT3G50240,AT5G65910,AT1G58290,AT4G37260,AT4G34710,AT3G18610,AT4G08970,AT3G59480,AT5G55990,AT5G65530,AT4G24810,AT1G80420,AT5G65310,AT3G19880,AT4G24280,AT1G63830,AT5G55070,AT3G03150,AT5G18680,AT2G04390,AT3G47500,AT5G63920,AT3G19680,AT5G03795,AT1G69880,AT2G05520,AT2G05620,AT5G17010,AT4G04340,AT5G43200,AT4G31390,AT2G47410,AT2G03340,AT5G62720,AT3G14000,AT1G56680,AT4G30660,AT4G02540,AT3G55330,AT2G28000,AT1G53210,AT3G14450,AT1G44100,AT5G52320,AT5G52040,AT1G66900,AT5G61410,AT2G15040
ATAAAACGT	10	5	['4-8', '0-4']	75	AT1G77090,AT1G01080,AT5G39080,AT2G18790,AT4G00370,AT3G62260,AT5G37600,AT1G63340,AT4G27990,AT1G72300,AT1G51240,AT3G61430,AT2G23600,AT5G24930,AT5G67200,AT4G26850,AT1G29240,AT2G28740,AT2G21560,AT5G58120,AT2G43010,AT1G66980,AT2G29250,AT3G50440,AT3G18600,AT3G21420,AT3G09630,AT3G50240,AT5G65910,AT1G58290,AT4G37260,AT4G34710,AT3G18610,AT4G08970,AT3G59480,AT5G55990,AT5G65530,AT4G24810,AT1G80420,AT5G65310,AT3G19880,AT4G24280,AT1G63830,AT5G55070,AT3G03150,AT5G18680,AT2G04390,AT3G47500,AT5G63920,AT3G19680,AT5G03795,AT1G69880,AT2G05520,AT2G05620,AT5G17010,AT4G04340,AT5G43200,AT4G31390,AT2G47410,AT2G03340,AT5G62720,AT3G14000,AT1G56680,AT4G30660,AT4G02540,AT3G55330,AT2G28000,AT1G53210,AT3G14450,AT1G44100,AT5G52320,AT5G52040,AT1G66900,AT5G61410,AT2G15040
ATACGTGT	10	1	['0-4']	97	AT3G53720,AT4G00895,AT1G15400,AT5G49710,AT3G52870,AT3G07700,AT3G07770,AT3G52470,AT1G16520,AT3G51240,AT1G28600,AT1G28960,AT1G01470,AT1G21000,AT3G49220,AT4G16845,AT1G58290,AT1G64860,AT3G47650,AT5G44730,AT1G20440,AT2G24090,AT4G16860,AT1G28050,AT1G68920,AT4G15110,AT1G69530,AT1G54880,AT1G56680,AT1G05720,AT5G04140,AT3G43790,AT5G40450,AT1G72770,AT1G75100,AT1G76590,AT5G37780,AT4G38520,AT1G73660,AT3G20680,AT5G35460,AT4G37560,AT3G30380,AT1G67623,AT1G26830,AT2G22990,AT4G33985,AT2G35940,AT3G13110,AT2G45590,AT2G42190,AT3G22060,AT3G15030,AT1G19300,AT2G24880,AT1G78670,AT5G10430,AT3G12780,AT4G27410,AT4G27670,AT3G23030,AT5G66410,AT1G17360,AT3G19500,AT5G64630,AT1G12970,AT1G05910,AT1G79770,AT1G03380,AT4G20160,AT3G17800,AT4G19710,AT2G17330,AT5G62130,AT1G23090,AT5G62140,AT4G19450,AT1G76990,AT4G18520,AT4G18280,AT3G62550,AT3G22420,AT3G62660,AT3G08030,AT5G57760,AT1G80840,AT5G57040,AT3G59060,AT1G80090,AT5G56020,AT4G05050,AT3G57770,AT3G57020,AT3G06410,AT1G70782,AT5G54290,AT1G72810

ATACGTGT	10	2	['0-4']	97	AT3G53720,AT4G00895,AT1G15400,AT5G49710,AT3G52870,AT3G07700,AT3G07770,AT3G52470,AT1G16520,AT3G51240,AT1G28600,AT1G28960,AT1G01470,AT1G21000,AT3G49220,AT4G16845,AT1G58290,AT1G64860,AT3G47650,AT5G44730,AT1G20440,AT2G24090,AT4G16860,AT1G28050,AT1G68920,AT4G15110,AT1G69530,AT1G54880,AT1G56680,AT1G05720,AT5G04140,AT3G43790,AT5G40450,AT1G72770,AT1G75100,AT1G76590,AT5G37780,AT4G38520,AT1G73660,AT3G20680,AT5G35460,AT4G37560,AT3G30380,AT1G67623,AT1G26830,AT2G22990,AT4G33985,AT2G35940,AT3G13110,AT2G45590,AT2G42190,AT3G22060,AT3G15030,AT1G19300,AT2G24880,AT1G78670,AT5G10430,AT3G12780,AT4G27410,AT4G27670,AT3G23030,AT5G66410,AT1G17360,AT3G19500,AT5G64630,AT1G12970,AT1G05910,AT1G79770,AT1G03380,AT4G20160,AT3G17800,AT4G19710,AT2G17330,AT5G62130,AT1G23090,AT5G62140,AT4G19450,AT1G76990,AT4G18520,AT4G18280,AT3G62550,AT3G22420,AT3G62660,AT3G08030,AT5G57760,AT1G80840,AT5G57040,AT3G59060,AT1G80090,AT5G56020,AT4G05050,AT3G57770,AT3G57020,AT3G06410,AT1G70782,AT5G54290,AT1G72810
ATACGTGT	10	3	['0-4']	97	AT3G53720,AT4G00895,AT1G15400,AT5G49710,AT3G52870,AT3G07700,AT3G07770,AT3G52470,AT1G16520,AT3G51240,AT1G28600,AT1G28960,AT1G01470,AT1G21000,AT3G49220,AT4G16845,AT1G58290,AT1G64860,AT3G47650,AT5G44730,AT1G20440,AT2G24090,AT4G16860,AT1G28050,AT1G68920,AT4G15110,AT1G69530,AT1G54880,AT1G56680,AT1G05720,AT5G04140,AT3G43790,AT5G40450,AT1G72770,AT1G75100,AT1G76590,AT5G37780,AT4G38520,AT1G73660,AT3G20680,AT5G35460,AT4G37560,AT3G30380,AT1G67623,AT1G26830,AT2G22990,AT4G33985,AT2G35940,AT3G13110,AT2G45590,AT2G42190,AT3G22060,AT3G15030,AT1G19300,AT2G24880,AT1G78670,AT5G10430,AT3G12780,AT4G27410,AT4G27670,AT3G23030,AT5G66410,AT1G17360,AT3G19500,AT5G64630,AT1G12970,AT1G05910,AT1G79770,AT1G03380,AT4G20160,AT3G17800,AT4G19710,AT2G17330,AT5G62130,AT1G23090,AT5G62140,AT4G19450,AT1G76990,AT4G18520,AT4G18280,AT3G62550,AT3G22420,AT3G62660,AT3G08030,AT5G57760,AT1G80840,AT5G57040,AT3G59060,AT1G80090,AT5G56020,AT4G05050,AT3G57770,AT3G57020,AT3G06410,AT1G70782,AT5G54290,AT1G72810
ATACGTGT	10	4	['0-4']	97	AT3G53720,AT4G00895,AT1G15400,AT5G49710,AT3G52870,AT3G07700,AT3G07770,AT3G52470,AT1G16520,AT3G51240,AT1G28600,AT1G28960,AT1G01470,AT1G21000,AT3G49220,AT4G16845,AT1G58290,AT1G64860,AT3G47650,AT5G44730,AT1G20440,AT2G24090,AT4G16860,AT1G28050,AT1G68920,AT4G15110,AT1G69530,AT1G54880,AT1G56680,AT1G05720,AT5G04140,AT3G43790,AT5G40450,AT1G72770,AT1G75100,AT1G76590,AT5G37780,AT4G38520,AT1G73660,AT3G20680,AT5G35460,AT4G37560,AT3G30380,AT1G67623,AT1G26830,AT2G22990,AT4G33985,AT2G35940,AT3G13110,AT2G45590,AT2G42190,AT3G22060,AT3G15030,AT1G19300,AT2G24880,AT1G78670,AT5G10430,AT3G12780,AT4G27410,AT4G27670,AT3G23030,AT5G66410,AT1G17360,AT3G19500,AT5G64630,AT1G12970,AT1G05910,AT1G79770,AT1G03380,AT4G20160,AT3G17800,AT4G19710,AT2G17330,AT5G62130,AT1G23090,AT5G62140,AT4G19450,AT1G76990,AT4G18520,AT4G18280,AT3G62550,AT3G22420,AT3G62660,AT3G08030,AT5G57760,AT1G80840,AT5G57040,AT3G59060,AT1G80090,AT5G56020,AT4G05050,AT3G57770,AT3G57020,AT3G06410,AT1G70782,AT5G54290,AT1G72810
ATACGTGT	10	5	['0-4']	97	AT3G53720,AT4G00895,AT1G15400,AT5G49710,AT3G52870,AT3G07700,AT3G07770,AT3G52470,AT1G16520,AT3G51240,AT1G28600,AT1G28960,AT1G01470,AT1G21000,AT3G49220,AT4G16845,AT1G58290,AT1G64860,AT3G47650,AT5G44730,AT1G20440,AT2G24090,AT4G16860,AT1G28050,AT1G68920,AT4G15110,AT1G69530,AT1G54880,AT1G56680,AT1G05720,AT5G04140,AT3G43790,AT5G40450,AT1G72770,AT1G75100,AT1G76590,AT5G37780,AT4G38520,AT1G73660,AT3G20680,AT5G35460,AT4G37560,AT3G30380,AT1G67623,AT1G26830,AT2G22990,AT4G33985,AT2G35940,AT3G13110,AT2G45590,AT2G42190,AT3G22060,AT3G15030,AT1G19300,AT2G24880,AT1G78670,AT5G10430,AT3G12780,AT4G27410,AT4G27670,AT3G23030,AT5G66410,AT1G17360,AT3G19500,AT5G64630,AT1G12970,AT1G05910,AT1G79770,AT1G03380,AT4G20160,AT3G17800,AT4G19710,AT2G17330,AT5G62130,AT1G23090,AT5G62140,AT4G19450,AT1G76990,AT4G18520,AT4G18280,AT3G62550,AT3G22420,AT3G62660,AT3G08030,AT5G57760,AT1G80840,AT5G57040,AT3G59060,AT1G80090,AT5G56020,AT4G05050,AT3G57770,AT3G57020,AT3G06410,AT1G70782,AT5G54290,AT1G72810
ATAGAAATCAA	10	1	['4-8', '12-16', '20-24', '8-12']	10	AT2G10370,AT5G22270,AT3G08570,AT3G17430,AT2G34430,AT1G21065,AT3G54990,AT3G19970,AT2G28200,AT2G31790
ATAGAAATCAA	10	2	['4-8', '12-16', '20-24']	10	AT2G10370,AT5G22270,AT3G08570,AT3G17430,AT2G34430,AT1G21065,AT3G54990,AT3G19970,AT2G28200,AT2G31790

ATAGAAATCAA	10	3	['4-8', '8-12', '12-16', '20-24']	10	AT2G10370,AT5G22270,AT3G08570,AT3G17430,AT2G34430,AT1G21065,AT3G54990,AT3G19970,AT2G28200,AT2G31790
ATAGAAATCAA	10	4	['4-8', '12-16', '20-24']	10	AT2G10370,AT5G22270,AT3G08570,AT3G17430,AT2G34430,AT1G21065,AT3G54990,AT3G19970,AT2G28200,AT2G31790
ATAGAAATCAA	10	5	['4-8', '12-16', '20-24']	10	AT2G10370,AT5G22270,AT3G08570,AT3G17430,AT2G34430,AT1G21065,AT3G54990,AT3G19970,AT2G28200,AT2G31790
ATCCAACC	10	1	['4-8', '8-12', '12-16']	81	AT1G63630,AT3G63310,AT1G54830,AT4G00960,AT4G39400,AT2G26600,AT2G26520,AT4G00895,AT1G75140,AT5G26770,AT5G49990,AT3G01750,AT3G62100,AT5G26020,AT2G17220,AT2G38400,AT5G59340,AT1G62480,AT1G62750,AT1G16720,AT5G49120,AT4G12730,AT1G19920,AT1G73820,AT3G52380,AT1G21500,AT3G07890,AT3G27090,AT3G07880,AT1G28530,AT1G06010,AT1G16330,AT4G37320,AT5G47640,AT2G04900,AT4G35890,AT4G15440,AT4G36470,AT4G09760,AT4G09970,AT1G21680,AT5G06830,AT5G65720,AT3G59060,AT1G07020,AT5G65000,AT4G16410,AT3G16220,AT3G47480,AT1G30120,AT5G18680,AT4G23820,AT5G18170,AT1G12780,AT5G55480,AT4G16140,AT2G05520,AT5G17870,AT5G03240,AT3G18080,AT5G53880,AT2G30570,AT5G61850,AT1G08640,AT5G03190,AT5G53770,AT5G02820,AT2G03420,AT1G03600,AT5G02830,AT2G17340,AT1G56720,AT2G19750,AT1G73020,AT3G55450,AT2G47010,AT1G72170,AT2G16500,AT4G00830,AT4G30060,AT4G19420
ATCCAACC	10	2	['4-8', '8-12', '12-16']	81	AT1G63630,AT3G63310,AT1G54830,AT4G00960,AT4G39400,AT2G26600,AT2G26520,AT4G00895,AT1G75140,AT5G26770,AT5G49990,AT3G01750,AT3G62100,AT5G26020,AT2G17220,AT2G38400,AT5G59340,AT1G62480,AT1G62750,AT1G16720,AT5G49120,AT4G12730,AT1G19920,AT1G73820,AT3G52380,AT1G21500,AT3G07890,AT3G27090,AT3G07880,AT1G28530,AT1G06010,AT1G16330,AT4G37320,AT5G47640,AT2G04900,AT4G35890,AT4G15440,AT4G36470,AT4G09760,AT4G09970,AT1G21680,AT5G06830,AT5G65720,AT3G59060,AT1G07020,AT5G65000,AT4G16410,AT3G16220,AT3G47480,AT1G30120,AT5G18680,AT4G23820,AT5G18170,AT1G12780,AT5G55480,AT4G16140,AT2G05520,AT5G17870,AT5G03240,AT3G18080,AT5G53880,AT2G30570,AT5G61850,AT1G08640,AT5G03190,AT5G53770,AT5G02820,AT2G03420,AT1G03600,AT5G02830,AT2G17340,AT1G56720,AT2G19750,AT1G73020,AT3G55450,AT2G47010,AT1G72170,AT2G16500,AT4G00830,AT4G30060,AT4G19420
ATCCAACC	10	3	['4-8', '8-12', '12-16']	81	AT1G63630,AT3G63310,AT1G54830,AT4G00960,AT4G39400,AT2G26600,AT2G26520,AT4G00895,AT1G75140,AT5G26770,AT5G49990,AT3G01750,AT3G62100,AT5G26020,AT2G17220,AT2G38400,AT5G59340,AT1G62480,AT1G62750,AT1G16720,AT5G49120,AT4G12730,AT1G19920,AT1G73820,AT3G52380,AT1G21500,AT3G07890,AT3G27090,AT3G07880,AT1G28530,AT1G06010,AT1G16330,AT4G37320,AT5G47640,AT2G04900,AT4G35890,AT4G15440,AT4G36470,AT4G09760,AT4G09970,AT1G21680,AT5G06830,AT5G65720,AT3G59060,AT1G07020,AT5G65000,AT4G16410,AT3G16220,AT3G47480,AT1G30120,AT5G18680,AT4G23820,AT5G18170,AT1G12780,AT5G55480,AT4G16140,AT2G05520,AT5G17870,AT5G03240,AT3G18080,AT5G53880,AT2G30570,AT5G61850,AT1G08640,AT5G03190,AT5G53770,AT5G02820,AT2G03420,AT1G03600,AT5G02830,AT2G17340,AT1G56720,AT2G19750,AT1G73020,AT3G55450,AT2G47010,AT1G72170,AT2G16500,AT4G00830,AT4G30060,AT4G19420
ATCCAACC	10	4	['4-8', '8-12', '12-16']	81	AT1G63630,AT3G63310,AT1G54830,AT4G00960,AT4G39400,AT2G26600,AT2G26520,AT4G00895,AT1G75140,AT5G26770,AT5G49990,AT3G01750,AT3G62100,AT5G26020,AT2G17220,AT2G38400,AT5G59340,AT1G62480,AT1G62750,AT1G16720,AT5G49120,AT4G12730,AT1G19920,AT1G73820,AT3G52380,AT1G21500,AT3G07890,AT3G27090,AT3G07880,AT1G28530,AT1G06010,AT1G16330,AT4G37320,AT5G47640,AT2G04900,AT4G35890,AT4G15440,AT4G36470,AT4G09760,AT4G09970,AT1G21680,AT5G06830,AT5G65720,AT3G59060,AT1G07020,AT5G65000,AT4G16410,AT3G16220,AT3G47480,AT1G30120,AT5G18680,AT4G23820,AT5G18170,AT1G12780,AT5G55480,AT4G16140,AT2G05520,AT5G17870,AT5G03240,AT3G18080,AT5G53880,AT2G30570,AT5G61850,AT1G08640,AT5G03190,AT5G53770,AT5G02820,AT2G03420,AT1G03600,AT5G02830,AT2G17340,AT1G56720,AT2G19750,AT1G73020,AT3G55450,AT2G47010,AT1G72170,AT2G16500,AT4G00830,AT4G30060,AT4G19420

ATCCAACC	10	5	['4-8', '8-12', '12-16']	81	AT1G63630,AT3G63310,AT1G54830,AT4G00960,AT4G39400,AT2G26600,AT2G26520,AT4G00895,AT1G75140,AT5G26770,AT5G49990,AT3G01750,AT3G62100,AT5G26020,AT2G17220,AT2G38400,AT5G59340,AT1G62480,AT1G62750,AT1G16720,AT5G49120,AT4G12730,AT1G19920,AT1G73820,AT3G52380,AT1G21500,AT3G07890,AT3G27090,AT3G07880,AT1G28530,AT1G06010,AT1G16330,AT4G37320,AT5G47640,AT2G04900,AT4G35890,AT4G15440,AT4G36470,AT4G09760,AT4G09970,AT1G21680,AT5G06830,AT5G65720,AT3G59060,AT1G07020,AT5G65000,AT4G16410,AT3G16220,AT3G47480,AT1G30120,AT5G18680,AT4G23820,AT5G18170,AT1G12780,AT5G55480,AT4G16140,AT2G05520,AT5G17870,AT5G03240,AT3G18080,AT5G53880,AT2G30570,AT5G61850,AT1G08640,AT5G03190,AT5G53770,AT5G02820,AT2G03420,AT1G03600,AT5G02830,AT2G17340,AT1G56720,AT2G19750,AT1G73020,AT3G55450,AT2G47010,AT1G72170,AT2G16500,AT4G00830,AT4G30060,AT4G19420
ATCCTACC	10	1	['4-8', '8-12', '16-20', '20-24']	33	AT3G15450,AT4G28740,AT5G11580,AT2G47450,AT2G41080,AT5G36950,AT3G01090,AT5G19290,AT1G13440,AT4G25530,AT1G08070,AT5G57760,AT1G44446,AT5G06870,AT5G06260,AT4G37220,AT3G19810,AT2G13260,AT2G46340,AT5G44730,AT3G14080,AT2G15620,AT2G44300,AT1G20440,AT5G17630,AT5G63620,AT4G02370,AT2G42190,AT5G14310,AT1G14400,AT1G79050,AT4G19390,AT5G40450
ATCCTACC	10	2	['4-8', '8-12', '16-20', '20-24']	33	AT3G15450,AT4G28740,AT5G11580,AT2G47450,AT2G41080,AT5G36950,AT3G01090,AT5G19290,AT1G13440,AT4G25530,AT1G08070,AT5G57760,AT1G44446,AT5G06870,AT5G06260,AT4G37220,AT3G19810,AT2G13260,AT2G46340,AT5G44730,AT3G14080,AT2G15620,AT2G44300,AT1G20440,AT5G17630,AT5G63620,AT4G02370,AT2G42190,AT5G14310,AT1G14400,AT1G79050,AT4G19390,AT5G40450
ATCCTACC	10	3	['4-8', '8-12', '16-20', '20-24']	33	AT3G15450,AT4G28740,AT5G11580,AT2G47450,AT2G41080,AT5G36950,AT3G01090,AT5G19290,AT1G13440,AT4G25530,AT1G08070,AT5G57760,AT1G44446,AT5G06870,AT5G06260,AT4G37220,AT3G19810,AT2G13260,AT2G46340,AT5G44730,AT3G14080,AT2G15620,AT2G44300,AT1G20440,AT5G17630,AT5G63620,AT4G02370,AT2G42190,AT5G14310,AT1G14400,AT1G79050,AT4G19390,AT5G40450
ATCCTACC	10	4	['4-8', '8-12', '16-20', '20-24']	33	AT3G15450,AT4G28740,AT5G11580,AT2G47450,AT2G41080,AT5G36950,AT3G01090,AT5G19290,AT1G13440,AT4G25530,AT1G08070,AT5G57760,AT1G44446,AT5G06870,AT5G06260,AT4G37220,AT3G19810,AT2G13260,AT2G46340,AT5G44730,AT3G14080,AT2G15620,AT2G44300,AT1G20440,AT5G17630,AT5G63620,AT4G02370,AT2G42190,AT5G14310,AT1G14400,AT1G79050,AT4G19390,AT5G40450
ATCCTACC	10	5	['4-8', '8-12', '16-20', '20-24']	33	AT3G15450,AT4G28740,AT5G11580,AT2G47450,AT2G41080,AT5G36950,AT3G01090,AT5G19290,AT1G13440,AT4G25530,AT1G08070,AT5G57760,AT1G44446,AT5G06870,AT5G06260,AT4G37220,AT3G19810,AT2G13260,AT2G46340,AT5G44730,AT3G14080,AT2G15620,AT2G44300,AT1G20440,AT5G17630,AT5G63620,AT4G02370,AT2G42190,AT5G14310,AT1G14400,AT1G79050,AT4G19390,AT5G40450
ATTCCCGC	10	1	['0-4', '8-12', '12-16', '20-24']	17	AT1G32090,AT5G11670,AT1G31814,AT1G75080,AT3G02340,AT3G60750,AT3G01860,AT3G19640,AT3G16190,AT4G13580,AT3G52720,AT5G13730,AT5G05950,AT4G34710,AT3G46970,AT5G27830,AT5G63135
ATTCCCGC	10	2	['0-4', '8-12', '12-16', '20-24']	17	AT1G32090,AT5G11670,AT1G31814,AT1G75080,AT3G02340,AT3G60750,AT3G01860,AT3G19640,AT3G16190,AT4G13580,AT3G52720,AT5G13730,AT5G05950,AT4G34710,AT3G46970,AT5G27830,AT5G63135
ATTCCCGC	10	3	['0-4', '8-12', '12-16', '20-24']	17	AT1G32090,AT5G11670,AT1G31814,AT1G75080,AT3G02340,AT3G60750,AT3G01860,AT3G19640,AT3G16190,AT4G13580,AT3G52720,AT5G13730,AT5G05950,AT4G34710,AT3G46970,AT5G27830,AT5G63135
ATTCCCGC	10	4	['0-4', '8-12', '12-16', '20-24']	17	AT1G32090,AT5G11670,AT1G31814,AT1G75080,AT3G02340,AT3G60750,AT3G01860,AT3G19640,AT3G16190,AT4G13580,AT3G52720,AT5G13730,AT5G05950,AT4G34710,AT3G46970,AT5G27830,AT5G63135
ATTCCCGC	10	5	['0-4', '8-12', '12-16', '20-24']	17	AT1G32090,AT5G11670,AT1G31814,AT1G75080,AT3G02340,AT3G60750,AT3G01860,AT3G19640,AT3G16190,AT4G13580,AT3G52720,AT5G13730,AT5G05950,AT4G34710,AT3G46970,AT5G27830,AT5G63135
ATTCGCGC	10	1	['0-4', '4-8', '12-16']	19	AT1G63440,AT5G56240,AT2G24930,AT3G12290,AT3G63190,AT5G67370,AT5G42950,AT5G15450,AT3G01500,AT1G18720,AT1G32920,AT1G03970,AT5G10860,AT4G09970,AT1G01430,AT1G11630,AT4G13010,AT1G19110,AT3G59350
ATTCGCGC	10	2	['0-4', '4-8', '12-16']	19	AT1G63440,AT5G56240,AT2G24930,AT3G12290,AT3G63190,AT5G67370,AT5G42950,AT5G15450,AT3G01500,AT1G18720,AT1G32920,AT1G03970,AT5G10860,AT4G09970,AT1G01430,AT1G11630,AT4G13010,AT1G19110,AT3G59350
ATTCGCGC	10	3	['0-4', '4-8', '12-16']	19	AT1G63440,AT5G56240,AT2G24930,AT3G12290,AT3G63190,AT5G67370,AT5G42950,AT5G15450,AT3G01500,AT1G18720,AT1G32920,AT1G03970,AT5G10860,AT4G09970,AT1G01430,AT1G11630,AT4G13010,AT1G19110,AT3G59350

ATTCGCGC	10	4	['0-4', '12-16', '4-8']	19	AT1G63440,AT5G56240,AT2G24930,AT3G12290,AT3G63190,AT5G67370,AT5G42950,AT5G15450,AT3G01500,AT1G18720,AT1G32920,AT1G03970,AT5G10860,AT4G09970,AT1G01430,AT1G11630,AT4G13010,AT1G19110,AT3G59350
ATTCGCGC	10	5	['0-4', '4-8', '12-16']	19	AT1G63440,AT5G56240,AT2G24930,AT3G12290,AT3G63190,AT5G67370,AT5G42950,AT5G15450,AT3G01500,AT1G18720,AT1G32920,AT1G03970,AT5G10860,AT4G09970,AT1G01430,AT1G11630,AT4G13010,AT1G19110,AT3G59350
ATTTATATAAAT	10	1	['4-8']	22	AT5G39210,AT1G34300,AT4G28420,AT2G31510,AT5G10210,AT1G51240,AT5G48230,AT1G18020,AT5G20380,AT1G52760,AT1G72710,AT2G31010,AT1G13130,AT1G12200,AT1G32870,AT2G15960,AT5G17730,AT4G29610,AT1G71810,AT1G71820,AT2G25680,AT5G40290
ATTTATATAAAT	10	2	['4-8']	22	AT5G39210,AT1G34300,AT4G28420,AT2G31510,AT5G10210,AT1G51240,AT5G48230,AT1G18020,AT5G20380,AT1G52760,AT1G72710,AT2G31010,AT1G13130,AT1G12200,AT1G32870,AT2G15960,AT5G17730,AT4G29610,AT1G71810,AT1G71820,AT2G25680,AT5G40290
ATTTATATAAAT	10	3	['4-8']	22	AT5G39210,AT1G34300,AT4G28420,AT2G31510,AT5G10210,AT1G51240,AT5G48230,AT1G18020,AT5G20380,AT1G52760,AT1G72710,AT2G31010,AT1G13130,AT1G12200,AT1G32870,AT2G15960,AT5G17730,AT4G29610,AT1G71810,AT1G71820,AT2G25680,AT5G40290
ATTTATATAAAT	10	4	['4-8']	22	AT5G39210,AT1G34300,AT4G28420,AT2G31510,AT5G10210,AT1G51240,AT5G48230,AT1G18020,AT5G20380,AT1G52760,AT1G72710,AT2G31010,AT1G13130,AT1G12200,AT1G32870,AT2G15960,AT5G17730,AT4G29610,AT1G71810,AT1G71820,AT2G25680,AT5G40290
ATTTATATAAAT	10	5	['4-8']	22	AT5G39210,AT1G34300,AT4G28420,AT2G31510,AT5G10210,AT1G51240,AT5G48230,AT1G18020,AT5G20380,AT1G52760,AT1G72710,AT2G31010,AT1G13130,AT1G12200,AT1G32870,AT2G15960,AT5G17730,AT4G29610,AT1G71810,AT1G71820,AT2G25680,AT5G40290
CAAAACGC	10	1	['12-16', '16-20']	101	AT1G14140,AT4G00400,AT1G06470,AT2G40890,AT2G38000,AT3G07640,AT2G33250,AT3G51430,AT3G03710,AT5G48140,AT3G50830,AT3G50530,AT3G50500,AT4G16760,AT5G46110,AT3G48780,AT1G07040,AT3G47430,AT1G51660,AT1G54390,AT1G63080,AT3G12470,AT2G25870,AT1G56700,AT1G78570,AT1G65090,AT5G41600,AT2G25950,AT1G76790,AT2G44670,AT5G39570,AT5G39090,AT2G39400,AT3G13750,AT5G24150,AT4G36910,AT4G35770,AT4G37260,AT4G34590,AT1G80440,AT5G19010,AT1G69880,AT5G17170,AT2G35940,AT2G12870,AT2G32180,AT2G22360,AT3G16520,AT3G14900,AT2G43520,AT5G14800,AT3G24190,AT3G29760,AT5G13730,AT4G29750,AT4G29670,AT4G27940,AT3G24715,AT5G11160,AT5G26030,AT4G28100,AT5G10240,AT5G66880,AT5G66920,AT5G06970,AT1G09380,AT1G09570,AT3G19930,AT1G19650,AT4G22930,AT5G63380,AT4G21180,AT1G03430,AT5G02880,AT4G19830,AT5G62220,AT5G01750,AT3G23325,AT3G16040,AT1G61140,AT3G15510,AT5G61130,AT1G01970,AT3G61830,AT1G47310,AT1G08380,AT4G11175,AT1G10650,AT3G59660,AT2G18230,AT5G56170,AT3G59052,AT2G06510,AT3G59400,AT5G55220,AT3G57040,AT3G57190,AT1G53840,AT3G56410,AT4G02940,AT5G52320
CAAAACGC	10	2	['16-20', '12-16']	101	AT1G14140,AT4G00400,AT1G06470,AT2G40890,AT2G38000,AT3G07640,AT2G33250,AT3G51430,AT3G03710,AT5G48140,AT3G50830,AT3G50530,AT3G50500,AT4G16760,AT5G46110,AT3G48780,AT1G07040,AT3G47430,AT1G51660,AT1G54390,AT1G63080,AT3G12470,AT2G25870,AT1G56700,AT1G78570,AT1G65090,AT5G41600,AT2G25950,AT1G76790,AT2G44670,AT5G39570,AT5G39090,AT2G39400,AT3G13750,AT5G24150,AT4G36910,AT4G35770,AT4G37260,AT4G34590,AT1G80440,AT5G19010,AT1G69880,AT5G17170,AT2G35940,AT2G12870,AT2G32180,AT2G22360,AT3G16520,AT3G14900,AT2G43520,AT5G14800,AT3G24190,AT3G29760,AT5G13730,AT4G29750,AT4G29670,AT4G27940,AT3G24715,AT5G11160,AT5G26030,AT4G28100,AT5G10240,AT5G66880,AT5G66920,AT5G06970,AT1G09380,AT1G09570,AT3G19930,AT1G19650,AT4G22930,AT5G63380,AT4G21180,AT1G03430,AT5G02880,AT4G19830,AT5G62220,AT5G01750,AT3G23325,AT3G16040,AT1G61140,AT3G15510,AT5G61130,AT1G01970,AT3G61830,AT1G47310,AT1G08380,AT4G11175,AT1G10650,AT3G59660,AT2G18230,AT5G56170,AT3G59052,AT2G06510,AT3G59400,AT5G55220,AT3G57040,AT3G57190,AT1G53840,AT3G56410,AT4G02940,AT5G52320

CAAAACGC	10	3	['12-16', '16-20']	101	AT1G14140,AT4G00400,AT1G06470,AT2G40890,AT2G38000,AT3G07640,AT2G33250,AT3G51430,AT3G03710,AT5G48140,AT3G50830,AT3G50530,AT3G50500,AT4G16760,AT5G46110,AT3G48780,AT1G07040,AT3G47430,AT1G51660,AT1G54390,AT1G63080,AT3G12470,AT2G25870,AT1G56700,AT1G78570,AT1G65090,AT5G41600,AT2G25950,AT1G76790,AT2G44670,AT5G39570,AT5G39090,AT2G39400,AT3G13750,AT5G24150,AT4G36910,AT4G35770,AT4G37260,AT4G34590,AT1G80440,AT5G19010,AT1G69880,AT5G17170,AT2G35940,AT2G12870,AT2G32180,AT2G22360,AT3G16520,AT3G14900,AT2G43520,AT5G14800,AT3G24190,AT3G29760,AT5G13730,AT4G29750,AT4G29670,AT4G27940,AT3G24715,AT5G11160,AT5G26030,AT4G28100,AT5G10240,AT5G66880,AT5G66920,AT5G06970,AT1G09380,AT1G09570,AT3G19930,AT1G19650,AT4G22930,AT5G63380,AT4G21180,AT1G03430,AT5G02880,AT4G19830,AT5G62220,AT5G01750,AT3G23325,AT3G16040,AT1G61140,AT3G15510,AT5G61130,AT1G01970,AT3G61830,AT1G47310,AT1G08380,AT4G11175,AT1G10650,AT3G59660,AT2G18230,AT5G56170,AT3G59052,AT2G06510,AT3G59400,AT5G55220,AT3G57040,AT3G57190,AT1G53840,AT3G56410,AT4G02940,AT5G52320
CAAAACGC	10	4	['12-16', '16-20']	101	AT1G14140,AT4G00400,AT1G06470,AT2G40890,AT2G38000,AT3G07640,AT2G33250,AT3G51430,AT3G03710,AT5G48140,AT3G50830,AT3G50530,AT3G50500,AT4G16760,AT5G46110,AT3G48780,AT1G07040,AT3G47430,AT1G51660,AT1G54390,AT1G63080,AT3G12470,AT2G25870,AT1G56700,AT1G78570,AT1G65090,AT5G41600,AT2G25950,AT1G76790,AT2G44670,AT5G39570,AT5G39090,AT2G39400,AT3G13750,AT5G24150,AT4G36910,AT4G35770,AT4G37260,AT4G34590,AT1G80440,AT5G19010,AT1G69880,AT5G17170,AT2G35940,AT2G12870,AT2G32180,AT2G22360,AT3G16520,AT3G14900,AT2G43520,AT5G14800,AT3G24190,AT3G29760,AT5G13730,AT4G29750,AT4G29670,AT4G27940,AT3G24715,AT5G11160,AT5G26030,AT4G28100,AT5G10240,AT5G66880,AT5G66920,AT5G06970,AT1G09380,AT1G09570,AT3G19930,AT1G19650,AT4G22930,AT5G63380,AT4G21180,AT1G03430,AT5G02880,AT4G19830,AT5G62220,AT5G01750,AT3G23325,AT3G16040,AT1G61140,AT3G15510,AT5G61130,AT1G01970,AT3G61830,AT1G47310,AT1G08380,AT4G11175,AT1G10650,AT3G59660,AT2G18230,AT5G56170,AT3G59052,AT2G06510,AT3G59400,AT5G55220,AT3G57040,AT3G57190,AT1G53840,AT3G56410,AT4G02940,AT5G52320
CAAAACGC	10	5	['12-16', '16-20']	101	AT1G14140,AT4G00400,AT1G06470,AT2G40890,AT2G38000,AT3G07640,AT2G33250,AT3G51430,AT3G03710,AT5G48140,AT3G50830,AT3G50530,AT3G50500,AT4G16760,AT5G46110,AT3G48780,AT1G07040,AT3G47430,AT1G51660,AT1G54390,AT1G63080,AT3G12470,AT2G25870,AT1G56700,AT1G78570,AT1G65090,AT5G41600,AT2G25950,AT1G76790,AT2G44670,AT5G39570,AT5G39090,AT2G39400,AT3G13750,AT5G24150,AT4G36910,AT4G35770,AT4G37260,AT4G34590,AT1G80440,AT5G19010,AT1G69880,AT5G17170,AT2G35940,AT2G12870,AT2G32180,AT2G22360,AT3G16520,AT3G14900,AT2G43520,AT5G14800,AT3G24190,AT3G29760,AT5G13730,AT4G29750,AT4G29670,AT4G27940,AT3G24715,AT5G11160,AT5G26030,AT4G28100,AT5G10240,AT5G66880,AT5G66920,AT5G06970,AT1G09380,AT1G09570,AT3G19930,AT1G19650,AT4G22930,AT5G63380,AT4G21180,AT1G03430,AT5G02880,AT4G19830,AT5G62220,AT5G01750,AT3G23325,AT3G16040,AT1G61140,AT3G15510,AT5G61130,AT1G01970,AT3G61830,AT1G47310,AT1G08380,AT4G11175,AT1G10650,AT3G59660,AT2G18230,AT5G56170,AT3G59052,AT2G06510,AT3G59400,AT5G55220,AT3G57040,AT3G57190,AT1G53840,AT3G56410,AT4G02940,AT5G52320
CAATAATTG	10	1	['0-4']	60	AT4G29040,ATCG00650,AT4G00730,AT5G11930,AT5G26340,AT1G14140,AT3G19553,AT1G06470,AT2G47600,AT1G78320,AT1G04120,AT3G01180,AT1G76280,AT1G06040,AT1G28580,AT2G01860,AT4G01070,AT3G13750,AT1G20840,AT1G01240,AT1G29900,AT1G74670,AT3G59920,AT2G38670,AT3G19810,AT2G30140,AT1G64640,AT4G08840,AT2G46400,AT1G20650,AT1G20620,AT2G46530,AT5G55530,AT5G44800,AT1G70000,AT5G18680,AT1G74940,AT1G74920,AT1G42970,AT2G36390,AT4G16480,AT5G54390,AT1G68920,AT2G45690,AT1G68190,AT3G27210,AT2G42770,AT5G42760,AT3G23690,AT1G49500,AT3G07310,AT1G05720,AT5G52320,AT5G34830,AT5G51970,AT1G68440,AT4G29670,AT5G11890,AT3G54240,AT5G12250
CAATAATTG	10	2	['0-4']	60	AT4G29040,ATCG00650,AT4G00730,AT5G11930,AT5G26340,AT1G14140,AT3G19553,AT1G06470,AT2G47600,AT1G78320,AT1G04120,AT3G01180,AT1G76280,AT1G06040,AT1G28580,AT2G01860,AT4G01070,AT3G13750,AT1G20840,AT1G01240,AT1G29900,AT1G74670,AT3G59920,AT2G38670,AT3G19810,AT2G30140,AT1G64640,AT4G08840,AT2G46400,AT1G20650,AT1G20620,AT2G46530,AT5G55530,AT5G44800,AT1G70000,AT5G18680,AT1G74940,AT1G74920,AT1G42970,AT2G36390,AT4G16480,AT5G54390,AT1G68920,AT2G45690,AT1G68190,AT3G27210,AT2G42770,AT5G42760,AT3G23690,AT1G49500,AT3G07310,AT1G05720,AT5G52320,AT5G34830,AT5G51970,AT1G68440,AT4G29670,AT5G11890,AT3G54240,AT5G12250

CAATAATTG	10	3	['0-4']	60	AT4G29040,ATCG00650,AT4G00730,AT5G11930,AT5G26340,AT1G14140,AT3G19553,AT1G06470,AT2G47600,AT1G78320,AT1G04120,AT3G01180,AT1G76280,AT1G06040,AT1G28580,AT2G01860,AT4G01070,AT3G13750,AT1G20840,AT1G01240,AT1G29900,AT1G74670,AT3G59920,AT2G38670,AT3G19810,AT2G30140,AT1G64640,AT4G08840,AT2G46400,AT1G20650,AT1G20620,AT2G46530,AT5G55530,AT5G44800,AT1G70000,AT5G18680,AT1G74940,AT1G74920,AT1G42970,AT2G36390,AT4G16480,AT5G54390,AT1G68920,AT2G45690,AT1G68190,AT3G27210,AT2G42770,AT5G42760,AT3G23690,AT1G49500,AT3G07310,AT1G05720,AT5G52320,AT5G34830,AT5G51970,AT1G68440,AT4G29670,AT5G11890,AT3G54240,AT5G12250
CAATAATTG	10	4	['0-4']	60	AT4G29040,ATCG00650,AT4G00730,AT5G11930,AT5G26340,AT1G14140,AT3G19553,AT1G06470,AT2G47600,AT1G78320,AT1G04120,AT3G01180,AT1G76280,AT1G06040,AT1G28580,AT2G01860,AT4G01070,AT3G13750,AT1G20840,AT1G01240,AT1G29900,AT1G74670,AT3G59920,AT2G38670,AT3G19810,AT2G30140,AT1G64640,AT4G08840,AT2G46400,AT1G20650,AT1G20620,AT2G46530,AT5G55530,AT5G44800,AT1G70000,AT5G18680,AT1G74940,AT1G74920,AT1G42970,AT2G36390,AT4G16480,AT5G54390,AT1G68920,AT2G45690,AT1G68190,AT3G27210,AT2G42770,AT5G42760,AT3G23690,AT1G49500,AT3G07310,AT1G05720,AT5G52320,AT5G34830,AT5G51970,AT1G68440,AT4G29670,AT5G11890,AT3G54240,AT5G12250
CAATAATTG	10	5	['0-4']	60	AT4G29040,ATCG00650,AT4G00730,AT5G11930,AT5G26340,AT1G14140,AT3G19553,AT1G06470,AT2G47600,AT1G78320,AT1G04120,AT3G01180,AT1G76280,AT1G06040,AT1G28580,AT2G01860,AT4G01070,AT3G13750,AT1G20840,AT1G01240,AT1G29900,AT1G74670,AT3G59920,AT2G38670,AT3G19810,AT2G30140,AT1G64640,AT4G08840,AT2G46400,AT1G20650,AT1G20620,AT2G46530,AT5G55530,AT5G44800,AT1G70000,AT5G18680,AT1G74940,AT1G74920,AT1G42970,AT2G36390,AT4G16480,AT5G54390,AT1G68920,AT2G45690,AT1G68190,AT3G27210,AT2G42770,AT5G42760,AT3G23690,AT1G49500,AT3G07310,AT1G05720,AT5G52320,AT5G34830,AT5G51970,AT1G68440,AT4G29670,AT5G11890,AT3G54240,AT5G12250
CAATCATTG	10	1	['0-4', '12-16', '16-20']	37	AT1G19540,AT1G19300,AT5G60850,AT5G38990,AT1G14990,AT4G39270,AT1G50030,AT3G61430,AT3G61470,AT1G76100,AT3G27170,AT1G12990,AT5G57960,AT1G01620,AT5G46020,AT3G05800,AT4G24810,AT1G16570,AT3G21240,AT3G47340,AT4G23820,AT2G44300,AT5G63860,AT1G75690,AT3G31980,AT1G26800,AT2G45690,AT5G63410,AT2G13020,AT4G03260,AT1G18720,AT1G72175,AT5G62090,AT3G15030,AT5G14090,AT5G34830,AT2G34620
CAATCATTG	10	2	['16-20']	37	AT1G19540,AT1G19300,AT5G60850,AT5G38990,AT1G14990,AT4G39270,AT1G50030,AT3G61430,AT3G61470,AT1G76100,AT3G27170,AT1G12990,AT5G57960,AT1G01620,AT5G46020,AT3G05800,AT4G24810,AT1G16570,AT3G21240,AT3G47340,AT4G23820,AT2G44300,AT5G63860,AT1G75690,AT3G31980,AT1G26800,AT2G45690,AT5G63410,AT2G13020,AT4G03260,AT1G18720,AT1G72175,AT5G62090,AT3G15030,AT5G14090,AT5G34830,AT2G34620
CAATCATTG	10	3	['0-4']	37	AT1G19540,AT1G19300,AT5G60850,AT5G38990,AT1G14990,AT4G39270,AT1G50030,AT3G61430,AT3G61470,AT1G76100,AT3G27170,AT1G12990,AT5G57960,AT1G01620,AT5G46020,AT3G05800,AT4G24810,AT1G16570,AT3G21240,AT3G47340,AT4G23820,AT2G44300,AT5G63860,AT1G75690,AT3G31980,AT1G26800,AT2G45690,AT5G63410,AT2G13020,AT4G03260,AT1G18720,AT1G72175,AT5G62090,AT3G15030,AT5G14090,AT5G34830,AT2G34620
CAATCATTG	10	4	['0-4', '16-20']	37	AT1G19540,AT1G19300,AT5G60850,AT5G38990,AT1G14990,AT4G39270,AT1G50030,AT3G61430,AT3G61470,AT1G76100,AT3G27170,AT1G12990,AT5G57960,AT1G01620,AT5G46020,AT3G05800,AT4G24810,AT1G16570,AT3G21240,AT3G47340,AT4G23820,AT2G44300,AT5G63860,AT1G75690,AT3G31980,AT1G26800,AT2G45690,AT5G63410,AT2G13020,AT4G03260,AT1G18720,AT1G72175,AT5G62090,AT3G15030,AT5G14090,AT5G34830,AT2G34620
CAATCATTG	10	5	['12-16', '0-4', '16-20']	37	AT1G19540,AT1G19300,AT5G60850,AT5G38990,AT1G14990,AT4G39270,AT1G50030,AT3G61430,AT3G61470,AT1G76100,AT3G27170,AT1G12990,AT5G57960,AT1G01620,AT5G46020,AT3G05800,AT4G24810,AT1G16570,AT3G21240,AT3G47340,AT4G23820,AT2G44300,AT5G63860,AT1G75690,AT3G31980,AT1G26800,AT2G45690,AT5G63410,AT2G13020,AT4G03260,AT1G18720,AT1G72175,AT5G62090,AT3G15030,AT5G14090,AT5G34830,AT2G34620
CAATGATTG	10	1	['8-12', '12-16', '16-20', '20-24']	35	AT1G54850,AT1G15400,AT5G59300,AT1G14040,AT2G45420,AT5G08520,AT4G25450,AT4G15560,AT1G74640,AT1G07440,AT4G17460,AT4G24220,AT2G40060,AT1G18170,AT5G55480,AT5G18540,AT2G44310,AT1G51805,AT4G16860,AT2G21880,AT1G20190,AT1G23390,AT5G16910,AT1G08540,AT5G16260,AT3G13110,AT3G45630,AT1G25230,AT1G29490,AT2G45590,AT3G45230,AT5G52580,AT4G30660,AT1G53320,AT4G29840

CAATGATTG	10	2	['8-12', '16-20', '20-24', '12-16']	35	AT1G54850,AT1G15400,AT5G59300,AT1G14040,AT2G45420,AT5G08520,AT4G25450,AT4G15560,AT1G74640,AT1G07440,AT4G17460,AT4G24220,AT2G40060,AT1G18170,AT5G55480,AT5G18540,AT2G44310,AT1G51805,AT4G16860,AT2G21880,AT1G20190,AT1G23390,AT5G16910,AT1G08540,AT5G16260,AT3G13110,AT3G45630,AT1G25230,AT1G29490,AT2G45590,AT3G45230,AT5G52580,AT4G30660,AT1G53320,AT4G29840
CAATGATTG	10	3	['8-12', '12-16', '16-20', '20-24']	35	AT1G54850,AT1G15400,AT5G59300,AT1G14040,AT2G45420,AT5G08520,AT4G25450,AT4G15560,AT1G74640,AT1G07440,AT4G17460,AT4G24220,AT2G40060,AT1G18170,AT5G55480,AT5G18540,AT2G44310,AT1G51805,AT4G16860,AT2G21880,AT1G20190,AT1G23390,AT5G16910,AT1G08540,AT5G16260,AT3G13110,AT3G45630,AT1G25230,AT1G29490,AT2G45590,AT3G45230,AT5G52580,AT4G30660,AT1G53320,AT4G29840
CAATGATTG	10	4	['8-12', '12-16', '16-20', '20-24']	35	AT1G54850,AT1G15400,AT5G59300,AT1G14040,AT2G45420,AT5G08520,AT4G25450,AT4G15560,AT1G74640,AT1G07440,AT4G17460,AT4G24220,AT2G40060,AT1G18170,AT5G55480,AT5G18540,AT2G44310,AT1G51805,AT4G16860,AT2G21880,AT1G20190,AT1G23390,AT5G16910,AT1G08540,AT5G16260,AT3G13110,AT3G45630,AT1G25230,AT1G29490,AT2G45590,AT3G45230,AT5G52580,AT4G30660,AT1G53320,AT4G29840
CAATGATTG	10	5	['8-12', '12-16', '16-20', '20-24']	35	AT1G54850,AT1G15400,AT5G59300,AT1G14040,AT2G45420,AT5G08520,AT4G25450,AT4G15560,AT1G74640,AT1G07440,AT4G17460,AT4G24220,AT2G40060,AT1G18170,AT5G55480,AT5G18540,AT2G44310,AT1G51805,AT4G16860,AT2G21880,AT1G20190,AT1G23390,AT5G16910,AT1G08540,AT5G16260,AT3G13110,AT3G45630,AT1G25230,AT1G29490,AT2G45590,AT3G45230,AT5G52580,AT4G30660,AT1G53320,AT4G29840
CAATSATTG	10	1	['8-12', '12-16', '16-20']	72	AT1G19540,AT1G54850,AT1G19300,AT3G31980,AT5G60850,AT5G55480,AT5G38990,AT1G15400,AT4G39270,AT1G14990,AT1G50030,AT5G59300,AT1G14040,AT2G45420,AT5G08520,AT3G61430,AT3G61470,AT1G76100,AT3G27170,AT1G12990,AT5G57960,AT1G01620,AT4G25450,AT4G15560,AT1G74640,AT5G46020,AT3G05800,AT1G07440,AT4G24810,AT4G17460,AT4G24220,AT2G40060,AT1G18170,AT1G16570,AT3G47340,AT2G44310,AT5G18540,AT1G51805,AT2G44300,AT4G16860,AT1G75690,AT2G21880,AT3G21240,AT4G23820,AT1G23390,AT5G16910,AT5G63860,AT1G20190,AT1G26800,AT5G16260,AT2G45690,AT1G08540,AT5G63410,AT2G13020,AT4G03260,AT3G13110,AT3G45630,AT1G25230,AT1G29490,AT2G45590,AT1G18720,AT3G45230,AT1G72175,AT4G30660,AT5G52580,AT5G62090,AT3G15030,AT1G53320,AT5G14090,AT5G34830,AT4G29840,AT2G34620
CAATSATTG	10	2	['8-12', '16-20', '12-16']	72	AT1G19540,AT1G54850,AT1G19300,AT3G31980,AT5G60850,AT5G55480,AT5G38990,AT1G15400,AT4G39270,AT1G14990,AT1G50030,AT5G59300,AT1G14040,AT2G45420,AT5G08520,AT3G61430,AT3G61470,AT1G76100,AT3G27170,AT1G12990,AT5G57960,AT1G01620,AT4G25450,AT4G15560,AT1G74640,AT5G46020,AT3G05800,AT1G07440,AT4G24810,AT4G17460,AT4G24220,AT2G40060,AT1G18170,AT1G16570,AT3G47340,AT2G44310,AT5G18540,AT1G51805,AT2G44300,AT4G16860,AT1G75690,AT2G21880,AT3G21240,AT4G23820,AT1G23390,AT5G16910,AT5G63860,AT1G20190,AT1G26800,AT5G16260,AT2G45690,AT1G08540,AT5G63410,AT2G13020,AT4G03260,AT3G13110,AT3G45630,AT1G25230,AT1G29490,AT2G45590,AT1G18720,AT3G45230,AT1G72175,AT4G30660,AT5G52580,AT5G62090,AT3G15030,AT1G53320,AT5G14090,AT5G34830,AT4G29840,AT2G34620
CAATSATTG	10	3	['8-12', '12-16', '16-20']	72	AT1G19540,AT1G54850,AT1G19300,AT3G31980,AT5G60850,AT5G55480,AT5G38990,AT1G15400,AT4G39270,AT1G14990,AT1G50030,AT5G59300,AT1G14040,AT2G45420,AT5G08520,AT3G61430,AT3G61470,AT1G76100,AT3G27170,AT1G12990,AT5G57960,AT1G01620,AT4G25450,AT4G15560,AT1G74640,AT5G46020,AT3G05800,AT1G07440,AT4G24810,AT4G17460,AT4G24220,AT2G40060,AT1G18170,AT1G16570,AT3G47340,AT2G44310,AT5G18540,AT1G51805,AT2G44300,AT4G16860,AT1G75690,AT2G21880,AT3G21240,AT4G23820,AT1G23390,AT5G16910,AT5G63860,AT1G20190,AT1G26800,AT5G16260,AT2G45690,AT1G08540,AT5G63410,AT2G13020,AT4G03260,AT3G13110,AT3G45630,AT1G25230,AT1G29490,AT2G45590,AT1G18720,AT3G45230,AT1G72175,AT4G30660,AT5G52580,AT5G62090,AT3G15030,AT1G53320,AT5G14090,AT5G34830,AT4G29840,AT2G34620
CAATSATTG	10	4	['8-12', '12-16', '16-20']	72	AT1G19540,AT1G54850,AT1G19300,AT3G31980,AT5G60850,AT5G55480,AT5G38990,AT1G15400,AT4G39270,AT1G14990,AT1G50030,AT5G59300,AT1G14040,AT2G45420,AT5G08520,AT3G61430,AT3G61470,AT1G76100,AT3G27170,AT1G12990,AT5G57960,AT1G01620,AT4G25450,AT4G15560,AT1G74640,AT5G46020,AT3G05800,AT1G07440,AT4G24810,AT4G17460,AT4G24220,AT2G40060,AT1G18170,AT1G16570,AT3G47340,AT2G44310,AT5G18540,AT1G51805,AT2G44300,AT4G16860,AT1G75690,AT2G21880,AT3G21240,AT4G23820,AT1G23390,AT5G16910,AT5G63860,AT1G20190,AT1G26800,AT5G16260,AT2G45690,AT1G08540,AT5G63410,AT2G13020,AT4G03260,AT3G13110,AT3G45630,AT1G25230,AT1G29490,AT2G45590,AT1G18720,AT3G45230,AT1G72175,AT4G30660,AT5G52580,AT5G62090,AT3G15030,AT1G53320,AT5G14090,AT5G34830,AT4G29840,AT2G34620

CAATSATTG	10	5	['8-12', '12-16', '16-20', '0-4']	72	AT1G19540,AT1G54850,AT1G19300,AT3G31980,AT5G60850,AT5G55480,AT5G38990,AT1G15400,AT4G39270,AT1G14990,AT1G50030,AT5G59300,AT1G14040,AT2G45420,AT5G08520,AT3G61430,AT3G61470,AT1G76100,AT3G27170,AT1G12990,AT5G57960,AT1G01620,AT4G25450,AT4G15560,AT1G74640,AT5G46020,AT3G05800,AT1G07440,AT4G24810,AT4G17460,AT4G24220,AT2G40060,AT1G18170,AT1G16570,AT3G47340,AT2G44310,AT5G18540,AT1G51805,AT2G44300,AT4G16860,AT1G75690,AT2G21880,AT3G21240,AT4G23820,AT1G23390,AT5G16910,AT5G63860,AT1G20190,AT1G26800,AT5G16260,AT2G45690,AT1G08540,AT5G63410,AT2G13020,AT4G03260,AT3G13110,AT3G45630,AT1G25230,AT1G29490,AT2G45590,AT1G18720,AT3G45230,AT1G72175,AT4G30660,AT5G52580,AT5G62090,AT3G15030,AT1G53320,AT5G14090,AT5G34830,AT4G29840,AT2G34620
CAATTATTG	10	1	['16-20']	62	AT4G00955,AT4G30260,AT1G32520,AT4G28660,AT1G67510,AT4G00660,AT5G59540,AT3G09010,AT1G68000,AT4G38770,AT4G12730,AT5G09760,AT3G50950,AT1G75800,AT3G50830,AT3G26740,AT3G50500,AT1G52870,AT5G66880,AT4G17615,AT5G23730,AT1G22280,AT1G01620,AT1G09390,AT4G25770,AT1G80760,AT3G05880,AT4G34710,AT1G19110,AT1G64640,AT3G48650,AT5G65310,AT2G06980,AT1G64800,AT5G04490,AT1G21270,AT2G35260,AT2G26170,AT5G44910,AT1G70250,AT4G05320,AT1G52730,AT1G75780,AT2G36560,AT1G62300,AT3G57090,AT2G05620,AT2G30390,AT3G46620,AT1G68160,AT4G03110,AT4G14270,AT4G31820,AT5G03140,AT5G02880,AT5G14800,AT3G09390,AT1G07890,AT1G78970,AT2G31400,AT1G75240,AT2G45990
CAATTATTG	10	2	['16-20']	62	AT4G00955,AT4G30260,AT1G32520,AT4G28660,AT1G67510,AT4G00660,AT5G59540,AT3G09010,AT1G68000,AT4G38770,AT4G12730,AT5G09760,AT3G50950,AT1G75800,AT3G50830,AT3G26740,AT3G50500,AT1G52870,AT5G66880,AT4G17615,AT5G23730,AT1G22280,AT1G01620,AT1G09390,AT4G25770,AT1G80760,AT3G05880,AT4G34710,AT1G19110,AT1G64640,AT3G48650,AT5G65310,AT2G06980,AT1G64800,AT5G04490,AT1G21270,AT2G35260,AT2G26170,AT5G44910,AT1G70250,AT4G05320,AT1G52730,AT1G75780,AT2G36560,AT1G62300,AT3G57090,AT2G05620,AT2G30390,AT3G46620,AT1G68160,AT4G03110,AT4G14270,AT4G31820,AT5G03140,AT5G02880,AT5G14800,AT3G09390,AT1G07890,AT1G78970,AT2G31400,AT1G75240,AT2G45990
CAATTATTG	10	3	['16-20']	62	AT4G00955,AT4G30260,AT1G32520,AT4G28660,AT1G67510,AT4G00660,AT5G59540,AT3G09010,AT1G68000,AT4G38770,AT4G12730,AT5G09760,AT3G50950,AT1G75800,AT3G50830,AT3G26740,AT3G50500,AT1G52870,AT5G66880,AT4G17615,AT5G23730,AT1G22280,AT1G01620,AT1G09390,AT4G25770,AT1G80760,AT3G05880,AT4G34710,AT1G19110,AT1G64640,AT3G48650,AT5G65310,AT2G06980,AT1G64800,AT5G04490,AT1G21270,AT2G35260,AT2G26170,AT5G44910,AT1G70250,AT4G05320,AT1G52730,AT1G75780,AT2G36560,AT1G62300,AT3G57090,AT2G05620,AT2G30390,AT3G46620,AT1G68160,AT4G03110,AT4G14270,AT4G31820,AT5G03140,AT5G02880,AT5G14800,AT3G09390,AT1G07890,AT1G78970,AT2G31400,AT1G75240,AT2G45990
CAATTATTG	10	4	['16-20']	62	AT4G00955,AT4G30260,AT1G32520,AT4G28660,AT1G67510,AT4G00660,AT5G59540,AT3G09010,AT1G68000,AT4G38770,AT4G12730,AT5G09760,AT3G50950,AT1G75800,AT3G50830,AT3G26740,AT3G50500,AT1G52870,AT5G66880,AT4G17615,AT5G23730,AT1G22280,AT1G01620,AT1G09390,AT4G25770,AT1G80760,AT3G05880,AT4G34710,AT1G19110,AT1G64640,AT3G48650,AT5G65310,AT2G06980,AT1G64800,AT5G04490,AT1G21270,AT2G35260,AT2G26170,AT5G44910,AT1G70250,AT4G05320,AT1G52730,AT1G75780,AT2G36560,AT1G62300,AT3G57090,AT2G05620,AT2G30390,AT3G46620,AT1G68160,AT4G03110,AT4G14270,AT4G31820,AT5G03140,AT5G02880,AT5G14800,AT3G09390,AT1G07890,AT1G78970,AT2G31400,AT1G75240,AT2G45990
CAATTATTG	10	5	['16-20']	62	AT4G00955,AT4G30260,AT1G32520,AT4G28660,AT1G67510,AT4G00660,AT5G59540,AT3G09010,AT1G68000,AT4G38770,AT4G12730,AT5G09760,AT3G50950,AT1G75800,AT3G50830,AT3G26740,AT3G50500,AT1G52870,AT5G66880,AT4G17615,AT5G23730,AT1G22280,AT1G01620,AT1G09390,AT4G25770,AT1G80760,AT3G05880,AT4G34710,AT1G19110,AT1G64640,AT3G48650,AT5G65310,AT2G06980,AT1G64800,AT5G04490,AT1G21270,AT2G35260,AT2G26170,AT5G44910,AT1G70250,AT4G05320,AT1G52730,AT1G75780,AT2G36560,AT1G62300,AT3G57090,AT2G05620,AT2G30390,AT3G46620,AT1G68160,AT4G03110,AT4G14270,AT4G31820,AT5G03140,AT5G02880,AT5G14800,AT3G09390,AT1G07890,AT1G78970,AT2G31400,AT1G75240,AT2G45990

CACCAACC	10	1	['0-4']	100	AT2G40890,AT5G49910,AT1G06680,AT1G32060,AT1G75900,AT5G48380,AT5G48140,AT5G59920,AT2G46650,AT2G32280,AT4G16570,AT1G07020,AT1G64800,AT2G47780,AT1G18170,AT5G43430,AT1G30360,AT1G51080,AT3G42800,AT1G72710,AT2G26080,AT2G45740,AT4G39400,AT2G34490,AT5G19860,AT4G39235,AT5G20720,AT5G08330,AT2G31750,AT5G35970,AT1G52760,AT1G70700,AT4G35090,AT3G56910,AT5G22270,AT2G36880,AT4G33985,AT3G14090,AT2G37040,AT2G44310,AT2G44300,AT5G17990,AT5G17010,AT4G32530,AT5G15710,AT2G32720,AT5G15860,AT3G13100,AT4G31390,AT5G16310,AT2G22360,AT1G55850,AT5G34830,AT4G29510,AT1G54780,AT4G28540,AT3G10840,AT1G14990,AT4G27990,AT1G78240,AT1G70330,AT1G04140,AT4G27130,AT3G26740,AT5G07400,AT5G66340,AT5G65910,AT4G24930,AT1G04910,AT3G21240,AT5G03860,AT4G23100,AT5G03430,AT3G17930,AT5G03140,AT1G03550,AT1G07890,AT4G18270,AT3G62100,AT4G13430,AT3G06510,AT3G61440,AT3G61580,AT1G15520,AT5G58070,AT3G61080,AT2G43060,AT3G08570,AT1G15950,AT3G60020,AT4G08690,AT5G55620,AT3G58610,AT5G54970,AT5G54500,AT3G57250,AT4G02910,AT3G07350,AT1G49250,AT3G04790
CACCAACC	10	2	['0-4']	100	AT2G40890,AT5G49910,AT1G06680,AT1G32060,AT1G75900,AT5G48380,AT5G48140,AT5G59920,AT2G46650,AT2G32280,AT4G16570,AT1G07020,AT1G64800,AT2G47780,AT1G18170,AT5G43430,AT1G30360,AT1G51080,AT3G42800,AT1G72710,AT2G26080,AT2G45740,AT4G39400,AT2G34490,AT5G19860,AT4G39235,AT5G20720,AT5G08330,AT2G31750,AT5G35970,AT1G52760,AT1G70700,AT4G35090,AT3G56910,AT5G22270,AT2G36880,AT4G33985,AT3G14090,AT2G37040,AT2G44310,AT2G44300,AT5G17990,AT5G17010,AT4G32530,AT5G15710,AT2G32720,AT5G15860,AT3G13100,AT4G31390,AT5G16310,AT2G22360,AT1G55850,AT5G34830,AT4G29510,AT1G54780,AT4G28540,AT3G10840,AT1G14990,AT4G27990,AT1G78240,AT1G70330,AT1G04140,AT4G27130,AT3G26740,AT5G07400,AT5G66340,AT5G65910,AT4G24930,AT1G04910,AT3G21240,AT5G03860,AT4G23100,AT5G03430,AT3G17930,AT5G03140,AT1G03550,AT1G07890,AT4G18270,AT3G62100,AT4G13430,AT3G06510,AT3G61440,AT3G61580,AT1G15520,AT5G58070,AT3G61080,AT2G43060,AT3G08570,AT1G15950,AT3G60020,AT4G08690,AT5G55620,AT3G58610,AT5G54970,AT5G54500,AT3G57250,AT4G02910,AT3G07350,AT1G49250,AT3G04790
CACCAACC	10	3	['0-4']	100	AT2G40890,AT5G49910,AT1G06680,AT1G32060,AT1G75900,AT5G48380,AT5G48140,AT5G59920,AT2G46650,AT2G32280,AT4G16570,AT1G07020,AT1G64800,AT2G47780,AT1G18170,AT5G43430,AT1G30360,AT1G51080,AT3G42800,AT1G72710,AT2G26080,AT2G45740,AT4G39400,AT2G34490,AT5G19860,AT4G39235,AT5G20720,AT5G08330,AT2G31750,AT5G35970,AT1G52760,AT1G70700,AT4G35090,AT3G56910,AT5G22270,AT2G36880,AT4G33985,AT3G14090,AT2G37040,AT2G44310,AT2G44300,AT5G17990,AT5G17010,AT4G32530,AT5G15710,AT2G32720,AT5G15860,AT3G13100,AT4G31390,AT5G16310,AT2G22360,AT1G55850,AT5G34830,AT4G29510,AT1G54780,AT4G28540,AT3G10840,AT1G14990,AT4G27990,AT1G78240,AT1G70330,AT1G04140,AT4G27130,AT3G26740,AT5G07400,AT5G66340,AT5G65910,AT4G24930,AT1G04910,AT3G21240,AT5G03860,AT4G23100,AT5G03430,AT3G17930,AT5G03140,AT1G03550,AT1G07890,AT4G18270,AT3G62100,AT4G13430,AT3G06510,AT3G61440,AT3G61580,AT1G15520,AT5G58070,AT3G61080,AT2G43060,AT3G08570,AT1G15950,AT3G60020,AT4G08690,AT5G55620,AT3G58610,AT5G54970,AT5G54500,AT3G57250,AT4G02910,AT3G07350,AT1G49250,AT3G04790
CACCAACC	10	4	['0-4']	100	AT2G40890,AT5G49910,AT1G06680,AT1G32060,AT1G75900,AT5G48380,AT5G48140,AT5G59920,AT2G46650,AT2G32280,AT4G16570,AT1G07020,AT1G64800,AT2G47780,AT1G18170,AT5G43430,AT1G30360,AT1G51080,AT3G42800,AT1G72710,AT2G26080,AT2G45740,AT4G39400,AT2G34490,AT5G19860,AT4G39235,AT5G20720,AT5G08330,AT2G31750,AT5G35970,AT1G52760,AT1G70700,AT4G35090,AT3G56910,AT5G22270,AT2G36880,AT4G33985,AT3G14090,AT2G37040,AT2G44310,AT2G44300,AT5G17990,AT5G17010,AT4G32530,AT5G15710,AT2G32720,AT5G15860,AT3G13100,AT4G31390,AT5G16310,AT2G22360,AT1G55850,AT5G34830,AT4G29510,AT1G54780,AT4G28540,AT3G10840,AT1G14990,AT4G27990,AT1G78240,AT1G70330,AT1G04140,AT4G27130,AT3G26740,AT5G07400,AT5G66340,AT5G65910,AT4G24930,AT1G04910,AT3G21240,AT5G03860,AT4G23100,AT5G03430,AT3G17930,AT5G03140,AT1G03550,AT1G07890,AT4G18270,AT3G62100,AT4G13430,AT3G06510,AT3G61440,AT3G61580,AT1G15520,AT5G58070,AT3G61080,AT2G43060,AT3G08570,AT1G15950,AT3G60020,AT4G08690,AT5G55620,AT3G58610,AT5G54970,AT5G54500,AT3G57250,AT4G02910,AT3G07350,AT1G49250,AT3G04790

CACCAACC	10	5	['0-4']	100	AT2G40890,AT5G49910,AT1G06680,AT1G32060,AT1G75900,AT5G48380,AT5G48140,AT5G59920,AT2G46650,AT2G32280,AT4G16570,AT1G07020,AT1G64800,AT2G47780,AT1G18170,AT5G43430,AT1G30360,AT1G51080,AT3G42800,AT1G72710,AT2G26080,AT2G45740,AT4G39400,AT2G34490,AT5G19860,AT4G39235,AT5G20720,AT5G08330,AT2G31750,AT5G35970,AT1G52760,AT1G70700,AT4G35090,AT3G56910,AT5G22270,AT2G36880,AT4G33985,AT3G14090,AT2G37040,AT2G44310,AT2G44300,AT5G17990,AT5G17010,AT4G32530,AT5G15710,AT2G32720,AT5G15860,AT3G13100,AT4G31390,AT5G16310,AT2G22360,AT1G55850,AT5G34830,AT4G29510,AT1G54780,AT4G28540,AT3G10840,AT1G14990,AT4G27990,AT1G78240,AT1G70330,AT1G04140,AT4G27130,AT3G26740,AT5G07400,AT5G66340,AT5G65910,AT4G24930,AT1G04910,AT3G21240,AT5G03860,AT4G23100,AT5G03430,AT3G17930,AT5G03140,AT1G03550,AT1G07890,AT4G18270,AT3G62100,AT4G13430,AT3G06510,AT3G61440,AT3G61580,AT1G15520,AT5G58070,AT3G61080,AT2G43060,AT3G08570,AT1G15950,AT3G60020,AT4G08690,AT5G55620,AT3G58610,AT5G54970,AT5G54500,AT3G57250,AT4G02910,AT3G07350,AT1G49250,AT3G04790
CACCTACC	10	1	['4-8', '8-12', '12-16', '16-20', '20-24']	42	AT5G50990,AT5G27380,AT1G24575,AT4G28460,AT2G26460,AT3G02468,AT4G27470,AT4G12320,AT3G52340,AT2G30860,AT1G10370,AT2G30900,AT1G22360,AT5G47870,AT4G09570,AT4G08690,AT3G51820,AT5G64860,AT3G47960,AT5G22300,AT1G09960,AT3G03160,AT1G69880,AT3G06350,AT3G24590,AT4G22300,AT2G41870,AT5G63370,AT2G32720,AT2G41760,AT5G02820,AT5G53170,AT1G54880,AT5G62200,AT1G47530,AT2G47010,AT5G41050,AT5G35180,AT2G25930,AT3G26570,AT5G13650,AT1G65430
CACCTACC	10	2	['4-8', '8-12', '20-24', '16-20', '12-16']	42	AT5G50990,AT5G27380,AT1G24575,AT4G28460,AT2G26460,AT3G02468,AT4G27470,AT4G12320,AT3G52340,AT2G30860,AT1G10370,AT2G30900,AT1G22360,AT5G47870,AT4G09570,AT4G08690,AT3G51820,AT5G64860,AT3G47960,AT5G22300,AT1G09960,AT3G03160,AT1G69880,AT3G06350,AT3G24590,AT4G22300,AT2G41870,AT5G63370,AT2G32720,AT2G41760,AT5G02820,AT5G53170,AT1G54880,AT5G62200,AT1G47530,AT2G47010,AT5G41050,AT5G35180,AT2G25930,AT3G26570,AT5G13650,AT1G65430
CACCTACC	10	3	['4-8', '8-12', '20-24', '12-16', '16-20']	42	AT5G50990,AT5G27380,AT1G24575,AT4G28460,AT2G26460,AT3G02468,AT4G27470,AT4G12320,AT3G52340,AT2G30860,AT1G10370,AT2G30900,AT1G22360,AT5G47870,AT4G09570,AT4G08690,AT3G51820,AT5G64860,AT3G47960,AT5G22300,AT1G09960,AT3G03160,AT1G69880,AT3G06350,AT3G24590,AT4G22300,AT2G41870,AT5G63370,AT2G32720,AT2G41760,AT5G02820,AT5G53170,AT1G54880,AT5G62200,AT1G47530,AT2G47010,AT5G41050,AT5G35180,AT2G25930,AT3G26570,AT5G13650,AT1G65430
CACCTACC	10	4	['4-8', '8-12', '16-20', '20-24']	42	AT5G50990,AT5G27380,AT1G24575,AT4G28460,AT2G26460,AT3G02468,AT4G27470,AT4G12320,AT3G52340,AT2G30860,AT1G10370,AT2G30900,AT1G22360,AT5G47870,AT4G09570,AT4G08690,AT3G51820,AT5G64860,AT3G47960,AT5G22300,AT1G09960,AT3G03160,AT1G69880,AT3G06350,AT3G24590,AT4G22300,AT2G41870,AT5G63370,AT2G32720,AT2G41760,AT5G02820,AT5G53170,AT1G54880,AT5G62200,AT1G47530,AT2G47010,AT5G41050,AT5G35180,AT2G25930,AT3G26570,AT5G13650,AT1G65430
CACCTACC	10	5	['4-8', '8-12', '12-16', '20-24', '16-20']	42	AT5G50990,AT5G27380,AT1G24575,AT4G28460,AT2G26460,AT3G02468,AT4G27470,AT4G12320,AT3G52340,AT2G30860,AT1G10370,AT2G30900,AT1G22360,AT5G47870,AT4G09570,AT4G08690,AT3G51820,AT5G64860,AT3G47960,AT5G22300,AT1G09960,AT3G03160,AT1G69880,AT3G06350,AT3G24590,AT4G22300,AT2G41870,AT5G63370,AT2G32720,AT2G41760,AT5G02820,AT5G53170,AT1G54880,AT5G62200,AT1G47530,AT2G47010,AT5G41050,AT5G35180,AT2G25930,AT3G26570,AT5G13650,AT1G65430
CACGCAAT	10	1	['8-12', '12-16']	33	AT3G63060,AT1G75210,AT2G11830,AT4G38570,AT5G49120,AT3G61470,AT1G13270,AT1G73177,AT1G53540,AT2G46650,AT4G25700,AT5G47870,AT4G14960,AT2G38610,AT2G21260,AT5G56260,AT1G04820,AT3G47860,AT2G26170,AT3G23580,AT3G05670,AT2G30490,AT3G18215,AT1G08540,AT5G63620,AT1G72430,AT5G15090,AT3G54730,AT1G03550,AT5G14640,AT5G62090,AT2G35620,AT3G04790
CACGCAAT	10	2	['8-12', '12-16']	33	AT3G63060,AT1G75210,AT2G11830,AT4G38570,AT5G49120,AT3G61470,AT1G13270,AT1G73177,AT1G53540,AT2G46650,AT4G25700,AT5G47870,AT4G14960,AT2G38610,AT2G21260,AT5G56260,AT1G04820,AT3G47860,AT2G26170,AT3G23580,AT3G05670,AT2G30490,AT3G18215,AT1G08540,AT5G63620,AT1G72430,AT5G15090,AT3G54730,AT1G03550,AT5G14640,AT5G62090,AT2G35620,AT3G04790

CACGCAAT	10	3	['8-12', '12-16']	33	AT3G63060,AT1G75210,AT2G11830,AT4G38570,AT5G49120,AT3G61470,AT1G13270,AT1G73177,AT1G53540,AT2G46650,AT4G25700,AT5G47870,AT4G14960,AT2G38610,AT2G21260,AT5G56260,AT1G04820,AT3G47860,AT2G26170,AT3G23580,AT3G05670,AT2G30490,AT3G18215,AT1G08540,AT5G63620,AT1G72430,AT5G15090,AT3G54730,AT1G03550,AT5G14640,AT5G62090,AT2G35620,AT3G04790
CACGCAAT	10	4	['8-12', '12-16']	33	AT3G63060,AT1G75210,AT2G11830,AT4G38570,AT5G49120,AT3G61470,AT1G13270,AT1G73177,AT1G53540,AT2G46650,AT4G25700,AT5G47870,AT4G14960,AT2G38610,AT2G21260,AT5G56260,AT1G04820,AT3G47860,AT2G26170,AT3G23580,AT3G05670,AT2G30490,AT3G18215,AT1G08540,AT5G63620,AT1G72430,AT5G15090,AT3G54730,AT1G03550,AT5G14640,AT5G62090,AT2G35620,AT3G04790
CACGCAAT	10	5	['8-12', '12-16']	33	AT3G63060,AT1G75210,AT2G11830,AT4G38570,AT5G49120,AT3G61470,AT1G13270,AT1G73177,AT1G53540,AT2G46650,AT4G25700,AT5G47870,AT4G14960,AT2G38610,AT2G21260,AT5G56260,AT1G04820,AT3G47860,AT2G26170,AT3G23580,AT3G05670,AT2G30490,AT3G18215,AT1G08540,AT5G63620,AT1G72430,AT5G15090,AT3G54730,AT1G03550,AT5G14640,AT5G62090,AT2G35620,AT3G04790
CACGTGGC	10	1	['4-8']	94	AT1G06680,AT2G38000,AT5G49120,AT3G05130,AT2G47490,AT1G13440,AT1G28530,AT4G25570,AT5G47640,AT1G22370,AT1G01520,AT4G16515,AT2G47780,AT1G56220,AT5G43850,AT3G12470,AT4G14270,AT1G52220,AT1G52230,AT1G68440,AT2G45990,AT5G39570,AT1G75210,AT2G34460,AT5G35460,AT3G29320,AT4G35850,AT5G23050,AT2G21330,AT3G15210,AT2G04550,AT5G18340,AT2G36390,AT1G10960,AT2G47400,AT4G31310,AT1G55850,ATMG01330,AT5G27520,AT4G28750,AT1G54850,AT1G32470,AT5G67370,AT3G12920,AT5G66570,AT1G01240,AT4G25450,AT3G25530,AT5G65630,AT1G55670,AT5G05270,AT5G05300,AT1G19660,AT5G64840,AT5G05200,AT4G24190,AT5G64260,AT1G09870,AT5G64180,AT1G22850,AT3G18110,AT5G03190,AT1G53090,AT3G13980,AT4G19710,AT1G18740,AT5G61530,AT3G63060,AT4G18240,AT3G63210,AT3G06780,AT1G18310,AT1G55480,AT5G59080,AT5G58770,AT5G58070,AT5G57760,AT5G57345,AT3G60200,AT4G09620,AT3G59660,AT4G09020,AT3G59220,AT4G08180,AT2G15970,AT3G03150,AT1G74840,AT4G03560,AT3G10410,AT3G56050,AT2G01290,AT5G52580,AT3G04860,AT5G51110
CACGTGGC	10	2	['4-8']	94	AT1G06680,AT2G38000,AT5G49120,AT3G05130,AT2G47490,AT1G13440,AT1G28530,AT4G25570,AT5G47640,AT1G22370,AT1G01520,AT4G16515,AT2G47780,AT1G56220,AT5G43850,AT3G12470,AT4G14270,AT1G52220,AT1G52230,AT1G68440,AT2G45990,AT5G39570,AT1G75210,AT2G34460,AT5G35460,AT3G29320,AT4G35850,AT5G23050,AT2G21330,AT3G15210,AT2G04550,AT5G18340,AT2G36390,AT1G10960,AT2G47400,AT4G31310,AT1G55850,ATMG01330,AT5G27520,AT4G28750,AT1G54850,AT1G32470,AT5G67370,AT3G12920,AT5G66570,AT1G01240,AT4G25450,AT3G25530,AT5G65630,AT1G55670,AT5G05270,AT5G05300,AT1G19660,AT5G64840,AT5G05200,AT4G24190,AT5G64260,AT1G09870,AT5G64180,AT1G22850,AT3G18110,AT5G03190,AT1G53090,AT3G13980,AT4G19710,AT1G18740,AT5G61530,AT3G63060,AT4G18240,AT3G63210,AT3G06780,AT1G18310,AT1G55480,AT5G59080,AT5G58770,AT5G58070,AT5G57760,AT5G57345,AT3G60200,AT4G09620,AT3G59660,AT4G09020,AT3G59220,AT4G08180,AT2G15970,AT3G03150,AT1G74840,AT4G03560,AT3G10410,AT3G56050,AT2G01290,AT5G52580,AT3G04860,AT5G51110
CACGTGGC	10	3	['4-8']	94	AT1G06680,AT2G38000,AT5G49120,AT3G05130,AT2G47490,AT1G13440,AT1G28530,AT4G25570,AT5G47640,AT1G22370,AT1G01520,AT4G16515,AT2G47780,AT1G56220,AT5G43850,AT3G12470,AT4G14270,AT1G52220,AT1G52230,AT1G68440,AT2G45990,AT5G39570,AT1G75210,AT2G34460,AT5G35460,AT3G29320,AT4G35850,AT5G23050,AT2G21330,AT3G15210,AT2G04550,AT5G18340,AT2G36390,AT1G10960,AT2G47400,AT4G31310,AT1G55850,ATMG01330,AT5G27520,AT4G28750,AT1G54850,AT1G32470,AT5G67370,AT3G12920,AT5G66570,AT1G01240,AT4G25450,AT3G25530,AT5G65630,AT1G55670,AT5G05270,AT5G05300,AT1G19660,AT5G64840,AT5G05200,AT4G24190,AT5G64260,AT1G09870,AT5G64180,AT1G22850,AT3G18110,AT5G03190,AT1G53090,AT3G13980,AT4G19710,AT1G18740,AT5G61530,AT3G63060,AT4G18240,AT3G63210,AT3G06780,AT1G18310,AT1G55480,AT5G59080,AT5G58770,AT5G58070,AT5G57760,AT5G57345,AT3G60200,AT4G09620,AT3G59660,AT4G09020,AT3G59220,AT4G08180,AT2G15970,AT3G03150,AT1G74840,AT4G03560,AT3G10410,AT3G56050,AT2G01290,AT5G52580,AT3G04860,AT5G51110

CACGTGGC	10	4	['4-8']	94	AT1G06680,AT2G38000,AT5G49120,AT3G05130,AT2G47490,AT1G13440,AT1G28530,AT4G25570,AT5G47640,AT1G22370,AT1G01520,AT4G16515,AT2G47780,AT1G56220,AT5G43850,AT3G12470,AT4G14270,AT1G52220,AT1G52230,AT1G68440,AT2G45990,AT5G39570,AT1G75210,AT2G34460,AT5G35460,AT3G29320,AT4G35850,AT5G23050,AT2G21330,AT3G15210,AT2G04550,AT5G18340,AT2G36390,AT1G10960,AT2G47400,AT4G31310,AT1G55850,ATMG01330,AT5G27520,AT4G28750,AT1G54850,AT1G32470,AT5G67370,AT3G12920,AT5G66570,AT1G01240,AT4G25450,AT3G25530,AT5G65630,AT1G55670,AT5G05270,AT5G05300,AT1G19660,AT5G64840,AT5G05200,AT4G24190,AT5G64260,AT1G09870,AT5G64180,AT1G22850,AT3G18110,AT5G03190,AT1G53090,AT3G13980,AT4G19710,AT1G18740,AT5G61530,AT3G63060,AT4G18240,AT3G63210,AT3G06780,AT1G18310,AT1G55480,AT5G59080,AT5G58770,AT5G58070,AT5G57760,AT5G57345,AT3G60200,AT4G09620,AT3G59660,AT4G09020,AT3G59220,AT4G08180,AT2G15970,AT3G03150,AT1G74840,AT4G03560,AT3G10410,AT3G56050,AT2G01290,AT5G52580,AT3G04860,AT5G51110
CACGTGGC	10	5	['4-8']	94	AT1G06680,AT2G38000,AT5G49120,AT3G05130,AT2G47490,AT1G13440,AT1G28530,AT4G25570,AT5G47640,AT1G22370,AT1G01520,AT4G16515,AT2G47780,AT1G56220,AT5G43850,AT3G12470,AT4G14270,AT1G52220,AT1G52230,AT1G68440,AT2G45990,AT5G39570,AT1G75210,AT2G34460,AT5G35460,AT3G29320,AT4G35850,AT5G23050,AT2G21330,AT3G15210,AT2G04550,AT5G18340,AT2G36390,AT1G10960,AT2G47400,AT4G31310,AT1G55850,ATMG01330,AT5G27520,AT4G28750,AT1G54850,AT1G32470,AT5G67370,AT3G12920,AT5G66570,AT1G01240,AT4G25450,AT3G25530,AT5G65630,AT1G55670,AT5G05270,AT5G05300,AT1G19660,AT5G64840,AT5G05200,AT4G24190,AT5G64260,AT1G09870,AT5G64180,AT1G22850,AT3G18110,AT5G03190,AT1G53090,AT3G13980,AT4G19710,AT1G18740,AT5G61530,AT3G63060,AT4G18240,AT3G63210,AT3G06780,AT1G18310,AT1G55480,AT5G59080,AT5G58770,AT5G58070,AT5G57760,AT5G57345,AT3G60200,AT4G09620,AT3G59660,AT4G09020,AT3G59220,AT4G08180,AT2G15970,AT3G03150,AT1G74840,AT4G03560,AT3G10410,AT3G56050,AT2G01290,AT5G52580,AT3G04860,AT5G51110
CAGAAGATA	10	1	['16-20', '20-24']	44	AT1G31330,AT3G10230,AT3G16560,AT5G11280,AT1G33270,AT1G78290,AT5G13090,AT3G32280,AT2G33250,AT1G29260,AT1G16520,AT1G21500,AT1G16470,AT1G29240,AT2G25080,AT3G27170,AT4G17615,AT4G16570,AT3G59660,AT2G41330,AT1G15740,AT1G65490,AT4G34100,AT3G58390,AT5G19010,AT2G40080,AT5G04810,AT1G70250,AT4G16860,AT2G33740,AT1G42970,AT3G57090,AT3G47160,AT1G05920,AT2G45990,AT1G26795,AT1G08550,AT3G45780,AT4G21210,AT5G42100,AT4G19670,AT3G07350,AT5G28140,AT3G15480
CAGAAGATA	10	2	['16-20', '20-24']	44	AT1G31330,AT3G10230,AT3G16560,AT5G11280,AT1G33270,AT1G78290,AT5G13090,AT3G32280,AT2G33250,AT1G29260,AT1G16520,AT1G21500,AT1G16470,AT1G29240,AT2G25080,AT3G27170,AT4G17615,AT4G16570,AT3G59660,AT2G41330,AT1G15740,AT1G65490,AT4G34100,AT3G58390,AT5G19010,AT2G40080,AT5G04810,AT1G70250,AT4G16860,AT2G33740,AT1G42970,AT3G57090,AT3G47160,AT1G05920,AT2G45990,AT1G26795,AT1G08550,AT3G45780,AT4G21210,AT5G42100,AT4G19670,AT3G07350,AT5G28140,AT3G15480
CAGAAGATA	10	3	['16-20', '20-24']	44	AT1G31330,AT3G10230,AT3G16560,AT5G11280,AT1G33270,AT1G78290,AT5G13090,AT3G32280,AT2G33250,AT1G29260,AT1G16520,AT1G21500,AT1G16470,AT1G29240,AT2G25080,AT3G27170,AT4G17615,AT4G16570,AT3G59660,AT2G41330,AT1G15740,AT1G65490,AT4G34100,AT3G58390,AT5G19010,AT2G40080,AT5G04810,AT1G70250,AT4G16860,AT2G33740,AT1G42970,AT3G57090,AT3G47160,AT1G05920,AT2G45990,AT1G26795,AT1G08550,AT3G45780,AT4G21210,AT5G42100,AT4G19670,AT3G07350,AT5G28140,AT3G15480
CAGAAGATA	10	4	['16-20', '20-24']	44	AT1G31330,AT3G10230,AT3G16560,AT5G11280,AT1G33270,AT1G78290,AT5G13090,AT3G32280,AT2G33250,AT1G29260,AT1G16520,AT1G21500,AT1G16470,AT1G29240,AT2G25080,AT3G27170,AT4G17615,AT4G16570,AT3G59660,AT2G41330,AT1G15740,AT1G65490,AT4G34100,AT3G58390,AT5G19010,AT2G40080,AT5G04810,AT1G70250,AT4G16860,AT2G33740,AT1G42970,AT3G57090,AT3G47160,AT1G05920,AT2G45990,AT1G26795,AT1G08550,AT3G45780,AT4G21210,AT5G42100,AT4G19670,AT3G07350,AT5G28140,AT3G15480

CAGAAGATA	10	5	['16-20', '20-24']	44	AT1G31330,AT3G10230,AT3G16560,AT5G11280,AT1G33270,AT1G78290,AT5G13090,AT3G32280,AT2G33250,AT1G29260,AT1G16520,AT1G21500,AT1G16470,AT1G29240,AT2G25080,AT3G27170,AT4G17615,AT4G16570,AT3G59660,AT2G41330,AT1G15740,AT1G65490,AT4G34100,AT3G58390,AT5G19010,AT2G40080,AT5G04810,AT1G70250,AT4G16860,AT2G33740,AT1G42970,AT3G57090,AT3G47160,AT1G05920,AT2G45990,AT1G26795,AT1G08550,AT3G45780,AT4G21210,AT5G42100,AT4G19670,AT3G07350,AT5G28140,AT3G15480
CAGATCNG	10	1	['12-16']	162	AT4G00955,AT3G53720,AT4G00730,AT3G10200,AT3G53470,AT3G01490,AT1G15100,AT1G14150,AT2G40935,AT1G06470,AT5G49540,AT3G53030,AT3G52150,AT5G49280,AT3G07770,AT2G29560,AT3G50500,AT3G50860,AT3G50240,AT1G11630,AT1G58360,AT1G07010,AT2G35190,AT1G20696,AT4G16330,AT2G47690,AT1G42970,AT3G47560,AT1G30200,AT3G46970,AT1G56290,AT1G63080,AT1G03130,AT3G46340,AT5G42470,AT3G45630,AT1G25230,AT1G78600,AT1G78450,AT1G72030,AT5G41760,AT2G25930,AT2G31360,AT4G40030,AT2G15290,AT2G03890,AT2G39480,AT2G13360,AT1G33270,AT4G38710,AT4G38570,AT2G41080,AT2G31670,AT2G41090,AT4G38460,AT5G19420,AT5G35970,AT2G42590,AT5G20470,AT5G35460,AT5G24620,AT1G67623,AT1G09340,AT4G36640,AT1G74370,AT4G36580,AT2G21540,AT5G19240,AT3G13430,AT4G34220,AT5G22270,AT1G51940,AT1G69390,AT1G69210,AT1G69830,AT5G16290,AT2G41760,AT2G41720,AT2G22360,AT2G45590,AT3G14900,AT3G15040,AT2G32880,AT5G13730,AT4G29670,AT5G11890,AT4G29260,AT1G19360,AT1G32310,AT5G11480,AT1G09230,AT1G78240,AT5G25070,AT5G24970,AT1G21500,AT5G67200,AT5G08100,AT5G08120,AT1G11860,AT3G26630,AT1G26590,AT5G06970,AT5G66410,AT5G06870,AT5G65630,AT1G55670,AT5G05740,AT1G04530,AT1G62960,AT4G24460,AT5G04810,AT5G63860,AT3G23580,AT3G24590,AT1G08640,AT3G21650,AT1G18740,AT4G19185,AT1G23205,AT1G14280,AT1G28410,AT3G16170,AT3G15480,AT4G18520,AT3G63310,AT1G49580,AT3G62580,AT1G50120,AT5G59540,AT1G01940,AT3G62280,AT1G18330,AT1G03900,AT5G59140,AT3G62370,AT5G58870,AT3G02750,AT3G02830,AT4G12010,AT5G58380,AT1G05030,AT3G60750,AT5G57930,AT5G57290,AT3G09750,AT3G59060,AT3G10850,AT2G46570,AT4G05320,AT2G15570,AT2G15520,AT3G31980,AT3G01440,AT4G03600,AT3G10690,AT2G48020,AT4G02630,AT4G01940,AT3G05000,AT1G21680,AT5G51390,AT1G17160
CAGATCNG	10	2	['12-16']	162	AT4G00955,AT3G53720,AT4G00730,AT3G10200,AT3G53470,AT3G01490,AT1G15100,AT1G14150,AT2G40935,AT1G06470,AT5G49540,AT3G53030,AT3G52150,AT5G49280,AT3G07770,AT2G29560,AT3G50500,AT3G50860,AT3G50240,AT1G11630,AT1G58360,AT1G07010,AT2G35190,AT1G20696,AT4G16330,AT2G47690,AT1G42970,AT3G47560,AT1G30200,AT3G46970,AT1G56290,AT1G63080,AT1G03130,AT3G46340,AT5G42470,AT3G45630,AT1G25230,AT1G78600,AT1G78450,AT1G72030,AT5G41760,AT2G25930,AT2G31360,AT4G40030,AT2G15290,AT2G03890,AT2G39480,AT2G13360,AT1G33270,AT4G38710,AT4G38570,AT2G41080,AT2G31670,AT2G41090,AT4G38460,AT5G19420,AT5G35970,AT2G42590,AT5G20470,AT5G35460,AT5G24620,AT1G67623,AT1G09340,AT4G36640,AT1G74370,AT4G36580,AT2G21540,AT5G19240,AT3G13430,AT4G34220,AT5G22270,AT1G51940,AT1G69390,AT1G69210,AT1G69830,AT5G16290,AT2G41760,AT2G41720,AT2G22360,AT2G45590,AT3G14900,AT3G15040,AT2G32880,AT5G13730,AT4G29670,AT5G11890,AT4G29260,AT1G19360,AT1G32310,AT5G11480,AT1G09230,AT1G78240,AT5G25070,AT5G24970,AT1G21500,AT5G67200,AT5G08100,AT5G08120,AT1G11860,AT3G26630,AT1G26590,AT5G06970,AT5G66410,AT5G06870,AT5G65630,AT1G55670,AT5G05740,AT1G04530,AT1G62960,AT4G24460,AT5G04810,AT5G63860,AT3G23580,AT3G24590,AT1G08640,AT3G21650,AT1G18740,AT4G19185,AT1G23205,AT1G14280,AT1G28410,AT3G16170,AT3G15480,AT4G18520,AT3G63310,AT1G49580,AT3G62580,AT1G50120,AT5G59540,AT1G01940,AT3G62280,AT1G18330,AT1G03900,AT5G59140,AT3G62370,AT5G58870,AT3G02750,AT3G02830,AT4G12010,AT5G58380,AT1G05030,AT3G60750,AT5G57930,AT5G57290,AT3G09750,AT3G59060,AT3G10850,AT2G46570,AT4G05320,AT2G15570,AT2G15520,AT3G31980,AT3G01440,AT4G03600,AT3G10690,AT2G48020,AT4G02630,AT4G01940,AT3G05000,AT1G21680,AT5G51390,AT1G17160

CAGATCNG	10	3	['12-16']	162	AT4G00955,AT3G53720,AT4G00730,AT3G10200,AT3G53470,AT3G01490,AT1G15100,AT1G14150,AT2G40935,AT1G06470,AT5G49540,AT3G53030,AT3G52150,AT5G49280,AT3G07770,AT2G29560,AT3G50500,AT3G50860,AT3G50240,AT1G11630,AT1G58360,AT1G07010,AT2G35190,AT1G20696,AT4G16330,AT2G47690,AT1G42970,AT3G47560,AT1G30200,AT3G46970,AT1G56290,AT1G63080,AT1G03130,AT3G46340,AT5G42470,AT3G45630,AT1G25230,AT1G78600,AT1G78450,AT1G72030,AT5G41760,AT2G25930,AT2G31360,AT4G40030,AT2G15290,AT2G03890,AT2G39480,AT2G13360,AT1G33270,AT4G38710,AT4G38570,AT2G41080,AT2G31670,AT2G41090,AT4G38460,AT5G19420,AT5G35970,AT2G42590,AT5G20470,AT5G35460,AT5G24620,AT1G67623,AT1G09340,AT4G36640,AT1G74370,AT4G36580,AT2G21540,AT5G19240,AT3G13430,AT4G34220,AT5G22270,AT1G51940,AT1G69390,AT1G69210,AT1G69830,AT5G16290,AT2G41760,AT2G41720,AT2G22360,AT2G45590,AT3G14900,AT3G15040,AT2G32880,AT5G13730,AT4G29670,AT5G11890,AT4G29260,AT1G19360,AT1G32310,AT5G11480,AT1G09230,AT1G78240,AT5G25070,AT5G24970,AT1G21500,AT5G67200,AT5G08100,AT5G08120,AT1G11860,AT3G26630,AT1G26590,AT5G06970,AT5G66410,AT5G06870,AT5G65630,AT1G55670,AT5G05740,AT1G04530,AT1G62960,AT4G24460,AT5G04810,AT5G63860,AT3G23580,AT3G24590,AT1G08640,AT3G21650,AT1G18740,AT4G19185,AT1G23205,AT1G14280,AT1G28410,AT3G16170,AT3G15480,AT4G18520,AT3G63310,AT1G49580,AT3G62580,AT1G50120,AT5G59540,AT1G01940,AT3G62280,AT1G18330,AT1G03900,AT5G59140,AT3G62370,AT5G58870,AT3G02750,AT3G02830,AT4G12010,AT5G58380,AT1G05030,AT3G60750,AT5G57930,AT5G57290,AT3G09750,AT3G59060,AT3G10850,AT2G46570,AT4G05320,AT2G15570,AT2G15520,AT3G31980,AT3G01440,AT4G03600,AT3G10690,AT2G48020,AT4G02630,AT4G01940,AT3G05000,AT1G21680,AT5G51390,AT1G17160
CAGATCNG	10	4	['12-16']	162	AT4G00955,AT3G53720,AT4G00730,AT3G10200,AT3G53470,AT3G01490,AT1G15100,AT1G14150,AT2G40935,AT1G06470,AT5G49540,AT3G53030,AT3G52150,AT5G49280,AT3G07770,AT2G29560,AT3G50500,AT3G50860,AT3G50240,AT1G11630,AT1G58360,AT1G07010,AT2G35190,AT1G20696,AT4G16330,AT2G47690,AT1G42970,AT3G47560,AT1G30200,AT3G46970,AT1G56290,AT1G63080,AT1G03130,AT3G46340,AT5G42470,AT3G45630,AT1G25230,AT1G78600,AT1G78450,AT1G72030,AT5G41760,AT2G25930,AT2G31360,AT4G40030,AT2G15290,AT2G03890,AT2G39480,AT2G13360,AT1G33270,AT4G38710,AT4G38570,AT2G41080,AT2G31670,AT2G41090,AT4G38460,AT5G19420,AT5G35970,AT2G42590,AT5G20470,AT5G35460,AT5G24620,AT1G67623,AT1G09340,AT4G36640,AT1G74370,AT4G36580,AT2G21540,AT5G19240,AT3G13430,AT4G34220,AT5G22270,AT1G51940,AT1G69390,AT1G69210,AT1G69830,AT5G16290,AT2G41760,AT2G41720,AT2G22360,AT2G45590,AT3G14900,AT3G15040,AT2G32880,AT5G13730,AT4G29670,AT5G11890,AT4G29260,AT1G19360,AT1G32310,AT5G11480,AT1G09230,AT1G78240,AT5G25070,AT5G24970,AT1G21500,AT5G67200,AT5G08100,AT5G08120,AT1G11860,AT3G26630,AT1G26590,AT5G06970,AT5G66410,AT5G06870,AT5G65630,AT1G55670,AT5G05740,AT1G04530,AT1G62960,AT4G24460,AT5G04810,AT5G63860,AT3G23580,AT3G24590,AT1G08640,AT3G21650,AT1G18740,AT4G19185,AT1G23205,AT1G14280,AT1G28410,AT3G16170,AT3G15480,AT4G18520,AT3G63310,AT1G49580,AT3G62580,AT1G50120,AT5G59540,AT1G01940,AT3G62280,AT1G18330,AT1G03900,AT5G59140,AT3G62370,AT5G58870,AT3G02750,AT3G02830,AT4G12010,AT5G58380,AT1G05030,AT3G60750,AT5G57930,AT5G57290,AT3G09750,AT3G59060,AT3G10850,AT2G46570,AT4G05320,AT2G15570,AT2G15520,AT3G31980,AT3G01440,AT4G03600,AT3G10690,AT2G48020,AT4G02630,AT4G01940,AT3G05000,AT1G21680,AT5G51390,AT1G17160

CAGATCNG	10	5	['12-16']	162	AT4G00955,AT3G53720,AT4G00730,AT3G10200,AT3G53470,AT3G01490,AT1G15100,AT1G14150,AT2G40935,AT1G06470,AT5G49540,AT3G53030,AT3G52150,AT5G49280,AT3G07770,AT2G29560,AT3G50500,AT3G50860,AT3G50240,AT1G11630,AT1G58360,AT1G07010,AT2G35190,AT1G20696,AT4G16330,AT2G47690,AT1G42970,AT3G47560,AT1G30200,AT3G46970,AT1G56290,AT1G63080,AT1G03130,AT3G46340,AT5G42470,AT3G45630,AT1G25230,AT1G78600,AT1G78450,AT1G72030,AT5G41760,AT2G25930,AT2G31360,AT4G40030,AT2G15290,AT2G03890,AT2G39480,AT2G13360,AT1G33270,AT4G38710,AT4G38570,AT2G41080,AT2G31670,AT2G41090,AT4G38460,AT5G19420,AT5G35970,AT2G42590,AT5G20470,AT5G35460,AT5G24620,AT1G67623,AT1G09340,AT4G36640,AT1G74370,AT4G36580,AT2G21540,AT5G19240,AT3G13430,AT4G34220,AT5G22270,AT1G51940,AT1G69390,AT1G69210,AT1G69830,AT5G16290,AT2G41760,AT2G41720,AT2G22360,AT2G45590,AT3G14900,AT3G15040,AT2G32880,AT5G13730,AT4G29670,AT5G11890,AT4G29260,AT1G19360,AT1G32310,AT5G11480,AT1G09230,AT1G78240,AT5G25070,AT5G24970,AT1G21500,AT5G67200,AT5G08100,AT5G08120,AT1G11860,AT3G26630,AT1G26590,AT5G06970,AT5G66410,AT5G06870,AT5G65630,AT1G55670,AT5G05740,AT1G04530,AT1G62960,AT4G24460,AT5G04810,AT5G63860,AT3G23580,AT3G24590,AT1G08640,AT3G21650,AT1G18740,AT4G19185,AT1G23205,AT1G14280,AT1G28410,AT3G16170,AT3G15480,AT4G18520,AT3G63310,AT1G49580,AT3G62580,AT1G50120,AT5G59540,AT1G01940,AT3G62280,AT1G18330,AT1G03900,AT5G59140,AT3G62370,AT5G58870,AT3G02750,AT3G02830,AT4G12010,AT5G58380,AT1G05030,AT3G60750,AT5G57930,AT5G57290,AT3G09750,AT3G59060,AT3G10850,AT2G46570,AT4G05320,AT2G15570,AT2G15520,AT3G31980,AT3G01440,AT4G03600,AT3G10690,AT2G48020,AT4G02630,AT4G01940,AT3G05000,AT1G21680,AT5G51390,AT1G17160
CANGTGTC	10	1	['0-4']	288	AT3G54050,AT3G05480,AT2G37450,AT4G00820,AT3G53470,AT3G03520,AT2G07738,AT3G01750,AT1G14150,AT3G01510,AT5G49540,AT1G32060,AT2G47600,AT1G16730,AT3G01060,AT1G75900,AT3G01090,AT2G02160,AT2G06850,AT2G28900,AT3G51240,AT2G28740,AT4G25570,AT1G06410,AT5G47620,AT1G44446,AT1G02340,AT1G12990,AT5G47640,AT1G10740,AT4G14960,AT1G11755,AT5G47020,AT5G46630,AT3G48690,AT1G19140,AT2G41330,AT1G15820,AT3G48530,AT1G15740,AT4G16515,AT1G65800,AT2G35260,AT2G46170,AT1G36830,AT5G44010,AT1G30130,AT1G50940,AT3G47160,AT5G43830,AT2G18170,AT5G59570,AT1G61065,AT4G15110,AT2G16070,AT3G46290,AT3G45260,AT1G78600,AT1G69530,AT3G45600,AT1G64330,AT1G28330,AT5G41600,AT1G73390,AT2G47010,AT3G11200,AT3G11050,AT2G31450,AT1G30210,AT1G75240,AT1G32550,AT1G45230,AT2G45740,AT1G31410,AT1G42190,AT1G71450,AT4G39990,AT4G39400,AT1G31580,AT5G39080,AT5G11060,AT1G76450,AT5G38480,AT4G38940,AT4G39120,AT4G38710,AT3G28780,AT5G37260,AT2G41040,AT2G38120,AT5G19290,AT2G23610,AT5G20360,AT2G37630,AT4G37480,AT1G71880,AT1G52890,AT4G35470,AT4G36600,AT4G37220,AT5G23000,AT3G18610,AT4G34490,AT2G21300,AT4G34950,AT3G56910,AT3G51840,AT4G33950,AT1G80530,AT4G34220,AT3G28700,AT1G69295,AT2G33740,AT2G33700,AT2G33810,AT3G30810,AT2G36060,AT2G42680,AT1G10920,AT4G32140,AT5G15230,AT2G30570,AT4G31820,AT3G13110,AT2G47400,AT5G15450,AT2G21130,AT5G14620,AT2G43840,AT5G15160,AT2G42160,AT2G45300,AT2G47350,AT5G14240,AT5G14310,AT2G10850,AT1G79040,AT1G78995,AT5G13630,AT1G55840,AT5G28020,AT4G30900,AT4G30500,AT5G11890,AT1G22710,AT5G11740,AT1G69040,AT1G32310,AT1G22770,AT1G67510,AT2G42220,AT4G27940,AT3G13860,AT5G26020,AT5G10510,AT3G12780,AT4G27410,AT3G18440,AT5G24950,AT5G24970,AT1G43670,AT4G27130,AT3G29200,AT2G25200,AT3G23070,AT3G23080,AT1G11860,AT1G10210,AT4G26860,AT1G10370,AT5G08000,AT4G26670,AT4G26080,AT3G16910,AT5G06930,AT5G06970,AT1G01240,AT3G17000,AT1G17455,AT1G10090,AT4G25660,AT3G17040,AT4G24540,AT1G04690,AT1G62960,AT5G65380,AT1G04820,AT1G65640,AT1G19650,AT3G19400,AT3G19500,AT1G09680,AT1G32870,AT5G04590,AT4G23750,AT1G12800,AT4G22930,AT1G62300,AT1G32840,AT1G26800,AT1G26780,AT5G03510,AT5G63410,AT5G02810,AT4G21180,AT5G03140,AT1G03600,AT5G02880,AT5G62670,AT3G14000,AT5G62090,AT4G19185,AT5G62220,AT5G01810,AT5G61820,AT5G61580,AT3G63170,AT4G18810,AT4G18530,AT3G63310,AT1G01080,AT3G15430,AT3G17250,AT3G62410,AT5G59950,AT3G62700,AT3G62100,AT3G62260,AT3G02468,AT3G02590,AT1G18460,AT3G61630,AT4G13250,AT1G76180,AT4G11600,AT2G43010,AT5G57760,AT1G50440,AT2G20260,AT5G57660,AT2G18240,AT3G59940,AT1G80730,AT3G59770,AT2G18230,AT2G18300,AT4G08980,AT3G59060,AT2G16600,AT3G59052,AT2G06510,AT3G59220,AT3G04670,AT4G05320,AT3G58120,AT2G15970,AT1G74840,AT4G05180,AT4G05100,AT4G05300,AT3G03160,AT5G54970,AT3G57770,AT3G57170,AT3G10050,AT3G56940,AT2G25510,AT1G27600,AT3G56370,AT5G53290,AT3G10410,AT1G73060,AT3G56090,AT2G06630,AT2G01220,AT2G48020,AT3G55770,AT1G50320,AT5G52420,AT4G02370,AT3G03320,AT4G01690,AT4G01940,AT3G07550,AT5G52010,AT5G51460,AT2G25620,AT2G25710

CANGTGTC	10	3	['0-4']	288	AT3G54050,AT3G05480,AT2G37450,AT4G00820,AT3G53470,AT3G03520,AT2G07738,AT3G01750,AT1G14150,AT3G01510,AT5G49540,AT1G32060,AT2G47600,AT1G16730,AT3G01060,AT1G75900,AT3G01090,AT2G02160,AT2G06850,AT2G28900,AT3G51240,AT2G28740,AT4G25570,AT1G06410,AT5G47620,AT1G44446,AT1G02340,AT1G12990,AT5G47640,AT1G10740,AT4G14960,AT1G11755,AT5G47020,AT5G46630,AT3G48690,AT1G19140,AT2G41330,AT1G15820,AT3G48530,AT1G15740,AT4G16515,AT1G65800,AT2G35260,AT2G46170,AT1G36830,AT5G44010,AT1G30130,AT1G50940,AT3G47160,AT5G43830,AT2G18170,AT5G59570,AT1G61065,AT4G15110,AT2G16070,AT3G46290,AT3G45260,AT1G78600,AT1G69530,AT3G45600,AT1G64330,AT1G28330,AT5G41600,AT1G73390,AT2G47010,AT3G11200,AT3G11050,AT2G31450,AT1G30210,AT1G75240,AT1G32550,AT1G45230,AT2G45740,AT1G31410,AT1G42190,AT1G71450,AT4G39990,AT4G39400,AT1G31580,AT5G39080,AT5G11060,AT1G76450,AT5G38480,AT4G38940,AT4G39120,AT4G38710,AT3G28780,AT5G37260,AT2G41040,AT2G38120,AT5G19290,AT2G23610,AT5G20360,AT2G37630,AT4G37480,AT1G71880,AT1G52890,AT4G35470,AT4G36600,AT4G37220,AT5G23000,AT3G18610,AT4G34490,AT2G21300,AT4G34950,AT3G56910,AT3G51840,AT4G33950,AT1G80530,AT4G34220,AT3G28700,AT1G69295,AT2G33740,AT2G33700,AT2G33810,AT3G30810,AT2G36060,AT2G42680,AT1G10920,AT4G32140,AT5G15230,AT2G30570,AT4G31820,AT3G13110,AT2G47400,AT5G15450,AT2G21130,AT5G14620,AT2G43840,AT5G15160,AT2G42160,AT2G45300,AT2G47350,AT5G14240,AT5G14310,AT2G10850,AT1G79040,AT1G78995,AT5G13630,AT1G55840,AT5G28020,AT4G30900,AT4G30500,AT5G11890,AT1G22710,AT5G11740,AT1G69040,AT1G32310,AT1G22770,AT1G67510,AT2G42220,AT4G27940,AT3G13860,AT5G26020,AT5G10510,AT3G12780,AT4G27410,AT3G18440,AT5G24950,AT5G24970,AT1G43670,AT4G27130,AT3G29200,AT2G25200,AT3G23070,AT3G23080,AT1G11860,AT1G10210,AT4G26860,AT1G10370,AT5G08000,AT4G26670,AT4G26080,AT3G16910,AT5G06930,AT5G06970,AT1G01240,AT3G17000,AT1G17455,AT1G10090,AT4G25660,AT3G17040,AT4G24540,AT1G04690,AT1G62960,AT5G65380,AT1G04820,AT1G65640,AT1G19650,AT3G19400,AT3G19500,AT1G09680,AT1G32870,AT5G04590,AT4G23750,AT1G12800,AT4G22930,AT1G62300,AT1G32840,AT1G26800,AT1G26780,AT5G03510,AT5G63410,AT5G02810,AT4G21180,AT5G03140,AT1G03600,AT5G02880,AT5G62670,AT3G14000,AT5G62090,AT4G19185,AT5G62220,AT5G01810,AT5G61820,AT5G61580,AT3G63170,AT4G18810,AT4G18530,AT3G63310,AT1G01080,AT3G15430,AT3G17250,AT3G62410,AT5G59950,AT3G62700,AT3G62100,AT3G62260,AT3G02468,AT3G02590,AT1G18460,AT3G61630,AT4G13250,AT1G76180,AT4G11600,AT2G43010,AT5G57760,AT1G50440,AT2G20260,AT5G57660,AT2G18240,AT3G59940,AT1G80730,AT3G59770,AT2G18230,AT2G18300,AT4G08980,AT3G59060,AT2G16600,AT3G59052,AT2G06510,AT3G59220,AT3G04670,AT4G05320,AT3G58120,AT2G15970,AT1G74840,AT4G05180,AT4G05100,AT4G05300,AT3G03160,AT5G54970,AT3G57770,AT3G57170,AT3G10050,AT3G56940,AT2G25510,AT1G27600,AT3G56370,AT5G53290,AT3G10410,AT1G73060,AT3G56090,AT2G06630,AT2G01220,AT2G48020,AT3G55770,AT1G50320,AT5G52420,AT4G02370,AT3G03320,AT4G01690,AT4G01940,AT3G07550,AT5G52010,AT5G51460,AT2G25620,AT2G25710

CANGTGTC	10	4	['0-4']	288	AT3G54050,AT3G05480,AT2G37450,AT4G00820,AT3G53470,AT3G03520,AT2G07738,AT3G01750,AT1G14150,AT3G01510,AT5G49540,AT1G32060,AT2G47600,AT1G16730,AT3G01060,AT1G75900,AT3G01090,AT2G02160,AT2G06850,AT2G28900,AT3G51240,AT2G28740,AT4G25570,AT1G06410,AT5G47620,AT1G44446,AT1G02340,AT1G12990,AT5G47640,AT1G10740,AT4G14960,AT1G11755,AT5G47020,AT5G46630,AT3G48690,AT1G19140,AT2G41330,AT1G15820,AT3G48530,AT1G15740,AT4G16515,AT1G65800,AT2G35260,AT2G46170,AT1G36830,AT5G44010,AT1G30130,AT1G50940,AT3G47160,AT5G43830,AT2G18170,AT5G59570,AT1G61065,AT4G15110,AT2G16070,AT3G46290,AT3G45260,AT1G78600,AT1G69530,AT3G45600,AT1G64330,AT1G28330,AT5G41600,AT1G73390,AT2G47010,AT3G11200,AT3G11050,AT2G31450,AT1G30210,AT1G75240,AT1G32550,AT1G45230,AT2G45740,AT1G31410,AT1G42190,AT1G71450,AT4G39990,AT4G39400,AT1G31580,AT5G39080,AT5G11060,AT1G76450,AT5G38480,AT4G38940,AT4G39120,AT4G38710,AT3G28780,AT5G37260,AT2G41040,AT2G38120,AT5G19290,AT2G23610,AT5G20360,AT2G37630,AT4G37480,AT1G71880,AT1G52890,AT4G35470,AT4G36600,AT4G37220,AT5G23000,AT3G18610,AT4G34490,AT2G21300,AT4G34950,AT3G56910,AT3G51840,AT4G33950,AT1G80530,AT4G34220,AT3G28700,AT1G69295,AT2G33740,AT2G33700,AT2G33810,AT3G30810,AT2G36060,AT2G42680,AT1G10920,AT4G32140,AT5G15230,AT2G30570,AT4G31820,AT3G13110,AT2G47400,AT5G15450,AT2G21130,AT5G14620,AT2G43840,AT5G15160,AT2G42160,AT2G45300,AT2G47350,AT5G14240,AT5G14310,AT2G10850,AT1G79040,AT1G78995,AT5G13630,AT1G55840,AT5G28020,AT4G30900,AT4G30500,AT5G11890,AT1G22710,AT5G11740,AT1G69040,AT1G32310,AT1G22770,AT1G67510,AT2G42220,AT4G27940,AT3G13860,AT5G26020,AT5G10510,AT3G12780,AT4G27410,AT3G18440,AT5G24950,AT5G24970,AT1G43670,AT4G27130,AT3G29200,AT2G25200,AT3G23070,AT3G23080,AT1G11860,AT1G10210,AT4G26860,AT1G10370,AT5G08000,AT4G26670,AT4G26080,AT3G16910,AT5G06930,AT5G06970,AT1G01240,AT3G17000,AT1G17455,AT1G10090,AT4G25660,AT3G17040,AT4G24540,AT1G04690,AT1G62960,AT5G65380,AT1G04820,AT1G65640,AT1G19650,AT3G19400,AT3G19500,AT1G09680,AT1G32870,AT5G04590,AT4G23750,AT1G12800,AT4G22930,AT1G62300,AT1G32840,AT1G26800,AT1G26780,AT5G03510,AT5G63410,AT5G02810,AT4G21180,AT5G03140,AT1G03600,AT5G02880,AT5G62670,AT3G14000,AT5G62090,AT4G19185,AT5G62220,AT5G01810,AT5G61820,AT5G61580,AT3G63170,AT4G18810,AT4G18530,AT3G63310,AT1G01080,AT3G15430,AT3G17250,AT3G62410,AT5G59950,AT3G62700,AT3G62100,AT3G62260,AT3G02468,AT3G02590,AT1G18460,AT3G61630,AT4G13250,AT1G76180,AT4G11600,AT2G43010,AT5G57760,AT1G50440,AT2G20260,AT5G57660,AT2G18240,AT3G59940,AT1G80730,AT3G59770,AT2G18230,AT2G18300,AT4G08980,AT3G59060,AT2G16600,AT3G59052,AT2G06510,AT3G59220,AT3G04670,AT4G05320,AT3G58120,AT2G15970,AT1G74840,AT4G05180,AT4G05100,AT4G05300,AT3G03160,AT5G54970,AT3G57770,AT3G57170,AT3G10050,AT3G56940,AT2G25510,AT1G27600,AT3G56370,AT5G53290,AT3G10410,AT1G73060,AT3G56090,AT2G06630,AT2G01220,AT2G48020,AT3G55770,AT1G50320,AT5G52420,AT4G02370,AT3G03320,AT4G01690,AT4G01940,AT3G07550,AT5G52010,AT5G51460,AT2G25620,AT2G25710

CANGTGTC	10	5	['0-4']	288	AT3G54050,AT3G05480,AT2G37450,AT4G00820,AT3G53470,AT3G03520,AT2G07738,AT3G01750,AT1G14150,AT3G01510,AT5G49540,AT1G32060,AT2G47600,AT1G16730,AT3G01060,AT1G75900,AT3G01090,AT2G02160,AT2G06850,AT2G28900,AT3G51240,AT2G28740,AT4G25570,AT1G06410,AT5G47620,AT1G44446,AT1G02340,AT1G12990,AT5G47640,AT1G10740,AT4G14960,AT1G11755,AT5G47020,AT5G46630,AT3G48690,AT1G19140,AT2G41330,AT1G15820,AT3G48530,AT1G15740,AT4G16515,AT1G65800,AT2G35260,AT2G46170,AT1G36830,AT5G44010,AT1G30130,AT1G50940,AT3G47160,AT5G43830,AT2G18170,AT5G59570,AT1G61065,AT4G15110,AT2G16070,AT3G46290,AT3G45260,AT1G78600,AT1G69530,AT3G45600,AT1G64330,AT1G28330,AT5G41600,AT1G73390,AT2G47010,AT3G11200,AT3G11050,AT2G31450,AT1G30210,AT1G75240,AT1G32550,AT1G45230,AT2G45740,AT1G31410,AT1G42190,AT1G71450,AT4G39990,AT4G39400,AT1G31580,AT5G39080,AT5G11060,AT1G76450,AT5G38480,AT4G38940,AT4G39120,AT4G38710,AT3G28780,AT5G37260,AT2G41040,AT2G38120,AT5G19290,AT2G23610,AT5G20360,AT2G37630,AT4G37480,AT1G71880,AT1G52890,AT4G35470,AT4G36600,AT4G37220,AT5G23000,AT3G18610,AT4G34490,AT2G21300,AT4G34950,AT3G56910,AT3G51840,AT4G33950,AT1G80530,AT4G34220,AT3G28700,AT1G69295,AT2G33740,AT2G33700,AT2G33810,AT3G30810,AT2G36060,AT2G42680,AT1G10920,AT4G32140,AT5G15230,AT2G30570,AT4G31820,AT3G13110,AT2G47400,AT5G15450,AT2G21130,AT5G14620,AT2G43840,AT5G15160,AT2G42160,AT2G45300,AT2G47350,AT5G14240,AT5G14310,AT2G10850,AT1G79040,AT1G78995,AT5G13630,AT1G55840,AT5G28020,AT4G30900,AT4G30500,AT5G11890,AT1G22710,AT5G11740,AT1G69040,AT1G32310,AT1G22770,AT1G67510,AT2G42220,AT4G27940,AT3G13860,AT5G26020,AT5G10510,AT3G12780,AT4G27410,AT3G18440,AT5G24950,AT5G24970,AT1G43670,AT4G27130,AT3G29200,AT2G25200,AT3G23070,AT3G23080,AT1G11860,AT1G10210,AT4G26860,AT1G10370,AT5G08000,AT4G26670,AT4G26080,AT3G16910,AT5G06930,AT5G06970,AT1G01240,AT3G17000,AT1G17455,AT1G10090,AT4G25660,AT3G17040,AT4G24540,AT1G04690,AT1G62960,AT5G65380,AT1G04820,AT1G65640,AT1G19650,AT3G19400,AT3G19500,AT1G09680,AT1G32870,AT5G04590,AT4G23750,AT1G12800,AT4G22930,AT1G62300,AT1G32840,AT1G26800,AT1G26780,AT5G03510,AT5G63410,AT5G02810,AT4G21180,AT5G03140,AT1G03600,AT5G02880,AT5G62670,AT3G14000,AT5G62090,AT4G19185,AT5G62220,AT5G01810,AT5G61820,AT5G61580,AT3G63170,AT4G18810,AT4G18530,AT3G63310,AT1G01080,AT3G15430,AT3G17250,AT3G62410,AT5G59950,AT3G62700,AT3G62100,AT3G62260,AT3G02468,AT3G02590,AT1G18460,AT3G61630,AT4G13250,AT1G76180,AT4G11600,AT2G43010,AT5G57760,AT1G50440,AT2G20260,AT5G57660,AT2G18240,AT3G59940,AT1G80730,AT3G59770,AT2G18230,AT2G18300,AT4G08980,AT3G59060,AT2G16600,AT3G59052,AT2G06510,AT3G59220,AT3G04670,AT4G05320,AT3G58120,AT2G15970,AT1G74840,AT4G05180,AT4G05100,AT4G05300,AT3G03160,AT5G54970,AT3G57770,AT3G57170,AT3G10050,AT3G56940,AT2G25510,AT1G27600,AT3G56370,AT5G53290,AT3G10410,AT1G73060,AT3G56090,AT2G06630,AT2G01220,AT2G48020,AT3G55770,AT1G50320,AT5G52420,AT4G02370,AT3G03320,AT4G01690,AT4G01940,AT3G07550,AT5G52010,AT5G51460,AT2G25620,AT2G25710
CATCCAACG	10	1	['4-8', '12-16']	16	AT1G05030,AT1G77590,AT5G39940,AT1G17880,AT3G13470,AT1G54500,AT5G23410,AT3G03380,AT3G03320,AT1G21000,AT3G60080,AT3G60200,AT5G40660,AT2G34620,AT1G15740,AT5G12250
CATCCAACG	10	2	['4-8', '12-16']	16	AT1G05030,AT1G77590,AT5G39940,AT1G17880,AT3G13470,AT1G54500,AT5G23410,AT3G03380,AT3G03320,AT1G21000,AT3G60080,AT3G60200,AT5G40660,AT2G34620,AT1G15740,AT5G12250
CATCCAACG	10	3	['4-8', '12-16']	16	AT1G05030,AT1G77590,AT5G39940,AT1G17880,AT3G13470,AT1G54500,AT5G23410,AT3G03380,AT3G03320,AT1G21000,AT3G60080,AT3G60200,AT5G40660,AT2G34620,AT1G15740,AT5G12250
CATCCAACG	10	4	['4-8', '12-16']	16	AT1G05030,AT1G77590,AT5G39940,AT1G17880,AT3G13470,AT1G54500,AT5G23410,AT3G03380,AT3G03320,AT1G21000,AT3G60080,AT3G60200,AT5G40660,AT2G34620,AT1G15740,AT5G12250
CATCCAACG	10	5	['4-8', '12-16']	16	AT1G05030,AT1G77590,AT5G39940,AT1G17880,AT3G13470,AT1G54500,AT5G23410,AT3G03380,AT3G03320,AT1G21000,AT3G60080,AT3G60200,AT5G40660,AT2G34620,AT1G15740,AT5G12250

CATGCATG	10	1	['0-4', '16-20', '4-8']	104	AT5G51030,AT5G51040,AT5G04130,AT5G50335,AT2G24270,AT3G10260,AT2G07738,AT1G15310,AT5G49480,AT1G28580,AT1G22200,AT1G13180,AT4G17070,AT1G13130,AT3G49190,AT2G43310,AT5G45830,AT3G48940,AT1G13730,AT3G47960,AT3G48110,AT1G18180,AT2G29670,AT1G54540,AT1G23960,AT1G65190,AT2G19790,AT3G45060,AT2G03310,AT1G52190,AT1G72140,AT1G38065,AT1G31500,AT4G39120,AT2G34490,AT1G68100,AT5G20630,AT2G21660,AT3G29370,AT5G23000,AT3G26380,AT4G36580,AT1G80420,AT3G51840,AT2G39930,AT4G34250,AT3G28180,AT5G16290,AT2G22500,AT4G31390,AT5G14920,AT5G15510,AT2G42160,AT1G53280,AT1G62340,AT5G12250,AT5G11930,AT5G11420,AT4G28420,AT1G49010,AT5G26230,AT4G27320,AT5G07860,AT1G06010,AT3G12980,AT3G12920,AT5G66920,AT1G26590,AT1G09430,AT5G66180,AT1G01250,AT3G19930,AT5G64630,AT3G21250,AT5G63940,AT3G23660,AT1G62290,AT4G21210,AT5G01890,AT5G01090,AT4G18270,AT3G62720,AT5G59540,AT5G59080,AT5G58910,AT2G36690,AT1G15520,AT3G08030,AT4G09570,AT1G64640,AT5G56260,AT5G55960,AT3G04650,AT4G05100,AT4G04930,AT4G04910,AT2G05520,AT5G54650,AT2G27490,AT5G52780,AT3G07390,AT1G47530,AT2G01620,AT5G51300
CATGCATG	10	2	['0-4', '4-8', '16-20']	104	AT5G51030,AT5G51040,AT5G04130,AT5G50335,AT2G24270,AT3G10260,AT2G07738,AT1G15310,AT5G49480,AT1G28580,AT1G22200,AT1G13180,AT4G17070,AT1G13130,AT3G49190,AT2G43310,AT5G45830,AT3G48940,AT1G13730,AT3G47960,AT3G48110,AT1G18180,AT2G29670,AT1G54540,AT1G23960,AT1G65190,AT2G19790,AT3G45060,AT2G03310,AT1G52190,AT1G72140,AT1G38065,AT1G31500,AT4G39120,AT2G34490,AT1G68100,AT5G20630,AT2G21660,AT3G29370,AT5G23000,AT3G26380,AT4G36580,AT1G80420,AT3G51840,AT2G39930,AT4G34250,AT3G28180,AT5G16290,AT2G22500,AT4G31390,AT5G14920,AT5G15510,AT2G42160,AT1G53280,AT1G62340,AT5G12250,AT5G11930,AT5G11420,AT4G28420,AT1G49010,AT5G26230,AT4G27320,AT5G07860,AT1G06010,AT3G12980,AT3G12920,AT5G66920,AT1G26590,AT1G09430,AT5G66180,AT1G01250,AT3G19930,AT5G64630,AT3G21250,AT5G63940,AT3G23660,AT1G62290,AT4G21210,AT5G01890,AT5G01090,AT4G18270,AT3G62720,AT5G59540,AT5G59080,AT5G58910,AT2G36690,AT1G15520,AT3G08030,AT4G09570,AT1G64640,AT5G56260,AT5G55960,AT3G04650,AT4G05100,AT4G04930,AT4G04910,AT2G05520,AT5G54650,AT2G27490,AT5G52780,AT3G07390,AT1G47530,AT2G01620,AT5G51300
CATGCATG	10	3	['0-4', '4-8', '16-20']	104	AT5G51030,AT5G51040,AT5G04130,AT5G50335,AT2G24270,AT3G10260,AT2G07738,AT1G15310,AT5G49480,AT1G28580,AT1G22200,AT1G13180,AT4G17070,AT1G13130,AT3G49190,AT2G43310,AT5G45830,AT3G48940,AT1G13730,AT3G47960,AT3G48110,AT1G18180,AT2G29670,AT1G54540,AT1G23960,AT1G65190,AT2G19790,AT3G45060,AT2G03310,AT1G52190,AT1G72140,AT1G38065,AT1G31500,AT4G39120,AT2G34490,AT1G68100,AT5G20630,AT2G21660,AT3G29370,AT5G23000,AT3G26380,AT4G36580,AT1G80420,AT3G51840,AT2G39930,AT4G34250,AT3G28180,AT5G16290,AT2G22500,AT4G31390,AT5G14920,AT5G15510,AT2G42160,AT1G53280,AT1G62340,AT5G12250,AT5G11930,AT5G11420,AT4G28420,AT1G49010,AT5G26230,AT4G27320,AT5G07860,AT1G06010,AT3G12980,AT3G12920,AT5G66920,AT1G26590,AT1G09430,AT5G66180,AT1G01250,AT3G19930,AT5G64630,AT3G21250,AT5G63940,AT3G23660,AT1G62290,AT4G21210,AT5G01890,AT5G01090,AT4G18270,AT3G62720,AT5G59540,AT5G59080,AT5G58910,AT2G36690,AT1G15520,AT3G08030,AT4G09570,AT1G64640,AT5G56260,AT5G55960,AT3G04650,AT4G05100,AT4G04930,AT4G04910,AT2G05520,AT5G54650,AT2G27490,AT5G52780,AT3G07390,AT1G47530,AT2G01620,AT5G51300
CATGCATG	10	4	['0-4', '16-20', '4-8']	104	AT5G51030,AT5G51040,AT5G04130,AT5G50335,AT2G24270,AT3G10260,AT2G07738,AT1G15310,AT5G49480,AT1G28580,AT1G22200,AT1G13180,AT4G17070,AT1G13130,AT3G49190,AT2G43310,AT5G45830,AT3G48940,AT1G13730,AT3G47960,AT3G48110,AT1G18180,AT2G29670,AT1G54540,AT1G23960,AT1G65190,AT2G19790,AT3G45060,AT2G03310,AT1G52190,AT1G72140,AT1G38065,AT1G31500,AT4G39120,AT2G34490,AT1G68100,AT5G20630,AT2G21660,AT3G29370,AT5G23000,AT3G26380,AT4G36580,AT1G80420,AT3G51840,AT2G39930,AT4G34250,AT3G28180,AT5G16290,AT2G22500,AT4G31390,AT5G14920,AT5G15510,AT2G42160,AT1G53280,AT1G62340,AT5G12250,AT5G11930,AT5G11420,AT4G28420,AT1G49010,AT5G26230,AT4G27320,AT5G07860,AT1G06010,AT3G12980,AT3G12920,AT5G66920,AT1G26590,AT1G09430,AT5G66180,AT1G01250,AT3G19930,AT5G64630,AT3G21250,AT5G63940,AT3G23660,AT1G62290,AT4G21210,AT5G01890,AT5G01090,AT4G18270,AT3G62720,AT5G59540,AT5G59080,AT5G58910,AT2G36690,AT1G15520,AT3G08030,AT4G09570,AT1G64640,AT5G56260,AT5G55960,AT3G04650,AT4G05100,AT4G04930,AT4G04910,AT2G05520,AT5G54650,AT2G27490,AT5G52780,AT3G07390,AT1G47530,AT2G01620,AT5G51300

CATGCATG	10	5	['4-8', '16-20', '0-4']	104	AT5G51030,AT5G51040,AT5G04130,AT5G50335,AT2G24270,AT3G10260,AT2G07738,AT1G15310,AT5G49480,AT1G28580,AT1G22200,AT1G13180,AT4G17070,AT1G13130,AT3G49190,AT2G43310,AT5G45830,AT3G48940,AT1G13730,AT3G47960,AT3G48110,AT1G18180,AT2G29670,AT1G54540,AT1G23960,AT1G65190,AT2G19790,AT3G45060,AT2G03310,AT1G52190,AT1G72140,AT1G38065,AT1G31500,AT4G39120,AT2G34490,AT1G68100,AT5G20630,AT2G21660,AT3G29370,AT5G23000,AT3G26380,AT4G36580,AT1G80420,AT3G51840,AT2G39930,AT4G34250,AT3G28180,AT5G16290,AT2G22500,AT4G31390,AT5G14920,AT5G15510,AT2G42160,AT1G53280,AT1G62340,AT5G12250,AT5G11930,AT5G11420,AT4G28420,AT1G49010,AT5G26230,AT4G27320,AT5G07860,AT1G06010,AT3G12980,AT3G12920,AT5G66920,AT1G26590,AT1G09430,AT5G66180,AT1G01250,AT3G19930,AT5G64630,AT3G21250,AT5G63940,AT3G23660,AT1G62290,AT4G21210,AT5G01890,AT5G01090,AT4G18270,AT3G62720,AT5G59540,AT5G59080,AT5G58910,AT2G36690,AT1G15520,AT3G08030,AT4G09570,AT1G64640,AT5G56260,AT5G55960,AT3G04650,AT4G05100,AT4G04930,AT4G04910,AT2G05520,AT5G54650,AT2G27490,AT5G52780,AT3G07390,AT1G47530,AT2G01620,AT5G51300
CATTAATTAG	10	1	['0-4', '4-8', '16-20', '20-24']	22	AT1G50030,AT4G38830,AT2G45140,AT5G49240,AT1G70290,AT5G35460,AT2G42930,AT1G22050,AT5G47390,AT3G11800,AT2G43310,AT4G16870,AT4G16480,AT5G63370,AT4G20930,AT3G12560,AT1G73030,AT1G79770,AT1G03160,AT5G62200,AT5G13630,AT5G01090
CATTAATTAG	10	2	['0-4', '4-8', '16-20', '20-24']	22	AT1G50030,AT4G38830,AT2G45140,AT5G49240,AT1G70290,AT5G35460,AT2G42930,AT1G22050,AT5G47390,AT3G11800,AT2G43310,AT4G16870,AT4G16480,AT5G63370,AT4G20930,AT3G12560,AT1G73030,AT1G79770,AT1G03160,AT5G62200,AT5G13630,AT5G01090
CATTAATTAG	10	3	['0-4', '4-8', '16-20', '20-24']	22	AT1G50030,AT4G38830,AT2G45140,AT5G49240,AT1G70290,AT5G35460,AT2G42930,AT1G22050,AT5G47390,AT3G11800,AT2G43310,AT4G16870,AT4G16480,AT5G63370,AT4G20930,AT3G12560,AT1G73030,AT1G79770,AT1G03160,AT5G62200,AT5G13630,AT5G01090
CATTAATTAG	10	4	['0-4', '16-20', '20-24', '4-8']	22	AT1G50030,AT4G38830,AT2G45140,AT5G49240,AT1G70290,AT5G35460,AT2G42930,AT1G22050,AT5G47390,AT3G11800,AT2G43310,AT4G16870,AT4G16480,AT5G63370,AT4G20930,AT3G12560,AT1G73030,AT1G79770,AT1G03160,AT5G62200,AT5G13630,AT5G01090
CATTAATTAG	10	5	['0-4', '4-8', '16-20', '20-24']	22	AT1G50030,AT4G38830,AT2G45140,AT5G49240,AT1G70290,AT5G35460,AT2G42930,AT1G22050,AT5G47390,AT3G11800,AT2G43310,AT4G16870,AT4G16480,AT5G63370,AT4G20930,AT3G12560,AT1G73030,AT1G79770,AT1G03160,AT5G62200,AT5G13630,AT5G01090
CCAAAAAAGG	10	1	['4-8', '8-12']	23	AT4G00355,AT2G45380,AT3G61130,AT2G22300,AT5G21100,AT1G43670,AT1G66980,AT3G12980,AT5G47870,AT3G27980,AT1G19110,AT3G18930,AT3G03150,AT5G64030,AT4G33300,AT1G70782,AT2G16070,AT1G03430,AT5G02880,AT4G02480,AT1G11130,AT1G65420,AT3G04790
CCAAAAAAGG	10	2	['4-8', '8-12']	23	AT4G00355,AT2G45380,AT3G61130,AT2G22300,AT5G21100,AT1G43670,AT1G66980,AT3G12980,AT5G47870,AT3G27980,AT1G19110,AT3G18930,AT3G03150,AT5G64030,AT4G33300,AT1G70782,AT2G16070,AT1G03430,AT5G02880,AT4G02480,AT1G11130,AT1G65420,AT3G04790
CCAAAAAAGG	10	3	['4-8', '8-12']	23	AT4G00355,AT2G45380,AT3G61130,AT2G22300,AT5G21100,AT1G43670,AT1G66980,AT3G12980,AT5G47870,AT3G27980,AT1G19110,AT3G18930,AT3G03150,AT5G64030,AT4G33300,AT1G70782,AT2G16070,AT1G03430,AT5G02880,AT4G02480,AT1G11130,AT1G65420,AT3G04790
CCAAAAAAGG	10	4	['4-8', '8-12']	23	AT4G00355,AT2G45380,AT3G61130,AT2G22300,AT5G21100,AT1G43670,AT1G66980,AT3G12980,AT5G47870,AT3G27980,AT1G19110,AT3G18930,AT3G03150,AT5G64030,AT4G33300,AT1G70782,AT2G16070,AT1G03430,AT5G02880,AT4G02480,AT1G11130,AT1G65420,AT3G04790
CCAAAAAAGG	10	5	['4-8', '8-12']	23	AT4G00355,AT2G45380,AT3G61130,AT2G22300,AT5G21100,AT1G43670,AT1G66980,AT3G12980,AT5G47870,AT3G27980,AT1G19110,AT3G18930,AT3G03150,AT5G64030,AT4G33300,AT1G70782,AT2G16070,AT1G03430,AT5G02880,AT4G02480,AT1G11130,AT1G65420,AT3G04790
CCAAAAATGG	10	1	['0-4', '4-8', '8-12', '12-16', '16-20']	10	AT1G28600,AT3G48110,AT2G21130,AT4G26080,AT5G26030,AT2G14080,AT1G30070,AT2G37940,AT2G25710,AT3G01090

CCAAAAATGG	10	2	['0-4', '4-8', '8-12', '12-16', '16-20']	10	AT1G28600,AT3G48110,AT2G21130,AT4G26080,AT5G26030,AT2G14080,AT1G30070,AT2G37940,AT2G25710,AT3G01090
CCAAAAATGG	10	3	['0-4', '4-8', '8-12', '12-16', '16-20']	10	AT1G28600,AT3G48110,AT2G21130,AT4G26080,AT5G26030,AT2G14080,AT1G30070,AT2G37940,AT2G25710,AT3G01090
CCAAAAATGG	10	4	['0-4', '4-8', '8-12', '12-16', '16-20']	10	AT1G28600,AT3G48110,AT2G21130,AT4G26080,AT5G26030,AT2G14080,AT1G30070,AT2G37940,AT2G25710,AT3G01090
CCAAAAATGG	10	5	['0-4', '4-8', '8-12', '12-16', '16-20']	10	AT1G28600,AT3G48110,AT2G21130,AT4G26080,AT5G26030,AT2G14080,AT1G30070,AT2G37940,AT2G25710,AT3G01090
CCAAAATTGG	10	1	['0-4', '8-12']	12	AT1G03550,AT2G19810,AT1G63830,AT5G04810,AT5G25900,AT3G16910,AT5G63860,AT3G49250,AT1G70330,AT5G65990,AT5G20630,AT2G16070
CCAAAATTGG	10	2	['0-4', '8-12']	12	AT1G03550,AT2G19810,AT1G63830,AT5G04810,AT5G25900,AT3G16910,AT5G63860,AT3G49250,AT1G70330,AT5G65990,AT5G20630,AT2G16070
CCAAAATTGG	10	3	['0-4', '8-12']	12	AT1G03550,AT2G19810,AT1G63830,AT5G04810,AT5G25900,AT3G16910,AT5G63860,AT3G49250,AT1G70330,AT5G65990,AT5G20630,AT2G16070
CCAAAATTGG	10	4	['0-4', '8-12']	12	AT1G03550,AT2G19810,AT1G63830,AT5G04810,AT5G25900,AT3G16910,AT5G63860,AT3G49250,AT1G70330,AT5G65990,AT5G20630,AT2G16070
CCAAAATTGG	10	5	['0-4', '8-12']	12	AT1G03550,AT2G19810,AT1G63830,AT5G04810,AT5G25900,AT3G16910,AT5G63860,AT3G49250,AT1G70330,AT5G65990,AT5G20630,AT2G16070
CCAAATAAGG	10	1	['0-4', '4-8', '8-12', '12-16', '20-24']	12	AT3G23760,AT4G18280,AT5G04610,AT3G19640,AT1G03970,AT5G62160,AT1G10760,AT2G32950,AT1G34000,AT5G03240,AT2G21330,AT2G36240
CCAAATAAGG	10	2	['0-4', '4-8', '8-12', '20-24', '12-16']	12	AT3G23760,AT4G18280,AT5G04610,AT3G19640,AT1G03970,AT5G62160,AT1G10760,AT2G32950,AT1G34000,AT5G03240,AT2G21330,AT2G36240
CCAAATAAGG	10	3	['0-4', '4-8', '8-12', '12-16', '20-24']	12	AT3G23760,AT4G18280,AT5G04610,AT3G19640,AT1G03970,AT5G62160,AT1G10760,AT2G32950,AT1G34000,AT5G03240,AT2G21330,AT2G36240
CCAAATAAGG	10	4	['0-4', '4-8', '8-12', '12-16', '20-24']	12	AT3G23760,AT4G18280,AT5G04610,AT3G19640,AT1G03970,AT5G62160,AT1G10760,AT2G32950,AT1G34000,AT5G03240,AT2G21330,AT2G36240
CCAAATAAGG	10	5	['0-4', '4-8', '8-12', '12-16', '20-24']	12	AT3G23760,AT4G18280,AT5G04610,AT3G19640,AT1G03970,AT5G62160,AT1G10760,AT2G32950,AT1G34000,AT5G03240,AT2G21330,AT2G36240
CCAAATTTGG	10	1	['8-12', '12-16', '16-20', '20-24']	14	AT2G36720,AT1G42190,AT1G73990,AT5G11500,AT4G39150,AT5G25070,AT2G32390,AT1G73980,AT4G25970,AT2G33860,AT3G43670,AT1G11630,AT5G36960,AT3G26380
CCAAATTTGG	10	2	['8-12', '12-16', '16-20', '20-24']	14	AT2G36720,AT1G42190,AT1G73990,AT5G11500,AT4G39150,AT5G25070,AT2G32390,AT1G73980,AT4G25970,AT2G33860,AT3G43670,AT1G11630,AT5G36960,AT3G26380
CCAAATTTGG	10	3	['8-12', '12-16', '20-24', '16-20']	14	AT2G36720,AT1G42190,AT1G73990,AT5G11500,AT4G39150,AT5G25070,AT2G32390,AT1G73980,AT4G25970,AT2G33860,AT3G43670,AT1G11630,AT5G36960,AT3G26380

CCAAATTTGG	10	4	['8-12', '12-16', '16-20', '20-24']	14	AT2G36720,AT1G42190,AT1G73990,AT5G11500,AT4G39150,AT5G25070,AT2G32390,AT1G73980,AT4G25970,AT2G33860,AT3G43670,AT1G11630,AT5G36960,AT3G26380
CCAAATTTGG	10	5	['8-12', '12-16', '16-20', '20-24']	14	AT2G36720,AT1G42190,AT1G73990,AT5G11500,AT4G39150,AT5G25070,AT2G32390,AT1G73980,AT4G25970,AT2G33860,AT3G43670,AT1G11630,AT5G36960,AT3G26380
CCAATAATGG	10	1	['0-4', '8-12', '16-20']	11	AT5G39570,AT5G05300,AT4G24700,AT1G70820,AT2G40110,AT5G02620,AT3G53870,AT5G60210,AT3G15690,AT3G18440,AT3G61580
CCAATAATGG	10	2	['0-4', '8-12', '16-20']	11	AT5G39570,AT5G05300,AT4G24700,AT1G70820,AT2G40110,AT5G02620,AT3G53870,AT5G60210,AT3G15690,AT3G18440,AT3G61580
CCAATAATGG	10	3	['0-4', '8-12', '16-20']	11	AT5G39570,AT5G05300,AT4G24700,AT1G70820,AT2G40110,AT5G02620,AT3G53870,AT5G60210,AT3G15690,AT3G18440,AT3G61580
CCAATAATGG	10	4	['0-4', '8-12', '16-20']	11	AT5G39570,AT5G05300,AT4G24700,AT1G70820,AT2G40110,AT5G02620,AT3G53870,AT5G60210,AT3G15690,AT3G18440,AT3G61580
CCAATAATGG	10	5	['0-4', '8-12', '16-20']	11	AT5G39570,AT5G05300,AT4G24700,AT1G70820,AT2G40110,AT5G02620,AT3G53870,AT5G60210,AT3G15690,AT3G18440,AT3G61580
CCAATTTAGG	10	1	['0-4', '4-8', '16-20', '20-24', '8-12']	10	AT3G42100,AT2G29850,AT1G58360,AT1G16000,AT5G57760,AT2G27130,AT1G47530,AT3G57470,AT3G52450,AT1G72800
CCAATTTAGG	10	2	['0-4', '4-8', '16-20', '20-24']	10	AT3G42100,AT2G29850,AT1G58360,AT1G16000,AT5G57760,AT2G27130,AT1G47530,AT3G57470,AT3G52450,AT1G72800
CCAATTTAGG	10	3	['0-4', '4-8', '8-12', '20-24', '16-20']	10	AT3G42100,AT2G29850,AT1G58360,AT1G16000,AT5G57760,AT2G27130,AT1G47530,AT3G57470,AT3G52450,AT1G72800
CCAATTTAGG	10	4	['0-4', '4-8', '16-20', '20-24']	10	AT3G42100,AT2G29850,AT1G58360,AT1G16000,AT5G57760,AT2G27130,AT1G47530,AT3G57470,AT3G52450,AT1G72800
CCAATTTAGG	10	5	['0-4', '4-8', '16-20', '20-24']	10	AT3G42100,AT2G29850,AT1G58360,AT1G16000,AT5G57760,AT2G27130,AT1G47530,AT3G57470,AT3G52450,AT1G72800
CCAATTTTGG	10	1	['0-4', '4-8', '8-12', '16-20']	14	AT1G27630,AT4G08320,AT3G26710,AT1G33270,AT5G44010,AT5G34883,AT2G36320,AT4G13170,AT3G19970,AT5G65990,AT2G02760,AT5G65840,AT3G02830,AT3G63170
CCAATTTTGG	10	2	['0-4', '4-8', '8-12', '16-20']	14	AT1G27630,AT4G08320,AT3G26710,AT1G33270,AT5G44010,AT5G34883,AT2G36320,AT4G13170,AT3G19970,AT5G65990,AT2G02760,AT5G65840,AT3G02830,AT3G63170
CCAATTTTGG	10	3	['0-4', '4-8', '8-12', '16-20']	14	AT1G27630,AT4G08320,AT3G26710,AT1G33270,AT5G44010,AT5G34883,AT2G36320,AT4G13170,AT3G19970,AT5G65990,AT2G02760,AT5G65840,AT3G02830,AT3G63170
CCAATTTTGG	10	4	['0-4', '4-8', '8-12', '16-20']	14	AT1G27630,AT4G08320,AT3G26710,AT1G33270,AT5G44010,AT5G34883,AT2G36320,AT4G13170,AT3G19970,AT5G65990,AT2G02760,AT5G65840,AT3G02830,AT3G63170
CCAATTTTGG	10	5	['4-8', '8-12', '16-20', '0-4']	14	AT1G27630,AT4G08320,AT3G26710,AT1G33270,AT5G44010,AT5G34883,AT2G36320,AT4G13170,AT3G19970,AT5G65990,AT2G02760,AT5G65840,AT3G02830,AT3G63170

CCACGTCA	10	1	['20-24']	95	AT5G49280,AT3G51620,AT2G39080,AT3G50910,AT3G50860,AT2G25620,AT5G45840,AT3G48940,AT4G14430,AT1G13370,AT1G56220,AT5G43830,AT1G03090,AT2G19810,AT5G41060,AT3G11050,AT1G76460,AT2G28720,AT1G73670,AT5G36790,AT2G42590,AT2G24930,AT2G38230,AT2G04690,AT3G22190,AT5G23420,AT4G35090,AT2G39900,AT3G14090,AT2G44360,AT5G16880,AT4G33430,AT4G33300,AT1G69800,AT4G32410,AT1G68190,AT2G32765,AT4G31340,AT5G14920,AT5G14800,AT4G30500,AT1G79050,AT5G13730,AT4G29610,AT1G65420,AT5G27920,AT4G28750,AT1G16150,AT1G32470,AT5G25430,AT1G03250,AT5G24930,AT5G67300,AT3G23080,AT5G66880,AT5G06870,AT5G66340,AT1G10090,AT5G65730,AT5G65110,AT4G24190,AT5G64260,AT1G62180,AT5G02810,AT5G02820,AT3G21560,AT1G79790,AT5G02880,AT5G61380,AT5G60100,AT5G58870,AT5G58620,AT1G76100,AT1G76140,AT4G11175,AT3G08590,AT2G20260,AT2G18240,AT3G11900,AT3G05800,AT3G10720,AT4G08180,AT5G55380,AT4G05180,AT4G05300,AT1G48370,AT5G54650,AT4G03510,AT5G54080,AT3G01990,AT3G08890,AT4G02940,AT3G01850,AT5G51460,AT1G21670
CCACGTCA	10	2	['20-24']	95	AT5G49280,AT3G51620,AT2G39080,AT3G50910,AT3G50860,AT2G25620,AT5G45840,AT3G48940,AT4G14430,AT1G13370,AT1G56220,AT5G43830,AT1G03090,AT2G19810,AT5G41060,AT3G11050,AT1G76460,AT2G28720,AT1G73670,AT5G36790,AT2G42590,AT2G24930,AT2G38230,AT2G04690,AT3G22190,AT5G23420,AT4G35090,AT2G39900,AT3G14090,AT2G44360,AT5G16880,AT4G33430,AT4G33300,AT1G69800,AT4G32410,AT1G68190,AT2G32765,AT4G31340,AT5G14920,AT5G14800,AT4G30500,AT1G79050,AT5G13730,AT4G29610,AT1G65420,AT5G27920,AT4G28750,AT1G16150,AT1G32470,AT5G25430,AT1G03250,AT5G24930,AT5G67300,AT3G23080,AT5G66880,AT5G06870,AT5G66340,AT1G10090,AT5G65730,AT5G65110,AT4G24190,AT5G64260,AT1G62180,AT5G02810,AT5G02820,AT3G21560,AT1G79790,AT5G02880,AT5G61380,AT5G60100,AT5G58870,AT5G58620,AT1G76100,AT1G76140,AT4G11175,AT3G08590,AT2G20260,AT2G18240,AT3G11900,AT3G05800,AT3G10720,AT4G08180,AT5G55380,AT4G05180,AT4G05300,AT1G48370,AT5G54650,AT4G03510,AT5G54080,AT3G01990,AT3G08890,AT4G02940,AT3G01850,AT5G51460,AT1G21670
CCACGTCA	10	3	['20-24']	95	AT5G49280,AT3G51620,AT2G39080,AT3G50910,AT3G50860,AT2G25620,AT5G45840,AT3G48940,AT4G14430,AT1G13370,AT1G56220,AT5G43830,AT1G03090,AT2G19810,AT5G41060,AT3G11050,AT1G76460,AT2G28720,AT1G73670,AT5G36790,AT2G42590,AT2G24930,AT2G38230,AT2G04690,AT3G22190,AT5G23420,AT4G35090,AT2G39900,AT3G14090,AT2G44360,AT5G16880,AT4G33430,AT4G33300,AT1G69800,AT4G32410,AT1G68190,AT2G32765,AT4G31340,AT5G14920,AT5G14800,AT4G30500,AT1G79050,AT5G13730,AT4G29610,AT1G65420,AT5G27920,AT4G28750,AT1G16150,AT1G32470,AT5G25430,AT1G03250,AT5G24930,AT5G67300,AT3G23080,AT5G66880,AT5G06870,AT5G66340,AT1G10090,AT5G65730,AT5G65110,AT4G24190,AT5G64260,AT1G62180,AT5G02810,AT5G02820,AT3G21560,AT1G79790,AT5G02880,AT5G61380,AT5G60100,AT5G58870,AT5G58620,AT1G76100,AT1G76140,AT4G11175,AT3G08590,AT2G20260,AT2G18240,AT3G11900,AT3G05800,AT3G10720,AT4G08180,AT5G55380,AT4G05180,AT4G05300,AT1G48370,AT5G54650,AT4G03510,AT5G54080,AT3G01990,AT3G08890,AT4G02940,AT3G01850,AT5G51460,AT1G21670
CCACGTCA	10	4	['20-24']	95	AT5G49280,AT3G51620,AT2G39080,AT3G50910,AT3G50860,AT2G25620,AT5G45840,AT3G48940,AT4G14430,AT1G13370,AT1G56220,AT5G43830,AT1G03090,AT2G19810,AT5G41060,AT3G11050,AT1G76460,AT2G28720,AT1G73670,AT5G36790,AT2G42590,AT2G24930,AT2G38230,AT2G04690,AT3G22190,AT5G23420,AT4G35090,AT2G39900,AT3G14090,AT2G44360,AT5G16880,AT4G33430,AT4G33300,AT1G69800,AT4G32410,AT1G68190,AT2G32765,AT4G31340,AT5G14920,AT5G14800,AT4G30500,AT1G79050,AT5G13730,AT4G29610,AT1G65420,AT5G27920,AT4G28750,AT1G16150,AT1G32470,AT5G25430,AT1G03250,AT5G24930,AT5G67300,AT3G23080,AT5G66880,AT5G06870,AT5G66340,AT1G10090,AT5G65730,AT5G65110,AT4G24190,AT5G64260,AT1G62180,AT5G02810,AT5G02820,AT3G21560,AT1G79790,AT5G02880,AT5G61380,AT5G60100,AT5G58870,AT5G58620,AT1G76100,AT1G76140,AT4G11175,AT3G08590,AT2G20260,AT2G18240,AT3G11900,AT3G05800,AT3G10720,AT4G08180,AT5G55380,AT4G05180,AT4G05300,AT1G48370,AT5G54650,AT4G03510,AT5G54080,AT3G01990,AT3G08890,AT4G02940,AT3G01850,AT5G51460,AT1G21670

CCACGTCA	10	5	['20-24']	95	AT5G49280,AT3G51620,AT2G39080,AT3G50910,AT3G50860,AT2G25620,AT5G45840,AT3G48940,AT4G14430,AT1G13370,AT1G56220,AT5G43830,AT1G03090,AT2G19810,AT5G41060,AT3G11050,AT1G76460,AT2G28720,AT1G73670,AT5G36790,AT2G42590,AT2G24930,AT2G38230,AT2G04690,AT3G22190,AT5G23420,AT4G35090,AT2G39900,AT3G14090,AT2G44360,AT5G16880,AT4G33430,AT4G33300,AT1G69800,AT4G32410,AT1G68190,AT2G32765,AT4G31340,AT5G14920,AT5G14800,AT4G30500,AT1G79050,AT5G13730,AT4G29610,AT1G65420,AT5G27920,AT4G28750,AT1G16150,AT1G32470,AT5G25430,AT1G03250,AT5G24930,AT5G67300,AT3G23080,AT5G66880,AT5G06870,AT5G66340,AT1G10090,AT5G65730,AT5G65110,AT4G24190,AT5G64260,AT1G62180,AT5G02810,AT5G02820,AT3G21560,AT1G79790,AT5G02880,AT5G61380,AT5G60100,AT5G58870,AT5G58620,AT1G76100,AT1G76140,AT4G11175,AT3G08590,AT2G20260,AT2G18240,AT3G11900,AT3G05800,AT3G10720,AT4G08180,AT5G55380,AT4G05180,AT4G05300,AT1G48370,AT5G54650,AT4G03510,AT5G54080,AT3G01990,AT3G08890,AT4G02940,AT3G01850,AT5G51460,AT1G21670
CCACGTCATC	10	1	['0-4', '4-8', '8-12', '12-16', '16-20', '20-24']	20	AT3G23080,AT3G01990,AT1G79790,AT3G01850,AT5G54080,AT3G08590,AT5G60100,AT5G14800,AT2G28720,AT2G20260,AT3G50860,AT3G22190,AT4G33430,AT1G62180,AT4G29610,AT1G21670,AT5G61380,AT1G68190,AT5G58620,AT5G65110
CCACGTCATC	10	2	['0-4', '4-8', '8-12', '16-20', '20-24', '12-16']	20	AT3G23080,AT3G01990,AT1G79790,AT3G01850,AT5G54080,AT3G08590,AT5G60100,AT5G14800,AT2G28720,AT2G20260,AT3G50860,AT3G22190,AT4G33430,AT1G62180,AT4G29610,AT1G21670,AT5G61380,AT1G68190,AT5G58620,AT5G65110
CCACGTCATC	10	3	['0-4', '4-8', '8-12', '12-16', '20-24', '16-20']	20	AT3G23080,AT3G01990,AT1G79790,AT3G01850,AT5G54080,AT3G08590,AT5G60100,AT5G14800,AT2G28720,AT2G20260,AT3G50860,AT3G22190,AT4G33430,AT1G62180,AT4G29610,AT1G21670,AT5G61380,AT1G68190,AT5G58620,AT5G65110
CCACGTCATC	10	4	['0-4', '4-8', '8-12', '12-16', '16-20', '20-24']	20	AT3G23080,AT3G01990,AT1G79790,AT3G01850,AT5G54080,AT3G08590,AT5G60100,AT5G14800,AT2G28720,AT2G20260,AT3G50860,AT3G22190,AT4G33430,AT1G62180,AT4G29610,AT1G21670,AT5G61380,AT1G68190,AT5G58620,AT5G65110
CCACGTCATC	10	5	['0-4', '4-8', '8-12', '12-16', '16-20', '20-24']	20	AT3G23080,AT3G01990,AT1G79790,AT3G01850,AT5G54080,AT3G08590,AT5G60100,AT5G14800,AT2G28720,AT2G20260,AT3G50860,AT3G22190,AT4G33430,AT1G62180,AT4G29610,AT1G21670,AT5G61380,AT1G68190,AT5G58620,AT5G65110

CCACTG	10	1	['12-16']	558	ATMG01040,AT3G03520,AT3G01510,AT2G47450,AT3G07640,AT3G07680,AT3G03770,AT1G44510,AT1G44478,AT1G06410,AT4G17230,AT4G17615,AT4G15550,AT1G15820,AT4G14410,AT4G16447,AT1G43930,AT1G20650,AT1G35290,AT1G21300,AT1G20440,AT1G20410,AT4G16710,AT4G16870,AT4G16140,AT4G16190,AT1G52360,AT4G15810,AT1G61065,AT1G71030,AT1G29390,AT1G29490,AT1G28330,AT1G73390,AT1G52230,AT1G76790,AT1G76360,AT1G74160,AT1G45230,AT1G75080,AT5G28290,AT1G26220,AT5G11070,AT5G19760,AT1G68100,AT5G20630,AT5G08410,AT1G73670,AT5G21060,AT5G21170,AT1G73820,AT1G35460,AT5G20320,AT5G20070,AT1G66640,AT1G66330,AT5G20840,AT1G70660,AT4G36790,AT1G70730,AT1G74600,AT4G37260,AT1G74560,AT1G31910,AT3G56910,AT1G80480,AT1G63830,AT1G51940,AT1G73990,AT5G17440,AT1G73980,AT5G16970,AT1G69830,AT1G68160,AT1G10920,AT1G51550,AT2G41760,AT2G47400,AT5G15510,AT2G43550,AT2G45300,AT2G43790,AT5G34830,AT1G08080,AT5G27150,AT5G26770,AT1G14990,AT5G25220,AT1G43715,AT1G06820,AT1G06750,AT1G21460,AT1G29240,AT1G21400,AT5G67320,AT5G67020,AT5G67030,AT5G66770,AT5G66340,AT1G01240,AT1G17455,AT5G66060,AT1G01230,AT5G65630,AT1G01250,AT1G07440,AT5G65430,AT1G75330,AT5G65000,AT1G19660,AT5G64860,AT1G04850,AT1G04910,AT5G64460,AT5G64260,AT1G12860,AT1G12800,AT5G64030,AT1G20190,AT1G05920,AT5G63820,AT5G63380,AT5G62940,AT5G62740,AT1G79730,AT5G62810,AT1G05385,AT5G62350,AT1G79610,AT1G18740,AT1G07890,AT5G61820,AT5G61990,AT1G71695,AT5G61380,AT5G60920,AT1G63630,AT1G01770,AT5G60550,AT1G27700,AT1G47290,AT1G32770,AT5G58730,AT5G58620,AT1G76090,AT5G58260,AT2G27210,AT1G76100,AT1G76280,AT5G57760,AT5G57110,AT1G80730,AT5G56790,AT1G22490,AT5G56260,AT1G35580,AT5G56030,AT5G55380,AT1G77920,AT1G52730,AT1G52720,AT5G55220,AT5G54950,AT5G54970,AT5G54980,AT1G74750,AT1G70985,AT1G71010,AT5G53290,AT1G49260,AT1G11260,AT5G52120,AT5G51970,AT5G51390,AT1G17145,AT1G17050,AT1G15290,AT5G50180,AT5G49990,AT5G49740,AT5G49540,AT5G49230,AT5G49450,AT1G06680,AT1G06570,AT1G62750,AT5G48810,AT1G76030,AT1G16240,AT1G16520,AT5G48230,AT1G43580,AT1G28530,AT1G12990,AT5G47610,AT1G10760,AT1G11755,AT1G14810,AT1G64970,AT5G45840,AT1G64770,AT1G19910,AT5G44730,AT5G44780,AT1G54410,AT5G44070,AT5G43930,AT1G54540,AT1G23980,AT5G42810,AT1G03130,AT1G65210,AT1G54100,AT5G41650,AT5G41760,AT5G41060,AT2G15290,AT2G03890,AT2G11830,AT2G31710,AT3G53870,AT5G37780,AT2G28630,AT2G31670,AT5G36950,AT2G31820,AT2G31790,AT5G35970,AT2G42590,AT2G24820,AT2G21660,AT5G24490,AT2G25330,AT2G25250,AT5G24010,AT5G23870,AT2G04840,AT1G26830,AT5G22750,AT2G21380,AT2G46450,AT5G19140,AT5G22300,AT5G18540,AT5G18640,AT2G04420,AT5G17990,AT5G18170,AT2G36320,AT5G17780,AT5G17660,AT2G42670,AT5G16400,AT5G15160,AT2G22540,AT5G14550,AT2G10850,AT5G14090,AT5G11930,AT1G22750,AT5G11480,AT5G11150,AT5G11160,AT5G11260,AT5G10530,AT1G78890,AT5G10430,AT1G70410,AT1G04140,AT5G09650,AT1G10210,AT5G07960,AT1G27450,AT1G67110,AT1G67120,AT5G06970,AT1G09570,AT5G05950,AT1G55670,AT1G05190,AT5G05740,AT1G04550,AT5G06060,AT5G05520,AT1G65640,AT1G08980,AT5G04940,AT5G05170,AT1G70250,AT1G70210,AT5G03760,AT5G03795,AT5G03190,AT5G02870,AT1G03380,AT1G03430,AT5G02490,AT5G02620,AT1G61210,AT5G02240,AT2G17270,AT1G60550,AT5G01890,AT1G60650,AT1G60490,AT3G63160,AT3G63310,AT1G67280,AT1G67310,AT3G62550,AT3G62860,AT3G61820,AT3G61460,AT1G23490,AT3G61580,AT2G36720,AT2G43010,AT3G60750,AT3G60020,AT2G18240,AT2G18410,AT3G60080,AT3G59350,AT3G59400,AT2G20830,AT3G58680,AT2G46490,AT3G57785,AT3G31980,AT2G05520,AT3G57190,AT3G57250,AT3G56410,AT3G56370,AT3G55680,AT3G55760,AT3G55770,AT2G01290,AT3G55450,AT2G01760,AT2G01680,AT2G25620,AT3G54500,AT3G54240,AT3G54270,AT3G53720,AT2G37240,AT3G53470,AT2G40780,AT2G18640,AT4G38290,AT3G53030,AT2G40700,AT3G52070,AT2G37860,AT2G02070,AT3G50700,AT3G50860,AT3G50240,AT2G41410,AT2G14690,AT2G38790,AT2G38610,AT2G07140,AT2G43310,AT2G47910,AT2G35190,AT2G06980,AT3G47590,AT2G24090,AT3G46780,AT3G46970,AT2G35605,AT2G35410,AT3G46170,AT3G46220,AT2G19810,AT2G19860,AT2G46820,AT3G44720,AT2G47010,AT3G44250,AT3G62250,AT3G43790,AT4G39950,AT4G39400,AT4G39100,AT2G39400,AT4G38710,AT4G38580,AT2G41120,AT4G38520,AT2G41430,AT2G32560,AT2G38130,AT2G30860,AT2G37520,AT2G31010,AT2G44130,AT4G34950,AT4G34350,AT2G30070,AT4G34190,AT4G34030,AT4G34090,AT4G34150,AT2G39900,AT2G40080,AT4G33640,AT2G44550,AT4G33500,AT2G33700,AT2G30490,AT2G30600,AT4G32270,AT2G45590,AT4G30900,AT5G09810,AT4G29890,AT4G28610,AT4G27790,AT4G27780,AT4G28130,AT4G27670,AT4G27585,AT4G27130,AT4G27300,AT4G26700,AT4G26520,AT4G26560,AT4G26530,AT4G26050,AT4G24930,AT4G24700,AT4G24350,AT4G24460,AT4G24470,AT4G23940,AT4G22300,AT4G20840,AT4G21210,AT4G20360,AT4G20170,AT4G19710,AT4G19120,AT4G19340,AT4G19350,AT4G19070,AT4G18240,AT4G13930,AT4G13010,AT4G11710,A

CCACTG	10	3	['12-16']	558	ATMG01040,AT3G03520,AT3G01510,AT2G47450,AT3G07640,AT3G07680,AT3G03770,AT1G44510,AT1G44478,AT1G06410,AT4G17230,AT4G17615,AT4G15550,AT1G15820,AT4G14410,AT4G16447,AT1G43930,AT1G20650,AT1G35290,AT1G21300,AT1G20440,AT1G20410,AT4G16710,AT4G16870,AT4G16140,AT4G16190,AT1G52360,AT4G15810,AT1G61065,AT1G71030,AT1G29390,AT1G29490,AT1G28330,AT1G73390,AT1G52230,AT1G76790,AT1G76360,AT1G74160,AT1G45230,AT1G75080,AT5G28290,AT1G26220,AT5G11070,AT5G19760,AT1G68100,AT5G20630,AT5G08410,AT1G73670,AT5G21060,AT5G21170,AT1G73820,AT1G35460,AT5G20320,AT5G20070,AT1G66640,AT1G66330,AT5G20840,AT1G70660,AT4G36790,AT1G70730,AT1G74600,AT4G37260,AT1G74560,AT1G31910,AT3G56910,AT1G80480,AT1G63830,AT1G51940,AT1G73990,AT5G17440,AT1G73980,AT5G16970,AT1G69830,AT1G68160,AT1G10920,AT1G51550,AT2G41760,AT2G47400,AT5G15510,AT2G43550,AT2G45300,AT2G43790,AT5G34830,AT1G08080,AT5G27150,AT5G26770,AT1G14990,AT5G25220,AT1G43715,AT1G06820,AT1G06750,AT1G21460,AT1G29240,AT1G21400,AT5G67320,AT5G67020,AT5G67030,AT5G66770,AT5G66340,AT1G01240,AT1G17455,AT5G66060,AT1G01230,AT5G65630,AT1G01250,AT1G07440,AT5G65430,AT1G75330,AT5G65000,AT1G19660,AT5G64860,AT1G04850,AT1G04910,AT5G64460,AT5G64260,AT1G12860,AT1G12800,AT5G64030,AT1G20190,AT1G05920,AT5G63820,AT5G63380,AT5G62940,AT5G62740,AT1G79730,AT5G62810,AT1G05385,AT5G62350,AT1G79610,AT1G18740,AT1G07890,AT5G61820,AT5G61990,AT1G71695,AT5G61380,AT5G60920,AT1G63630,AT1G01770,AT5G60550,AT1G27700,AT1G47290,AT1G32770,AT5G58730,AT5G58620,AT1G76090,AT5G58260,AT2G27210,AT1G76100,AT1G76280,AT5G57760,AT5G57110,AT1G80730,AT5G56790,AT1G22490,AT5G56260,AT1G35580,AT5G56030,AT5G55380,AT1G77920,AT1G52730,AT1G52720,AT5G55220,AT5G54950,AT5G54970,AT5G54980,AT1G74750,AT1G70985,AT1G71010,AT5G53290,AT1G49260,AT1G11260,AT5G52120,AT5G51970,AT5G51390,AT1G17145,AT1G17050,AT1G15290,AT5G50180,AT5G49990,AT5G49740,AT5G49540,AT5G49230,AT5G49450,AT1G06680,AT1G06570,AT1G62750,AT5G48810,AT1G76030,AT1G16240,AT1G16520,AT5G48230,AT1G43580,AT1G28530,AT1G12990,AT5G47610,AT1G10760,AT1G11755,AT1G14810,AT1G64970,AT5G45840,AT1G64770,AT1G19910,AT5G44730,AT5G44780,AT1G54410,AT5G44070,AT5G43930,AT1G54540,AT1G23980,AT5G42810,AT1G03130,AT1G65210,AT1G54100,AT5G41650,AT5G41760,AT5G41060,AT2G15290,AT2G03890,AT2G11830,AT2G31710,AT3G53870,AT5G37780,AT2G28630,AT2G31670,AT5G36950,AT2G31820,AT2G31790,AT5G35970,AT2G42590,AT2G24820,AT2G21660,AT5G24490,AT2G25330,AT2G25250,AT5G24010,AT5G23870,AT2G04840,AT1G26830,AT5G22750,AT2G21380,AT2G46450,AT5G19140,AT5G22300,AT5G18540,AT5G18640,AT2G04420,AT5G17990,AT5G18170,AT2G36320,AT5G17780,AT5G17660,AT2G42670,AT5G16400,AT5G15160,AT2G22540,AT5G14550,AT2G10850,AT5G14090,AT5G11930,AT1G22750,AT5G11480,AT5G11150,AT5G11160,AT5G11260,AT5G10530,AT1G78890,AT5G10430,AT1G70410,AT1G04140,AT5G09650,AT1G10210,AT5G07960,AT1G27450,AT1G67110,AT1G67120,AT5G06970,AT1G09570,AT5G05950,AT1G55670,AT1G05190,AT5G05740,AT1G04550,AT5G06060,AT5G05520,AT1G65640,AT1G08980,AT5G04940,AT5G05170,AT1G70250,AT1G70210,AT5G03760,AT5G03795,AT5G03190,AT5G02870,AT1G03380,AT1G03430,AT5G02490,AT5G02620,AT1G61210,AT5G02240,AT2G17270,AT1G60550,AT5G01890,AT1G60650,AT1G60490,AT3G63160,AT3G63310,AT1G67280,AT1G67310,AT3G62550,AT3G62860,AT3G61820,AT3G61460,AT1G23490,AT3G61580,AT2G36720,AT2G43010,AT3G60750,AT3G60020,AT2G18240,AT2G18410,AT3G60080,AT3G59350,AT3G59400,AT2G20830,AT3G58680,AT2G46490,AT3G57785,AT3G31980,AT2G05520,AT3G57190,AT3G57250,AT3G56410,AT3G56370,AT3G55680,AT3G55760,AT3G55770,AT2G01290,AT3G55450,AT2G01760,AT2G01680,AT2G25620,AT3G54500,AT3G54240,AT3G54270,AT3G53720,AT2G37240,AT3G53470,AT2G40780,AT2G18640,AT4G38290,AT3G53030,AT2G40700,AT3G52070,AT2G37860,AT2G02070,AT3G50700,AT3G50860,AT3G50240,AT2G41410,AT2G14690,AT2G38790,AT2G38610,AT2G07140,AT2G43310,AT2G47910,AT2G35190,AT2G06980,AT3G47590,AT2G24090,AT3G46780,AT3G46970,AT2G35605,AT2G35410,AT3G46170,AT3G46220,AT2G19810,AT2G19860,AT2G46820,AT3G44720,AT2G47010,AT3G44250,AT3G62250,AT3G43790,AT4G39950,AT4G39400,AT4G39100,AT2G39400,AT4G38710,AT4G38580,AT2G41120,AT4G38520,AT2G41430,AT2G32560,AT2G38130,AT2G30860,AT2G37520,AT2G31010,AT2G44130,AT4G34950,AT4G34350,AT2G30070,AT4G34190,AT4G34030,AT4G34090,AT4G34150,AT2G39900,AT2G40080,AT4G33640,AT2G44550,AT4G33500,AT2G33700,AT2G30490,AT2G30600,AT4G32270,AT2G45590,AT4G30900,AT5G09810,AT4G29890,AT4G28610,AT4G27790,AT4G27780,AT4G28130,AT4G27670,AT4G27585,AT4G27130,AT4G27300,AT4G26700,AT4G26520,AT4G26560,AT4G26530,AT4G26050,AT4G24930,AT4G24700,AT4G24350,AT4G24460,AT4G24470,AT4G23940,AT4G22300,AT4G20840,AT4G21210,AT4G20360,AT4G20170,AT4G19710,AT4G19120,AT4G19340,AT4G19350,AT4G19070,AT4G18240,AT4G13930,AT4G13010,AT4G11710,A

CCACTG	10	5	['12-16']	558	ATMG01040,AT3G03520,AT3G01510,AT2G47450,AT3G07640,AT3G07680,AT3G03770,AT1G44510,AT1G44478,AT1G06410,AT4G17230,AT4G17615,AT4G15550,AT1G15820,AT4G14410,AT4G16447,AT1G43930,AT1G20650,AT1G35290,AT1G21300,AT1G20440,AT1G20410,AT4G16710,AT4G16870,AT4G16140,AT4G16190,AT1G52360,AT4G15810,AT1G61065,AT1G71030,AT1G29390,AT1G29490,AT1G28330,AT1G73390,AT1G52230,AT1G76790,AT1G76360,AT1G74160,AT1G45230,AT1G75080,AT5G28290,AT1G26220,AT5G11070,AT5G19760,AT1G68100,AT5G20630,AT5G08410,AT1G73670,AT5G21060,AT5G21170,AT1G73820,AT1G35460,AT5G20320,AT5G20070,AT1G66640,AT1G66330,AT5G20840,AT1G70660,AT4G36790,AT1G70730,AT1G74600,AT4G37260,AT1G74560,AT1G31910,AT3G56910,AT1G80480,AT1G63830,AT1G51940,AT1G73990,AT5G17440,AT1G73980,AT5G16970,AT1G69830,AT1G68160,AT1G10920,AT1G51550,AT2G41760,AT2G47400,AT5G15510,AT2G43550,AT2G45300,AT2G43790,AT5G34830,AT1G08080,AT5G27150,AT5G26770,AT1G14990,AT5G25220,AT1G43715,AT1G06820,AT1G06750,AT1G21460,AT1G29240,AT1G21400,AT5G67320,AT5G67020,AT5G67030,AT5G66770,AT5G66340,AT1G01240,AT1G17455,AT5G66060,AT1G01230,AT5G65630,AT1G01250,AT1G07440,AT5G65430,AT1G75330,AT5G65000,AT1G19660,AT5G64860,AT1G04850,AT1G04910,AT5G64460,AT5G64260,AT1G12860,AT1G12800,AT5G64030,AT1G20190,AT1G05920,AT5G63820,AT5G63380,AT5G62940,AT5G62740,AT1G79730,AT5G62810,AT1G05385,AT5G62350,AT1G79610,AT1G18740,AT1G07890,AT5G61820,AT5G61990,AT1G71695,AT5G61380,AT5G60920,AT1G63630,AT1G01770,AT5G60550,AT1G27700,AT1G47290,AT1G32770,AT5G58730,AT5G58620,AT1G76090,AT5G58260,AT2G27210,AT1G76100,AT1G76280,AT5G57760,AT5G57110,AT1G80730,AT5G56790,AT1G22490,AT5G56260,AT1G35580,AT5G56030,AT5G55380,AT1G77920,AT1G52730,AT1G52720,AT5G55220,AT5G54950,AT5G54970,AT5G54980,AT1G74750,AT1G70985,AT1G71010,AT5G53290,AT1G49260,AT1G11260,AT5G52120,AT5G51970,AT5G51390,AT1G17145,AT1G17050,AT1G15290,AT5G50180,AT5G49990,AT5G49740,AT5G49540,AT5G49230,AT5G49450,AT1G06680,AT1G06570,AT1G62750,AT5G48810,AT1G76030,AT1G16240,AT1G16520,AT5G48230,AT1G43580,AT1G28530,AT1G12990,AT5G47610,AT1G10760,AT1G11755,AT1G14810,AT1G64970,AT5G45840,AT1G64770,AT1G19910,AT5G44730,AT5G44780,AT1G54410,AT5G44070,AT5G43930,AT1G54540,AT1G23980,AT5G42810,AT1G03130,AT1G65210,AT1G54100,AT5G41650,AT5G41760,AT5G41060,AT2G15290,AT2G03890,AT2G11830,AT2G31710,AT3G53870,AT5G37780,AT2G28630,AT2G31670,AT5G36950,AT2G31820,AT2G31790,AT5G35970,AT2G42590,AT2G24820,AT2G21660,AT5G24490,AT2G25330,AT2G25250,AT5G24010,AT5G23870,AT2G04840,AT1G26830,AT5G22750,AT2G21380,AT2G46450,AT5G19140,AT5G22300,AT5G18540,AT5G18640,AT2G04420,AT5G17990,AT5G18170,AT2G36320,AT5G17780,AT5G17660,AT2G42670,AT5G16400,AT5G15160,AT2G22540,AT5G14550,AT2G10850,AT5G14090,AT5G11930,AT1G22750,AT5G11480,AT5G11150,AT5G11160,AT5G11260,AT5G10530,AT1G78890,AT5G10430,AT1G70410,AT1G04140,AT5G09650,AT1G10210,AT5G07960,AT1G27450,AT1G67110,AT1G67120,AT5G06970,AT1G09570,AT5G05950,AT1G55670,AT1G05190,AT5G05740,AT1G04550,AT5G06060,AT5G05520,AT1G65640,AT1G08980,AT5G04940,AT5G05170,AT1G70250,AT1G70210,AT5G03760,AT5G03795,AT5G03190,AT5G02870,AT1G03380,AT1G03430,AT5G02490,AT5G02620,AT1G61210,AT5G02240,AT2G17270,AT1G60550,AT5G01890,AT1G60650,AT1G60490,AT3G63160,AT3G63310,AT1G67280,AT1G67310,AT3G62550,AT3G62860,AT3G61820,AT3G61460,AT1G23490,AT3G61580,AT2G36720,AT2G43010,AT3G60750,AT3G60020,AT2G18240,AT2G18410,AT3G60080,AT3G59350,AT3G59400,AT2G20830,AT3G58680,AT2G46490,AT3G57785,AT3G31980,AT2G05520,AT3G57190,AT3G57250,AT3G56410,AT3G56370,AT3G55680,AT3G55760,AT3G55770,AT2G01290,AT3G55450,AT2G01760,AT2G01680,AT2G25620,AT3G54500,AT3G54240,AT3G54270,AT3G53720,AT2G37240,AT3G53470,AT2G40780,AT2G18640,AT4G38290,AT3G53030,AT2G40700,AT3G52070,AT2G37860,AT2G02070,AT3G50700,AT3G50860,AT3G50240,AT2G41410,AT2G14690,AT2G38790,AT2G38610,AT2G07140,AT2G43310,AT2G47910,AT2G35190,AT2G06980,AT3G47590,AT2G24090,AT3G46780,AT3G46970,AT2G35605,AT2G35410,AT3G46170,AT3G46220,AT2G19810,AT2G19860,AT2G46820,AT3G44720,AT2G47010,AT3G44250,AT3G62250,AT3G43790,AT4G39950,AT4G39400,AT4G39100,AT2G39400,AT4G38710,AT4G38580,AT2G41120,AT4G38520,AT2G41430,AT2G32560,AT2G38130,AT2G30860,AT2G37520,AT2G31010,AT2G44130,AT4G34950,AT4G34350,AT2G30070,AT4G34190,AT4G34030,AT4G34090,AT4G34150,AT2G39900,AT2G40080,AT4G33640,AT2G44550,AT4G33500,AT2G33700,AT2G30490,AT2G30600,AT4G32270,AT2G45590,AT4G30900,AT5G09810,AT4G29890,AT4G28610,AT4G27790,AT4G27780,AT4G28130,AT4G27670,AT4G27585,AT4G27130,AT4G27300,AT4G26700,AT4G26520,AT4G26560,AT4G26530,AT4G26050,AT4G24930,AT4G24700,AT4G24350,AT4G24460,AT4G24470,AT4G23940,AT4G22300,AT4G20840,AT4G21210,AT4G20360,AT4G20170,AT4G19710,AT4G19120,AT4G19340,AT4G19350,AT4G19070,AT4G18240,AT4G13930,AT4G13010,AT4G11710,A
CCAGGTGG	10	1	['0-4', '4-8', '8-12', '16-20', '20-24']	38	AT3G62550,AT4G00660,AT5G26820,AT1G06430,AT1G47330,AT5G08330,AT3G18440,AT5G49240,AT1G70290,AT2G02070,AT5G12470,AT3G60620,AT1G53440,AT5G47640,AT4G35830,AT4G35060,AT4G37250,AT3G19810,AT4G08320,AT5G19250,AT3G19500,AT1G59580,AT3G56720,AT5G18525,AT3G14090,AT1G30120,AT1G69880,AT4G32410,AT5G63410,AT4G20900,AT1G13860,AT1G25230,AT2G43530,AT3G55770,AT3G07310,AT2G17410,AT3G07470,AT5G40500
CCAGGTGG	10	2	['0-4', '4-8', '8-12', '16-20', '20-24']	38	AT3G62550,AT4G00660,AT5G26820,AT1G06430,AT1G47330,AT5G08330,AT3G18440,AT5G49240,AT1G70290,AT2G02070,AT5G12470,AT3G60620,AT1G53440,AT5G47640,AT4G35830,AT4G35060,AT4G37250,AT3G19810,AT4G08320,AT5G19250,AT3G19500,AT1G59580,AT3G56720,AT5G18525,AT3G14090,AT1G30120,AT1G69880,AT4G32410,AT5G63410,AT4G20900,AT1G13860,AT1G25230,AT2G43530,AT3G55770,AT3G07310,AT2G17410,AT3G07470,AT5G40500

CCAGGTGG	10	3	['0-4', '4-8', '8-12', '16-20', '20-24']	38	AT3G62550,AT4G00660,AT5G26820,AT1G06430,AT1G47330,AT5G08330,AT3G18440,AT5G49240,AT1G70290,AT2G02070,AT5G12470,AT3G60620,AT1G53440,AT5G47640,AT4G35830,AT4G35060,AT4G37250,AT3G19810,AT4G08320,AT5G19250,AT3G19500,AT1G59580,AT3G56720,AT5G18525,AT3G14090,AT1G30120,AT1G69880,AT4G32410,AT5G63410,AT4G20900,AT1G13860,AT1G25230,AT2G43530,AT3G55770,AT3G07310,AT2G17410,AT3G07470,AT5G40500
CCAGGTGG	10	4	['0-4', '4-8', '8-12', '16-20', '20-24']	38	AT3G62550,AT4G00660,AT5G26820,AT1G06430,AT1G47330,AT5G08330,AT3G18440,AT5G49240,AT1G70290,AT2G02070,AT5G12470,AT3G60620,AT1G53440,AT5G47640,AT4G35830,AT4G35060,AT4G37250,AT3G19810,AT4G08320,AT5G19250,AT3G19500,AT1G59580,AT3G56720,AT5G18525,AT3G14090,AT1G30120,AT1G69880,AT4G32410,AT5G63410,AT4G20900,AT1G13860,AT1G25230,AT2G43530,AT3G55770,AT3G07310,AT2G17410,AT3G07470,AT5G40500
CCAGGTGG	10	5	['4-8', '8-12', '16-20', '20-24', '0-4']	38	AT3G62550,AT4G00660,AT5G26820,AT1G06430,AT1G47330,AT5G08330,AT3G18440,AT5G49240,AT1G70290,AT2G02070,AT5G12470,AT3G60620,AT1G53440,AT5G47640,AT4G35830,AT4G35060,AT4G37250,AT3G19810,AT4G08320,AT5G19250,AT3G19500,AT1G59580,AT3G56720,AT5G18525,AT3G14090,AT1G30120,AT1G69880,AT4G32410,AT5G63410,AT4G20900,AT1G13860,AT1G25230,AT2G43530,AT3G55770,AT3G07310,AT2G17410,AT3G07470,AT5G40500
CCATACATT	10	1	['0-4', '4-8', '12-16', '20-24']	18	AT4G29260,AT2G02040,AT4G17640,AT5G39740,AT2G39930,AT4G14965,AT1G72810,AT2G39400,AT3G07350,AT2G01170,AT4G38690,AT5G02160,AT3G11620,AT1G17360,AT2G01760,AT5G43810,AT1G65970,AT1G22590
CCATACATT	10	2	['0-4', '4-8', '12-16', '20-24']	18	AT4G29260,AT2G02040,AT4G17640,AT5G39740,AT2G39930,AT4G14965,AT1G72810,AT2G39400,AT3G07350,AT2G01170,AT4G38690,AT5G02160,AT3G11620,AT1G17360,AT2G01760,AT5G43810,AT1G65970,AT1G22590
CCATACATT	10	3	['0-4', '4-8', '12-16', '20-24']	18	AT4G29260,AT2G02040,AT4G17640,AT5G39740,AT2G39930,AT4G14965,AT1G72810,AT2G39400,AT3G07350,AT2G01170,AT4G38690,AT5G02160,AT3G11620,AT1G17360,AT2G01760,AT5G43810,AT1G65970,AT1G22590
CCATACATT	10	4	['0-4', '12-16', '20-24', '4-8']	18	AT4G29260,AT2G02040,AT4G17640,AT5G39740,AT2G39930,AT4G14965,AT1G72810,AT2G39400,AT3G07350,AT2G01170,AT4G38690,AT5G02160,AT3G11620,AT1G17360,AT2G01760,AT5G43810,AT1G65970,AT1G22590
CCATACATT	10	5	['0-4', '4-8', '12-16', '20-24']	18	AT4G29260,AT2G02040,AT4G17640,AT5G39740,AT2G39930,AT4G14965,AT1G72810,AT2G39400,AT3G07350,AT2G01170,AT4G38690,AT5G02160,AT3G11620,AT1G17360,AT2G01760,AT5G43810,AT1G65970,AT1G22590
CCATTAATGG	10	1	['4-8']	10	AT5G51040,AT2G46490,AT1G29670,AT2G06005,AT3G58040,AT5G04790,AT1G54520,AT3G44660,AT3G44630,AT1G09430
CCATTAATGG	10	2	['4-8']	10	AT5G51040,AT2G46490,AT1G29670,AT2G06005,AT3G58040,AT5G04790,AT1G54520,AT3G44660,AT3G44630,AT1G09430
CCATTAATGG	10	3	['4-8']	10	AT5G51040,AT2G46490,AT1G29670,AT2G06005,AT3G58040,AT5G04790,AT1G54520,AT3G44660,AT3G44630,AT1G09430
CCATTAATGG	10	4	['4-8']	10	AT5G51040,AT2G46490,AT1G29670,AT2G06005,AT3G58040,AT5G04790,AT1G54520,AT3G44660,AT3G44630,AT1G09430
CCATTAATGG	10	5	['4-8']	10	AT5G51040,AT2G46490,AT1G29670,AT2G06005,AT3G58040,AT5G04790,AT1G54520,AT3G44660,AT3G44630,AT1G09430
CCATTTTAGG	10	1	['4-8', '8-12', '12-16']	10	AT3G21210,AT4G14870,AT5G25900,AT5G37640,AT1G74910,AT1G55910,AT1G23390,AT1G79050,AT1G60590,AT1G76090
CCATTTTAGG	10	2	['4-8', '8-12', '12-16']	10	AT3G21210,AT4G14870,AT5G25900,AT5G37640,AT1G74910,AT1G55910,AT1G23390,AT1G79050,AT1G60590,AT1G76090
CCATTTTAGG	10	3	['4-8', '8-12', '12-16']	10	AT3G21210,AT4G14870,AT5G25900,AT5G37640,AT1G74910,AT1G55910,AT1G23390,AT1G79050,AT1G60590,AT1G76090
CCATTTTAGG	10	4	['4-8', '8-12', '12-16']	10	AT3G21210,AT4G14870,AT5G25900,AT5G37640,AT1G74910,AT1G55910,AT1G23390,AT1G79050,AT1G60590,AT1G76090
CCATTTTAGG	10	5	['4-8', '8-12', '12-16']	10	AT3G21210,AT4G14870,AT5G25900,AT5G37640,AT1G74910,AT1G55910,AT1G23390,AT1G79050,AT1G60590,AT1G76090
CCATTTTTAG	10	1	['0-4', '4-8', '8-12', '16-20', '20-24']	19	AT2G25200,AT1G71020,AT4G37680,AT2G25870,AT2G26620,AT1G18180,AT3G50440,AT4G00370,AT2G34250,AT1G18350,AT3G08650,AT5G02190,AT1G44100,AT2G41080,AT2G22980,AT5G08330,AT1G60800,AT5G58770,AT3G18110
CCATTTTTAG	10	2	['0-4', '4-8', '8-12', '16-20', '20-24']	19	AT2G25200,AT1G71020,AT4G37680,AT2G25870,AT2G26620,AT1G18180,AT3G50440,AT4G00370,AT2G34250,AT1G18350,AT3G08650,AT5G02190,AT1G44100,AT2G41080,AT2G22980,AT5G08330,AT1G60800,AT5G58770,AT3G18110

CCATTTTTAG	10	3	['0-4', '4-8', '8-12', '16-20', '20-24']	19	AT2G25200,AT1G71020,AT4G37680,AT2G25870,AT2G26620,AT1G18180,AT3G50440,AT4G00370,AT2G34250,AT1G18350,AT3G08650,AT5G02190,AT1G44100,AT2G41080,AT2G22980,AT5G08330,AT1G60800,AT5G58770,AT3G18110
CCATTTTTAG	10	4	['0-4', '4-8', '8-12', '16-20', '20-24']	19	AT2G25200,AT1G71020,AT4G37680,AT2G25870,AT2G26620,AT1G18180,AT3G50440,AT4G00370,AT2G34250,AT1G18350,AT3G08650,AT5G02190,AT1G44100,AT2G41080,AT2G22980,AT5G08330,AT1G60800,AT5G58770,AT3G18110
CCATTTTTAG	10	5	['0-4', '4-8', '8-12', '16-20', '20-24']	19	AT2G25200,AT1G71020,AT4G37680,AT2G25870,AT2G26620,AT1G18180,AT3G50440,AT4G00370,AT2G34250,AT1G18350,AT3G08650,AT5G02190,AT1G44100,AT2G41080,AT2G22980,AT5G08330,AT1G60800,AT5G58770,AT3G18110
CCATTTTTGG	10	1	['4-8', '8-12', '12-16', '16-20', '20-24']	11	AT4G24860,AT1G76180,AT5G67370,AT5G14920,AT1G21190,AT1G16570,AT4G02630,AT1G49300,AT3G02468,AT5G26020,AT3G15480
CCATTTTTGG	10	2	['4-8', '8-12', '12-16', '16-20', '20-24']	11	AT4G24860,AT1G76180,AT5G67370,AT5G14920,AT1G21190,AT1G16570,AT4G02630,AT1G49300,AT3G02468,AT5G26020,AT3G15480
CCATTTTTGG	10	3	['4-8', '8-12', '12-16', '20-24', '16-20']	11	AT4G24860,AT1G76180,AT5G67370,AT5G14920,AT1G21190,AT1G16570,AT4G02630,AT1G49300,AT3G02468,AT5G26020,AT3G15480
CCATTTTTGG	10	4	['4-8', '8-12', '12-16', '16-20', '20-24']	11	AT4G24860,AT1G76180,AT5G67370,AT5G14920,AT1G21190,AT1G16570,AT4G02630,AT1G49300,AT3G02468,AT5G26020,AT3G15480
CCATTTTTGG	10	5	['4-8', '8-12', '12-16', '16-20', '20-24']	11	AT4G24860,AT1G76180,AT5G67370,AT5G14920,AT1G21190,AT1G16570,AT4G02630,AT1G49300,AT3G02468,AT5G26020,AT3G15480
CCTAAAAAGG	10	1	['8-12', '12-16', '16-20']	15	AT1G05030,AT3G56290,AT2G25870,AT1G03430,AT3G57280,AT1G75690,AT4G02195,AT5G66530,AT3G15850,AT3G07770,AT5G01950,AT4G34710,AT4G38460,AT1G76090,AT1G64770
CCTAAAAAGG	10	2	['8-12', '16-20', '12-16']	15	AT1G05030,AT3G56290,AT2G25870,AT1G03430,AT3G57280,AT1G75690,AT4G02195,AT5G66530,AT3G15850,AT3G07770,AT5G01950,AT4G34710,AT4G38460,AT1G76090,AT1G64770
CCTAAAAAGG	10	3	['8-12', '12-16', '16-20']	15	AT1G05030,AT3G56290,AT2G25870,AT1G03430,AT3G57280,AT1G75690,AT4G02195,AT5G66530,AT3G15850,AT3G07770,AT5G01950,AT4G34710,AT4G38460,AT1G76090,AT1G64770
CCTAAAAAGG	10	4	['8-12', '12-16', '16-20']	15	AT1G05030,AT3G56290,AT2G25870,AT1G03430,AT3G57280,AT1G75690,AT4G02195,AT5G66530,AT3G15850,AT3G07770,AT5G01950,AT4G34710,AT4G38460,AT1G76090,AT1G64770
CCTAAAAAGG	10	5	['8-12', '12-16', '16-20']	15	AT1G05030,AT3G56290,AT2G25870,AT1G03430,AT3G57280,AT1G75690,AT4G02195,AT5G66530,AT3G15850,AT3G07770,AT5G01950,AT4G34710,AT4G38460,AT1G76090,AT1G64770
CCTAATTTGG	10	1	['0-4', '4-8', '20-24', '8-12']	10	ATMG00610,AT2G05070,AT4G14965,AT3G50750,AT2G33840,AT5G13630,AT5G40850,AT5G36790,AT5G43430,AT1G08380
CCTAATTTGG	10	2	['0-4', '4-8', '20-24']	10	ATMG00610,AT2G05070,AT4G14965,AT3G50750,AT2G33840,AT5G13630,AT5G40850,AT5G36790,AT5G43430,AT1G08380
CCTAATTTGG	10	3	['0-4', '4-8', '8-12', '20-24']	10	ATMG00610,AT2G05070,AT4G14965,AT3G50750,AT2G33840,AT5G13630,AT5G40850,AT5G36790,AT5G43430,AT1G08380
CCTAATTTGG	10	4	['0-4', '4-8', '20-24']	10	ATMG00610,AT2G05070,AT4G14965,AT3G50750,AT2G33840,AT5G13630,AT5G40850,AT5G36790,AT5G43430,AT1G08380
CCTAATTTGG	10	5	['0-4', '4-8', '20-24']	10	ATMG00610,AT2G05070,AT4G14965,AT3G50750,AT2G33840,AT5G13630,AT5G40850,AT5G36790,AT5G43430,AT1G08380

CCTATTTTGG	10	1	['0-4', '4-8', '12-16', '16-20', '20-24']	12	AT5G42940,AT4G08320,AT3G07880,AT1G17970,AT5G53170,AT3G60750,AT2G19750,AT3G07170,AT5G47620,AT5G17780,AT3G16180,AT4G34490
CCTATTTTGG	10	2	['0-4', '4-8', '16-20', '20-24', '12-16']	12	AT5G42940,AT4G08320,AT3G07880,AT1G17970,AT5G53170,AT3G60750,AT2G19750,AT3G07170,AT5G47620,AT5G17780,AT3G16180,AT4G34490
CCTATTTTGG	10	3	['0-4', '4-8', '12-16', '20-24', '16-20']	12	AT5G42940,AT4G08320,AT3G07880,AT1G17970,AT5G53170,AT3G60750,AT2G19750,AT3G07170,AT5G47620,AT5G17780,AT3G16180,AT4G34490
CCTATTTTGG	10	4	['0-4', '4-8', '12-16', '16-20', '20-24']	12	AT5G42940,AT4G08320,AT3G07880,AT1G17970,AT5G53170,AT3G60750,AT2G19750,AT3G07170,AT5G47620,AT5G17780,AT3G16180,AT4G34490
CCTATTTTGG	10	5	['0-4', '4-8', '12-16', '16-20', '20-24']	12	AT5G42940,AT4G08320,AT3G07880,AT1G17970,AT5G53170,AT3G60750,AT2G19750,AT3G07170,AT5G47620,AT5G17780,AT3G16180,AT4G34490
CCTTTTGTCTC	10	1	['0-4', '4-8', '8-12', '12-16']	11	AT4G20900,AT1G42190,AT3G13080,AT5G07860,AT1G64330,AT4G33980,AT3G28780,AT4G35890,AT4G02420,AT1G74670,AT2G35410
CCTTTTGTCTC	10	2	['0-4', '4-8', '8-12', '12-16']	11	AT4G20900,AT1G42190,AT3G13080,AT5G07860,AT1G64330,AT4G33980,AT3G28780,AT4G35890,AT4G02420,AT1G74670,AT2G35410
CCTTTTGTCTC	10	3	['0-4', '4-8', '8-12', '12-16']	11	AT4G20900,AT1G42190,AT3G13080,AT5G07860,AT1G64330,AT4G33980,AT3G28780,AT4G35890,AT4G02420,AT1G74670,AT2G35410
CCTTTTGTCTC	10	4	['0-4', '4-8', '8-12', '12-16']	11	AT4G20900,AT1G42190,AT3G13080,AT5G07860,AT1G64330,AT4G33980,AT3G28780,AT4G35890,AT4G02420,AT1G74670,AT2G35410
CCTTTTGTCTC	10	5	['0-4', '4-8', '8-12', '12-16']	11	AT4G20900,AT1G42190,AT3G13080,AT5G07860,AT1G64330,AT4G33980,AT3G28780,AT4G35890,AT4G02420,AT1G74670,AT2G35410
CCTTTTTTGG	10	1	['0-4', '16-20']	19	AT1G16520,AT5G06060,AT3G04780,AT3G13080,AT1G59860,AT1G16810,AT3G21630,AT1G78080,AT4G28460,AT1G32920,AT1G18740,AT1G20410,AT3G15850,AT5G23760,AT5G23350,AT1G07210,AT1G06750,AT1G26780,AT2G02070
CCTTTTTTGG	10	2	['0-4', '16-20']	19	AT1G16520,AT5G06060,AT3G04780,AT3G13080,AT1G59860,AT1G16810,AT3G21630,AT1G78080,AT4G28460,AT1G32920,AT1G18740,AT1G20410,AT3G15850,AT5G23760,AT5G23350,AT1G07210,AT1G06750,AT1G26780,AT2G02070
CCTTTTTTGG	10	3	['0-4', '16-20']	19	AT1G16520,AT5G06060,AT3G04780,AT3G13080,AT1G59860,AT1G16810,AT3G21630,AT1G78080,AT4G28460,AT1G32920,AT1G18740,AT1G20410,AT3G15850,AT5G23760,AT5G23350,AT1G07210,AT1G06750,AT1G26780,AT2G02070
CCTTTTTTGG	10	4	['0-4', '16-20']	19	AT1G16520,AT5G06060,AT3G04780,AT3G13080,AT1G59860,AT1G16810,AT3G21630,AT1G78080,AT4G28460,AT1G32920,AT1G18740,AT1G20410,AT3G15850,AT5G23760,AT5G23350,AT1G07210,AT1G06750,AT1G26780,AT2G02070
CCTTTTTTGG	10	5	['0-4', '16-20']	19	AT1G16520,AT5G06060,AT3G04780,AT3G13080,AT1G59860,AT1G16810,AT3G21630,AT1G78080,AT4G28460,AT1G32920,AT1G18740,AT1G20410,AT3G15850,AT5G23760,AT5G23350,AT1G07210,AT1G06750,AT1G26780,AT2G02070
CGCGGATC	10	1	['0-4', '8-12', '16-20', '20-24']	10	AT1G54880,AT3G02630,AT5G11070,AT1G05610,AT1G10470,AT3G52450,AT1G48330,AT1G12080,AT4G32060,AT3G12600
CGCGGATC	10	2	['0-4', '8-12', '16-20', '20-24']	10	AT1G54880,AT3G02630,AT5G11070,AT1G05610,AT1G10470,AT3G52450,AT1G48330,AT1G12080,AT4G32060,AT3G12600
CGCGGATC	10	3	['0-4', '8-12', '20-24', '16-20']	10	AT1G54880,AT3G02630,AT5G11070,AT1G05610,AT1G10470,AT3G52450,AT1G48330,AT1G12080,AT4G32060,AT3G12600
CGCGGATC	10	4	['0-4', '8-12', '16-20', '20-24']	10	AT1G54880,AT3G02630,AT5G11070,AT1G05610,AT1G10470,AT3G52450,AT1G48330,AT1G12080,AT4G32060,AT3G12600

CGCGGATC	10	5	['0-4', '8-12', '16-20', '20-24']	10	AT1G54880,AT3G02630,AT5G11070,AT1G05610,AT1G10470,AT3G52450,AT1G48330,AT1G12080,AT4G32060,AT3G12600
CTAGGGTN	10	1	['16-20']	201	AT4G01120,AT1G17100,AT4G00780,ATCG00160,AT1G31330,AT2G18640,AT5G49230,AT1G14040,AT1G16750,AT2G33240,AT1G66930,AT1G44446,AT5G46910,AT1G29900,AT1G18990,AT1G64770,AT1G15670,AT1G07020,AT4G16690,AT1G79500,AT4G17760,AT4G16520,AT3G47560,AT1G42970,AT1G75780,AT1G50940,AT2G18170,AT5G42765,AT1G71020,AT3G12290,AT2G25870,AT1G78450,AT2G19860,AT1G54880,AT5G41600,AT1G05720,AT1G52230,AT3G62250,AT3G11050,AT2G25930,AT2G31400,AT3G42970,AT2G26080,AT2G34580,AT4G39400,AT2G39450,AT5G39740,AT1G75210,AT1G66080,AT4G39150,AT5G19860,AT4G39235,AT5G19540,AT5G13090,AT3G28540,AT5G08410,AT2G31670,AT1G73760,AT3G52180,AT5G35970,AT2G42590,AT2G37630,AT3G52240,AT4G37320,AT2G04670,AT5G24150,AT1G35950,AT5G23760,AT1G74660,AT3G23940,AT4G34500,AT3G13430,AT5G19140,AT5G22340,AT1G80280,AT3G13470,AT1G70610,AT1G63830,AT4G33640,AT3G14090,AT1G69390,AT2G21970,AT3G28180,AT5G17630,AT4G32940,AT1G69780,AT5G17780,AT5G17310,AT2G45690,AT2G36240,AT2G30570,AT3G13070,AT5G16290,AT2G41720,AT2G22360,AT2G21200,AT5G14620,AT5G14760,AT1G29720,AT4G30900,AT2G32950,AT2G32910,AT1G79050,AT4G29750,AT1G08070,AT5G27650,AT1G02140,AT2G01800,AT1G22790,AT5G26790,AT4G28610,AT1G48090,AT5G11480,AT4G27800,AT1G78890,AT1G43715,AT5G09840,AT4G27585,AT5G24910,AT5G08580,AT4G26860,AT5G67020,AT1G27480,AT1G10200,AT1G53450,AT5G66060,AT5G65530,AT1G17460,AT3G19850,AT5G65310,AT3G20770,AT5G05170,AT5G04610,AT5G64460,AT1G09750,AT4G23820,AT5G63920,AT2G04390,AT1G22850,AT3G26080,AT1G05920,AT3G18215,AT1G26795,AT3G18050,AT1G08640,AT5G02810,AT2G17860,AT5G62740,AT5G62980,AT3G21690,AT5G61990,AT5G01990,AT1G63630,AT1G49660,AT1G24450,AT5G59540,AT5G59300,AT1G18310,AT1G55490,AT4G12590,AT3G60770,AT5G57990,AT1G76100,AT4G11590,AT4G11600,AT1G76240,AT1G25155,AT3G60620,AT3G61030,AT3G07880,AT1G08130,AT1G11545,AT3G08690,AT3G60320,AT1G50440,AT3G08640,AT4G09970,AT3G59930,AT4G10120,AT3G60020,AT4G08980,AT5G56260,AT4G07970,AT5G56030,AT4G05370,AT4G05100,AT5G54950,AT3G09970,AT1G71010,AT4G03030,AT3G55680,AT1G49270,AT3G55760,AT5G52390,AT4G02630,AT1G50320,AT3G07470,AT1G49250,AT1G11310,AT5G51460,AT3G04860
CTAGGGTN	10	2	['16-20']	201	AT4G01120,AT1G17100,AT4G00780,ATCG00160,AT1G31330,AT2G18640,AT5G49230,AT1G14040,AT1G16750,AT2G33240,AT1G66930,AT1G44446,AT5G46910,AT1G29900,AT1G18990,AT1G64770,AT1G15670,AT1G07020,AT4G16690,AT1G79500,AT4G17760,AT4G16520,AT3G47560,AT1G42970,AT1G75780,AT1G50940,AT2G18170,AT5G42765,AT1G71020,AT3G12290,AT2G25870,AT1G78450,AT2G19860,AT1G54880,AT5G41600,AT1G05720,AT1G52230,AT3G62250,AT3G11050,AT2G25930,AT2G31400,AT3G42970,AT2G26080,AT2G34580,AT4G39400,AT2G39450,AT5G39740,AT1G75210,AT1G66080,AT4G39150,AT5G19860,AT4G39235,AT5G19540,AT5G13090,AT3G28540,AT5G08410,AT2G31670,AT1G73760,AT3G52180,AT5G35970,AT2G42590,AT2G37630,AT3G52240,AT4G37320,AT2G04670,AT5G24150,AT1G35950,AT5G23760,AT1G74660,AT3G23940,AT4G34500,AT3G13430,AT5G19140,AT5G22340,AT1G80280,AT3G13470,AT1G70610,AT1G63830,AT4G33640,AT3G14090,AT1G69390,AT2G21970,AT3G28180,AT5G17630,AT4G32940,AT1G69780,AT5G17780,AT5G17310,AT2G45690,AT2G36240,AT2G30570,AT3G13070,AT5G16290,AT2G41720,AT2G22360,AT2G21200,AT5G14620,AT5G14760,AT1G29720,AT4G30900,AT2G32950,AT2G32910,AT1G79050,AT4G29750,AT1G08070,AT5G27650,AT1G02140,AT2G01800,AT1G22790,AT5G26790,AT4G28610,AT1G48090,AT5G11480,AT4G27800,AT1G78890,AT1G43715,AT5G09840,AT4G27585,AT5G24910,AT5G08580,AT4G26860,AT5G67020,AT1G27480,AT1G10200,AT1G53450,AT5G66060,AT5G65530,AT1G17460,AT3G19850,AT5G65310,AT3G20770,AT5G05170,AT5G04610,AT5G64460,AT1G09750,AT4G23820,AT5G63920,AT2G04390,AT1G22850,AT3G26080,AT1G05920,AT3G18215,AT1G26795,AT3G18050,AT1G08640,AT5G02810,AT2G17860,AT5G62740,AT5G62980,AT3G21690,AT5G61990,AT5G01990,AT1G63630,AT1G49660,AT1G24450,AT5G59540,AT5G59300,AT1G18310,AT1G55490,AT4G12590,AT3G60770,AT5G57990,AT1G76100,AT4G11590,AT4G11600,AT1G76240,AT1G25155,AT3G60620,AT3G61030,AT3G07880,AT1G08130,AT1G11545,AT3G08690,AT3G60320,AT1G50440,AT3G08640,AT4G09970,AT3G59930,AT4G10120,AT3G60020,AT4G08980,AT5G56260,AT4G07970,AT5G56030,AT4G05370,AT4G05100,AT5G54950,AT3G09970,AT1G71010,AT4G03030,AT3G55680,AT1G49270,AT3G55760,AT5G52390,AT4G02630,AT1G50320,AT3G07470,AT1G49250,AT1G11310,AT5G51460,AT3G04860

CTAGGGTN	10	3	['16-20']	201	AT4G01120,AT1G17100,AT4G00780,ATCG00160,AT1G31330,AT2G18640,AT5G49230,AT1G14040,AT1G16750,AT2G33240,AT1G66930,AT1G44446,AT5G46910,AT1G29900,AT1G18990,AT1G64770,AT1G15670,AT1G07020,AT4G16690,AT1G79500,AT4G17760,AT4G16520,AT3G47560,AT1G42970,AT1G75780,AT1G50940,AT2G18170,AT5G42765,AT1G71020,AT3G12290,AT2G25870,AT1G78450,AT2G19860,AT1G54880,AT5G41600,AT1G05720,AT1G52230,AT3G62250,AT3G11050,AT2G25930,AT2G31400,AT3G42970,AT2G26080,AT2G34580,AT4G39400,AT2G39450,AT5G39740,AT1G75210,AT1G66080,AT4G39150,AT5G19860,AT4G39235,AT5G19540,AT5G13090,AT3G28540,AT5G08410,AT2G31670,AT1G73760,AT3G52180,AT5G35970,AT2G42590,AT2G37630,AT3G52240,AT4G37320,AT2G04670,AT5G24150,AT1G35950,AT5G23760,AT1G74660,AT3G23940,AT4G34500,AT3G13430,AT5G19140,AT5G22340,AT1G80280,AT3G13470,AT1G70610,AT1G63830,AT4G33640,AT3G14090,AT1G69390,AT2G21970,AT3G28180,AT5G17630,AT4G32940,AT1G69780,AT5G17780,AT5G17310,AT2G45690,AT2G36240,AT2G30570,AT3G13070,AT5G16290,AT2G41720,AT2G22360,AT2G21200,AT5G14620,AT5G14760,AT1G29720,AT4G30900,AT2G32950,AT2G32910,AT1G79050,AT4G29750,AT1G08070,AT5G27650,AT1G02140,AT2G01800,AT1G22790,AT5G26790,AT4G28610,AT1G48090,AT5G11480,AT4G27800,AT1G78890,AT1G43715,AT5G09840,AT4G27585,AT5G24910,AT5G08580,AT4G26860,AT5G67020,AT1G27480,AT1G10200,AT1G53450,AT5G66060,AT5G65530,AT1G17460,AT3G19850,AT5G65310,AT3G20770,AT5G05170,AT5G04610,AT5G64460,AT1G09750,AT4G23820,AT5G63920,AT2G04390,AT1G22850,AT3G26080,AT1G05920,AT3G18215,AT1G26795,AT3G18050,AT1G08640,AT5G02810,AT2G17860,AT5G62740,AT5G62980,AT3G21690,AT5G61990,AT5G01990,AT1G63630,AT1G49660,AT1G24450,AT5G59540,AT5G59300,AT1G18310,AT1G55490,AT4G12590,AT3G60770,AT5G57990,AT1G76100,AT4G11590,AT4G11600,AT1G76240,AT1G25155,AT3G60620,AT3G61030,AT3G07880,AT1G08130,AT1G11545,AT3G08690,AT3G60320,AT1G50440,AT3G08640,AT4G09970,AT3G59930,AT4G10120,AT3G60020,AT4G08980,AT5G56260,AT4G07970,AT5G56030,AT4G05370,AT4G05100,AT5G54950,AT3G09970,AT1G71010,AT4G03030,AT3G55680,AT1G49270,AT3G55760,AT5G52390,AT4G02630,AT1G50320,AT3G07470,AT1G49250,AT1G11310,AT5G51460,AT3G04860
CTAGGGTN	10	4	['16-20']	201	AT4G01120,AT1G17100,AT4G00780,ATCG00160,AT1G31330,AT2G18640,AT5G49230,AT1G14040,AT1G16750,AT2G33240,AT1G66930,AT1G44446,AT5G46910,AT1G29900,AT1G18990,AT1G64770,AT1G15670,AT1G07020,AT4G16690,AT1G79500,AT4G17760,AT4G16520,AT3G47560,AT1G42970,AT1G75780,AT1G50940,AT2G18170,AT5G42765,AT1G71020,AT3G12290,AT2G25870,AT1G78450,AT2G19860,AT1G54880,AT5G41600,AT1G05720,AT1G52230,AT3G62250,AT3G11050,AT2G25930,AT2G31400,AT3G42970,AT2G26080,AT2G34580,AT4G39400,AT2G39450,AT5G39740,AT1G75210,AT1G66080,AT4G39150,AT5G19860,AT4G39235,AT5G19540,AT5G13090,AT3G28540,AT5G08410,AT2G31670,AT1G73760,AT3G52180,AT5G35970,AT2G42590,AT2G37630,AT3G52240,AT4G37320,AT2G04670,AT5G24150,AT1G35950,AT5G23760,AT1G74660,AT3G23940,AT4G34500,AT3G13430,AT5G19140,AT5G22340,AT1G80280,AT3G13470,AT1G70610,AT1G63830,AT4G33640,AT3G14090,AT1G69390,AT2G21970,AT3G28180,AT5G17630,AT4G32940,AT1G69780,AT5G17780,AT5G17310,AT2G45690,AT2G36240,AT2G30570,AT3G13070,AT5G16290,AT2G41720,AT2G22360,AT2G21200,AT5G14620,AT5G14760,AT1G29720,AT4G30900,AT2G32950,AT2G32910,AT1G79050,AT4G29750,AT1G08070,AT5G27650,AT1G02140,AT2G01800,AT1G22790,AT5G26790,AT4G28610,AT1G48090,AT5G11480,AT4G27800,AT1G78890,AT1G43715,AT5G09840,AT4G27585,AT5G24910,AT5G08580,AT4G26860,AT5G67020,AT1G27480,AT1G10200,AT1G53450,AT5G66060,AT5G65530,AT1G17460,AT3G19850,AT5G65310,AT3G20770,AT5G05170,AT5G04610,AT5G64460,AT1G09750,AT4G23820,AT5G63920,AT2G04390,AT1G22850,AT3G26080,AT1G05920,AT3G18215,AT1G26795,AT3G18050,AT1G08640,AT5G02810,AT2G17860,AT5G62740,AT5G62980,AT3G21690,AT5G61990,AT5G01990,AT1G63630,AT1G49660,AT1G24450,AT5G59540,AT5G59300,AT1G18310,AT1G55490,AT4G12590,AT3G60770,AT5G57990,AT1G76100,AT4G11590,AT4G11600,AT1G76240,AT1G25155,AT3G60620,AT3G61030,AT3G07880,AT1G08130,AT1G11545,AT3G08690,AT3G60320,AT1G50440,AT3G08640,AT4G09970,AT3G59930,AT4G10120,AT3G60020,AT4G08980,AT5G56260,AT4G07970,AT5G56030,AT4G05370,AT4G05100,AT5G54950,AT3G09970,AT1G71010,AT4G03030,AT3G55680,AT1G49270,AT3G55760,AT5G52390,AT4G02630,AT1G50320,AT3G07470,AT1G49250,AT1G11310,AT5G51460,AT3G04860

CTAGGGTN	10	5	['16-20']	201	AT4G01120,AT1G17100,AT4G00780,ATCG00160,AT1G31330,AT2G18640,AT5G49230,AT1G14040,AT1G16750,AT2G33240,AT1G66930,AT1G44446,AT5G46910,AT1G29900,AT1G18990,AT1G64770,AT1G15670,AT1G07020,AT4G16690,AT1G79500,AT4G17760,AT4G16520,AT3G47560,AT1G42970,AT1G75780,AT1G50940,AT2G18170,AT5G42765,AT1G71020,AT3G12290,AT2G25870,AT1G78450,AT2G19860,AT1G54880,AT5G41600,AT1G05720,AT1G52230,AT3G62250,AT3G11050,AT2G25930,AT2G31400,AT3G42970,AT2G26080,AT2G34580,AT4G39400,AT2G39450,AT5G39740,AT1G75210,AT1G66080,AT4G39150,AT5G19860,AT4G39235,AT5G19540,AT5G13090,AT3G28540,AT5G08410,AT2G31670,AT1G73760,AT3G52180,AT5G35970,AT2G42590,AT2G37630,AT3G52240,AT4G37320,AT2G04670,AT5G24150,AT1G35950,AT5G23760,AT1G74660,AT3G23940,AT4G34500,AT3G13430,AT5G19140,AT5G22340,AT1G80280,AT3G13470,AT1G70610,AT1G63830,AT4G33640,AT3G14090,AT1G69390,AT2G21970,AT3G28180,AT5G17630,AT4G32940,AT1G69780,AT5G17780,AT5G17310,AT2G45690,AT2G36240,AT2G30570,AT3G13070,AT5G16290,AT2G41720,AT2G22360,AT2G21200,AT5G14620,AT5G14760,AT1G29720,AT4G30900,AT2G32950,AT2G32910,AT1G79050,AT4G29750,AT1G08070,AT5G27650,AT1G02140,AT2G01800,AT1G22790,AT5G26790,AT4G28610,AT1G48090,AT5G11480,AT4G27800,AT1G78890,AT1G43715,AT5G09840,AT4G27585,AT5G24910,AT5G08580,AT4G26860,AT5G67020,AT1G27480,AT1G10200,AT1G53450,AT5G66060,AT5G65530,AT1G17460,AT3G19850,AT5G65310,AT3G20770,AT5G05170,AT5G04610,AT5G64460,AT1G09750,AT4G23820,AT5G63920,AT2G04390,AT1G22850,AT3G26080,AT1G05920,AT3G18215,AT1G26795,AT3G18050,AT1G08640,AT5G02810,AT2G17860,AT5G62740,AT5G62980,AT3G21690,AT5G61990,AT5G01990,AT1G63630,AT1G49660,AT1G24450,AT5G59540,AT5G59300,AT1G18310,AT1G55490,AT4G12590,AT3G60770,AT5G57990,AT1G76100,AT4G11590,AT4G11600,AT1G76240,AT1G25155,AT3G60620,AT3G61030,AT3G07880,AT1G08130,AT1G11545,AT3G08690,AT3G60320,AT1G50440,AT3G08640,AT4G09970,AT3G59930,AT4G10120,AT3G60020,AT4G08980,AT5G56260,AT4G07970,AT5G56030,AT4G05370,AT4G05100,AT5G54950,AT3G09970,AT1G71010,AT4G03030,AT3G55680,AT1G49270,AT3G55760,AT5G52390,AT4G02630,AT1G50320,AT3G07470,AT1G49250,AT1G11310,AT5G51460,AT3G04860
CTATAAATAC	10	1	['20-24']	38	AT2G45980,AT1G75210,AT5G01090,AT5G50180,AT1G13930,AT4G27790,AT3G28270,AT3G07650,AT2G37830,AT5G49360,AT5G08520,AT1G62780,AT5G12310,AT5G58410,AT3G50610,AT4G11320,AT1G71880,AT2G04670,AT1G53580,AT3G50660,AT1G80730,AT2G46400,AT2G06925,AT2G40060,AT5G18525,AT1G54520,AT5G54970,AT4G16480,AT5G17310,AT4G03510,AT4G32060,AT1G03055,AT1G78460,AT3G45060,AT5G14800,AT1G53210,AT5G30510,AT1G71820
CTATAAATAC	10	2	['20-24']	38	AT2G45980,AT1G75210,AT5G01090,AT5G50180,AT1G13930,AT4G27790,AT3G28270,AT3G07650,AT2G37830,AT5G49360,AT5G08520,AT1G62780,AT5G12310,AT5G58410,AT3G50610,AT4G11320,AT1G71880,AT2G04670,AT1G53580,AT3G50660,AT1G80730,AT2G46400,AT2G06925,AT2G40060,AT5G18525,AT1G54520,AT5G54970,AT4G16480,AT5G17310,AT4G03510,AT4G32060,AT1G03055,AT1G78460,AT3G45060,AT5G14800,AT1G53210,AT5G30510,AT1G71820
CTATAAATAC	10	3	['20-24']	38	AT2G45980,AT1G75210,AT5G01090,AT5G50180,AT1G13930,AT4G27790,AT3G28270,AT3G07650,AT2G37830,AT5G49360,AT5G08520,AT1G62780,AT5G12310,AT5G58410,AT3G50610,AT4G11320,AT1G71880,AT2G04670,AT1G53580,AT3G50660,AT1G80730,AT2G46400,AT2G06925,AT2G40060,AT5G18525,AT1G54520,AT5G54970,AT4G16480,AT5G17310,AT4G03510,AT4G32060,AT1G03055,AT1G78460,AT3G45060,AT5G14800,AT1G53210,AT5G30510,AT1G71820
CTATAAATAC	10	4	['20-24']	38	AT2G45980,AT1G75210,AT5G01090,AT5G50180,AT1G13930,AT4G27790,AT3G28270,AT3G07650,AT2G37830,AT5G49360,AT5G08520,AT1G62780,AT5G12310,AT5G58410,AT3G50610,AT4G11320,AT1G71880,AT2G04670,AT1G53580,AT3G50660,AT1G80730,AT2G46400,AT2G06925,AT2G40060,AT5G18525,AT1G54520,AT5G54970,AT4G16480,AT5G17310,AT4G03510,AT4G32060,AT1G03055,AT1G78460,AT3G45060,AT5G14800,AT1G53210,AT5G30510,AT1G71820
CTATAAATAC	10	5	['20-24']	38	AT2G45980,AT1G75210,AT5G01090,AT5G50180,AT1G13930,AT4G27790,AT3G28270,AT3G07650,AT2G37830,AT5G49360,AT5G08520,AT1G62780,AT5G12310,AT5G58410,AT3G50610,AT4G11320,AT1G71880,AT2G04670,AT1G53580,AT3G50660,AT1G80730,AT2G46400,AT2G06925,AT2G40060,AT5G18525,AT1G54520,AT5G54970,AT4G16480,AT5G17310,AT4G03510,AT4G32060,AT1G03055,AT1G78460,AT3G45060,AT5G14800,AT1G53210,AT5G30510,AT1G71820
CTCAAGTGA	10	1	['0-4', '4-8', '8-12', '12-16']	21	AT1G31500,AT1G58080,AT2G26520,AT3G02690,AT1G62620,AT5G37140,AT2G24930,AT3G50830,AT1G34160,AT1G19110,AT1G07260,AT3G19500,AT3G23660,AT5G53170,AT1G24764,AT3G02340,AT2G48020,AT4G30440,AT3G43680,AT3G04870,AT3G54300

CTCAAGTGA	10	2	['0-4', '4-8', '8-12', '12-16']	21	AT1G31500,AT1G58080,AT2G26520,AT3G02690,AT1G62620,AT5G37140,AT2G24930,AT3G50830,AT1G34160,AT1G19110,AT1G07260,AT3G19500,AT3G23660,AT5G53170,AT1G24764,AT3G02340,AT2G48020,AT4G30440,AT3G43680,AT3G04870,AT3G54300
CTCAAGTGA	10	3	['0-4', '4-8', '8-12', '12-16']	21	AT1G31500,AT1G58080,AT2G26520,AT3G02690,AT1G62620,AT5G37140,AT2G24930,AT3G50830,AT1G34160,AT1G19110,AT1G07260,AT3G19500,AT3G23660,AT5G53170,AT1G24764,AT3G02340,AT2G48020,AT4G30440,AT3G43680,AT3G04870,AT3G54300
CTCAAGTGA	10	4	['0-4', '4-8', '8-12', '12-16']	21	AT1G31500,AT1G58080,AT2G26520,AT3G02690,AT1G62620,AT5G37140,AT2G24930,AT3G50830,AT1G34160,AT1G19110,AT1G07260,AT3G19500,AT3G23660,AT5G53170,AT1G24764,AT3G02340,AT2G48020,AT4G30440,AT3G43680,AT3G04870,AT3G54300
CTCAAGTGA	10	5	['0-4', '4-8', '8-12', '12-16']	21	AT1G31500,AT1G58080,AT2G26520,AT3G02690,AT1G62620,AT5G37140,AT2G24930,AT3G50830,AT1G34160,AT1G19110,AT1G07260,AT3G19500,AT3G23660,AT5G53170,AT1G24764,AT3G02340,AT2G48020,AT4G30440,AT3G43680,AT3G04870,AT3G54300
CTCCAACC	10	1	['8-12', '12-16', '16-20']	53	AT3G15430,ATMG01040,AT2G15320,AT1G69040,AT5G26340,AT2G28720,AT2G18770,AT5G26230,AT1G43670,AT2G36630,AT3G02830,AT4G27030,AT1G10210,AT5G48140,AT3G50830,AT1G17880,AT1G76100,AT4G11600,AT1G22370,AT3G16910,AT1G13900,AT4G37250,AT5G56790,AT4G08970,AT1G07040,AT4G24460,AT4G16330,AT3G19450,AT2G36900,AT2G15570,AT2G04550,AT3G47620,AT4G16870,AT1G55330,AT3G28860,AT5G63590,AT1G77570,AT3G46170,AT3G24260,AT1G10920,AT3G17930,AT3G01990,AT4G14350,AT2G32640,AT1G79790,AT1G13860,AT1G03430,AT1G23080,AT5G13650,AT3G43310,ATMG01250,AT1G65430,AT5G12210
CTCCAACC	10	2	['8-12', '12-16', '16-20']	53	AT3G15430,ATMG01040,AT2G15320,AT1G69040,AT5G26340,AT2G28720,AT2G18770,AT5G26230,AT1G43670,AT2G36630,AT3G02830,AT4G27030,AT1G10210,AT5G48140,AT3G50830,AT1G17880,AT1G76100,AT4G11600,AT1G22370,AT3G16910,AT1G13900,AT4G37250,AT5G56790,AT4G08970,AT1G07040,AT4G24460,AT4G16330,AT3G19450,AT2G36900,AT2G15570,AT2G04550,AT3G47620,AT4G16870,AT1G55330,AT3G28860,AT5G63590,AT1G77570,AT3G46170,AT3G24260,AT1G10920,AT3G17930,AT3G01990,AT4G14350,AT2G32640,AT1G79790,AT1G13860,AT1G03430,AT1G23080,AT5G13650,AT3G43310,ATMG01250,AT1G65430,AT5G12210
CTCCAACC	10	3	['8-12', '12-16', '16-20']	53	AT3G15430,ATMG01040,AT2G15320,AT1G69040,AT5G26340,AT2G28720,AT2G18770,AT5G26230,AT1G43670,AT2G36630,AT3G02830,AT4G27030,AT1G10210,AT5G48140,AT3G50830,AT1G17880,AT1G76100,AT4G11600,AT1G22370,AT3G16910,AT1G13900,AT4G37250,AT5G56790,AT4G08970,AT1G07040,AT4G24460,AT4G16330,AT3G19450,AT2G36900,AT2G15570,AT2G04550,AT3G47620,AT4G16870,AT1G55330,AT3G28860,AT5G63590,AT1G77570,AT3G46170,AT3G24260,AT1G10920,AT3G17930,AT3G01990,AT4G14350,AT2G32640,AT1G79790,AT1G13860,AT1G03430,AT1G23080,AT5G13650,AT3G43310,ATMG01250,AT1G65430,AT5G12210
CTCCAACC	10	4	['8-12', '12-16', '16-20']	53	AT3G15430,ATMG01040,AT2G15320,AT1G69040,AT5G26340,AT2G28720,AT2G18770,AT5G26230,AT1G43670,AT2G36630,AT3G02830,AT4G27030,AT1G10210,AT5G48140,AT3G50830,AT1G17880,AT1G76100,AT4G11600,AT1G22370,AT3G16910,AT1G13900,AT4G37250,AT5G56790,AT4G08970,AT1G07040,AT4G24460,AT4G16330,AT3G19450,AT2G36900,AT2G15570,AT2G04550,AT3G47620,AT4G16870,AT1G55330,AT3G28860,AT5G63590,AT1G77570,AT3G46170,AT3G24260,AT1G10920,AT3G17930,AT3G01990,AT4G14350,AT2G32640,AT1G79790,AT1G13860,AT1G03430,AT1G23080,AT5G13650,AT3G43310,ATMG01250,AT1G65430,AT5G12210
CTCCAACC	10	5	['8-12', '12-16', '16-20']	53	AT3G15430,ATMG01040,AT2G15320,AT1G69040,AT5G26340,AT2G28720,AT2G18770,AT5G26230,AT1G43670,AT2G36630,AT3G02830,AT4G27030,AT1G10210,AT5G48140,AT3G50830,AT1G17880,AT1G76100,AT4G11600,AT1G22370,AT3G16910,AT1G13900,AT4G37250,AT5G56790,AT4G08970,AT1G07040,AT4G24460,AT4G16330,AT3G19450,AT2G36900,AT2G15570,AT2G04550,AT3G47620,AT4G16870,AT1G55330,AT3G28860,AT5G63590,AT1G77570,AT3G46170,AT3G24260,AT1G10920,AT3G17930,AT3G01990,AT4G14350,AT2G32640,AT1G79790,AT1G13860,AT1G03430,AT1G23080,AT5G13650,AT3G43310,ATMG01250,AT1G65430,AT5G12210

CTCCCAC	10	1	['20-24']	133	AT3G54050,AT4G01120,AT2G18790,AT5G50012,AT3G52720,AT5G49230,AT1G32060,AT5G49480,AT3G01180,AT2G45440,AT1G66940,AT1G18040,AT1G19140,AT1G15820,AT1G64800,AT1G79470,AT1G51790,AT1G56510,AT2G25850,AT3G45780,AT1G30360,AT1G72060,AT1G66840,AT2G16500,AT1G72300,AT4G39950,AT1G31410,AT1G75210,AT3G18850,AT3G52180,AT2G32560,AT3G27170,AT1G71880,AT2G22990,AT3G56700,AT1G80310,AT1G16570,AT1G58150,AT2G44300,AT1G73980,AT2G33860,AT5G17660,AT2G30490,AT5G16970,AT5G17050,AT2G35880,AT5G17310,AT1G10990,AT3G23690,AT5G16400,AT5G35220,AT1G55850,AT5G34830,AT5G13730,ATMG01180,AT5G27650,AT1G02140,AT5G12250,AT1G19540,AT1G19450,AT5G11930,AT4G28610,AT1G48210,AT5G11340,AT3G13370,AT5G25210,AT5G08520,AT5G08590,AT5G24910,AT2G25200,AT4G27030,AT5G07860,AT4G26130,AT5G66770,AT4G25970,AT1G07420,AT3G19880,AT4G24280,AT4G24350,AT4G24220,AT4G24470,AT4G23820,AT5G63940,AT3G14720,AT3G24590,AT1G53090,AT1G79770,AT5G63050,AT3G15840,AT5G62130,AT3G27690,AT5G01820,AT3G16110,AT3G15510,AT1G01790,AT3G63170,AT1G49750,AT3G62700,AT5G60210,AT1G01940,AT1G50030,AT3G02630,AT1G61520,AT3G06780,AT4G12730,AT1G31050,AT1G08490,AT5G58490,AT3G08010,AT5G58140,AT1G76240,AT1G76280,AT4G10840,AT2G18280,AT5G56520,AT2G16710,AT2G46490,AT5G54970,AT4G04630,AT3G10050,AT3G56940,AT5G54080,AT3G01990,AT3G56290,AT4G02910,AT4G02940,AT3G08920,AT3G08940,AT3G07310,AT4G02420,AT3G07470,AT3G05000,AT5G51770
CTCCCAC	10	2	['20-24']	133	AT3G54050,AT4G01120,AT2G18790,AT5G50012,AT3G52720,AT5G49230,AT1G32060,AT5G49480,AT3G01180,AT2G45440,AT1G66940,AT1G18040,AT1G19140,AT1G15820,AT1G64800,AT1G79470,AT1G51790,AT1G56510,AT2G25850,AT3G45780,AT1G30360,AT1G72060,AT1G66840,AT2G16500,AT1G72300,AT4G39950,AT1G31410,AT1G75210,AT3G18850,AT3G52180,AT2G32560,AT3G27170,AT1G71880,AT2G22990,AT3G56700,AT1G80310,AT1G16570,AT1G58150,AT2G44300,AT1G73980,AT2G33860,AT5G17660,AT2G30490,AT5G16970,AT5G17050,AT2G35880,AT5G17310,AT1G10990,AT3G23690,AT5G16400,AT5G35220,AT1G55850,AT5G34830,AT5G13730,ATMG01180,AT5G27650,AT1G02140,AT5G12250,AT1G19540,AT1G19450,AT5G11930,AT4G28610,AT1G48210,AT5G11340,AT3G13370,AT5G25210,AT5G08520,AT5G08590,AT5G24910,AT2G25200,AT4G27030,AT5G07860,AT4G26130,AT5G66770,AT4G25970,AT1G07420,AT3G19880,AT4G24280,AT4G24350,AT4G24220,AT4G24470,AT4G23820,AT5G63940,AT3G14720,AT3G24590,AT1G53090,AT1G79770,AT5G63050,AT3G15840,AT5G62130,AT3G27690,AT5G01820,AT3G16110,AT3G15510,AT1G01790,AT3G63170,AT1G49750,AT3G62700,AT5G60210,AT1G01940,AT1G50030,AT3G02630,AT1G61520,AT3G06780,AT4G12730,AT1G31050,AT1G08490,AT5G58490,AT3G08010,AT5G58140,AT1G76240,AT1G76280,AT4G10840,AT2G18280,AT5G56520,AT2G16710,AT2G46490,AT5G54970,AT4G04630,AT3G10050,AT3G56940,AT5G54080,AT3G01990,AT3G56290,AT4G02910,AT4G02940,AT3G08920,AT3G08940,AT3G07310,AT4G02420,AT3G07470,AT3G05000,AT5G51770
CTCCCAC	10	3	['20-24']	133	AT3G54050,AT4G01120,AT2G18790,AT5G50012,AT3G52720,AT5G49230,AT1G32060,AT5G49480,AT3G01180,AT2G45440,AT1G66940,AT1G18040,AT1G19140,AT1G15820,AT1G64800,AT1G79470,AT1G51790,AT1G56510,AT2G25850,AT3G45780,AT1G30360,AT1G72060,AT1G66840,AT2G16500,AT1G72300,AT4G39950,AT1G31410,AT1G75210,AT3G18850,AT3G52180,AT2G32560,AT3G27170,AT1G71880,AT2G22990,AT3G56700,AT1G80310,AT1G16570,AT1G58150,AT2G44300,AT1G73980,AT2G33860,AT5G17660,AT2G30490,AT5G16970,AT5G17050,AT2G35880,AT5G17310,AT1G10990,AT3G23690,AT5G16400,AT5G35220,AT1G55850,AT5G34830,AT5G13730,ATMG01180,AT5G27650,AT1G02140,AT5G12250,AT1G19540,AT1G19450,AT5G11930,AT4G28610,AT1G48210,AT5G11340,AT3G13370,AT5G25210,AT5G08520,AT5G08590,AT5G24910,AT2G25200,AT4G27030,AT5G07860,AT4G26130,AT5G66770,AT4G25970,AT1G07420,AT3G19880,AT4G24280,AT4G24350,AT4G24220,AT4G24470,AT4G23820,AT5G63940,AT3G14720,AT3G24590,AT1G53090,AT1G79770,AT5G63050,AT3G15840,AT5G62130,AT3G27690,AT5G01820,AT3G16110,AT3G15510,AT1G01790,AT3G63170,AT1G49750,AT3G62700,AT5G60210,AT1G01940,AT1G50030,AT3G02630,AT1G61520,AT3G06780,AT4G12730,AT1G31050,AT1G08490,AT5G58490,AT3G08010,AT5G58140,AT1G76240,AT1G76280,AT4G10840,AT2G18280,AT5G56520,AT2G16710,AT2G46490,AT5G54970,AT4G04630,AT3G10050,AT3G56940,AT5G54080,AT3G01990,AT3G56290,AT4G02910,AT4G02940,AT3G08920,AT3G08940,AT3G07310,AT4G02420,AT3G07470,AT3G05000,AT5G51770

CTCCCAC	10	4	['20-24']	133	AT3G54050,AT4G01120,AT2G18790,AT5G50012,AT3G52720,AT5G49230,AT1G32060,AT5G49480,AT3G01180,AT2G45440,AT1G66940,AT1G18040,AT1G19140,AT1G15820,AT1G64800,AT1G79470,AT1G51790,AT1G56510,AT2G25850,AT3G45780,AT1G30360,AT1G72060,AT1G66840,AT2G16500,AT1G72300,AT4G39950,AT1G31410,AT1G75210,AT3G18850,AT3G52180,AT2G32560,AT3G27170,AT1G71880,AT2G22990,AT3G56700,AT1G80310,AT1G16570,AT1G58150,AT2G44300,AT1G73980,AT2G33860,AT5G17660,AT2G30490,AT5G16970,AT5G17050,AT2G35880,AT5G17310,AT1G10990,AT3G23690,AT5G16400,AT5G35220,AT1G55850,AT5G34830,AT5G13730,ATMG01180,AT5G27650,AT1G02140,AT5G12250,AT1G19540,AT1G19450,AT5G11930,AT4G28610,AT1G48210,AT5G11340,AT3G13370,AT5G25210,AT5G08520,AT5G08590,AT5G24910,AT2G25200,AT4G27030,AT5G07860,AT4G26130,AT5G66770,AT4G25970,AT1G07420,AT3G19880,AT4G24280,AT4G24350,AT4G24220,AT4G24470,AT4G23820,AT5G63940,AT3G14720,AT3G24590,AT1G53090,AT1G79770,AT5G63050,AT3G15840,AT5G62130,AT3G27690,AT5G01820,AT3G16110,AT3G15510,AT1G01790,AT3G63170,AT1G49750,AT3G62700,AT5G60210,AT1G01940,AT1G50030,AT3G02630,AT1G61520,AT3G06780,AT4G12730,AT1G31050,AT1G08490,AT5G58490,AT3G08010,AT5G58140,AT1G76240,AT1G76280,AT4G10840,AT2G18280,AT5G56520,AT2G16710,AT2G46490,AT5G54970,AT4G04630,AT3G10050,AT3G56940,AT5G54080,AT3G01990,AT3G56290,AT4G02910,AT4G02940,AT3G08920,AT3G08940,AT3G07310,AT4G02420,AT3G07470,AT3G05000,AT5G51770
CTCCCAC	10	5	['20-24']	133	AT3G54050,AT4G01120,AT2G18790,AT5G50012,AT3G52720,AT5G49230,AT1G32060,AT5G49480,AT3G01180,AT2G45440,AT1G66940,AT1G18040,AT1G19140,AT1G15820,AT1G64800,AT1G79470,AT1G51790,AT1G56510,AT2G25850,AT3G45780,AT1G30360,AT1G72060,AT1G66840,AT2G16500,AT1G72300,AT4G39950,AT1G31410,AT1G75210,AT3G18850,AT3G52180,AT2G32560,AT3G27170,AT1G71880,AT2G22990,AT3G56700,AT1G80310,AT1G16570,AT1G58150,AT2G44300,AT1G73980,AT2G33860,AT5G17660,AT2G30490,AT5G16970,AT5G17050,AT2G35880,AT5G17310,AT1G10990,AT3G23690,AT5G16400,AT5G35220,AT1G55850,AT5G34830,AT5G13730,ATMG01180,AT5G27650,AT1G02140,AT5G12250,AT1G19540,AT1G19450,AT5G11930,AT4G28610,AT1G48210,AT5G11340,AT3G13370,AT5G25210,AT5G08520,AT5G08590,AT5G24910,AT2G25200,AT4G27030,AT5G07860,AT4G26130,AT5G66770,AT4G25970,AT1G07420,AT3G19880,AT4G24280,AT4G24350,AT4G24220,AT4G24470,AT4G23820,AT5G63940,AT3G14720,AT3G24590,AT1G53090,AT1G79770,AT5G63050,AT3G15840,AT5G62130,AT3G27690,AT5G01820,AT3G16110,AT3G15510,AT1G01790,AT3G63170,AT1G49750,AT3G62700,AT5G60210,AT1G01940,AT1G50030,AT3G02630,AT1G61520,AT3G06780,AT4G12730,AT1G31050,AT1G08490,AT5G58490,AT3G08010,AT5G58140,AT1G76240,AT1G76280,AT4G10840,AT2G18280,AT5G56520,AT2G16710,AT2G46490,AT5G54970,AT4G04630,AT3G10050,AT3G56940,AT5G54080,AT3G01990,AT3G56290,AT4G02910,AT4G02940,AT3G08920,AT3G08940,AT3G07310,AT4G02420,AT3G07470,AT3G05000,AT5G51770
CTCCTAATT	10	1	['4-8', '8-12']	31	AT3G42100,AT1G31500,AT4G28130,AT1G14110,AT5G58760,AT5G49240,AT2G02160,AT2G02040,ATMG00610,AT2G37520,AT2G01940,AT4G10750,AT1G35950,AT3G59480,AT1G15820,AT1G64860,AT3G28715,AT2G06050,AT2G29670,AT5G54250,AT2G35450,AT2G28200,AT1G77740,AT3G46510,AT3G12110,AT5G15020,AT3G06250,AT3G15610,AT3G06160,AT5G40850,AT3G15510
CTCCTAATT	10	2	['4-8', '8-12']	31	AT3G42100,AT1G31500,AT4G28130,AT1G14110,AT5G58760,AT5G49240,AT2G02160,AT2G02040,ATMG00610,AT2G37520,AT2G01940,AT4G10750,AT1G35950,AT3G59480,AT1G15820,AT1G64860,AT3G28715,AT2G06050,AT2G29670,AT5G54250,AT2G35450,AT2G28200,AT1G77740,AT3G46510,AT3G12110,AT5G15020,AT3G06250,AT3G15610,AT3G06160,AT5G40850,AT3G15510
CTCCTAATT	10	3	['4-8', '8-12']	31	AT3G42100,AT1G31500,AT4G28130,AT1G14110,AT5G58760,AT5G49240,AT2G02160,AT2G02040,ATMG00610,AT2G37520,AT2G01940,AT4G10750,AT1G35950,AT3G59480,AT1G15820,AT1G64860,AT3G28715,AT2G06050,AT2G29670,AT5G54250,AT2G35450,AT2G28200,AT1G77740,AT3G46510,AT3G12110,AT5G15020,AT3G06250,AT3G15610,AT3G06160,AT5G40850,AT3G15510
CTCCTAATT	10	4	['4-8', '8-12']	31	AT3G42100,AT1G31500,AT4G28130,AT1G14110,AT5G58760,AT5G49240,AT2G02160,AT2G02040,ATMG00610,AT2G37520,AT2G01940,AT4G10750,AT1G35950,AT3G59480,AT1G15820,AT1G64860,AT3G28715,AT2G06050,AT2G29670,AT5G54250,AT2G35450,AT2G28200,AT1G77740,AT3G46510,AT3G12110,AT5G15020,AT3G06250,AT3G15610,AT3G06160,AT5G40850,AT3G15510
CTCCTAATT	10	5	['4-8', '8-12']	31	AT3G42100,AT1G31500,AT4G28130,AT1G14110,AT5G58760,AT5G49240,AT2G02160,AT2G02040,ATMG00610,AT2G37520,AT2G01940,AT4G10750,AT1G35950,AT3G59480,AT1G15820,AT1G64860,AT3G28715,AT2G06050,AT2G29670,AT5G54250,AT2G35450,AT2G28200,AT1G77740,AT3G46510,AT3G12110,AT5G15020,AT3G06250,AT3G15610,AT3G06160,AT5G40850,AT3G15510

CTCCTACC	10	1	['20-24']	39	AT1G37537,AT1G49660,AT5G11480,AT1G67470,AT2G40780,AT4G27790,AT1G09160,AT4G13930,AT2G45380,AT1G06570,AT1G43755,AT2G45270,AT3G61970,AT2G41090,AT2G41430,AT1G66980,AT2G42900,AT5G47870,AT5G47240,AT5G65730,AT3G19820,AT5G19140,AT5G64860,AT1G13540,AT5G03860,AT4G16870,AT3G09970,AT2G30490,AT1G36980,AT1G08550,AT1G78600,AT2G03750,AT1G73390,AT1G11130,AT5G01820,AT5G01950,AT3G54680,AT1G65430,AT1G02160
CTCCTACC	10	3	['20-24']	39	AT1G37537,AT1G49660,AT5G11480,AT1G67470,AT2G40780,AT4G27790,AT1G09160,AT4G13930,AT2G45380,AT1G06570,AT1G43755,AT2G45270,AT3G61970,AT2G41090,AT2G41430,AT1G66980,AT2G42900,AT5G47870,AT5G47240,AT5G65730,AT3G19820,AT5G19140,AT5G64860,AT1G13540,AT5G03860,AT4G16870,AT3G09970,AT2G30490,AT1G36980,AT1G08550,AT1G78600,AT2G03750,AT1G73390,AT1G11130,AT5G01820,AT5G01950,AT3G54680,AT1G65430,AT1G02160
CTCCTACC	10	4	['20-24']	39	AT1G37537,AT1G49660,AT5G11480,AT1G67470,AT2G40780,AT4G27790,AT1G09160,AT4G13930,AT2G45380,AT1G06570,AT1G43755,AT2G45270,AT3G61970,AT2G41090,AT2G41430,AT1G66980,AT2G42900,AT5G47870,AT5G47240,AT5G65730,AT3G19820,AT5G19140,AT5G64860,AT1G13540,AT5G03860,AT4G16870,AT3G09970,AT2G30490,AT1G36980,AT1G08550,AT1G78600,AT2G03750,AT1G73390,AT1G11130,AT5G01820,AT5G01950,AT3G54680,AT1G65430,AT1G02160
CTCCTACC	10	5	['20-24']	39	AT1G37537,AT1G49660,AT5G11480,AT1G67470,AT2G40780,AT4G27790,AT1G09160,AT4G13930,AT2G45380,AT1G06570,AT1G43755,AT2G45270,AT3G61970,AT2G41090,AT2G41430,AT1G66980,AT2G42900,AT5G47870,AT5G47240,AT5G65730,AT3G19820,AT5G19140,AT5G64860,AT1G13540,AT5G03860,AT4G16870,AT3G09970,AT2G30490,AT1G36980,AT1G08550,AT1G78600,AT2G03750,AT1G73390,AT1G11130,AT5G01820,AT5G01950,AT3G54680,AT1G65430,AT1G02160
CTGAAGAAGAA	10	1	['0-4', '8-12', '12-16', '16-20']	20	AT1G63440,AT4G40040,AT1G71010,AT2G19270,AT1G36980,AT5G67480,AT5G42720,AT2G20480,AT5G47890,AT4G38940,AT3G01790,AT1G07050,AT1G67970,AT4G33500,AT2G18280,AT5G62130,AT1G09415,AT1G60000,AT3G10020,AT3G10720
CTGAAGAAGAA	10	2	['0-4', '8-12', '16-20', '12-16']	20	AT1G63440,AT4G40040,AT1G71010,AT2G19270,AT1G36980,AT5G67480,AT5G42720,AT2G20480,AT5G47890,AT4G38940,AT3G01790,AT1G07050,AT1G67970,AT4G33500,AT2G18280,AT5G62130,AT1G09415,AT1G60000,AT3G10020,AT3G10720
CTGAAGAAGAA	10	3	['0-4', '8-12', '12-16', '16-20']	20	AT1G63440,AT4G40040,AT1G71010,AT2G19270,AT1G36980,AT5G67480,AT5G42720,AT2G20480,AT5G47890,AT4G38940,AT3G01790,AT1G07050,AT1G67970,AT4G33500,AT2G18280,AT5G62130,AT1G09415,AT1G60000,AT3G10020,AT3G10720
CTGAAGAAGAA	10	4	['0-4', '8-12', '12-16', '16-20']	20	AT1G63440,AT4G40040,AT1G71010,AT2G19270,AT1G36980,AT5G67480,AT5G42720,AT2G20480,AT5G47890,AT4G38940,AT3G01790,AT1G07050,AT1G67970,AT4G33500,AT2G18280,AT5G62130,AT1G09415,AT1G60000,AT3G10020,AT3G10720
CTGAAGAAGAA	10	5	['8-12', '12-16', '16-20', '0-4']	20	AT1G63440,AT4G40040,AT1G71010,AT2G19270,AT1G36980,AT5G67480,AT5G42720,AT2G20480,AT5G47890,AT4G38940,AT3G01790,AT1G07050,AT1G67970,AT4G33500,AT2G18280,AT5G62130,AT1G09415,AT1G60000,AT3G10020,AT3G10720

CTTATCCN	10	1	['8-12', '20-24']	290	ATMG00650,AT3G54050,AT3G01310,AT3G54220,AT1G31330,AT1G34220,AT5G50680,AT3G53500,AT3G01500,AT2G26530,AT2G26520,AT1G15290,AT3G01660,AT2G18670,AT3G52840,AT2G18700,AT2G41680,AT2G40700,AT1G32060,AT3G52070,AT3G07640,AT1G62750,AT5G49360,AT3G01180,AT5G49120,AT3G05130,AT1G75950,AT3G01090,AT3G51430,AT5G47910,AT1G28600,AT3G16320,AT4G25530,AT1G06410,AT1G28580,AT1G02305,AT3G50530,AT3G50610,AT3G04870,AT1G10740,AT1G01620,AT3G48580,AT1G14920,AT2G07140,AT1G07040,AT1G64720,AT4G16690,AT3G48140,AT4G16330,AT1G79500,AT1G21300,AT3G47340,AT2G24050,AT2G35500,AT1G56510,AT5G43930,AT1G30200,AT4G16480,AT1G55330,AT5G43830,AT3G47160,AT2G18170,AT1G56220,AT1G03130,AT1G71020,AT1G71180,AT4G14270,AT2G25870,AT1G29510,AT1G24764,AT3G45690,AT1G64330,AT3G45060,AT2G46780,AT1G66820,AT1G05720,AT3G06180,AT1G52230,AT2G16530,AT2G25950,AT1G66890,AT2G26900,AT3G42630,AT5G40500,AT2G38820,AT1G32130,AT2G10370,AT2G15320,AT3G13080,AT4G39710,AT2G05710,AT1G26220,AT5G39080,AT5G11060,AT5G11070,AT3G32930,AT3G28340,AT5G37540,AT2G28630,AT2G41430,AT2G23600,AT4G37870,AT3G20680,AT1G35460,AT4G37290,AT5G35630,AT4G37680,AT5G24490,AT5G20935,AT5G24620,AT1G71880,AT1G52760,AT2G25210,AT2G30990,AT3G27170,AT1G67623,AT4G36390,AT4G36790,AT4G36600,AT4G37250,AT4G35770,AT2G22990,AT4G37260,AT2G22980,AT2G19450,AT5G23140,AT3G13430,AT1G80530,AT1G16680,AT5G22340,AT1G80310,AT5G19010,AT5G18850,AT3G14090,AT5G18640,AT5G18680,AT3G15290,AT4G33500,AT2G33860,AT5G17780,AT2G33830,AT2G02710,AT2G36240,AT4G32530,AT5G15770,AT4G32270,AT2G32640,AT2G47320,AT5G16560,AT5G15580,AT5G14620,AT3G24170,AT2G42190,AT5G35170,AT5G35220,AT2G10850,AT5G14180,AT1G55960,AT1G55840,AT3G15570,AT5G27720,AT1G32360,AT5G11670,AT5G27290,AT5G11740,AT5G26930,AT1G32310,AT1G69040,AT5G10860,AT1G49010,AT4G27800,AT5G26030,AT4G28080,AT1G78890,AT3G26280,AT1G78260,AT4G27410,AT4G27450,AT5G08590,AT5G24930,AT1G21500,AT4G27320,AT3G23080,AT3G26650,AT4G26520,AT1G06020,AT4G26560,AT4G26530,AT3G26740,AT3G17750,AT5G66570,AT1G26590,AT3G16940,AT1G01240,AT5G06550,AT5G65910,AT1G09570,AT5G66060,AT1G01320,AT4G25660,AT3G23160,AT3G19930,AT5G65380,AT1G19660,AT5G05200,AT5G64630,AT5G04490,AT1G09750,AT5G04590,AT4G24190,AT1G70000,AT5G64180,AT5G63920,AT4G23940,AT3G16220,AT5G04040,AT1G62290,AT3G14640,AT5G63780,AT5G63810,AT1G26800,AT1G08570,AT3G18080,AT5G03350,AT5G63380,AT1G08640,AT5G02810,AT5G02490,AT2G17330,AT3G15630,AT5G02160,AT3G15850,AT5G62360,AT5G60680,AT1G49750,AT3G62910,AT3G22430,AT3G22420,AT3G62580,AT1G61590,AT5G60210,AT1G01940,AT3G62860,AT3G62100,AT5G59140,AT5G59080,AT1G47290,AT5G59180,AT5G58770,AT3G02750,AT2G36800,AT3G61430,AT1G08380,AT1G76180,AT5G58380,AT3G60770,AT4G11590,AT5G58070,AT3G07890,AT3G08010,AT5G57815,AT3G08590,AT4G10920,AT3G21390,AT3G11800,AT2G18290,AT2G16600,AT3G10720,AT3G10740,AT3G59400,AT2G46550,AT2G15890,AT5G55120,AT4G05050,AT3G31980,AT3G10020,AT1G70820,AT3G08890,AT5G53500,AT3G55670,AT5G52580,AT2G06630,AT3G07310,AT5G52320,AT3G03320,AT3G54990,AT2G42390,AT5G51460,AT3G54810,AT1G17160

CTTATCCN	10	2	['8-12', '20-24']	290	ATMG00650,AT3G54050,AT3G01310,AT3G54220,AT1G31330,AT1G34220,AT5G50680,AT3G53500,AT3G01500,AT2G26530,AT2G26520,AT1G15290,AT3G01660,AT2G18670,AT3G52840,AT2G18700,AT2G41680,AT2G40700,AT1G32060,AT3G52070,AT3G07640,AT1G62750,AT5G49360,AT3G01180,AT5G49120,AT3G05130,AT1G75950,AT3G01090,AT3G51430,AT5G47910,AT1G28600,AT3G16320,AT4G25530,AT1G06410,AT1G28580,AT1G02305,AT3G50530,AT3G50610,AT3G04870,AT1G10740,AT1G01620,AT3G48580,AT1G14920,AT2G07140,AT1G07040,AT1G64720,AT4G16690,AT3G48140,AT4G16330,AT1G79500,AT1G21300,AT3G47340,AT2G24050,AT2G35500,AT1G56510,AT5G43930,AT1G30200,AT4G16480,AT1G55330,AT5G43830,AT3G47160,AT2G18170,AT1G56220,AT1G03130,AT1G71020,AT1G71180,AT4G14270,AT2G25870,AT1G29510,AT1G24764,AT3G45690,AT1G64330,AT3G45060,AT2G46780,AT1G66820,AT1G05720,AT3G06180,AT1G52230,AT2G16530,AT2G25950,AT1G66890,AT2G26900,AT3G42630,AT5G40500,AT2G38820,AT1G32130,AT2G10370,AT2G15320,AT3G13080,AT4G39710,AT2G05710,AT1G26220,AT5G39080,AT5G11060,AT5G11070,AT3G32930,AT3G28340,AT5G37540,AT2G28630,AT2G41430,AT2G23600,AT4G37870,AT3G20680,AT1G35460,AT4G37290,AT5G35630,AT4G37680,AT5G24490,AT5G20935,AT5G24620,AT1G71880,AT1G52760,AT2G25210,AT2G30990,AT3G27170,AT1G67623,AT4G36390,AT4G36790,AT4G36600,AT4G37250,AT4G35770,AT2G22990,AT4G37260,AT2G22980,AT2G19450,AT5G23140,AT3G13430,AT1G80530,AT1G16680,AT5G22340,AT1G80310,AT5G19010,AT5G18850,AT3G14090,AT5G18640,AT5G18680,AT3G15290,AT4G33500,AT2G33860,AT5G17780,AT2G33830,AT2G02710,AT2G36240,AT4G32530,AT5G15770,AT4G32270,AT2G32640,AT2G47320,AT5G16560,AT5G15580,AT5G14620,AT3G24170,AT2G42190,AT5G35170,AT5G35220,AT2G10850,AT5G14180,AT1G55960,AT1G55840,AT3G15570,AT5G27720,AT1G32360,AT5G11670,AT5G27290,AT5G11740,AT5G26930,AT1G32310,AT1G69040,AT5G10860,AT1G49010,AT4G27800,AT5G26030,AT4G28080,AT1G78890,AT3G26280,AT1G78260,AT4G27410,AT4G27450,AT5G08590,AT5G24930,AT1G21500,AT4G27320,AT3G23080,AT3G26650,AT4G26520,AT1G06020,AT4G26560,AT4G26530,AT3G26740,AT3G17750,AT5G66570,AT1G26590,AT3G16940,AT1G01240,AT5G06550,AT5G65910,AT1G09570,AT5G66060,AT1G01320,AT4G25660,AT3G23160,AT3G19930,AT5G65380,AT1G19660,AT5G05200,AT5G64630,AT5G04490,AT1G09750,AT5G04590,AT4G24190,AT1G70000,AT5G64180,AT5G63920,AT4G23940,AT3G16220,AT5G04040,AT1G62290,AT3G14640,AT5G63780,AT5G63810,AT1G26800,AT1G08570,AT3G18080,AT5G03350,AT5G63380,AT1G08640,AT5G02810,AT5G02490,AT2G17330,AT3G15630,AT5G02160,AT3G15850,AT5G62360,AT5G60680,AT1G49750,AT3G62910,AT3G22430,AT3G22420,AT3G62580,AT1G61590,AT5G60210,AT1G01940,AT3G62860,AT3G62100,AT5G59140,AT5G59080,AT1G47290,AT5G59180,AT5G58770,AT3G02750,AT2G36800,AT3G61430,AT1G08380,AT1G76180,AT5G58380,AT3G60770,AT4G11590,AT5G58070,AT3G07890,AT3G08010,AT5G57815,AT3G08590,AT4G10920,AT3G21390,AT3G11800,AT2G18290,AT2G16600,AT3G10720,AT3G10740,AT3G59400,AT2G46550,AT2G15890,AT5G55120,AT4G05050,AT3G31980,AT3G10020,AT1G70820,AT3G08890,AT5G53500,AT3G55670,AT5G52580,AT2G06630,AT3G07310,AT5G52320,AT3G03320,AT3G54990,AT2G42390,AT5G51460,AT3G54810,AT1G17160

CTTATCCN	10	3	['8-12', '20-24']	290	ATMG00650,AT3G54050,AT3G01310,AT3G54220,AT1G31330,AT1G34220,AT5G50680,AT3G53500,AT3G01500,AT2G26530,AT2G26520,AT1G15290,AT3G01660,AT2G18670,AT3G52840,AT2G18700,AT2G41680,AT2G40700,AT1G32060,AT3G52070,AT3G07640,AT1G62750,AT5G49360,AT3G01180,AT5G49120,AT3G05130,AT1G75950,AT3G01090,AT3G51430,AT5G47910,AT1G28600,AT3G16320,AT4G25530,AT1G06410,AT1G28580,AT1G02305,AT3G50530,AT3G50610,AT3G04870,AT1G10740,AT1G01620,AT3G48580,AT1G14920,AT2G07140,AT1G07040,AT1G64720,AT4G16690,AT3G48140,AT4G16330,AT1G79500,AT1G21300,AT3G47340,AT2G24050,AT2G35500,AT1G56510,AT5G43930,AT1G30200,AT4G16480,AT1G55330,AT5G43830,AT3G47160,AT2G18170,AT1G56220,AT1G03130,AT1G71020,AT1G71180,AT4G14270,AT2G25870,AT1G29510,AT1G24764,AT3G45690,AT1G64330,AT3G45060,AT2G46780,AT1G66820,AT1G05720,AT3G06180,AT1G52230,AT2G16530,AT2G25950,AT1G66890,AT2G26900,AT3G42630,AT5G40500,AT2G38820,AT1G32130,AT2G10370,AT2G15320,AT3G13080,AT4G39710,AT2G05710,AT1G26220,AT5G39080,AT5G11060,AT5G11070,AT3G32930,AT3G28340,AT5G37540,AT2G28630,AT2G41430,AT2G23600,AT4G37870,AT3G20680,AT1G35460,AT4G37290,AT5G35630,AT4G37680,AT5G24490,AT5G20935,AT5G24620,AT1G71880,AT1G52760,AT2G25210,AT2G30990,AT3G27170,AT1G67623,AT4G36390,AT4G36790,AT4G36600,AT4G37250,AT4G35770,AT2G22990,AT4G37260,AT2G22980,AT2G19450,AT5G23140,AT3G13430,AT1G80530,AT1G16680,AT5G22340,AT1G80310,AT5G19010,AT5G18850,AT3G14090,AT5G18640,AT5G18680,AT3G15290,AT4G33500,AT2G33860,AT5G17780,AT2G33830,AT2G02710,AT2G36240,AT4G32530,AT5G15770,AT4G32270,AT2G32640,AT2G47320,AT5G16560,AT5G15580,AT5G14620,AT3G24170,AT2G42190,AT5G35170,AT5G35220,AT2G10850,AT5G14180,AT1G55960,AT1G55840,AT3G15570,AT5G27720,AT1G32360,AT5G11670,AT5G27290,AT5G11740,AT5G26930,AT1G32310,AT1G69040,AT5G10860,AT1G49010,AT4G27800,AT5G26030,AT4G28080,AT1G78890,AT3G26280,AT1G78260,AT4G27410,AT4G27450,AT5G08590,AT5G24930,AT1G21500,AT4G27320,AT3G23080,AT3G26650,AT4G26520,AT1G06020,AT4G26560,AT4G26530,AT3G26740,AT3G17750,AT5G66570,AT1G26590,AT3G16940,AT1G01240,AT5G06550,AT5G65910,AT1G09570,AT5G66060,AT1G01320,AT4G25660,AT3G23160,AT3G19930,AT5G65380,AT1G19660,AT5G05200,AT5G64630,AT5G04490,AT1G09750,AT5G04590,AT4G24190,AT1G70000,AT5G64180,AT5G63920,AT4G23940,AT3G16220,AT5G04040,AT1G62290,AT3G14640,AT5G63780,AT5G63810,AT1G26800,AT1G08570,AT3G18080,AT5G03350,AT5G63380,AT1G08640,AT5G02810,AT5G02490,AT2G17330,AT3G15630,AT5G02160,AT3G15850,AT5G62360,AT5G60680,AT1G49750,AT3G62910,AT3G22430,AT3G22420,AT3G62580,AT1G61590,AT5G60210,AT1G01940,AT3G62860,AT3G62100,AT5G59140,AT5G59080,AT1G47290,AT5G59180,AT5G58770,AT3G02750,AT2G36800,AT3G61430,AT1G08380,AT1G76180,AT5G58380,AT3G60770,AT4G11590,AT5G58070,AT3G07890,AT3G08010,AT5G57815,AT3G08590,AT4G10920,AT3G21390,AT3G11800,AT2G18290,AT2G16600,AT3G10720,AT3G10740,AT3G59400,AT2G46550,AT2G15890,AT5G55120,AT4G05050,AT3G31980,AT3G10020,AT1G70820,AT3G08890,AT5G53500,AT3G55670,AT5G52580,AT2G06630,AT3G07310,AT5G52320,AT3G03320,AT3G54990,AT2G42390,AT5G51460,AT3G54810,AT1G17160

CTTATCCN	10	4	['8-12', '20-24']	290	ATMG00650,AT3G54050,AT3G01310,AT3G54220,AT1G31330,AT1G34220,AT5G50680,AT3G53500,AT3G01500,AT2G26530,AT2G26520,AT1G15290,AT3G01660,AT2G18670,AT3G52840,AT2G18700,AT2G41680,AT2G40700,AT1G32060,AT3G52070,AT3G07640,AT1G62750,AT5G49360,AT3G01180,AT5G49120,AT3G05130,AT1G75950,AT3G01090,AT3G51430,AT5G47910,AT1G28600,AT3G16320,AT4G25530,AT1G06410,AT1G28580,AT1G02305,AT3G50530,AT3G50610,AT3G04870,AT1G10740,AT1G01620,AT3G48580,AT1G14920,AT2G07140,AT1G07040,AT1G64720,AT4G16690,AT3G48140,AT4G16330,AT1G79500,AT1G21300,AT3G47340,AT2G24050,AT2G35500,AT1G56510,AT5G43930,AT1G30200,AT4G16480,AT1G55330,AT5G43830,AT3G47160,AT2G18170,AT1G56220,AT1G03130,AT1G71020,AT1G71180,AT4G14270,AT2G25870,AT1G29510,AT1G24764,AT3G45690,AT1G64330,AT3G45060,AT2G46780,AT1G66820,AT1G05720,AT3G06180,AT1G52230,AT2G16530,AT2G25950,AT1G66890,AT2G26900,AT3G42630,AT5G40500,AT2G38820,AT1G32130,AT2G10370,AT2G15320,AT3G13080,AT4G39710,AT2G05710,AT1G26220,AT5G39080,AT5G11060,AT5G11070,AT3G32930,AT3G28340,AT5G37540,AT2G28630,AT2G41430,AT2G23600,AT4G37870,AT3G20680,AT1G35460,AT4G37290,AT5G35630,AT4G37680,AT5G24490,AT5G20935,AT5G24620,AT1G71880,AT1G52760,AT2G25210,AT2G30990,AT3G27170,AT1G67623,AT4G36390,AT4G36790,AT4G36600,AT4G37250,AT4G35770,AT2G22990,AT4G37260,AT2G22980,AT2G19450,AT5G23140,AT3G13430,AT1G80530,AT1G16680,AT5G22340,AT1G80310,AT5G19010,AT5G18850,AT3G14090,AT5G18640,AT5G18680,AT3G15290,AT4G33500,AT2G33860,AT5G17780,AT2G33830,AT2G02710,AT2G36240,AT4G32530,AT5G15770,AT4G32270,AT2G32640,AT2G47320,AT5G16560,AT5G15580,AT5G14620,AT3G24170,AT2G42190,AT5G35170,AT5G35220,AT2G10850,AT5G14180,AT1G55960,AT1G55840,AT3G15570,AT5G27720,AT1G32360,AT5G11670,AT5G27290,AT5G11740,AT5G26930,AT1G32310,AT1G69040,AT5G10860,AT1G49010,AT4G27800,AT5G26030,AT4G28080,AT1G78890,AT3G26280,AT1G78260,AT4G27410,AT4G27450,AT5G08590,AT5G24930,AT1G21500,AT4G27320,AT3G23080,AT3G26650,AT4G26520,AT1G06020,AT4G26560,AT4G26530,AT3G26740,AT3G17750,AT5G66570,AT1G26590,AT3G16940,AT1G01240,AT5G06550,AT5G65910,AT1G09570,AT5G66060,AT1G01320,AT4G25660,AT3G23160,AT3G19930,AT5G65380,AT1G19660,AT5G05200,AT5G64630,AT5G04490,AT1G09750,AT5G04590,AT4G24190,AT1G70000,AT5G64180,AT5G63920,AT4G23940,AT3G16220,AT5G04040,AT1G62290,AT3G14640,AT5G63780,AT5G63810,AT1G26800,AT1G08570,AT3G18080,AT5G03350,AT5G63380,AT1G08640,AT5G02810,AT5G02490,AT2G17330,AT3G15630,AT5G02160,AT3G15850,AT5G62360,AT5G60680,AT1G49750,AT3G62910,AT3G22430,AT3G22420,AT3G62580,AT1G61590,AT5G60210,AT1G01940,AT3G62860,AT3G62100,AT5G59140,AT5G59080,AT1G47290,AT5G59180,AT5G58770,AT3G02750,AT2G36800,AT3G61430,AT1G08380,AT1G76180,AT5G58380,AT3G60770,AT4G11590,AT5G58070,AT3G07890,AT3G08010,AT5G57815,AT3G08590,AT4G10920,AT3G21390,AT3G11800,AT2G18290,AT2G16600,AT3G10720,AT3G10740,AT3G59400,AT2G46550,AT2G15890,AT5G55120,AT4G05050,AT3G31980,AT3G10020,AT1G70820,AT3G08890,AT5G53500,AT3G55670,AT5G52580,AT2G06630,AT3G07310,AT5G52320,AT3G03320,AT3G54990,AT2G42390,AT5G51460,AT3G54810,AT1G17160

CTTATCCN	10	5	['8-12', '20-24']	290	ATMG00650,AT3G54050,AT3G01310,AT3G54220,AT1G31330,AT1G34220,AT5G50680,AT3G53500,AT3G01500,AT2G26530,AT2G26520,AT1G15290,AT3G01660,AT2G18670,AT3G52840,AT2G18700,AT2G41680,AT2G40700,AT1G32060,AT3G52070,AT3G07640,AT1G62750,AT5G49360,AT3G01180,AT5G49120,AT3G05130,AT1G75950,AT3G01090,AT3G51430,AT5G47910,AT1G28600,AT3G16320,AT4G25530,AT1G06410,AT1G28580,AT1G02305,AT3G50530,AT3G50610,AT3G04870,AT1G10740,AT1G01620,AT3G48580,AT1G14920,AT2G07140,AT1G07040,AT1G64720,AT4G16690,AT3G48140,AT4G16330,AT1G79500,AT1G21300,AT3G47340,AT2G24050,AT2G35500,AT1G56510,AT5G43930,AT1G30200,AT4G16480,AT1G55330,AT5G43830,AT3G47160,AT2G18170,AT1G56220,AT1G03130,AT1G71020,AT1G71180,AT4G14270,AT2G25870,AT1G29510,AT1G24764,AT3G45690,AT1G64330,AT3G45060,AT2G46780,AT1G66820,AT1G05720,AT3G06180,AT1G52230,AT2G16530,AT2G25950,AT1G66890,AT2G26900,AT3G42630,AT5G40500,AT2G38820,AT1G32130,AT2G10370,AT2G15320,AT3G13080,AT4G39710,AT2G05710,AT1G26220,AT5G39080,AT5G11060,AT5G11070,AT3G32930,AT3G28340,AT5G37540,AT2G28630,AT2G41430,AT2G23600,AT4G37870,AT3G20680,AT1G35460,AT4G37290,AT5G35630,AT4G37680,AT5G24490,AT5G20935,AT5G24620,AT1G71880,AT1G52760,AT2G25210,AT2G30990,AT3G27170,AT1G67623,AT4G36390,AT4G36790,AT4G36600,AT4G37250,AT4G35770,AT2G22990,AT4G37260,AT2G22980,AT2G19450,AT5G23140,AT3G13430,AT1G80530,AT1G16680,AT5G22340,AT1G80310,AT5G19010,AT5G18850,AT3G14090,AT5G18640,AT5G18680,AT3G15290,AT4G33500,AT2G33860,AT5G17780,AT2G33830,AT2G02710,AT2G36240,AT4G32530,AT5G15770,AT4G32270,AT2G32640,AT2G47320,AT5G16560,AT5G15580,AT5G14620,AT3G24170,AT2G42190,AT5G35170,AT5G35220,AT2G10850,AT5G14180,AT1G55960,AT1G55840,AT3G15570,AT5G27720,AT1G32360,AT5G11670,AT5G27290,AT5G11740,AT5G26930,AT1G32310,AT1G69040,AT5G10860,AT1G49010,AT4G27800,AT5G26030,AT4G28080,AT1G78890,AT3G26280,AT1G78260,AT4G27410,AT4G27450,AT5G08590,AT5G24930,AT1G21500,AT4G27320,AT3G23080,AT3G26650,AT4G26520,AT1G06020,AT4G26560,AT4G26530,AT3G26740,AT3G17750,AT5G66570,AT1G26590,AT3G16940,AT1G01240,AT5G06550,AT5G65910,AT1G09570,AT5G66060,AT1G01320,AT4G25660,AT3G23160,AT3G19930,AT5G65380,AT1G19660,AT5G05200,AT5G64630,AT5G04490,AT1G09750,AT5G04590,AT4G24190,AT1G70000,AT5G64180,AT5G63920,AT4G23940,AT3G16220,AT5G04040,AT1G62290,AT3G14640,AT5G63780,AT5G63810,AT1G26800,AT1G08570,AT3G18080,AT5G03350,AT5G63380,AT1G08640,AT5G02810,AT5G02490,AT2G17330,AT3G15630,AT5G02160,AT3G15850,AT5G62360,AT5G60680,AT1G49750,AT3G62910,AT3G22430,AT3G22420,AT3G62580,AT1G61590,AT5G60210,AT1G01940,AT3G62860,AT3G62100,AT5G59140,AT5G59080,AT1G47290,AT5G59180,AT5G58770,AT3G02750,AT2G36800,AT3G61430,AT1G08380,AT1G76180,AT5G58380,AT3G60770,AT4G11590,AT5G58070,AT3G07890,AT3G08010,AT5G57815,AT3G08590,AT4G10920,AT3G21390,AT3G11800,AT2G18290,AT2G16600,AT3G10720,AT3G10740,AT3G59400,AT2G46550,AT2G15890,AT5G55120,AT4G05050,AT3G31980,AT3G10020,AT1G70820,AT3G08890,AT5G53500,AT3G55670,AT5G52580,AT2G06630,AT3G07310,AT5G52320,AT3G03320,AT3G54990,AT2G42390,AT5G51460,AT3G54810,AT1G17160
GAAWTTGTGA	10	1	['0-4', '16-20']	40	AT4G29060,AT1G67360,AT5G26790,AT3G01660,AT1G14140,AT1G01940,AT4G00050,AT1G62480,AT2G38000,AT4G01020,AT1G12090,AT2G25180,AT2G02280,AT1G12990,AT1G02340,AT5G47800,AT2G25950,AT1G01320,AT2G18290,AT1G74430,AT5G23060,AT3G59060,AT4G08840,AT2G46570,AT2G44550,AT1G61380,AT1G05850,AT3G17930,AT1G72810,AT1G49500,AT3G07310,AT3G07390,AT5G27710,AT3G44250,AT5G51840,AT2G16500,AT5G01600,AT1G61140,AT3G15480,AT4G39950
GAAWTTGTGA	10	2	['0-4', '16-20']	40	AT4G29060,AT1G67360,AT5G26790,AT3G01660,AT1G14140,AT1G01940,AT4G00050,AT1G62480,AT2G38000,AT4G01020,AT1G12090,AT2G25180,AT2G02280,AT1G12990,AT1G02340,AT5G47800,AT2G25950,AT1G01320,AT2G18290,AT1G74430,AT5G23060,AT3G59060,AT4G08840,AT2G46570,AT2G44550,AT1G61380,AT1G05850,AT3G17930,AT1G72810,AT1G49500,AT3G07310,AT3G07390,AT5G27710,AT3G44250,AT5G51840,AT2G16500,AT5G01600,AT1G61140,AT3G15480,AT4G39950
GAAWTTGTGA	10	3	['0-4', '16-20']	40	AT4G29060,AT1G67360,AT5G26790,AT3G01660,AT1G14140,AT1G01940,AT4G00050,AT1G62480,AT2G38000,AT4G01020,AT1G12090,AT2G25180,AT2G02280,AT1G12990,AT1G02340,AT5G47800,AT2G25950,AT1G01320,AT2G18290,AT1G74430,AT5G23060,AT3G59060,AT4G08840,AT2G46570,AT2G44550,AT1G61380,AT1G05850,AT3G17930,AT1G72810,AT1G49500,AT3G07310,AT3G07390,AT5G27710,AT3G44250,AT5G51840,AT2G16500,AT5G01600,AT1G61140,AT3G15480,AT4G39950

GAAWTTGTGA	10	4	['0-4', '16-20']	40	AT4G29060,AT1G67360,AT5G26790,AT3G01660,AT1G14140,AT1G01940,AT4G00050,AT1G62480,AT2G38000,AT4G01020,AT1G12090,AT2G25180,AT2G02280,AT1G12990,AT1G02340,AT5G47800,AT2G25950,AT1G01320,AT2G18290,AT1G74430,AT5G23060,AT3G59060,AT4G08840,AT2G46570,AT2G44550,AT1G61380,AT1G05850,AT3G17930,AT1G72810,AT1G49500,AT3G07310,AT3G07390,AT5G27710,AT3G44250,AT5G51840,AT2G16500,AT5G01600,AT1G61140,AT3G15480,AT4G39950
GAAWTTGTGA	10	5	['16-20', '0-4']	40	AT4G29060,AT1G67360,AT5G26790,AT3G01660,AT1G14140,AT1G01940,AT4G00050,AT1G62480,AT2G38000,AT4G01020,AT1G12090,AT2G25180,AT2G02280,AT1G12990,AT1G02340,AT5G47800,AT2G25950,AT1G01320,AT2G18290,AT1G74430,AT5G23060,AT3G59060,AT4G08840,AT2G46570,AT2G44550,AT1G61380,AT1G05850,AT3G17930,AT1G72810,AT1G49500,AT3G07310,AT3G07390,AT5G27710,AT3G44250,AT5G51840,AT2G16500,AT5G01600,AT1G61140,AT3G15480,AT4G39950
GACCGACAA	10	1	['0-4', '4-8', '8-12', '12-16', '16-20']	14	AT5G66880,AT5G02650,AT5G57960,AT1G54520,AT3G24715,AT4G35800,AT3G15480,AT3G06510,AT1G69870,AT1G68820,AT2G21300,AT5G16715,AT2G27360,AT4G00270
GACCGACAA	10	2	['0-4', '4-8', '8-12', '12-16', '16-20']	14	AT5G66880,AT5G02650,AT5G57960,AT1G54520,AT3G24715,AT4G35800,AT3G15480,AT3G06510,AT1G69870,AT1G68820,AT2G21300,AT5G16715,AT2G27360,AT4G00270
GACCGACAA	10	3	['0-4', '4-8', '8-12', '12-16', '16-20']	14	AT5G66880,AT5G02650,AT5G57960,AT1G54520,AT3G24715,AT4G35800,AT3G15480,AT3G06510,AT1G69870,AT1G68820,AT2G21300,AT5G16715,AT2G27360,AT4G00270
GACCGACAA	10	4	['0-4', '4-8', '8-12', '12-16', '16-20']	14	AT5G66880,AT5G02650,AT5G57960,AT1G54520,AT3G24715,AT4G35800,AT3G15480,AT3G06510,AT1G69870,AT1G68820,AT2G21300,AT5G16715,AT2G27360,AT4G00270
GACCGACAA	10	5	['4-8', '8-12', '12-16', '16-20', '0-4']	14	AT5G66880,AT5G02650,AT5G57960,AT1G54520,AT3G24715,AT4G35800,AT3G15480,AT3G06510,AT1G69870,AT1G68820,AT2G21300,AT5G16715,AT2G27360,AT4G00270
GACCGACAT	10	1	['4-8', '8-12', '12-16', '16-20']	12	AT5G39940,AT1G73030,AT1G79500,AT3G12980,AT1G49270,AT2G17330,AT1G20440,AT3G20390,AT1G01430,AT1G47290,AT2G17840,AT1G32440
GACCGACAT	10	2	['4-8', '8-12', '16-20', '12-16']	12	AT5G39940,AT1G73030,AT1G79500,AT3G12980,AT1G49270,AT2G17330,AT1G20440,AT3G20390,AT1G01430,AT1G47290,AT2G17840,AT1G32440
GACCGACAT	10	3	['4-8', '8-12', '12-16', '16-20']	12	AT5G39940,AT1G73030,AT1G79500,AT3G12980,AT1G49270,AT2G17330,AT1G20440,AT3G20390,AT1G01430,AT1G47290,AT2G17840,AT1G32440
GACCGACAT	10	4	['4-8', '8-12', '12-16', '16-20']	12	AT5G39940,AT1G73030,AT1G79500,AT3G12980,AT1G49270,AT2G17330,AT1G20440,AT3G20390,AT1G01430,AT1G47290,AT2G17840,AT1G32440
GACCGACAT	10	5	['4-8', '8-12', '12-16', '16-20']	12	AT5G39940,AT1G73030,AT1G79500,AT3G12980,AT1G49270,AT2G17330,AT1G20440,AT3G20390,AT1G01430,AT1G47290,AT2G17840,AT1G32440
GACCGACTA	10	1	['0-4', '12-16', '20-24']	14	AT5G65310,AT5G27520,AT2G27050,AT4G33980,AT3G17130,AT3G22910,AT4G16480,AT1G55210,AT1G08570,AT3G04360,AT1G19920,AT1G61140,AT1G36940,AT5G61530
GACCGACTA	10	2	['0-4', '12-16', '20-24']	14	AT5G65310,AT5G27520,AT2G27050,AT4G33980,AT3G17130,AT3G22910,AT4G16480,AT1G55210,AT1G08570,AT3G04360,AT1G19920,AT1G61140,AT1G36940,AT5G61530
GACCGACTA	10	3	['0-4', '12-16', '20-24']	14	AT5G65310,AT5G27520,AT2G27050,AT4G33980,AT3G17130,AT3G22910,AT4G16480,AT1G55210,AT1G08570,AT3G04360,AT1G19920,AT1G61140,AT1G36940,AT5G61530
GACCGACTA	10	4	['0-4', '12-16', '20-24']	14	AT5G65310,AT5G27520,AT2G27050,AT4G33980,AT3G17130,AT3G22910,AT4G16480,AT1G55210,AT1G08570,AT3G04360,AT1G19920,AT1G61140,AT1G36940,AT5G61530
GACCGACTA	10	5	['0-4', '12-16', '20-24']	14	AT5G65310,AT5G27520,AT2G27050,AT4G33980,AT3G17130,AT3G22910,AT4G16480,AT1G55210,AT1G08570,AT3G04360,AT1G19920,AT1G61140,AT1G36940,AT5G61530

GACCGACTT	10	1	['0-4', '4-8', '8-12', '16-20', '20-24']	11	AT5G48540,AT2G34770,AT5G51010,AT1G59870,AT1G16150,AT3G27050,AT3G45260,AT5G46690,AT3G50610,AT4G33140,AT4G29190
GACCGACTT	10	2	['0-4', '4-8', '8-12', '16-20', '20-24']	11	AT5G48540,AT2G34770,AT5G51010,AT1G59870,AT1G16150,AT3G27050,AT3G45260,AT5G46690,AT3G50610,AT4G33140,AT4G29190
GACCGACTT	10	3	['0-4', '4-8', '8-12', '20-24', '16-20']	11	AT5G48540,AT2G34770,AT5G51010,AT1G59870,AT1G16150,AT3G27050,AT3G45260,AT5G46690,AT3G50610,AT4G33140,AT4G29190
GACCGACTT	10	4	['0-4', '4-8', '8-12', '16-20', '20-24']	11	AT5G48540,AT2G34770,AT5G51010,AT1G59870,AT1G16150,AT3G27050,AT3G45260,AT5G46690,AT3G50610,AT4G33140,AT4G29190
GACCGACTT	10	5	['0-4', '4-8', '8-12', '16-20', '20-24']	11	AT5G48540,AT2G34770,AT5G51010,AT1G59870,AT1G16150,AT3G27050,AT3G45260,AT5G46690,AT3G50610,AT4G33140,AT4G29190
GACCGTTN	10	1	['20-24']	155	AT5G49950,AT3G53190,AT2G23200,AT3G52720,AT2G47610,AT1G06680,AT3G52450,AT5G49030,AT2G45440,AT3G01120,AT2G47590,AT3G05180,AT5G48830,AT2G33250,AT1G75900,AT1G16240,AT2G33420,AT1G66970,AT1G18040,AT2G29560,AT1G06410,AT1G44446,AT1G22330,AT3G50810,AT3G50240,AT1G14810,AT1G15750,AT1G20693,AT1G75690,AT4G16950,AT1G75780,AT2G29670,AT4G15840,AT1G65190,AT1G71020,AT5G42760,AT1G29390,AT1G25275,AT3G45230,AT5G41600,AT5G04140,AT1G52200,AT5G41220,AT1G35150,AT5G40840,AT5G41140,AT2G31360,AT4G40040,AT5G39740,AT2G39480,AT1G68820,AT4G38420,AT3G52340,AT5G20070,AT4G37480,AT5G24610,AT2G25250,AT1G52870,AT4G35790,AT5G23760,AT5G23420,AT3G24010,AT3G13510,AT2G40400,AT2G36870,AT1G69295,AT5G16880,AT4G33490,AT5G17020,AT5G17440,AT4G33430,AT4G33480,AT3G28180,AT5G16260,AT2G45680,AT2G42700,AT5G17230,AT5G15230,AT2G32990,AT5G14310,AT4G30440,AT1G54830,AT5G11740,AT2G23880,AT1G48060,AT4G27800,AT4G28100,AT1G78260,AT3G18420,AT5G24930,AT1G53580,AT5G66340,AT1G10090,AT5G65630,AT5G06170,AT1G07440,AT4G24830,AT1G04710,AT4G24220,AT5G64630,AT4G23820,AT1G12960,AT1G70250,AT2G04390,AT5G03860,AT1G26760,AT3G18215,AT1G05850,AT5G63370,AT1G79730,AT5G02820,AT5G62540,AT1G23210,AT5G62130,AT1G14280,AT1G61140,AT1G76990,AT1G01700,AT3G02690,AT3G61770,AT3G61820,AT5G59180,AT3G61430,AT4G12010,AT3G60600,AT1G05000,AT1G25155,AT1G16000,AT4G11410,AT4G10840,AT4G09760,AT3G09630,AT3G59940,AT1G65060,AT4G09880,AT3G59350,AT3G11780,AT5G56030,AT4G05320,AT5G55220,AT1G74880,AT1G78010,AT5G54510,AT5G54680,AT4G03510,AT2G01290,AT3G55630,AT3G07470,AT3G09250,AT3G55390,AT2G01680,AT2G25620,AT4G00830,AT2G15040,AT3G04880

GACCGTTN	10	3	['20-24']	155	AT5G49950,AT3G53190,AT2G23200,AT3G52720,AT2G47610,AT1G06680,AT3G52450,AT5G49030,AT2G45440,AT3G01120,AT2G47590,AT3G05180,AT5G48830,AT2G33250,AT1G75900,AT1G16240,AT2G33420,AT1G66970,AT1G18040,AT2G29560,AT1G06410,AT1G44446,AT1G22330,AT3G50810,AT3G50240,AT1G14810,AT1G15750,AT1G20693,AT1G75690,AT4G16950,AT1G75780,AT2G29670,AT4G15840,AT1G65190,AT1G71020,AT5G42760,AT1G29390,AT1G25275,AT3G45230,AT5G41600,AT5G04140,AT1G52200,AT5G41220,AT1G35150,AT5G40840,AT5G41140,AT2G31360,AT4G40040,AT5G39740,AT2G39480,AT1G68820,AT4G38420,AT3G52340,AT5G20070,AT4G37480,AT5G24610,AT2G25250,AT1G52870,AT4G35790,AT5G23760,AT5G23420,AT3G24010,AT3G13510,AT2G40400,AT2G36870,AT1G69295,AT5G16880,AT4G33490,AT5G17020,AT5G17440,AT4G33430,AT4G33480,AT3G28180,AT5G16260,AT2G45680,AT2G42700,AT5G17230,AT5G15230,AT2G32990,AT5G14310,AT4G30440,AT1G54830,AT5G11740,AT2G23880,AT1G48060,AT4G27800,AT4G28100,AT1G78260,AT3G18420,AT5G24930,AT1G53580,AT5G66340,AT1G10090,AT5G65630,AT5G06170,AT1G07440,AT4G24830,AT1G04710,AT4G24220,AT5G64630,AT4G23820,AT1G12960,AT1G70250,AT2G04390,AT5G03860,AT1G26760,AT3G18215,AT1G05850,AT5G63370,AT1G79730,AT5G02820,AT5G62540,AT1G23210,AT5G62130,AT1G14280,AT1G61140,AT1G76990,AT1G01700,AT3G02690,AT3G61770,AT3G61820,AT5G59180,AT3G61430,AT4G12010,AT3G60600,AT1G05000,AT1G25155,AT1G16000,AT4G11410,AT4G10840,AT4G09760,AT3G09630,AT3G59940,AT1G65060,AT4G09880,AT3G59350,AT3G11780,AT5G56030,AT4G05320,AT5G55220,AT1G74880,AT1G78010,AT5G54510,AT5G54680,AT4G03510,AT2G01290,AT3G55630,AT3G07470,AT3G09250,AT3G55390,AT2G01680,AT2G25620,AT4G00830,AT2G15040,AT3G04880
GACCGTTN	10	4	['20-24']	155	AT5G49950,AT3G53190,AT2G23200,AT3G52720,AT2G47610,AT1G06680,AT3G52450,AT5G49030,AT2G45440,AT3G01120,AT2G47590,AT3G05180,AT5G48830,AT2G33250,AT1G75900,AT1G16240,AT2G33420,AT1G66970,AT1G18040,AT2G29560,AT1G06410,AT1G44446,AT1G22330,AT3G50810,AT3G50240,AT1G14810,AT1G15750,AT1G20693,AT1G75690,AT4G16950,AT1G75780,AT2G29670,AT4G15840,AT1G65190,AT1G71020,AT5G42760,AT1G29390,AT1G25275,AT3G45230,AT5G41600,AT5G04140,AT1G52200,AT5G41220,AT1G35150,AT5G40840,AT5G41140,AT2G31360,AT4G40040,AT5G39740,AT2G39480,AT1G68820,AT4G38420,AT3G52340,AT5G20070,AT4G37480,AT5G24610,AT2G25250,AT1G52870,AT4G35790,AT5G23760,AT5G23420,AT3G24010,AT3G13510,AT2G40400,AT2G36870,AT1G69295,AT5G16880,AT4G33490,AT5G17020,AT5G17440,AT4G33430,AT4G33480,AT3G28180,AT5G16260,AT2G45680,AT2G42700,AT5G17230,AT5G15230,AT2G32990,AT5G14310,AT4G30440,AT1G54830,AT5G11740,AT2G23880,AT1G48060,AT4G27800,AT4G28100,AT1G78260,AT3G18420,AT5G24930,AT1G53580,AT5G66340,AT1G10090,AT5G65630,AT5G06170,AT1G07440,AT4G24830,AT1G04710,AT4G24220,AT5G64630,AT4G23820,AT1G12960,AT1G70250,AT2G04390,AT5G03860,AT1G26760,AT3G18215,AT1G05850,AT5G63370,AT1G79730,AT5G02820,AT5G62540,AT1G23210,AT5G62130,AT1G14280,AT1G61140,AT1G76990,AT1G01700,AT3G02690,AT3G61770,AT3G61820,AT5G59180,AT3G61430,AT4G12010,AT3G60600,AT1G05000,AT1G25155,AT1G16000,AT4G11410,AT4G10840,AT4G09760,AT3G09630,AT3G59940,AT1G65060,AT4G09880,AT3G59350,AT3G11780,AT5G56030,AT4G05320,AT5G55220,AT1G74880,AT1G78010,AT5G54510,AT5G54680,AT4G03510,AT2G01290,AT3G55630,AT3G07470,AT3G09250,AT3G55390,AT2G01680,AT2G25620,AT4G00830,AT2G15040,AT3G04880

GACCGTTN	10	5	['20-24']	155	AT5G49950,AT3G53190,AT2G23200,AT3G52720,AT2G47610,AT1G06680,AT3G52450,AT5G49030,AT2G45440,AT3G01120,AT2G47590,AT3G05180,AT5G48830,AT2G33250,AT1G75900,AT1G16240,AT2G33420,AT1G66970,AT1G18040,AT2G29560,AT1G06410,AT1G44446,AT1G22330,AT3G50810,AT3G50240,AT1G14810,AT1G15750,AT1G20693,AT1G75690,AT4G16950,AT1G75780,AT2G29670,AT4G15840,AT1G65190,AT1G71020,AT5G42760,AT1G29390,AT1G25275,AT3G45230,AT5G41600,AT5G04140,AT1G52200,AT5G41220,AT1G35150,AT5G40840,AT5G41140,AT2G31360,AT4G40040,AT5G39740,AT2G39480,AT1G68820,AT4G38420,AT3G52340,AT5G20070,AT4G37480,AT5G24610,AT2G25250,AT1G52870,AT4G35790,AT5G23760,AT5G23420,AT3G24010,AT3G13510,AT2G40400,AT2G36870,AT1G69295,AT5G16880,AT4G33490,AT5G17020,AT5G17440,AT4G33430,AT4G33480,AT3G28180,AT5G16260,AT2G45680,AT2G42700,AT5G17230,AT5G15230,AT2G32990,AT5G14310,AT4G30440,AT1G54830,AT5G11740,AT2G23880,AT1G48060,AT4G27800,AT4G28100,AT1G78260,AT3G18420,AT5G24930,AT1G53580,AT5G66340,AT1G10090,AT5G65630,AT5G06170,AT1G07440,AT4G24830,AT1G04710,AT4G24220,AT5G64630,AT4G23820,AT1G12960,AT1G70250,AT2G04390,AT5G03860,AT1G26760,AT3G18215,AT1G05850,AT5G63370,AT1G79730,AT5G02820,AT5G62540,AT1G23210,AT5G62130,AT1G14280,AT1G61140,AT1G76990,AT1G01700,AT3G02690,AT3G61770,AT3G61820,AT5G59180,AT3G61430,AT4G12010,AT3G60600,AT1G05000,AT1G25155,AT1G16000,AT4G11410,AT4G10840,AT4G09760,AT3G09630,AT3G59940,AT1G65060,AT4G09880,AT3G59350,AT3G11780,AT5G56030,AT4G05320,AT5G55220,AT1G74880,AT1G78010,AT5G54510,AT5G54680,AT4G03510,AT2G01290,AT3G55630,AT3G07470,AT3G09250,AT3G55390,AT2G01680,AT2G25620,AT4G00830,AT2G15040,AT3G04880
GACGTGGA	10	1	['4-8', '20-24']	46	AT4G29040,AT3G16690,AT3G63190,AT1G75080,AT1G50020,AT3G62100,AT1G06680,AT5G58770,AT1G21880,AT3G04460,AT2G34090,AT3G08010,AT4G37480,AT1G70660,AT5G47870,AT2G41410,AT3G17000,AT1G44000,AT5G05950,AT3G48690,AT4G34490,AT1G14910,AT3G13510,AT2G46490,AT1G73990,AT1G69210,AT5G03905,AT1G51790,AT3G03180,AT5G64030,AT2G28200,AT3G27270,AT2G17845,AT4G03110,AT5G02810,AT5G02820,AT1G29390,AT1G79790,AT3G21690,AT1G73120,AT3G01860,AT4G31050,AT5G62200,AT1G23205,AT5G35180,AT1G76730
GACGTGGA	10	2	['4-8', '20-24']	46	AT4G29040,AT3G16690,AT3G63190,AT1G75080,AT1G50020,AT3G62100,AT1G06680,AT5G58770,AT1G21880,AT3G04460,AT2G34090,AT3G08010,AT4G37480,AT1G70660,AT5G47870,AT2G41410,AT3G17000,AT1G44000,AT5G05950,AT3G48690,AT4G34490,AT1G14910,AT3G13510,AT2G46490,AT1G73990,AT1G69210,AT5G03905,AT1G51790,AT3G03180,AT5G64030,AT2G28200,AT3G27270,AT2G17845,AT4G03110,AT5G02810,AT5G02820,AT1G29390,AT1G79790,AT3G21690,AT1G73120,AT3G01860,AT4G31050,AT5G62200,AT1G23205,AT5G35180,AT1G76730
GACGTGGA	10	3	['4-8', '20-24']	46	AT4G29040,AT3G16690,AT3G63190,AT1G75080,AT1G50020,AT3G62100,AT1G06680,AT5G58770,AT1G21880,AT3G04460,AT2G34090,AT3G08010,AT4G37480,AT1G70660,AT5G47870,AT2G41410,AT3G17000,AT1G44000,AT5G05950,AT3G48690,AT4G34490,AT1G14910,AT3G13510,AT2G46490,AT1G73990,AT1G69210,AT5G03905,AT1G51790,AT3G03180,AT5G64030,AT2G28200,AT3G27270,AT2G17845,AT4G03110,AT5G02810,AT5G02820,AT1G29390,AT1G79790,AT3G21690,AT1G73120,AT3G01860,AT4G31050,AT5G62200,AT1G23205,AT5G35180,AT1G76730
GACGTGGA	10	4	['4-8', '20-24']	46	AT4G29040,AT3G16690,AT3G63190,AT1G75080,AT1G50020,AT3G62100,AT1G06680,AT5G58770,AT1G21880,AT3G04460,AT2G34090,AT3G08010,AT4G37480,AT1G70660,AT5G47870,AT2G41410,AT3G17000,AT1G44000,AT5G05950,AT3G48690,AT4G34490,AT1G14910,AT3G13510,AT2G46490,AT1G73990,AT1G69210,AT5G03905,AT1G51790,AT3G03180,AT5G64030,AT2G28200,AT3G27270,AT2G17845,AT4G03110,AT5G02810,AT5G02820,AT1G29390,AT1G79790,AT3G21690,AT1G73120,AT3G01860,AT4G31050,AT5G62200,AT1G23205,AT5G35180,AT1G76730
GACGTGGA	10	5	['4-8', '20-24']	46	AT4G29040,AT3G16690,AT3G63190,AT1G75080,AT1G50020,AT3G62100,AT1G06680,AT5G58770,AT1G21880,AT3G04460,AT2G34090,AT3G08010,AT4G37480,AT1G70660,AT5G47870,AT2G41410,AT3G17000,AT1G44000,AT5G05950,AT3G48690,AT4G34490,AT1G14910,AT3G13510,AT2G46490,AT1G73990,AT1G69210,AT5G03905,AT1G51790,AT3G03180,AT5G64030,AT2G28200,AT3G27270,AT2G17845,AT4G03110,AT5G02810,AT5G02820,AT1G29390,AT1G79790,AT3G21690,AT1G73120,AT3G01860,AT4G31050,AT5G62200,AT1G23205,AT5G35180,AT1G76730

GACGTGGC	10	1	['0-4', '16-20']	77	AT1G30800,AT2G37240,AT5G51010,AT2G37450,AT1G53670,AT1G22750,AT5G19860,AT1G14150,AT2G34250,AT1G55510,AT5G49540,AT3G52950,AT3G63520,AT1G18330,AT3G07680,AT4G27410,AT4G13010,AT5G58770,AT5G24970,AT1G76030,AT2G31790,AT5G24810,AT5G36170,AT1G43770,AT3G04460,AT3G51240,AT1G76150,AT2G27710,AT5G19940,AT4G37480,AT1G27480,AT5G06870,AT3G50240,AT3G22970,AT3G49590,AT4G15560,AT3G22890,AT1G07140,AT5G65990,AT1G10070,AT3G15360,AT1G17460,AT5G65430,AT3G10720,AT1G20696,AT2G36870,AT3G47450,AT5G18850,AT2G40080,AT3G19100,AT4G33980,AT4G17140,AT5G17990,AT1G78020,AT5G54770,AT3G46780,AT5G17660,AT1G69830,AT5G59570,AT3G56490,AT4G03600,AT5G15860,AT2G41760,AT3G56260,AT2G03340,AT5G15510,AT3G01860,AT3G44950,AT4G19830,AT3G07470,AT1G05570,AT5G41050,AT5G13650,AT5G61380,AT3G54500,AT1G02020,AT1G32130
GACGTGGC	10	2	['0-4', '16-20']	77	AT1G30800,AT2G37240,AT5G51010,AT2G37450,AT1G53670,AT1G22750,AT5G19860,AT1G14150,AT2G34250,AT1G55510,AT5G49540,AT3G52950,AT3G63520,AT1G18330,AT3G07680,AT4G27410,AT4G13010,AT5G58770,AT5G24970,AT1G76030,AT2G31790,AT5G24810,AT5G36170,AT1G43770,AT3G04460,AT3G51240,AT1G76150,AT2G27710,AT5G19940,AT4G37480,AT1G27480,AT5G06870,AT3G50240,AT3G22970,AT3G49590,AT4G15560,AT3G22890,AT1G07140,AT5G65990,AT1G10070,AT3G15360,AT1G17460,AT5G65430,AT3G10720,AT1G20696,AT2G36870,AT3G47450,AT5G18850,AT2G40080,AT3G19100,AT4G33980,AT4G17140,AT5G17990,AT1G78020,AT5G54770,AT3G46780,AT5G17660,AT1G69830,AT5G59570,AT3G56490,AT4G03600,AT5G15860,AT2G41760,AT3G56260,AT2G03340,AT5G15510,AT3G01860,AT3G44950,AT4G19830,AT3G07470,AT1G05570,AT5G41050,AT5G13650,AT5G61380,AT3G54500,AT1G02020,AT1G32130
GACGTGGC	10	3	['0-4', '16-20']	77	AT1G30800,AT2G37240,AT5G51010,AT2G37450,AT1G53670,AT1G22750,AT5G19860,AT1G14150,AT2G34250,AT1G55510,AT5G49540,AT3G52950,AT3G63520,AT1G18330,AT3G07680,AT4G27410,AT4G13010,AT5G58770,AT5G24970,AT1G76030,AT2G31790,AT5G24810,AT5G36170,AT1G43770,AT3G04460,AT3G51240,AT1G76150,AT2G27710,AT5G19940,AT4G37480,AT1G27480,AT5G06870,AT3G50240,AT3G22970,AT3G49590,AT4G15560,AT3G22890,AT1G07140,AT5G65990,AT1G10070,AT3G15360,AT1G17460,AT5G65430,AT3G10720,AT1G20696,AT2G36870,AT3G47450,AT5G18850,AT2G40080,AT3G19100,AT4G33980,AT4G17140,AT5G17990,AT1G78020,AT5G54770,AT3G46780,AT5G17660,AT1G69830,AT5G59570,AT3G56490,AT4G03600,AT5G15860,AT2G41760,AT3G56260,AT2G03340,AT5G15510,AT3G01860,AT3G44950,AT4G19830,AT3G07470,AT1G05570,AT5G41050,AT5G13650,AT5G61380,AT3G54500,AT1G02020,AT1G32130
GACGTGGC	10	4	['0-4', '16-20']	77	AT1G30800,AT2G37240,AT5G51010,AT2G37450,AT1G53670,AT1G22750,AT5G19860,AT1G14150,AT2G34250,AT1G55510,AT5G49540,AT3G52950,AT3G63520,AT1G18330,AT3G07680,AT4G27410,AT4G13010,AT5G58770,AT5G24970,AT1G76030,AT2G31790,AT5G24810,AT5G36170,AT1G43770,AT3G04460,AT3G51240,AT1G76150,AT2G27710,AT5G19940,AT4G37480,AT1G27480,AT5G06870,AT3G50240,AT3G22970,AT3G49590,AT4G15560,AT3G22890,AT1G07140,AT5G65990,AT1G10070,AT3G15360,AT1G17460,AT5G65430,AT3G10720,AT1G20696,AT2G36870,AT3G47450,AT5G18850,AT2G40080,AT3G19100,AT4G33980,AT4G17140,AT5G17990,AT1G78020,AT5G54770,AT3G46780,AT5G17660,AT1G69830,AT5G59570,AT3G56490,AT4G03600,AT5G15860,AT2G41760,AT3G56260,AT2G03340,AT5G15510,AT3G01860,AT3G44950,AT4G19830,AT3G07470,AT1G05570,AT5G41050,AT5G13650,AT5G61380,AT3G54500,AT1G02020,AT1G32130
GACGTGGC	10	5	['16-20', '0-4']	77	AT1G30800,AT2G37240,AT5G51010,AT2G37450,AT1G53670,AT1G22750,AT5G19860,AT1G14150,AT2G34250,AT1G55510,AT5G49540,AT3G52950,AT3G63520,AT1G18330,AT3G07680,AT4G27410,AT4G13010,AT5G58770,AT5G24970,AT1G76030,AT2G31790,AT5G24810,AT5G36170,AT1G43770,AT3G04460,AT3G51240,AT1G76150,AT2G27710,AT5G19940,AT4G37480,AT1G27480,AT5G06870,AT3G50240,AT3G22970,AT3G49590,AT4G15560,AT3G22890,AT1G07140,AT5G65990,AT1G10070,AT3G15360,AT1G17460,AT5G65430,AT3G10720,AT1G20696,AT2G36870,AT3G47450,AT5G18850,AT2G40080,AT3G19100,AT4G33980,AT4G17140,AT5G17990,AT1G78020,AT5G54770,AT3G46780,AT5G17660,AT1G69830,AT5G59570,AT3G56490,AT4G03600,AT5G15860,AT2G41760,AT3G56260,AT2G03340,AT5G15510,AT3G01860,AT3G44950,AT4G19830,AT3G07470,AT1G05570,AT5G41050,AT5G13650,AT5G61380,AT3G54500,AT1G02020,AT1G32130

GACGTGTA	10	1	['8-12', '12-16', '16-20']	48	AT2G05070,AT5G26790,AT2G34430,AT3G28270,AT1G32060,AT1G04170,AT3G12710,AT1G62780,AT2G39080,AT1G66970,AT3G03710,AT4G26700,AT3G13790,AT3G16910,AT2G20260,AT5G23870,AT3G17000,AT5G23050,AT5G05520,AT5G19260,AT3G47960,AT5G56030,AT2G36885,AT5G55530,AT2G37130,AT4G23940,AT4G07425,AT1G58110,AT1G12800,AT2G21970,AT2G44550,AT1G20190,AT5G64170,AT1G23010,AT1G74750,AT4G15730,AT3G57190,AT5G63135,AT5G03510,AT1G65210,AT4G30610,AT3G15630,AT1G49250,AT1G79040,AT5G01600,AT3G14270,AT5G51110,AT5G12250
GACGTGTA	10	2	['8-12', '16-20', '12-16']	48	AT2G05070,AT5G26790,AT2G34430,AT3G28270,AT1G32060,AT1G04170,AT3G12710,AT1G62780,AT2G39080,AT1G66970,AT3G03710,AT4G26700,AT3G13790,AT3G16910,AT2G20260,AT5G23870,AT3G17000,AT5G23050,AT5G05520,AT5G19260,AT3G47960,AT5G56030,AT2G36885,AT5G55530,AT2G37130,AT4G23940,AT4G07425,AT1G58110,AT1G12800,AT2G21970,AT2G44550,AT1G20190,AT5G64170,AT1G23010,AT1G74750,AT4G15730,AT3G57190,AT5G63135,AT5G03510,AT1G65210,AT4G30610,AT3G15630,AT1G49250,AT1G79040,AT5G01600,AT3G14270,AT5G51110,AT5G12250
GACGTGTA	10	3	['8-12', '12-16', '16-20']	48	AT2G05070,AT5G26790,AT2G34430,AT3G28270,AT1G32060,AT1G04170,AT3G12710,AT1G62780,AT2G39080,AT1G66970,AT3G03710,AT4G26700,AT3G13790,AT3G16910,AT2G20260,AT5G23870,AT3G17000,AT5G23050,AT5G05520,AT5G19260,AT3G47960,AT5G56030,AT2G36885,AT5G55530,AT2G37130,AT4G23940,AT4G07425,AT1G58110,AT1G12800,AT2G21970,AT2G44550,AT1G20190,AT5G64170,AT1G23010,AT1G74750,AT4G15730,AT3G57190,AT5G63135,AT5G03510,AT1G65210,AT4G30610,AT3G15630,AT1G49250,AT1G79040,AT5G01600,AT3G14270,AT5G51110,AT5G12250
GACGTGTA	10	4	['8-12', '12-16', '16-20']	48	AT2G05070,AT5G26790,AT2G34430,AT3G28270,AT1G32060,AT1G04170,AT3G12710,AT1G62780,AT2G39080,AT1G66970,AT3G03710,AT4G26700,AT3G13790,AT3G16910,AT2G20260,AT5G23870,AT3G17000,AT5G23050,AT5G05520,AT5G19260,AT3G47960,AT5G56030,AT2G36885,AT5G55530,AT2G37130,AT4G23940,AT4G07425,AT1G58110,AT1G12800,AT2G21970,AT2G44550,AT1G20190,AT5G64170,AT1G23010,AT1G74750,AT4G15730,AT3G57190,AT5G63135,AT5G03510,AT1G65210,AT4G30610,AT3G15630,AT1G49250,AT1G79040,AT5G01600,AT3G14270,AT5G51110,AT5G12250
GACGTGTA	10	5	['8-12', '12-16', '16-20']	48	AT2G05070,AT5G26790,AT2G34430,AT3G28270,AT1G32060,AT1G04170,AT3G12710,AT1G62780,AT2G39080,AT1G66970,AT3G03710,AT4G26700,AT3G13790,AT3G16910,AT2G20260,AT5G23870,AT3G17000,AT5G23050,AT5G05520,AT5G19260,AT3G47960,AT5G56030,AT2G36885,AT5G55530,AT2G37130,AT4G23940,AT4G07425,AT1G58110,AT1G12800,AT2G21970,AT2G44550,AT1G20190,AT5G64170,AT1G23010,AT1G74750,AT4G15730,AT3G57190,AT5G63135,AT5G03510,AT1G65210,AT4G30610,AT3G15630,AT1G49250,AT1G79040,AT5G01600,AT3G14270,AT5G51110,AT5G12250
GACGTGTC	10	1	['0-4', '4-8']	45	ATMG00650,AT4G28740,AT1G49780,AT4G00960,AT2G03890,AT4G00490,AT3G28270,AT1G68830,AT1G62750,AT5G36870,AT3G18850,AT2G45170,AT4G38460,AT1G23540,AT2G38130,AT5G48100,AT3G22200,AT1G22070,AT2G18220,AT3G49680,AT4G25660,AT2G23670,AT5G65110,AT5G56240,AT3G59052,AT1G80530,AT4G23820,AT1G54270,AT1G62290,AT5G18340,AT3G06410,AT1G08720,AT5G03190,AT2G19790,AT2G19860,AT1G18740,AT5G02150,AT4G19185,AT3G27690,AT1G53320,AT5G41150,AT4G19420,AT5G40660,AT5G51390,AT4G01130
GACGTGTC	10	2	['0-4', '4-8']	45	ATMG00650,AT4G28740,AT1G49780,AT4G00960,AT2G03890,AT4G00490,AT3G28270,AT1G68830,AT1G62750,AT5G36870,AT3G18850,AT2G45170,AT4G38460,AT1G23540,AT2G38130,AT5G48100,AT3G22200,AT1G22070,AT2G18220,AT3G49680,AT4G25660,AT2G23670,AT5G65110,AT5G56240,AT3G59052,AT1G80530,AT4G23820,AT1G54270,AT1G62290,AT5G18340,AT3G06410,AT1G08720,AT5G03190,AT2G19790,AT2G19860,AT1G18740,AT5G02150,AT4G19185,AT3G27690,AT1G53320,AT5G41150,AT4G19420,AT5G40660,AT5G51390,AT4G01130
GACGTGTC	10	3	['0-4', '4-8']	45	ATMG00650,AT4G28740,AT1G49780,AT4G00960,AT2G03890,AT4G00490,AT3G28270,AT1G68830,AT1G62750,AT5G36870,AT3G18850,AT2G45170,AT4G38460,AT1G23540,AT2G38130,AT5G48100,AT3G22200,AT1G22070,AT2G18220,AT3G49680,AT4G25660,AT2G23670,AT5G65110,AT5G56240,AT3G59052,AT1G80530,AT4G23820,AT1G54270,AT1G62290,AT5G18340,AT3G06410,AT1G08720,AT5G03190,AT2G19790,AT2G19860,AT1G18740,AT5G02150,AT4G19185,AT3G27690,AT1G53320,AT5G41150,AT4G19420,AT5G40660,AT5G51390,AT4G01130

GACGTGTC	10	4	['0-4', '4-8']	45	ATMG00650,AT4G28740,AT1G49780,AT4G00960,AT2G03890,AT4G00490,AT3G28270,AT1G68830,AT1G62750,AT5G36870,AT3G18850,AT2G45170,AT4G38460,AT1G23540,AT2G38130,AT5G48100,AT3G22200,AT1G22070,AT2G18220,AT3G49680,AT4G25660,AT2G23670,AT5G65110,AT5G56240,AT3G59052,AT1G80530,AT4G23820,AT1G54270,AT1G62290,AT5G18340,AT3G06410,AT1G08720,AT5G03190,AT2G19790,AT2G19860,AT1G18740,AT5G02150,AT4G19185,AT3G27690,AT1G53320,AT5G41150,AT4G19420,AT5G40660,AT5G51390,AT4G01130
GACGTGTC	10	5	['4-8', '0-4']	45	ATMG00650,AT4G28740,AT1G49780,AT4G00960,AT2G03890,AT4G00490,AT3G28270,AT1G68830,AT1G62750,AT5G36870,AT3G18850,AT2G45170,AT4G38460,AT1G23540,AT2G38130,AT5G48100,AT3G22200,AT1G22070,AT2G18220,AT3G49680,AT4G25660,AT2G23670,AT5G65110,AT5G56240,AT3G59052,AT1G80530,AT4G23820,AT1G54270,AT1G62290,AT5G18340,AT3G06410,AT1G08720,AT5G03190,AT2G19790,AT2G19860,AT1G18740,AT5G02150,AT4G19185,AT3G27690,AT1G53320,AT5G41150,AT4G19420,AT5G40660,AT5G51390,AT4G01130
GAGAGAGAGAGAGAGA	10	1	['12-16', '4-8']	46	AT1G74160,AT2G18960,AT4G28395,AT1G61520,AT4G00130,AT5G11270,AT3G07650,AT1G78260,AT4G38470,AT5G09840,AT2G32560,AT3G03710,AT3G61030,AT5G58140,AT1G28600,AT2G29560,AT2G01940,AT5G28410,AT5G56730,AT4G36540,AT3G59020,AT3G56880,AT3G13510,AT4G17640,AT1G80030,AT4G34220,AT1G34440,AT1G56090,AT2G46490,AT2G19270,AT2G36870,AT4G17650,AT5G44800,AT5G55480,AT1G69210,AT1G69780,AT5G03470,AT5G03270,AT2G35605,AT4G31820,AT3G23690,AT2G32100,AT5G15160,AT3G55960,AT5G04170,AT4G19340
GAGAGAGAGAGAGAGA	10	2	['12-16']	46	AT1G74160,AT2G18960,AT4G28395,AT1G61520,AT4G00130,AT5G11270,AT3G07650,AT1G78260,AT4G38470,AT5G09840,AT2G32560,AT3G03710,AT3G61030,AT5G58140,AT1G28600,AT2G29560,AT2G01940,AT5G28410,AT5G56730,AT4G36540,AT3G59020,AT3G56880,AT3G13510,AT4G17640,AT1G80030,AT4G34220,AT1G34440,AT1G56090,AT2G46490,AT2G19270,AT2G36870,AT4G17650,AT5G44800,AT5G55480,AT1G69210,AT1G69780,AT5G03470,AT5G03270,AT2G35605,AT4G31820,AT3G23690,AT2G32100,AT5G15160,AT3G55960,AT5G04170,AT4G19340
GAGAGAGAGAGAGAGA	10	3	['12-16', '4-8']	46	AT1G74160,AT2G18960,AT4G28395,AT1G61520,AT4G00130,AT5G11270,AT3G07650,AT1G78260,AT4G38470,AT5G09840,AT2G32560,AT3G03710,AT3G61030,AT5G58140,AT1G28600,AT2G29560,AT2G01940,AT5G28410,AT5G56730,AT4G36540,AT3G59020,AT3G56880,AT3G13510,AT4G17640,AT1G80030,AT4G34220,AT1G34440,AT1G56090,AT2G46490,AT2G19270,AT2G36870,AT4G17650,AT5G44800,AT5G55480,AT1G69210,AT1G69780,AT5G03470,AT5G03270,AT2G35605,AT4G31820,AT3G23690,AT2G32100,AT5G15160,AT3G55960,AT5G04170,AT4G19340
GAGAGAGAGAGAGAGA	10	4	['12-16']	46	AT1G74160,AT2G18960,AT4G28395,AT1G61520,AT4G00130,AT5G11270,AT3G07650,AT1G78260,AT4G38470,AT5G09840,AT2G32560,AT3G03710,AT3G61030,AT5G58140,AT1G28600,AT2G29560,AT2G01940,AT5G28410,AT5G56730,AT4G36540,AT3G59020,AT3G56880,AT3G13510,AT4G17640,AT1G80030,AT4G34220,AT1G34440,AT1G56090,AT2G46490,AT2G19270,AT2G36870,AT4G17650,AT5G44800,AT5G55480,AT1G69210,AT1G69780,AT5G03470,AT5G03270,AT2G35605,AT4G31820,AT3G23690,AT2G32100,AT5G15160,AT3G55960,AT5G04170,AT4G19340
GAGAGAGAGAGAGAGA	10	5	['12-16', '4-8']	46	AT1G74160,AT2G18960,AT4G28395,AT1G61520,AT4G00130,AT5G11270,AT3G07650,AT1G78260,AT4G38470,AT5G09840,AT2G32560,AT3G03710,AT3G61030,AT5G58140,AT1G28600,AT2G29560,AT2G01940,AT5G28410,AT5G56730,AT4G36540,AT3G59020,AT3G56880,AT3G13510,AT4G17640,AT1G80030,AT4G34220,AT1G34440,AT1G56090,AT2G46490,AT2G19270,AT2G36870,AT4G17650,AT5G44800,AT5G55480,AT1G69210,AT1G69780,AT5G03470,AT5G03270,AT2G35605,AT4G31820,AT3G23690,AT2G32100,AT5G15160,AT3G55960,AT5G04170,AT4G19340
GAGAGAGAGAGAGAGAGA	10	1	['0-4', '12-16', '16-20', '20-24']	22	AT1G74160,AT2G18960,AT1G61520,AT4G38470,AT2G32560,AT3G03710,AT5G58140,AT2G29560,AT5G56730,AT4G36540,AT3G59020,AT1G56090,AT1G34440,AT5G55480,AT4G17650,AT5G44800,AT1G69210,AT4G31820,AT3G23690,AT5G15160,AT3G55960,AT5G04170
GAGAGAGAGAGAGAGAGA	10	2	['0-4', '12-16', '16-20', '20-24']	22	AT1G74160,AT2G18960,AT1G61520,AT4G38470,AT2G32560,AT3G03710,AT5G58140,AT2G29560,AT5G56730,AT4G36540,AT3G59020,AT1G56090,AT1G34440,AT5G55480,AT4G17650,AT5G44800,AT1G69210,AT4G31820,AT3G23690,AT5G15160,AT3G55960,AT5G04170
GAGAGAGAGAGAGAGAGA	10	3	['0-4', '12-16', '16-20', '20-24']	22	AT1G74160,AT2G18960,AT1G61520,AT4G38470,AT2G32560,AT3G03710,AT5G58140,AT2G29560,AT5G56730,AT4G36540,AT3G59020,AT1G56090,AT1G34440,AT5G55480,AT4G17650,AT5G44800,AT1G69210,AT4G31820,AT3G23690,AT5G15160,AT3G55960,AT5G04170

GAGAGAGAGA GAGAGAGA	10	4	['0-4', '12-16', '16-20', '20-24']	22	AT1G74160,AT2G18960,AT1G61520,AT4G38470,AT2G32560,AT3G03710,AT5G58140,AT2G29560,AT5G56730,AT4G36540,AT3G59020,AT1G56090,AT1G34440,AT5G55480,AT4G17650,AT5G44800,AT1G69210,AT4G31820,AT3G23690,AT5G15160,AT3G55960,AT5G04170
GAGAGAGAGA GAGAGAGA	10	5	['12-16', '16-20', '20-24', '0-4']	22	AT1G74160,AT2G18960,AT1G61520,AT4G38470,AT2G32560,AT3G03710,AT5G58140,AT2G29560,AT5G56730,AT4G36540,AT3G59020,AT1G56090,AT1G34440,AT5G55480,AT4G17650,AT5G44800,AT1G69210,AT4G31820,AT3G23690,AT5G15160,AT3G55960,AT5G04170
GATAAGR	10	1	['4-8']	699	ATMG00650,ATCG00650,AT5G04130,AT2G26580,AT3G01500,AT2G26500,AT2G26460,AT2G40935,AT2G40890,AT2G47610,AT2G47600,AT3G07680,AT2G45440,AT3G05130,AT2G47490,AT3G05180,AT3G05270,AT2G33240,AT2G33150,AT3G03710,AT1G67740,AT5G12310,AT1G13280,AT3G16320,AT3G16470,AT1G02340,AT1G06310,AT4G17740,AT4G14960,AT4G16845,AT1G18990,AT1G19110,AT4G16515,AT1G20630,AT4G17640,AT4G14430,AT1G20620,AT1G35290,AT4G16410,AT1G20490,AT1G56430,AT4G15730,AT1G77740,AT4G15840,AT1G63080,AT1G68920,AT1G71030,AT1G56700,AT1G29395,AT1G28330,AT5G22060,AT1G80630,AT5G04140,AT1G52220,AT1G72300,AT1G68440,AT1G76730,AT1G45976,AT1G76360,AT1G74170,AT1G75100,AT1G42190,AT1G58380,AT1G76590,AT5G11060,AT5G11070,AT1G76450,AT5G19850,AT1G76490,AT5G19540,AT5G13090,AT5G20660,AT1G68820,AT1G73655,AT5G08330,AT5G10695,AT1G73680,AT5G21170,AT1G73830,AT5G08380,AT1G73660,AT5G20320,AT1G73177,AT1G66640,AT1G71880,AT5G20840,AT1G52760,AT1G52880,AT4G36470,AT1G67623,AT4G36790,AT4G36640,AT1G74670,AT4G37260,AT1G74370,AT1G74430,AT1G68540,AT1G80500,AT1G63670,AT1G70610,AT1G63800,AT1G69210,AT5G17580,AT1G73980,AT1G68160,AT5G15860,AT1G51550,AT5G14920,AT5G15450,AT2G47400,AT2G43530,AT2G43535,AT2G43840,AT2G47390,AT5G35170,AT1G55850,AT5G27650,AT5G27830,AT1G08080,AT1G32440,AT5G27150,AT5G27380,AT1G19300,AT2G23880,AT5G26030,AT1G06180,AT1G78270,AT5G25460,AT1G78240,AT5G25290,AT1G06820,AT1G29260,AT5G67300,AT1G21590,AT5G67320,AT5G67030,AT5G66770,AT5G66590,AT5G66530,AT5G66180,AT5G65660,AT5G65720,AT1G62960,AT5G64860,AT5G64630,AT5G64800,AT5G64460,AT5G64330,AT5G64180,AT1G12900,AT1G19980,AT1G20190,AT5G63620,AT5G63135,AT1G36940,AT5G63420,AT1G18720,AT1G05385,AT1G79610,AT5G62200,AT1G07890,AT1G07700,AT1G14380,AT5G62360,AT5G62140,AT1G14280,AT5G61410,AT1G71820,AT5G61580,AT5G61590,AT1G49780,AT5G60550,AT5G59950,AT1G18310,AT5G58950,AT1G47290,AT1G32670,AT1G76180,AT5G57990,AT1G76100,AT5G58410,AT5G57630,AT5G57290,AT1G80760,AT1G80840,AT1G80920,AT1G65060,AT5G57070,AT1G22530,AT1G22490,AT1G64640,AT1G77920,AT5G54540,AT1G74940,AT1G48350,AT1G48330,AT5G54290,AT5G53370,AT5G53500,AT5G53170,AT1G73020,AT1G49260,AT5G52780,AT5G52390,AT1G49480,AT1G49300,AT1G50320,AT5G52040,AT1G11260,AT1G50250,AT5G51460,AT1G21670,AT5G51040,AT1G34340,AT5G49945,AT5G50250,AT1G13930,AT5G49710,AT1G06470,AT5G49910,AT1G06460,AT5G49450,AT1G06570,AT5G49120,AT1G75830,AT5G48900,AT1G62780,AT5G48485,AT1G43580,AT1G75840,AT5G47910,AT1G12990,AT1G10740,AT5G47350,AT5G47240,AT5G47330,AT1G11680,AT5G45820,AT1G64720,AT5G45170,AT5G45240,AT1G59860,AT1G65800,AT5G44910,AT5G44410,AT5G44020,AT1G75690,AT5G43610,AT1G23390,AT2G27190,AT2G16070,AT1G65190,AT5G42950,AT1G54590,AT1G78460,AT1G78600,AT1G78570,AT5G42100,AT1G03160,AT1G03000,AT5G41600,AT5G41150,AT5G40840,AT5G41140,AT2G31400,AT2G31360,AT2G38800,AT2G04240,AT5G39785,AT5G39740,AT5G38510,AT2G04039,AT2G31510,AT5G37140,AT2G31800,AT2G42600,AT5G24520,AT5G24610,AT5G24530,AT2G04900,AT5G23730,AT5G23870,AT5G23920,AT1G04400,AT5G23350,AT5G23420,AT5G23210,AT1G26830,AT1G60010,AT2G21260,AT5G22460,AT5G19240,AT2G46400,AT5G19140,AT2G36900,AT5G19210,AT5G18850,AT5G18640,AT5G18170,AT2G21880,AT5G18340,AT2G22030,AT2G36320,AT5G17870,AT2G35880,AT2G12870,AT5G16610,AT2G42670,AT2G42790,AT2G21130,AT2G21200,AT2G03750,AT2G03710,AT2G27880,AT2G28000,AT5G14180,AT5G13950,AT1G78995,AT1G78970,AT5G12250,AT5G12200,AT5G12050,AT1G54570,AT1G22770,AT5G11340,AT1G09240,AT5G11150,AT1G78690,AT5G10530,AT5G10430,AT5G10210,AT1G04140,AT1G04120,AT1G70290,AT1G12080,AT5G08520,AT5G08580,AT2G25190,AT2G25080,AT5G08000,AT1G27360,AT5G06690,AT5G06930,AT1G09415,AT1G09570,AT1G10070,AT5G06170,AT1G04750,AT5G05740,AT5G05710,AT5G05200,AT1G09930,AT1G09750,AT5G04810,AT5G04760,AT5G04040,AT5G03795,AT5G03560,AT1G62180,AT5G03470,AT5G03555,AT1G62300,AT1G08540,AT5G03320,AT5G03350,AT1G08550,AT5G03190,AT1G08640,AT1G03600,AT2G17340,AT1G24320,AT5G02710,AT2G17410,AT5G02150,AT1G23080,AT1G23090,AT2G17270,AT1G60800,AT5G01600,AT3G63170,AT3G63160,AT1G76990,AT3G62410,AT3G62550,AT3G62580,AT1G24440,AT3G62700,AT3G62100,AT3G62280,AT3G61630,AT3G61120,AT1G23540,AT3G61430,AT3G61470,AT3G60600,AT2G36690,AT2G43010,AT2G28390,AT3G60130,AT3G59930,AT3G59940,AT3G59920,AT3G60080,AT2G18350,AT2G18390,AT2G18330,AT2G18300,AT3G59060,AT2G16600,AT2G13260,AT2G20830,AT2G46530,AT3G58390,AT2G46550,AT3G58120,AT3G58620,AT2G15960,AT2G15520,AT3G57770,AT2G15880,AT2G07490,AT3G57190,AT3G56660,AT3G56380,AT2G03470,AT2G40110,AT2G32100,AT3G55760,AT3G55770,AT3G55630,AT3G56000,AT3G55330,AT2G35690,AT3G55390,AT2G42390,AT3G55120,AT3G55010,AT3G54720,AT3G54680,AT2G01760,AT3G54810,AT2G42490,AT3G54270,AT2G19650,AT3G53720,AT3G53470,AT2G07738,AT2G18790,AT3G52720,AT2G18700,AT3G52950,AT3G52150,A

GATAAGR	10	2	['4-8']	699	ATMG00650,ATCG00650,AT5G04130,AT2G26580,AT3G01500,AT2G26500,AT2G26460,AT2G40935,AT2G40890,AT2G47610,AT2G47600,AT3G07680,AT2G45440,AT3G05130,AT2G47490,AT3G05180,AT3G05270,AT2G33240,AT2G33150,AT3G03710,AT1G67740,AT5G12310,AT1G13280,AT3G16320,AT3G16470,AT1G02340,AT1G06310,AT4G17740,AT4G14960,AT4G16845,AT1G18990,AT1G19110,AT4G16515,AT1G20630,AT4G17640,AT4G14430,AT1G20620,AT1G35290,AT4G16410,AT1G20490,AT1G56430,AT4G15730,AT1G77740,AT4G15840,AT1G63080,AT1G68920,AT1G71030,AT1G56700,AT1G29395,AT1G28330,AT5G22060,AT1G80630,AT5G04140,AT1G52220,AT1G72300,AT1G68440,AT1G76730,AT1G45976,AT1G76360,AT1G74170,AT1G75100,AT1G42190,AT1G58380,AT1G76590,AT5G11060,AT5G11070,AT1G76450,AT5G19850,AT1G76490,AT5G19540,AT5G13090,AT5G20660,AT1G68820,AT1G73655,AT5G08330,AT5G10695,AT1G73680,AT5G21170,AT1G73830,AT5G08380,AT1G73660,AT5G20320,AT1G73177,AT1G66640,AT1G71880,AT5G20840,AT1G52760,AT1G52880,AT4G36470,AT1G67623,AT4G36790,AT4G36640,AT1G74670,AT4G37260,AT1G74370,AT1G74430,AT1G68540,AT1G80500,AT1G63670,AT1G70610,AT1G63800,AT1G69210,AT5G17580,AT1G73980,AT1G68160,AT5G15860,AT1G51550,AT5G14920,AT5G15450,AT2G47400,AT2G43530,AT2G43535,AT2G43840,AT2G47390,AT5G35170,AT1G55850,AT5G27650,AT5G27830,AT1G08080,AT1G32440,AT5G27150,AT5G27380,AT1G19300,AT2G23880,AT5G26030,AT1G06180,AT1G78270,AT5G25460,AT1G78240,AT5G25290,AT1G06820,AT1G29260,AT5G67300,AT1G21590,AT5G67320,AT5G67030,AT5G66770,AT5G66590,AT5G66530,AT5G66180,AT5G65660,AT5G65720,AT1G62960,AT5G64860,AT5G64630,AT5G64800,AT5G64460,AT5G64330,AT5G64180,AT1G12900,AT1G19980,AT1G20190,AT5G63620,AT5G63135,AT1G36940,AT5G63420,AT1G18720,AT1G05385,AT1G79610,AT5G62200,AT1G07890,AT1G07700,AT1G14380,AT5G62360,AT5G62140,AT1G14280,AT5G61410,AT1G71820,AT5G61580,AT5G61590,AT1G49780,AT5G60550,AT5G59950,AT1G18310,AT5G58950,AT1G47290,AT1G32670,AT1G76180,AT5G57990,AT1G76100,AT5G58410,AT5G57630,AT5G57290,AT1G80760,AT1G80840,AT1G80920,AT1G65060,AT5G57070,AT1G22530,AT1G22490,AT1G64640,AT1G77920,AT5G54540,AT1G74940,AT1G48350,AT1G48330,AT5G54290,AT5G53370,AT5G53500,AT5G53170,AT1G73020,AT1G49260,AT5G52780,AT5G52390,AT1G49480,AT1G49300,AT1G50320,AT5G52040,AT1G11260,AT1G50250,AT5G51460,AT1G21670,AT5G51040,AT1G34340,AT5G49945,AT5G50250,AT1G13930,AT5G49710,AT1G06470,AT5G49910,AT1G06460,AT5G49450,AT1G06570,AT5G49120,AT1G75830,AT5G48900,AT1G62780,AT5G48485,AT1G43580,AT1G75840,AT5G47910,AT1G12990,AT1G10740,AT5G47350,AT5G47240,AT5G47330,AT1G11680,AT5G45820,AT1G64720,AT5G45170,AT5G45240,AT1G59860,AT1G65800,AT5G44910,AT5G44410,AT5G44020,AT1G75690,AT5G43610,AT1G23390,AT2G27190,AT2G16070,AT1G65190,AT5G42950,AT1G54590,AT1G78460,AT1G78600,AT1G78570,AT5G42100,AT1G03160,AT1G03000,AT5G41600,AT5G41150,AT5G40840,AT5G41140,AT2G31400,AT2G31360,AT2G38800,AT2G04240,AT5G39785,AT5G39740,AT5G38510,AT2G04039,AT2G31510,AT5G37140,AT2G31800,AT2G42600,AT5G24520,AT5G24610,AT5G24530,AT2G04900,AT5G23730,AT5G23870,AT5G23920,AT1G04400,AT5G23350,AT5G23420,AT5G23210,AT1G26830,AT1G60010,AT2G21260,AT5G22460,AT5G19240,AT2G46400,AT5G19140,AT2G36900,AT5G19210,AT5G18850,AT5G18640,AT5G18170,AT2G21880,AT5G18340,AT2G22030,AT2G36320,AT5G17870,AT2G35880,AT2G12870,AT5G16610,AT2G42670,AT2G42790,AT2G21130,AT2G21200,AT2G03750,AT2G03710,AT2G27880,AT2G28000,AT5G14180,AT5G13950,AT1G78995,AT1G78970,AT5G12250,AT5G12200,AT5G12050,AT1G54570,AT1G22770,AT5G11340,AT1G09240,AT5G11150,AT1G78690,AT5G10530,AT5G10430,AT5G10210,AT1G04140,AT1G04120,AT1G70290,AT1G12080,AT5G08520,AT5G08580,AT2G25190,AT2G25080,AT5G08000,AT1G27360,AT5G06690,AT5G06930,AT1G09415,AT1G09570,AT1G10070,AT5G06170,AT1G04750,AT5G05740,AT5G05710,AT5G05200,AT1G09930,AT1G09750,AT5G04810,AT5G04760,AT5G04040,AT5G03795,AT5G03560,AT1G62180,AT5G03470,AT5G03555,AT1G62300,AT1G08540,AT5G03320,AT5G03350,AT1G08550,AT5G03190,AT1G08640,AT1G03600,AT2G17340,AT1G24320,AT5G02710,AT2G17410,AT5G02150,AT1G23080,AT1G23090,AT2G17270,AT1G60800,AT5G01600,AT3G63170,AT3G63160,AT1G76990,AT3G62410,AT3G62550,AT3G62580,AT1G24440,AT3G62700,AT3G62100,AT3G62280,AT3G61630,AT3G61120,AT1G23540,AT3G61430,AT3G61470,AT3G60600,AT2G36690,AT2G43010,AT2G28390,AT3G60130,AT3G59930,AT3G59940,AT3G59920,AT3G60080,AT2G18350,AT2G18390,AT2G18330,AT2G18300,AT3G59060,AT2G16600,AT2G13260,AT2G20830,AT2G46530,AT3G58390,AT2G46550,AT3G58120,AT3G58620,AT2G15960,AT2G15520,AT3G57770,AT2G15880,AT2G07490,AT3G57190,AT3G56660,AT3G56380,AT2G03470,AT2G40110,AT2G32100,AT3G55760,AT3G55770,AT3G55630,AT3G56000,AT3G55330,AT2G35690,AT3G55390,AT2G42390,AT3G55120,AT3G55010,AT3G54720,AT3G54680,AT2G01760,AT3G54810,AT2G42490,AT3G54270,AT2G19650,AT3G53720,AT3G53470,AT2G07738,AT2G18790,AT3G52720,AT2G18700,AT3G52950,AT3G52150,A

GATAAGR	10	3	['4-8']	699	ATMG00650,ATCG00650,AT5G04130,AT2G26580,AT3G01500,AT2G26500,AT2G26460,AT2G40935,AT2G40890,AT2G47610,AT2G47600,AT3G07680,AT2G45440,AT3G05130,AT2G47490,AT3G05180,AT3G05270,AT2G33240,AT2G33150,AT3G03710,AT1G67740,AT5G12310,AT1G13280,AT3G16320,AT3G16470,AT1G02340,AT1G06310,AT4G17740,AT4G14960,AT4G16845,AT1G18990,AT1G19110,AT4G16515,AT1G20630,AT4G17640,AT4G14430,AT1G20620,AT1G35290,AT4G16410,AT1G20490,AT1G56430,AT4G15730,AT1G77740,AT4G15840,AT1G63080,AT1G68920,AT1G71030,AT1G56700,AT1G29395,AT1G28330,AT5G22060,AT1G80630,AT5G04140,AT1G52220,AT1G72300,AT1G68440,AT1G76730,AT1G45976,AT1G76360,AT1G74170,AT1G75100,AT1G42190,AT1G58380,AT1G76590,AT5G11060,AT5G11070,AT1G76450,AT5G19850,AT1G76490,AT5G19540,AT5G13090,AT5G20660,AT1G68820,AT1G73655,AT5G08330,AT5G10695,AT1G73680,AT5G21170,AT1G73830,AT5G08380,AT1G73660,AT5G20320,AT1G73177,AT1G66640,AT1G71880,AT5G20840,AT1G52760,AT1G52880,AT4G36470,AT1G67623,AT4G36790,AT4G36640,AT1G74670,AT4G37260,AT1G74370,AT1G74430,AT1G68540,AT1G80500,AT1G63670,AT1G70610,AT1G63800,AT1G69210,AT5G17580,AT1G73980,AT1G68160,AT5G15860,AT1G51550,AT5G14920,AT5G15450,AT2G47400,AT2G43530,AT2G43535,AT2G43840,AT2G47390,AT5G35170,AT1G55850,AT5G27650,AT5G27830,AT1G08080,AT1G32440,AT5G27150,AT5G27380,AT1G19300,AT2G23880,AT5G26030,AT1G06180,AT1G78270,AT5G25460,AT1G78240,AT5G25290,AT1G06820,AT1G29260,AT5G67300,AT1G21590,AT5G67320,AT5G67030,AT5G66770,AT5G66590,AT5G66530,AT5G66180,AT5G65660,AT5G65720,AT1G62960,AT5G64860,AT5G64630,AT5G64800,AT5G64460,AT5G64330,AT5G64180,AT1G12900,AT1G19980,AT1G20190,AT5G63620,AT5G63135,AT1G36940,AT5G63420,AT1G18720,AT1G05385,AT1G79610,AT5G62200,AT1G07890,AT1G07700,AT1G14380,AT5G62360,AT5G62140,AT1G14280,AT5G61410,AT1G71820,AT5G61580,AT5G61590,AT1G49780,AT5G60550,AT5G59950,AT1G18310,AT5G58950,AT1G47290,AT1G32670,AT1G76180,AT5G57990,AT1G76100,AT5G58410,AT5G57630,AT5G57290,AT1G80760,AT1G80840,AT1G80920,AT1G65060,AT5G57070,AT1G22530,AT1G22490,AT1G64640,AT1G77920,AT5G54540,AT1G74940,AT1G48350,AT1G48330,AT5G54290,AT5G53370,AT5G53500,AT5G53170,AT1G73020,AT1G49260,AT5G52780,AT5G52390,AT1G49480,AT1G49300,AT1G50320,AT5G52040,AT1G11260,AT1G50250,AT5G51460,AT1G21670,AT5G51040,AT1G34340,AT5G49945,AT5G50250,AT1G13930,AT5G49710,AT1G06470,AT5G49910,AT1G06460,AT5G49450,AT1G06570,AT5G49120,AT1G75830,AT5G48900,AT1G62780,AT5G48485,AT1G43580,AT1G75840,AT5G47910,AT1G12990,AT1G10740,AT5G47350,AT5G47240,AT5G47330,AT1G11680,AT5G45820,AT1G64720,AT5G45170,AT5G45240,AT1G59860,AT1G65800,AT5G44910,AT5G44410,AT5G44020,AT1G75690,AT5G43610,AT1G23390,AT2G27190,AT2G16070,AT1G65190,AT5G42950,AT1G54590,AT1G78460,AT1G78600,AT1G78570,AT5G42100,AT1G03160,AT1G03000,AT5G41600,AT5G41150,AT5G40840,AT5G41140,AT2G31400,AT2G31360,AT2G38800,AT2G04240,AT5G39785,AT5G39740,AT5G38510,AT2G04039,AT2G31510,AT5G37140,AT2G31800,AT2G42600,AT5G24520,AT5G24610,AT5G24530,AT2G04900,AT5G23730,AT5G23870,AT5G23920,AT1G04400,AT5G23350,AT5G23420,AT5G23210,AT1G26830,AT1G60010,AT2G21260,AT5G22460,AT5G19240,AT2G46400,AT5G19140,AT2G36900,AT5G19210,AT5G18850,AT5G18640,AT5G18170,AT2G21880,AT5G18340,AT2G22030,AT2G36320,AT5G17870,AT2G35880,AT2G12870,AT5G16610,AT2G42670,AT2G42790,AT2G21130,AT2G21200,AT2G03750,AT2G03710,AT2G27880,AT2G28000,AT5G14180,AT5G13950,AT1G78995,AT1G78970,AT5G12250,AT5G12200,AT5G12050,AT1G54570,AT1G22770,AT5G11340,AT1G09240,AT5G11150,AT1G78690,AT5G10530,AT5G10430,AT5G10210,AT1G04140,AT1G04120,AT1G70290,AT1G12080,AT5G08520,AT5G08580,AT2G25190,AT2G25080,AT5G08000,AT1G27360,AT5G06690,AT5G06930,AT1G09415,AT1G09570,AT1G10070,AT5G06170,AT1G04750,AT5G05740,AT5G05710,AT5G05200,AT1G09930,AT1G09750,AT5G04810,AT5G04760,AT5G04040,AT5G03795,AT5G03560,AT1G62180,AT5G03470,AT5G03555,AT1G62300,AT1G08540,AT5G03320,AT5G03350,AT1G08550,AT5G03190,AT1G08640,AT1G03600,AT2G17340,AT1G24320,AT5G02710,AT2G17410,AT5G02150,AT1G23080,AT1G23090,AT2G17270,AT1G60800,AT5G01600,AT3G63170,AT3G63160,AT1G76990,AT3G62410,AT3G62550,AT3G62580,AT1G24440,AT3G62700,AT3G62100,AT3G62280,AT3G61630,AT3G61120,AT1G23540,AT3G61430,AT3G61470,AT3G60600,AT2G36690,AT2G43010,AT2G28390,AT3G60130,AT3G59930,AT3G59940,AT3G59920,AT3G60080,AT2G18350,AT2G18390,AT2G18330,AT2G18300,AT3G59060,AT2G16600,AT2G13260,AT2G20830,AT2G46530,AT3G58390,AT2G46550,AT3G58120,AT3G58620,AT2G15960,AT2G15520,AT3G57770,AT2G15880,AT2G07490,AT3G57190,AT3G56660,AT3G56380,AT2G03470,AT2G40110,AT2G32100,AT3G55760,AT3G55770,AT3G55630,AT3G56000,AT3G55330,AT2G35690,AT3G55390,AT2G42390,AT3G55120,AT3G55010,AT3G54720,AT3G54680,AT2G01760,AT3G54810,AT2G42490,AT3G54270,AT2G19650,AT3G53720,AT3G53470,AT2G07738,AT2G18790,AT3G52720,AT2G18700,AT3G52950,AT3G52150,A

GATAAGR	10	4	['4-8']	699	ATMG00650,ATCG00650,AT5G04130,AT2G26580,AT3G01500,AT2G26500,AT2G26460,AT2G40935,AT2G40890,AT2G47610,AT2G47600,AT3G07680,AT2G45440,AT3G05130,AT2G47490,AT3G05180,AT3G05270,AT2G33240,AT2G33150,AT3G03710,AT1G67740,AT5G12310,AT1G13280,AT3G16320,AT3G16470,AT1G02340,AT1G06310,AT4G17740,AT4G14960,AT4G16845,AT1G18990,AT1G19110,AT4G16515,AT1G20630,AT4G17640,AT4G14430,AT1G20620,AT1G35290,AT4G16410,AT1G20490,AT1G56430,AT4G15730,AT1G77740,AT4G15840,AT1G63080,AT1G68920,AT1G71030,AT1G56700,AT1G29395,AT1G28330,AT5G22060,AT1G80630,AT5G04140,AT1G52220,AT1G72300,AT1G68440,AT1G76730,AT1G45976,AT1G76360,AT1G74170,AT1G75100,AT1G42190,AT1G58380,AT1G76590,AT5G11060,AT5G11070,AT1G76450,AT5G19850,AT1G76490,AT5G19540,AT5G13090,AT5G20660,AT1G68820,AT1G73655,AT5G08330,AT5G10695,AT1G73680,AT5G21170,AT1G73830,AT5G08380,AT1G73660,AT5G20320,AT1G73177,AT1G66640,AT1G71880,AT5G20840,AT1G52760,AT1G52880,AT4G36470,AT1G67623,AT4G36790,AT4G36640,AT1G74670,AT4G37260,AT1G74370,AT1G74430,AT1G68540,AT1G80500,AT1G63670,AT1G70610,AT1G63800,AT1G69210,AT5G17580,AT1G73980,AT1G68160,AT5G15860,AT1G51550,AT5G14920,AT5G15450,AT2G47400,AT2G43530,AT2G43535,AT2G43840,AT2G47390,AT5G35170,AT1G55850,AT5G27650,AT5G27830,AT1G08080,AT1G32440,AT5G27150,AT5G27380,AT1G19300,AT2G23880,AT5G26030,AT1G06180,AT1G78270,AT5G25460,AT1G78240,AT5G25290,AT1G06820,AT1G29260,AT5G67300,AT1G21590,AT5G67320,AT5G67030,AT5G66770,AT5G66590,AT5G66530,AT5G66180,AT5G65660,AT5G65720,AT1G62960,AT5G64860,AT5G64630,AT5G64800,AT5G64460,AT5G64330,AT5G64180,AT1G12900,AT1G19980,AT1G20190,AT5G63620,AT5G63135,AT1G36940,AT5G63420,AT1G18720,AT1G05385,AT1G79610,AT5G62200,AT1G07890,AT1G07700,AT1G14380,AT5G62360,AT5G62140,AT1G14280,AT5G61410,AT1G71820,AT5G61580,AT5G61590,AT1G49780,AT5G60550,AT5G59950,AT1G18310,AT5G58950,AT1G47290,AT1G32670,AT1G76180,AT5G57990,AT1G76100,AT5G58410,AT5G57630,AT5G57290,AT1G80760,AT1G80840,AT1G80920,AT1G65060,AT5G57070,AT1G22530,AT1G22490,AT1G64640,AT1G77920,AT5G54540,AT1G74940,AT1G48350,AT1G48330,AT5G54290,AT5G53370,AT5G53500,AT5G53170,AT1G73020,AT1G49260,AT5G52780,AT5G52390,AT1G49480,AT1G49300,AT1G50320,AT5G52040,AT1G11260,AT1G50250,AT5G51460,AT1G21670,AT5G51040,AT1G34340,AT5G49945,AT5G50250,AT1G13930,AT5G49710,AT1G06470,AT5G49910,AT1G06460,AT5G49450,AT1G06570,AT5G49120,AT1G75830,AT5G48900,AT1G62780,AT5G48485,AT1G43580,AT1G75840,AT5G47910,AT1G12990,AT1G10740,AT5G47350,AT5G47240,AT5G47330,AT1G11680,AT5G45820,AT1G64720,AT5G45170,AT5G45240,AT1G59860,AT1G65800,AT5G44910,AT5G44410,AT5G44020,AT1G75690,AT5G43610,AT1G23390,AT2G27190,AT2G16070,AT1G65190,AT5G42950,AT1G54590,AT1G78460,AT1G78600,AT1G78570,AT5G42100,AT1G03160,AT1G03000,AT5G41600,AT5G41150,AT5G40840,AT5G41140,AT2G31400,AT2G31360,AT2G38800,AT2G04240,AT5G39785,AT5G39740,AT5G38510,AT2G04039,AT2G31510,AT5G37140,AT2G31800,AT2G42600,AT5G24520,AT5G24610,AT5G24530,AT2G04900,AT5G23730,AT5G23870,AT5G23920,AT1G04400,AT5G23350,AT5G23420,AT5G23210,AT1G26830,AT1G60010,AT2G21260,AT5G22460,AT5G19240,AT2G46400,AT5G19140,AT2G36900,AT5G19210,AT5G18850,AT5G18640,AT5G18170,AT2G21880,AT5G18340,AT2G22030,AT2G36320,AT5G17870,AT2G35880,AT2G12870,AT5G16610,AT2G42670,AT2G42790,AT2G21130,AT2G21200,AT2G03750,AT2G03710,AT2G27880,AT2G28000,AT5G14180,AT5G13950,AT1G78995,AT1G78970,AT5G12250,AT5G12200,AT5G12050,AT1G54570,AT1G22770,AT5G11340,AT1G09240,AT5G11150,AT1G78690,AT5G10530,AT5G10430,AT5G10210,AT1G04140,AT1G04120,AT1G70290,AT1G12080,AT5G08520,AT5G08580,AT2G25190,AT2G25080,AT5G08000,AT1G27360,AT5G06690,AT5G06930,AT1G09415,AT1G09570,AT1G10070,AT5G06170,AT1G04750,AT5G05740,AT5G05710,AT5G05200,AT1G09930,AT1G09750,AT5G04810,AT5G04760,AT5G04040,AT5G03795,AT5G03560,AT1G62180,AT5G03470,AT5G03555,AT1G62300,AT1G08540,AT5G03320,AT5G03350,AT1G08550,AT5G03190,AT1G08640,AT1G03600,AT2G17340,AT1G24320,AT5G02710,AT2G17410,AT5G02150,AT1G23080,AT1G23090,AT2G17270,AT1G60800,AT5G01600,AT3G63170,AT3G63160,AT1G76990,AT3G62410,AT3G62550,AT3G62580,AT1G24440,AT3G62700,AT3G62100,AT3G62280,AT3G61630,AT3G61120,AT1G23540,AT3G61430,AT3G61470,AT3G60600,AT2G36690,AT2G43010,AT2G28390,AT3G60130,AT3G59930,AT3G59940,AT3G59920,AT3G60080,AT2G18350,AT2G18390,AT2G18330,AT2G18300,AT3G59060,AT2G16600,AT2G13260,AT2G20830,AT2G46530,AT3G58390,AT2G46550,AT3G58120,AT3G58620,AT2G15960,AT2G15520,AT3G57770,AT2G15880,AT2G07490,AT3G57190,AT3G56660,AT3G56380,AT2G03470,AT2G40110,AT2G32100,AT3G55760,AT3G55770,AT3G55630,AT3G56000,AT3G55330,AT2G35690,AT3G55390,AT2G42390,AT3G55120,AT3G55010,AT3G54720,AT3G54680,AT2G01760,AT3G54810,AT2G42490,AT3G54270,AT2G19650,AT3G53720,AT3G53470,AT2G07738,AT2G18790,AT3G52720,AT2G18700,AT3G52950,AT3G52150,A

GATAAGR	10	5	['4-8']	699	ATMG00650,ATCG00650,AT5G04130,AT2G26580,AT3G01500,AT2G26500,AT2G26460,AT2G40935,AT2G40890,AT2G47610,AT2G47600,AT3G07680,AT2G45440,AT3G05130,AT2G47490,AT3G05180,AT3G05270,AT2G33240,AT2G33150,AT3G03710,AT1G67740,AT5G12310,AT1G13280,AT3G16320,AT3G16470,AT1G02340,AT1G06310,AT4G17740,AT4G14960,AT4G16845,AT1G18990,AT1G19110,AT4G16515,AT1G20630,AT4G17640,AT4G14430,AT1G20620,AT1G35290,AT4G16410,AT1G20490,AT1G56430,AT4G15730,AT1G77740,AT4G15840,AT1G63080,AT1G68920,AT1G71030,AT1G56700,AT1G29395,AT1G28330,AT5G22060,AT1G80630,AT5G04140,AT1G52220,AT1G72300,AT1G68440,AT1G76730,AT1G45976,AT1G76360,AT1G74170,AT1G75100,AT1G42190,AT1G58380,AT1G76590,AT5G11060,AT5G11070,AT1G76450,AT5G19850,AT1G76490,AT5G19540,AT5G13090,AT5G20660,AT1G68820,AT1G73655,AT5G08330,AT5G10695,AT1G73680,AT5G21170,AT1G73830,AT5G08380,AT1G73660,AT5G20320,AT1G73177,AT1G66640,AT1G71880,AT5G20840,AT1G52760,AT1G52880,AT4G36470,AT1G67623,AT4G36790,AT4G36640,AT1G74670,AT4G37260,AT1G74370,AT1G74430,AT1G68540,AT1G80500,AT1G63670,AT1G70610,AT1G63800,AT1G69210,AT5G17580,AT1G73980,AT1G68160,AT5G15860,AT1G51550,AT5G14920,AT5G15450,AT2G47400,AT2G43530,AT2G43535,AT2G43840,AT2G47390,AT5G35170,AT1G55850,AT5G27650,AT5G27830,AT1G08080,AT1G32440,AT5G27150,AT5G27380,AT1G19300,AT2G23880,AT5G26030,AT1G06180,AT1G78270,AT5G25460,AT1G78240,AT5G25290,AT1G06820,AT1G29260,AT5G67300,AT1G21590,AT5G67320,AT5G67030,AT5G66770,AT5G66590,AT5G66530,AT5G66180,AT5G65660,AT5G65720,AT1G62960,AT5G64860,AT5G64630,AT5G64800,AT5G64460,AT5G64330,AT5G64180,AT1G12900,AT1G19980,AT1G20190,AT5G63620,AT5G63135,AT1G36940,AT5G63420,AT1G18720,AT1G05385,AT1G79610,AT5G62200,AT1G07890,AT1G07700,AT1G14380,AT5G62360,AT5G62140,AT1G14280,AT5G61410,AT1G71820,AT5G61580,AT5G61590,AT1G49780,AT5G60550,AT5G59950,AT1G18310,AT5G58950,AT1G47290,AT1G32670,AT1G76180,AT5G57990,AT1G76100,AT5G58410,AT5G57630,AT5G57290,AT1G80760,AT1G80840,AT1G80920,AT1G65060,AT5G57070,AT1G22530,AT1G22490,AT1G64640,AT1G77920,AT5G54540,AT1G74940,AT1G48350,AT1G48330,AT5G54290,AT5G53370,AT5G53500,AT5G53170,AT1G73020,AT1G49260,AT5G52780,AT5G52390,AT1G49480,AT1G49300,AT1G50320,AT5G52040,AT1G11260,AT1G50250,AT5G51460,AT1G21670,AT5G51040,AT1G34340,AT5G49945,AT5G50250,AT1G13930,AT5G49710,AT1G06470,AT5G49910,AT1G06460,AT5G49450,AT1G06570,AT5G49120,AT1G75830,AT5G48900,AT1G62780,AT5G48485,AT1G43580,AT1G75840,AT5G47910,AT1G12990,AT1G10740,AT5G47350,AT5G47240,AT5G47330,AT1G11680,AT5G45820,AT1G64720,AT5G45170,AT5G45240,AT1G59860,AT1G65800,AT5G44910,AT5G44410,AT5G44020,AT1G75690,AT5G43610,AT1G23390,AT2G27190,AT2G16070,AT1G65190,AT5G42950,AT1G54590,AT1G78460,AT1G78600,AT1G78570,AT5G42100,AT1G03160,AT1G03000,AT5G41600,AT5G41150,AT5G40840,AT5G41140,AT2G31400,AT2G31360,AT2G38800,AT2G04240,AT5G39785,AT5G39740,AT5G38510,AT2G04039,AT2G31510,AT5G37140,AT2G31800,AT2G42600,AT5G24520,AT5G24610,AT5G24530,AT2G04900,AT5G23730,AT5G23870,AT5G23920,AT1G04400,AT5G23350,AT5G23420,AT5G23210,AT1G26830,AT1G60010,AT2G21260,AT5G22460,AT5G19240,AT2G46400,AT5G19140,AT2G36900,AT5G19210,AT5G18850,AT5G18640,AT5G18170,AT2G21880,AT5G18340,AT2G22030,AT2G36320,AT5G17870,AT2G35880,AT2G12870,AT5G16610,AT2G42670,AT2G42790,AT2G21130,AT2G21200,AT2G03750,AT2G03710,AT2G27880,AT2G28000,AT5G14180,AT5G13950,AT1G78995,AT1G78970,AT5G12250,AT5G12200,AT5G12050,AT1G54570,AT1G22770,AT5G11340,AT1G09240,AT5G11150,AT1G78690,AT5G10530,AT5G10430,AT5G10210,AT1G04140,AT1G04120,AT1G70290,AT1G12080,AT5G08520,AT5G08580,AT2G25190,AT2G25080,AT5G08000,AT1G27360,AT5G06690,AT5G06930,AT1G09415,AT1G09570,AT1G10070,AT5G06170,AT1G04750,AT5G05740,AT5G05710,AT5G05200,AT1G09930,AT1G09750,AT5G04810,AT5G04760,AT5G04040,AT5G03795,AT5G03560,AT1G62180,AT5G03470,AT5G03555,AT1G62300,AT1G08540,AT5G03320,AT5G03350,AT1G08550,AT5G03190,AT1G08640,AT1G03600,AT2G17340,AT1G24320,AT5G02710,AT2G17410,AT5G02150,AT1G23080,AT1G23090,AT2G17270,AT1G60800,AT5G01600,AT3G63170,AT3G63160,AT1G76990,AT3G62410,AT3G62550,AT3G62580,AT1G24440,AT3G62700,AT3G62100,AT3G62280,AT3G61630,AT3G61120,AT1G23540,AT3G61430,AT3G61470,AT3G60600,AT2G36690,AT2G43010,AT2G28390,AT3G60130,AT3G59930,AT3G59940,AT3G59920,AT3G60080,AT2G18350,AT2G18390,AT2G18330,AT2G18300,AT3G59060,AT2G16600,AT2G13260,AT2G20830,AT2G46530,AT3G58390,AT2G46550,AT3G58120,AT3G58620,AT2G15960,AT2G15520,AT3G57770,AT2G15880,AT2G07490,AT3G57190,AT3G56660,AT3G56380,AT2G03470,AT2G40110,AT2G32100,AT3G55760,AT3G55770,AT3G55630,AT3G56000,AT3G55330,AT2G35690,AT3G55390,AT2G42390,AT3G55120,AT3G55010,AT3G54720,AT3G54680,AT2G01760,AT3G54810,AT2G42490,AT3G54270,AT2G19650,AT3G53720,AT3G53470,AT2G07738,AT2G18790,AT3G52720,AT2G18700,AT3G52950,AT3G52150,A
GATGAYRTGG	10	1	['0-4', '4-8', '12-16', '20-24']	39	AT1G77000,AT1G63630,AT3G62580,AT5G26820,AT5G49540,AT5G24810,AT5G36170,AT3G04460,AT4G26860,AT1G59218,AT1G58807,AT5G20070,AT4G37480,AT3G08730,AT1G17745,AT3G22200,AT4G16760,AT1G70660,AT1G07140,AT1G19140,AT1G07280,AT1G20696,AT3G19450,AT5G18850,AT4G33980,AT5G03905,AT3G03180,AT2G36430,AT2G28200,AT1G08720,AT5G14920,AT3G21690,AT3G01860,AT2G42220,AT4G30740,AT5G02190,AT1G05570,AT5G61380,AT1G32130
GATGAYRTGG	10	2	['0-4', '4-8', '12-16', '20-24']	39	AT1G77000,AT1G63630,AT3G62580,AT5G26820,AT5G49540,AT5G24810,AT5G36170,AT3G04460,AT4G26860,AT1G59218,AT1G58807,AT5G20070,AT4G37480,AT3G08730,AT1G17745,AT3G22200,AT4G16760,AT1G70660,AT1G07140,AT1G19140,AT1G07280,AT1G20696,AT3G19450,AT5G18850,AT4G33980,AT5G03905,AT3G03180,AT2G36430,AT2G28200,AT1G08720,AT5G14920,AT3G21690,AT3G01860,AT2G42220,AT4G30740,AT5G02190,AT1G05570,AT5G61380,AT1G32130

GATGAYRTGG	10	3	['0-4', '4-8', '12-16', '20-24']	39	AT1G77000,AT1G63630,AT3G62580,AT5G26820,AT5G49540,AT5G24810,AT5G36170,AT3G04460,AT4G26860,AT1G59218,AT1G58807,AT5G20070,AT4G37480,AT3G08730,AT1G17745,AT3G22200,AT4G16760,AT1G70660,AT1G07140,AT1G19140,AT1G07280,AT1G20696,AT3G19450,AT5G18850,AT4G33980,AT5G03905,AT3G03180,AT2G36430,AT2G28200,AT1G08720,AT5G14920,AT3G21690,AT3G01860,AT2G42220,AT4G30740,AT5G02190,AT1G05570,AT5G61380,AT1G32130
GATGAYRTGG	10	4	['0-4', '4-8', '12-16', '20-24']	39	AT1G77000,AT1G63630,AT3G62580,AT5G26820,AT5G49540,AT5G24810,AT5G36170,AT3G04460,AT4G26860,AT1G59218,AT1G58807,AT5G20070,AT4G37480,AT3G08730,AT1G17745,AT3G22200,AT4G16760,AT1G70660,AT1G07140,AT1G19140,AT1G07280,AT1G20696,AT3G19450,AT5G18850,AT4G33980,AT5G03905,AT3G03180,AT2G36430,AT2G28200,AT1G08720,AT5G14920,AT3G21690,AT3G01860,AT2G42220,AT4G30740,AT5G02190,AT1G05570,AT5G61380,AT1G32130
GATGAYRTGG	10	5	['0-4', '4-8', '12-16', '20-24']	39	AT1G77000,AT1G63630,AT3G62580,AT5G26820,AT5G49540,AT5G24810,AT5G36170,AT3G04460,AT4G26860,AT1G59218,AT1G58807,AT5G20070,AT4G37480,AT3G08730,AT1G17745,AT3G22200,AT4G16760,AT1G70660,AT1G07140,AT1G19140,AT1G07280,AT1G20696,AT3G19450,AT5G18850,AT4G33980,AT5G03905,AT3G03180,AT2G36430,AT2G28200,AT1G08720,AT5G14920,AT3G21690,AT3G01860,AT2G42220,AT4G30740,AT5G02190,AT1G05570,AT5G61380,AT1G32130
GCCACGTGGC	10	1	['4-8', '12-16', '20-24']	13	AT4G16515,AT1G10960,AT5G67370,AT1G28530,AT2G47780,AT2G15970,AT2G34460,AT1G52220,AT1G18310,AT1G52230,AT1G06680,AT4G09620,AT4G03560
GCCACGTGGC	10	2	['4-8', '12-16']	13	AT4G16515,AT1G10960,AT5G67370,AT1G28530,AT2G47780,AT2G15970,AT2G34460,AT1G52220,AT1G18310,AT1G52230,AT1G06680,AT4G09620,AT4G03560
GCCACGTGGC	10	3	['4-8', '12-16', '20-24']	13	AT4G16515,AT1G10960,AT5G67370,AT1G28530,AT2G47780,AT2G15970,AT2G34460,AT1G52220,AT1G18310,AT1G52230,AT1G06680,AT4G09620,AT4G03560
GCCACGTGGC	10	4	['4-8', '12-16', '20-24']	13	AT4G16515,AT1G10960,AT5G67370,AT1G28530,AT2G47780,AT2G15970,AT2G34460,AT1G52220,AT1G18310,AT1G52230,AT1G06680,AT4G09620,AT4G03560
GCCACGTGGC	10	5	['4-8', '12-16', '20-24']	13	AT4G16515,AT1G10960,AT5G67370,AT1G28530,AT2G47780,AT2G15970,AT2G34460,AT1G52220,AT1G18310,AT1G52230,AT1G06680,AT4G09620,AT4G03560
GCGGCAAA	10	1	['12-16', '16-20', '20-24']	37	AT5G51010,AT5G04130,AT1G31330,AT3G22430,AT4G27790,AT1G76460,AT1G68000,AT2G24930,AT1G76100,AT3G60750,AT5G57760,AT1G53440,AT4G36750,AT2G20260,AT3G05940,AT5G46020,AT3G24010,AT1G09870,AT3G47340,AT5G18540,AT3G03180,AT5G54970,AT2G35450,AT2G30490,AT3G12350,AT1G77570,AT3G46510,AT3G12290,AT5G53290,AT3G21560,AT3G02340,AT5G02650,AT2G35780,AT4G30350,AT5G41150,AT2G01760,AT5G41140
GCGGCAAA	10	2	['12-16', '16-20', '20-24']	37	AT5G51010,AT5G04130,AT1G31330,AT3G22430,AT4G27790,AT1G76460,AT1G68000,AT2G24930,AT1G76100,AT3G60750,AT5G57760,AT1G53440,AT4G36750,AT2G20260,AT3G05940,AT5G46020,AT3G24010,AT1G09870,AT3G47340,AT5G18540,AT3G03180,AT5G54970,AT2G35450,AT2G30490,AT3G12350,AT1G77570,AT3G46510,AT3G12290,AT5G53290,AT3G21560,AT3G02340,AT5G02650,AT2G35780,AT4G30350,AT5G41150,AT2G01760,AT5G41140
GCGGCAAA	10	3	['12-16', '16-20', '20-24']	37	AT5G51010,AT5G04130,AT1G31330,AT3G22430,AT4G27790,AT1G76460,AT1G68000,AT2G24930,AT1G76100,AT3G60750,AT5G57760,AT1G53440,AT4G36750,AT2G20260,AT3G05940,AT5G46020,AT3G24010,AT1G09870,AT3G47340,AT5G18540,AT3G03180,AT5G54970,AT2G35450,AT2G30490,AT3G12350,AT1G77570,AT3G46510,AT3G12290,AT5G53290,AT3G21560,AT3G02340,AT5G02650,AT2G35780,AT4G30350,AT5G41150,AT2G01760,AT5G41140
GCGGCAAA	10	4	['12-16', '16-20', '20-24']	37	AT5G51010,AT5G04130,AT1G31330,AT3G22430,AT4G27790,AT1G76460,AT1G68000,AT2G24930,AT1G76100,AT3G60750,AT5G57760,AT1G53440,AT4G36750,AT2G20260,AT3G05940,AT5G46020,AT3G24010,AT1G09870,AT3G47340,AT5G18540,AT3G03180,AT5G54970,AT2G35450,AT2G30490,AT3G12350,AT1G77570,AT3G46510,AT3G12290,AT5G53290,AT3G21560,AT3G02340,AT5G02650,AT2G35780,AT4G30350,AT5G41150,AT2G01760,AT5G41140
GCGGCAAA	10	5	['12-16', '16-20', '20-24']	37	AT5G51010,AT5G04130,AT1G31330,AT3G22430,AT4G27790,AT1G76460,AT1G68000,AT2G24930,AT1G76100,AT3G60750,AT5G57760,AT1G53440,AT4G36750,AT2G20260,AT3G05940,AT5G46020,AT3G24010,AT1G09870,AT3G47340,AT5G18540,AT3G03180,AT5G54970,AT2G35450,AT2G30490,AT3G12350,AT1G77570,AT3G46510,AT3G12290,AT5G53290,AT3G21560,AT3G02340,AT5G02650,AT2G35780,AT4G30350,AT5G41150,AT2G01760,AT5G41140

GCGGGAAA	10	1	['4-8', '12-16']	42	AT3G63160,AT1G31270,AT1G69040,AT2G38410,AT3G07640,AT5G58140,AT1G18040,AT3G08570,AT5G57040,AT1G70730,AT1G74600,AT5G65430,AT3G20810,AT3G59052,AT2G19310,AT1G80310,AT4G17760,AT3G47450,AT3G58040,AT5G44010,AT5G03795,AT4G33700,AT2G07490,AT1G56220,AT1G48350,AT2G25450,AT2G17840,AT3G12290,AT5G02810,AT2G29780,AT5G53370,AT5G02880,AT3G44660,AT1G23210,AT1G07890,AT3G06250,AT5G14240,AT3G06160,AT3G23325,AT4G30470,AT1G71695,AT3G43020
GCGGGAAA	10	2	['4-8', '12-16']	42	AT3G63160,AT1G31270,AT1G69040,AT2G38410,AT3G07640,AT5G58140,AT1G18040,AT3G08570,AT5G57040,AT1G70730,AT1G74600,AT5G65430,AT3G20810,AT3G59052,AT2G19310,AT1G80310,AT4G17760,AT3G47450,AT3G58040,AT5G44010,AT5G03795,AT4G33700,AT2G07490,AT1G56220,AT1G48350,AT2G25450,AT2G17840,AT3G12290,AT5G02810,AT2G29780,AT5G53370,AT5G02880,AT3G44660,AT1G23210,AT1G07890,AT3G06250,AT5G14240,AT3G06160,AT3G23325,AT4G30470,AT1G71695,AT3G43020
GCGGGAAA	10	3	['4-8', '12-16']	42	AT3G63160,AT1G31270,AT1G69040,AT2G38410,AT3G07640,AT5G58140,AT1G18040,AT3G08570,AT5G57040,AT1G70730,AT1G74600,AT5G65430,AT3G20810,AT3G59052,AT2G19310,AT1G80310,AT4G17760,AT3G47450,AT3G58040,AT5G44010,AT5G03795,AT4G33700,AT2G07490,AT1G56220,AT1G48350,AT2G25450,AT2G17840,AT3G12290,AT5G02810,AT2G29780,AT5G53370,AT5G02880,AT3G44660,AT1G23210,AT1G07890,AT3G06250,AT5G14240,AT3G06160,AT3G23325,AT4G30470,AT1G71695,AT3G43020
GCGGGAAA	10	4	['4-8', '12-16']	42	AT3G63160,AT1G31270,AT1G69040,AT2G38410,AT3G07640,AT5G58140,AT1G18040,AT3G08570,AT5G57040,AT1G70730,AT1G74600,AT5G65430,AT3G20810,AT3G59052,AT2G19310,AT1G80310,AT4G17760,AT3G47450,AT3G58040,AT5G44010,AT5G03795,AT4G33700,AT2G07490,AT1G56220,AT1G48350,AT2G25450,AT2G17840,AT3G12290,AT5G02810,AT2G29780,AT5G53370,AT5G02880,AT3G44660,AT1G23210,AT1G07890,AT3G06250,AT5G14240,AT3G06160,AT3G23325,AT4G30470,AT1G71695,AT3G43020
GCGGGAAA	10	5	['4-8', '12-16']	42	AT3G63160,AT1G31270,AT1G69040,AT2G38410,AT3G07640,AT5G58140,AT1G18040,AT3G08570,AT5G57040,AT1G70730,AT1G74600,AT5G65430,AT3G20810,AT3G59052,AT2G19310,AT1G80310,AT4G17760,AT3G47450,AT3G58040,AT5G44010,AT5G03795,AT4G33700,AT2G07490,AT1G56220,AT1G48350,AT2G25450,AT2G17840,AT3G12290,AT5G02810,AT2G29780,AT5G53370,AT5G02880,AT3G44660,AT1G23210,AT1G07890,AT3G06250,AT5G14240,AT3G06160,AT3G23325,AT4G30470,AT1G71695,AT3G43020
GCGGGAAN	10	1	['12-16']	85	AT1G31270,ATCG00650,AT5G49950,AT3G07640,AT2G02280,AT5G48370,AT1G18040,AT1G44478,AT1G07010,AT4G17760,AT3G47450,AT5G44010,AT3G47560,AT1G56220,AT3G12350,AT3G12290,AT2G29780,AT4G14270,AT5G42640,AT3G44660,AT3G06250,AT3G06160,AT5G40850,AT3G43020,AT1G76730,AT2G38410,AT5G35560,AT5G20380,AT5G20900,AT1G79910,AT1G70730,AT1G74600,AT1G26880,AT1G16680,AT2G19310,AT1G80310,AT2G33700,AT4G33700,AT2G02760,AT5G14240,AT1G53210,AT4G30470,AT5G27930,AT1G69040,AT5G11600,AT1G32310,AT5G08100,AT3G17020,AT5G65110,AT5G65430,AT3G20810,AT4G24340,AT5G03795,AT5G03430,AT2G17840,AT5G02810,AT1G79730,AT5G02880,AT1G07890,AT1G23210,AT3G23325,AT1G71695,AT3G63160,AT1G01970,AT1G03900,AT3G61630,AT1G23540,AT5G58140,AT3G08570,AT4G11410,AT3G60350,AT5G57040,AT2G18330,AT3G59052,AT3G58040,AT2G07490,AT3G05720,AT1G48350,AT2G25450,AT5G54080,AT2G24490,AT5G53370,AT3G07390,AT3G07550,AT1G17090
GCGGGAAN	10	2	['12-16']	85	AT1G31270,ATCG00650,AT5G49950,AT3G07640,AT2G02280,AT5G48370,AT1G18040,AT1G44478,AT1G07010,AT4G17760,AT3G47450,AT5G44010,AT3G47560,AT1G56220,AT3G12350,AT3G12290,AT2G29780,AT4G14270,AT5G42640,AT3G44660,AT3G06250,AT3G06160,AT5G40850,AT3G43020,AT1G76730,AT2G38410,AT5G35560,AT5G20380,AT5G20900,AT1G79910,AT1G70730,AT1G74600,AT1G26880,AT1G16680,AT2G19310,AT1G80310,AT2G33700,AT4G33700,AT2G02760,AT5G14240,AT1G53210,AT4G30470,AT5G27930,AT1G69040,AT5G11600,AT1G32310,AT5G08100,AT3G17020,AT5G65110,AT5G65430,AT3G20810,AT4G24340,AT5G03795,AT5G03430,AT2G17840,AT5G02810,AT1G79730,AT5G02880,AT1G07890,AT1G23210,AT3G23325,AT1G71695,AT3G63160,AT1G01970,AT1G03900,AT3G61630,AT1G23540,AT5G58140,AT3G08570,AT4G11410,AT3G60350,AT5G57040,AT2G18330,AT3G59052,AT3G58040,AT2G07490,AT3G05720,AT1G48350,AT2G25450,AT5G54080,AT2G24490,AT5G53370,AT3G07390,AT3G07550,AT1G17090

GCGGGAAN	10	3	['12-16']	85	AT1G31270,ATCG00650,AT5G49950,AT3G07640,AT2G02280,AT5G48370,AT1G18040,AT1G44478,AT1G07010,AT4G17760,AT3G47450,AT5G44010,AT3G47560,AT1G56220,AT3G12350,AT3G12290,AT2G29780,AT4G14270,AT5G42640,AT3G44660,AT3G06250,AT3G06160,AT5G40850,AT3G43020,AT1G76730,AT2G38410,AT5G35560,AT5G20380,AT5G20900,AT1G79910,AT1G70730,AT1G74600,AT1G26880,AT1G16680,AT2G19310,AT1G80310,AT2G33700,AT4G33700,AT2G02760,AT5G14240,AT1G53210,AT4G30470,AT5G27930,AT1G69040,AT5G11600,AT1G32310,AT5G08100,AT3G17020,AT5G65110,AT5G65430,AT3G20810,AT4G24340,AT5G03795,AT5G03430,AT2G17840,AT5G02810,AT1G79730,AT5G02880,AT1G07890,AT1G23210,AT3G23325,AT1G71695,AT3G63160,AT1G01970,AT1G03900,AT3G61630,AT1G23540,AT5G58140,AT3G08570,AT4G11410,AT3G60350,AT5G57040,AT2G18330,AT3G59052,AT3G58040,AT2G07490,AT3G05720,AT1G48350,AT2G25450,AT5G54080,AT2G24490,AT5G53370,AT3G07390,AT3G07550,AT1G17090
GCGGGAAN	10	4	['12-16']	85	AT1G31270,ATCG00650,AT5G49950,AT3G07640,AT2G02280,AT5G48370,AT1G18040,AT1G44478,AT1G07010,AT4G17760,AT3G47450,AT5G44010,AT3G47560,AT1G56220,AT3G12350,AT3G12290,AT2G29780,AT4G14270,AT5G42640,AT3G44660,AT3G06250,AT3G06160,AT5G40850,AT3G43020,AT1G76730,AT2G38410,AT5G35560,AT5G20380,AT5G20900,AT1G79910,AT1G70730,AT1G74600,AT1G26880,AT1G16680,AT2G19310,AT1G80310,AT2G33700,AT4G33700,AT2G02760,AT5G14240,AT1G53210,AT4G30470,AT5G27930,AT1G69040,AT5G11600,AT1G32310,AT5G08100,AT3G17020,AT5G65110,AT5G65430,AT3G20810,AT4G24340,AT5G03795,AT5G03430,AT2G17840,AT5G02810,AT1G79730,AT5G02880,AT1G07890,AT1G23210,AT3G23325,AT1G71695,AT3G63160,AT1G01970,AT1G03900,AT3G61630,AT1G23540,AT5G58140,AT3G08570,AT4G11410,AT3G60350,AT5G57040,AT2G18330,AT3G59052,AT3G58040,AT2G07490,AT3G05720,AT1G48350,AT2G25450,AT5G54080,AT2G24490,AT5G53370,AT3G07390,AT3G07550,AT1G17090
GCGGGAAN	10	5	['12-16']	85	AT1G31270,ATCG00650,AT5G49950,AT3G07640,AT2G02280,AT5G48370,AT1G18040,AT1G44478,AT1G07010,AT4G17760,AT3G47450,AT5G44010,AT3G47560,AT1G56220,AT3G12350,AT3G12290,AT2G29780,AT4G14270,AT5G42640,AT3G44660,AT3G06250,AT3G06160,AT5G40850,AT3G43020,AT1G76730,AT2G38410,AT5G35560,AT5G20380,AT5G20900,AT1G79910,AT1G70730,AT1G74600,AT1G26880,AT1G16680,AT2G19310,AT1G80310,AT2G33700,AT4G33700,AT2G02760,AT5G14240,AT1G53210,AT4G30470,AT5G27930,AT1G69040,AT5G11600,AT1G32310,AT5G08100,AT3G17020,AT5G65110,AT5G65430,AT3G20810,AT4G24340,AT5G03795,AT5G03430,AT2G17840,AT5G02810,AT1G79730,AT5G02880,AT1G07890,AT1G23210,AT3G23325,AT1G71695,AT3G63160,AT1G01970,AT1G03900,AT3G61630,AT1G23540,AT5G58140,AT3G08570,AT4G11410,AT3G60350,AT5G57040,AT2G18330,AT3G59052,AT3G58040,AT2G07490,AT3G05720,AT1G48350,AT2G25450,AT5G54080,AT2G24490,AT5G53370,AT3G07390,AT3G07550,AT1G17090
GCGGTAATT	10	1	['8-12', '12-16']	20	AT1G30800,AT2G33240,AT1G80500,AT1G73177,AT3G62720,AT1G56700,AT1G63830,AT1G72030,AT5G60120,AT5G52390,AT1G01940,AT4G09760,AT5G11270,AT5G54770,AT4G19110,AT1G29900,AT3G01060,AT3G26380,AT2G12870,AT1G62780
GCGGTAATT	10	2	['8-12', '12-16']	20	AT1G30800,AT2G33240,AT1G80500,AT1G73177,AT3G62720,AT1G56700,AT1G63830,AT1G72030,AT5G60120,AT5G52390,AT1G01940,AT4G09760,AT5G11270,AT5G54770,AT4G19110,AT1G29900,AT3G01060,AT3G26380,AT2G12870,AT1G62780
GCGGTAATT	10	3	['8-12', '12-16']	20	AT1G30800,AT2G33240,AT1G80500,AT1G73177,AT3G62720,AT1G56700,AT1G63830,AT1G72030,AT5G60120,AT5G52390,AT1G01940,AT4G09760,AT5G11270,AT5G54770,AT4G19110,AT1G29900,AT3G01060,AT3G26380,AT2G12870,AT1G62780
GCGGTAATT	10	4	['8-12', '12-16']	20	AT1G30800,AT2G33240,AT1G80500,AT1G73177,AT3G62720,AT1G56700,AT1G63830,AT1G72030,AT5G60120,AT5G52390,AT1G01940,AT4G09760,AT5G11270,AT5G54770,AT4G19110,AT1G29900,AT3G01060,AT3G26380,AT2G12870,AT1G62780
GCGGTAATT	10	5	['8-12', '12-16']	20	AT1G30800,AT2G33240,AT1G80500,AT1G73177,AT3G62720,AT1G56700,AT1G63830,AT1G72030,AT5G60120,AT5G52390,AT1G01940,AT4G09760,AT5G11270,AT5G54770,AT4G19110,AT1G29900,AT3G01060,AT3G26380,AT2G12870,AT1G62780
GGCCAATCT	10	1	['12-16', '16-20']	16	AT3G54120,AT2G25080,AT1G20630,AT2G18940,AT3G18110,AT5G15450,AT4G35850,AT5G41650,AT5G57345,AT5G36220,AT5G09810,AT1G47330,AT5G20720,AT1G55960,AT5G25070,AT5G24970
GGCCAATCT	10	2	['12-16', '16-20']	16	AT3G54120,AT2G25080,AT1G20630,AT2G18940,AT3G18110,AT5G15450,AT4G35850,AT5G41650,AT5G57345,AT5G36220,AT5G09810,AT1G47330,AT5G20720,AT1G55960,AT5G25070,AT5G24970
GGCCAATCT	10	3	['12-16', '16-20']	16	AT3G54120,AT2G25080,AT1G20630,AT2G18940,AT3G18110,AT5G15450,AT4G35850,AT5G41650,AT5G57345,AT5G36220,AT5G09810,AT1G47330,AT5G20720,AT1G55960,AT5G25070,AT5G24970

GGCCAATCT	10	4	['12-16', '16-20']	16	AT3G54120,AT2G25080,AT1G20630,AT2G18940,AT3G18110,AT5G15450,AT4G35850,AT5G41650,AT5G57345,AT5G36220,AT5G09810,AT1G47330,AT5G20720,AT1G55960,AT5G25070,AT5G24970
GGCCAATCT	10	5	['12-16', '16-20']	16	AT3G54120,AT2G25080,AT1G20630,AT2G18940,AT3G18110,AT5G15450,AT4G35850,AT5G41650,AT5G57345,AT5G36220,AT5G09810,AT1G47330,AT5G20720,AT1G55960,AT5G25070,AT5G24970
GGCCGACTT	10	1	['0-4', '4-8', '8-12', '16-20', '20-24']	11	ATMG00650,AT2G47850,AT1G49740,AT5G50180,AT2G19750,AT5G14660,AT5G46910,AT5G34830,AT1G71720,AT1G76270,AT1G15820
GGCCGACTT	10	2	['0-4', '4-8', '8-12', '16-20', '20-24']	11	ATMG00650,AT2G47850,AT1G49740,AT5G50180,AT2G19750,AT5G14660,AT5G46910,AT5G34830,AT1G71720,AT1G76270,AT1G15820
GGCCGACTT	10	3	['0-4', '4-8', '8-12', '16-20', '20-24']	11	ATMG00650,AT2G47850,AT1G49740,AT5G50180,AT2G19750,AT5G14660,AT5G46910,AT5G34830,AT1G71720,AT1G76270,AT1G15820
GGCCGACTT	10	4	['0-4', '4-8', '8-12', '16-20', '20-24']	11	ATMG00650,AT2G47850,AT1G49740,AT5G50180,AT2G19750,AT5G14660,AT5G46910,AT5G34830,AT1G71720,AT1G76270,AT1G15820
GGCCGACTT	10	5	['0-4', '4-8', '8-12', '16-20', '20-24']	11	ATMG00650,AT2G47850,AT1G49740,AT5G50180,AT2G19750,AT5G14660,AT5G46910,AT5G34830,AT1G71720,AT1G76270,AT1G15820
GGTCCCAT	10	1	['0-4', '12-16']	28	AT1G02170,AT1G75100,AT4G39710,AT1G71450,AT2G43730,AT3G53470,AT5G49945,AT3G52840,AT2G34357,AT5G11270,AT4G27710,AT3G52180,AT5G24810,AT5G67020,AT1G66330,AT3G60380,AT3G50750,AT1G52890,AT1G19140,AT3G59480,AT3G48200,AT1G16810,AT2G21970,AT1G69780,AT2G45680,AT3G27210,AT1G54590,AT1G72140
GGTCCCAT	10	2	['0-4', '12-16']	28	AT1G02170,AT1G75100,AT4G39710,AT1G71450,AT2G43730,AT3G53470,AT5G49945,AT3G52840,AT2G34357,AT5G11270,AT4G27710,AT3G52180,AT5G24810,AT5G67020,AT1G66330,AT3G60380,AT3G50750,AT1G52890,AT1G19140,AT3G59480,AT3G48200,AT1G16810,AT2G21970,AT1G69780,AT2G45680,AT3G27210,AT1G54590,AT1G72140
GGTCCCAT	10	3	['0-4', '12-16']	28	AT1G02170,AT1G75100,AT4G39710,AT1G71450,AT2G43730,AT3G53470,AT5G49945,AT3G52840,AT2G34357,AT5G11270,AT4G27710,AT3G52180,AT5G24810,AT5G67020,AT1G66330,AT3G60380,AT3G50750,AT1G52890,AT1G19140,AT3G59480,AT3G48200,AT1G16810,AT2G21970,AT1G69780,AT2G45680,AT3G27210,AT1G54590,AT1G72140
GGTCCCAT	10	4	['0-4', '12-16']	28	AT1G02170,AT1G75100,AT4G39710,AT1G71450,AT2G43730,AT3G53470,AT5G49945,AT3G52840,AT2G34357,AT5G11270,AT4G27710,AT3G52180,AT5G24810,AT5G67020,AT1G66330,AT3G60380,AT3G50750,AT1G52890,AT1G19140,AT3G59480,AT3G48200,AT1G16810,AT2G21970,AT1G69780,AT2G45680,AT3G27210,AT1G54590,AT1G72140
GGTCCCAT	10	5	['12-16', '0-4']	28	AT1G02170,AT1G75100,AT4G39710,AT1G71450,AT2G43730,AT3G53470,AT5G49945,AT3G52840,AT2G34357,AT5G11270,AT4G27710,AT3G52180,AT5G24810,AT5G67020,AT1G66330,AT3G60380,AT3G50750,AT1G52890,AT1G19140,AT3G59480,AT3G48200,AT1G16810,AT2G21970,AT1G69780,AT2G45680,AT3G27210,AT1G54590,AT1G72140
GGWAGGGT	10	1	['12-16']	64	AT3G42100,AT5G61130,AT4G18240,AT1G34340,AT5G28280,AT1G75210,AT4G13830,AT4G39100,AT3G26660,AT5G59850,AT3G54810,AT5G19860,AT2G41120,AT3G61970,AT1G18620,AT3G61440,AT5G36170,AT3G23070,AT3G51140,AT2G37630,AT5G24490,AT1G66930,AT2G37760,AT4G10750,AT2G31010,AT3G08640,AT1G13900,AT3G20970,AT4G36470,AT1G13130,AT4G35470,AT4G25760,AT5G23420,AT1G50530,AT2G18390,AT1G01250,AT4G34830,AT4G25080,AT1G15820,AT5G45830,AT3G59190,AT3G04780,AT3G07200,AT5G63920,AT1G27930,AT5G54950,AT5G16660,AT5G63380,AT2G41870,AT1G48610,AT1G73120,AT5G16560,AT4G02480,AT4G30660,AT1G66820,AT4G19860,AT5G62200,AT3G55450,AT5G51570,AT5G52120,ATMG01180,AT1G60800,AT3G42800,AT3G63170

GGWAGGGT	10	2	['12-16']	64	AT3G42100,AT5G61130,AT4G18240,AT1G34340,AT5G28280,AT1G75210,AT4G13830,AT4G39100,AT3G26660,AT5G59850,AT3G54810,AT5G19860,AT2G41120,AT3G61970,AT1G18620,AT3G61440,AT5G36170,AT3G23070,AT3G51140,AT2G37630,AT5G24490,AT1G66930,AT2G37760,AT4G10750,AT2G31010,AT3G08640,AT1G13900,AT3G20970,AT4G36470,AT1G13130,AT4G35470,AT4G25760,AT5G23420,AT1G50530,AT2G18390,AT1G01250,AT4G34830,AT4G25080,AT1G15820,AT5G45830,AT3G59190,AT3G04780,AT3G07200,AT5G63920,AT1G27930,AT5G54950,AT5G16660,AT5G63380,AT2G41870,AT1G48610,AT1G73120,AT5G16560,AT4G02480,AT4G30660,AT1G66820,AT4G19860,AT5G62200,AT3G55450,AT5G51570,AT5G52120,ATMG01180,AT1G60800,AT3G42800,AT3G63170
GGWAGGGT	10	3	['12-16']	64	AT3G42100,AT5G61130,AT4G18240,AT1G34340,AT5G28280,AT1G75210,AT4G13830,AT4G39100,AT3G26660,AT5G59850,AT3G54810,AT5G19860,AT2G41120,AT3G61970,AT1G18620,AT3G61440,AT5G36170,AT3G23070,AT3G51140,AT2G37630,AT5G24490,AT1G66930,AT2G37760,AT4G10750,AT2G31010,AT3G08640,AT1G13900,AT3G20970,AT4G36470,AT1G13130,AT4G35470,AT4G25760,AT5G23420,AT1G50530,AT2G18390,AT1G01250,AT4G34830,AT4G25080,AT1G15820,AT5G45830,AT3G59190,AT3G04780,AT3G07200,AT5G63920,AT1G27930,AT5G54950,AT5G16660,AT5G63380,AT2G41870,AT1G48610,AT1G73120,AT5G16560,AT4G02480,AT4G30660,AT1G66820,AT4G19860,AT5G62200,AT3G55450,AT5G51570,AT5G52120,ATMG01180,AT1G60800,AT3G42800,AT3G63170
GGWAGGGT	10	4	['12-16']	64	AT3G42100,AT5G61130,AT4G18240,AT1G34340,AT5G28280,AT1G75210,AT4G13830,AT4G39100,AT3G26660,AT5G59850,AT3G54810,AT5G19860,AT2G41120,AT3G61970,AT1G18620,AT3G61440,AT5G36170,AT3G23070,AT3G51140,AT2G37630,AT5G24490,AT1G66930,AT2G37760,AT4G10750,AT2G31010,AT3G08640,AT1G13900,AT3G20970,AT4G36470,AT1G13130,AT4G35470,AT4G25760,AT5G23420,AT1G50530,AT2G18390,AT1G01250,AT4G34830,AT4G25080,AT1G15820,AT5G45830,AT3G59190,AT3G04780,AT3G07200,AT5G63920,AT1G27930,AT5G54950,AT5G16660,AT5G63380,AT2G41870,AT1G48610,AT1G73120,AT5G16560,AT4G02480,AT4G30660,AT1G66820,AT4G19860,AT5G62200,AT3G55450,AT5G51570,AT5G52120,ATMG01180,AT1G60800,AT3G42800,AT3G63170
GGWAGGGT	10	5	['12-16']	64	AT3G42100,AT5G61130,AT4G18240,AT1G34340,AT5G28280,AT1G75210,AT4G13830,AT4G39100,AT3G26660,AT5G59850,AT3G54810,AT5G19860,AT2G41120,AT3G61970,AT1G18620,AT3G61440,AT5G36170,AT3G23070,AT3G51140,AT2G37630,AT5G24490,AT1G66930,AT2G37760,AT4G10750,AT2G31010,AT3G08640,AT1G13900,AT3G20970,AT4G36470,AT1G13130,AT4G35470,AT4G25760,AT5G23420,AT1G50530,AT2G18390,AT1G01250,AT4G34830,AT4G25080,AT1G15820,AT5G45830,AT3G59190,AT3G04780,AT3G07200,AT5G63920,AT1G27930,AT5G54950,AT5G16660,AT5G63380,AT2G41870,AT1G48610,AT1G73120,AT5G16560,AT4G02480,AT4G30660,AT1G66820,AT4G19860,AT5G62200,AT3G55450,AT5G51570,AT5G52120,ATMG01180,AT1G60800,AT3G42800,AT3G63170

GTACGTG	10	1	['0-4']	184	AT2G07738,AT2G18790,AT5G49280,AT2G37830,AT5G49480,AT1G14040,AT1G16720,AT1G75830,AT5G48830,AT5G48490,AT1G66940,AT3G50950,AT1G18020,AT3G50610,AT3G50500,AT3G50750,AT1G22050,AT1G01520,AT2G14690,AT1G11680,AT4G14030,AT1G14810,AT3G48580,AT3G48940,AT1G15750,AT2G07050,AT1G35350,AT3G47860,AT1G36830,AT5G44390,AT4G16140,AT2G29670,AT1G54390,AT2G35370,AT5G59570,AT3G12610,AT4G15110,AT4G15160,AT4G14605,AT1G71030,AT1G25230,AT2G03310,AT1G03000,AT1G66840,AT3G06180,AT5G04140,AT5G41810,AT1G72140,AT1G72300,AT2G44670,AT2G38820,AT4G39950,AT1G75140,AT4G39120,AT3G31320,AT5G13090,AT4G38580,AT3G32930,AT5G20720,AT2G41080,AT5G20630,AT5G36960,AT1G73680,AT1G35460,AT4G37300,AT4G37680,AT3G29370,AT2G01860,AT4G35800,AT1G67623,AT1G70700,AT5G23350,AT1G26920,AT4G34950,AT2G21380,AT2G36885,AT1G73990,AT2G36970,AT1G69295,AT3G18260,AT4G33470,AT2G33700,AT1G72430,AT3G27210,AT3G23690,AT2G32990,AT2G03750,AT4G30720,AT1G29720,AT3G15030,AT2G32880,AT3G15580,AT1G79050,AT1G78970,AT4G29840,AT1G02160,AT5G27380,AT5G12050,AT5G11670,AT2G24880,AT1G22790,AT4G28660,AT3G26660,AT5G11500,AT1G48210,AT1G09240,AT4G27780,AT5G10510,AT5G10210,AT4G27410,AT1G12080,AT1G11850,AT5G67300,AT1G21400,AT5G67030,AT3G16910,AT5G66410,AT1G01240,AT5G66060,AT3G23210,AT1G01250,AT3G17040,AT5G65380,AT1G65560,AT5G05170,AT1G04910,AT4G24470,AT3G21250,AT5G04590,AT1G62290,AT1G26795,AT1G26780,AT1G53090,AT1G79730,AT1G79770,AT5G03150,AT1G53160,AT1G05385,AT3G13950,AT1G23205,AT5G62360,AT5G61990,AT3G16110,AT5G61570,AT3G15430,AT5G60680,AT1G24450,AT5G60210,AT3G07010,AT1G62480,AT1G32770,AT3G02750,AT2G36800,AT2G01980,AT5G57345,AT3G60380,AT2G18350,AT4G09570,AT4G09620,AT3G05940,AT3G59350,AT3G04550,AT2G46570,AT1G77920,AT3G10030,AT5G54390,AT1G74680,AT1G48350,AT3G10460,AT3G56200,AT5G53290,AT1G70820,AT3G01990,AT1G73120,AT3G08870,AT3G08890,AT2G06630,AT5G52180,AT5G51720,AT4G01690,AT2G35750,AT3G54900,AT2G42490,AT3G54240
GTACGTG	10	2	['0-4']	184	AT2G07738,AT2G18790,AT5G49280,AT2G37830,AT5G49480,AT1G14040,AT1G16720,AT1G75830,AT5G48830,AT5G48490,AT1G66940,AT3G50950,AT1G18020,AT3G50610,AT3G50500,AT3G50750,AT1G22050,AT1G01520,AT2G14690,AT1G11680,AT4G14030,AT1G14810,AT3G48580,AT3G48940,AT1G15750,AT2G07050,AT1G35350,AT3G47860,AT1G36830,AT5G44390,AT4G16140,AT2G29670,AT1G54390,AT2G35370,AT5G59570,AT3G12610,AT4G15110,AT4G15160,AT4G14605,AT1G71030,AT1G25230,AT2G03310,AT1G03000,AT1G66840,AT3G06180,AT5G04140,AT5G41810,AT1G72140,AT1G72300,AT2G44670,AT2G38820,AT4G39950,AT1G75140,AT4G39120,AT3G31320,AT5G13090,AT4G38580,AT3G32930,AT5G20720,AT2G41080,AT5G20630,AT5G36960,AT1G73680,AT1G35460,AT4G37300,AT4G37680,AT3G29370,AT2G01860,AT4G35800,AT1G67623,AT1G70700,AT5G23350,AT1G26920,AT4G34950,AT2G21380,AT2G36885,AT1G73990,AT2G36970,AT1G69295,AT3G18260,AT4G33470,AT2G33700,AT1G72430,AT3G27210,AT3G23690,AT2G32990,AT2G03750,AT4G30720,AT1G29720,AT3G15030,AT2G32880,AT3G15580,AT1G79050,AT1G78970,AT4G29840,AT1G02160,AT5G27380,AT5G12050,AT5G11670,AT2G24880,AT1G22790,AT4G28660,AT3G26660,AT5G11500,AT1G48210,AT1G09240,AT4G27780,AT5G10510,AT5G10210,AT4G27410,AT1G12080,AT1G11850,AT5G67300,AT1G21400,AT5G67030,AT3G16910,AT5G66410,AT1G01240,AT5G66060,AT3G23210,AT1G01250,AT3G17040,AT5G65380,AT1G65560,AT5G05170,AT1G04910,AT4G24470,AT3G21250,AT5G04590,AT1G62290,AT1G26795,AT1G26780,AT1G53090,AT1G79730,AT1G79770,AT5G03150,AT1G53160,AT1G05385,AT3G13950,AT1G23205,AT5G62360,AT5G61990,AT3G16110,AT5G61570,AT3G15430,AT5G60680,AT1G24450,AT5G60210,AT3G07010,AT1G62480,AT1G32770,AT3G02750,AT2G36800,AT2G01980,AT5G57345,AT3G60380,AT2G18350,AT4G09570,AT4G09620,AT3G05940,AT3G59350,AT3G04550,AT2G46570,AT1G77920,AT3G10030,AT5G54390,AT1G74680,AT1G48350,AT3G10460,AT3G56200,AT5G53290,AT1G70820,AT3G01990,AT1G73120,AT3G08870,AT3G08890,AT2G06630,AT5G52180,AT5G51720,AT4G01690,AT2G35750,AT3G54900,AT2G42490,AT3G54240

GTACGTG	10	3	['0-4']	184	AT2G07738,AT2G18790,AT5G49280,AT2G37830,AT5G49480,AT1G14040,AT1G16720,AT1G75830,AT5G48830,AT5G48490,AT1G66940,AT3G50950,AT1G18020,AT3G50610,AT3G50500,AT3G50750,AT1G22050,AT1G01520,AT2G14690,AT1G11680,AT4G14030,AT1G14810,AT3G48580,AT3G48940,AT1G15750,AT2G07050,AT1G35350,AT3G47860,AT1G36830,AT5G44390,AT4G16140,AT2G29670,AT1G54390,AT2G35370,AT5G59570,AT3G12610,AT4G15110,AT4G15160,AT4G14605,AT1G71030,AT1G25230,AT2G03310,AT1G03000,AT1G66840,AT3G06180,AT5G04140,AT5G41810,AT1G72140,AT1G72300,AT2G44670,AT2G38820,AT4G39950,AT1G75140,AT4G39120,AT3G31320,AT5G13090,AT4G38580,AT3G32930,AT5G20720,AT2G41080,AT5G20630,AT5G36960,AT1G73680,AT1G35460,AT4G37300,AT4G37680,AT3G29370,AT2G01860,AT4G35800,AT1G67623,AT1G70700,AT5G23350,AT1G26920,AT4G34950,AT2G21380,AT2G36885,AT1G73990,AT2G36970,AT1G69295,AT3G18260,AT4G33470,AT2G33700,AT1G72430,AT3G27210,AT3G23690,AT2G32990,AT2G03750,AT4G30720,AT1G29720,AT3G15030,AT2G32880,AT3G15580,AT1G79050,AT1G78970,AT4G29840,AT1G02160,AT5G27380,AT5G12050,AT5G11670,AT2G24880,AT1G22790,AT4G28660,AT3G26660,AT5G11500,AT1G48210,AT1G09240,AT4G27780,AT5G10510,AT5G10210,AT4G27410,AT1G12080,AT1G11850,AT5G67300,AT1G21400,AT5G67030,AT3G16910,AT5G66410,AT1G01240,AT5G66060,AT3G23210,AT1G01250,AT3G17040,AT5G65380,AT1G65560,AT5G05170,AT1G04910,AT4G24470,AT3G21250,AT5G04590,AT1G62290,AT1G26795,AT1G26780,AT1G53090,AT1G79730,AT1G79770,AT5G03150,AT1G53160,AT1G05385,AT3G13950,AT1G23205,AT5G62360,AT5G61990,AT3G16110,AT5G61570,AT3G15430,AT5G60680,AT1G24450,AT5G60210,AT3G07010,AT1G62480,AT1G32770,AT3G02750,AT2G36800,AT2G01980,AT5G57345,AT3G60380,AT2G18350,AT4G09570,AT4G09620,AT3G05940,AT3G59350,AT3G04550,AT2G46570,AT1G77920,AT3G10030,AT5G54390,AT1G74680,AT1G48350,AT3G10460,AT3G56200,AT5G53290,AT1G70820,AT3G01990,AT1G73120,AT3G08870,AT3G08890,AT2G06630,AT5G52180,AT5G51720,AT4G01690,AT2G35750,AT3G54900,AT2G42490,AT3G54240
GTACGTG	10	4	['0-4']	184	AT2G07738,AT2G18790,AT5G49280,AT2G37830,AT5G49480,AT1G14040,AT1G16720,AT1G75830,AT5G48830,AT5G48490,AT1G66940,AT3G50950,AT1G18020,AT3G50610,AT3G50500,AT3G50750,AT1G22050,AT1G01520,AT2G14690,AT1G11680,AT4G14030,AT1G14810,AT3G48580,AT3G48940,AT1G15750,AT2G07050,AT1G35350,AT3G47860,AT1G36830,AT5G44390,AT4G16140,AT2G29670,AT1G54390,AT2G35370,AT5G59570,AT3G12610,AT4G15110,AT4G15160,AT4G14605,AT1G71030,AT1G25230,AT2G03310,AT1G03000,AT1G66840,AT3G06180,AT5G04140,AT5G41810,AT1G72140,AT1G72300,AT2G44670,AT2G38820,AT4G39950,AT1G75140,AT4G39120,AT3G31320,AT5G13090,AT4G38580,AT3G32930,AT5G20720,AT2G41080,AT5G20630,AT5G36960,AT1G73680,AT1G35460,AT4G37300,AT4G37680,AT3G29370,AT2G01860,AT4G35800,AT1G67623,AT1G70700,AT5G23350,AT1G26920,AT4G34950,AT2G21380,AT2G36885,AT1G73990,AT2G36970,AT1G69295,AT3G18260,AT4G33470,AT2G33700,AT1G72430,AT3G27210,AT3G23690,AT2G32990,AT2G03750,AT4G30720,AT1G29720,AT3G15030,AT2G32880,AT3G15580,AT1G79050,AT1G78970,AT4G29840,AT1G02160,AT5G27380,AT5G12050,AT5G11670,AT2G24880,AT1G22790,AT4G28660,AT3G26660,AT5G11500,AT1G48210,AT1G09240,AT4G27780,AT5G10510,AT5G10210,AT4G27410,AT1G12080,AT1G11850,AT5G67300,AT1G21400,AT5G67030,AT3G16910,AT5G66410,AT1G01240,AT5G66060,AT3G23210,AT1G01250,AT3G17040,AT5G65380,AT1G65560,AT5G05170,AT1G04910,AT4G24470,AT3G21250,AT5G04590,AT1G62290,AT1G26795,AT1G26780,AT1G53090,AT1G79730,AT1G79770,AT5G03150,AT1G53160,AT1G05385,AT3G13950,AT1G23205,AT5G62360,AT5G61990,AT3G16110,AT5G61570,AT3G15430,AT5G60680,AT1G24450,AT5G60210,AT3G07010,AT1G62480,AT1G32770,AT3G02750,AT2G36800,AT2G01980,AT5G57345,AT3G60380,AT2G18350,AT4G09570,AT4G09620,AT3G05940,AT3G59350,AT3G04550,AT2G46570,AT1G77920,AT3G10030,AT5G54390,AT1G74680,AT1G48350,AT3G10460,AT3G56200,AT5G53290,AT1G70820,AT3G01990,AT1G73120,AT3G08870,AT3G08890,AT2G06630,AT5G52180,AT5G51720,AT4G01690,AT2G35750,AT3G54900,AT2G42490,AT3G54240

GTACGTG	10	5	['0-4']	184	AT2G07738,AT2G18790,AT5G49280,AT2G37830,AT5G49480,AT1G14040,AT1G16720,AT1G75830,AT5G48830,AT5G48490,AT1G66940,AT3G50950,AT1G18020,AT3G50610,AT3G50500,AT3G50750,AT1G22050,AT1G01520,AT2G14690,AT1G11680,AT4G14030,AT1G14810,AT3G48580,AT3G48940,AT1G15750,AT2G07050,AT1G35350,AT3G47860,AT1G36830,AT5G44390,AT4G16140,AT2G29670,AT1G54390,AT2G35370,AT5G59570,AT3G12610,AT4G15110,AT4G15160,AT4G14605,AT1G71030,AT1G25230,AT2G03310,AT1G03000,AT1G66840,AT3G06180,AT5G04140,AT5G41810,AT1G72140,AT1G72300,AT2G44670,AT2G38820,AT4G39950,AT1G75140,AT4G39120,AT3G31320,AT5G13090,AT4G38580,AT3G32930,AT5G20720,AT2G41080,AT5G20630,AT5G36960,AT1G73680,AT1G35460,AT4G37300,AT4G37680,AT3G29370,AT2G01860,AT4G35800,AT1G67623,AT1G70700,AT5G23350,AT1G26920,AT4G34950,AT2G21380,AT2G36885,AT1G73990,AT2G36970,AT1G69295,AT3G18260,AT4G33470,AT2G33700,AT1G72430,AT3G27210,AT3G23690,AT2G32990,AT2G03750,AT4G30720,AT1G29720,AT3G15030,AT2G32880,AT3G15580,AT1G79050,AT1G78970,AT4G29840,AT1G02160,AT5G27380,AT5G12050,AT5G11670,AT2G24880,AT1G22790,AT4G28660,AT3G26660,AT5G11500,AT1G48210,AT1G09240,AT4G27780,AT5G10510,AT5G10210,AT4G27410,AT1G12080,AT1G11850,AT5G67300,AT1G21400,AT5G67030,AT3G16910,AT5G66410,AT1G01240,AT5G66060,AT3G23210,AT1G01250,AT3G17040,AT5G65380,AT1G65560,AT5G05170,AT1G04910,AT4G24470,AT3G21250,AT5G04590,AT1G62290,AT1G26795,AT1G26780,AT1G53090,AT1G79730,AT1G79770,AT5G03150,AT1G53160,AT1G05385,AT3G13950,AT1G23205,AT5G62360,AT5G61990,AT3G16110,AT5G61570,AT3G15430,AT5G60680,AT1G24450,AT5G60210,AT3G07010,AT1G62480,AT1G32770,AT3G02750,AT2G36800,AT2G01980,AT5G57345,AT3G60380,AT2G18350,AT4G09570,AT4G09620,AT3G05940,AT3G59350,AT3G04550,AT2G46570,AT1G77920,AT3G10030,AT5G54390,AT1G74680,AT1G48350,AT3G10460,AT3G56200,AT5G53290,AT1G70820,AT3G01990,AT1G73120,AT3G08870,AT3G08890,AT2G06630,AT5G52180,AT5G51720,AT4G01690,AT2G35750,AT3G54900,AT2G42490,AT3G54240
GTGATCAC	10	1	['0-4', '8-12', '12-16', '16-20']	58	AT5G61130,AT4G39990,AT3G62720,AT3G26280,AT3G06510,AT1G04170,AT3G07680,AT3G32280,AT2G33480,AT3G05180,AT5G24930,AT1G16470,AT3G07880,AT2G28840,AT3G16320,AT1G05960,AT2G29310,AT5G66880,AT2G01860,AT1G70660,AT2G20260,AT1G01620,AT3G60020,AT1G11680,AT1G10070,AT5G46630,AT3G05880,AT1G74640,AT3G56880,AT2G47910,AT1G80090,AT1G13540,AT5G45300,AT2G06925,AT5G05170,AT1G21250,AT1G70000,AT3G16200,AT1G12845,AT5G54540,AT5G17440,AT1G75690,AT1G42970,AT4G23060,AT1G26760,AT4G22540,AT2G42790,AT5G03050,AT3G15040,AT4G03050,AT1G23210,AT1G47530,AT1G52190,AT1G73390,AT3G62250,AT5G41050,AT5G41060,AT1G76990
GTGATCAC	10	2	['0-4', '8-12', '12-16', '16-20']	58	AT5G61130,AT4G39990,AT3G62720,AT3G26280,AT3G06510,AT1G04170,AT3G07680,AT3G32280,AT2G33480,AT3G05180,AT5G24930,AT1G16470,AT3G07880,AT2G28840,AT3G16320,AT1G05960,AT2G29310,AT5G66880,AT2G01860,AT1G70660,AT2G20260,AT1G01620,AT3G60020,AT1G11680,AT1G10070,AT5G46630,AT3G05880,AT1G74640,AT3G56880,AT2G47910,AT1G80090,AT1G13540,AT5G45300,AT2G06925,AT5G05170,AT1G21250,AT1G70000,AT3G16200,AT1G12845,AT5G54540,AT5G17440,AT1G75690,AT1G42970,AT4G23060,AT1G26760,AT4G22540,AT2G42790,AT5G03050,AT3G15040,AT4G03050,AT1G23210,AT1G47530,AT1G52190,AT1G73390,AT3G62250,AT5G41050,AT5G41060,AT1G76990
GTGATCAC	10	3	['0-4', '8-12', '12-16', '16-20']	58	AT5G61130,AT4G39990,AT3G62720,AT3G26280,AT3G06510,AT1G04170,AT3G07680,AT3G32280,AT2G33480,AT3G05180,AT5G24930,AT1G16470,AT3G07880,AT2G28840,AT3G16320,AT1G05960,AT2G29310,AT5G66880,AT2G01860,AT1G70660,AT2G20260,AT1G01620,AT3G60020,AT1G11680,AT1G10070,AT5G46630,AT3G05880,AT1G74640,AT3G56880,AT2G47910,AT1G80090,AT1G13540,AT5G45300,AT2G06925,AT5G05170,AT1G21250,AT1G70000,AT3G16200,AT1G12845,AT5G54540,AT5G17440,AT1G75690,AT1G42970,AT4G23060,AT1G26760,AT4G22540,AT2G42790,AT5G03050,AT3G15040,AT4G03050,AT1G23210,AT1G47530,AT1G52190,AT1G73390,AT3G62250,AT5G41050,AT5G41060,AT1G76990
GTGATCAC	10	4	['0-4', '8-12', '12-16', '16-20']	58	AT5G61130,AT4G39990,AT3G62720,AT3G26280,AT3G06510,AT1G04170,AT3G07680,AT3G32280,AT2G33480,AT3G05180,AT5G24930,AT1G16470,AT3G07880,AT2G28840,AT3G16320,AT1G05960,AT2G29310,AT5G66880,AT2G01860,AT1G70660,AT2G20260,AT1G01620,AT3G60020,AT1G11680,AT1G10070,AT5G46630,AT3G05880,AT1G74640,AT3G56880,AT2G47910,AT1G80090,AT1G13540,AT5G45300,AT2G06925,AT5G05170,AT1G21250,AT1G70000,AT3G16200,AT1G12845,AT5G54540,AT5G17440,AT1G75690,AT1G42970,AT4G23060,AT1G26760,AT4G22540,AT2G42790,AT5G03050,AT3G15040,AT4G03050,AT1G23210,AT1G47530,AT1G52190,AT1G73390,AT3G62250,AT5G41050,AT5G41060,AT1G76990

GTGATCAC	10	5	['0-4', '8-12', '12-16', '16-20']	58	AT5G61130,AT4G39990,AT3G62720,AT3G26280,AT3G06510,AT1G04170,AT3G07680,AT3G32280,AT2G33480,AT3G05180,AT5G24930,AT1G16470,AT3G07880,AT2G28840,AT3G16320,AT1G05960,AT2G29310,AT5G66880,AT2G01860,AT1G70660,AT2G20260,AT1G01620,AT3G60020,AT1G11680,AT1G10070,AT5G46630,AT3G05880,AT1G74640,AT3G56880,AT2G47910,AT1G80090,AT1G13540,AT5G45300,AT2G06925,AT5G05170,AT1G21250,AT1G70000,AT3G16200,AT1G12845,AT5G54540,AT5G17440,AT1G75690,AT1G42970,AT4G23060,AT1G26760,AT4G22540,AT2G42790,AT5G03050,AT3G15040,AT4G03050,AT1G23210,AT1G47530,AT1G52190,AT1G73390,AT3G62250,AT5G41050,AT5G41060,AT1G76990
GTGCCCTT	10	1	['0-4', '16-20']	20	AT1G21460,AT2G42580,AT5G16610,AT2G38820,AT2G43010,AT1G80310,AT3G60860,AT4G35850,AT1G50020,AT1G67110,AT4G02420,AT1G73390,AT4G38420,AT1G70730,AT3G10050,AT5G01750,AT1G74680,AT1G47310,AT1G35150,AT1G03250
GTGCCCTT	10	2	['0-4', '16-20']	20	AT1G21460,AT2G42580,AT5G16610,AT2G38820,AT2G43010,AT1G80310,AT3G60860,AT4G35850,AT1G50020,AT1G67110,AT4G02420,AT1G73390,AT4G38420,AT1G70730,AT3G10050,AT5G01750,AT1G74680,AT1G47310,AT1G35150,AT1G03250
GTGCCCTT	10	3	['0-4', '16-20']	20	AT1G21460,AT2G42580,AT5G16610,AT2G38820,AT2G43010,AT1G80310,AT3G60860,AT4G35850,AT1G50020,AT1G67110,AT4G02420,AT1G73390,AT4G38420,AT1G70730,AT3G10050,AT5G01750,AT1G74680,AT1G47310,AT1G35150,AT1G03250
GTGCCCTT	10	4	['0-4', '16-20']	20	AT1G21460,AT2G42580,AT5G16610,AT2G38820,AT2G43010,AT1G80310,AT3G60860,AT4G35850,AT1G50020,AT1G67110,AT4G02420,AT1G73390,AT4G38420,AT1G70730,AT3G10050,AT5G01750,AT1G74680,AT1G47310,AT1G35150,AT1G03250
GTGCCCTT	10	5	['16-20', '0-4']	20	AT1G21460,AT2G42580,AT5G16610,AT2G38820,AT2G43010,AT1G80310,AT3G60860,AT4G35850,AT1G50020,AT1G67110,AT4G02420,AT1G73390,AT4G38420,AT1G70730,AT3G10050,AT5G01750,AT1G74680,AT1G47310,AT1G35150,AT1G03250
GTNWAYATTNATNNG	10	1	['4-8', '8-12', '12-16', '16-20']	16	AT5G50840,AT1G03090,AT2G17900,AT1G78480,AT4G26520,AT1G35290,AT5G50012,AT1G10760,AT5G63880,AT2G17220,AT3G56000,AT3G03420,AT3G59920,AT5G43670,AT5G54390,AT5G61850
GTNWAYATTNATNNG	10	2	['4-8', '8-12', '12-16', '16-20']	16	AT5G50840,AT1G03090,AT2G17900,AT1G78480,AT4G26520,AT1G35290,AT5G50012,AT1G10760,AT5G63880,AT2G17220,AT3G56000,AT3G03420,AT3G59920,AT5G43670,AT5G54390,AT5G61850
GTNWAYATTNATNNG	10	3	['4-8', '8-12', '12-16', '16-20']	16	AT5G50840,AT1G03090,AT2G17900,AT1G78480,AT4G26520,AT1G35290,AT5G50012,AT1G10760,AT5G63880,AT2G17220,AT3G56000,AT3G03420,AT3G59920,AT5G43670,AT5G54390,AT5G61850
GTNWAYATTNATNNG	10	4	['4-8', '8-12', '12-16', '16-20']	16	AT5G50840,AT1G03090,AT2G17900,AT1G78480,AT4G26520,AT1G35290,AT5G50012,AT1G10760,AT5G63880,AT2G17220,AT3G56000,AT3G03420,AT3G59920,AT5G43670,AT5G54390,AT5G61850
GTNWAYATTNATNNG	10	5	['4-8', '8-12', '12-16', '16-20']	16	AT5G50840,AT1G03090,AT2G17900,AT1G78480,AT4G26520,AT1G35290,AT5G50012,AT1G10760,AT5G63880,AT2G17220,AT3G56000,AT3G03420,AT3G59920,AT5G43670,AT5G54390,AT5G61850
GTTAGGTTC	10	1	['8-12', '12-16', '16-20']	14	AT3G42100,AT2G03870,AT3G14080,AT5G55220,AT5G59830,AT3G47590,AT4G28080,AT1G52220,AT3G47130,AT1G06820,AT1G14810,AT3G14415,AT5G05740,AT3G43020
GTTAGGTTC	10	2	['8-12', '12-16', '16-20']	14	AT3G42100,AT2G03870,AT3G14080,AT5G55220,AT5G59830,AT3G47590,AT4G28080,AT1G52220,AT3G47130,AT1G06820,AT1G14810,AT3G14415,AT5G05740,AT3G43020
GTTAGGTTC	10	3	['8-12', '12-16', '16-20']	14	AT3G42100,AT2G03870,AT3G14080,AT5G55220,AT5G59830,AT3G47590,AT4G28080,AT1G52220,AT3G47130,AT1G06820,AT1G14810,AT3G14415,AT5G05740,AT3G43020
GTTAGGTTC	10	4	['8-12', '12-16', '16-20']	14	AT3G42100,AT2G03870,AT3G14080,AT5G55220,AT5G59830,AT3G47590,AT4G28080,AT1G52220,AT3G47130,AT1G06820,AT1G14810,AT3G14415,AT5G05740,AT3G43020
GTTAGGTTC	10	5	['8-12', '12-16', '16-20']	14	AT3G42100,AT2G03870,AT3G14080,AT5G55220,AT5G59830,AT3G47590,AT4G28080,AT1G52220,AT3G47130,AT1G06820,AT1G14810,AT3G14415,AT5G05740,AT3G43020
KGTCCCAT	10	1	['0-4', '4-8']	70	AT1G02170,AT3G54120,AT1G75100,AT4G39710,AT1G71450,AT2G43730,AT3G53470,AT5G49945,AT2G40780,AT3G52840,AT5G60210,AT5G19540,AT2G34357,AT5G11270,AT1G62620,AT5G49450,AT1G75830,AT4G27710,AT3G52180,AT5G24810,AT4G12500,AT2G42590,AT5G58730,AT2G25080,AT5G58270,AT4G26520,AT1G66330,AT5G67020,AT3G50750,AT3G60380,AT3G20970,AT1G52890,AT4G35470,AT1G09415,AT1G31812,AT1G19140,AT3G23940,AT3G59480,AT3G59350,AT1G64860,AT3G48200,AT4G34220,AT1G16810,AT5G55380,AT2G21970,AT3G15310,AT3G19680,AT1G69780,AT5G64040,AT2G35940,AT3G12350,AT2G45680,AT3G27210,AT3G12600,AT5G42810,AT1G36940,AT1G54590,AT5G54310,AT2G27360,AT5G53370,AT3G56340,AT3G09390,AT2G43840,AT3G55450,AT5G62280,AT3G11050,AT1G72140,AT4G30500,AT3G54810,AT1G32550

KGTCCCAT	10	2	['0-4', '4-8']	70	AT1G02170,AT3G54120,AT1G75100,AT4G39710,AT1G71450,AT2G43730,AT3G53470,AT5G49945,AT2G40780,AT3G52840,AT5G60210,AT5G19540,AT2G34357,AT5G11270,AT1G62620,AT5G49450,AT1G75830,AT4G27710,AT3G52180,AT5G24810,AT4G12500,AT2G42590,AT5G58730,AT2G25080,AT5G58270,AT4G26520,AT1G66330,AT5G67020,AT3G50750,AT3G60380,AT3G20970,AT1G52890,AT4G35470,AT1G09415,AT1G31812,AT1G19140,AT3G23940,AT3G59480,AT3G59350,AT1G64860,AT3G48200,AT4G34220,AT1G16810,AT5G55380,AT2G21970,AT3G15310,AT3G19680,AT1G69780,AT5G64040,AT2G35940,AT3G12350,AT2G45680,AT3G27210,AT3G12600,AT5G42810,AT1G36940,AT1G54590,AT5G54310,AT2G27360,AT5G53370,AT3G56340,AT3G09390,AT2G43840,AT3G55450,AT5G62280,AT3G11050,AT1G72140,AT4G30500,AT3G54810,AT1G32550
KGTCCCAT	10	3	['0-4', '4-8']	70	AT1G02170,AT3G54120,AT1G75100,AT4G39710,AT1G71450,AT2G43730,AT3G53470,AT5G49945,AT2G40780,AT3G52840,AT5G60210,AT5G19540,AT2G34357,AT5G11270,AT1G62620,AT5G49450,AT1G75830,AT4G27710,AT3G52180,AT5G24810,AT4G12500,AT2G42590,AT5G58730,AT2G25080,AT5G58270,AT4G26520,AT1G66330,AT5G67020,AT3G50750,AT3G60380,AT3G20970,AT1G52890,AT4G35470,AT1G09415,AT1G31812,AT1G19140,AT3G23940,AT3G59480,AT3G59350,AT1G64860,AT3G48200,AT4G34220,AT1G16810,AT5G55380,AT2G21970,AT3G15310,AT3G19680,AT1G69780,AT5G64040,AT2G35940,AT3G12350,AT2G45680,AT3G27210,AT3G12600,AT5G42810,AT1G36940,AT1G54590,AT5G54310,AT2G27360,AT5G53370,AT3G56340,AT3G09390,AT2G43840,AT3G55450,AT5G62280,AT3G11050,AT1G72140,AT4G30500,AT3G54810,AT1G32550
KGTCCCAT	10	4	['0-4', '4-8']	70	AT1G02170,AT3G54120,AT1G75100,AT4G39710,AT1G71450,AT2G43730,AT3G53470,AT5G49945,AT2G40780,AT3G52840,AT5G60210,AT5G19540,AT2G34357,AT5G11270,AT1G62620,AT5G49450,AT1G75830,AT4G27710,AT3G52180,AT5G24810,AT4G12500,AT2G42590,AT5G58730,AT2G25080,AT5G58270,AT4G26520,AT1G66330,AT5G67020,AT3G50750,AT3G60380,AT3G20970,AT1G52890,AT4G35470,AT1G09415,AT1G31812,AT1G19140,AT3G23940,AT3G59480,AT3G59350,AT1G64860,AT3G48200,AT4G34220,AT1G16810,AT5G55380,AT2G21970,AT3G15310,AT3G19680,AT1G69780,AT5G64040,AT2G35940,AT3G12350,AT2G45680,AT3G27210,AT3G12600,AT5G42810,AT1G36940,AT1G54590,AT5G54310,AT2G27360,AT5G53370,AT3G56340,AT3G09390,AT2G43840,AT3G55450,AT5G62280,AT3G11050,AT1G72140,AT4G30500,AT3G54810,AT1G32550
KGTCCCAT	10	5	['4-8', '0-4']	70	AT1G02170,AT3G54120,AT1G75100,AT4G39710,AT1G71450,AT2G43730,AT3G53470,AT5G49945,AT2G40780,AT3G52840,AT5G60210,AT5G19540,AT2G34357,AT5G11270,AT1G62620,AT5G49450,AT1G75830,AT4G27710,AT3G52180,AT5G24810,AT4G12500,AT2G42590,AT5G58730,AT2G25080,AT5G58270,AT4G26520,AT1G66330,AT5G67020,AT3G50750,AT3G60380,AT3G20970,AT1G52890,AT4G35470,AT1G09415,AT1G31812,AT1G19140,AT3G23940,AT3G59480,AT3G59350,AT1G64860,AT3G48200,AT4G34220,AT1G16810,AT5G55380,AT2G21970,AT3G15310,AT3G19680,AT1G69780,AT5G64040,AT2G35940,AT3G12350,AT2G45680,AT3G27210,AT3G12600,AT5G42810,AT1G36940,AT1G54590,AT5G54310,AT2G27360,AT5G53370,AT3G56340,AT3G09390,AT2G43840,AT3G55450,AT5G62280,AT3G11050,AT1G72140,AT4G30500,AT3G54810,AT1G32550
MAGGTAAGT	10	1	['4-8', '8-12', '20-24']	56	AT3G63060,AT4G00960,AT1G53670,AT5G11500,AT3G62700,AT5G26820,AT3G52610,AT1G03970,AT4G28100,AT1G32060,AT4G27330,AT3G52380,AT2G02280,AT2G33420,AT5G08100,AT5G08030,AT3G16320,AT2G37540,AT3G08580,AT1G35950,AT3G42570,AT3G22530,AT5G65430,AT1G80440,AT4G17640,AT2G46570,AT2G36880,AT1G09930,AT2G46530,AT5G56090,AT5G04810,AT3G15290,AT1G75710,AT2G05630,AT2G22030,AT5G03795,AT4G04930,AT3G57170,AT4G04910,AT3G46610,AT4G22300,AT3G56660,AT1G29390,AT1G73120,AT3G55680,AT4G19910,AT5G42100,AT5G52450,AT5G52420,AT5G14180,AT5G01890,AT3G05000,AT2G44770,AT4G29700,AT2G26080,AT3G63520
MAGGTAAGT	10	2	['4-8', '8-12', '20-24']	56	AT3G63060,AT4G00960,AT1G53670,AT5G11500,AT3G62700,AT5G26820,AT3G52610,AT1G03970,AT4G28100,AT1G32060,AT4G27330,AT3G52380,AT2G02280,AT2G33420,AT5G08100,AT5G08030,AT3G16320,AT2G37540,AT3G08580,AT1G35950,AT3G42570,AT3G22530,AT5G65430,AT1G80440,AT4G17640,AT2G46570,AT2G36880,AT1G09930,AT2G46530,AT5G56090,AT5G04810,AT3G15290,AT1G75710,AT2G05630,AT2G22030,AT5G03795,AT4G04930,AT3G57170,AT4G04910,AT3G46610,AT4G22300,AT3G56660,AT1G29390,AT1G73120,AT3G55680,AT4G19910,AT5G42100,AT5G52450,AT5G52420,AT5G14180,AT5G01890,AT3G05000,AT2G44770,AT4G29700,AT2G26080,AT3G63520

MAGGTAAGT	10	3	['4-8', '8-12', '20-24']	56	AT3G63060,AT4G00960,AT1G53670,AT5G11500,AT3G62700,AT5G26820,AT3G52610,AT1G03970,AT4G28100,AT1G32060,AT4G27330,AT3G52380,AT2G02280,AT2G33420,AT5G08100,AT5G08030,AT3G16320,AT2G37540,AT3G08580,AT1G35950,AT3G42570,AT3G22530,AT5G65430,AT1G80440,AT4G17640,AT2G46570,AT2G36880,AT1G09930,AT2G46530,AT5G56090,AT5G04810,AT3G15290,AT1G75710,AT2G05630,AT2G22030,AT5G03795,AT4G04930,AT3G57170,AT4G04910,AT3G46610,AT4G22300,AT3G56660,AT1G29390,AT1G73120,AT3G55680,AT4G19910,AT5G42100,AT5G52450,AT5G52420,AT5G14180,AT5G01890,AT3G05000,AT2G44770,AT4G29700,AT2G26080,AT3G63520
MAGGTAAGT	10	4	['4-8', '8-12', '20-24']	56	AT3G63060,AT4G00960,AT1G53670,AT5G11500,AT3G62700,AT5G26820,AT3G52610,AT1G03970,AT4G28100,AT1G32060,AT4G27330,AT3G52380,AT2G02280,AT2G33420,AT5G08100,AT5G08030,AT3G16320,AT2G37540,AT3G08580,AT1G35950,AT3G42570,AT3G22530,AT5G65430,AT1G80440,AT4G17640,AT2G46570,AT2G36880,AT1G09930,AT2G46530,AT5G56090,AT5G04810,AT3G15290,AT1G75710,AT2G05630,AT2G22030,AT5G03795,AT4G04930,AT3G57170,AT4G04910,AT3G46610,AT4G22300,AT3G56660,AT1G29390,AT1G73120,AT3G55680,AT4G19910,AT5G42100,AT5G52450,AT5G52420,AT5G14180,AT5G01890,AT3G05000,AT2G44770,AT4G29700,AT2G26080,AT3G63520
MAGGTAAGT	10	5	['4-8', '8-12', '20-24']	56	AT3G63060,AT4G00960,AT1G53670,AT5G11500,AT3G62700,AT5G26820,AT3G52610,AT1G03970,AT4G28100,AT1G32060,AT4G27330,AT3G52380,AT2G02280,AT2G33420,AT5G08100,AT5G08030,AT3G16320,AT2G37540,AT3G08580,AT1G35950,AT3G42570,AT3G22530,AT5G65430,AT1G80440,AT4G17640,AT2G46570,AT2G36880,AT1G09930,AT2G46530,AT5G56090,AT5G04810,AT3G15290,AT1G75710,AT2G05630,AT2G22030,AT5G03795,AT4G04930,AT3G57170,AT4G04910,AT3G46610,AT4G22300,AT3G56660,AT1G29390,AT1G73120,AT3G55680,AT4G19910,AT5G42100,AT5G52450,AT5G52420,AT5G14180,AT5G01890,AT3G05000,AT2G44770,AT4G29700,AT2G26080,AT3G63520
MCACGTGGC	10	1	['4-8']	80	AT4G28750,AT5G39570,AT5G27520,AT1G54850,AT3G63060,AT4G18240,AT3G63210,AT1G32470,AT2G34460,AT3G06780,AT1G18310,AT1G55480,AT1G06680,AT5G59080,AT2G38000,AT5G49120,AT5G35460,AT5G67370,AT5G58070,AT1G28530,AT4G25570,AT4G35850,AT5G57760,AT5G47640,AT5G57345,AT5G66570,AT1G01520,AT1G01240,AT4G25450,AT3G25530,AT3G60200,AT5G23050,AT5G65630,AT1G55670,AT4G09620,AT2G21330,AT3G59660,AT4G16515,AT5G05270,AT5G05300,AT1G19660,AT3G59220,AT5G05200,AT2G47780,AT5G64840,AT4G08180,AT4G24190,AT5G64260,AT1G09870,AT2G15970,AT5G64180,AT2G04550,AT3G15210,AT1G22850,AT1G74840,AT5G18340,AT3G03150,AT2G36390,AT1G56220,AT5G43850,AT3G12470,AT4G03560,AT1G10960,AT1G53090,AT4G14270,AT2G47400,AT3G56050,AT4G31310,AT3G13980,AT2G01290,AT4G19710,AT5G52580,AT1G18740,AT1G52220,AT1G52230,AT1G55850,AT1G68440,AT3G04860,AT2G45990,AT5G61530
MCACGTGGC	10	2	['4-8']	80	AT4G28750,AT5G39570,AT5G27520,AT1G54850,AT3G63060,AT4G18240,AT3G63210,AT1G32470,AT2G34460,AT3G06780,AT1G18310,AT1G55480,AT1G06680,AT5G59080,AT2G38000,AT5G49120,AT5G35460,AT5G67370,AT5G58070,AT1G28530,AT4G25570,AT4G35850,AT5G57760,AT5G47640,AT5G57345,AT5G66570,AT1G01520,AT1G01240,AT4G25450,AT3G25530,AT3G60200,AT5G23050,AT5G65630,AT1G55670,AT4G09620,AT2G21330,AT3G59660,AT4G16515,AT5G05270,AT5G05300,AT1G19660,AT3G59220,AT5G05200,AT2G47780,AT5G64840,AT4G08180,AT4G24190,AT5G64260,AT1G09870,AT2G15970,AT5G64180,AT2G04550,AT3G15210,AT1G22850,AT1G74840,AT5G18340,AT3G03150,AT2G36390,AT1G56220,AT5G43850,AT3G12470,AT4G03560,AT1G10960,AT1G53090,AT4G14270,AT2G47400,AT3G56050,AT4G31310,AT3G13980,AT2G01290,AT4G19710,AT5G52580,AT1G18740,AT1G52220,AT1G52230,AT1G55850,AT1G68440,AT3G04860,AT2G45990,AT5G61530
MCACGTGGC	10	3	['4-8']	80	AT4G28750,AT5G39570,AT5G27520,AT1G54850,AT3G63060,AT4G18240,AT3G63210,AT1G32470,AT2G34460,AT3G06780,AT1G18310,AT1G55480,AT1G06680,AT5G59080,AT2G38000,AT5G49120,AT5G35460,AT5G67370,AT5G58070,AT1G28530,AT4G25570,AT4G35850,AT5G57760,AT5G47640,AT5G57345,AT5G66570,AT1G01520,AT1G01240,AT4G25450,AT3G25530,AT3G60200,AT5G23050,AT5G65630,AT1G55670,AT4G09620,AT2G21330,AT3G59660,AT4G16515,AT5G05270,AT5G05300,AT1G19660,AT3G59220,AT5G05200,AT2G47780,AT5G64840,AT4G08180,AT4G24190,AT5G64260,AT1G09870,AT2G15970,AT5G64180,AT2G04550,AT3G15210,AT1G22850,AT1G74840,AT5G18340,AT3G03150,AT2G36390,AT1G56220,AT5G43850,AT3G12470,AT4G03560,AT1G10960,AT1G53090,AT4G14270,AT2G47400,AT3G56050,AT4G31310,AT3G13980,AT2G01290,AT4G19710,AT5G52580,AT1G18740,AT1G52220,AT1G52230,AT1G55850,AT1G68440,AT3G04860,AT2G45990,AT5G61530

MCACGTGGC	10	4	['4-8']	80	AT4G28750,AT5G39570,AT5G27520,AT1G54850,AT3G63060,AT4G18240,AT3G63210,AT1G32470,AT2G34460,AT3G06780,AT1G18310,AT1G55480,AT1G06680,AT5G59080,AT2G38000,AT5G49120,AT5G35460,AT5G67370,AT5G58070,AT1G28530,AT4G25570,AT4G35850,AT5G57760,AT5G47640,AT5G57345,AT5G66570,AT1G01520,AT1G01240,AT4G25450,AT3G25530,AT3G60200,AT5G23050,AT5G65630,AT1G55670,AT4G09620,AT2G21330,AT3G59660,AT4G16515,AT5G05270,AT5G05300,AT1G19660,AT3G59220,AT5G05200,AT2G47780,AT5G64840,AT4G08180,AT4G24190,AT5G64260,AT1G09870,AT2G15970,AT5G64180,AT2G04550,AT3G15210,AT1G22850,AT1G74840,AT5G18340,AT3G03150,AT2G36390,AT1G56220,AT5G43850,AT3G12470,AT4G03560,AT1G10960,AT1G53090,AT4G14270,AT2G47400,AT3G56050,AT4G31310,AT3G13980,AT2G01290,AT4G19710,AT5G52580,AT1G18740,AT1G52220,AT1G52230,AT1G55850,AT1G68440,AT3G04860,AT2G45990,AT5G61530
MCACGTGGC	10	5	['4-8']	80	AT4G28750,AT5G39570,AT5G27520,AT1G54850,AT3G63060,AT4G18240,AT3G63210,AT1G32470,AT2G34460,AT3G06780,AT1G18310,AT1G55480,AT1G06680,AT5G59080,AT2G38000,AT5G49120,AT5G35460,AT5G67370,AT5G58070,AT1G28530,AT4G25570,AT4G35850,AT5G57760,AT5G47640,AT5G57345,AT5G66570,AT1G01520,AT1G01240,AT4G25450,AT3G25530,AT3G60200,AT5G23050,AT5G65630,AT1G55670,AT4G09620,AT2G21330,AT3G59660,AT4G16515,AT5G05270,AT5G05300,AT1G19660,AT3G59220,AT5G05200,AT2G47780,AT5G64840,AT4G08180,AT4G24190,AT5G64260,AT1G09870,AT2G15970,AT5G64180,AT2G04550,AT3G15210,AT1G22850,AT1G74840,AT5G18340,AT3G03150,AT2G36390,AT1G56220,AT5G43850,AT3G12470,AT4G03560,AT1G10960,AT1G53090,AT4G14270,AT2G47400,AT3G56050,AT4G31310,AT3G13980,AT2G01290,AT4G19710,AT5G52580,AT1G18740,AT1G52220,AT1G52230,AT1G55850,AT1G68440,AT3G04860,AT2G45990,AT5G61530
NCCCGCCA	10	1	['8-12', '12-16', '16-20']	68	AT3G05480,AT4G39990,AT1G75210,AT1G26220,AT1G24450,AT5G11500,AT5G60120,AT2G38400,AT5G20720,AT5G58760,AT2G41430,AT2G31790,AT1G62780,AT5G35840,AT5G24890,AT3G23070,AT5G58730,AT5G58620,AT4G14700,AT3G02930,AT3G60320,AT1G53450,AT2G22720,AT4G16570,AT4G09900,AT1G58290,AT5G66060,AT1G17145,AT3G26380,AT5G23140,AT1G15820,AT3G04880,AT3G56910,AT3G59190,AT3G59400,AT1G80530,AT1G19650,AT1G08890,AT4G24340,AT4G17760,AT2G35260,AT4G23940,AT4G05180,AT1G12845,AT1G76920,AT5G16150,AT2G16070,AT3G18210,AT3G10690,AT5G15770,AT3G17930,AT3G12530,AT5G42640,AT1G03550,AT1G78450,AT5G03150,AT5G14610,AT3G01850,AT1G72060,AT3G55760,AT2G46820,AT3G07390,AT3G43790,AT5G40500,AT1G65430,AT1G17160,AT1G76360,AT5G61530
NCCCGCCA	10	2	['8-12', '12-16', '16-20']	68	AT3G05480,AT4G39990,AT1G75210,AT1G26220,AT1G24450,AT5G11500,AT5G60120,AT2G38400,AT5G20720,AT5G58760,AT2G41430,AT2G31790,AT1G62780,AT5G35840,AT5G24890,AT3G23070,AT5G58730,AT5G58620,AT4G14700,AT3G02930,AT3G60320,AT1G53450,AT2G22720,AT4G16570,AT4G09900,AT1G58290,AT5G66060,AT1G17145,AT3G26380,AT5G23140,AT1G15820,AT3G04880,AT3G56910,AT3G59190,AT3G59400,AT1G80530,AT1G19650,AT1G08890,AT4G24340,AT4G17760,AT2G35260,AT4G23940,AT4G05180,AT1G12845,AT1G76920,AT5G16150,AT2G16070,AT3G18210,AT3G10690,AT5G15770,AT3G17930,AT3G12530,AT5G42640,AT1G03550,AT1G78450,AT5G03150,AT5G14610,AT3G01850,AT1G72060,AT3G55760,AT2G46820,AT3G07390,AT3G43790,AT5G40500,AT1G65430,AT1G17160,AT1G76360,AT5G61530
NCCCGCCA	10	3	['8-12', '12-16', '16-20']	68	AT3G05480,AT4G39990,AT1G75210,AT1G26220,AT1G24450,AT5G11500,AT5G60120,AT2G38400,AT5G20720,AT5G58760,AT2G41430,AT2G31790,AT1G62780,AT5G35840,AT5G24890,AT3G23070,AT5G58730,AT5G58620,AT4G14700,AT3G02930,AT3G60320,AT1G53450,AT2G22720,AT4G16570,AT4G09900,AT1G58290,AT5G66060,AT1G17145,AT3G26380,AT5G23140,AT1G15820,AT3G04880,AT3G56910,AT3G59190,AT3G59400,AT1G80530,AT1G19650,AT1G08890,AT4G24340,AT4G17760,AT2G35260,AT4G23940,AT4G05180,AT1G12845,AT1G76920,AT5G16150,AT2G16070,AT3G18210,AT3G10690,AT5G15770,AT3G17930,AT3G12530,AT5G42640,AT1G03550,AT1G78450,AT5G03150,AT5G14610,AT3G01850,AT1G72060,AT3G55760,AT2G46820,AT3G07390,AT3G43790,AT5G40500,AT1G65430,AT1G17160,AT1G76360,AT5G61530
NCCCGCCA	10	4	['8-12', '12-16', '16-20']	68	AT3G05480,AT4G39990,AT1G75210,AT1G26220,AT1G24450,AT5G11500,AT5G60120,AT2G38400,AT5G20720,AT5G58760,AT2G41430,AT2G31790,AT1G62780,AT5G35840,AT5G24890,AT3G23070,AT5G58730,AT5G58620,AT4G14700,AT3G02930,AT3G60320,AT1G53450,AT2G22720,AT4G16570,AT4G09900,AT1G58290,AT5G66060,AT1G17145,AT3G26380,AT5G23140,AT1G15820,AT3G04880,AT3G56910,AT3G59190,AT3G59400,AT1G80530,AT1G19650,AT1G08890,AT4G24340,AT4G17760,AT2G35260,AT4G23940,AT4G05180,AT1G12845,AT1G76920,AT5G16150,AT2G16070,AT3G18210,AT3G10690,AT5G15770,AT3G17930,AT3G12530,AT5G42640,AT1G03550,AT1G78450,AT5G03150,AT5G14610,AT3G01850,AT1G72060,AT3G55760,AT2G46820,AT3G07390,AT3G43790,AT5G40500,AT1G65430,AT1G17160,AT1G76360,AT5G61530

NCCCGCCA	10	5	['8-12', '12-16', '16-20']	68	AT3G05480,AT4G39990,AT1G75210,AT1G26220,AT1G24450,AT5G11500,AT5G60120,AT2G38400,AT5G20720,AT5G58760,AT2G41430,AT2G31790,AT1G62780,AT5G35840,AT5G24890,AT3G23070,AT5G58730,AT5G58620,AT4G14700,AT3G02930,AT3G60320,AT1G53450,AT2G22720,AT4G16570,AT4G09900,AT1G58290,AT5G66060,AT1G17145,AT3G26380,AT5G23140,AT1G15820,AT3G04880,AT3G56910,AT3G59190,AT3G59400,AT1G80530,AT1G19650,AT1G08890,AT4G24340,AT4G17760,AT2G35260,AT4G23940,AT4G05180,AT1G12845,AT1G76920,AT5G16150,AT2G16070,AT3G18210,AT3G10690,AT5G15770,AT3G17930,AT3G12530,AT5G42640,AT1G03550,AT1G78450,AT5G03150,AT5G14610,AT3G01850,AT1G72060,AT3G55760,AT2G46820,AT3G07390,AT3G43790,AT5G40500,AT1G65430,AT1G17160,AT1G76360,AT5G61530
NNWNCCAWWWWTRGWWAN	10	1	['0-4', '8-12', '12-16', '20-24']	36	AT3G15450,AT2G19480,AT5G39570,AT2G13360,AT1G33270,AT4G00370,AT1G18350,AT5G26030,AT2G17220,AT2G25180,AT5G24910,AT5G08580,AT5G67370,AT5G08110,AT2G25210,AT3G08650,AT2G32390,AT4G36600,AT1G65900,AT3G48580,AT3G26380,AT4G14430,AT1G16570,AT3G14080,AT3G09970,AT2G18170,AT2G17880,AT4G21430,AT1G27630,AT3G17840,AT5G02810,AT1G29670,AT5G62540,AT2G22450,AT3G44630,AT1G32360
NNWNCCAWWWWTRGWWAN	10	2	['0-4', '8-12', '12-16', '20-24']	36	AT3G15450,AT2G19480,AT5G39570,AT2G13360,AT1G33270,AT4G00370,AT1G18350,AT5G26030,AT2G17220,AT2G25180,AT5G24910,AT5G08580,AT5G67370,AT5G08110,AT2G25210,AT3G08650,AT2G32390,AT4G36600,AT1G65900,AT3G48580,AT3G26380,AT4G14430,AT1G16570,AT3G14080,AT3G09970,AT2G18170,AT2G17880,AT4G21430,AT1G27630,AT3G17840,AT5G02810,AT1G29670,AT5G62540,AT2G22450,AT3G44630,AT1G32360
NNWNCCAWWWWTRGWWAN	10	3	['0-4', '8-12', '12-16', '20-24']	36	AT3G15450,AT2G19480,AT5G39570,AT2G13360,AT1G33270,AT4G00370,AT1G18350,AT5G26030,AT2G17220,AT2G25180,AT5G24910,AT5G08580,AT5G67370,AT5G08110,AT2G25210,AT3G08650,AT2G32390,AT4G36600,AT1G65900,AT3G48580,AT3G26380,AT4G14430,AT1G16570,AT3G14080,AT3G09970,AT2G18170,AT2G17880,AT4G21430,AT1G27630,AT3G17840,AT5G02810,AT1G29670,AT5G62540,AT2G22450,AT3G44630,AT1G32360
NNWNCCAWWWWTRGWWAN	10	4	['0-4', '8-12', '12-16', '20-24']	36	AT3G15450,AT2G19480,AT5G39570,AT2G13360,AT1G33270,AT4G00370,AT1G18350,AT5G26030,AT2G17220,AT2G25180,AT5G24910,AT5G08580,AT5G67370,AT5G08110,AT2G25210,AT3G08650,AT2G32390,AT4G36600,AT1G65900,AT3G48580,AT3G26380,AT4G14430,AT1G16570,AT3G14080,AT3G09970,AT2G18170,AT2G17880,AT4G21430,AT1G27630,AT3G17840,AT5G02810,AT1G29670,AT5G62540,AT2G22450,AT3G44630,AT1G32360
NNWNCCAWWWWTRGWWAN	10	5	['0-4', '8-12', '12-16', '20-24']	36	AT3G15450,AT2G19480,AT5G39570,AT2G13360,AT1G33270,AT4G00370,AT1G18350,AT5G26030,AT2G17220,AT2G25180,AT5G24910,AT5G08580,AT5G67370,AT5G08110,AT2G25210,AT3G08650,AT2G32390,AT4G36600,AT1G65900,AT3G48580,AT3G26380,AT4G14430,AT1G16570,AT3G14080,AT3G09970,AT2G18170,AT2G17880,AT4G21430,AT1G27630,AT3G17840,AT5G02810,AT1G29670,AT5G62540,AT2G22450,AT3G44630,AT1G32360
NTTDCCWWWWNNGGWAAN	10	1	['4-8', '12-16', '16-20', '20-24']	11	AT5G27150,AT5G03140,AT4G14965,AT1G33270,AT4G02480,AT1G74920,AT1G09390,AT5G23760,AT4G14960,AT5G65910,AT2G02710
NTTDCCWWWWNNGGWAAN	10	2	['4-8', '12-16', '16-20', '20-24']	11	AT5G27150,AT5G03140,AT4G14965,AT1G33270,AT4G02480,AT1G74920,AT1G09390,AT5G23760,AT4G14960,AT5G65910,AT2G02710
NTTDCCWWWWNNGGWAAN	10	3	['4-8', '12-16', '20-24', '16-20']	11	AT5G27150,AT5G03140,AT4G14965,AT1G33270,AT4G02480,AT1G74920,AT1G09390,AT5G23760,AT4G14960,AT5G65910,AT2G02710
NTTDCCWWWWNNGGWAAN	10	4	['4-8', '12-16', '16-20', '20-24']	11	AT5G27150,AT5G03140,AT4G14965,AT1G33270,AT4G02480,AT1G74920,AT1G09390,AT5G23760,AT4G14960,AT5G65910,AT2G02710
NTTDCCWWWWNNGGWAAN	10	5	['4-8', '12-16', '16-20', '20-24']	11	AT5G27150,AT5G03140,AT4G14965,AT1G33270,AT4G02480,AT1G74920,AT1G09390,AT5G23760,AT4G14960,AT5G65910,AT2G02710

RGTGACNNNGC	10	1	['12-16']	58	AT1G01700,AT1G22750,AT1G75140,AT3G53680,AT5G11500,AT2G40780,AT3G54500,AT5G19760,AT5G49540,AT3G53030,AT3G20300,AT3G61780,AT2G37830,AT1G62620,AT1G04140,AT4G13010,AT2G31820,AT1G43670,AT1G76030,AT5G58730,AT1G73830,AT1G21460,AT5G67200,AT3G05000,AT1G28530,AT5G67020,AT1G44478,AT5G57290,AT5G47870,AT1G10070,AT5G23000,AT1G07140,AT1G07280,AT5G65840,AT3G56910,AT2G06980,AT4G34250,AT1G73990,AT4G16710,AT5G17990,AT3G30810,AT5G17660,AT3G10670,AT3G10690,AT1G71030,AT2G41760,AT1G79730,AT1G73020,AT1G29670,AT5G15510,AT4G31040,AT3G13950,AT5G52180,AT3G27690,AT3G03320,AT3G10970,AT1G71695,AT1G65430
RGTGACNNNGC	10	2	['12-16']	58	AT1G01700,AT1G22750,AT1G75140,AT3G53680,AT5G11500,AT2G40780,AT3G54500,AT5G19760,AT5G49540,AT3G53030,AT3G20300,AT3G61780,AT2G37830,AT1G62620,AT1G04140,AT4G13010,AT2G31820,AT1G43670,AT1G76030,AT5G58730,AT1G73830,AT1G21460,AT5G67200,AT3G05000,AT1G28530,AT5G67020,AT1G44478,AT5G57290,AT5G47870,AT1G10070,AT5G23000,AT1G07140,AT1G07280,AT5G65840,AT3G56910,AT2G06980,AT4G34250,AT1G73990,AT4G16710,AT5G17990,AT3G30810,AT5G17660,AT3G10670,AT3G10690,AT1G71030,AT2G41760,AT1G79730,AT1G73020,AT1G29670,AT5G15510,AT4G31040,AT3G13950,AT5G52180,AT3G27690,AT3G03320,AT3G10970,AT1G71695,AT1G65430
RGTGACNNNGC	10	3	['12-16']	58	AT1G01700,AT1G22750,AT1G75140,AT3G53680,AT5G11500,AT2G40780,AT3G54500,AT5G19760,AT5G49540,AT3G53030,AT3G20300,AT3G61780,AT2G37830,AT1G62620,AT1G04140,AT4G13010,AT2G31820,AT1G43670,AT1G76030,AT5G58730,AT1G73830,AT1G21460,AT5G67200,AT3G05000,AT1G28530,AT5G67020,AT1G44478,AT5G57290,AT5G47870,AT1G10070,AT5G23000,AT1G07140,AT1G07280,AT5G65840,AT3G56910,AT2G06980,AT4G34250,AT1G73990,AT4G16710,AT5G17990,AT3G30810,AT5G17660,AT3G10670,AT3G10690,AT1G71030,AT2G41760,AT1G79730,AT1G73020,AT1G29670,AT5G15510,AT4G31040,AT3G13950,AT5G52180,AT3G27690,AT3G03320,AT3G10970,AT1G71695,AT1G65430
RGTGACNNNGC	10	4	['12-16']	58	AT1G01700,AT1G22750,AT1G75140,AT3G53680,AT5G11500,AT2G40780,AT3G54500,AT5G19760,AT5G49540,AT3G53030,AT3G20300,AT3G61780,AT2G37830,AT1G62620,AT1G04140,AT4G13010,AT2G31820,AT1G43670,AT1G76030,AT5G58730,AT1G73830,AT1G21460,AT5G67200,AT3G05000,AT1G28530,AT5G67020,AT1G44478,AT5G57290,AT5G47870,AT1G10070,AT5G23000,AT1G07140,AT1G07280,AT5G65840,AT3G56910,AT2G06980,AT4G34250,AT1G73990,AT4G16710,AT5G17990,AT3G30810,AT5G17660,AT3G10670,AT3G10690,AT1G71030,AT2G41760,AT1G79730,AT1G73020,AT1G29670,AT5G15510,AT4G31040,AT3G13950,AT5G52180,AT3G27690,AT3G03320,AT3G10970,AT1G71695,AT1G65430
RGTGACNNNGC	10	5	['12-16']	58	AT1G01700,AT1G22750,AT1G75140,AT3G53680,AT5G11500,AT2G40780,AT3G54500,AT5G19760,AT5G49540,AT3G53030,AT3G20300,AT3G61780,AT2G37830,AT1G62620,AT1G04140,AT4G13010,AT2G31820,AT1G43670,AT1G76030,AT5G58730,AT1G73830,AT1G21460,AT5G67200,AT3G05000,AT1G28530,AT5G67020,AT1G44478,AT5G57290,AT5G47870,AT1G10070,AT5G23000,AT1G07140,AT1G07280,AT5G65840,AT3G56910,AT2G06980,AT4G34250,AT1G73990,AT4G16710,AT5G17990,AT3G30810,AT5G17660,AT3G10670,AT3G10690,AT1G71030,AT2G41760,AT1G79730,AT1G73020,AT1G29670,AT5G15510,AT4G31040,AT3G13950,AT5G52180,AT3G27690,AT3G03320,AT3G10970,AT1G71695,AT1G65430
RTGAGTCAT	10	1	['0-4', '16-20', '8-12']	43	AT1G02170,AT1G34260,AT3G54050,AT1G01770,AT1G01700,AT4G28660,AT5G38980,AT3G02570,AT2G28720,AT3G02468,AT2G18770,AT2G41040,AT5G09870,AT5G58730,AT1G05030,AT3G51140,AT1G28600,AT5G07440,AT3G60320,AT5G57040,AT5G65910,AT3G60080,AT1G18990,AT1G22530,AT3G59140,AT1G20693,AT2G07050,AT5G54270,AT1G77570,AT5G43430,AT1G60970,AT1G78480,AT5G29000,AT1G69530,AT5G62540,AT3G02340,AT2G03750,AT5G02240,AT5G62360,AT1G52200,AT3G16110,AT1G21670,AT5G61590
RTGAGTCAT	10	2	['0-4', '8-12', '16-20']	43	AT1G02170,AT1G34260,AT3G54050,AT1G01770,AT1G01700,AT4G28660,AT5G38980,AT3G02570,AT2G28720,AT3G02468,AT2G18770,AT2G41040,AT5G09870,AT5G58730,AT1G05030,AT3G51140,AT1G28600,AT5G07440,AT3G60320,AT5G57040,AT5G65910,AT3G60080,AT1G18990,AT1G22530,AT3G59140,AT1G20693,AT2G07050,AT5G54270,AT1G77570,AT5G43430,AT1G60970,AT1G78480,AT5G29000,AT1G69530,AT5G62540,AT3G02340,AT2G03750,AT5G02240,AT5G62360,AT1G52200,AT3G16110,AT1G21670,AT5G61590

RTGAGTCAT	10	3	['0-4', '8-12', '16-20']	43	AT1G02170,AT1G34260,AT3G54050,AT1G01770,AT1G01700,AT4G28660,AT5G38980,AT3G02570,AT2G28720,AT3G02468,AT2G18770,AT2G41040,AT5G09870,AT5G58730,AT1G05030,AT3G51140,AT1G28600,AT5G07440,AT3G60320,AT5G57040,AT5G65910,AT3G60080,AT1G18990,AT1G22530,AT3G59140,AT1G20693,AT2G07050,AT5G54270,AT1G77570,AT5G43430,AT1G60970,AT1G78480,AT5G29000,AT1G69530,AT5G62540,AT3G02340,AT2G03750,AT5G02240,AT5G62360,AT1G52200,AT3G16110,AT1G21670,AT5G61590
RTGAGTCAT	10	4	['0-4', '16-20', '8-12']	43	AT1G02170,AT1G34260,AT3G54050,AT1G01770,AT1G01700,AT4G28660,AT5G38980,AT3G02570,AT2G28720,AT3G02468,AT2G18770,AT2G41040,AT5G09870,AT5G58730,AT1G05030,AT3G51140,AT1G28600,AT5G07440,AT3G60320,AT5G57040,AT5G65910,AT3G60080,AT1G18990,AT1G22530,AT3G59140,AT1G20693,AT2G07050,AT5G54270,AT1G77570,AT5G43430,AT1G60970,AT1G78480,AT5G29000,AT1G69530,AT5G62540,AT3G02340,AT2G03750,AT5G02240,AT5G62360,AT1G52200,AT3G16110,AT1G21670,AT5G61590
RTGAGTCAT	10	5	['8-12', '16-20', '0-4']	43	AT1G02170,AT1G34260,AT3G54050,AT1G01770,AT1G01700,AT4G28660,AT5G38980,AT3G02570,AT2G28720,AT3G02468,AT2G18770,AT2G41040,AT5G09870,AT5G58730,AT1G05030,AT3G51140,AT1G28600,AT5G07440,AT3G60320,AT5G57040,AT5G65910,AT3G60080,AT1G18990,AT1G22530,AT3G59140,AT1G20693,AT2G07050,AT5G54270,AT1G77570,AT5G43430,AT1G60970,AT1G78480,AT5G29000,AT1G69530,AT5G62540,AT3G02340,AT2G03750,AT5G02240,AT5G62360,AT1G52200,AT3G16110,AT1G21670,AT5G61590
TAACTCGTT	10	1	['0-4', '4-8', '8-12', '16-20']	32	AT3G02700,AT1G70330,AT3G07770,AT2G31750,AT5G58730,AT1G73660,AT1G76240,AT5G48100,AT3G26710,AT2G18290,AT1G01230,AT2G23690,AT4G15140,AT5G45310,AT3G51820,AT5G04940,AT5G05200,AT3G56700,AT1G09960,AT1G63830,AT5G17660,AT1G69830,AT4G03560,AT4G20280,AT5G15510,AT3G15095,AT5G41810,AT3G15850,AT1G35150,AT1G21660,AT4G01610,AT1G30270
TAACTCGTT	10	2	['0-4', '4-8', '8-12', '16-20']	32	AT3G02700,AT1G70330,AT3G07770,AT2G31750,AT5G58730,AT1G73660,AT1G76240,AT5G48100,AT3G26710,AT2G18290,AT1G01230,AT2G23690,AT4G15140,AT5G45310,AT3G51820,AT5G04940,AT5G05200,AT3G56700,AT1G09960,AT1G63830,AT5G17660,AT1G69830,AT4G03560,AT4G20280,AT5G15510,AT3G15095,AT5G41810,AT3G15850,AT1G35150,AT1G21660,AT4G01610,AT1G30270
TAACTCGTT	10	3	['0-4', '4-8', '8-12', '16-20']	32	AT3G02700,AT1G70330,AT3G07770,AT2G31750,AT5G58730,AT1G73660,AT1G76240,AT5G48100,AT3G26710,AT2G18290,AT1G01230,AT2G23690,AT4G15140,AT5G45310,AT3G51820,AT5G04940,AT5G05200,AT3G56700,AT1G09960,AT1G63830,AT5G17660,AT1G69830,AT4G03560,AT4G20280,AT5G15510,AT3G15095,AT5G41810,AT3G15850,AT1G35150,AT1G21660,AT4G01610,AT1G30270
TAACTCGTT	10	4	['0-4', '4-8', '8-12', '16-20']	32	AT3G02700,AT1G70330,AT3G07770,AT2G31750,AT5G58730,AT1G73660,AT1G76240,AT5G48100,AT3G26710,AT2G18290,AT1G01230,AT2G23690,AT4G15140,AT5G45310,AT3G51820,AT5G04940,AT5G05200,AT3G56700,AT1G09960,AT1G63830,AT5G17660,AT1G69830,AT4G03560,AT4G20280,AT5G15510,AT3G15095,AT5G41810,AT3G15850,AT1G35150,AT1G21660,AT4G01610,AT1G30270
TAACTCGTT	10	5	['0-4', '4-8', '8-12', '16-20']	32	AT3G02700,AT1G70330,AT3G07770,AT2G31750,AT5G58730,AT1G73660,AT1G76240,AT5G48100,AT3G26710,AT2G18290,AT1G01230,AT2G23690,AT4G15140,AT5G45310,AT3G51820,AT5G04940,AT5G05200,AT3G56700,AT1G09960,AT1G63830,AT5G17660,AT1G69830,AT4G03560,AT4G20280,AT5G15510,AT3G15095,AT5G41810,AT3G15850,AT1G35150,AT1G21660,AT4G01610,AT1G30270
TAACTGGTT	10	1	['12-16']	55	AT4G39960,AT1G19300,AT5G27280,AT5G28290,AT1G33270,AT5G26820,AT1G06470,AT3G52720,AT2G18770,AT5G10510,AT5G37960,AT3G30840,AT3G52450,AT1G04170,AT3G28340,AT5G48540,AT1G29240,AT5G35630,AT5G57760,AT4G17230,AT1G13195,AT4G14960,AT2G18410,AT5G23210,AT1G26920,AT1G19110,AT1G22490,AT5G65430,AT5G56260,AT5G56460,AT3G28700,AT2G36870,AT1G09795,AT4G05180,AT5G03905,AT2G44550,AT3G47560,AT1G30130,AT3G06380,AT5G54510,AT4G32340,AT5G16660,AT3G18110,AT5G03320,AT5G42940,AT2G17860,AT1G03090,AT1G24764,AT3G45690,AT3G45230,AT1G18740,AT1G05570,AT1G79040,AT3G26520,AT4G19390

TAACTGGTT	10	2	['12-16']	55	AT4G39960,AT1G19300,AT5G27280,AT5G28290,AT1G33270,AT5G26820,AT1G06470,AT3G52720,AT2G18770,AT5G10510,AT5G37960,AT3G30840,AT3G52450,AT1G04170,AT3G28340,AT5G48540,AT1G29240,AT5G35630,AT5G57760,AT4G17230,AT1G13195,AT4G14960,AT2G18410,AT5G23210,AT1G26920,AT1G19110,AT1G22490,AT5G65430,AT5G56260,AT5G56460,AT3G28700,AT2G36870,AT1G09795,AT4G05180,AT5G03905,AT2G44550,AT3G47560,AT1G30130,AT3G06380,AT5G54510,AT4G32340,AT5G16660,AT3G18110,AT5G03320,AT5G42940,AT2G17860,AT1G03090,AT1G24764,AT3G45690,AT3G45230,AT1G18740,AT1G05570,AT1G79040,AT3G26520,AT4G19390
TAACTGGTT	10	3	['12-16']	55	AT4G39960,AT1G19300,AT5G27280,AT5G28290,AT1G33270,AT5G26820,AT1G06470,AT3G52720,AT2G18770,AT5G10510,AT5G37960,AT3G30840,AT3G52450,AT1G04170,AT3G28340,AT5G48540,AT1G29240,AT5G35630,AT5G57760,AT4G17230,AT1G13195,AT4G14960,AT2G18410,AT5G23210,AT1G26920,AT1G19110,AT1G22490,AT5G65430,AT5G56260,AT5G56460,AT3G28700,AT2G36870,AT1G09795,AT4G05180,AT5G03905,AT2G44550,AT3G47560,AT1G30130,AT3G06380,AT5G54510,AT4G32340,AT5G16660,AT3G18110,AT5G03320,AT5G42940,AT2G17860,AT1G03090,AT1G24764,AT3G45690,AT3G45230,AT1G18740,AT1G05570,AT1G79040,AT3G26520,AT4G19390
TAACTGGTT	10	4	['12-16']	55	AT4G39960,AT1G19300,AT5G27280,AT5G28290,AT1G33270,AT5G26820,AT1G06470,AT3G52720,AT2G18770,AT5G10510,AT5G37960,AT3G30840,AT3G52450,AT1G04170,AT3G28340,AT5G48540,AT1G29240,AT5G35630,AT5G57760,AT4G17230,AT1G13195,AT4G14960,AT2G18410,AT5G23210,AT1G26920,AT1G19110,AT1G22490,AT5G65430,AT5G56260,AT5G56460,AT3G28700,AT2G36870,AT1G09795,AT4G05180,AT5G03905,AT2G44550,AT3G47560,AT1G30130,AT3G06380,AT5G54510,AT4G32340,AT5G16660,AT3G18110,AT5G03320,AT5G42940,AT2G17860,AT1G03090,AT1G24764,AT3G45690,AT3G45230,AT1G18740,AT1G05570,AT1G79040,AT3G26520,AT4G19390
TAACTGGTT	10	5	['12-16']	55	AT4G39960,AT1G19300,AT5G27280,AT5G28290,AT1G33270,AT5G26820,AT1G06470,AT3G52720,AT2G18770,AT5G10510,AT5G37960,AT3G30840,AT3G52450,AT1G04170,AT3G28340,AT5G48540,AT1G29240,AT5G35630,AT5G57760,AT4G17230,AT1G13195,AT4G14960,AT2G18410,AT5G23210,AT1G26920,AT1G19110,AT1G22490,AT5G65430,AT5G56260,AT5G56460,AT3G28700,AT2G36870,AT1G09795,AT4G05180,AT5G03905,AT2G44550,AT3G47560,AT1G30130,AT3G06380,AT5G54510,AT4G32340,AT5G16660,AT3G18110,AT5G03320,AT5G42940,AT2G17860,AT1G03090,AT1G24764,AT3G45690,AT3G45230,AT1G18740,AT1G05570,AT1G79040,AT3G26520,AT4G19390
TAATCATTA	10	1	['4-8', '16-20']	108	ATCG00650,AT3G10230,AT2G26520,AT3G53190,AT5G50012,AT5G49910,AT1G62750,AT3G01060,AT1G66940,AT3G50610,AT3G50500,AT2G32390,AT3G50240,AT2G41410,AT3G49590,AT3G48530,AT5G45830,AT1G20620,AT5G44580,AT1G54520,AT4G15530,AT3G46510,AT3G12110,AT1G78480,AT1G76790,AT2G34580,AT5G39210,AT5G13090,AT5G08410,AT3G52380,AT3G52340,AT2G42600,AT3G30380,AT2G42580,AT4G37680,AT1G66410,AT5G20840,AT2G30900,AT4G37020,AT4G36040,AT5G23730,AT1G70700,AT4G36600,AT4G35090,AT2G21540,AT2G46450,AT2G46340,AT2G40080,AT3G19100,AT2G44360,AT3G28180,AT5G17600,AT2G36430,AT5G15860,AT5G15510,AT4G30610,AT3G17430,AT1G53280,AT4G28610,AT5G27140,AT5G25900,AT5G10510,AT5G24890,AT5G67300,AT5G67390,AT5G08000,AT3G12980,AT1G10200,AT5G66710,AT5G06720,AT3G17130,AT1G09380,AT5G66530,AT3G17100,AT5G65990,AT1G01430,AT3G23280,AT4G23750,AT4G23200,AT4G19670,AT1G79610,AT1G24320,AT5G01750,AT3G23325,AT1G60490,AT1G49750,AT4G17880,AT5G59540,AT1G18350,AT3G61430,AT1G08130,AT1G10650,AT1G50530,AT3G11900,AT1G22530,AT5G56520,AT3G59020,AT2G16600,AT1G35580,AT5G56020,AT2G25510,AT5G54110,AT1G27600,AT4G03030,AT3G09250,AT2G01680,AT3G54680,AT4G01130

TAATCATTA	10	2	['4-8', '16-20']	108	ATCG00650,AT3G10230,AT2G26520,AT3G53190,AT5G50012,AT5G49910,AT1G62750,AT3G01060,AT1G66940,AT3G50610,AT3G50500,AT2G32390,AT3G50240,AT2G41410,AT3G49590,AT3G48530,AT5G45830,AT1G20620,AT5G44580,AT1G54520,AT4G15530,AT3G46510,AT3G12110,AT1G78480,AT1G76790,AT2G34580,AT5G39210,AT5G13090,AT5G08410,AT3G52380,AT3G52340,AT2G42600,AT3G30380,AT2G42580,AT4G37680,AT1G66410,AT5G20840,AT2G30900,AT4G37020,AT4G36040,AT5G23730,AT1G70700,AT4G36600,AT4G35090,AT2G21540,AT2G46450,AT2G46340,AT2G40080,AT3G19100,AT2G44360,AT3G28180,AT5G17600,AT2G36430,AT5G15860,AT5G15510,AT4G30610,AT3G17430,AT1G53280,AT4G28610,AT5G27140,AT5G25900,AT5G10510,AT5G24890,AT5G67300,AT5G67390,AT5G08000,AT3G12980,AT1G10200,AT5G66710,AT5G06720,AT3G17130,AT1G09380,AT5G66530,AT3G17100,AT5G65990,AT1G01430,AT3G23280,AT4G23750,AT4G23200,AT4G19670,AT1G79610,AT1G24320,AT5G01750,AT3G23325,AT1G60490,AT1G49750,AT4G17880,AT5G59540,AT1G18350,AT3G61430,AT1G08130,AT1G10650,AT1G50530,AT3G11900,AT1G22530,AT5G56520,AT3G59020,AT2G16600,AT1G35580,AT5G56020,AT2G25510,AT5G54110,AT1G27600,AT4G03030,AT3G09250,AT2G01680,AT3G54680,AT4G01130
TAATCATTA	10	3	['4-8', '16-20']	108	ATCG00650,AT3G10230,AT2G26520,AT3G53190,AT5G50012,AT5G49910,AT1G62750,AT3G01060,AT1G66940,AT3G50610,AT3G50500,AT2G32390,AT3G50240,AT2G41410,AT3G49590,AT3G48530,AT5G45830,AT1G20620,AT5G44580,AT1G54520,AT4G15530,AT3G46510,AT3G12110,AT1G78480,AT1G76790,AT2G34580,AT5G39210,AT5G13090,AT5G08410,AT3G52380,AT3G52340,AT2G42600,AT3G30380,AT2G42580,AT4G37680,AT1G66410,AT5G20840,AT2G30900,AT4G37020,AT4G36040,AT5G23730,AT1G70700,AT4G36600,AT4G35090,AT2G21540,AT2G46450,AT2G46340,AT2G40080,AT3G19100,AT2G44360,AT3G28180,AT5G17600,AT2G36430,AT5G15860,AT5G15510,AT4G30610,AT3G17430,AT1G53280,AT4G28610,AT5G27140,AT5G25900,AT5G10510,AT5G24890,AT5G67300,AT5G67390,AT5G08000,AT3G12980,AT1G10200,AT5G66710,AT5G06720,AT3G17130,AT1G09380,AT5G66530,AT3G17100,AT5G65990,AT1G01430,AT3G23280,AT4G23750,AT4G23200,AT4G19670,AT1G79610,AT1G24320,AT5G01750,AT3G23325,AT1G60490,AT1G49750,AT4G17880,AT5G59540,AT1G18350,AT3G61430,AT1G08130,AT1G10650,AT1G50530,AT3G11900,AT1G22530,AT5G56520,AT3G59020,AT2G16600,AT1G35580,AT5G56020,AT2G25510,AT5G54110,AT1G27600,AT4G03030,AT3G09250,AT2G01680,AT3G54680,AT4G01130
TAATCATTA	10	4	['16-20', '4-8']	108	ATCG00650,AT3G10230,AT2G26520,AT3G53190,AT5G50012,AT5G49910,AT1G62750,AT3G01060,AT1G66940,AT3G50610,AT3G50500,AT2G32390,AT3G50240,AT2G41410,AT3G49590,AT3G48530,AT5G45830,AT1G20620,AT5G44580,AT1G54520,AT4G15530,AT3G46510,AT3G12110,AT1G78480,AT1G76790,AT2G34580,AT5G39210,AT5G13090,AT5G08410,AT3G52380,AT3G52340,AT2G42600,AT3G30380,AT2G42580,AT4G37680,AT1G66410,AT5G20840,AT2G30900,AT4G37020,AT4G36040,AT5G23730,AT1G70700,AT4G36600,AT4G35090,AT2G21540,AT2G46450,AT2G46340,AT2G40080,AT3G19100,AT2G44360,AT3G28180,AT5G17600,AT2G36430,AT5G15860,AT5G15510,AT4G30610,AT3G17430,AT1G53280,AT4G28610,AT5G27140,AT5G25900,AT5G10510,AT5G24890,AT5G67300,AT5G67390,AT5G08000,AT3G12980,AT1G10200,AT5G66710,AT5G06720,AT3G17130,AT1G09380,AT5G66530,AT3G17100,AT5G65990,AT1G01430,AT3G23280,AT4G23750,AT4G23200,AT4G19670,AT1G79610,AT1G24320,AT5G01750,AT3G23325,AT1G60490,AT1G49750,AT4G17880,AT5G59540,AT1G18350,AT3G61430,AT1G08130,AT1G10650,AT1G50530,AT3G11900,AT1G22530,AT5G56520,AT3G59020,AT2G16600,AT1G35580,AT5G56020,AT2G25510,AT5G54110,AT1G27600,AT4G03030,AT3G09250,AT2G01680,AT3G54680,AT4G01130
TAATCATTA	10	5	['4-8', '16-20']	108	ATCG00650,AT3G10230,AT2G26520,AT3G53190,AT5G50012,AT5G49910,AT1G62750,AT3G01060,AT1G66940,AT3G50610,AT3G50500,AT2G32390,AT3G50240,AT2G41410,AT3G49590,AT3G48530,AT5G45830,AT1G20620,AT5G44580,AT1G54520,AT4G15530,AT3G46510,AT3G12110,AT1G78480,AT1G76790,AT2G34580,AT5G39210,AT5G13090,AT5G08410,AT3G52380,AT3G52340,AT2G42600,AT3G30380,AT2G42580,AT4G37680,AT1G66410,AT5G20840,AT2G30900,AT4G37020,AT4G36040,AT5G23730,AT1G70700,AT4G36600,AT4G35090,AT2G21540,AT2G46450,AT2G46340,AT2G40080,AT3G19100,AT2G44360,AT3G28180,AT5G17600,AT2G36430,AT5G15860,AT5G15510,AT4G30610,AT3G17430,AT1G53280,AT4G28610,AT5G27140,AT5G25900,AT5G10510,AT5G24890,AT5G67300,AT5G67390,AT5G08000,AT3G12980,AT1G10200,AT5G66710,AT5G06720,AT3G17130,AT1G09380,AT5G66530,AT3G17100,AT5G65990,AT1G01430,AT3G23280,AT4G23750,AT4G23200,AT4G19670,AT1G79610,AT1G24320,AT5G01750,AT3G23325,AT1G60490,AT1G49750,AT4G17880,AT5G59540,AT1G18350,AT3G61430,AT1G08130,AT1G10650,AT1G50530,AT3G11900,AT1G22530,AT5G56520,AT3G59020,AT2G16600,AT1G35580,AT5G56020,AT2G25510,AT5G54110,AT1G27600,AT4G03030,AT3G09250,AT2G01680,AT3G54680,AT4G01130

TACCGACAA	10	1	['0-4', '4-8', '8-12', '20-24']	19	AT3G04780,AT5G16430,AT1G17050,AT3G07200,AT5G19210,AT1G03430,AT3G62660,AT1G29395,AT1G13930,AT5G62670,AT2G28720,AT1G35950,AT1G24320,AT1G01240,AT3G01440,AT5G62140,AT1G55960,AT5G46020,AT5G12470
TACCGACAA	10	2	['0-4', '4-8', '8-12', '20-24']	19	AT3G04780,AT5G16430,AT1G17050,AT3G07200,AT5G19210,AT1G03430,AT3G62660,AT1G29395,AT1G13930,AT5G62670,AT2G28720,AT1G35950,AT1G24320,AT1G01240,AT3G01440,AT5G62140,AT1G55960,AT5G46020,AT5G12470
TACCGACAA	10	3	['0-4', '4-8', '8-12', '20-24']	19	AT3G04780,AT5G16430,AT1G17050,AT3G07200,AT5G19210,AT1G03430,AT3G62660,AT1G29395,AT1G13930,AT5G62670,AT2G28720,AT1G35950,AT1G24320,AT1G01240,AT3G01440,AT5G62140,AT1G55960,AT5G46020,AT5G12470
TACCGACAA	10	4	['0-4', '4-8', '8-12', '20-24']	19	AT3G04780,AT5G16430,AT1G17050,AT3G07200,AT5G19210,AT1G03430,AT3G62660,AT1G29395,AT1G13930,AT5G62670,AT2G28720,AT1G35950,AT1G24320,AT1G01240,AT3G01440,AT5G62140,AT1G55960,AT5G46020,AT5G12470
TACCGACAA	10	5	['0-4', '4-8', '8-12', '20-24']	19	AT3G04780,AT5G16430,AT1G17050,AT3G07200,AT5G19210,AT1G03430,AT3G62660,AT1G29395,AT1G13930,AT5G62670,AT2G28720,AT1G35950,AT1G24320,AT1G01240,AT3G01440,AT5G62140,AT1G55960,AT5G46020,AT5G12470
TACCGACAT	10	1	['4-8', '8-12', '16-20', '20-24']	15	AT3G45600,AT4G19670,AT2G15970,AT3G19553,AT2G43710,AT5G17990,AT5G49910,AT5G57660,AT3G42570,AT1G09340,AT5G51840,AT1G50940,AT2G25510,AT4G24810,AT1G64770
TACCGACAT	10	2	['4-8', '8-12', '16-20', '20-24']	15	AT3G45600,AT4G19670,AT2G15970,AT3G19553,AT2G43710,AT5G17990,AT5G49910,AT5G57660,AT3G42570,AT1G09340,AT5G51840,AT1G50940,AT2G25510,AT4G24810,AT1G64770
TACCGACAT	10	3	['4-8', '8-12', '20-24', '16-20']	15	AT3G45600,AT4G19670,AT2G15970,AT3G19553,AT2G43710,AT5G17990,AT5G49910,AT5G57660,AT3G42570,AT1G09340,AT5G51840,AT1G50940,AT2G25510,AT4G24810,AT1G64770
TACCGACAT	10	4	['4-8', '8-12', '16-20', '20-24']	15	AT3G45600,AT4G19670,AT2G15970,AT3G19553,AT2G43710,AT5G17990,AT5G49910,AT5G57660,AT3G42570,AT1G09340,AT5G51840,AT1G50940,AT2G25510,AT4G24810,AT1G64770
TACCGACAT	10	5	['4-8', '8-12', '16-20', '20-24', '0-4']	15	AT3G45600,AT4G19670,AT2G15970,AT3G19553,AT2G43710,AT5G17990,AT5G49910,AT5G57660,AT3G42570,AT1G09340,AT5G51840,AT1G50940,AT2G25510,AT4G24810,AT1G64770
TACCGACCA	10	1	['4-8', '8-12', '12-16', '20-24']	15	AT5G54110,AT1G63630,AT4G32230,AT5G55960,AT5G39740,AT5G19940,AT1G79600,AT3G08730,AT2G17450,AT5G24150,AT1G74910,AT2G18280,AT4G01490,AT2G35940,AT5G16715
TACCGACCA	10	2	['4-8', '8-12', '12-16', '20-24']	15	AT5G54110,AT1G63630,AT4G32230,AT5G55960,AT5G39740,AT5G19940,AT1G79600,AT3G08730,AT2G17450,AT5G24150,AT1G74910,AT2G18280,AT4G01490,AT2G35940,AT5G16715
TACCGACCA	10	3	['4-8', '8-12', '12-16', '20-24']	15	AT5G54110,AT1G63630,AT4G32230,AT5G55960,AT5G39740,AT5G19940,AT1G79600,AT3G08730,AT2G17450,AT5G24150,AT1G74910,AT2G18280,AT4G01490,AT2G35940,AT5G16715
TACCGACCA	10	4	['4-8', '8-12', '12-16', '20-24']	15	AT5G54110,AT1G63630,AT4G32230,AT5G55960,AT5G39740,AT5G19940,AT1G79600,AT3G08730,AT2G17450,AT5G24150,AT1G74910,AT2G18280,AT4G01490,AT2G35940,AT5G16715
TACCGACCA	10	5	['4-8', '8-12', '12-16', '20-24', '0-4']	15	AT5G54110,AT1G63630,AT4G32230,AT5G55960,AT5G39740,AT5G19940,AT1G79600,AT3G08730,AT2G17450,AT5G24150,AT1G74910,AT2G18280,AT4G01490,AT2G35940,AT5G16715
TACCGACCT	10	1	['8-12', '16-20', '20-24']	10	AT5G56020,AT4G26670,AT1G14990,AT4G38570,AT1G18460,AT2G35660,AT1G63080,AT4G29510,AT1G07420,AT2G30570
TACCGACCT	10	2	['8-12', '16-20', '20-24']	10	AT5G56020,AT4G26670,AT1G14990,AT4G38570,AT1G18460,AT2G35660,AT1G63080,AT4G29510,AT1G07420,AT2G30570
TACCGACCT	10	3	['8-12', '20-24', '16-20']	10	AT5G56020,AT4G26670,AT1G14990,AT4G38570,AT1G18460,AT2G35660,AT1G63080,AT4G29510,AT1G07420,AT2G30570
TACCGACCT	10	4	['8-12', '16-20', '20-24']	10	AT5G56020,AT4G26670,AT1G14990,AT4G38570,AT1G18460,AT2G35660,AT1G63080,AT4G29510,AT1G07420,AT2G30570
TACCGACCT	10	5	['8-12', '16-20', '20-24']	10	AT5G56020,AT4G26670,AT1G14990,AT4G38570,AT1G18460,AT2G35660,AT1G63080,AT4G29510,AT1G07420,AT2G30570

TACCGACGA	10	1	['0-4', '4-8', '8-12', '12-16', '16-20', '20-24']	14	AT1G27630,AT3G07890,AT2G32640,AT4G34220,AT3G45260,AT5G16380,AT5G08110,AT4G28210,AT3G22200,AT1G27700,AT4G30720,AT5G52180,AT3G50860,AT4G25080
TACCGACGA	10	2	['0-4', '4-8', '8-12', '12-16', '16-20', '20-24']	14	AT1G27630,AT3G07890,AT2G32640,AT4G34220,AT3G45260,AT5G16380,AT5G08110,AT4G28210,AT3G22200,AT1G27700,AT4G30720,AT5G52180,AT3G50860,AT4G25080
TACCGACGA	10	3	['0-4', '4-8', '8-12', '12-16', '16-20', '20-24']	14	AT1G27630,AT3G07890,AT2G32640,AT4G34220,AT3G45260,AT5G16380,AT5G08110,AT4G28210,AT3G22200,AT1G27700,AT4G30720,AT5G52180,AT3G50860,AT4G25080
TACCGACGA	10	4	['0-4', '4-8', '8-12', '12-16', '16-20', '20-24']	14	AT1G27630,AT3G07890,AT2G32640,AT4G34220,AT3G45260,AT5G16380,AT5G08110,AT4G28210,AT3G22200,AT1G27700,AT4G30720,AT5G52180,AT3G50860,AT4G25080
TACCGACGA	10	5	['0-4', '4-8', '8-12', '12-16', '16-20', '20-24']	14	AT1G27630,AT3G07890,AT2G32640,AT4G34220,AT3G45260,AT5G16380,AT5G08110,AT4G28210,AT3G22200,AT1G27700,AT4G30720,AT5G52180,AT3G50860,AT4G25080
TACCGACGT	10	1	['0-4', '4-8', '8-12', '16-20']	20	AT1G21460,AT3G48940,AT4G12060,AT1G20696,AT1G69830,AT5G45300,AT5G67480,AT3G62410,AT4G24340,AT3G12530,AT5G36220,AT5G47800,AT4G29510,AT1G23090,AT1G32380,AT3G12780,AT1G75950,AT3G52640,AT4G32060,AT5G54080
TACCGACGT	10	2	['0-4', '4-8', '8-12', '16-20']	20	AT1G21460,AT3G48940,AT4G12060,AT1G20696,AT1G69830,AT5G45300,AT5G67480,AT3G62410,AT4G24340,AT3G12530,AT5G36220,AT5G47800,AT4G29510,AT1G23090,AT1G32380,AT3G12780,AT1G75950,AT3G52640,AT4G32060,AT5G54080
TACCGACGT	10	3	['0-4', '4-8', '8-12', '16-20']	20	AT1G21460,AT3G48940,AT4G12060,AT1G20696,AT1G69830,AT5G45300,AT5G67480,AT3G62410,AT4G24340,AT3G12530,AT5G36220,AT5G47800,AT4G29510,AT1G23090,AT1G32380,AT3G12780,AT1G75950,AT3G52640,AT4G32060,AT5G54080
TACCGACGT	10	4	['0-4', '4-8', '8-12', '16-20']	20	AT1G21460,AT3G48940,AT4G12060,AT1G20696,AT1G69830,AT5G45300,AT5G67480,AT3G62410,AT4G24340,AT3G12530,AT5G36220,AT5G47800,AT4G29510,AT1G23090,AT1G32380,AT3G12780,AT1G75950,AT3G52640,AT4G32060,AT5G54080
TACCGACGT	10	5	['0-4', '4-8', '8-12', '16-20']	20	AT1G21460,AT3G48940,AT4G12060,AT1G20696,AT1G69830,AT5G45300,AT5G67480,AT3G62410,AT4G24340,AT3G12530,AT5G36220,AT5G47800,AT4G29510,AT1G23090,AT1G32380,AT3G12780,AT1G75950,AT3G52640,AT4G32060,AT5G54080
TACCGACTA	10	1	['0-4', '4-8', '8-12', '12-16', '16-20']	19	AT1G53280,AT1G01230,AT1G05910,AT4G26860,AT3G01850,AT5G62350,AT2G34460,AT3G20050,AT2G07360,AT1G67623,AT3G15630,AT3G19680,AT3G07640,AT5G08410,AT1G48330,AT3G19810,AT3G43790,AT4G24540,AT3G46170
TACCGACTA	10	2	['0-4', '4-8', '8-12', '12-16', '16-20']	19	AT1G53280,AT1G01230,AT1G05910,AT4G26860,AT3G01850,AT5G62350,AT2G34460,AT3G20050,AT2G07360,AT1G67623,AT3G15630,AT3G19680,AT3G07640,AT5G08410,AT1G48330,AT3G19810,AT3G43790,AT4G24540,AT3G46170
TACCGACTA	10	3	['0-4', '4-8', '8-12', '12-16', '16-20']	19	AT1G53280,AT1G01230,AT1G05910,AT4G26860,AT3G01850,AT5G62350,AT2G34460,AT3G20050,AT2G07360,AT1G67623,AT3G15630,AT3G19680,AT3G07640,AT5G08410,AT1G48330,AT3G19810,AT3G43790,AT4G24540,AT3G46170
TACCGACTA	10	4	['0-4', '8-12', '12-16', '16-20', '4-8']	19	AT1G53280,AT1G01230,AT1G05910,AT4G26860,AT3G01850,AT5G62350,AT2G34460,AT3G20050,AT2G07360,AT1G67623,AT3G15630,AT3G19680,AT3G07640,AT5G08410,AT1G48330,AT3G19810,AT3G43790,AT4G24540,AT3G46170
TACCGACTA	10	5	['4-8', '8-12', '12-16', '0-4', '16-20']	19	AT1G53280,AT1G01230,AT1G05910,AT4G26860,AT3G01850,AT5G62350,AT2G34460,AT3G20050,AT2G07360,AT1G67623,AT3G15630,AT3G19680,AT3G07640,AT5G08410,AT1G48330,AT3G19810,AT3G43790,AT4G24540,AT3G46170
TACCGACTT	10	1	['0-4', '4-8', '8-12', '12-16', '16-20']	10	AT1G62890,AT3G47960,AT4G00770,AT1G20440,AT5G61440,AT2G35660,AT3G06160,AT5G13630,AT3G24590,AT4G15140

TACCGACTT	10	2	['0-4', '4-8', '8-12', '16-20', '12-16']	10	AT1G62890,AT3G47960,AT4G00770,AT1G20440,AT5G61440,AT2G35660,AT3G06160,AT5G13630,AT3G24590,AT4G15140
TACCGACTT	10	3	['0-4', '4-8', '8-12', '12-16', '16-20']	10	AT1G62890,AT3G47960,AT4G00770,AT1G20440,AT5G61440,AT2G35660,AT3G06160,AT5G13630,AT3G24590,AT4G15140
TACCGACTT	10	4	['0-4', '4-8', '8-12', '12-16', '16-20']	10	AT1G62890,AT3G47960,AT4G00770,AT1G20440,AT5G61440,AT2G35660,AT3G06160,AT5G13630,AT3G24590,AT4G15140
TACCGACTT	10	5	['0-4', '4-8', '8-12', '12-16', '16-20']	10	AT1G62890,AT3G47960,AT4G00770,AT1G20440,AT5G61440,AT2G35660,AT3G06160,AT5G13630,AT3G24590,AT4G15140
TACGTACAA	10	1	['8-12', '20-24']	44	AT5G61130,AT5G11930,AT2G03890,AT5G50335,AT2G24270,AT3G53250,AT4G38940,AT1G50020,AT2G39400,AT1G49010,AT1G78290,AT4G28130,AT3G30290,AT3G07880,AT1G28580,AT5G07440,AT1G53640,AT3G13690,AT1G02340,AT1G52880,AT1G09390,AT4G17070,AT1G58290,AT5G46690,AT5G05710,AT5G04940,AT5G55960,AT3G48110,AT5G04470,AT2G27490,AT5G63410,AT2G22500,AT2G22420,AT2G19790,AT5G15450,AT2G40110,AT3G45060,AT2G01290,AT4G30610,AT1G73390,AT5G62280,AT5G35180,AT2G25950,AT2G44670
TACGTACAA	10	2	['8-12', '20-24']	44	AT5G61130,AT5G11930,AT2G03890,AT5G50335,AT2G24270,AT3G53250,AT4G38940,AT1G50020,AT2G39400,AT1G49010,AT1G78290,AT4G28130,AT3G30290,AT3G07880,AT1G28580,AT5G07440,AT1G53640,AT3G13690,AT1G02340,AT1G52880,AT1G09390,AT4G17070,AT1G58290,AT5G46690,AT5G05710,AT5G04940,AT5G55960,AT3G48110,AT5G04470,AT2G27490,AT5G63410,AT2G22500,AT2G22420,AT2G19790,AT5G15450,AT2G40110,AT3G45060,AT2G01290,AT4G30610,AT1G73390,AT5G62280,AT5G35180,AT2G25950,AT2G44670
TACGTACAA	10	3	['8-12', '20-24']	44	AT5G61130,AT5G11930,AT2G03890,AT5G50335,AT2G24270,AT3G53250,AT4G38940,AT1G50020,AT2G39400,AT1G49010,AT1G78290,AT4G28130,AT3G30290,AT3G07880,AT1G28580,AT5G07440,AT1G53640,AT3G13690,AT1G02340,AT1G52880,AT1G09390,AT4G17070,AT1G58290,AT5G46690,AT5G05710,AT5G04940,AT5G55960,AT3G48110,AT5G04470,AT2G27490,AT5G63410,AT2G22500,AT2G22420,AT2G19790,AT5G15450,AT2G40110,AT3G45060,AT2G01290,AT4G30610,AT1G73390,AT5G62280,AT5G35180,AT2G25950,AT2G44670
TACGTACAA	10	4	['8-12', '20-24']	44	AT5G61130,AT5G11930,AT2G03890,AT5G50335,AT2G24270,AT3G53250,AT4G38940,AT1G50020,AT2G39400,AT1G49010,AT1G78290,AT4G28130,AT3G30290,AT3G07880,AT1G28580,AT5G07440,AT1G53640,AT3G13690,AT1G02340,AT1G52880,AT1G09390,AT4G17070,AT1G58290,AT5G46690,AT5G05710,AT5G04940,AT5G55960,AT3G48110,AT5G04470,AT2G27490,AT5G63410,AT2G22500,AT2G22420,AT2G19790,AT5G15450,AT2G40110,AT3G45060,AT2G01290,AT4G30610,AT1G73390,AT5G62280,AT5G35180,AT2G25950,AT2G44670
TACGTACAA	10	5	['8-12', '20-24']	44	AT5G61130,AT5G11930,AT2G03890,AT5G50335,AT2G24270,AT3G53250,AT4G38940,AT1G50020,AT2G39400,AT1G49010,AT1G78290,AT4G28130,AT3G30290,AT3G07880,AT1G28580,AT5G07440,AT1G53640,AT3G13690,AT1G02340,AT1G52880,AT1G09390,AT4G17070,AT1G58290,AT5G46690,AT5G05710,AT5G04940,AT5G55960,AT3G48110,AT5G04470,AT2G27490,AT5G63410,AT2G22500,AT2G22420,AT2G19790,AT5G15450,AT2G40110,AT3G45060,AT2G01290,AT4G30610,AT1G73390,AT5G62280,AT5G35180,AT2G25950,AT2G44670
TACGTGGA	10	1	['0-4', '4-8', '8-12']	63	AT5G01600,AT4G28540,AT1G22790,AT3G62410,AT5G11600,AT4G17840,AT5G09270,AT5G60120,AT4G00370,AT2G18640,AT5G35840,AT2G33240,AT2G36720,AT1G17970,AT4G26700,AT2G27600,AT1G17745,AT5G23730,AT1G33970,AT4G14030,AT5G23050,AT5G05950,AT3G17040,AT4G09620,AT5G05710,AT3G59480,AT3G59350,AT2G46570,AT5G44650,AT3G21250,AT5G64460,AT1G70000,AT1G36830,AT5G04790,AT1G51660,AT1G73980,AT4G33470,AT1G69870,AT3G05670,AT4G32890,AT2G35880,AT2G02710,AT1G72430,AT3G27210,AT3G57190,AT1G03130,AT4G03510,AT5G15230,AT3G01990,AT2G32765,AT1G78600,AT5G15510,AT3G17800,AT1G66840,AT2G43710,AT2G27880,AT3G27690,AT2G35620,AT3G15580,AT1G72300,AT4G29610,AT1G30210,AT2G26080

TACGTGGA	10	2	['0-4', '4-8', '8-12']	63	AT5G01600,AT4G28540,AT1G22790,AT3G62410,AT5G11600,AT4G17840,AT5G09270,AT5G60120,AT4G00370,AT2G18640,AT5G35840,AT2G33240,AT2G36720,AT1G17970,AT4G26700,AT2G27600,AT1G17745,AT5G23730,AT1G33970,AT4G14030,AT5G23050,AT5G05950,AT3G17040,AT4G09620,AT5G05710,AT3G59480,AT3G59350,AT2G46570,AT5G44650,AT3G21250,AT5G64460,AT1G70000,AT1G36830,AT5G04790,AT1G51660,AT1G73980,AT4G33470,AT1G69870,AT3G05670,AT4G32890,AT2G35880,AT2G02710,AT1G72430,AT3G27210,AT3G57190,AT1G03130,AT4G03510,AT5G15230,AT3G01990,AT2G32765,AT1G78600,AT5G15510,AT3G17800,AT1G66840,AT2G43710,AT2G27880,AT3G27690,AT2G35620,AT3G15580,AT1G72300,AT4G29610,AT1G30210,AT2G26080
TACGTGGA	10	3	['0-4', '4-8', '8-12']	63	AT5G01600,AT4G28540,AT1G22790,AT3G62410,AT5G11600,AT4G17840,AT5G09270,AT5G60120,AT4G00370,AT2G18640,AT5G35840,AT2G33240,AT2G36720,AT1G17970,AT4G26700,AT2G27600,AT1G17745,AT5G23730,AT1G33970,AT4G14030,AT5G23050,AT5G05950,AT3G17040,AT4G09620,AT5G05710,AT3G59480,AT3G59350,AT2G46570,AT5G44650,AT3G21250,AT5G64460,AT1G70000,AT1G36830,AT5G04790,AT1G51660,AT1G73980,AT4G33470,AT1G69870,AT3G05670,AT4G32890,AT2G35880,AT2G02710,AT1G72430,AT3G27210,AT3G57190,AT1G03130,AT4G03510,AT5G15230,AT3G01990,AT2G32765,AT1G78600,AT5G15510,AT3G17800,AT1G66840,AT2G43710,AT2G27880,AT3G27690,AT2G35620,AT3G15580,AT1G72300,AT4G29610,AT1G30210,AT2G26080
TACGTGGA	10	4	['0-4', '4-8', '8-12']	63	AT5G01600,AT4G28540,AT1G22790,AT3G62410,AT5G11600,AT4G17840,AT5G09270,AT5G60120,AT4G00370,AT2G18640,AT5G35840,AT2G33240,AT2G36720,AT1G17970,AT4G26700,AT2G27600,AT1G17745,AT5G23730,AT1G33970,AT4G14030,AT5G23050,AT5G05950,AT3G17040,AT4G09620,AT5G05710,AT3G59480,AT3G59350,AT2G46570,AT5G44650,AT3G21250,AT5G64460,AT1G70000,AT1G36830,AT5G04790,AT1G51660,AT1G73980,AT4G33470,AT1G69870,AT3G05670,AT4G32890,AT2G35880,AT2G02710,AT1G72430,AT3G27210,AT3G57190,AT1G03130,AT4G03510,AT5G15230,AT3G01990,AT2G32765,AT1G78600,AT5G15510,AT3G17800,AT1G66840,AT2G43710,AT2G27880,AT3G27690,AT2G35620,AT3G15580,AT1G72300,AT4G29610,AT1G30210,AT2G26080
TACGTGGA	10	5	['4-8', '8-12', '0-4']	63	AT5G01600,AT4G28540,AT1G22790,AT3G62410,AT5G11600,AT4G17840,AT5G09270,AT5G60120,AT4G00370,AT2G18640,AT5G35840,AT2G33240,AT2G36720,AT1G17970,AT4G26700,AT2G27600,AT1G17745,AT5G23730,AT1G33970,AT4G14030,AT5G23050,AT5G05950,AT3G17040,AT4G09620,AT5G05710,AT3G59480,AT3G59350,AT2G46570,AT5G44650,AT3G21250,AT5G64460,AT1G70000,AT1G36830,AT5G04790,AT1G51660,AT1G73980,AT4G33470,AT1G69870,AT3G05670,AT4G32890,AT2G35880,AT2G02710,AT1G72430,AT3G27210,AT3G57190,AT1G03130,AT4G03510,AT5G15230,AT3G01990,AT2G32765,AT1G78600,AT5G15510,AT3G17800,AT1G66840,AT2G43710,AT2G27880,AT3G27690,AT2G35620,AT3G15580,AT1G72300,AT4G29610,AT1G30210,AT2G26080
TACGTGTA	10	1	['4-8']	102	AT4G01120,AT1G15400,AT2G26500,AT5G49710,AT1G14040,AT2G47590,AT3G01090,AT5G48490,AT3G51240,AT3G50950,AT1G66940,AT3G50530,AT1G22200,AT1G17745,AT3G49220,AT1G14810,AT2G07140,AT3G47650,AT1G28050,AT1G54390,AT1G68920,AT3G12610,AT4G15110,AT5G42940,AT1G54880,AT1G03000,AT1G05720,AT5G41810,AT5G41220,AT5G40450,AT4G39950,AT1G74160,AT1G53670,AT1G66100,AT2G39560,AT4G39120,AT5G37780,AT2G38410,AT3G28340,AT2G41430,AT3G30380,AT5G35460,AT2G34140,AT2G01860,AT4G35800,AT1G67623,AT1G74660,AT5G19250,AT2G39930,AT4G34250,AT4G33985,AT3G24982,AT2G35940,AT5G17380,AT3G13110,AT3G23690,AT2G45590,AT2G42190,AT3G15030,AT1G32440,AT3G26660,AT1G09240,AT5G10510,AT5G10430,AT5G10210,AT4G27670,AT1G11850,AT3G23030,AT1G06040,AT1G17360,AT5G06060,AT1G65640,AT4G24350,AT1G26800,AT1G26780,AT3G17800,AT5G62670,AT2G17330,AT1G23205,AT1G23080,AT1G23090,AT5G62130,AT5G62280,AT5G62140,AT3G16110,AT4G18520,AT3G17250,AT3G62660,AT3G06780,AT3G07010,AT5G58870,AT2G36800,AT5G57760,AT3G59060,AT3G04550,AT3G57020,AT3G06410,AT1G70782,AT3G55670,AT2G35750,AT3G54900,AT3G54240

TACGTGTA	10	2	['4-8']	102	AT4G01120,AT1G15400,AT2G26500,AT5G49710,AT1G14040,AT2G47590,AT3G01090,AT5G48490,AT3G51240,AT3G50950,AT1G66940,AT3G50530,AT1G22200,AT1G17745,AT3G49220,AT1G14810,AT2G07140,AT3G47650,AT1G28050,AT1G54390,AT1G68920,AT3G12610,AT4G15110,AT5G42940,AT1G54880,AT1G03000,AT1G05720,AT5G41810,AT5G41220,AT5G40450,AT4G39950,AT1G74160,AT1G53670,AT1G66100,AT2G39560,AT4G39120,AT5G37780,AT2G38410,AT3G28340,AT2G41430,AT3G30380,AT5G35460,AT2G34140,AT2G01860,AT4G35800,AT1G67623,AT1G74660,AT5G19250,AT2G39930,AT4G34250,AT4G33985,AT3G24982,AT2G35940,AT5G17380,AT3G13110,AT3G23690,AT2G45590,AT2G42190,AT3G15030,AT1G32440,AT3G26660,AT1G09240,AT5G10510,AT5G10430,AT5G10210,AT4G27670,AT1G11850,AT3G23030,AT1G06040,AT1G17360,AT5G06060,AT1G65640,AT4G24350,AT1G26800,AT1G26780,AT3G17800,AT5G62670,AT2G17330,AT1G23205,AT1G23080,AT1G23090,AT5G62130,AT5G62280,AT5G62140,AT3G16110,AT4G18520,AT3G17250,AT3G62660,AT3G06780,AT3G07010,AT5G58870,AT2G36800,AT5G57760,AT3G59060,AT3G04550,AT3G57020,AT3G06410,AT1G70782,AT3G55670,AT2G35750,AT3G54900,AT3G54240
TACGTGTA	10	3	['4-8']	102	AT4G01120,AT1G15400,AT2G26500,AT5G49710,AT1G14040,AT2G47590,AT3G01090,AT5G48490,AT3G51240,AT3G50950,AT1G66940,AT3G50530,AT1G22200,AT1G17745,AT3G49220,AT1G14810,AT2G07140,AT3G47650,AT1G28050,AT1G54390,AT1G68920,AT3G12610,AT4G15110,AT5G42940,AT1G54880,AT1G03000,AT1G05720,AT5G41810,AT5G41220,AT5G40450,AT4G39950,AT1G74160,AT1G53670,AT1G66100,AT2G39560,AT4G39120,AT5G37780,AT2G38410,AT3G28340,AT2G41430,AT3G30380,AT5G35460,AT2G34140,AT2G01860,AT4G35800,AT1G67623,AT1G74660,AT5G19250,AT2G39930,AT4G34250,AT4G33985,AT3G24982,AT2G35940,AT5G17380,AT3G13110,AT3G23690,AT2G45590,AT2G42190,AT3G15030,AT1G32440,AT3G26660,AT1G09240,AT5G10510,AT5G10430,AT5G10210,AT4G27670,AT1G11850,AT3G23030,AT1G06040,AT1G17360,AT5G06060,AT1G65640,AT4G24350,AT1G26800,AT1G26780,AT3G17800,AT5G62670,AT2G17330,AT1G23205,AT1G23080,AT1G23090,AT5G62130,AT5G62280,AT5G62140,AT3G16110,AT4G18520,AT3G17250,AT3G62660,AT3G06780,AT3G07010,AT5G58870,AT2G36800,AT5G57760,AT3G59060,AT3G04550,AT3G57020,AT3G06410,AT1G70782,AT3G55670,AT2G35750,AT3G54900,AT3G54240
TACGTGTA	10	4	['4-8']	102	AT4G01120,AT1G15400,AT2G26500,AT5G49710,AT1G14040,AT2G47590,AT3G01090,AT5G48490,AT3G51240,AT3G50950,AT1G66940,AT3G50530,AT1G22200,AT1G17745,AT3G49220,AT1G14810,AT2G07140,AT3G47650,AT1G28050,AT1G54390,AT1G68920,AT3G12610,AT4G15110,AT5G42940,AT1G54880,AT1G03000,AT1G05720,AT5G41810,AT5G41220,AT5G40450,AT4G39950,AT1G74160,AT1G53670,AT1G66100,AT2G39560,AT4G39120,AT5G37780,AT2G38410,AT3G28340,AT2G41430,AT3G30380,AT5G35460,AT2G34140,AT2G01860,AT4G35800,AT1G67623,AT1G74660,AT5G19250,AT2G39930,AT4G34250,AT4G33985,AT3G24982,AT2G35940,AT5G17380,AT3G13110,AT3G23690,AT2G45590,AT2G42190,AT3G15030,AT1G32440,AT3G26660,AT1G09240,AT5G10510,AT5G10430,AT5G10210,AT4G27670,AT1G11850,AT3G23030,AT1G06040,AT1G17360,AT5G06060,AT1G65640,AT4G24350,AT1G26800,AT1G26780,AT3G17800,AT5G62670,AT2G17330,AT1G23205,AT1G23080,AT1G23090,AT5G62130,AT5G62280,AT5G62140,AT3G16110,AT4G18520,AT3G17250,AT3G62660,AT3G06780,AT3G07010,AT5G58870,AT2G36800,AT5G57760,AT3G59060,AT3G04550,AT3G57020,AT3G06410,AT1G70782,AT3G55670,AT2G35750,AT3G54900,AT3G54240
TACGTGTA	10	5	['4-8']	102	AT4G01120,AT1G15400,AT2G26500,AT5G49710,AT1G14040,AT2G47590,AT3G01090,AT5G48490,AT3G51240,AT3G50950,AT1G66940,AT3G50530,AT1G22200,AT1G17745,AT3G49220,AT1G14810,AT2G07140,AT3G47650,AT1G28050,AT1G54390,AT1G68920,AT3G12610,AT4G15110,AT5G42940,AT1G54880,AT1G03000,AT1G05720,AT5G41810,AT5G41220,AT5G40450,AT4G39950,AT1G74160,AT1G53670,AT1G66100,AT2G39560,AT4G39120,AT5G37780,AT2G38410,AT3G28340,AT2G41430,AT3G30380,AT5G35460,AT2G34140,AT2G01860,AT4G35800,AT1G67623,AT1G74660,AT5G19250,AT2G39930,AT4G34250,AT4G33985,AT3G24982,AT2G35940,AT5G17380,AT3G13110,AT3G23690,AT2G45590,AT2G42190,AT3G15030,AT1G32440,AT3G26660,AT1G09240,AT5G10510,AT5G10430,AT5G10210,AT4G27670,AT1G11850,AT3G23030,AT1G06040,AT1G17360,AT5G06060,AT1G65640,AT4G24350,AT1G26800,AT1G26780,AT3G17800,AT5G62670,AT2G17330,AT1G23205,AT1G23080,AT1G23090,AT5G62130,AT5G62280,AT5G62140,AT3G16110,AT4G18520,AT3G17250,AT3G62660,AT3G06780,AT3G07010,AT5G58870,AT2G36800,AT5G57760,AT3G59060,AT3G04550,AT3G57020,AT3G06410,AT1G70782,AT3G55670,AT2G35750,AT3G54900,AT3G54240

TACGTGTC	10	1	['0-4', '16-20', '20-24']	59	AT1G72770,AT1G19300,AT3G53720,AT2G07730,AT4G18280,AT5G50680,AT4G00895,AT2G05920,AT2G26500,AT5G11500,AT1G27700,AT4G27780,AT3G07700,AT1G18460,AT3G07770,AT5G37540,AT4G27410,AT1G16520,AT1G07950,AT3G60770,AT4G37560,AT3G18610,AT1G31812,AT3G19810,AT4G34950,AT4G24830,AT5G19260,AT1G80090,AT1G32870,AT5G44730,AT1G77920,AT1G20440,AT1G70230,AT4G05050,AT4G16860,AT4G16870,AT2G33700,AT3G57770,AT2G29670,AT1G50940,AT2G24490,AT4G21180,AT1G79770,AT1G69530,AT1G78450,AT1G25230,AT4G20160,AT5G02490,AT3G45230,AT5G02650,AT2G03310,AT3G08890,AT5G02150,AT3G07350,AT5G04140,AT5G51720,AT5G61850,AT3G54680,AT3G15360
TACGTGTC	10	2	['0-4', '16-20', '20-24']	59	AT1G72770,AT1G19300,AT3G53720,AT2G07730,AT4G18280,AT5G50680,AT4G00895,AT2G05920,AT2G26500,AT5G11500,AT1G27700,AT4G27780,AT3G07700,AT1G18460,AT3G07770,AT5G37540,AT4G27410,AT1G16520,AT1G07950,AT3G60770,AT4G37560,AT3G18610,AT1G31812,AT3G19810,AT4G34950,AT4G24830,AT5G19260,AT1G80090,AT1G32870,AT5G44730,AT1G77920,AT1G20440,AT1G70230,AT4G05050,AT4G16860,AT4G16870,AT2G33700,AT3G57770,AT2G29670,AT1G50940,AT2G24490,AT4G21180,AT1G79770,AT1G69530,AT1G78450,AT1G25230,AT4G20160,AT5G02490,AT3G45230,AT5G02650,AT2G03310,AT3G08890,AT5G02150,AT3G07350,AT5G04140,AT5G51720,AT5G61850,AT3G54680,AT3G15360
TACGTGTC	10	3	['0-4', '16-20', '20-24']	59	AT1G72770,AT1G19300,AT3G53720,AT2G07730,AT4G18280,AT5G50680,AT4G00895,AT2G05920,AT2G26500,AT5G11500,AT1G27700,AT4G27780,AT3G07700,AT1G18460,AT3G07770,AT5G37540,AT4G27410,AT1G16520,AT1G07950,AT3G60770,AT4G37560,AT3G18610,AT1G31812,AT3G19810,AT4G34950,AT4G24830,AT5G19260,AT1G80090,AT1G32870,AT5G44730,AT1G77920,AT1G20440,AT1G70230,AT4G05050,AT4G16860,AT4G16870,AT2G33700,AT3G57770,AT2G29670,AT1G50940,AT2G24490,AT4G21180,AT1G79770,AT1G69530,AT1G78450,AT1G25230,AT4G20160,AT5G02490,AT3G45230,AT5G02650,AT2G03310,AT3G08890,AT5G02150,AT3G07350,AT5G04140,AT5G51720,AT5G61850,AT3G54680,AT3G15360
TACGTGTC	10	4	['0-4', '16-20', '20-24']	59	AT1G72770,AT1G19300,AT3G53720,AT2G07730,AT4G18280,AT5G50680,AT4G00895,AT2G05920,AT2G26500,AT5G11500,AT1G27700,AT4G27780,AT3G07700,AT1G18460,AT3G07770,AT5G37540,AT4G27410,AT1G16520,AT1G07950,AT3G60770,AT4G37560,AT3G18610,AT1G31812,AT3G19810,AT4G34950,AT4G24830,AT5G19260,AT1G80090,AT1G32870,AT5G44730,AT1G77920,AT1G20440,AT1G70230,AT4G05050,AT4G16860,AT4G16870,AT2G33700,AT3G57770,AT2G29670,AT1G50940,AT2G24490,AT4G21180,AT1G79770,AT1G69530,AT1G78450,AT1G25230,AT4G20160,AT5G02490,AT3G45230,AT5G02650,AT2G03310,AT3G08890,AT5G02150,AT3G07350,AT5G04140,AT5G51720,AT5G61850,AT3G54680,AT3G15360
TACGTGTC	10	5	['16-20', '20-24', '0-4']	59	AT1G72770,AT1G19300,AT3G53720,AT2G07730,AT4G18280,AT5G50680,AT4G00895,AT2G05920,AT2G26500,AT5G11500,AT1G27700,AT4G27780,AT3G07700,AT1G18460,AT3G07770,AT5G37540,AT4G27410,AT1G16520,AT1G07950,AT3G60770,AT4G37560,AT3G18610,AT1G31812,AT3G19810,AT4G34950,AT4G24830,AT5G19260,AT1G80090,AT1G32870,AT5G44730,AT1G77920,AT1G20440,AT1G70230,AT4G05050,AT4G16860,AT4G16870,AT2G33700,AT3G57770,AT2G29670,AT1G50940,AT2G24490,AT4G21180,AT1G79770,AT1G69530,AT1G78450,AT1G25230,AT4G20160,AT5G02490,AT3G45230,AT5G02650,AT2G03310,AT3G08890,AT5G02150,AT3G07350,AT5G04140,AT5G51720,AT5G61850,AT3G54680,AT3G15360
TAGTGCTGT	10	1	['0-4', '4-8', '8-12', '12-16', '16-20', '20-24']	10	AT2G42590,AT1G18180,AT1G63800,AT5G43830,AT5G11060,AT1G16720,AT3G59660,AT3G02720,AT3G27300,AT2G30390
TAGTGCTGT	10	2	['0-4', '4-8', '8-12', '12-16', '16-20', '20-24']	10	AT2G42590,AT1G18180,AT1G63800,AT5G43830,AT5G11060,AT1G16720,AT3G59660,AT3G02720,AT3G27300,AT2G30390
TAGTGCTGT	10	3	['0-4', '4-8', '8-12', '12-16', '16-20', '20-24']	10	AT2G42590,AT1G18180,AT1G63800,AT5G43830,AT5G11060,AT1G16720,AT3G59660,AT3G02720,AT3G27300,AT2G30390
TAGTGCTGT	10	4	['0-4', '4-8', '8-12', '12-16', '16-20', '20-24']	10	AT2G42590,AT1G18180,AT1G63800,AT5G43830,AT5G11060,AT1G16720,AT3G59660,AT3G02720,AT3G27300,AT2G30390

TAGTGCTGT	10	5	['0-4', '4-8', '8-12', '12-16', '16-20', '20-24']	10	AT2G42590,AT1G18180,AT1G63800,AT5G43830,AT5G11060,AT1G16720,AT3G59660,AT3G02720,AT3G27300,AT2G30390
TAGTGGAT	10	1	['4-8', '20-24']	101	AT2G19650,AT2G26520,AT4G00400,AT5G48900,AT3G51970,AT2G27600,AT3G16470,AT2G29250,AT5G47620,AT1G22360,AT4G17615,AT1G22280,AT4G16845,AT5G46630,AT1G59930,AT5G44520,AT2G46170,AT4G17140,AT1G75690,AT3G12470,AT1G54590,AT1G69500,AT5G41760,AT2G47010,AT1G30220,AT2G38820,AT2G38800,AT5G19860,AT5G37780,AT2G38120,AT5G35840,AT5G20280,AT5G35560,AT5G20380,AT4G34490,AT3G22550,AT3G13430,AT3G51820,AT2G40080,AT5G18170,AT2G22030,AT1G69800,AT4G31390,AT3G14900,AT4G31050,AT2G43750,AT5G30510,AT3G26570,AT1G02170,AT1G54830,AT1G67510,AT3G26290,AT4G27470,AT5G25290,AT5G08580,AT1G05960,AT1G67110,AT1G77270,AT5G66950,AT1G09380,AT1G01430,AT5G05300,AT1G65560,AT4G23290,AT1G61390,AT5G63135,AT5G62160,AT4G19530,AT5G61440,AT1G60590,AT1G27090,AT3G62580,AT5G60210,AT4G12800,AT5G58910,AT3G61120,AT3G02750,AT3G04460,AT4G12060,AT3G61210,AT1G80840,AT5G57040,AT5G57110,AT4G08930,AT1G64640,AT2G20930,AT4G08390,AT4G08090,AT3G07200,AT3G04680,AT5G54540,AT3G57170,AT3G57090,AT3G06380,AT1G27600,AT5G53580,AT3G01990,AT3G55680,AT2G48020,AT3G55960,AT4G01280
TAGTGGAT	10	2	['4-8', '20-24']	101	AT2G19650,AT2G26520,AT4G00400,AT5G48900,AT3G51970,AT2G27600,AT3G16470,AT2G29250,AT5G47620,AT1G22360,AT4G17615,AT1G22280,AT4G16845,AT5G46630,AT1G59930,AT5G44520,AT2G46170,AT4G17140,AT1G75690,AT3G12470,AT1G54590,AT1G69500,AT5G41760,AT2G47010,AT1G30220,AT2G38820,AT2G38800,AT5G19860,AT5G37780,AT2G38120,AT5G35840,AT5G20280,AT5G35560,AT5G20380,AT4G34490,AT3G22550,AT3G13430,AT3G51820,AT2G40080,AT5G18170,AT2G22030,AT1G69800,AT4G31390,AT3G14900,AT4G31050,AT2G43750,AT5G30510,AT3G26570,AT1G02170,AT1G54830,AT1G67510,AT3G26290,AT4G27470,AT5G25290,AT5G08580,AT1G05960,AT1G67110,AT1G77270,AT5G66950,AT1G09380,AT1G01430,AT5G05300,AT1G65560,AT4G23290,AT1G61390,AT5G63135,AT5G62160,AT4G19530,AT5G61440,AT1G60590,AT1G27090,AT3G62580,AT5G60210,AT4G12800,AT5G58910,AT3G61120,AT3G02750,AT3G04460,AT4G12060,AT3G61210,AT1G80840,AT5G57040,AT5G57110,AT4G08930,AT1G64640,AT2G20930,AT4G08390,AT4G08090,AT3G07200,AT3G04680,AT5G54540,AT3G57170,AT3G57090,AT3G06380,AT1G27600,AT5G53580,AT3G01990,AT3G55680,AT2G48020,AT3G55960,AT4G01280
TAGTGGAT	10	3	['4-8', '20-24']	101	AT2G19650,AT2G26520,AT4G00400,AT5G48900,AT3G51970,AT2G27600,AT3G16470,AT2G29250,AT5G47620,AT1G22360,AT4G17615,AT1G22280,AT4G16845,AT5G46630,AT1G59930,AT5G44520,AT2G46170,AT4G17140,AT1G75690,AT3G12470,AT1G54590,AT1G69500,AT5G41760,AT2G47010,AT1G30220,AT2G38820,AT2G38800,AT5G19860,AT5G37780,AT2G38120,AT5G35840,AT5G20280,AT5G35560,AT5G20380,AT4G34490,AT3G22550,AT3G13430,AT3G51820,AT2G40080,AT5G18170,AT2G22030,AT1G69800,AT4G31390,AT3G14900,AT4G31050,AT2G43750,AT5G30510,AT3G26570,AT1G02170,AT1G54830,AT1G67510,AT3G26290,AT4G27470,AT5G25290,AT5G08580,AT1G05960,AT1G67110,AT1G77270,AT5G66950,AT1G09380,AT1G01430,AT5G05300,AT1G65560,AT4G23290,AT1G61390,AT5G63135,AT5G62160,AT4G19530,AT5G61440,AT1G60590,AT1G27090,AT3G62580,AT5G60210,AT4G12800,AT5G58910,AT3G61120,AT3G02750,AT3G04460,AT4G12060,AT3G61210,AT1G80840,AT5G57040,AT5G57110,AT4G08930,AT1G64640,AT2G20930,AT4G08390,AT4G08090,AT3G07200,AT3G04680,AT5G54540,AT3G57170,AT3G57090,AT3G06380,AT1G27600,AT5G53580,AT3G01990,AT3G55680,AT2G48020,AT3G55960,AT4G01280
TAGTGGAT	10	4	['4-8', '20-24']	101	AT2G19650,AT2G26520,AT4G00400,AT5G48900,AT3G51970,AT2G27600,AT3G16470,AT2G29250,AT5G47620,AT1G22360,AT4G17615,AT1G22280,AT4G16845,AT5G46630,AT1G59930,AT5G44520,AT2G46170,AT4G17140,AT1G75690,AT3G12470,AT1G54590,AT1G69500,AT5G41760,AT2G47010,AT1G30220,AT2G38820,AT2G38800,AT5G19860,AT5G37780,AT2G38120,AT5G35840,AT5G20280,AT5G35560,AT5G20380,AT4G34490,AT3G22550,AT3G13430,AT3G51820,AT2G40080,AT5G18170,AT2G22030,AT1G69800,AT4G31390,AT3G14900,AT4G31050,AT2G43750,AT5G30510,AT3G26570,AT1G02170,AT1G54830,AT1G67510,AT3G26290,AT4G27470,AT5G25290,AT5G08580,AT1G05960,AT1G67110,AT1G77270,AT5G66950,AT1G09380,AT1G01430,AT5G05300,AT1G65560,AT4G23290,AT1G61390,AT5G63135,AT5G62160,AT4G19530,AT5G61440,AT1G60590,AT1G27090,AT3G62580,AT5G60210,AT4G12800,AT5G58910,AT3G61120,AT3G02750,AT3G04460,AT4G12060,AT3G61210,AT1G80840,AT5G57040,AT5G57110,AT4G08930,AT1G64640,AT2G20930,AT4G08390,AT4G08090,AT3G07200,AT3G04680,AT5G54540,AT3G57170,AT3G57090,AT3G06380,AT1G27600,AT5G53580,AT3G01990,AT3G55680,AT2G48020,AT3G55960,AT4G01280

TAGTGGAT	10	5	['4-8', '20-24']	101	AT2G19650,AT2G26520,AT4G00400,AT5G48900,AT3G51970,AT2G27600,AT3G16470,AT2G29250,AT5G47620,AT1G22360,AT4G17615,AT1G22280,AT4G16845,AT5G46630,AT1G59930,AT5G44520,AT2G46170,AT4G17140,AT1G75690,AT3G12470,AT1G54590,AT1G69500,AT5G41760,AT2G47010,AT1G30220,AT2G38820,AT2G38800,AT5G19860,AT5G37780,AT2G38120,AT5G35840,AT5G20280,AT5G35560,AT5G20380,AT4G34490,AT3G22550,AT3G13430,AT3G51820,AT2G40080,AT5G18170,AT2G22030,AT1G69800,AT4G31390,AT3G14900,AT4G31050,AT2G43750,AT5G30510,AT3G26570,AT1G02170,AT1G54830,AT1G67510,AT3G26290,AT4G27470,AT5G25290,AT5G08580,AT1G05960,AT1G67110,AT1G77270,AT5G66950,AT1G09380,AT1G01430,AT5G05300,AT1G65560,AT4G23290,AT1G61390,AT5G63135,AT5G62160,AT4G19530,AT5G61440,AT1G60590,AT1G27090,AT3G62580,AT5G60210,AT4G12800,AT5G58910,AT3G61120,AT3G02750,AT3G04460,AT4G12060,AT3G61210,AT1G80840,AT5G57040,AT5G57110,AT4G08930,AT1G64640,AT2G20930,AT4G08390,AT4G08090,AT3G07200,AT3G04680,AT5G54540,AT3G57170,AT3G57090,AT3G06380,AT1G27600,AT5G53580,AT3G01990,AT3G55680,AT2G48020,AT3G55960,AT4G01280
TCATCTTCTT	10	1	['8-12', '12-16']	80	AT3G01320,AT1G75100,AT1G63630,AT5G27010,AT5G11280,AT4G28420,AT3G62550,AT2G23200,AT3G53870,AT5G10860,AT1G78290,AT2G47450,AT4G27700,AT4G38470,AT5G58910,AT2G38120,AT3G04480,AT3G61460,AT4G38160,AT5G12480,AT5G58770,AT5G58620,AT4G12590,AT1G78180,AT5G24610,AT5G57760,AT3G07840,AT3G18600,AT4G35800,AT1G33970,AT3G22970,AT5G65910,AT5G65990,AT1G55730,AT1G19060,AT1G14685,AT5G65530,AT5G05520,AT1G33780,AT2G35190,AT1G04850,AT5G44650,AT5G55530,AT3G58140,AT1G21250,AT2G36970,AT5G64180,AT1G54270,AT2G46240,AT1G73980,AT1G22850,AT1G62290,AT1G23960,AT4G15810,AT3G27300,AT5G16150,AT5G17380,AT2G25510,AT1G10960,AT2G41720,AT5G62980,AT2G19790,AT2G47320,AT5G03150,AT3G27840,AT2G26770,AT2G03310,AT1G79790,AT2G46820,AT4G20170,AT3G55680,AT2G32910,AT2G07360,AT1G73470,AT5G41150,AT1G10510,AT3G06160,AT1G79040,AT1G14380,AT5G01820
TCATCTTCTT	10	2	['8-12', '12-16']	80	AT3G01320,AT1G75100,AT1G63630,AT5G27010,AT5G11280,AT4G28420,AT3G62550,AT2G23200,AT3G53870,AT5G10860,AT1G78290,AT2G47450,AT4G27700,AT4G38470,AT5G58910,AT2G38120,AT3G04480,AT3G61460,AT4G38160,AT5G12480,AT5G58770,AT5G58620,AT4G12590,AT1G78180,AT5G24610,AT5G57760,AT3G07840,AT3G18600,AT4G35800,AT1G33970,AT3G22970,AT5G65910,AT5G65990,AT1G55730,AT1G19060,AT1G14685,AT5G65530,AT5G05520,AT1G33780,AT2G35190,AT1G04850,AT5G44650,AT5G55530,AT3G58140,AT1G21250,AT2G36970,AT5G64180,AT1G54270,AT2G46240,AT1G73980,AT1G22850,AT1G62290,AT1G23960,AT4G15810,AT3G27300,AT5G16150,AT5G17380,AT2G25510,AT1G10960,AT2G41720,AT5G62980,AT2G19790,AT2G47320,AT5G03150,AT3G27840,AT2G26770,AT2G03310,AT1G79790,AT2G46820,AT4G20170,AT3G55680,AT2G32910,AT2G07360,AT1G73470,AT5G41150,AT1G10510,AT3G06160,AT1G79040,AT1G14380,AT5G01820
TCATCTTCTT	10	3	['8-12', '12-16']	80	AT3G01320,AT1G75100,AT1G63630,AT5G27010,AT5G11280,AT4G28420,AT3G62550,AT2G23200,AT3G53870,AT5G10860,AT1G78290,AT2G47450,AT4G27700,AT4G38470,AT5G58910,AT2G38120,AT3G04480,AT3G61460,AT4G38160,AT5G12480,AT5G58770,AT5G58620,AT4G12590,AT1G78180,AT5G24610,AT5G57760,AT3G07840,AT3G18600,AT4G35800,AT1G33970,AT3G22970,AT5G65910,AT5G65990,AT1G55730,AT1G19060,AT1G14685,AT5G65530,AT5G05520,AT1G33780,AT2G35190,AT1G04850,AT5G44650,AT5G55530,AT3G58140,AT1G21250,AT2G36970,AT5G64180,AT1G54270,AT2G46240,AT1G73980,AT1G22850,AT1G62290,AT1G23960,AT4G15810,AT3G27300,AT5G16150,AT5G17380,AT2G25510,AT1G10960,AT2G41720,AT5G62980,AT2G19790,AT2G47320,AT5G03150,AT3G27840,AT2G26770,AT2G03310,AT1G79790,AT2G46820,AT4G20170,AT3G55680,AT2G32910,AT2G07360,AT1G73470,AT5G41150,AT1G10510,AT3G06160,AT1G79040,AT1G14380,AT5G01820
TCATCTTCTT	10	4	['8-12', '12-16']	80	AT3G01320,AT1G75100,AT1G63630,AT5G27010,AT5G11280,AT4G28420,AT3G62550,AT2G23200,AT3G53870,AT5G10860,AT1G78290,AT2G47450,AT4G27700,AT4G38470,AT5G58910,AT2G38120,AT3G04480,AT3G61460,AT4G38160,AT5G12480,AT5G58770,AT5G58620,AT4G12590,AT1G78180,AT5G24610,AT5G57760,AT3G07840,AT3G18600,AT4G35800,AT1G33970,AT3G22970,AT5G65910,AT5G65990,AT1G55730,AT1G19060,AT1G14685,AT5G65530,AT5G05520,AT1G33780,AT2G35190,AT1G04850,AT5G44650,AT5G55530,AT3G58140,AT1G21250,AT2G36970,AT5G64180,AT1G54270,AT2G46240,AT1G73980,AT1G22850,AT1G62290,AT1G23960,AT4G15810,AT3G27300,AT5G16150,AT5G17380,AT2G25510,AT1G10960,AT2G41720,AT5G62980,AT2G19790,AT2G47320,AT5G03150,AT3G27840,AT2G26770,AT2G03310,AT1G79790,AT2G46820,AT4G20170,AT3G55680,AT2G32910,AT2G07360,AT1G73470,AT5G41150,AT1G10510,AT3G06160,AT1G79040,AT1G14380,AT5G01820

TCATCTTCTT	10	5	['8-12', '12-16']	80	AT3G01320,AT1G75100,AT1G63630,AT5G27010,AT5G11280,AT4G28420,AT3G62550,AT2G23200,AT3G53870,AT5G10860,AT1G78290,AT2G47450,AT4G27700,AT4G38470,AT5G58910,AT2G38120,AT3G04480,AT3G61460,AT4G38160,AT5G12480,AT5G58770,AT5G58620,AT4G12590,AT1G78180,AT5G24610,AT5G57760,AT3G07840,AT3G18600,AT4G35800,AT1G33970,AT3G22970,AT5G65910,AT5G65990,AT1G55730,AT1G19060,AT1G14685,AT5G65530,AT5G05520,AT1G33780,AT2G35190,AT1G04850,AT5G44650,AT5G55530,AT3G58140,AT1G21250,AT2G36970,AT5G64180,AT1G54270,AT2G46240,AT1G73980,AT1G22850,AT1G62290,AT1G23960,AT4G15810,AT3G27300,AT5G16150,AT5G17380,AT2G25510,AT1G10960,AT2G41720,AT5G62980,AT2G19790,AT2G47320,AT5G03150,AT3G27840,AT2G26770,AT2G03310,AT1G79790,AT2G46820,AT4G20170,AT3G55680,AT2G32910,AT2G07360,AT1G73470,AT5G41150,AT1G10510,AT3G06160,AT1G79040,AT1G14380,AT5G01820
TCCACGTGGC	10	1	['0-4', '4-8', '16-20', '20-24']	19	AT1G54850,AT5G58070,AT5G05200,AT5G65630,AT4G25570,AT3G56050,AT5G64260,AT5G52580,AT5G57345,AT2G04550,AT1G18740,AT1G01520,AT1G52230,AT1G55850,AT4G25450,AT1G56220,AT1G55670,AT3G59660,AT2G21330
TCCACGTGGC	10	2	['0-4', '4-8', '16-20', '20-24']	19	AT1G54850,AT5G58070,AT5G05200,AT5G65630,AT4G25570,AT3G56050,AT5G64260,AT5G52580,AT5G57345,AT2G04550,AT1G18740,AT1G01520,AT1G52230,AT1G55850,AT4G25450,AT1G56220,AT1G55670,AT3G59660,AT2G21330
TCCACGTGGC	10	3	['0-4', '4-8', '16-20', '20-24']	19	AT1G54850,AT5G58070,AT5G05200,AT5G65630,AT4G25570,AT3G56050,AT5G64260,AT5G52580,AT5G57345,AT2G04550,AT1G18740,AT1G01520,AT1G52230,AT1G55850,AT4G25450,AT1G56220,AT1G55670,AT3G59660,AT2G21330
TCCACGTGGC	10	4	['0-4', '4-8', '16-20', '20-24']	19	AT1G54850,AT5G58070,AT5G05200,AT5G65630,AT4G25570,AT3G56050,AT5G64260,AT5G52580,AT5G57345,AT2G04550,AT1G18740,AT1G01520,AT1G52230,AT1G55850,AT4G25450,AT1G56220,AT1G55670,AT3G59660,AT2G21330
TCCACGTGGC	10	5	['4-8', '16-20', '20-24', '0-4']	19	AT1G54850,AT5G58070,AT5G05200,AT5G65630,AT4G25570,AT3G56050,AT5G64260,AT5G52580,AT5G57345,AT2G04550,AT1G18740,AT1G01520,AT1G52230,AT1G55850,AT4G25450,AT1G56220,AT1G55670,AT3G59660,AT2G21330
TCCACGTGTC	10	1	['0-4', '4-8']	20	AT3G15430,AT1G65640,AT3G59220,AT4G39990,AT4G11600,AT3G56090,AT5G03140,AT2G35260,AT3G03160,AT3G11050,AT5G37260,AT1G10090,AT3G47160,AT4G30500,AT3G01060,AT5G13630,AT3G48530,AT1G15820,AT2G16600,AT4G18810
TCCACGTGTC	10	2	['0-4', '4-8']	20	AT3G15430,AT1G65640,AT3G59220,AT4G39990,AT4G11600,AT3G56090,AT5G03140,AT2G35260,AT3G03160,AT3G11050,AT5G37260,AT1G10090,AT3G47160,AT4G30500,AT3G01060,AT5G13630,AT3G48530,AT1G15820,AT2G16600,AT4G18810
TCCACGTGTC	10	3	['0-4', '4-8']	20	AT3G15430,AT1G65640,AT3G59220,AT4G39990,AT4G11600,AT3G56090,AT5G03140,AT2G35260,AT3G03160,AT3G11050,AT5G37260,AT1G10090,AT3G47160,AT4G30500,AT3G01060,AT5G13630,AT3G48530,AT1G15820,AT2G16600,AT4G18810
TCCACGTGTC	10	4	['0-4', '4-8']	20	AT3G15430,AT1G65640,AT3G59220,AT4G39990,AT4G11600,AT3G56090,AT5G03140,AT2G35260,AT3G03160,AT3G11050,AT5G37260,AT1G10090,AT3G47160,AT4G30500,AT3G01060,AT5G13630,AT3G48530,AT1G15820,AT2G16600,AT4G18810
TCCACGTGTC	10	5	['0-4', '4-8']	20	AT3G15430,AT1G65640,AT3G59220,AT4G39990,AT4G11600,AT3G56090,AT5G03140,AT2G35260,AT3G03160,AT3G11050,AT5G37260,AT1G10090,AT3G47160,AT4G30500,AT3G01060,AT5G13630,AT3G48530,AT1G15820,AT2G16600,AT4G18810
TCCATGCAT	10	1	['0-4', '4-8', '8-12', '12-16', '20-24']	19	AT1G07020,AT1G22690,AT4G11710,AT5G55960,AT5G64630,AT3G28715,AT3G53250,AT3G19930,AT2G23200,AT5G52580,AT2G17330,AT1G26590,AT1G80840,AT5G23350,AT5G37540,AT3G16040,AT3G26380,AT5G48900,AT4G27585
TCCATGCAT	10	2	['0-4', '4-8', '8-12', '12-16', '20-24']	19	AT1G07020,AT1G22690,AT4G11710,AT5G55960,AT5G64630,AT3G28715,AT3G53250,AT3G19930,AT2G23200,AT5G52580,AT2G17330,AT1G26590,AT1G80840,AT5G23350,AT5G37540,AT3G16040,AT3G26380,AT5G48900,AT4G27585
TCCATGCAT	10	3	['0-4', '4-8', '8-12', '12-16', '20-24']	19	AT1G07020,AT1G22690,AT4G11710,AT5G55960,AT5G64630,AT3G28715,AT3G53250,AT3G19930,AT2G23200,AT5G52580,AT2G17330,AT1G26590,AT1G80840,AT5G23350,AT5G37540,AT3G16040,AT3G26380,AT5G48900,AT4G27585
TCCATGCAT	10	4	['0-4', '4-8', '8-12', '12-16', '20-24']	19	AT1G07020,AT1G22690,AT4G11710,AT5G55960,AT5G64630,AT3G28715,AT3G53250,AT3G19930,AT2G23200,AT5G52580,AT2G17330,AT1G26590,AT1G80840,AT5G23350,AT5G37540,AT3G16040,AT3G26380,AT5G48900,AT4G27585
TCCATGCAT	10	5	['0-4', '4-8', '8-12', '12-16', '20-24']	19	AT1G07020,AT1G22690,AT4G11710,AT5G55960,AT5G64630,AT3G28715,AT3G53250,AT3G19930,AT2G23200,AT5G52580,AT2G17330,AT1G26590,AT1G80840,AT5G23350,AT5G37540,AT3G16040,AT3G26380,AT5G48900,AT4G27585

TCCGTACAA	10	1	['0-4', '4-8', '16-20', '20-24']	21	AT2G19650,AT3G62720,AT1G76580,AT2G31510,AT4G27700,AT5G59180,AT4G37680,AT1G13880,AT3G56880,AT2G40400,AT1G09795,AT4G16860,AT3G57090,AT5G63370,AT4G14230,AT3G46340,AT2G06630,AT1G14380,AT5G01890,AT3G04980,AT1G08080
TCCGTACAA	10	2	['0-4', '4-8', '16-20', '20-24']	21	AT2G19650,AT3G62720,AT1G76580,AT2G31510,AT4G27700,AT5G59180,AT4G37680,AT1G13880,AT3G56880,AT2G40400,AT1G09795,AT4G16860,AT3G57090,AT5G63370,AT4G14230,AT3G46340,AT2G06630,AT1G14380,AT5G01890,AT3G04980,AT1G08080
TCCGTACAA	10	3	['0-4', '4-8', '20-24', '16-20']	21	AT2G19650,AT3G62720,AT1G76580,AT2G31510,AT4G27700,AT5G59180,AT4G37680,AT1G13880,AT3G56880,AT2G40400,AT1G09795,AT4G16860,AT3G57090,AT5G63370,AT4G14230,AT3G46340,AT2G06630,AT1G14380,AT5G01890,AT3G04980,AT1G08080
TCCGTACAA	10	4	['0-4', '4-8', '16-20', '20-24']	21	AT2G19650,AT3G62720,AT1G76580,AT2G31510,AT4G27700,AT5G59180,AT4G37680,AT1G13880,AT3G56880,AT2G40400,AT1G09795,AT4G16860,AT3G57090,AT5G63370,AT4G14230,AT3G46340,AT2G06630,AT1G14380,AT5G01890,AT3G04980,AT1G08080
TCCGTACAA	10	5	['4-8', '16-20', '20-24', '0-4']	21	AT2G19650,AT3G62720,AT1G76580,AT2G31510,AT4G27700,AT5G59180,AT4G37680,AT1G13880,AT3G56880,AT2G40400,AT1G09795,AT4G16860,AT3G57090,AT5G63370,AT4G14230,AT3G46340,AT2G06630,AT1G14380,AT5G01890,AT3G04980,AT1G08080
TGACGTAA	10	1	['0-4', '8-12']	62	AT4G18240,AT1G67280,AT5G11480,AT2G40840,AT1G49010,AT2G31510,AT2G41120,AT1G68830,AT1G47330,AT1G51240,AT4G12800,AT3G18440,AT1G73680,AT1G75900,AT1G62780,AT3G04460,AT2G33420,AT3G51140,AT1G35460,AT1G15520,AT4G11600,AT1G21780,AT2G29150,AT5G57030,AT5G66060,AT1G01230,AT1G14770,AT5G46020,AT3G05910,AT3G59020,AT3G59140,AT4G34100,AT1G19650,AT5G64860,AT3G58750,AT5G04610,AT2G46550,AT5G64260,AT4G23630,AT5G44410,AT1G20410,AT3G15220,AT4G22750,AT1G62300,AT5G43830,AT1G63080,AT1G05850,AT4G14605,AT1G71180,AT2G32180,AT1G29400,AT1G73020,AT3G08940,AT2G32100,AT2G01290,AT5G02240,AT2G35750,AT1G55850,AT5G40850,AT5G28140,AT3G16180,AT5G12150
TGACGTAA	10	2	['0-4', '8-12']	62	AT4G18240,AT1G67280,AT5G11480,AT2G40840,AT1G49010,AT2G31510,AT2G41120,AT1G68830,AT1G47330,AT1G51240,AT4G12800,AT3G18440,AT1G73680,AT1G75900,AT1G62780,AT3G04460,AT2G33420,AT3G51140,AT1G35460,AT1G15520,AT4G11600,AT1G21780,AT2G29150,AT5G57030,AT5G66060,AT1G01230,AT1G14770,AT5G46020,AT3G05910,AT3G59020,AT3G59140,AT4G34100,AT1G19650,AT5G64860,AT3G58750,AT5G04610,AT2G46550,AT5G64260,AT4G23630,AT5G44410,AT1G20410,AT3G15220,AT4G22750,AT1G62300,AT5G43830,AT1G63080,AT1G05850,AT4G14605,AT1G71180,AT2G32180,AT1G29400,AT1G73020,AT3G08940,AT2G32100,AT2G01290,AT5G02240,AT2G35750,AT1G55850,AT5G40850,AT5G28140,AT3G16180,AT5G12150
TGACGTAA	10	3	['0-4', '8-12']	62	AT4G18240,AT1G67280,AT5G11480,AT2G40840,AT1G49010,AT2G31510,AT2G41120,AT1G68830,AT1G47330,AT1G51240,AT4G12800,AT3G18440,AT1G73680,AT1G75900,AT1G62780,AT3G04460,AT2G33420,AT3G51140,AT1G35460,AT1G15520,AT4G11600,AT1G21780,AT2G29150,AT5G57030,AT5G66060,AT1G01230,AT1G14770,AT5G46020,AT3G05910,AT3G59020,AT3G59140,AT4G34100,AT1G19650,AT5G64860,AT3G58750,AT5G04610,AT2G46550,AT5G64260,AT4G23630,AT5G44410,AT1G20410,AT3G15220,AT4G22750,AT1G62300,AT5G43830,AT1G63080,AT1G05850,AT4G14605,AT1G71180,AT2G32180,AT1G29400,AT1G73020,AT3G08940,AT2G32100,AT2G01290,AT5G02240,AT2G35750,AT1G55850,AT5G40850,AT5G28140,AT3G16180,AT5G12150
TGACGTAA	10	4	['0-4', '8-12']	62	AT4G18240,AT1G67280,AT5G11480,AT2G40840,AT1G49010,AT2G31510,AT2G41120,AT1G68830,AT1G47330,AT1G51240,AT4G12800,AT3G18440,AT1G73680,AT1G75900,AT1G62780,AT3G04460,AT2G33420,AT3G51140,AT1G35460,AT1G15520,AT4G11600,AT1G21780,AT2G29150,AT5G57030,AT5G66060,AT1G01230,AT1G14770,AT5G46020,AT3G05910,AT3G59020,AT3G59140,AT4G34100,AT1G19650,AT5G64860,AT3G58750,AT5G04610,AT2G46550,AT5G64260,AT4G23630,AT5G44410,AT1G20410,AT3G15220,AT4G22750,AT1G62300,AT5G43830,AT1G63080,AT1G05850,AT4G14605,AT1G71180,AT2G32180,AT1G29400,AT1G73020,AT3G08940,AT2G32100,AT2G01290,AT5G02240,AT2G35750,AT1G55850,AT5G40850,AT5G28140,AT3G16180,AT5G12150

TGACGTAA	10	5	['8-12', '0-4']	62	AT4G18240,AT1G67280,AT5G11480,AT2G40840,AT1G49010,AT2G31510,AT2G41120,AT1G68830,AT1G47330,AT1G51240,AT4G12800,AT3G18440,AT1G73680,AT1G75900,AT1G62780,AT3G04460,AT2G33420,AT3G51140,AT1G35460,AT1G15520,AT4G11600,AT1G21780,AT2G29150,AT5G57030,AT5G66060,AT1G01230,AT1G14770,AT5G46020,AT3G05910,AT3G59020,AT3G59140,AT4G34100,AT1G19650,AT5G64860,AT3G58750,AT5G04610,AT2G46550,AT5G64260,AT4G23630,AT5G44410,AT1G20410,AT3G15220,AT4G22750,AT1G62300,AT5G43830,AT1G63080,AT1G05850,AT4G14605,AT1G71180,AT2G32180,AT1G29400,AT1G73020,AT3G08940,AT2G32100,AT2G01290,AT5G02240,AT2G35750,AT1G55850,AT5G40850,AT5G28140,AT3G16180,AT5G12150
TGACGTCA	10	1	['20-24']	67	AT5G27520,AT2G45740,AT3G05480,AT2G23880,AT1G15290,AT2G39400,AT5G11060,AT2G23200,AT5G49990,AT4G00370,AT3G62860,AT3G09010,AT1G78670,AT3G53030,AT3G52070,AT5G25210,AT2G45420,AT5G25070,AT3G05270,AT3G04460,AT5G48230,AT2G42580,AT1G06010,AT5G24530,AT5G66770,AT2G18240,AT1G70730,AT2G14660,AT2G01680,AT2G18230,AT1G74560,AT1G14920,AT1G15820,AT1G07280,AT5G56170,AT1G62890,AT3G11780,AT1G80530,AT1G16880,AT3G19450,AT5G54540,AT1G69690,AT2G14900,AT3G30810,AT4G23060,AT5G16970,AT3G57020,AT3G57040,AT3G56410,AT2G16070,AT5G03350,AT3G56660,AT1G68670,AT2G29780,AT1G73060,AT3G16520,AT3G01850,AT2G43710,AT1G05720,AT2G32910,AT2G47010,AT3G03420,AT5G61820,AT3G26520,AT1G21680,AT1G60590,AT3G63520
TGACGTCA	10	2	['20-24']	67	AT5G27520,AT2G45740,AT3G05480,AT2G23880,AT1G15290,AT2G39400,AT5G11060,AT2G23200,AT5G49990,AT4G00370,AT3G62860,AT3G09010,AT1G78670,AT3G53030,AT3G52070,AT5G25210,AT2G45420,AT5G25070,AT3G05270,AT3G04460,AT5G48230,AT2G42580,AT1G06010,AT5G24530,AT5G66770,AT2G18240,AT1G70730,AT2G14660,AT2G01680,AT2G18230,AT1G74560,AT1G14920,AT1G15820,AT1G07280,AT5G56170,AT1G62890,AT3G11780,AT1G80530,AT1G16880,AT3G19450,AT5G54540,AT1G69690,AT2G14900,AT3G30810,AT4G23060,AT5G16970,AT3G57020,AT3G57040,AT3G56410,AT2G16070,AT5G03350,AT3G56660,AT1G68670,AT2G29780,AT1G73060,AT3G16520,AT3G01850,AT2G43710,AT1G05720,AT2G32910,AT2G47010,AT3G03420,AT5G61820,AT3G26520,AT1G21680,AT1G60590,AT3G63520
TGACGTCA	10	3	['20-24']	67	AT5G27520,AT2G45740,AT3G05480,AT2G23880,AT1G15290,AT2G39400,AT5G11060,AT2G23200,AT5G49990,AT4G00370,AT3G62860,AT3G09010,AT1G78670,AT3G53030,AT3G52070,AT5G25210,AT2G45420,AT5G25070,AT3G05270,AT3G04460,AT5G48230,AT2G42580,AT1G06010,AT5G24530,AT5G66770,AT2G18240,AT1G70730,AT2G14660,AT2G01680,AT2G18230,AT1G74560,AT1G14920,AT1G15820,AT1G07280,AT5G56170,AT1G62890,AT3G11780,AT1G80530,AT1G16880,AT3G19450,AT5G54540,AT1G69690,AT2G14900,AT3G30810,AT4G23060,AT5G16970,AT3G57020,AT3G57040,AT3G56410,AT2G16070,AT5G03350,AT3G56660,AT1G68670,AT2G29780,AT1G73060,AT3G16520,AT3G01850,AT2G43710,AT1G05720,AT2G32910,AT2G47010,AT3G03420,AT5G61820,AT3G26520,AT1G21680,AT1G60590,AT3G63520
TGACGTCA	10	4	['20-24']	67	AT5G27520,AT2G45740,AT3G05480,AT2G23880,AT1G15290,AT2G39400,AT5G11060,AT2G23200,AT5G49990,AT4G00370,AT3G62860,AT3G09010,AT1G78670,AT3G53030,AT3G52070,AT5G25210,AT2G45420,AT5G25070,AT3G05270,AT3G04460,AT5G48230,AT2G42580,AT1G06010,AT5G24530,AT5G66770,AT2G18240,AT1G70730,AT2G14660,AT2G01680,AT2G18230,AT1G74560,AT1G14920,AT1G15820,AT1G07280,AT5G56170,AT1G62890,AT3G11780,AT1G80530,AT1G16880,AT3G19450,AT5G54540,AT1G69690,AT2G14900,AT3G30810,AT4G23060,AT5G16970,AT3G57020,AT3G57040,AT3G56410,AT2G16070,AT5G03350,AT3G56660,AT1G68670,AT2G29780,AT1G73060,AT3G16520,AT3G01850,AT2G43710,AT1G05720,AT2G32910,AT2G47010,AT3G03420,AT5G61820,AT3G26520,AT1G21680,AT1G60590,AT3G63520
TGACGTCA	10	5	['20-24']	67	AT5G27520,AT2G45740,AT3G05480,AT2G23880,AT1G15290,AT2G39400,AT5G11060,AT2G23200,AT5G49990,AT4G00370,AT3G62860,AT3G09010,AT1G78670,AT3G53030,AT3G52070,AT5G25210,AT2G45420,AT5G25070,AT3G05270,AT3G04460,AT5G48230,AT2G42580,AT1G06010,AT5G24530,AT5G66770,AT2G18240,AT1G70730,AT2G14660,AT2G01680,AT2G18230,AT1G74560,AT1G14920,AT1G15820,AT1G07280,AT5G56170,AT1G62890,AT3G11780,AT1G80530,AT1G16880,AT3G19450,AT5G54540,AT1G69690,AT2G14900,AT3G30810,AT4G23060,AT5G16970,AT3G57020,AT3G57040,AT3G56410,AT2G16070,AT5G03350,AT3G56660,AT1G68670,AT2G29780,AT1G73060,AT3G16520,AT3G01850,AT2G43710,AT1G05720,AT2G32910,AT2G47010,AT3G03420,AT5G61820,AT3G26520,AT1G21680,AT1G60590,AT3G63520

TGACGTGG	10	1	['12-16']	83	AT3G63160,AT2G37240,AT5G51010,AT2G37450,AT1G34220,AT1G22750,AT3G16690,AT4G39100,AT5G19850,AT1G55510,AT1G18330,AT5G49540,AT1G06680,AT3G07680,AT4G13010,AT1G76030,AT2G31790,AT1G73650,AT5G36170,AT5G24810,AT3G04460,AT2G34090,AT5G58070,AT1G59218,AT1G58807,AT5G19940,AT4G37480,AT3G22200,AT3G50240,AT2G41410,AT3G17000,AT1G44000,AT4G15560,AT3G22890,AT1G07140,AT4G34720,AT1G10070,AT3G15360,AT2G18230,AT1G17460,AT5G65430,AT3G13510,AT1G20696,AT5G64840,AT1G80310,AT5G18850,AT1G78020,AT4G33980,AT4G17140,AT5G17990,AT5G03905,AT3G03180,AT5G54770,AT3G46780,AT3G57550,AT5G17660,AT1G69830,AT2G28200,AT5G17310,AT5G59570,AT1G08720,AT5G16715,AT3G56490,AT4G03600,AT5G02810,AT1G29390,AT1G78480,AT5G02820,AT1G79790,AT5G15510,AT1G29395,AT3G01860,AT3G44950,AT1G05570,AT5G41050,AT1G23205,AT5G35180,AT3G15580,AT5G61380,AT3G54500,AT1G76730,AT1G02020,AT1G32130
TGACGTGG	10	2	['12-16']	83	AT3G63160,AT2G37240,AT5G51010,AT2G37450,AT1G34220,AT1G22750,AT3G16690,AT4G39100,AT5G19850,AT1G55510,AT1G18330,AT5G49540,AT1G06680,AT3G07680,AT4G13010,AT1G76030,AT2G31790,AT1G73650,AT5G36170,AT5G24810,AT3G04460,AT2G34090,AT5G58070,AT1G59218,AT1G58807,AT5G19940,AT4G37480,AT3G22200,AT3G50240,AT2G41410,AT3G17000,AT1G44000,AT4G15560,AT3G22890,AT1G07140,AT4G34720,AT1G10070,AT3G15360,AT2G18230,AT1G17460,AT5G65430,AT3G13510,AT1G20696,AT5G64840,AT1G80310,AT5G18850,AT1G78020,AT4G33980,AT4G17140,AT5G17990,AT5G03905,AT3G03180,AT5G54770,AT3G46780,AT3G57550,AT5G17660,AT1G69830,AT2G28200,AT5G17310,AT5G59570,AT1G08720,AT5G16715,AT3G56490,AT4G03600,AT5G02810,AT1G29390,AT1G78480,AT5G02820,AT1G79790,AT5G15510,AT1G29395,AT3G01860,AT3G44950,AT1G05570,AT5G41050,AT1G23205,AT5G35180,AT3G15580,AT5G61380,AT3G54500,AT1G76730,AT1G02020,AT1G32130
TGACGTGG	10	3	['12-16']	83	AT3G63160,AT2G37240,AT5G51010,AT2G37450,AT1G34220,AT1G22750,AT3G16690,AT4G39100,AT5G19850,AT1G55510,AT1G18330,AT5G49540,AT1G06680,AT3G07680,AT4G13010,AT1G76030,AT2G31790,AT1G73650,AT5G36170,AT5G24810,AT3G04460,AT2G34090,AT5G58070,AT1G59218,AT1G58807,AT5G19940,AT4G37480,AT3G22200,AT3G50240,AT2G41410,AT3G17000,AT1G44000,AT4G15560,AT3G22890,AT1G07140,AT4G34720,AT1G10070,AT3G15360,AT2G18230,AT1G17460,AT5G65430,AT3G13510,AT1G20696,AT5G64840,AT1G80310,AT5G18850,AT1G78020,AT4G33980,AT4G17140,AT5G17990,AT5G03905,AT3G03180,AT5G54770,AT3G46780,AT3G57550,AT5G17660,AT1G69830,AT2G28200,AT5G17310,AT5G59570,AT1G08720,AT5G16715,AT3G56490,AT4G03600,AT5G02810,AT1G29390,AT1G78480,AT5G02820,AT1G79790,AT5G15510,AT1G29395,AT3G01860,AT3G44950,AT1G05570,AT5G41050,AT1G23205,AT5G35180,AT3G15580,AT5G61380,AT3G54500,AT1G76730,AT1G02020,AT1G32130
TGACGTGG	10	4	['12-16']	83	AT3G63160,AT2G37240,AT5G51010,AT2G37450,AT1G34220,AT1G22750,AT3G16690,AT4G39100,AT5G19850,AT1G55510,AT1G18330,AT5G49540,AT1G06680,AT3G07680,AT4G13010,AT1G76030,AT2G31790,AT1G73650,AT5G36170,AT5G24810,AT3G04460,AT2G34090,AT5G58070,AT1G59218,AT1G58807,AT5G19940,AT4G37480,AT3G22200,AT3G50240,AT2G41410,AT3G17000,AT1G44000,AT4G15560,AT3G22890,AT1G07140,AT4G34720,AT1G10070,AT3G15360,AT2G18230,AT1G17460,AT5G65430,AT3G13510,AT1G20696,AT5G64840,AT1G80310,AT5G18850,AT1G78020,AT4G33980,AT4G17140,AT5G17990,AT5G03905,AT3G03180,AT5G54770,AT3G46780,AT3G57550,AT5G17660,AT1G69830,AT2G28200,AT5G17310,AT5G59570,AT1G08720,AT5G16715,AT3G56490,AT4G03600,AT5G02810,AT1G29390,AT1G78480,AT5G02820,AT1G79790,AT5G15510,AT1G29395,AT3G01860,AT3G44950,AT1G05570,AT5G41050,AT1G23205,AT5G35180,AT3G15580,AT5G61380,AT3G54500,AT1G76730,AT1G02020,AT1G32130

TGACGTGG	10	5	['12-16']	83	AT3G63160,AT2G37240,AT5G51010,AT2G37450,AT1G34220,AT1G22750,AT3G16690,AT4G39100,AT5G19850,AT1G55510,AT1G18330,AT5G49540,AT1G06680,AT3G07680,AT4G13010,AT1G76030,AT2G31790,AT1G73650,AT5G36170,AT5G24810,AT3G04460,AT2G34090,AT5G58070,AT1G59218,AT1G58807,AT5G19940,AT4G37480,AT3G22200,AT3G50240,AT2G41410,AT3G17000,AT1G44000,AT4G15560,AT3G22890,AT1G07140,AT4G34720,AT1G10070,AT3G15360,AT2G18230,AT1G17460,AT5G65430,AT3G13510,AT1G20696,AT5G64840,AT1G80310,AT5G18850,AT1G78020,AT4G33980,AT4G17140,AT5G17990,AT5G03905,AT3G03180,AT5G54770,AT3G46780,AT3G57550,AT5G17660,AT1G69830,AT2G28200,AT5G17310,AT5G59570,AT1G08720,AT5G16715,AT3G56490,AT4G03600,AT5G02810,AT1G29390,AT1G78480,AT5G02820,AT1G79790,AT5G15510,AT1G29395,AT3G01860,AT3G44950,AT1G05570,AT5G41050,AT1G23205,AT5G35180,AT3G15580,AT5G61380,AT3G54500,AT1G76730,AT1G02020,AT1G32130
TGACGTGGC	10	1	['12-16', '20-24']	45	AT2G37240,AT5G51010,AT2G37450,AT1G22750,AT3G54500,AT1G55510,AT1G18330,AT5G49540,AT3G07680,AT4G13010,AT1G76030,AT5G24810,AT2G31790,AT5G36170,AT3G04460,AT5G19940,AT4G37480,AT3G50240,AT3G22890,AT4G15560,AT1G10070,AT1G07140,AT1G17460,AT5G65430,AT1G20696,AT5G18850,AT1G78020,AT4G33980,AT4G17140,AT5G17990,AT5G54770,AT3G46780,AT5G17660,AT1G69830,AT5G59570,AT3G56490,AT4G03600,AT5G15510,AT3G01860,AT3G44950,AT1G05570,AT5G41050,AT5G61380,AT1G32130,AT3G15360
TGACGTGGC	10	2	['20-24', '12-16']	45	AT2G37240,AT5G51010,AT2G37450,AT1G22750,AT3G54500,AT1G55510,AT1G18330,AT5G49540,AT3G07680,AT4G13010,AT1G76030,AT5G24810,AT2G31790,AT5G36170,AT3G04460,AT5G19940,AT4G37480,AT3G50240,AT3G22890,AT4G15560,AT1G10070,AT1G07140,AT1G17460,AT5G65430,AT1G20696,AT5G18850,AT1G78020,AT4G33980,AT4G17140,AT5G17990,AT5G54770,AT3G46780,AT5G17660,AT1G69830,AT5G59570,AT3G56490,AT4G03600,AT5G15510,AT3G01860,AT3G44950,AT1G05570,AT5G41050,AT5G61380,AT1G32130,AT3G15360
TGACGTGGC	10	3	['12-16', '20-24']	45	AT2G37240,AT5G51010,AT2G37450,AT1G22750,AT3G54500,AT1G55510,AT1G18330,AT5G49540,AT3G07680,AT4G13010,AT1G76030,AT5G24810,AT2G31790,AT5G36170,AT3G04460,AT5G19940,AT4G37480,AT3G50240,AT3G22890,AT4G15560,AT1G10070,AT1G07140,AT1G17460,AT5G65430,AT1G20696,AT5G18850,AT1G78020,AT4G33980,AT4G17140,AT5G17990,AT5G54770,AT3G46780,AT5G17660,AT1G69830,AT5G59570,AT3G56490,AT4G03600,AT5G15510,AT3G01860,AT3G44950,AT1G05570,AT5G41050,AT5G61380,AT1G32130,AT3G15360
TGACGTGGC	10	4	['12-16', '20-24']	45	AT2G37240,AT5G51010,AT2G37450,AT1G22750,AT3G54500,AT1G55510,AT1G18330,AT5G49540,AT3G07680,AT4G13010,AT1G76030,AT5G24810,AT2G31790,AT5G36170,AT3G04460,AT5G19940,AT4G37480,AT3G50240,AT3G22890,AT4G15560,AT1G10070,AT1G07140,AT1G17460,AT5G65430,AT1G20696,AT5G18850,AT1G78020,AT4G33980,AT4G17140,AT5G17990,AT5G54770,AT3G46780,AT5G17660,AT1G69830,AT5G59570,AT3G56490,AT4G03600,AT5G15510,AT3G01860,AT3G44950,AT1G05570,AT5G41050,AT5G61380,AT1G32130,AT3G15360
TGACGTGGC	10	5	['12-16', '20-24']	45	AT2G37240,AT5G51010,AT2G37450,AT1G22750,AT3G54500,AT1G55510,AT1G18330,AT5G49540,AT3G07680,AT4G13010,AT1G76030,AT5G24810,AT2G31790,AT5G36170,AT3G04460,AT5G19940,AT4G37480,AT3G50240,AT3G22890,AT4G15560,AT1G10070,AT1G07140,AT1G17460,AT5G65430,AT1G20696,AT5G18850,AT1G78020,AT4G33980,AT4G17140,AT5G17990,AT5G54770,AT3G46780,AT5G17660,AT1G69830,AT5G59570,AT3G56490,AT4G03600,AT5G15510,AT3G01860,AT3G44950,AT1G05570,AT5G41050,AT5G61380,AT1G32130,AT3G15360
TGCCGACAA	10	1	['0-4', '4-8', '8-12', '12-16', '20-24']	11	AT3G13100,AT5G07960,AT5G53280,AT1G06010,AT1G49270,AT5G66920,AT3G19970,AT5G16260,AT4G32410,AT2G25900,AT5G15740
TGCCGACAA	10	2	['0-4', '4-8', '8-12', '12-16', '20-24']	11	AT3G13100,AT5G07960,AT5G53280,AT1G06010,AT1G49270,AT5G66920,AT3G19970,AT5G16260,AT4G32410,AT2G25900,AT5G15740
TGCCGACAA	10	3	['0-4', '4-8', '8-12', '12-16', '20-24']	11	AT3G13100,AT5G07960,AT5G53280,AT1G06010,AT1G49270,AT5G66920,AT3G19970,AT5G16260,AT4G32410,AT2G25900,AT5G15740

TGCCGACAA	10	4	['0-4', '4-8', '8-12', '12-16', '20-24']	11	AT3G13100,AT5G07960,AT5G53280,AT1G06010,AT1G49270,AT5G66920,AT3G19970,AT5G16260,AT4G32410,AT2G25900,AT5G15740
TGCCGACAA	10	5	['0-4', '4-8', '8-12', '12-16', '20-24']	11	AT3G13100,AT5G07960,AT5G53280,AT1G06010,AT1G49270,AT5G66920,AT3G19970,AT5G16260,AT4G32410,AT2G25900,AT5G15740
TGCCGACAT	10	1	['0-4', '4-8', '8-12', '12-16', '20-24']	12	AT5G54110,AT5G63370,AT2G20830,AT1G69160,AT3G55760,AT2G15970,AT1G78010,AT1G69880,AT3G17000,AT3G02750,AT4G32410,AT1G01790
TGCCGACAT	10	2	['0-4', '4-8', '8-12', '12-16', '20-24']	12	AT5G54110,AT5G63370,AT2G20830,AT1G69160,AT3G55760,AT2G15970,AT1G78010,AT1G69880,AT3G17000,AT3G02750,AT4G32410,AT1G01790
TGCCGACAT	10	3	['0-4', '4-8', '8-12', '12-16', '20-24']	12	AT5G54110,AT5G63370,AT2G20830,AT1G69160,AT3G55760,AT2G15970,AT1G78010,AT1G69880,AT3G17000,AT3G02750,AT4G32410,AT1G01790
TGCCGACAT	10	4	['0-4', '4-8', '8-12', '12-16', '20-24']	12	AT5G54110,AT5G63370,AT2G20830,AT1G69160,AT3G55760,AT2G15970,AT1G78010,AT1G69880,AT3G17000,AT3G02750,AT4G32410,AT1G01790
TGCCGACAT	10	5	['0-4', '4-8', '8-12', '12-16', '20-24']	12	AT5G54110,AT5G63370,AT2G20830,AT1G69160,AT3G55760,AT2G15970,AT1G78010,AT1G69880,AT3G17000,AT3G02750,AT4G32410,AT1G01790
TGCCGACTA	10	1	['0-4', '8-12', '12-16', '16-20']	10	AT5G08380,AT1G65490,AT2G24930,AT2G47400,AT4G28610,AT2G26530,AT2G18770,AT5G23080,AT5G43150,AT1G02020
TGCCGACTA	10	2	['0-4', '8-12', '12-16', '16-20']	10	AT5G08380,AT1G65490,AT2G24930,AT2G47400,AT4G28610,AT2G26530,AT2G18770,AT5G23080,AT5G43150,AT1G02020
TGCCGACTA	10	3	['0-4', '8-12', '12-16', '16-20']	10	AT5G08380,AT1G65490,AT2G24930,AT2G47400,AT4G28610,AT2G26530,AT2G18770,AT5G23080,AT5G43150,AT1G02020
TGCCGACTA	10	4	['0-4', '8-12', '12-16', '16-20']	10	AT5G08380,AT1G65490,AT2G24930,AT2G47400,AT4G28610,AT2G26530,AT2G18770,AT5G23080,AT5G43150,AT1G02020
TGCCGACTA	10	5	['0-4', '8-12', '12-16', '16-20']	10	AT5G08380,AT1G65490,AT2G24930,AT2G47400,AT4G28610,AT2G26530,AT2G18770,AT5G23080,AT5G43150,AT1G02020
TGCGTACAA	10	1	['4-8', '12-16', '20-24']	11	AT5G19290,AT1G16470,AT5G63380,AT5G48030,AT3G01850,AT2G26170,AT2G28000,AT2G32910,AT3G22970,AT1G19060,AT1G73650
TGCGTACAA	10	2	['4-8', '20-24', '12-16']	11	AT5G19290,AT1G16470,AT5G63380,AT5G48030,AT3G01850,AT2G26170,AT2G28000,AT2G32910,AT3G22970,AT1G19060,AT1G73650
TGCGTACAA	10	3	['4-8', '20-24', '12-16']	11	AT5G19290,AT1G16470,AT5G63380,AT5G48030,AT3G01850,AT2G26170,AT2G28000,AT2G32910,AT3G22970,AT1G19060,AT1G73650
TGCGTACAA	10	4	['4-8', '20-24']	11	AT5G19290,AT1G16470,AT5G63380,AT5G48030,AT3G01850,AT2G26170,AT2G28000,AT2G32910,AT3G22970,AT1G19060,AT1G73650
TGCGTACAA	10	5	['4-8', '12-16', '20-24']	11	AT5G19290,AT1G16470,AT5G63380,AT5G48030,AT3G01850,AT2G26170,AT2G28000,AT2G32910,AT3G22970,AT1G19060,AT1G73650

TGGACGG	10	1	['8-12', '16-20']	97	AT3G54050,ATCG00470,AT3G53990,AT3G53470,AT4G00050,AT2G18770,AT2G37970,AT3G52500,AT3G52470,AT3G51430,AT1G13270,AT5G47620,AT1G22360,AT4G17615,AT5G47350,AT4G17070,AT2G01680,AT4G14960,AT4G16520,AT1G51805,AT2G23840,AT5G44010,AT3G12350,AT4G14440,AT5G41600,AT3G11050,AT2G39480,AT4G38940,AT5G37290,AT2G41430,AT5G35970,AT5G35540,AT1G66640,AT5G20935,AT5G28410,AT2G25250,AT1G52870,AT4G36470,AT1G52890,AT1G26940,AT3G18610,AT4G34490,AT3G13430,AT2G04550,AT4G33500,AT3G30810,AT2G35880,AT3G24260,AT2G32640,AT5G15450,AT5G16400,AT4G31310,AT4G30900,AT5G14550,AT3G26520,AT4G30060,AT5G26790,AT5G25210,AT4G27470,AT4G27130,AT3G23030,AT3G23080,AT2G25190,AT5G07400,AT5G66410,AT3G17000,AT5G04590,AT1G70210,AT5G03430,AT1G08570,AT5G63820,AT3G21650,AT3G63060,AT1G01080,AT1G24440,AT1G01940,AT1G03900,AT1G55510,AT3G02590,AT1G18460,AT3G05970,AT3G04480,AT3G61580,AT3G61430,AT3G61080,AT1G76150,AT5G57070,AT5G55620,AT2G15960,AT2G27470,AT3G56200,AT2G01350,AT3G03380,AT3G03320,AT1G21780,AT1G21680,AT5G51300
TGGACGG	10	2	['8-12', '16-20']	97	AT3G54050,ATCG00470,AT3G53990,AT3G53470,AT4G00050,AT2G18770,AT2G37970,AT3G52500,AT3G52470,AT3G51430,AT1G13270,AT5G47620,AT1G22360,AT4G17615,AT5G47350,AT4G17070,AT2G01680,AT4G14960,AT4G16520,AT1G51805,AT2G23840,AT5G44010,AT3G12350,AT4G14440,AT5G41600,AT3G11050,AT2G39480,AT4G38940,AT5G37290,AT2G41430,AT5G35970,AT5G35540,AT1G66640,AT5G20935,AT5G28410,AT2G25250,AT1G52870,AT4G36470,AT1G52890,AT1G26940,AT3G18610,AT4G34490,AT3G13430,AT2G04550,AT4G33500,AT3G30810,AT2G35880,AT3G24260,AT2G32640,AT5G15450,AT5G16400,AT4G31310,AT4G30900,AT5G14550,AT3G26520,AT4G30060,AT5G26790,AT5G25210,AT4G27470,AT4G27130,AT3G23030,AT3G23080,AT2G25190,AT5G07400,AT5G66410,AT3G17000,AT5G04590,AT1G70210,AT5G03430,AT1G08570,AT5G63820,AT3G21650,AT3G63060,AT1G01080,AT1G24440,AT1G01940,AT1G03900,AT1G55510,AT3G02590,AT1G18460,AT3G05970,AT3G04480,AT3G61580,AT3G61430,AT3G61080,AT1G76150,AT5G57070,AT5G55620,AT2G15960,AT2G27470,AT3G56200,AT2G01350,AT3G03380,AT3G03320,AT1G21780,AT1G21680,AT5G51300
TGGACGG	10	3	['8-12', '16-20']	97	AT3G54050,ATCG00470,AT3G53990,AT3G53470,AT4G00050,AT2G18770,AT2G37970,AT3G52500,AT3G52470,AT3G51430,AT1G13270,AT5G47620,AT1G22360,AT4G17615,AT5G47350,AT4G17070,AT2G01680,AT4G14960,AT4G16520,AT1G51805,AT2G23840,AT5G44010,AT3G12350,AT4G14440,AT5G41600,AT3G11050,AT2G39480,AT4G38940,AT5G37290,AT2G41430,AT5G35970,AT5G35540,AT1G66640,AT5G20935,AT5G28410,AT2G25250,AT1G52870,AT4G36470,AT1G52890,AT1G26940,AT3G18610,AT4G34490,AT3G13430,AT2G04550,AT4G33500,AT3G30810,AT2G35880,AT3G24260,AT2G32640,AT5G15450,AT5G16400,AT4G31310,AT4G30900,AT5G14550,AT3G26520,AT4G30060,AT5G26790,AT5G25210,AT4G27470,AT4G27130,AT3G23030,AT3G23080,AT2G25190,AT5G07400,AT5G66410,AT3G17000,AT5G04590,AT1G70210,AT5G03430,AT1G08570,AT5G63820,AT3G21650,AT3G63060,AT1G01080,AT1G24440,AT1G01940,AT1G03900,AT1G55510,AT3G02590,AT1G18460,AT3G05970,AT3G04480,AT3G61580,AT3G61430,AT3G61080,AT1G76150,AT5G57070,AT5G55620,AT2G15960,AT2G27470,AT3G56200,AT2G01350,AT3G03380,AT3G03320,AT1G21780,AT1G21680,AT5G51300
TGGACGG	10	4	['8-12', '16-20']	97	AT3G54050,ATCG00470,AT3G53990,AT3G53470,AT4G00050,AT2G18770,AT2G37970,AT3G52500,AT3G52470,AT3G51430,AT1G13270,AT5G47620,AT1G22360,AT4G17615,AT5G47350,AT4G17070,AT2G01680,AT4G14960,AT4G16520,AT1G51805,AT2G23840,AT5G44010,AT3G12350,AT4G14440,AT5G41600,AT3G11050,AT2G39480,AT4G38940,AT5G37290,AT2G41430,AT5G35970,AT5G35540,AT1G66640,AT5G20935,AT5G28410,AT2G25250,AT1G52870,AT4G36470,AT1G52890,AT1G26940,AT3G18610,AT4G34490,AT3G13430,AT2G04550,AT4G33500,AT3G30810,AT2G35880,AT3G24260,AT2G32640,AT5G15450,AT5G16400,AT4G31310,AT4G30900,AT5G14550,AT3G26520,AT4G30060,AT5G26790,AT5G25210,AT4G27470,AT4G27130,AT3G23030,AT3G23080,AT2G25190,AT5G07400,AT5G66410,AT3G17000,AT5G04590,AT1G70210,AT5G03430,AT1G08570,AT5G63820,AT3G21650,AT3G63060,AT1G01080,AT1G24440,AT1G01940,AT1G03900,AT1G55510,AT3G02590,AT1G18460,AT3G05970,AT3G04480,AT3G61580,AT3G61430,AT3G61080,AT1G76150,AT5G57070,AT5G55620,AT2G15960,AT2G27470,AT3G56200,AT2G01350,AT3G03380,AT3G03320,AT1G21780,AT1G21680,AT5G51300

TGGACGG	10	5	['8-12', '16-20']	97	AT3G54050,ATCG00470,AT3G53990,AT3G53470,AT4G00050,AT2G18770,AT2G37970,AT3G52500,AT3G52470,AT3G51430,AT1G13270,AT5G47620,AT1G22360,AT4G17615,AT5G47350,AT4G17070,AT2G01680,AT4G14960,AT4G16520,AT1G51805,AT2G23840,AT5G44010,AT3G12350,AT4G14440,AT5G41600,AT3G11050,AT2G39480,AT4G38940,AT5G37290,AT2G41430,AT5G35970,AT5G35540,AT1G66640,AT5G20935,AT5G28410,AT2G25250,AT1G52870,AT4G36470,AT1G52890,AT1G26940,AT3G18610,AT4G34490,AT3G13430,AT2G04550,AT4G33500,AT3G30810,AT2G35880,AT3G24260,AT2G32640,AT5G15450,AT5G16400,AT4G31310,AT4G30900,AT5G14550,AT3G26520,AT4G30060,AT5G26790,AT5G25210,AT4G27470,AT4G27130,AT3G23030,AT3G23080,AT2G25190,AT5G07400,AT5G66410,AT3G17000,AT5G04590,AT1G70210,AT5G03430,AT1G08570,AT5G63820,AT3G21650,AT3G63060,AT1G01080,AT1G24440,AT1G01940,AT1G03900,AT1G55510,AT3G02590,AT1G18460,AT3G05970,AT3G04480,AT3G61580,AT3G61430,AT3G61080,AT1G76150,AT5G57070,AT5G55620,AT2G15960,AT2G27470,AT3G56200,AT2G01350,AT3G03380,AT3G03320,AT1G21780,AT1G21680,AT5G51300
TGGCCGAC	10	1	['0-4', '8-12', '12-16', '20-24']	21	ATMG00650,AT1G67360,AT1G32470,AT2G26460,AT1G76270,AT1G71720,AT3G05940,AT1G15820,AT3G10850,AT1G20693,AT4G23940,AT4G33640,AT1G54410,AT5G17660,AT5G63200,AT5G63370,AT4G14440,AT5G62220,AT3G54890,AT5G61990,AT2G45990
TGGCCGAC	10	2	['0-4', '8-12', '12-16', '20-24']	21	ATMG00650,AT1G67360,AT1G32470,AT2G26460,AT1G76270,AT1G71720,AT3G05940,AT1G15820,AT3G10850,AT1G20693,AT4G23940,AT4G33640,AT1G54410,AT5G17660,AT5G63200,AT5G63370,AT4G14440,AT5G62220,AT3G54890,AT5G61990,AT2G45990
TGGCCGAC	10	3	['0-4', '8-12', '12-16', '20-24']	21	ATMG00650,AT1G67360,AT1G32470,AT2G26460,AT1G76270,AT1G71720,AT3G05940,AT1G15820,AT3G10850,AT1G20693,AT4G23940,AT4G33640,AT1G54410,AT5G17660,AT5G63200,AT5G63370,AT4G14440,AT5G62220,AT3G54890,AT5G61990,AT2G45990
TGGCCGAC	10	4	['0-4', '8-12', '12-16', '20-24']	21	ATMG00650,AT1G67360,AT1G32470,AT2G26460,AT1G76270,AT1G71720,AT3G05940,AT1G15820,AT3G10850,AT1G20693,AT4G23940,AT4G33640,AT1G54410,AT5G17660,AT5G63200,AT5G63370,AT4G14440,AT5G62220,AT3G54890,AT5G61990,AT2G45990
TGGCCGAC	10	5	['0-4', '8-12', '12-16', '20-24']	21	ATMG00650,AT1G67360,AT1G32470,AT2G26460,AT1G76270,AT1G71720,AT3G05940,AT1G15820,AT3G10850,AT1G20693,AT4G23940,AT4G33640,AT1G54410,AT5G17660,AT5G63200,AT5G63370,AT4G14440,AT5G62220,AT3G54890,AT5G61990,AT2G45990

TGTAAAGT	10	1	['4-8']	236	AT5G51010,AT2G18790,AT3G52610,AT3G54240,AT3G52950,AT2G47610,AT3G53030,AT3G07700,AT2G37830,AT3G07640,AT3G07680,AT5G49280,AT1G06460,AT5G49120,AT1G19920,AT3G05180,AT4G10300,AT1G16520,AT3G51430,AT1G07950,AT1G66940,AT5G12310,AT1G67830,AT2G28740,AT1G69160,AT2G29560,AT3G16470,AT3G50620,AT2G29150,AT2G29450,AT5G48100,AT4G16760,AT5G48250,AT1G22200,AT4G17740,AT2G41230,AT5G47730,AT1G29900,AT2G38610,AT5G46320,AT1G15820,AT5G45820,AT1G07040,AT1G07020,AT2G35190,AT1G20696,AT3G48460,AT1G17050,AT1G59930,AT5G45300,AT1G65800,AT1G21270,AT5G44410,AT1G27930,AT5G44020,AT2G14900,AT5G43930,AT2G29670,AT1G30200,AT1G55210,AT1G56300,AT2G18170,AT2G29650,AT1G25275,AT1G72060,AT1G54880,AT1G05540,AT5G04140,AT3G11200,AT3G11170,AT2G25950,AT1G66900,AT1G32590,AT5G40160,AT5G39410,AT2G03890,AT1G76510,AT4G39120,AT5G38480,AT1G68120,AT4G38570,AT2G22250,AT5G08330,AT2G41140,AT1G78210,AT5G36950,AT4G38050,AT5G36790,AT2G23600,AT2G31790,AT5G35840,AT4G37300,AT1G73180,AT5G35735,AT3G13750,AT4G36420,AT2G04690,AT1G04400,AT1G52890,AT5G23350,AT2G22990,AT4G34720,AT4G37260,AT1G74660,AT2G19450,AT5G23210,AT4G34950,AT1G26920,AT5G23050,AT3G26380,AT2G21385,AT3G51840,AT2G36900,AT1G77350,AT3G19100,AT1G58110,AT1G69830,AT5G17010,AT3G28860,AT2G36240,AT4G32530,AT2G30600,AT5G16290,AT2G32700,AT2G42160,AT2G22450,AT4G30740,AT5G14310,AT5G34883,AT5G14320,AT5G28020,AT2G43140,AT1G19540,AT4G28750,AT5G11740,AT4G28395,AT1G22710,AT5G11600,AT4G28460,AT4G28080,AT3G20390,AT5G67300,AT2G25080,AT5G07580,AT4G26050,AT5G66570,AT5G66590,AT3G25530,AT5G06730,AT1G01230,AT5G65630,AT4G24650,AT5G65530,AT1G17460,AT5G65380,AT3G19500,AT5G64260,AT3G21240,AT5G64330,AT1G70000,AT1G12780,AT1G12970,AT1G20020,AT2G17880,AT5G03350,AT1G48610,AT1G53090,AT1G03530,AT4G20360,AT3G21690,AT1G03600,AT3G17800,AT1G18740,AT3G15840,AT5G02150,AT4G19110,AT1G14380,AT4G19380,AT1G71720,AT3G16040,AT1G71820,AT3G63170,AT4G18280,AT5G60850,AT1G01700,AT1G49750,AT4G17840,AT5G59850,AT3G62660,AT1G50030,AT5G58910,AT1G76090,AT2G36800,AT5G58270,AT3G61580,AT2G43060,AT2G43010,AT3G08580,AT3G07840,AT1G80760,AT5G57060,AT1G80840,AT1G80730,AT3G11900,AT1G22540,AT5G56170,AT4G08980,AT1G22590,AT4G08390,AT4G07425,AT5G55380,AT1G52730,AT5G55070,AT1G74940,AT5G54950,AT1G78010,AT3G57170,AT5G54500,AT2G28200,AT4G03560,AT4G03600,AT1G27630,AT5G53290,AT3G56290,AT1G49270,AT1G49500,AT4G02540,AT3G07310,AT2G48020,AT3G09250,AT2G01760,AT4G01490,AT2G25710,AT5G51110,AT5G59920,AT4G01070

TGTAAAGT	10	2	['4-8']	236	AT5G51010,AT2G18790,AT3G52610,AT3G54240,AT3G52950,AT2G47610,AT3G53030,AT3G07700,AT2G37830,AT3G07640,AT3G07680,AT5G49280,AT1G06460,AT5G49120,AT1G19920,AT3G05180,AT4G10300,AT1G16520,AT3G51430,AT1G07950,AT1G66940,AT5G12310,AT1G67830,AT2G28740,AT1G69160,AT2G29560,AT3G16470,AT3G50620,AT2G29150,AT2G29450,AT5G48100,AT4G16760,AT5G48250,AT1G22200,AT4G17740,AT2G41230,AT5G47730,AT1G29900,AT2G38610,AT5G46320,AT1G15820,AT5G45820,AT1G07040,AT1G07020,AT2G35190,AT1G20696,AT3G48460,AT1G17050,AT1G59930,AT5G45300,AT1G65800,AT1G21270,AT5G44410,AT1G27930,AT5G44020,AT2G14900,AT5G43930,AT2G29670,AT1G30200,AT1G55210,AT1G56300,AT2G18170,AT2G29650,AT1G25275,AT1G72060,AT1G54880,AT1G05540,AT5G04140,AT3G11200,AT3G11170,AT2G25950,AT1G66900,AT1G32590,AT5G40160,AT5G39410,AT2G03890,AT1G76510,AT4G39120,AT5G38480,AT1G68120,AT4G38570,AT2G22250,AT5G08330,AT2G41140,AT1G78210,AT5G36950,AT4G38050,AT5G36790,AT2G23600,AT2G31790,AT5G35840,AT4G37300,AT1G73180,AT5G35735,AT3G13750,AT4G36420,AT2G04690,AT1G04400,AT1G52890,AT5G23350,AT2G22990,AT4G34720,AT4G37260,AT1G74660,AT2G19450,AT5G23210,AT4G34950,AT1G26920,AT5G23050,AT3G26380,AT2G21385,AT3G51840,AT2G36900,AT1G77350,AT3G19100,AT1G58110,AT1G69830,AT5G17010,AT3G28860,AT2G36240,AT4G32530,AT2G30600,AT5G16290,AT2G32700,AT2G42160,AT2G22450,AT4G30740,AT5G14310,AT5G34883,AT5G14320,AT5G28020,AT2G43140,AT1G19540,AT4G28750,AT5G11740,AT4G28395,AT1G22710,AT5G11600,AT4G28460,AT4G28080,AT3G20390,AT5G67300,AT2G25080,AT5G07580,AT4G26050,AT5G66570,AT5G66590,AT3G25530,AT5G06730,AT1G01230,AT5G65630,AT4G24650,AT5G65530,AT1G17460,AT5G65380,AT3G19500,AT5G64260,AT3G21240,AT5G64330,AT1G70000,AT1G12780,AT1G12970,AT1G20020,AT2G17880,AT5G03350,AT1G48610,AT1G53090,AT1G03530,AT4G20360,AT3G21690,AT1G03600,AT3G17800,AT1G18740,AT3G15840,AT5G02150,AT4G19110,AT1G14380,AT4G19380,AT1G71720,AT3G16040,AT1G71820,AT3G63170,AT4G18280,AT5G60850,AT1G01700,AT1G49750,AT4G17840,AT5G59850,AT3G62660,AT1G50030,AT5G58910,AT1G76090,AT2G36800,AT5G58270,AT3G61580,AT2G43060,AT2G43010,AT3G08580,AT3G07840,AT1G80760,AT5G57060,AT1G80840,AT1G80730,AT3G11900,AT1G22540,AT5G56170,AT4G08980,AT1G22590,AT4G08390,AT4G07425,AT5G55380,AT1G52730,AT5G55070,AT1G74940,AT5G54950,AT1G78010,AT3G57170,AT5G54500,AT2G28200,AT4G03560,AT4G03600,AT1G27630,AT5G53290,AT3G56290,AT1G49270,AT1G49500,AT4G02540,AT3G07310,AT2G48020,AT3G09250,AT2G01760,AT4G01490,AT2G25710,AT5G51110,AT5G59920,AT4G01070

TGTAAAGT	10	3	['4-8']	236	AT5G51010,AT2G18790,AT3G52610,AT3G54240,AT3G52950,AT2G47610,AT3G53030,AT3G07700,AT2G37830,AT3G07640,AT3G07680,AT5G49280,AT1G06460,AT5G49120,AT1G19920,AT3G05180,AT4G10300,AT1G16520,AT3G51430,AT1G07950,AT1G66940,AT5G12310,AT1G67830,AT2G28740,AT1G69160,AT2G29560,AT3G16470,AT3G50620,AT2G29150,AT2G29450,AT5G48100,AT4G16760,AT5G48250,AT1G22200,AT4G17740,AT2G41230,AT5G47730,AT1G29900,AT2G38610,AT5G46320,AT1G15820,AT5G45820,AT1G07040,AT1G07020,AT2G35190,AT1G20696,AT3G48460,AT1G17050,AT1G59930,AT5G45300,AT1G65800,AT1G21270,AT5G44410,AT1G27930,AT5G44020,AT2G14900,AT5G43930,AT2G29670,AT1G30200,AT1G55210,AT1G56300,AT2G18170,AT2G29650,AT1G25275,AT1G72060,AT1G54880,AT1G05540,AT5G04140,AT3G11200,AT3G11170,AT2G25950,AT1G66900,AT1G32590,AT5G40160,AT5G39410,AT2G03890,AT1G76510,AT4G39120,AT5G38480,AT1G68120,AT4G38570,AT2G22250,AT5G08330,AT2G41140,AT1G78210,AT5G36950,AT4G38050,AT5G36790,AT2G23600,AT2G31790,AT5G35840,AT4G37300,AT1G73180,AT5G35735,AT3G13750,AT4G36420,AT2G04690,AT1G04400,AT1G52890,AT5G23350,AT2G22990,AT4G34720,AT4G37260,AT1G74660,AT2G19450,AT5G23210,AT4G34950,AT1G26920,AT5G23050,AT3G26380,AT2G21385,AT3G51840,AT2G36900,AT1G77350,AT3G19100,AT1G58110,AT1G69830,AT5G17010,AT3G28860,AT2G36240,AT4G32530,AT2G30600,AT5G16290,AT2G32700,AT2G42160,AT2G22450,AT4G30740,AT5G14310,AT5G34883,AT5G14320,AT5G28020,AT2G43140,AT1G19540,AT4G28750,AT5G11740,AT4G28395,AT1G22710,AT5G11600,AT4G28460,AT4G28080,AT3G20390,AT5G67300,AT2G25080,AT5G07580,AT4G26050,AT5G66570,AT5G66590,AT3G25530,AT5G06730,AT1G01230,AT5G65630,AT4G24650,AT5G65530,AT1G17460,AT5G65380,AT3G19500,AT5G64260,AT3G21240,AT5G64330,AT1G70000,AT1G12780,AT1G12970,AT1G20020,AT2G17880,AT5G03350,AT1G48610,AT1G53090,AT1G03530,AT4G20360,AT3G21690,AT1G03600,AT3G17800,AT1G18740,AT3G15840,AT5G02150,AT4G19110,AT1G14380,AT4G19380,AT1G71720,AT3G16040,AT1G71820,AT3G63170,AT4G18280,AT5G60850,AT1G01700,AT1G49750,AT4G17840,AT5G59850,AT3G62660,AT1G50030,AT5G58910,AT1G76090,AT2G36800,AT5G58270,AT3G61580,AT2G43060,AT2G43010,AT3G08580,AT3G07840,AT1G80760,AT5G57060,AT1G80840,AT1G80730,AT3G11900,AT1G22540,AT5G56170,AT4G08980,AT1G22590,AT4G08390,AT4G07425,AT5G55380,AT1G52730,AT5G55070,AT1G74940,AT5G54950,AT1G78010,AT3G57170,AT5G54500,AT2G28200,AT4G03560,AT4G03600,AT1G27630,AT5G53290,AT3G56290,AT1G49270,AT1G49500,AT4G02540,AT3G07310,AT2G48020,AT3G09250,AT2G01760,AT4G01490,AT2G25710,AT5G51110,AT5G59920,AT4G01070

TGTAAAGT	10	4	['4-8']	236	AT5G51010,AT2G18790,AT3G52610,AT3G54240,AT3G52950,AT2G47610,AT3G53030,AT3G07700,AT2G37830,AT3G07640,AT3G07680,AT5G49280,AT1G06460,AT5G49120,AT1G19920,AT3G05180,AT4G10300,AT1G16520,AT3G51430,AT1G07950,AT1G66940,AT5G12310,AT1G67830,AT2G28740,AT1G69160,AT2G29560,AT3G16470,AT3G50620,AT2G29150,AT2G29450,AT5G48100,AT4G16760,AT5G48250,AT1G22200,AT4G17740,AT2G41230,AT5G47730,AT1G29900,AT2G38610,AT5G46320,AT1G15820,AT5G45820,AT1G07040,AT1G07020,AT2G35190,AT1G20696,AT3G48460,AT1G17050,AT1G59930,AT5G45300,AT1G65800,AT1G21270,AT5G44410,AT1G27930,AT5G44020,AT2G14900,AT5G43930,AT2G29670,AT1G30200,AT1G55210,AT1G56300,AT2G18170,AT2G29650,AT1G25275,AT1G72060,AT1G54880,AT1G05540,AT5G04140,AT3G11200,AT3G11170,AT2G25950,AT1G66900,AT1G32590,AT5G40160,AT5G39410,AT2G03890,AT1G76510,AT4G39120,AT5G38480,AT1G68120,AT4G38570,AT2G22250,AT5G08330,AT2G41140,AT1G78210,AT5G36950,AT4G38050,AT5G36790,AT2G23600,AT2G31790,AT5G35840,AT4G37300,AT1G73180,AT5G35735,AT3G13750,AT4G36420,AT2G04690,AT1G04400,AT1G52890,AT5G23350,AT2G22990,AT4G34720,AT4G37260,AT1G74660,AT2G19450,AT5G23210,AT4G34950,AT1G26920,AT5G23050,AT3G26380,AT2G21385,AT3G51840,AT2G36900,AT1G77350,AT3G19100,AT1G58110,AT1G69830,AT5G17010,AT3G28860,AT2G36240,AT4G32530,AT2G30600,AT5G16290,AT2G32700,AT2G42160,AT2G22450,AT4G30740,AT5G14310,AT5G34883,AT5G14320,AT5G28020,AT2G43140,AT1G19540,AT4G28750,AT5G11740,AT4G28395,AT1G22710,AT5G11600,AT4G28460,AT4G28080,AT3G20390,AT5G67300,AT2G25080,AT5G07580,AT4G26050,AT5G66570,AT5G66590,AT3G25530,AT5G06730,AT1G01230,AT5G65630,AT4G24650,AT5G65530,AT1G17460,AT5G65380,AT3G19500,AT5G64260,AT3G21240,AT5G64330,AT1G70000,AT1G12780,AT1G12970,AT1G20020,AT2G17880,AT5G03350,AT1G48610,AT1G53090,AT1G03530,AT4G20360,AT3G21690,AT1G03600,AT3G17800,AT1G18740,AT3G15840,AT5G02150,AT4G19110,AT1G14380,AT4G19380,AT1G71720,AT3G16040,AT1G71820,AT3G63170,AT4G18280,AT5G60850,AT1G01700,AT1G49750,AT4G17840,AT5G59850,AT3G62660,AT1G50030,AT5G58910,AT1G76090,AT2G36800,AT5G58270,AT3G61580,AT2G43060,AT2G43010,AT3G08580,AT3G07840,AT1G80760,AT5G57060,AT1G80840,AT1G80730,AT3G11900,AT1G22540,AT5G56170,AT4G08980,AT1G22590,AT4G08390,AT4G07425,AT5G55380,AT1G52730,AT5G55070,AT1G74940,AT5G54950,AT1G78010,AT3G57170,AT5G54500,AT2G28200,AT4G03560,AT4G03600,AT1G27630,AT5G53290,AT3G56290,AT1G49270,AT1G49500,AT4G02540,AT3G07310,AT2G48020,AT3G09250,AT2G01760,AT4G01490,AT2G25710,AT5G51110,AT5G59920,AT4G01070

TGTAAAGT	10	5	['4-8']	236	AT5G51010,AT2G18790,AT3G52610,AT3G54240,AT3G52950,AT2G47610,AT3G53030,AT3G07700,AT2G37830,AT3G07640,AT3G07680,AT5G49280,AT1G06460,AT5G49120,AT1G19920,AT3G05180,AT4G10300,AT1G16520,AT3G51430,AT1G07950,AT1G66940,AT5G12310,AT1G67830,AT2G28740,AT1G69160,AT2G29560,AT3G16470,AT3G50620,AT2G29150,AT2G29450,AT5G48100,AT4G16760,AT5G48250,AT1G22200,AT4G17740,AT2G41230,AT5G47730,AT1G29900,AT2G38610,AT5G46320,AT1G15820,AT5G45820,AT1G07040,AT1G07020,AT2G35190,AT1G20696,AT3G48460,AT1G17050,AT1G59930,AT5G45300,AT1G65800,AT1G21270,AT5G44410,AT1G27930,AT5G44020,AT2G14900,AT5G43930,AT2G29670,AT1G30200,AT1G55210,AT1G56300,AT2G18170,AT2G29650,AT1G25275,AT1G72060,AT1G54880,AT1G05540,AT5G04140,AT3G11200,AT3G11170,AT2G25950,AT1G66900,AT1G32590,AT5G40160,AT5G39410,AT2G03890,AT1G76510,AT4G39120,AT5G38480,AT1G68120,AT4G38570,AT2G22250,AT5G08330,AT2G41140,AT1G78210,AT5G36950,AT4G38050,AT5G36790,AT2G23600,AT2G31790,AT5G35840,AT4G37300,AT1G73180,AT5G35735,AT3G13750,AT4G36420,AT2G04690,AT1G04400,AT1G52890,AT5G23350,AT2G22990,AT4G34720,AT4G37260,AT1G74660,AT2G19450,AT5G23210,AT4G34950,AT1G26920,AT5G23050,AT3G26380,AT2G21385,AT3G51840,AT2G36900,AT1G77350,AT3G19100,AT1G58110,AT1G69830,AT5G17010,AT3G28860,AT2G36240,AT4G32530,AT2G30600,AT5G16290,AT2G32700,AT2G42160,AT2G22450,AT4G30740,AT5G14310,AT5G34883,AT5G14320,AT5G28020,AT2G43140,AT1G19540,AT4G28750,AT5G11740,AT4G28395,AT1G22710,AT5G11600,AT4G28460,AT4G28080,AT3G20390,AT5G67300,AT2G25080,AT5G07580,AT4G26050,AT5G66570,AT5G66590,AT3G25530,AT5G06730,AT1G01230,AT5G65630,AT4G24650,AT5G65530,AT1G17460,AT5G65380,AT3G19500,AT5G64260,AT3G21240,AT5G64330,AT1G70000,AT1G12780,AT1G12970,AT1G20020,AT2G17880,AT5G03350,AT1G48610,AT1G53090,AT1G03530,AT4G20360,AT3G21690,AT1G03600,AT3G17800,AT1G18740,AT3G15840,AT5G02150,AT4G19110,AT1G14380,AT4G19380,AT1G71720,AT3G16040,AT1G71820,AT3G63170,AT4G18280,AT5G60850,AT1G01700,AT1G49750,AT4G17840,AT5G59850,AT3G62660,AT1G50030,AT5G58910,AT1G76090,AT2G36800,AT5G58270,AT3G61580,AT2G43060,AT2G43010,AT3G08580,AT3G07840,AT1G80760,AT5G57060,AT1G80840,AT1G80730,AT3G11900,AT1G22540,AT5G56170,AT4G08980,AT1G22590,AT4G08390,AT4G07425,AT5G55380,AT1G52730,AT5G55070,AT1G74940,AT5G54950,AT1G78010,AT3G57170,AT5G54500,AT2G28200,AT4G03560,AT4G03600,AT1G27630,AT5G53290,AT3G56290,AT1G49270,AT1G49500,AT4G02540,AT3G07310,AT2G48020,AT3G09250,AT2G01760,AT4G01490,AT2G25710,AT5G51110,AT5G59920,AT4G01070

TGTATATAT	10	1	['4-8']	289	AT5G50840,AT4G00780,AT2G19480,ATCG00650,AT1G31330,AT1G34340,AT3G10200,AT3G10180,AT1G34300,AT2G26520,AT3G54030,AT2G05920,AT4G00960,AT4G00355,AT5G50012,AT3G52840,AT2G18700,AT4G00400,AT4G00150,AT2G47600,AT2G37830,AT2G38000,AT2G45420,AT3G52450,AT2G45200,AT5G48900,AT3G05180,AT1G76030,AT2G45170,AT1G16520,AT1G07950,AT3G51550,AT1G67740,AT2G40610,AT2G33420,AT1G69160,AT3G16470,AT3G50500,AT4G01070,AT3G50740,AT1G22330,AT5G47330,AT4G15550,AT5G47020,AT2G14690,AT1G64720,AT1G59860,AT3G48460,AT4G17460,AT3G47960,AT2G47690,AT1G79410,AT1G20540,AT2G35260,AT1G54520,AT3G47550,AT1G56430,AT1G54390,AT5G43670,AT1G23390,AT4G15730,AT3G47240,AT2G18170,AT3G47160,AT2G29660,AT3G12110,AT3G45260,AT1G25275,AT3G45690,AT1G28250,AT1G72060,AT2G46820,AT1G66230,AT5G41810,AT5G41050,AT5G41060,AT2G03110,AT1G10470,AT3G44720,AT1G66900,AT5G40840,AT2G31450,AT2G31360,AT3G43790,AT1G30270,AT2G44770,AT1G76360,AT5G40290,AT2G45990,AT4G39960,AT2G04240,AT1G75240,AT1G32590,AT2G39380,AT3G29770,AT5G19860,AT5G38550,AT3G19553,AT1G68120,AT1G68000,AT4G38830,AT2G41430,AT4G38050,AT5G35970,AT5G08380,AT5G20280,AT1G73180,AT2G01860,AT5G20840,AT1G52880,AT1G31814,AT1G60000,AT5G23080,AT2G21260,AT4G34710,AT3G24640,AT3G22530,AT3G56690,AT1G80500,AT1G63670,AT2G40400,AT3G56720,AT3G56840,AT2G19310,AT1G63800,AT2G36835,AT2G37040,AT5G17020,AT5G17990,AT5G17730,AT4G33300,AT2G35940,AT3G28860,AT1G72430,AT4G32410,AT4G32530,AT5G17230,AT3G23760,AT5G15230,AT4G32270,AT3G16520,AT5G15580,AT2G43840,AT5G15160,AT4G30610,AT3G24170,AT2G47350,AT1G53210,AT5G14180,AT1G79370,AT1G55960,AT4G30060,AT1G53280,AT4G29610,AT3G26590,AT5G27710,AT5G27920,AT1G24620,AT1G19540,AT5G11930,AT4G28740,AT5G26930,AT1G69040,AT1G09230,AT5G11150,AT4G27820,AT3G26290,AT5G25210,AT1G43715,AT5G25290,AT5G25220,AT5G09760,AT4G27670,AT3G12780,AT5G24890,AT5G24930,AT1G21460,AT5G24910,AT1G29270,AT5G67390,AT5G08120,AT3G26710,AT4G26130,AT1G67110,AT5G66950,AT3G17130,AT1G27440,AT1G26560,AT1G09390,AT1G01240,AT3G42570,AT1G01430,AT1G01250,AT4G25050,AT5G65730,AT4G25080,AT3G20770,AT1G62960,AT5G65380,AT5G05300,AT3G19880,AT3G19500,AT4G24340,AT3G19360,AT3G21250,AT1G32920,AT5G64330,AT4G23750,AT5G63880,AT1G62080,AT5G04040,AT3G14720,AT1G26780,AT2G17880,AT1G36980,AT5G63420,AT3G17900,AT1G79770,AT1G03550,AT3G13980,AT1G79700,AT3G13950,AT4G19830,AT5G02150,AT1G23205,AT1G23090,AT3G15790,AT1G14400,AT3G23325,AT5G61850,AT5G61410,AT1G71695,AT5G61580,AT4G18270,AT1G67280,AT3G16690,AT5G60540,AT3G62410,AT3G02660,AT1G03900,AT1G18330,AT3G07010,AT1G47290,AT3G04360,AT4G12390,AT4G12320,AT1G15520,AT4G12060,AT1G76240,AT1G25155,AT1G10650,AT3G07840,AT2G14080,AT4G10040,AT2G18390,AT3G59660,AT3G05800,AT3G59480,AT3G10912,AT5G56240,AT4G08840,AT2G46550,AT5G55530,AT3G58120,AT3G58620,AT2G15890,AT2G15960,AT1G74940,AT3G57770,AT1G78010,AT3G57020,AT4G04630,AT3G05720,AT3G57040,AT3G06350,AT2G25510,AT2G27490,AT1G70790,AT3G10460,AT1G73120,AT5G53370,AT2G32100,AT3G55670,AT3G07550,AT4G01940,AT3G55010,AT4G01500,AT1G34190,AT4G00270

TGTATATAT	10	2	['4-8']	289	AT5G50840,AT4G00780,AT2G19480,ATCG00650,AT1G31330,AT1G34340,AT3G10200,AT3G10180,AT1G34300,AT2G26520,AT3G54030,AT2G05920,AT4G00960,AT4G00355,AT5G50012,AT3G52840,AT2G18700,AT4G00400,AT4G00150,AT2G47600,AT2G37830,AT2G38000,AT2G45420,AT3G52450,AT2G45200,AT5G48900,AT3G05180,AT1G76030,AT2G45170,AT1G16520,AT1G07950,AT3G51550,AT1G67740,AT2G40610,AT2G33420,AT1G69160,AT3G16470,AT3G50500,AT4G01070,AT3G50740,AT1G22330,AT5G47330,AT4G15550,AT5G47020,AT2G14690,AT1G64720,AT1G59860,AT3G48460,AT4G17460,AT3G47960,AT2G47690,AT1G79410,AT1G20540,AT2G35260,AT1G54520,AT3G47550,AT1G56430,AT1G54390,AT5G43670,AT1G23390,AT4G15730,AT3G47240,AT2G18170,AT3G47160,AT2G29660,AT3G12110,AT3G45260,AT1G25275,AT3G45690,AT1G28250,AT1G72060,AT2G46820,AT1G66230,AT5G41810,AT5G41050,AT5G41060,AT2G03110,AT1G10470,AT3G44720,AT1G66900,AT5G40840,AT2G31450,AT2G31360,AT3G43790,AT1G30270,AT2G44770,AT1G76360,AT5G40290,AT2G45990,AT4G39960,AT2G04240,AT1G75240,AT1G32590,AT2G39380,AT3G29770,AT5G19860,AT5G38550,AT3G19553,AT1G68120,AT1G68000,AT4G38830,AT2G41430,AT4G38050,AT5G35970,AT5G08380,AT5G20280,AT1G73180,AT2G01860,AT5G20840,AT1G52880,AT1G31814,AT1G60000,AT5G23080,AT2G21260,AT4G34710,AT3G24640,AT3G22530,AT3G56690,AT1G80500,AT1G63670,AT2G40400,AT3G56720,AT3G56840,AT2G19310,AT1G63800,AT2G36835,AT2G37040,AT5G17020,AT5G17990,AT5G17730,AT4G33300,AT2G35940,AT3G28860,AT1G72430,AT4G32410,AT4G32530,AT5G17230,AT3G23760,AT5G15230,AT4G32270,AT3G16520,AT5G15580,AT2G43840,AT5G15160,AT4G30610,AT3G24170,AT2G47350,AT1G53210,AT5G14180,AT1G79370,AT1G55960,AT4G30060,AT1G53280,AT4G29610,AT3G26590,AT5G27710,AT5G27920,AT1G24620,AT1G19540,AT5G11930,AT4G28740,AT5G26930,AT1G69040,AT1G09230,AT5G11150,AT4G27820,AT3G26290,AT5G25210,AT1G43715,AT5G25290,AT5G25220,AT5G09760,AT4G27670,AT3G12780,AT5G24890,AT5G24930,AT1G21460,AT5G24910,AT1G29270,AT5G67390,AT5G08120,AT3G26710,AT4G26130,AT1G67110,AT5G66950,AT3G17130,AT1G27440,AT1G26560,AT1G09390,AT1G01240,AT3G42570,AT1G01430,AT1G01250,AT4G25050,AT5G65730,AT4G25080,AT3G20770,AT1G62960,AT5G65380,AT5G05300,AT3G19880,AT3G19500,AT4G24340,AT3G19360,AT3G21250,AT1G32920,AT5G64330,AT4G23750,AT5G63880,AT1G62080,AT5G04040,AT3G14720,AT1G26780,AT2G17880,AT1G36980,AT5G63420,AT3G17900,AT1G79770,AT1G03550,AT3G13980,AT1G79700,AT3G13950,AT4G19830,AT5G02150,AT1G23205,AT1G23090,AT3G15790,AT1G14400,AT3G23325,AT5G61850,AT5G61410,AT1G71695,AT5G61580,AT4G18270,AT1G67280,AT3G16690,AT5G60540,AT3G62410,AT3G02660,AT1G03900,AT1G18330,AT3G07010,AT1G47290,AT3G04360,AT4G12390,AT4G12320,AT1G15520,AT4G12060,AT1G76240,AT1G25155,AT1G10650,AT3G07840,AT2G14080,AT4G10040,AT2G18390,AT3G59660,AT3G05800,AT3G59480,AT3G10912,AT5G56240,AT4G08840,AT2G46550,AT5G55530,AT3G58120,AT3G58620,AT2G15890,AT2G15960,AT1G74940,AT3G57770,AT1G78010,AT3G57020,AT4G04630,AT3G05720,AT3G57040,AT3G06350,AT2G25510,AT2G27490,AT1G70790,AT3G10460,AT1G73120,AT5G53370,AT2G32100,AT3G55670,AT3G07550,AT4G01940,AT3G55010,AT4G01500,AT1G34190,AT4G00270

TGTATATAT	10	3	['4-8']	289	AT5G50840,AT4G00780,AT2G19480,ATCG00650,AT1G31330,AT1G34340,AT3G10200,AT3G10180,AT1G34300,AT2G26520,AT3G54030,AT2G05920,AT4G00960,AT4G00355,AT5G50012,AT3G52840,AT2G18700,AT4G00400,AT4G00150,AT2G47600,AT2G37830,AT2G38000,AT2G45420,AT3G52450,AT2G45200,AT5G48900,AT3G05180,AT1G76030,AT2G45170,AT1G16520,AT1G07950,AT3G51550,AT1G67740,AT2G40610,AT2G33420,AT1G69160,AT3G16470,AT3G50500,AT4G01070,AT3G50740,AT1G22330,AT5G47330,AT4G15550,AT5G47020,AT2G14690,AT1G64720,AT1G59860,AT3G48460,AT4G17460,AT3G47960,AT2G47690,AT1G79410,AT1G20540,AT2G35260,AT1G54520,AT3G47550,AT1G56430,AT1G54390,AT5G43670,AT1G23390,AT4G15730,AT3G47240,AT2G18170,AT3G47160,AT2G29660,AT3G12110,AT3G45260,AT1G25275,AT3G45690,AT1G28250,AT1G72060,AT2G46820,AT1G66230,AT5G41810,AT5G41050,AT5G41060,AT2G03110,AT1G10470,AT3G44720,AT1G66900,AT5G40840,AT2G31450,AT2G31360,AT3G43790,AT1G30270,AT2G44770,AT1G76360,AT5G40290,AT2G45990,AT4G39960,AT2G04240,AT1G75240,AT1G32590,AT2G39380,AT3G29770,AT5G19860,AT5G38550,AT3G19553,AT1G68120,AT1G68000,AT4G38830,AT2G41430,AT4G38050,AT5G35970,AT5G08380,AT5G20280,AT1G73180,AT2G01860,AT5G20840,AT1G52880,AT1G31814,AT1G60000,AT5G23080,AT2G21260,AT4G34710,AT3G24640,AT3G22530,AT3G56690,AT1G80500,AT1G63670,AT2G40400,AT3G56720,AT3G56840,AT2G19310,AT1G63800,AT2G36835,AT2G37040,AT5G17020,AT5G17990,AT5G17730,AT4G33300,AT2G35940,AT3G28860,AT1G72430,AT4G32410,AT4G32530,AT5G17230,AT3G23760,AT5G15230,AT4G32270,AT3G16520,AT5G15580,AT2G43840,AT5G15160,AT4G30610,AT3G24170,AT2G47350,AT1G53210,AT5G14180,AT1G79370,AT1G55960,AT4G30060,AT1G53280,AT4G29610,AT3G26590,AT5G27710,AT5G27920,AT1G24620,AT1G19540,AT5G11930,AT4G28740,AT5G26930,AT1G69040,AT1G09230,AT5G11150,AT4G27820,AT3G26290,AT5G25210,AT1G43715,AT5G25290,AT5G25220,AT5G09760,AT4G27670,AT3G12780,AT5G24890,AT5G24930,AT1G21460,AT5G24910,AT1G29270,AT5G67390,AT5G08120,AT3G26710,AT4G26130,AT1G67110,AT5G66950,AT3G17130,AT1G27440,AT1G26560,AT1G09390,AT1G01240,AT3G42570,AT1G01430,AT1G01250,AT4G25050,AT5G65730,AT4G25080,AT3G20770,AT1G62960,AT5G65380,AT5G05300,AT3G19880,AT3G19500,AT4G24340,AT3G19360,AT3G21250,AT1G32920,AT5G64330,AT4G23750,AT5G63880,AT1G62080,AT5G04040,AT3G14720,AT1G26780,AT2G17880,AT1G36980,AT5G63420,AT3G17900,AT1G79770,AT1G03550,AT3G13980,AT1G79700,AT3G13950,AT4G19830,AT5G02150,AT1G23205,AT1G23090,AT3G15790,AT1G14400,AT3G23325,AT5G61850,AT5G61410,AT1G71695,AT5G61580,AT4G18270,AT1G67280,AT3G16690,AT5G60540,AT3G62410,AT3G02660,AT1G03900,AT1G18330,AT3G07010,AT1G47290,AT3G04360,AT4G12390,AT4G12320,AT1G15520,AT4G12060,AT1G76240,AT1G25155,AT1G10650,AT3G07840,AT2G14080,AT4G10040,AT2G18390,AT3G59660,AT3G05800,AT3G59480,AT3G10912,AT5G56240,AT4G08840,AT2G46550,AT5G55530,AT3G58120,AT3G58620,AT2G15890,AT2G15960,AT1G74940,AT3G57770,AT1G78010,AT3G57020,AT4G04630,AT3G05720,AT3G57040,AT3G06350,AT2G25510,AT2G27490,AT1G70790,AT3G10460,AT1G73120,AT5G53370,AT2G32100,AT3G55670,AT3G07550,AT4G01940,AT3G55010,AT4G01500,AT1G34190,AT4G00270

TGTATATAT	10	4	['4-8']	289	AT5G50840,AT4G00780,AT2G19480,ATCG00650,AT1G31330,AT1G34340,AT3G10200,AT3G10180,AT1G34300,AT2G26520,AT3G54030,AT2G05920,AT4G00960,AT4G00355,AT5G50012,AT3G52840,AT2G18700,AT4G00400,AT4G00150,AT2G47600,AT2G37830,AT2G38000,AT2G45420,AT3G52450,AT2G45200,AT5G48900,AT3G05180,AT1G76030,AT2G45170,AT1G16520,AT1G07950,AT3G51550,AT1G67740,AT2G40610,AT2G33420,AT1G69160,AT3G16470,AT3G50500,AT4G01070,AT3G50740,AT1G22330,AT5G47330,AT4G15550,AT5G47020,AT2G14690,AT1G64720,AT1G59860,AT3G48460,AT4G17460,AT3G47960,AT2G47690,AT1G79410,AT1G20540,AT2G35260,AT1G54520,AT3G47550,AT1G56430,AT1G54390,AT5G43670,AT1G23390,AT4G15730,AT3G47240,AT2G18170,AT3G47160,AT2G29660,AT3G12110,AT3G45260,AT1G25275,AT3G45690,AT1G28250,AT1G72060,AT2G46820,AT1G66230,AT5G41810,AT5G41050,AT5G41060,AT2G03110,AT1G10470,AT3G44720,AT1G66900,AT5G40840,AT2G31450,AT2G31360,AT3G43790,AT1G30270,AT2G44770,AT1G76360,AT5G40290,AT2G45990,AT4G39960,AT2G04240,AT1G75240,AT1G32590,AT2G39380,AT3G29770,AT5G19860,AT5G38550,AT3G19553,AT1G68120,AT1G68000,AT4G38830,AT2G41430,AT4G38050,AT5G35970,AT5G08380,AT5G20280,AT1G73180,AT2G01860,AT5G20840,AT1G52880,AT1G31814,AT1G60000,AT5G23080,AT2G21260,AT4G34710,AT3G24640,AT3G22530,AT3G56690,AT1G80500,AT1G63670,AT2G40400,AT3G56720,AT3G56840,AT2G19310,AT1G63800,AT2G36835,AT2G37040,AT5G17020,AT5G17990,AT5G17730,AT4G33300,AT2G35940,AT3G28860,AT1G72430,AT4G32410,AT4G32530,AT5G17230,AT3G23760,AT5G15230,AT4G32270,AT3G16520,AT5G15580,AT2G43840,AT5G15160,AT4G30610,AT3G24170,AT2G47350,AT1G53210,AT5G14180,AT1G79370,AT1G55960,AT4G30060,AT1G53280,AT4G29610,AT3G26590,AT5G27710,AT5G27920,AT1G24620,AT1G19540,AT5G11930,AT4G28740,AT5G26930,AT1G69040,AT1G09230,AT5G11150,AT4G27820,AT3G26290,AT5G25210,AT1G43715,AT5G25290,AT5G25220,AT5G09760,AT4G27670,AT3G12780,AT5G24890,AT5G24930,AT1G21460,AT5G24910,AT1G29270,AT5G67390,AT5G08120,AT3G26710,AT4G26130,AT1G67110,AT5G66950,AT3G17130,AT1G27440,AT1G26560,AT1G09390,AT1G01240,AT3G42570,AT1G01430,AT1G01250,AT4G25050,AT5G65730,AT4G25080,AT3G20770,AT1G62960,AT5G65380,AT5G05300,AT3G19880,AT3G19500,AT4G24340,AT3G19360,AT3G21250,AT1G32920,AT5G64330,AT4G23750,AT5G63880,AT1G62080,AT5G04040,AT3G14720,AT1G26780,AT2G17880,AT1G36980,AT5G63420,AT3G17900,AT1G79770,AT1G03550,AT3G13980,AT1G79700,AT3G13950,AT4G19830,AT5G02150,AT1G23205,AT1G23090,AT3G15790,AT1G14400,AT3G23325,AT5G61850,AT5G61410,AT1G71695,AT5G61580,AT4G18270,AT1G67280,AT3G16690,AT5G60540,AT3G62410,AT3G02660,AT1G03900,AT1G18330,AT3G07010,AT1G47290,AT3G04360,AT4G12390,AT4G12320,AT1G15520,AT4G12060,AT1G76240,AT1G25155,AT1G10650,AT3G07840,AT2G14080,AT4G10040,AT2G18390,AT3G59660,AT3G05800,AT3G59480,AT3G10912,AT5G56240,AT4G08840,AT2G46550,AT5G55530,AT3G58120,AT3G58620,AT2G15890,AT2G15960,AT1G74940,AT3G57770,AT1G78010,AT3G57020,AT4G04630,AT3G05720,AT3G57040,AT3G06350,AT2G25510,AT2G27490,AT1G70790,AT3G10460,AT1G73120,AT5G53370,AT2G32100,AT3G55670,AT3G07550,AT4G01940,AT3G55010,AT4G01500,AT1G34190,AT4G00270

TGTATATAT	10	5	['4-8']	289	AT5G50840,AT4G00780,AT2G19480,ATCG00650,AT1G31330,AT1G34340,AT3G10200,AT3G10180,AT1G34300,AT2G26520,AT3G54030,AT2G05920,AT4G00960,AT4G00355,AT5G50012,AT3G52840,AT2G18700,AT4G00400,AT4G00150,AT2G47600,AT2G37830,AT2G38000,AT2G45420,AT3G52450,AT2G45200,AT5G48900,AT3G05180,AT1G76030,AT2G45170,AT1G16520,AT1G07950,AT3G51550,AT1G67740,AT2G40610,AT2G33420,AT1G69160,AT3G16470,AT3G50500,AT4G01070,AT3G50740,AT1G22330,AT5G47330,AT4G15550,AT5G47020,AT2G14690,AT1G64720,AT1G59860,AT3G48460,AT4G17460,AT3G47960,AT2G47690,AT1G79410,AT1G20540,AT2G35260,AT1G54520,AT3G47550,AT1G56430,AT1G54390,AT5G43670,AT1G23390,AT4G15730,AT3G47240,AT2G18170,AT3G47160,AT2G29660,AT3G12110,AT3G45260,AT1G25275,AT3G45690,AT1G28250,AT1G72060,AT2G46820,AT1G66230,AT5G41810,AT5G41050,AT5G41060,AT2G03110,AT1G10470,AT3G44720,AT1G66900,AT5G40840,AT2G31450,AT2G31360,AT3G43790,AT1G30270,AT2G44770,AT1G76360,AT5G40290,AT2G45990,AT4G39960,AT2G04240,AT1G75240,AT1G32590,AT2G39380,AT3G29770,AT5G19860,AT5G38550,AT3G19553,AT1G68120,AT1G68000,AT4G38830,AT2G41430,AT4G38050,AT5G35970,AT5G08380,AT5G20280,AT1G73180,AT2G01860,AT5G20840,AT1G52880,AT1G31814,AT1G60000,AT5G23080,AT2G21260,AT4G34710,AT3G24640,AT3G22530,AT3G56690,AT1G80500,AT1G63670,AT2G40400,AT3G56720,AT3G56840,AT2G19310,AT1G63800,AT2G36835,AT2G37040,AT5G17020,AT5G17990,AT5G17730,AT4G33300,AT2G35940,AT3G28860,AT1G72430,AT4G32410,AT4G32530,AT5G17230,AT3G23760,AT5G15230,AT4G32270,AT3G16520,AT5G15580,AT2G43840,AT5G15160,AT4G30610,AT3G24170,AT2G47350,AT1G53210,AT5G14180,AT1G79370,AT1G55960,AT4G30060,AT1G53280,AT4G29610,AT3G26590,AT5G27710,AT5G27920,AT1G24620,AT1G19540,AT5G11930,AT4G28740,AT5G26930,AT1G69040,AT1G09230,AT5G11150,AT4G27820,AT3G26290,AT5G25210,AT1G43715,AT5G25290,AT5G25220,AT5G09760,AT4G27670,AT3G12780,AT5G24890,AT5G24930,AT1G21460,AT5G24910,AT1G29270,AT5G67390,AT5G08120,AT3G26710,AT4G26130,AT1G67110,AT5G66950,AT3G17130,AT1G27440,AT1G26560,AT1G09390,AT1G01240,AT3G42570,AT1G01430,AT1G01250,AT4G25050,AT5G65730,AT4G25080,AT3G20770,AT1G62960,AT5G65380,AT5G05300,AT3G19880,AT3G19500,AT4G24340,AT3G19360,AT3G21250,AT1G32920,AT5G64330,AT4G23750,AT5G63880,AT1G62080,AT5G04040,AT3G14720,AT1G26780,AT2G17880,AT1G36980,AT5G63420,AT3G17900,AT1G79770,AT1G03550,AT3G13980,AT1G79700,AT3G13950,AT4G19830,AT5G02150,AT1G23205,AT1G23090,AT3G15790,AT1G14400,AT3G23325,AT5G61850,AT5G61410,AT1G71695,AT5G61580,AT4G18270,AT1G67280,AT3G16690,AT5G60540,AT3G62410,AT3G02660,AT1G03900,AT1G18330,AT3G07010,AT1G47290,AT3G04360,AT4G12390,AT4G12320,AT1G15520,AT4G12060,AT1G76240,AT1G25155,AT1G10650,AT3G07840,AT2G14080,AT4G10040,AT2G18390,AT3G59660,AT3G05800,AT3G59480,AT3G10912,AT5G56240,AT4G08840,AT2G46550,AT5G55530,AT3G58120,AT3G58620,AT2G15890,AT2G15960,AT1G74940,AT3G57770,AT1G78010,AT3G57020,AT4G04630,AT3G05720,AT3G57040,AT3G06350,AT2G25510,AT2G27490,AT1G70790,AT3G10460,AT1G73120,AT5G53370,AT2G32100,AT3G55670,AT3G07550,AT4G01940,AT3G55010,AT4G01500,AT1G34190,AT4G00270
TTATACTAGT	10	1	['4-8', '16-20']	23	AT1G58380,AT1G55490,AT5G59140,AT2G41430,AT3G60600,AT1G77270,AT1G80500,AT4G34220,AT3G19360,AT1G48650,AT1G12860,AT3G47560,AT3G56660,AT5G53770,AT3G01990,AT3G21690,AT3G01790,AT2G43840,AT1G53210,AT3G15690,AT2G16530,AT4G29700,AT1G74170
TTATACTAGT	10	2	['4-8', '16-20']	23	AT1G58380,AT1G55490,AT5G59140,AT2G41430,AT3G60600,AT1G77270,AT1G80500,AT4G34220,AT3G19360,AT1G48650,AT1G12860,AT3G47560,AT3G56660,AT5G53770,AT3G01990,AT3G21690,AT3G01790,AT2G43840,AT1G53210,AT3G15690,AT2G16530,AT4G29700,AT1G74170
TTATACTAGT	10	3	['4-8', '16-20']	23	AT1G58380,AT1G55490,AT5G59140,AT2G41430,AT3G60600,AT1G77270,AT1G80500,AT4G34220,AT3G19360,AT1G48650,AT1G12860,AT3G47560,AT3G56660,AT5G53770,AT3G01990,AT3G21690,AT3G01790,AT2G43840,AT1G53210,AT3G15690,AT2G16530,AT4G29700,AT1G74170
TTATACTAGT	10	4	['4-8', '16-20']	23	AT1G58380,AT1G55490,AT5G59140,AT2G41430,AT3G60600,AT1G77270,AT1G80500,AT4G34220,AT3G19360,AT1G48650,AT1G12860,AT3G47560,AT3G56660,AT5G53770,AT3G01990,AT3G21690,AT3G01790,AT2G43840,AT1G53210,AT3G15690,AT2G16530,AT4G29700,AT1G74170
TTATACTAGT	10	5	['4-8', '16-20']	23	AT1G58380,AT1G55490,AT5G59140,AT2G41430,AT3G60600,AT1G77270,AT1G80500,AT4G34220,AT3G19360,AT1G48650,AT1G12860,AT3G47560,AT3G56660,AT5G53770,AT3G01990,AT3G21690,AT3G01790,AT2G43840,AT1G53210,AT3G15690,AT2G16530,AT4G29700,AT1G74170

TTCCCTGTT	10	1	['8-12', '12-16']	31	AT3G15430,AT1G31280,AT1G16150,AT5G50780,AT4G13830,AT5G26760,AT1G18310,AT4G13250,AT5G36960,AT1G73650,AT1G10200,AT2G30990,AT3G13790,AT3G59910,AT1G07140,AT5G65660,AT4G08690,AT5G05520,AT1G65490,AT2G19230,AT4G33670,AT3G16200,AT2G25740,AT3G11250,AT5G15770,AT1G71180,AT5G35380,AT2G47390,AT3G44250,AT5G01890,AT5G12210
TTCCCTGTT	10	2	['8-12', '12-16']	31	AT3G15430,AT1G31280,AT1G16150,AT5G50780,AT4G13830,AT5G26760,AT1G18310,AT4G13250,AT5G36960,AT1G73650,AT1G10200,AT2G30990,AT3G13790,AT3G59910,AT1G07140,AT5G65660,AT4G08690,AT5G05520,AT1G65490,AT2G19230,AT4G33670,AT3G16200,AT2G25740,AT3G11250,AT5G15770,AT1G71180,AT5G35380,AT2G47390,AT3G44250,AT5G01890,AT5G12210
TTCCCTGTT	10	3	['8-12', '12-16']	31	AT3G15430,AT1G31280,AT1G16150,AT5G50780,AT4G13830,AT5G26760,AT1G18310,AT4G13250,AT5G36960,AT1G73650,AT1G10200,AT2G30990,AT3G13790,AT3G59910,AT1G07140,AT5G65660,AT4G08690,AT5G05520,AT1G65490,AT2G19230,AT4G33670,AT3G16200,AT2G25740,AT3G11250,AT5G15770,AT1G71180,AT5G35380,AT2G47390,AT3G44250,AT5G01890,AT5G12210
TTCCCTGTT	10	4	['8-12', '12-16']	31	AT3G15430,AT1G31280,AT1G16150,AT5G50780,AT4G13830,AT5G26760,AT1G18310,AT4G13250,AT5G36960,AT1G73650,AT1G10200,AT2G30990,AT3G13790,AT3G59910,AT1G07140,AT5G65660,AT4G08690,AT5G05520,AT1G65490,AT2G19230,AT4G33670,AT3G16200,AT2G25740,AT3G11250,AT5G15770,AT1G71180,AT5G35380,AT2G47390,AT3G44250,AT5G01890,AT5G12210
TTCCCTGTT	10	5	['8-12', '12-16']	31	AT3G15430,AT1G31280,AT1G16150,AT5G50780,AT4G13830,AT5G26760,AT1G18310,AT4G13250,AT5G36960,AT1G73650,AT1G10200,AT2G30990,AT3G13790,AT3G59910,AT1G07140,AT5G65660,AT4G08690,AT5G05520,AT1G65490,AT2G19230,AT4G33670,AT3G16200,AT2G25740,AT3G11250,AT5G15770,AT1G71180,AT5G35380,AT2G47390,AT3G44250,AT5G01890,AT5G12210
TTCGTACAA	10	1	['0-4', '16-20']	39	AT1G17100,AT1G67360,ATCG00470,AT1G24450,AT5G50012,AT1G06570,AT3G61430,AT4G38160,AT2G42600,AT1G07950,AT1G67830,AT4G11100,AT1G10200,AT1G11545,AT4G26070,AT5G57290,AT3G19810,AT1G04620,AT5G23060,AT1G74430,AT4G09020,AT5G19010,AT3G14080,AT1G79470,AT3G47550,AT2G44500,AT5G17440,AT4G23060,AT2G35370,AT1G69730,AT2G35880,AT2G17840,AT5G63380,AT1G79730,AT1G78570,AT2G43790,AT4G01690,AT1G55840,AT2G25680
TTCGTACAA	10	2	['0-4', '16-20']	39	AT1G17100,AT1G67360,ATCG00470,AT1G24450,AT5G50012,AT1G06570,AT3G61430,AT4G38160,AT2G42600,AT1G07950,AT1G67830,AT4G11100,AT1G10200,AT1G11545,AT4G26070,AT5G57290,AT3G19810,AT1G04620,AT5G23060,AT1G74430,AT4G09020,AT5G19010,AT3G14080,AT1G79470,AT3G47550,AT2G44500,AT5G17440,AT4G23060,AT2G35370,AT1G69730,AT2G35880,AT2G17840,AT5G63380,AT1G79730,AT1G78570,AT2G43790,AT4G01690,AT1G55840,AT2G25680
TTCGTACAA	10	3	['0-4', '16-20']	39	AT1G17100,AT1G67360,ATCG00470,AT1G24450,AT5G50012,AT1G06570,AT3G61430,AT4G38160,AT2G42600,AT1G07950,AT1G67830,AT4G11100,AT1G10200,AT1G11545,AT4G26070,AT5G57290,AT3G19810,AT1G04620,AT5G23060,AT1G74430,AT4G09020,AT5G19010,AT3G14080,AT1G79470,AT3G47550,AT2G44500,AT5G17440,AT4G23060,AT2G35370,AT1G69730,AT2G35880,AT2G17840,AT5G63380,AT1G79730,AT1G78570,AT2G43790,AT4G01690,AT1G55840,AT2G25680
TTCGTACAA	10	4	['0-4', '16-20']	39	AT1G17100,AT1G67360,ATCG00470,AT1G24450,AT5G50012,AT1G06570,AT3G61430,AT4G38160,AT2G42600,AT1G07950,AT1G67830,AT4G11100,AT1G10200,AT1G11545,AT4G26070,AT5G57290,AT3G19810,AT1G04620,AT5G23060,AT1G74430,AT4G09020,AT5G19010,AT3G14080,AT1G79470,AT3G47550,AT2G44500,AT5G17440,AT4G23060,AT2G35370,AT1G69730,AT2G35880,AT2G17840,AT5G63380,AT1G79730,AT1G78570,AT2G43790,AT4G01690,AT1G55840,AT2G25680
TTCGTACAA	10	5	['16-20', '0-4']	39	AT1G17100,AT1G67360,ATCG00470,AT1G24450,AT5G50012,AT1G06570,AT3G61430,AT4G38160,AT2G42600,AT1G07950,AT1G67830,AT4G11100,AT1G10200,AT1G11545,AT4G26070,AT5G57290,AT3G19810,AT1G04620,AT5G23060,AT1G74430,AT4G09020,AT5G19010,AT3G14080,AT1G79470,AT3G47550,AT2G44500,AT5G17440,AT4G23060,AT2G35370,AT1G69730,AT2G35880,AT2G17840,AT5G63380,AT1G79730,AT1G78570,AT2G43790,AT4G01690,AT1G55840,AT2G25680
TTTTACTAGT	10	1	['0-4', '4-8', '8-12', '20-24']	26	AT4G29260,AT4G28460,AT2G34250,AT1G78690,AT5G25430,AT5G58730,AT1G21460,AT2G34090,AT4G37640,AT5G67480,AT3G20970,AT2G18240,AT3G59940,AT5G23060,AT5G56240,AT2G39930,AT3G28580,AT4G04840,AT3G46970,AT1G08720,AT4G02540,AT4G30690,AT4G30900,AT5G02240,AT1G30220,AT2G26080
TTTTACTAGT	10	2	['0-4', '4-8', '8-12', '20-24']	26	AT4G29260,AT4G28460,AT2G34250,AT1G78690,AT5G25430,AT5G58730,AT1G21460,AT2G34090,AT4G37640,AT5G67480,AT3G20970,AT2G18240,AT3G59940,AT5G23060,AT5G56240,AT2G39930,AT3G28580,AT4G04840,AT3G46970,AT1G08720,AT4G02540,AT4G30690,AT4G30900,AT5G02240,AT1G30220,AT2G26080
TTTTACTAGT	10	3	['0-4', '4-8', '8-12', '20-24']	26	AT4G29260,AT4G28460,AT2G34250,AT1G78690,AT5G25430,AT5G58730,AT1G21460,AT2G34090,AT4G37640,AT5G67480,AT3G20970,AT2G18240,AT3G59940,AT5G23060,AT5G56240,AT2G39930,AT3G28580,AT4G04840,AT3G46970,AT1G08720,AT4G02540,AT4G30690,AT4G30900,AT5G02240,AT1G30220,AT2G26080

TTTTACTAGT	10	4	['0-4', '4-8', '8-12', '20-24']	26	AT4G29260,AT4G28460,AT2G34250,AT1G78690,AT5G25430,AT5G58730,AT1G21460,AT2G34090,AT4G37640,AT5G67480,AT3G20970,AT2G18240,AT3G59940,AT5G23060,AT5G56240,AT2G39930,AT3G28580,AT4G04840,AT3G46970,AT1G08720,AT4G02540,AT4G30690,AT4G30900,AT5G02240,AT1G30220,AT2G26080
TTTTACTAGT	10	5	['4-8', '8-12', '20-24', '0-4']	26	AT4G29260,AT4G28460,AT2G34250,AT1G78690,AT5G25430,AT5G58730,AT1G21460,AT2G34090,AT4G37640,AT5G67480,AT3G20970,AT2G18240,AT3G59940,AT5G23060,AT5G56240,AT2G39930,AT3G28580,AT4G04840,AT3G46970,AT1G08720,AT4G02540,AT4G30690,AT4G30900,AT5G02240,AT1G30220,AT2G26080
TTWCCWWWWNNGGWW	10	1	['4-8', '12-16']	34	AT5G27150,AT1G75080,AT1G33270,AT5G19860,AT5G37640,AT1G31050,AT5G25070,AT4G35790,AT1G09390,AT5G23760,AT4G14960,AT5G65910,AT1G14920,AT3G13510,AT4G14965,AT1G77920,AT4G05150,AT2G15670,AT5G44010,AT1G74920,AT4G04930,AT4G16140,AT1G23010,AT2G02710,AT1G72430,AT4G03600,AT5G03140,AT3G21690,AT2G17340,AT4G02480,AT3G44660,AT2G32950,AT5G51570,AT1G30210
TTWCCWWWWNNGGWW	10	2	['4-8', '12-16']	34	AT5G27150,AT1G75080,AT1G33270,AT5G19860,AT5G37640,AT1G31050,AT5G25070,AT4G35790,AT1G09390,AT5G23760,AT4G14960,AT5G65910,AT1G14920,AT3G13510,AT4G14965,AT1G77920,AT4G05150,AT2G15670,AT5G44010,AT1G74920,AT4G04930,AT4G16140,AT1G23010,AT2G02710,AT1G72430,AT4G03600,AT5G03140,AT3G21690,AT2G17340,AT4G02480,AT3G44660,AT2G32950,AT5G51570,AT1G30210
TTWCCWWWWNNGGWW	10	3	['4-8', '12-16']	34	AT5G27150,AT1G75080,AT1G33270,AT5G19860,AT5G37640,AT1G31050,AT5G25070,AT4G35790,AT1G09390,AT5G23760,AT4G14960,AT5G65910,AT1G14920,AT3G13510,AT4G14965,AT1G77920,AT4G05150,AT2G15670,AT5G44010,AT1G74920,AT4G04930,AT4G16140,AT1G23010,AT2G02710,AT1G72430,AT4G03600,AT5G03140,AT3G21690,AT2G17340,AT4G02480,AT3G44660,AT2G32950,AT5G51570,AT1G30210
TTWCCWWWWNNGGWW	10	4	['4-8', '12-16']	34	AT5G27150,AT1G75080,AT1G33270,AT5G19860,AT5G37640,AT1G31050,AT5G25070,AT4G35790,AT1G09390,AT5G23760,AT4G14960,AT5G65910,AT1G14920,AT3G13510,AT4G14965,AT1G77920,AT4G05150,AT2G15670,AT5G44010,AT1G74920,AT4G04930,AT4G16140,AT1G23010,AT2G02710,AT1G72430,AT4G03600,AT5G03140,AT3G21690,AT2G17340,AT4G02480,AT3G44660,AT2G32950,AT5G51570,AT1G30210
TTWCCWWWWNNGGWW	10	5	['4-8', '12-16']	34	AT5G27150,AT1G75080,AT1G33270,AT5G19860,AT5G37640,AT1G31050,AT5G25070,AT4G35790,AT1G09390,AT5G23760,AT4G14960,AT5G65910,AT1G14920,AT3G13510,AT4G14965,AT1G77920,AT4G05150,AT2G15670,AT5G44010,AT1G74920,AT4G04930,AT4G16140,AT1G23010,AT2G02710,AT1G72430,AT4G03600,AT5G03140,AT3G21690,AT2G17340,AT4G02480,AT3G44660,AT2G32950,AT5G51570,AT1G30210
TYTCCCGCC	10	1	['0-4', '4-8', '12-16', '16-20']	21	AT4G18530,AT3G62550,AT5G58760,AT3G52360,AT4G14700,AT1G21880,AT1G53450,AT2G22720,AT4G17530,AT1G15820,AT3G56910,AT2G06980,AT1G75780,AT5G54970,AT1G56430,AT5G63380,AT3G12530,AT5G03150,AT3G44630,AT3G43790,AT1G65430
TYTCCCGCC	10	2	['0-4', '4-8', '12-16', '16-20']	21	AT4G18530,AT3G62550,AT5G58760,AT3G52360,AT4G14700,AT1G21880,AT1G53450,AT2G22720,AT4G17530,AT1G15820,AT3G56910,AT2G06980,AT1G75780,AT5G54970,AT1G56430,AT5G63380,AT3G12530,AT5G03150,AT3G44630,AT3G43790,AT1G65430
TYTCCCGCC	10	3	['0-4', '4-8', '12-16', '16-20']	21	AT4G18530,AT3G62550,AT5G58760,AT3G52360,AT4G14700,AT1G21880,AT1G53450,AT2G22720,AT4G17530,AT1G15820,AT3G56910,AT2G06980,AT1G75780,AT5G54970,AT1G56430,AT5G63380,AT3G12530,AT5G03150,AT3G44630,AT3G43790,AT1G65430
TYTCCCGCC	10	4	['0-4', '4-8', '12-16', '16-20']	21	AT4G18530,AT3G62550,AT5G58760,AT3G52360,AT4G14700,AT1G21880,AT1G53450,AT2G22720,AT4G17530,AT1G15820,AT3G56910,AT2G06980,AT1G75780,AT5G54970,AT1G56430,AT5G63380,AT3G12530,AT5G03150,AT3G44630,AT3G43790,AT1G65430
TYTCCCGCC	10	5	['4-8', '12-16', '16-20', '0-4']	21	AT4G18530,AT3G62550,AT5G58760,AT3G52360,AT4G14700,AT1G21880,AT1G53450,AT2G22720,AT4G17530,AT1G15820,AT3G56910,AT2G06980,AT1G75780,AT5G54970,AT1G56430,AT5G63380,AT3G12530,AT5G03150,AT3G44630,AT3G43790,AT1G65430

YACGTGGC	10	1	['4-8']	135	AT4G01120,AT2G07738,AT1G06680,AT2G38000,AT1G16720,AT5G49120,AT1G75830,AT3G05130,AT2G47490,AT1G13440,AT1G28530,AT4G25570,AT5G47640,AT1G22370,AT1G01520,AT1G01540,AT3G48580,AT4G16515,AT2G06980,AT2G47780,AT1G56220,AT5G43850,AT3G12470,AT4G15110,AT4G14270,AT1G52220,AT1G52230,AT1G68440,AT2G45990,AT5G39570,AT1G75210,AT1G75140,AT4G39100,AT5G38510,AT3G31320,AT2G34460,AT5G37260,AT5G37290,AT1G35460,AT5G35460,AT2G24820,AT3G29320,AT4G35850,AT4G37240,AT4G37220,AT5G23050,AT2G21330,AT3G15210,AT2G04550,AT5G18340,AT2G36390,AT1G68190,AT2G41870,AT1G10960,AT5G15230,AT2G47400,AT4G31310,AT3G14930,AT2G42220,AT1G55850,AT1G79040,ATMG01330,AT5G27520,AT4G28750,AT1G54850,AT1G32470,AT5G26820,AT5G26020,AT3G20300,AT1G78240,AT5G67370,AT3G12920,AT5G06690,AT5G66570,AT1G01240,AT3G17000,AT4G25450,AT3G25530,AT5G65630,AT1G55670,AT5G05270,AT5G05300,AT1G19660,AT5G64840,AT5G05200,AT4G24190,AT5G64260,AT1G09870,AT5G64180,AT1G22850,AT1G19980,AT3G18110,AT5G03190,AT1G53090,AT3G13980,AT4G19710,AT1G18740,AT5G61530,AT3G63060,AT4G18240,AT3G63210,AT1G49580,AT1G49660,AT3G06780,AT1G18310,AT1G55480,AT5G59080,AT3G61830,AT3G61220,AT5G58770,AT5G58070,AT5G57760,AT5G57345,AT3G08650,AT3G60200,AT4G09620,AT3G05880,AT3G59660,AT4G09020,AT3G59220,AT4G08180,AT5G55120,AT1G74840,AT2G15970,AT3G03150,AT1G74880,AT3G10670,AT4G03560,AT3G10410,AT3G56050,AT2G01290,AT5G52580,AT1G11130,AT3G04860,AT5G51110
YACGTGGC	10	2	['4-8']	135	AT4G01120,AT2G07738,AT1G06680,AT2G38000,AT1G16720,AT5G49120,AT1G75830,AT3G05130,AT2G47490,AT1G13440,AT1G28530,AT4G25570,AT5G47640,AT1G22370,AT1G01520,AT1G01540,AT3G48580,AT4G16515,AT2G06980,AT2G47780,AT1G56220,AT5G43850,AT3G12470,AT4G15110,AT4G14270,AT1G52220,AT1G52230,AT1G68440,AT2G45990,AT5G39570,AT1G75210,AT1G75140,AT4G39100,AT5G38510,AT3G31320,AT2G34460,AT5G37260,AT5G37290,AT1G35460,AT5G35460,AT2G24820,AT3G29320,AT4G35850,AT4G37240,AT4G37220,AT5G23050,AT2G21330,AT3G15210,AT2G04550,AT5G18340,AT2G36390,AT1G68190,AT2G41870,AT1G10960,AT5G15230,AT2G47400,AT4G31310,AT3G14930,AT2G42220,AT1G55850,AT1G79040,ATMG01330,AT5G27520,AT4G28750,AT1G54850,AT1G32470,AT5G26820,AT5G26020,AT3G20300,AT1G78240,AT5G67370,AT3G12920,AT5G06690,AT5G66570,AT1G01240,AT3G17000,AT4G25450,AT3G25530,AT5G65630,AT1G55670,AT5G05270,AT5G05300,AT1G19660,AT5G64840,AT5G05200,AT4G24190,AT5G64260,AT1G09870,AT5G64180,AT1G22850,AT1G19980,AT3G18110,AT5G03190,AT1G53090,AT3G13980,AT4G19710,AT1G18740,AT5G61530,AT3G63060,AT4G18240,AT3G63210,AT1G49580,AT1G49660,AT3G06780,AT1G18310,AT1G55480,AT5G59080,AT3G61830,AT3G61220,AT5G58770,AT5G58070,AT5G57760,AT5G57345,AT3G08650,AT3G60200,AT4G09620,AT3G05880,AT3G59660,AT4G09020,AT3G59220,AT4G08180,AT5G55120,AT1G74840,AT2G15970,AT3G03150,AT1G74880,AT3G10670,AT4G03560,AT3G10410,AT3G56050,AT2G01290,AT5G52580,AT1G11130,AT3G04860,AT5G51110
YACGTGGC	10	3	['4-8']	135	AT4G01120,AT2G07738,AT1G06680,AT2G38000,AT1G16720,AT5G49120,AT1G75830,AT3G05130,AT2G47490,AT1G13440,AT1G28530,AT4G25570,AT5G47640,AT1G22370,AT1G01520,AT1G01540,AT3G48580,AT4G16515,AT2G06980,AT2G47780,AT1G56220,AT5G43850,AT3G12470,AT4G15110,AT4G14270,AT1G52220,AT1G52230,AT1G68440,AT2G45990,AT5G39570,AT1G75210,AT1G75140,AT4G39100,AT5G38510,AT3G31320,AT2G34460,AT5G37260,AT5G37290,AT1G35460,AT5G35460,AT2G24820,AT3G29320,AT4G35850,AT4G37240,AT4G37220,AT5G23050,AT2G21330,AT3G15210,AT2G04550,AT5G18340,AT2G36390,AT1G68190,AT2G41870,AT1G10960,AT5G15230,AT2G47400,AT4G31310,AT3G14930,AT2G42220,AT1G55850,AT1G79040,ATMG01330,AT5G27520,AT4G28750,AT1G54850,AT1G32470,AT5G26820,AT5G26020,AT3G20300,AT1G78240,AT5G67370,AT3G12920,AT5G06690,AT5G66570,AT1G01240,AT3G17000,AT4G25450,AT3G25530,AT5G65630,AT1G55670,AT5G05270,AT5G05300,AT1G19660,AT5G64840,AT5G05200,AT4G24190,AT5G64260,AT1G09870,AT5G64180,AT1G22850,AT1G19980,AT3G18110,AT5G03190,AT1G53090,AT3G13980,AT4G19710,AT1G18740,AT5G61530,AT3G63060,AT4G18240,AT3G63210,AT1G49580,AT1G49660,AT3G06780,AT1G18310,AT1G55480,AT5G59080,AT3G61830,AT3G61220,AT5G58770,AT5G58070,AT5G57760,AT5G57345,AT3G08650,AT3G60200,AT4G09620,AT3G05880,AT3G59660,AT4G09020,AT3G59220,AT4G08180,AT5G55120,AT1G74840,AT2G15970,AT3G03150,AT1G74880,AT3G10670,AT4G03560,AT3G10410,AT3G56050,AT2G01290,AT5G52580,AT1G11130,AT3G04860,AT5G51110

YACGTGGC	10	4	['4-8']	135	AT4G01120,AT2G07738,AT1G06680,AT2G38000,AT1G16720,AT5G49120,AT1G75830,AT3G05130,AT2G47490,AT1G13440,AT1G28530,AT4G25570,AT5G47640,AT1G22370,AT1G01520,AT1G01540,AT3G48580,AT4G16515,AT2G06980,AT2G47780,AT1G56220,AT5G43850,AT3G12470,AT4G15110,AT4G14270,AT1G52220,AT1G52230,AT1G68440,AT2G45990,AT5G39570,AT1G75210,AT1G75140,AT4G39100,AT5G38510,AT3G31320,AT2G34460,AT5G37260,AT5G37290,AT1G35460,AT5G35460,AT2G24820,AT3G29320,AT4G35850,AT4G37240,AT4G37220,AT5G23050,AT2G21330,AT3G15210,AT2G04550,AT5G18340,AT2G36390,AT1G68190,AT2G41870,AT1G10960,AT5G15230,AT2G47400,AT4G31310,AT3G14930,AT2G42220,AT1G55850,AT1G79040,ATMG01330,AT5G27520,AT4G28750,AT1G54850,AT1G32470,AT5G26820,AT5G26020,AT3G20300,AT1G78240,AT5G67370,AT3G12920,AT5G06690,AT5G66570,AT1G01240,AT3G17000,AT4G25450,AT3G25530,AT5G65630,AT1G55670,AT5G05270,AT5G05300,AT1G19660,AT5G64840,AT5G05200,AT4G24190,AT5G64260,AT1G09870,AT5G64180,AT1G22850,AT1G19980,AT3G18110,AT5G03190,AT1G53090,AT3G13980,AT4G19710,AT1G18740,AT5G61530,AT3G63060,AT4G18240,AT3G63210,AT1G49580,AT1G49660,AT3G06780,AT1G18310,AT1G55480,AT5G59080,AT3G61830,AT3G61220,AT5G58770,AT5G58070,AT5G57760,AT5G57345,AT3G08650,AT3G60200,AT4G09620,AT3G05880,AT3G59660,AT4G09020,AT3G59220,AT4G08180,AT5G55120,AT1G74840,AT2G15970,AT3G03150,AT1G74880,AT3G10670,AT4G03560,AT3G10410,AT3G56050,AT2G01290,AT5G52580,AT1G11130,AT3G04860,AT5G51110
YACGTGGC	10	5	['4-8']	135	AT4G01120,AT2G07738,AT1G06680,AT2G38000,AT1G16720,AT5G49120,AT1G75830,AT3G05130,AT2G47490,AT1G13440,AT1G28530,AT4G25570,AT5G47640,AT1G22370,AT1G01520,AT1G01540,AT3G48580,AT4G16515,AT2G06980,AT2G47780,AT1G56220,AT5G43850,AT3G12470,AT4G15110,AT4G14270,AT1G52220,AT1G52230,AT1G68440,AT2G45990,AT5G39570,AT1G75210,AT1G75140,AT4G39100,AT5G38510,AT3G31320,AT2G34460,AT5G37260,AT5G37290,AT1G35460,AT5G35460,AT2G24820,AT3G29320,AT4G35850,AT4G37240,AT4G37220,AT5G23050,AT2G21330,AT3G15210,AT2G04550,AT5G18340,AT2G36390,AT1G68190,AT2G41870,AT1G10960,AT5G15230,AT2G47400,AT4G31310,AT3G14930,AT2G42220,AT1G55850,AT1G79040,ATMG01330,AT5G27520,AT4G28750,AT1G54850,AT1G32470,AT5G26820,AT5G26020,AT3G20300,AT1G78240,AT5G67370,AT3G12920,AT5G06690,AT5G66570,AT1G01240,AT3G17000,AT4G25450,AT3G25530,AT5G65630,AT1G55670,AT5G05270,AT5G05300,AT1G19660,AT5G64840,AT5G05200,AT4G24190,AT5G64260,AT1G09870,AT5G64180,AT1G22850,AT1G19980,AT3G18110,AT5G03190,AT1G53090,AT3G13980,AT4G19710,AT1G18740,AT5G61530,AT3G63060,AT4G18240,AT3G63210,AT1G49580,AT1G49660,AT3G06780,AT1G18310,AT1G55480,AT5G59080,AT3G61830,AT3G61220,AT5G58770,AT5G58070,AT5G57760,AT5G57345,AT3G08650,AT3G60200,AT4G09620,AT3G05880,AT3G59660,AT4G09020,AT3G59220,AT4G08180,AT5G55120,AT1G74840,AT2G15970,AT3G03150,AT1G74880,AT3G10670,AT4G03560,AT3G10410,AT3G56050,AT2G01290,AT5G52580,AT1G11130,AT3G04860,AT5G51110
YCYYACCWACC	10	1	['4-8', '8-12', '12-16', '20-24']	18	AT5G50990,AT5G27380,AT3G04780,AT3G17930,AT1G76150,AT2G36880,AT4G02910,AT1G04910,AT1G49740,AT1G52760,AT2G37040,AT2G44300,AT2G22720,AT2G40890,AT3G03160,AT3G26570,AT3G24590,AT4G08690
YCYYACCWACC	10	2	['4-8', '8-12', '12-16', '20-24']	18	AT5G50990,AT5G27380,AT3G04780,AT3G17930,AT1G76150,AT2G36880,AT4G02910,AT1G04910,AT1G49740,AT1G52760,AT2G37040,AT2G44300,AT2G22720,AT2G40890,AT3G03160,AT3G26570,AT3G24590,AT4G08690
YCYYACCWACC	10	3	['4-8', '8-12', '12-16', '20-24']	18	AT5G50990,AT5G27380,AT3G04780,AT3G17930,AT1G76150,AT2G36880,AT4G02910,AT1G04910,AT1G49740,AT1G52760,AT2G37040,AT2G44300,AT2G22720,AT2G40890,AT3G03160,AT3G26570,AT3G24590,AT4G08690
YCYYACCWACC	10	4	['4-8', '8-12', '12-16', '20-24']	18	AT5G50990,AT5G27380,AT3G04780,AT3G17930,AT1G76150,AT2G36880,AT4G02910,AT1G04910,AT1G49740,AT1G52760,AT2G37040,AT2G44300,AT2G22720,AT2G40890,AT3G03160,AT3G26570,AT3G24590,AT4G08690
YCYYACCWACC	10	5	['4-8', '8-12', '12-16', '20-24']	18	AT5G50990,AT5G27380,AT3G04780,AT3G17930,AT1G76150,AT2G36880,AT4G02910,AT1G04910,AT1G49740,AT1G52760,AT2G37040,AT2G44300,AT2G22720,AT2G40890,AT3G03160,AT3G26570,AT3G24590,AT4G08690
YTYYMMCMAMCMMC	10	1	['4-8', '8-12', '20-24']	17	AT1G03055,AT3G12110,AT1G21400,AT4G21180,AT2G36880,AT1G53230,AT1G15950,AT2G46170,AT4G05210,AT2G40890,AT1G47530,AT2G36430,AT5G61410,AT3G52470,AT4G21990,AT3G51520,AT3G51730
YTYYMMCMAMCMMC	10	2	['4-8', '8-12', '20-24']	17	AT1G03055,AT3G12110,AT1G21400,AT4G21180,AT2G36880,AT1G53230,AT1G15950,AT2G46170,AT4G05210,AT2G40890,AT1G47530,AT2G36430,AT5G61410,AT3G52470,AT4G21990,AT3G51520,AT3G51730
YTYYMMCMAMCMMC	10	3	['4-8', '8-12', '20-24']	17	AT1G03055,AT3G12110,AT1G21400,AT4G21180,AT2G36880,AT1G53230,AT1G15950,AT2G46170,AT4G05210,AT2G40890,AT1G47530,AT2G36430,AT5G61410,AT3G52470,AT4G21990,AT3G51520,AT3G51730

YTYYMMCMAMCMMC	10	4	['4-8', '8-12', '20-24']	17	AT1G03055,AT3G12110,AT1G21400,AT4G21180,AT2G36880,AT1G53230,AT1G15950,AT2G46170,AT4G05210,AT2G40890,AT1G47530,AT2G36430,AT5G61410,AT3G52470,AT4G21990,AT3G51520,AT3G51730
YTYYMMCMAMCMMC	10	5	['4-8', '8-12', '20-24']	17	AT1G03055,AT3G12110,AT1G21400,AT4G21180,AT2G36880,AT1G53230,AT1G15950,AT2G46170,AT4G05210,AT2G40890,AT1G47530,AT2G36430,AT5G61410,AT3G52470,AT4G21990,AT3G51520,AT3G51730
