# Supplementary material for: Computational exploration of cis-regulatory modules in rhythmic expression data using the “Exploration of Distinctive CREs and CRMs” (EDCC) and “CRM Network Generator” (CNG) programs
Source: PLoS One. 2018 Jan 3;13(1):e0190421. doi: 10.1371/journal.pone.0190421 (PMC5752016; doi:10.1371/journal.pone.0190421)
Supplement: S3 Table — Given are 21 CRE pairs that have been found to correlate with a shift in peak expression time of circadianly expressed genes in Arabidopsis. All listed pairs occurred in at least 30 promoters and deviated from the background by at least five standard deviations in all five EDCC runs. (PDF) [file pone.0190421.s006.pdf]

S3 Table. Candidate CRE pairs that were used for CNG analysis						
**single sequence**	**interesting timepoints**	**sum of matches**	**distance test pval**	**order test pval**	**bowley skewness**	**genes**
ATGNTTCA,ACGTGGC	['16-20']	39	0.923223135	7.03E-05	-0.128113879	AT4G28750,AT2G37450,AT1G75210,AT1G32470,AT4G39100,AT4G39150,AT5G19860,AT5G26020,AT3G06780,AT1G78240,AT1G62620,AT4G13010,AT5G24970,AT4G37640,AT1G01240,AT5G65990,AT4G15560,AT2G18350,AT5G23050,AT1G17460,AT1G20696,AT4G34190,AT1G19660,AT3G19100,AT5G55120,AT1G78020,AT5G54770,AT5G53880,AT4G03600,AT1G71020,AT3G56490,AT3G56260,AT1G05570,AT1G11130,AT1G55850,AT5G13650,AT3G54500,AT2G45990,AT5G61530
AAAATATCT,ATGGCNNC	['8-12']	31	0.219729589	0.47312966	-0.076436479	AT2G15090,AT3G63160,AT5G11670,AT4G28660,AT5G08410,AT2G22300,AT3G08030,AT4G37300,AT2G28840,AT4G37480,AT4G26530,AT3G26740,AT3G60260,AT1G27440,AT4G25660,AT1G07190,AT3G20810,AT1G59870,AT3G04550,AT5G64860,AT3G47860,AT4G33985,AT4G05370,AT1G28050,AT3G06410,AT2G45990,AT4G30660,AT3G09250,AT5G01950,AT4G29510,AT5G61590
CCNNCACN,GTGATCAC	['0-4']	32	0.308358121	1	0.117757009	AT4G39990,AT3G26280,AT1G04170,AT5G24930,AT1G16470,AT3G07880,AT3G16320,AT2G29310,AT2G01860,AT1G70660,AT2G20260,AT1G01620,AT3G60020,AT1G11680,AT1G74640,AT2G47910,AT1G80090,AT1G13540,AT5G05170,AT1G70000,AT1G12845,AT1G75690,AT1G42970,AT1G26760,AT4G22540,AT3G15040,AT4G03050,AT1G23210,AT1G47530,AT1G73390,AT5G41060,AT1G76990
TGTCACA,TGAGTCA	['4-8']	31	0.263942045	1	0.146718147	AT1G42580,AT5G27380,AT5G58950,AT5G49120,AT1G05030,AT3G04460,AT5G07960,AT4G26700,AT5G07440,AT1G12990,AT5G57660,AT3G60020,AT5G57040,AT1G18990,AT3G10720,AT4G14430,AT3G26932,AT1G16880,AT2G33810,AT3G14640,AT3G57090,AT1G63080,AT5G03040,AT1G60970,AT1G71020,AT5G63380,AT5G62540,AT3G22060,AT3G07550,AT2G26900,AT1G32360
ACANTACN,MCACGTGGC	['4-8']	34	0.332917264	3.86E-05	-0.160483871	AT4G28750,AT3G63060,AT3G63210,AT4G18240,AT1G32470,AT3G06780,AT1G55480,AT1G06680,AT2G38000,AT5G49120,AT4G25570,AT5G57760,AT5G66570,AT1G01520,AT1G01240,AT4G25450,AT5G65630,AT1G55670,AT4G16515,AT5G05270,AT5G05300,AT1G19660,AT4G24190,AT5G64260,AT2G04550,AT5G43850,AT3G12470,AT4G03560,AT1G10960,AT4G31310,AT3G13980,AT2G01290,AT1G52220,AT1G52230
AGNGATAN,MCACGTGGC	['4-8']	33	0.174405337	0.035082033	-0.022526146	AT5G39570,AT3G63210,AT1G32470,AT2G34460,AT3G06780,AT1G18310,AT1G55480,AT5G59080,AT5G49120,AT5G58070,AT4G35850,AT5G57760,AT5G66570,AT4G25450,AT3G60200,AT3G59660,AT4G16515,AT3G59220,AT5G05300,AT1G19660,AT4G08180,AT5G05200,AT5G64840,AT5G64260,AT3G15210,AT1G22850,AT5G43850,AT3G12470,AT1G10960,AT3G13980,AT1G18740,AT1G52230,AT3G04860
TGTGNGNA,TAGTGGAT	['4-8']	32	0.003427802	0.596614896	0.107826087	AT1G54830,AT1G27090,AT2G26520,AT1G67510,AT5G60210,AT5G19860,AT4G12800,AT5G58910,AT3G61120,AT5G08580,AT4G12060,AT3G16470,AT1G05960,AT1G22360,AT5G66950,AT1G22280,AT1G80840,AT1G01430,AT1G64640,AT5G44520,AT3G07200,AT2G40080,AT2G46170,AT5G18170,AT4G23290,AT3G57090,AT1G61390,AT2G48020,AT5G62160,AT1G30220,AT2G38820,AT2G38800

ACACCGG,AAGNGTNG	['12-16']	30	0.340273847	0.200488422	-0.092592593	AT1G37537,AT1G31500,AT2G06005,AT4G28210,AT1G01940,AT4G00400,AT3G02690,AT3G20390,AT3G06510,AT5G20280,AT2G38130,AT2G25210,AT5G07400,AT5G06720,AT5G23240,AT3G59770,AT5G46760,AT3G26380,AT3G10810,AT4G16146,AT4G24350,AT2G04390,AT4G33700,AT2G16070,AT1G05850,AT3G14000,AT3G24170,AT1G07890,AT5G62200,AT1G53210
CNANAGAA,GACGTGTA	['16-20']	32	0.203803725	0.002102402	0.012838802	AT5G26790,AT2G34430,AT3G28270,AT1G32060,AT3G12710,AT1G62780,AT2G39080,AT1G66970,AT3G03710,AT4G26700,AT3G13790,AT3G16910,AT2G20260,AT5G23870,AT5G23050,AT5G19260,AT3G47960,AT5G56030,AT4G07425,AT5G55530,AT1G58110,AT1G12800,AT2G21970,AT2G44550,AT1G20190,AT5G64170,AT1G23010,AT1G74750,AT4G15730,AT3G57190,AT3G14270,AT5G12250
ACANTACN,ATCCAACC	['4-8']	44	0.491032141	0.022628841	-0.201816347	AT1G63630,AT3G63310,AT4G39400,AT4G00960,AT2G26600,AT2G26520,AT1G75140,AT5G26770,AT3G01750,AT3G62100,AT5G26020,AT2G38400,AT4G12730,AT1G16720,AT5G49120,AT1G73820,AT3G52380,AT1G21500,AT3G07890,AT3G27090,AT3G07880,AT1G06010,AT4G15440,AT4G36470,AT4G09760,AT1G21680,AT3G59060,AT5G65000,AT5G18170,AT1G12780,AT4G16140,AT5G03240,AT5G53880,AT2G30570,AT1G08640,AT5G53770,AT5G02820,AT1G73020,AT5G02830,AT1G56720,AT1G72170,AT5G61850,AT4G00830,AT4G30060
ACACATG,AAAATATCT	['8-12']	30	0.018547343	1	-0.177777778	AT1G76590,AT3G02630,AT1G78210,AT1G73760,AT2G28840,AT2G22720,AT5G23870,AT1G80760,AT1G01500,AT1G26940,AT2G18220,AT1G20620,AT5G64860,AT1G19650,AT1G34440,AT4G33985,AT2G23840,AT4G16860,AT4G33500,AT2G33830,AT2G02710,AT2G30600,AT1G08660,AT2G03680,AT3G09390,AT1G07700,AT1G44100,AT3G16040,AT1G62430,AT5G61590
AATNCCNC,AAAATATCT	['8-12']	52	0.725781914	0.126347076	-0.089820359	AT2G15090,AT2G10370,AT1G77000,AT4G39960,AT3G53800,AT1G34340,AT4G28660,AT3G42800,AT1G67970,AT4G27780,AT1G68820,AT5G49120,AT1G78210,AT4G29840,AT1G73760,AT3G27090,AT2G36720,AT3G08030,AT2G21660,AT3G26740,AT5G57630,AT4G10920,AT2G22720,AT3G59940,AT1G55730,AT1G08080,AT3G59480,AT1G59870,AT5G64860,AT5G64260,AT1G58150,AT4G16860,AT1G12970,AT4G16950,AT2G33810,AT2G02710,AT2G02760,AT5G15710,AT4G03600,AT3G46440,AT4G14270,AT2G29650,AT1G78460,AT1G73020,AT5G40500,AT4G02420,AT3G09250,AT5G13950,AT5G01950,AT4G29510,AT5G12150,AT2G45990
GACGTGTA,CNNACANC	['16-20']	30	0.78779266	0.016124802	0.043723554	AT5G26790,AT1G32060,AT3G12710,AT2G39080,AT3G03710,AT3G13790,AT3G16910,AT2G20260,AT5G23870,AT5G23050,AT5G05520,AT3G47960,AT5G56030,AT2G36885,AT2G37130,AT1G12800,AT2G21970,AT1G20190,AT5G64170,AT1G23010,AT1G74750,AT3G57190,AT5G63135,AT5G03510,AT1G65210,AT4G30610,AT1G49250,AT1G79040,AT5G51110,AT5G12250
TCNTNAGA,CAAAACGC	['16-20']	31	0.239074261	1	0.080233079	AT5G61130,AT1G01970,AT2G39400,AT1G14140,AT4G28100,AT5G48140,AT3G50530,AT4G16760,AT2G18230,AT5G46110,AT5G56170,AT3G19930,AT4G34590,AT3G59052,AT2G06510,AT3G59400,AT1G19650,AT5G55220,AT1G54390,AT5G17170,AT3G56410,AT5G63380,AT2G25870,AT4G02940,AT3G14900,AT1G78570,AT5G02880,AT5G14800,AT5G52320,AT5G62220,AT4G29670

AAAATATCT,GAANGAGA	['8-12']	56	0.612288594	0.228805541	-0.086294416	AT3G63160,AT4G29040,AT1G34340,AT5G38510,AT3G02630,AT4G39270,AT1G67970,AT2G17220,AT4G27780,AT1G18460,AT3G28340,AT5G08410,AT4G12500,AT5G08520,AT3G61470,AT5G58120,AT2G37520,AT1G06040,AT5G48250,AT1G66980,AT4G26130,AT5G23870,AT3G59940,AT4G25660,AT1G60000,AT1G04710,AT1G62960,AT3G20810,AT1G33780,AT4G24390,AT4G33985,AT4G05370,AT1G51790,AT4G16860,AT4G33500,AT1G69780,AT3G57170,AT5G63820,AT2G18170,AT5G03220,AT1G48330,AT2G30600,AT1G29400,AT1G78600,AT1G73020,AT1G29670,AT5G14730,AT4G31050,AT3G09390,AT4G30650,AT1G44100,AT5G13950,AT1G35150,AT1G14350,AT5G01950,AT4G02260
ANCACATG,AAAATATCT	['8-12']	36	0.124545257	1	-0.256385998	AT1G34340,AT1G22770,AT4G00400,AT1G18330,AT3G52950,AT2G22300,AT1G78210,AT4G27710,AT4G37300,AT2G28840,AT5G48250,AT3G26740,AT2G22720,AT1G01470,AT2G18220,AT3G05800,AT1G07040,AT1G20620,AT1G35350,AT5G64860,AT4G33985,AT4G16860,AT2G33810,AT2G33830,AT2G18170,AT2G30600,AT3G46290,AT1G08660,AT3G21560,AT3G55450,AT1G14380,AT1G44100,AT3G15570,AT3G26570,AT5G61590,AT1G32440
CTCATTTN,AGATCCAA	['4-8']	30	0.833536409	1	-0.346912794	AT4G18240,AT4G39400,AT5G50335,AT3G62700,AT4G00620,AT2G18790,AT4G38830,AT4G13250,AT5G49120,AT5G08580,AT1G05000,AT3G27090,AT4G11840,AT1G66930,AT5G24530,AT2G42900,AT2G04900,AT5G06830,AT5G23140,AT3G19820,AT5G56220,AT3G19880,AT4G08320,AT4G08090,AT5G18850,AT5G55480,AT3G15290,AT5G53350,AT5G51720,AT5G12210
CATGCATG,NGCNTGAA	['4-8']	30	0.012693686	0.584664712	-0.274600721	AT1G38065,AT4G18270,AT5G11420,AT4G28420,AT1G15310,AT5G49480,AT5G59080,AT5G58910,AT2G36690,AT1G15520,AT3G08030,AT3G29370,AT5G66920,AT1G26590,AT1G09430,AT3G26380,AT4G36580,AT3G48940,AT1G64640,AT5G56260,AT3G04650,AT3G48110,AT5G64630,AT2G27490,AT4G31390,AT2G42160,AT5G52780,AT1G47530,AT1G72140,AT1G62340
CATGCATG,AGNAACAA	['4-8']	34	0.982198087	0.607591361	-0.049627792	AT5G04130,AT5G11420,AT3G10260,AT4G28420,AT1G15310,AT1G68100,AT5G49480,AT5G59080,AT2G36690,AT4G27320,AT3G08030,AT5G07860,AT2G21660,AT1G06010,AT1G13180,AT1G26590,AT4G17070,AT1G13130,AT1G09430,AT4G09570,AT5G66180,AT3G26380,AT3G19930,AT5G45830,AT5G56260,AT3G51840,AT4G34250,AT4G04930,AT2G22500,AT4G31390,AT2G42160,AT1G47530,AT1G72140,AT1G62340
AAAATATCT,CCAGTG	['8-12']	38	0.849046506	0.62710257	-0.417558887	AT4G29040,AT1G24440,AT4G39270,AT4G29510,AT1G18330,AT1G78210,AT3G61470,AT4G37300,AT1G28600,AT1G06040,AT3G26740,AT5G57630,AT3G07840,AT5G57345,AT5G57040,AT3G05880,AT1G74470,AT1G07420,AT1G07190,AT4G24390,AT1G80280,AT2G46530,AT3G57170,AT3G06410,AT2G02760,AT2G18170,AT3G27300,AT1G08660,AT2G22430,AT4G31550,AT1G29670,AT2G32100,AT4G31050,AT4G02260,AT5G13950,AT3G15570,AT3G42800,AT3G63170
ATACGTGT,TAACAAA	['0-4']	40	0.784836248	0.635828003	-0.017612524	AT2G24880,AT3G53720,AT3G22420,AT1G15400,AT1G78670,AT4G27670,AT1G16520,AT3G30380,AT3G51240,AT1G28960,AT1G01470,AT5G66410,AT1G80840,AT1G21000,AT3G49220,AT4G16845,AT1G17360,AT3G59060,AT1G64860,AT1G80090,AT3G19500,AT4G16860,AT1G12970,AT2G35940,AT1G68920,AT3G13110,AT1G79770,AT1G69530,AT4G20160,AT1G54880,AT1G56680,AT4G19710,AT1G05720,AT5G04140,AT3G15030,AT5G62130,AT3G43790,AT4G19450,AT5G40450,AT1G76990
