# Supplementary material for: Computational exploration of cis-regulatory modules in rhythmic expression data using the “Exploration of Distinctive CREs and CRMs” (EDCC) and “CRM Network Generator” (CNG) programs
Source: PLoS One. 2018 Jan 3;13(1):e0190421. doi: 10.1371/journal.pone.0190421 (PMC5752016; doi:10.1371/journal.pone.0190421)
Supplement: S1 File — The manual is also available as.html file under https://sourceforge.net/projects/edcc/files/edcc_cng.zip/download. (DOCX) [file pone.0190421.s007.docx]

**EDCC-CNG - Manual**

Copyright (C) 2017 PSB & TT.
Permission is granted to copy, distribute and/or modify this document under the terms of the GNU Free Documentation License, Version 1.3 or any later version published by the Free Software Foundation; with no Invariant Sections, no Front-Cover Texts, and no Back-Cover Texts. A copy of the license is included in the section entitled "GNU Free Documentation License".

**Index**

- Introduction
- Installation
- ...2.1 Installation guide
- [...2.2 Docker container](file:///E:\Manuscripts\CRM\Revision\submit\docs\manual.html#docker)
- Tutorial
- ...3.1 Aims
- ...3.2 Preparing the data
- ...3.3 Finding potential CREs or CRMs with EDCC
- ...3.4 Categorizing potential CRMs with CNG
- ...3.5 Re-using generated categorization networks with CNG Viewer
- ...3.6 Mutating a CRM of interest
- Overview of GUI commands
- ...4.1 EDCC
- ...4.2 CNG
- ...4.3 CNG Viewer
- Addendum: GNU Free Documentation License

**1. Introduction**

EDCC-CNG is a free and open-source program suite which can be used to identify, analyze and categorize cis-regulatory elements (CRE) and/or cis-regulatory modules (CRM) of organisms. It is written in Python 3.6 (using SciPy) and R 3.3. The programs provide a GUI - and additionally for the case of EDCC a CLI - to access their functions. EDCC-CNG is written by Pavlos Stephanos Bekiaris and Tobias Tekath, based on ideas of Prof. Dr. Dorothee Staiger and Dr. Selahattin Danisman of the University of Bielefeld. The whole program suite consists of the following programs:

1. EDCC: Identifies potential CREs or CRMs from a given set of sequences is a CRM, and analyzes the CRM's position, order and distance behavior.
2. CRM Network Generator (CNG): Categorizes identified CRMs with a two-class neural network, using data from the statistical analyses of EDCC.
3. CNG Viewer: Can apply a two-class neural network generated by CNG on identified and analyzed CRMs which were not used in the network's training set.

The methods and a scientific usage example (in *Arabidopsis thaliana*) of EDCC-CNG are further described in its publication **(Citation)** . This manual explains the GUIs and their usage, as well as the installation of EDCC-CNG.

**2. Installation**

To be able to use EDCC-CNG correctly, you have to perform the following steps:

- Make sure that you have installed Python 3.6 or greater (<https://www.python.org/>) on your system.
- In addition to Python itself, you also have to install (if not done) the Python packages "tkinter" (which is pre-installed on many Python distributions) and "SciPy" (<https://www.scipy.org/>).
- To run CNG correctly, you have (if not done) to install R 3.3 or greater (<https://www.r-project.org/>). Additionally, you have to add R's "RScript" executable to your system's PATH variable.
- After Python and R are installed and set up, you can run the "edcc_gui.py", "edcc_console.py", "network_generator_gui.py", or "network_viewer.py" using the "python" command.
- EDCC-CNG can read databases about the circadian rhythm of genes in the form of the CSV file format, using semicolons as row separators. This CSV must provide a row (with the header "Name") which contains the gene names, a row (with the header "Phase") which contains the maximal expression peak time, and a row (with the header "Sequence") with the DNA sequences of the genes.

**2.1 Installation guide**

This guide will show you how to install the necessary dependencies of EDCC-CNG. First, we will install a Python 3 environment. For convenience, we will rely on the [miniconda package](https://conda.io/miniconda.html). Download the appropriate setup file for your OS. Please double-check to choose the **Python 3** version.

Execute the downloaded setup file. If you use a Unix-based OS, you may have to mark the script as executable with

chmod +x setup_name.sh

before executing it. Then simply follow the setup, if you are asked to add the installation directory to your PATH, please answer 'yes'. Now open up a **new**terminal or CMD console and try

python -V

If the installation was successfully added to your PATH (and you chose the right setup), the shown version number should be above 3.6. Next, install the necessary Python packages Numpy and Scipy using the conda installer:

conda install numpy scipy

**For macOS 10.8 or greater:**

In order to use the pre-installed Tkinter package, please refer to [Anaconda FAQ](https://support.continuum.io/customer/en/portal/articles/2621153-anaconda-tkinter-with-python-3-in-os-x)

Now Python 3 and its dependencies are installed and you should be able to run edcc_console.py and edcc_gui.py on your system. You can make sure everything went right by changing to your edcc_cng directory and executing

python edcc_console.py -q AAAATATCT

or

python edcc_gui.py

and using 'AAAATATCT' (the evening element) as an example CRE query. The evening element is a well-known CRE and should be marked as 'interesting' and, among other information, a list of genes containing this CRE should be shown.

Now only a R installation is needed to also run the CNG network generator and viewer. Windows and macOS users can download pre-compiled setups from [R-Project](https://cran.r-project.org/mirrors.html). There is also a section for Unix-based users, depending on your distro you can try

sudo apt-get install r-base

or for Red Hat Linux (centOS etc.) download EPEL for your Version <https://fedoraproject.org/wiki/EPEL> and install the downloaded RPM file with

sudo rpm -Uvh *your_file*.rpm

sudo yum update

sudo yum install R

For Windows you need to add the Rscript.exe (from *R_installation_directory*/bin/)to your PATH variable. For other OS this should happen automatically. If you can start R from your console with the command 'Rscript' (Windows) or 'R' (Unix), the installation was successful. The last step is to add some R packages with the command

Rscript -e "install.packages(c('smacof','ggrepel','DescTools'),repo='http://cran.us.r-project.org')"

or

R -e "install.packages(c('smacof','ggrepel','DescTools'),repo='http://cran.us.r-project.org')"

respectively.

**2.2 Docker container**

For systems where a local installation of EDCC-CNG and its dependencies is not favoured, we provide a Docker container file. Please note that you must have a working and running Docker environment to execute the following commands. Please also note that due to virtualization the performance of the programs in the Docker container might be way worse than on a native system. To build a working image from the file, navigate to the */docker* directory and execute

docker build -t edcc_cng .

The script downloads and prepares everything needed for a working image including the EDCC-CNG suite. Then just run the image to start the EDCC command line version with the evening element as input query:

docker run edcc_cng

Alternatively, to have an interactive shell:

docker run -ti edcc_cng /bin/bash

If you have a native Unix-based system with a working displayserver you can also run the GUI programs from the Docker image. Please note that a Unix-based system in a VM will not be able to display them. The commands to display the different GUI applications are:

docker run -ti --rm -e DISPLAY=$DISPLAY -v /tmp/.X11-unix:/tmp/.X11-unix edcc_cng python edcc_gui.py

docker run -ti --rm -e DISPLAY=$DISPLAY -v /tmp/.X11-unix:/tmp/.X11-unix edcc_cng python network_generator_gui.py

docker run -ti --rm -e DISPLAY=$DISPLAY -v /tmp/.X11-unix:/tmp/.X11-unix edcc_cng python network_viewer_gui.py

Alternatively, you can start the bash and call the programs one after another from there:

docker run -ti --rm -e DISPLAY=$DISPLAY -v /tmp/.X11-unix:/tmp/.X11-unix edcc_cng /bin/bash

**3 Tutorial**

**3.1 Aims**

In this tutorial, you will learn step-by-step how you can use the EDCC-CNG program suite to find and categorize potential *cis*-regulatory modules (CRM). In our use case we will find and categorize potential CRMs, which may be involved in the regulation of the circadian rhythm of the model plant *Arabidopsis thaliana*. For more information about this biological subject, you may read the associated publication of this program suite !. In this publication, you can also find more information about the CRE/CRM search and categorization methods used by the EDCC-CNG program suite.

In order to be able to follow the tutorial's instructions, you need a correctly installed and working EDCC-CNG program suite. Consult this manual's "Installation guide" if you have problems to install and run the EDCC-CNG program suite.

All data used in this tutorial is part of the EDCC-CNG release package and can be found at [SourceForge](https://sourceforge.net/projects/edcc/).

The first step in our EDCC-CNG usage example is the generation of data files which can be read by the potential CRE/CRM finder EDCC.

**3.2 Preparing the data**

As input data, EDCC needs a file with a list of nucleotide sequences in which potential CRMs shall be searched. In addition, each nucleotide sequence of this list needs an identifier (the "name") as well as a numeric value (the "phase").

As we are looking for circadian regulated genes of *A. thaliana*, we will use a list of genes which were identified as circadian regulated. This list was generated by analysing the microarray experiment E-MEXP-1304 (Michael et al., 2008!) with the ARSER package (Yang & Su, 2010!). In our input file for EDCC, the list of nucleotide sequences is the list of promoter regions (defined as the 1000 bp before each gene's transcription start site) of the identified circadian regulated genes. In addition, the names are the TAIR identifiers of each identified circadian regulated gene. In general, the names of each gene have to be unique. The phases are the calculated time of the identified transcription rate peak of each gene.

The input file itself needs to be in the "character separated values" (CSV) format. I.e., in the first line, EDCC reads CSV files which use the semicolon as separator. The first line of these CSV files consists the row titles ("Name", "Phase" and "Sequence"), again separated by semicolons. The following lines contain each gene's data separated by a semicolon, i.e., each gene's name, phase, and sequence.

In our use case, we will use an already prepared CSV files with the data from the microarray experiment (Michael et al., 2008!) and ARSER (Yang & Su, 2010!). The first lines of this file look like this (the "..." stand for a longer sequence and are not included in the actual file):

Name;Phase;Sequence

ATMG00650;20.773146;GCGGAGGGAAGGGATCTCTTTTCTGCAACGAAAAAAAAAAACGGAGCAGATTTGACTCGGCACAACCTAACGATACATCCAA...

ATMG01040;7.9104886;TTGTTGAACTCGAGGTTGGTAATAAACTGAAGCTGCTCAGTCTTCTAGCATAGCGGAGTGATATATATCGAAGAATGAACTT...

ATMG00630;26.14417;GTACTGTTGCTAGAGAGCTGCTTTCCATCTATCTTCCTACATGAAAGGATCTAAGTCTATCCAAATAAAATAGCCCAGAAAAT...

This prepared file is part of the EDCC-CNG release and can be found at */data/total_database.csv*.

As already mentioned, EDCC can use this file to search and identify potential CRMs in it. In the next chapter, you will learn how EDCC itself can be used.

**3.3 Finding potential CREs or CRMs with EDCC**

Run "edcc_gui.py" to open EDCC's Graphical User Interface (GUI). As you can see, EDCC provides many ways to change the parameters of the search for potential CRMs. In this tutorial, we will only discuss the most important options. All other options are explained in [Chapter 4.1](file:///E:\Manuscripts\CRM\Revision\submit\docs\manual.html#overview-edcc). As already mentioned, the associated publication of this program suite (!) provides much more information about EDCC's computational methods. Please keep in mind that, depending on your used parameters and input data, EDCC can heavily use the resources of your computer. Based on your amount of RAM and CPU cores the execution of other programs while running an EDCC search can be extremely slowed or completely halted.

By clicking on the "Set database..." button, you can choose a file containing data in the CSV format (as described in the [previous chapter](file:///E:\Manuscripts\CRM\Revision\submit\docs\manual.html#tutorial-aims), i.e. the genes with their sequence, name, and a numeric value). By default, the database we will use for our example is preselected.

In the text field on the top of the GUI, you can write in sequences which shall be searched in the database file. You can search for potential CREs by typing in one sequence per line. You can search for CRMs by separating the sequences in a line with a comma. Instead of writing the sequences into the text field, it is also possible to load a text file containing the potential CREs/CRMs. In our case, we load */data/tutorial/list_of_atcoecis_motifs_doubled.txt*. This file contains over 1.5 million potential CRMs; it was created by pairing a list of 1755 single CREs from the AtCOEcis database (Vandepoele et al., Plant Physiol. 2009) with itself. The original file with the 1755 CREs is also present as */data/tutorial/list_of_atcoecis_motifs.txt*. To load the CRM file, click on the "Change..." button under the text field and select the list_of_atcoecis_motifs_doubled.txt file.

Because of the vast amount of CRMs in the input file, we determine a harder threshold a potential CRM has to pass. This means, that genes whose promoter region contain the CRM have to show a more exceptional expression pattern. To raise the threshold, we increase the standard deviation factor ("Std. deviation factor") to 6. This will reduce the number of found potential CRMs and speed up the following computations. If you do not want to raise the threshold this far, but still want acceptable computation time, you can disable the position analyses (giving the -y flag to the CLI version). The statistical tests that are performed during position analyses take quite some time and have to be performed for every potential CRM that passed the threshold.

In order to search these sequences in the database file, you just have to click on "Start" at the bottom of the GUI's window. Before starting please consider the setting the number of CPU cores the search shall occupy. By default, EDCC uses all available cores. Depending on your computer's capabilities, the search may take 10+ minutes.

If you do not want to use the GUI version of EDCC, you can execute the same search by changing to the */edcc_cng* directory and executing

python edcc_console.py -f ./data/tutorial/list_of_atcoecis_motifs_doubled.txt -s 6 -o tutorial_output.txt

As the search is completed, a new window appears. This window contains a list of all searched potential CRE/CRM sequences which showed an exceptional expression pattern according to the defined threshold. If none of the given sequences were determined to be a potential CRE/CRM, the output would just be "No interesting matches.".

The output itself is in CSV format, using the semicolon as separator. These CSVs are different from EDCC's input files: In the first row ("single sequence"), the input nucleotide sequence of the potential CRE/CRM is shown. The second row ("interesting timepoints") shows all time periods in which an overrepresentation of a CRM was found. The third row ("sum of matches") contains the number of given database file sequences in which the potential CRE/CRM was found. The last row ("genes") lists the names of all of these genes. All other rows only contain a content if a CRM ("distance test pval", "order test pval", "bowley skewness"), and not a CRE, is searched. Their meaning is explained in the publication !.

You can use this file to already analyze the list of potential CRMs. However, given a long list of potential CRMs, it might be useful to categorize the CRMs according to their statistical features. In order to categorize the list of potential CRMs, you will use CNG in the next chapter. To do this, you have to save the EDCC output by selecting "File"->"Save this result as a text file..." in the menu (for example tutorial_output.txt).

**3.4 Categorizing potential CRMs with CNG**

Run "network_generator_gui.py" to open CNG's GUI. Just like in EDCC, you can see many options to change the categorization. As only the most important actions are explained in this chapter, you can find an explanation of all other settings in [Chapter 4.2](file:///E:\Manuscripts\CRM\Revision\submit\docs\manual.html#overview-cng).

Click on "Change..." next to the "Positive sequences" label (i.e., the sequences that shall be included in a categorization) and select the output file which was [generated by EDCC](file:///E:\Manuscripts\CRM\Revision\submit\docs\manual.html#tutorial-edcc) (tutorial_output.txt). Then click on "Start" in the bottom left and let CNG run until a pop-up appears noting that the categorization has finished. Note that while the categorization is running, you can see its current status. For more information about the categorization method itself, read the associated publication (!) of this program suite.

Now, save the results of this categorization by clicking on "Create and show total result in folder..." and choose an output folder of your choice (in order to prevent potential overwriting of existing files, an empty folder is recommended). After the generation of this total result report completed, your standard web browser will pop up and show you the "index.html" result file, which is located in the selected output folder (HTML is the standard markup language for internet pages). "index.html" contains an overview of the total categorization process. The numbered HTML files, which are linked at the bottom of "index.html", contain a detailed categorization report for each network, including diagrams and tables about the categorized sequence's features. Just like "index.html", all other HTML files are located in the selected output folder.

One example result report is included in */data/tutorial/example_report.zip*. This report was created by categorizing the tutorial_output.txt, created in the previous section.

**3.5 Reusing generated categorization networks with CNG Viewer**

In order to reuse categorization networks generated by CNG, you can use the CNG Viewer. CNG Viewer runs the categorization - according to the selected network - with a new given list of EDCC outputs of potential CRMs.

As an example, we will use the 8th network of our example report created in the previous section (in */data/tutorial/example_report.zip* the file 8.ann). For convenience, this network is already present in the */data/tutorial/* folder as to_check.ann. The potential CRMs we want to categorize are present in */data/tutorial/to_check.txt*, which is the output file of another run with the same parameters as in [Chapter 3.2](file:///E:\Manuscripts\CRM\Revision\submit\docs\manual.html#tutorial-edcc)

Run "network_viewer_gui.py" to open CRM Viewer's GUI. The files we want to use for the categorization are preselected. If you want to use other files, click on the first "Change..." button to select your network file (.ann) and click on the second "Change" button to select the EDCC output file with the potential CRMs. Press "Check" to run the categorization.

After the categorization process, all CRMs which fit in the given network's categorization are shown in the GUI's text field. In order to save the results as a text file, click on "Save fitting results" and select the text file's location. Just like EDCC's output, CNG can use CNG Viewer's output.

**3.6 Mutating a CRM of interest**

If you have found a CRM that attracts your attention, you may be interested to test how conserved your CRM is, i.e. if one CRE of the CRM commits more to the circadian regulation than the other. An easy approach to this question is to mutate a given CRM multiple times and test if these mutated CRMs still pass the EDCC threshold.

For the generation of mutated CRMs we provide a python script (mutation.py), that generates a unique set of mutated CRMs for a given CRM. The script randomly creates a mutated version of each CRE of the CRM. A random number of mutations (up to length of CRE) are introduced into each CRE, at a random position. Using this procedure a unmutated version of a CRE with a mutated version of the other CRE can be added to your set by pure chance.

As an example we use a CRM ('ACACATG,AAAATATCT') that was analyzed in the corresponding publication of this program suite. We generate a list of one million mutated CRMs that can be used as an input for EDCC:

python mutation.py -crm ACACATG,AAAATATCT -n 1000000 -o cre_mutation.txt

A zipped possible result file can be found at */data/tutorial/cre_mutation.zip.*

***4 Overview of GUI commands***

***4.1 EDCC***

*The GUI is located in "edcc_gui.py", the CLI in "edcc_console.py".

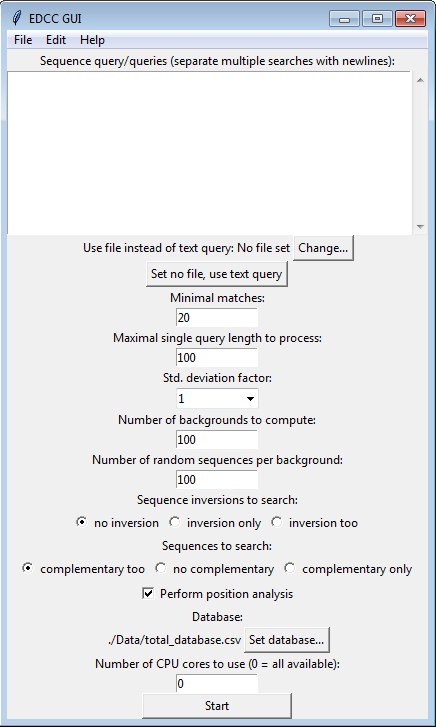

Menu:*

- *File: "Open text file as new query" loads the selected file's texts directly into the "Sequence query/queries" field. "Save query as text file" saves the current text content of the "Sequence query/queries" field into the selected text file.*
- *Edit: "Replace text" opens a dialog which permits the user to replace a given text string of the "Sequence query/queries" field with another given text string.*
- *Help: "Manual (in web browser)..." opens this manual; "About..." shows EDCC's copyright and license information.*

*Sequence query:*

- *Text field: Here, you can type in the potential CRM sequences as a DNA sequence (ambiguous code is permitted). To search for a sequence pair, you have to separate the sequences with a comma. To perform searches for multiple potential CRMs, you have to separate them with newlines.*
- *"Use file instead of text query": The text field will be ignored (Until "Set no file, use text query" is pressed), and the text file's content will be used as query.*

*CRM search and analyses parameters:*

- *Minimal matches: The minimal number of occurrences of the searched query which is necessary to display it in the search result list.*
- *Maximal single query length to process: The maximal allowed query length.*
- *Std. deviation factor: The standard deviation factor difference between the searched sequence and the background distributions which is necessary to display the searched sequence in the search result list.*
- *Number of backgrounds to compute: The number of background distributions, which are made of random sequences.*
- *Number of random sequences per background: The number of the random sequences à distribution.*
- *Sequence inversions to search: Determines if inverted versions (e.g. ATCG->GCTA) of the searched sequences shall be searched.*
- *Sequences to search: Determines if complementary versions (e.g. A->T and C->G) of the searched sequences shall be searched.*
- *Perform position analysis: Determines if the statistical position, order and distance analyses shall be performed. The order and distance test only work for sequence pairs. The test results are shown in the result window which pops up after the CRM search and analyses are finished.*
- *Database: Determines the CSV database (as described in*[*Installation*](file:///E:\Manuscripts\CRM\Revision\submit\docs\manual.html#install)*).*
- *Number of CPU cores to use: As the sequence search is implemented as a multi-threadable method, the user may choose how many of the computer's CPU cores shall be used for the CRM search and analyses.*
- *Start/Stop: Starts the CRM search and (if chosen) analyses the results. Changes to a "Stop" Button when the computation has been started, which (hard) stops the computation and closes the program. After the search is completed, a result window pops up. In its text field, a new CSV file which contains all identified potential CRM and their statistical test results are given. Via the menu entry "File", the pop-up window provides a way to save the text content in a text file. This resulting CSV can be used with CNG (see the next chapter).*

***4.2 CNG***

*The GUI is located in "network_generator_gui.py".

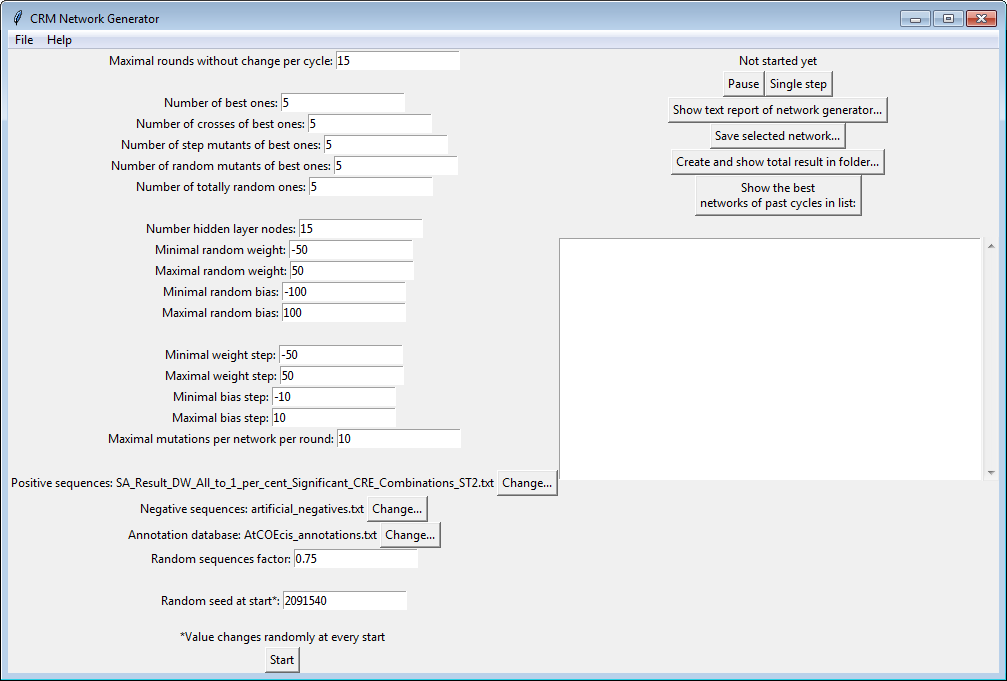

Menu:*

- *File: "Load/Save current network generator state as file" can be used to pause a training process, and to start it again even if CNG was closed in the meantime. "Exit" closes CNG.*
- *Help: "Manual (in web browser)..." opens this manual; "About..." shows CNG's copyright and license information.*

*Two-class neural network and training settings (These settings cannot be changed after "Start" is clicked):*

- *Maximal rounds without change per cycle: If no change of the rating or the number of categorized sequences of cycle's best network occurred in the given amount of rounds, the cycle stops and the best network is stored.*
- *Number of best ones: The number of the best networks of the last round which are kept for next round. In the first round of a cycle, these networks are random ones.*
- *Number of crosses of best ones: Crosses (i.e., one or more neurons of one network are put into another one) of best networks per round. In the first round of a cycle, these networks are random ones.*
- *Number of step mutants of best ones: Mutants with a small change compared to the best one from which they are derived (see step settings below) per round. In the first round of a cycle, these networks are random ones.*
- *Number of random mutants of best ones: Mutants with random change of a neuron (see "random" settings below) per round. In the first round of a cycle, these networks are random ones.*
- *Number of totally random ones: Their values are set by the "random" settings below.*
- *Number hidden layer nodes: Number of nodes à network in their hidden layer.*
- *Minimal/maximal random weight: The minimal/maximal random node weight value given to a randomized mutant.*
- *Minimal/maximal random bias: The minimal/maximal random bias value given to a randomized mutant.*
- *Minimal/maximal random weight step: The minimal/maximal random node weight step value given to a "step" mutant.*
- *Minimal/maximal random bias step: The minimal/maximal random node bias step value given to a "step" mutant.*
- *Maximal mutations per network per round: These mutations include random ones, crosses and random steps.*
- *Positive sequences: CGN creates compatible positive sequence databases.*
- *Negative sequences: Text file database in the CNG output format containing all sequences which must not be categorized.*
- *Annotation database (Optional): CSV (using the semicolon as separator)*
- *Random sequences factor: Number of random sequences in comparison to the number of positive and negative sequences.*
- *Random seed at start: Using the same seed with the same settings and datasets should lead to the same results.*
- *Start: Starts the training cycles. The status of the training will be shown in the text label which is by default "Not started yet".*

*Actions during training run:*

- *Pause: Pauses the training until this button is clicked again.*
- *Single step: If the training is paused, the training will proceed with one more round.*
- *Save selected network...: Saves a binary file including the instance of the selected network (shown in the list below if "Show the best networks of the past cycles in list" was clicked). This binary can be loaded in Python 3 or with CNG Viewer (see below).*
- *Create and show total result in folder: Saves a binary of the network generation process and the single sequences (the latter can be loaded with CNG Viewer) in the selected folder. Additionally, HTML and text files showing information, plots and gene lists of the network generation and the single networks will be stored. The "index.html" containing the network generation information will be opened by the system's default browser.*
- *Show the best networks of the past cycles in list: Shows the best sequences in the list below. Double-clicking on one of the entries shows some of its properties.*

***4.3 CNG Viewer***

*The GUI is located in "network_viewer.py".

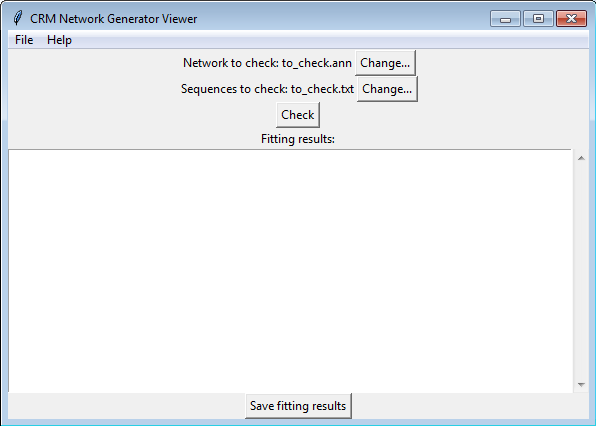

Menu:*

- *File: "Exit" closes CNG Viewer.*
- *Help: "Manual (in web browser)..." opens this manual; "About..." shows CNG Viewer's copyright and license information.*

*Loading and running of a network:*

- *Network to check: The specified network (in form of a binary file, as generated by CNG) is used for the categorization.*
- *Sequences to check: The CSV of CRMs (as generated by EDCC) to be categorized by CNG.*
- *Check: Start the categorization, the results will be shown in the text field under "Fitting results" in the form of all sequences which fit into the network's categorization.*
- *Save fitting results: Saves the text field content in the specified text file. This file can be used by CNG again.*

***Addendum: GNU Free Documentation License***

*GNU Free Documentation License
Version 1.3, 3 November 2008
Copyright © 2000, 2001, 2002, 2007, 2008 Free Software Foundation, Inc.

Everyone is permitted to copy and distribute verbatim copies of this license document, but changing it is not allowed.

0. PREAMBLE
The purpose of this License is to make a manual, textbook, or other functional and useful document "free" in the sense of freedom: to assure everyone the effective freedom to copy and redistribute it, with or without modifying it, either commercially or noncommercially. Secondarily, this License preserves for the author and publisher a way to get credit for their work, while not being considered responsible for modifications made by others. This License is a kind of "copyleft", which means that derivative works of the document must themselves be free in the same sense. It complements the GNU General Public License, which is a copyleft license designed for free software. We have designed this License in order to use it for manuals for free software, because free software needs free documentation: a free program should come with manuals providing the same freedoms that the software does. But this License is not limited to software manuals; it can be used for any textual work, regardless of subject matter or whether it is published as a printed book. We recommend this License principally for works whose purpose is instruction or reference. 

1. APPLICABILITY AND DEFINITIONS
This License applies to any manual or other work, in any medium, that contains a notice placed by the copyright holder saying it can be distributed under the terms of this License. Such a notice grants a world-wide, royalty-free license, unlimited in duration, to use that work under the conditions stated herein. The "Document", below, refers to any such manual or work. Any member of the public is a licensee, and is addressed as "you". You accept the license if you copy, modify or distribute the work in a way requiring permission under copyright law. A "Modified Version" of the Document means any work containing the Document or a portion of it, either copied verbatim, or with modifications and/or translated into another language. A "Secondary Section" is a named appendix or a front-matter section of the Document that deals exclusively with the relationship of the publishers or authors of the Document to the Document's overall subject (or to related matters) and contains nothing that could fall directly within that overall subject. (Thus, if the Document is in part a textbook of mathematics, a Secondary Section may not explain any mathematics.) The relationship could be a matter of historical connection with the subject or with related matters, or of legal, commercial, philosophical, ethical or political position regarding them. The "Invariant Sections" are certain Secondary Sections whose titles are designated, as being those of Invariant Sections, in the notice that says that the Document is released under this License. If a section does not fit the above definition of Secondary then it is not allowed to be designated as Invariant. The Document may contain zero Invariant Sections. If the Document does not identify any Invariant Sections then there are none. The "Cover Texts" are certain short passages of text that are listed, as Front-Cover Texts or Back-Cover Texts, in the notice that says that the Document is released under this License. A Front-Cover Text may be at most 5 words, and a Back-Cover Text may be at most 25 words. A "Transparent" copy of the Document means a machine-readable copy, represented in a format whose specification is available to the general public, that is suitable for revising the document straightforwardly with generic text editors or (for images composed of pixels) generic paint programs or (for drawings) some widely available drawing editor, and that is suitable for input to text formatters or for automatic translation to a variety of formats suitable for input to text formatters. A copy made in an otherwise Transparent file format whose markup, or absence of markup, has been arranged to thwart or discourage subsequent modification by readers is not Transparent. An image format is not Transparent if used for any substantial amount of text. A copy that is not "Transparent" is called "Opaque". Examples of suitable formats for Transparent copies include plain ASCII without markup, Texinfo input format, LaTeX input format, SGML or XML using a publicly available DTD, and standard-conforming simple HTML, PostScript or PDF designed for human modification. Examples of transparent image formats include PNG, XCF and JPG. Opaque formats include proprietary formats that can be read and edited only by proprietary word processors, SGML or XML for which the DTD and/or processing tools are not generally available, and the machine-generated HTML, PostScript or PDF produced by some word processors for output purposes only. The "Title Page" means, for a printed book, the title page itself, plus such following pages as are needed to hold, legibly, the material this License requires to appear in the title page. For works in formats which do not have any title page as such, "Title Page" means the text near the most prominent appearance of the work's title, preceding the beginning of the body of the text. The "publisher" means any person or entity that distributes copies of the Document to the public. A section "Entitled XYZ" means a named subunit of the Document whose title either is precisely XYZ or contains XYZ in parentheses following text that translates XYZ in another language. (Here XYZ stands for a specific section name mentioned below, such as "Acknowledgements", "Dedications", "Endorsements", or "History".) To "Preserve the Title" of such a section when you modify the Document means that it remains a section "Entitled XYZ" according to this definition. The Document may include Warranty Disclaimers next to the notice which states that this License applies to the Document. These Warranty Disclaimers are considered to be included by reference in this License, but only as regards disclaiming warranties: any other implication that these Warranty Disclaimers may have is void and has no effect on the meaning of this License. 

2. VERBATIM COPYING
You may copy and distribute the Document in any medium, either commercially or noncommercially, provided that this License, the copyright notices, and the license notice saying this License applies to the Document are reproduced in all copies, and that you add no other conditions whatsoever to those of this License. You may not use technical measures to obstruct or control the reading or further copying of the copies you make or distribute. However, you may accept compensation in exchange for copies. If you distribute a large enough number of copies you must also follow the conditions in section 3. You may also lend copies, under the same conditions stated above, and you may publicly display copies. 

3. COPYING IN QUANTITY
If you publish printed copies (or copies in media that commonly have printed covers) of the Document, numbering more than 100, and the Document's license notice requires Cover Texts, you must enclose the copies in covers that carry, clearly and legibly, all these Cover Texts: Front-Cover Texts on the front cover, and Back-Cover Texts on the back cover. Both covers must also clearly and legibly identify you as the publisher of these copies. The front cover must present the full title with all words of the title equally prominent and visible. You may add other material on the covers in addition. Copying with changes limited to the covers, as long as they preserve the title of the Document and satisfy these conditions, can be treated as verbatim copying in other respects. If the required texts for either cover are too voluminous to fit legibly, you should put the first ones listed (as many as fit reasonably) on the actual cover, and continue the rest onto adjacent pages. If you publish or distribute Opaque copies of the Document numbering more than 100, you must either include a machine-readable Transparent copy along with each Opaque copy, or state in or with each Opaque copy a computer-network location from which the general network-using public has access to download using public-standard network protocols a complete Transparent copy of the Document, free of added material. If you use the latter option, you must take reasonably prudent steps, when you begin distribution of Opaque copies in quantity, to ensure that this Transparent copy will remain thus accessible at the stated location until at least one year after the last time you distribute an Opaque copy (directly or through your agents or retailers) of that edition to the public. It is requested, but not required, that you contact the authors of the Document well before redistributing any large number of copies, to give them a chance to provide you with an updated version of the Document. 

4. MODIFICATIONS
You may copy and distribute a Modified Version of the Document under the conditions of sections 2 and 3 above, provided that you release the Modified Version under precisely this License, with the Modified Version filling the role of the Document, thus licensing distribution and modification of the Modified Version to whoever possesses a copy of it. In addition, you must do these things in the Modified Version: 

A. Use in the Title Page (and on the covers, if any) a title distinct from that of the Document, and from those of previous versions (which should, if there were any, be listed in the History section of the Document). You may use the same title as a previous version if the original publisher of that version gives permission.
B. List on the Title Page, as authors, one or more persons or entities responsible for authorship of the modifications in the Modified Version, together with at least five of the principal authors of the Document (all of its principal authors, if it has fewer than five), unless they release you from this requirement.
C. State on the Title page the name of the publisher of the Modified Version, as the publisher.
D. Preserve all the copyright notices of the Document.
E. Add an appropriate copyright notice for your modifications adjacent to the other copyright notices.
F. Include, immediately after the copyright notices, a license notice giving the public permission to use the Modified Version under the terms of this License, in the form shown in the Addendum below.
G. Preserve in that license notice the full lists of Invariant Sections and required Cover Texts given in the Document's license notice.
H. Include an unaltered copy of this License.
I. Preserve the section Entitled "History", Preserve its Title, and add to it an item stating at least the title, year, new authors, and publisher of the Modified Version as given on the Title Page. If there is no section Entitled "History" in the Document, create one stating the title, year, authors, and publisher of the Document as given on its Title Page, then add an item describing the Modified Version as stated in the previous sentence.
J. Preserve the network location, if any, given in the Document for public access to a Transparent copy of the Document, and likewise the network locations given in the Document for previous versions it was based on. These may be placed in the "History" section. You may omit a network location for a work that was published at least four years before the Document itself, or if the original publisher of the version it refers to gives permission.
K. For any section Entitled "Acknowledgements" or "Dedications", Preserve the Title of the section, and preserve in the section all the substance and tone of each of the contributor acknowledgements and/or dedications given therein.
L. Preserve all the Invariant Sections of the Document, unaltered in their text and in their titles. Section numbers or the equivalent are not considered part of the section titles.
M. Delete any section Entitled "Endorsements". Such a section may not be included in the Modified Version.
N. Do not retitle any existing section to be Entitled "Endorsements" or to conflict in title with any Invariant Section.
O. Preserve any Warranty Disclaimers.

If the Modified Version includes new front-matter sections or appendices that qualify as Secondary Sections and contain no material copied from the Document, you may at your option designate some or all of these sections as invariant. To do this, add their titles to the list of Invariant Sections in the Modified Version's license notice. These titles must be distinct from any other section titles. You may add a section Entitled "Endorsements", provided it contains nothing but endorsements of your Modified Version by various parties—for example, statements of peer review or that the text has been approved by an organization as the authoritative definition of a standard. You may add a passage of up to five words as a Front-Cover Text, and a passage of up to 25 words as a Back-Cover Text, to the end of the list of Cover Texts in the Modified Version. Only one passage of Front-Cover Text and one of Back-Cover Text may be added by (or through arrangements made by) any one entity. If the Document already includes a cover text for the same cover, previously added by you or by arrangement made by the same entity you are acting on behalf of, you may not add another; but you may replace the old one, on explicit permission from the previous publisher that added the old one. The author(s) and publisher(s) of the Document do not by this License give permission to use their names for publicity for or to assert or imply endorsement of any Modified Version. 

5. COMBINING DOCUMENTS
You may combine the Document with other documents released under this License, under the terms defined in section 4 above for modified versions, provided that you include in the combination all of the Invariant Sections of all of the original documents, unmodified, and list them all as Invariant Sections of your combined work in its license notice, and that you preserve all their Warranty Disclaimers. The combined work need only contain one copy of this License, and multiple identical Invariant Sections may be replaced with a single copy. If there are multiple Invariant Sections with the same name but different contents, make the title of each such section unique by adding at the end of it, in parentheses, the name of the original author or publisher of that section if known, or else a unique number. Make the same adjustment to the section titles in the list of Invariant Sections in the license notice of the combined work. In the combination, you must combine any sections Entitled "History" in the various original documents, forming one section Entitled "History"; likewise combine any sections Entitled "Acknowledgements", and any sections Entitled "Dedications". You must delete all sections Entitled "Endorsements". 

6. COLLECTIONS OF DOCUMENTS
You may make a collection consisting of the Document and other documents released under this License, and replace the individual copies of this License in the various documents with a single copy that is included in the collection, provided that you follow the rules of this License for verbatim copying of each of the documents in all other respects. You may extract a single document from such a collection, and distribute it individually under this License, provided you insert a copy of this License into the extracted document, and follow this License in all other respects regarding verbatim copying of that document. 

7. AGGREGATION WITH INDEPENDENT WORKS A compilation of the Document or its derivatives with other separate and independent documents or works, in or on a volume of a storage or distribution medium, is called an "aggregate" if the copyright resulting from the compilation is not used to limit the legal rights of the compilation's users beyond what the individual works permit. When the Document is included in an aggregate, this License does not apply to the other works in the aggregate which are not themselves derivative works of the Document. If the Cover Text requirement of section 3 is applicable to these copies of the Document, then if the Document is less than one half of the entire aggregate, the Document's Cover Texts may be placed on covers that bracket the Document within the aggregate, or the electronic equivalent of covers if the Document is in electronic form. Otherwise they must appear on printed covers that bracket the whole aggregate. 

8. TRANSLATION
Translation is considered a kind of modification, so you may distribute translations of the Document under the terms of section 4. Replacing Invariant Sections with translations requires special permission from their copyright holders, but you may include translations of some or all Invariant Sections in addition to the original versions of these Invariant Sections. You may include a translation of this License, and all the license notices in the Document, and any Warranty Disclaimers, provided that you also include the original English version of this License and the original versions of those notices and disclaimers. In case of a disagreement between the translation and the original version of this License or a notice or disclaimer, the original version will prevail. If a section in the Document is Entitled "Acknowledgements", "Dedications", or "History", the requirement (section 4) to Preserve its Title (section 1) will typically require changing the actual title. 

9. TERMINATION
You may not copy, modify, sublicense, or distribute the Document except as expressly provided under this License. Any attempt otherwise to copy, modify, sublicense, or distribute it is void, and will automatically terminate your rights under this License. However, if you cease all violation of this License, then your license from a particular copyright holder is reinstated (a) provisionally, unless and until the copyright holder explicitly and finally terminates your license, and (b) permanently, if the copyright holder fails to notify you of the violation by some reasonable means prior to 60 days after the cessation. Moreover, your license from a particular copyright holder is reinstated permanently if the copyright holder notifies you of the violation by some reasonable means, this is the first time you have received notice of violation of this License (for any work) from that copyright holder, and you cure the violation prior to 30 days after your receipt of the notice. Termination of your rights under this section does not terminate the licenses of parties who have received copies or rights from you under this License. If your rights have been terminated and not permanently reinstated, receipt of a copy of some or all of the same material does not give you any rights to use it. 

10. FUTURE REVISIONS OF THIS LICENSE
The Free Software Foundation may publish new, revised versions of the GNU Free Documentation License from time to time. Such new versions will be similar in spirit to the present version, but may differ in detail to address new problems or concerns. See http://www.gnu.org/copyleft/. Each version of the License is given a distinguishing version number. If the Document specifies that a particular numbered version of this License "or any later version" applies to it, you have the option of following the terms and conditions either of that specified version or of any later version that has been published (not as a draft) by the Free Software Foundation. If the Document does not specify a version number of this License, you may choose any version ever published (not as a draft) by the Free Software Foundation. If the Document specifies that a proxy can decide which future versions of this License can be used, that proxy's public statement of acceptance of a version permanently authorizes you to choose that version for the Document. 

11. RELICENSING
"Massive Multiauthor Collaboration Site" (or "MMC Site") means any World Wide Web server that publishes copyrightable works and also provides prominent facilities for anybody to edit those works. A public wiki that anybody can edit is an example of such a server. A "Massive Multiauthor Collaboration" (or "MMC") contained in the site means any set of copyrightable works thus published on the MMC site. "CC-BY-SA" means the Creative Commons Attribution-Share Alike 3.0 license published by Creative Commons Corporation, a not-for-profit corporation with a principal place of business in San Francisco, California, as well as future copyleft versions of that license published by that same organization. "Incorporate" means to publish or republish a Document, in whole or in part, as part of another Document. An MMC is "eligible for relicensing" if it is licensed under this License, and if all works that were first published under this License somewhere other than this MMC, and subsequently incorporated in whole or in part into the MMC, (1) had no cover texts or invariant sections, and (2) were thus incorporated prior to November 1, 2008. The operator of an MMC Site may republish an MMC contained in the site under CC-BY-SA on the same site at any time before August 1, 2009, provided the MMC is eligible for relicensing.*
